# Supplementary material for: Enantioselective nickel-catalyzed anodic oxidative dienylation and allylation reactions
Source: Nat Commun. 2024 May 25;15:4477. doi: 10.1038/s41467-024-48936-4 (PMC11127924; doi:10.1038/s41467-024-48936-4)
Supplement: Supplementary file 1 — Supplementary information [file 41467_2024_48936_MOESM1_ESM.pdf]

Enantioselective Nickel-Catalyzed Anodic Oxidative Dienylation and Allylation Reactions

Qinglin Zhang[1], Jiayin Zhang[1], Wangjie Zhu[1], Ruimin Lu[1] & Chang Guo[1]*

[1]Hefei National Research Center for Physical Sciences at the Microscale and Department of Chemistry, University of Science and Technology of China, 230026 Hefei, China

*Email: guochang@ustc.edu.cn

Table of Contents

1. General information **S2**

2. Preparation of substrates **S2**

3. Photographic guide for electrochemical reactions **S16**

4. Optimization studies **S17**

5. Synthesis and characterization of products **S23**

6. Large-scale reaction and transformation of products **S39**

7. Evaluation of chiral Boox ligands **S48**

8. Cyclic voltammetry studies **S48**

9. Controlled experiments **S51**

10. Computational Study. **S62**

11. X-ray crystallography data **S71**

12. NMR spectra **S74**

13. HPLC traces **S160**

14. References **S229**

1. General information

Unless otherwise noted, All the electrochemical reactions were performed in an undivided cell equipped with carbon rod (d = 6 mm) as the anode and platinum plate (1.0 cm × 1.0 cm × 0.2 mm) as the cathode. all reagents were purchased from commercial suppliers and used without further purification. Cyclic voltammograms were recorded on a CHI 760E potentiostat. ^{1}H NMR and ^{13}C NMR spectra were recorded at 25 °C on Bruker Advance 400M NMR spectrometers, Bruker Advance 500M NMR spectrometers and Bruker Advance 600M NMR spectrometers. Chemical shifts for ^{1}H NMR spectra are reported as δ in units of parts per million (ppm) downfield from $SiMe_4$ (δ 0.00) and relative to the signal of chloroform-*d* (δ 7.26, singlet). Multiplicities were given as: s (singlet); d (doublet); t (triplet); q (quartet); dd (doublet of doublets); dt (doublet of triplets); m (multiplets), etc. Coupling constants are reported as a *J* value in Hz. ^{13}C NMR spectra are reported as δ in units of parts per million (ppm) downfield from $SiMe_4$ (δ 0.00) and relative to the signal of chloroform-*d* (δ 77.16, triplet). High-resolution mass spectral analysis (HRMS) was performed on Waters XEVO G2 Q-TOF. Optical rotations were determined at 589 nm (sodium D line) by using a Perkin-Elmer-343 polarimeter. The measurement of enantiomeric excesses was performed on Waters-Alliance (2998, Photodiode Array Detector). CHIRALCEL IA, IB N-5, IC, IE, IF, and IG columns were purchased from Daicel Chemical Industries, LTD.

2. Preparation of substrates

Starting materials were purchased from commercial suppliers (Aldrich, Acros, TCI, Adamas-beta, Energy etc.) and used as supplied unless otherwise stated.

2.1 Preparation of benzoxazolyl acetates

Benzoxazolyl acetates **1a**, **S1a**, **S1b**, **S1d-S1h**, **S1k**, **S1l**, **S1t**, **S1w-S1z**, **S1ac-S1ag** were prepared by following the literature report[1-3].

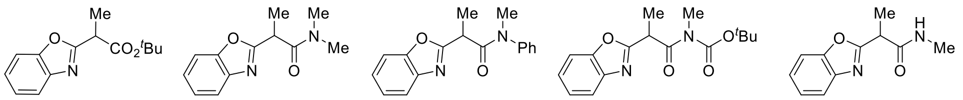

1a **S1a** **S1b** **S1c** **S1d**

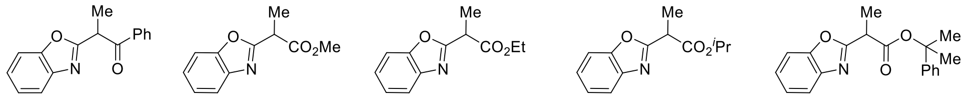

S1e **S1f** **S1g** **S1h** **S1i**

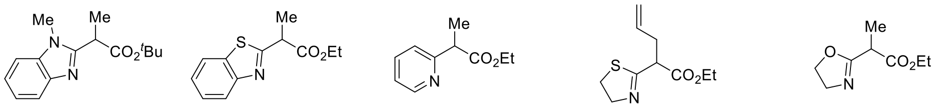

S1j **S1k** **S1l** **S1m** **S1n**

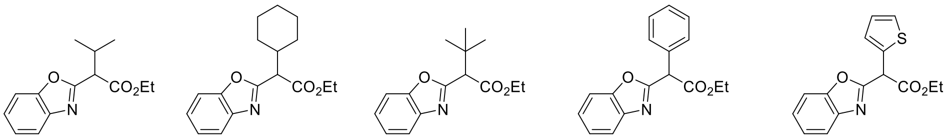

S1o **S1p** **S1q** **S1r** **S1s**

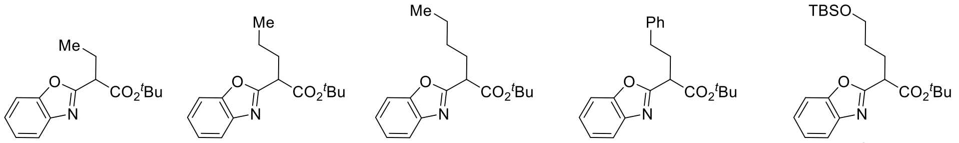

S1t **S1u** **S1v** **S1w** **S1x**

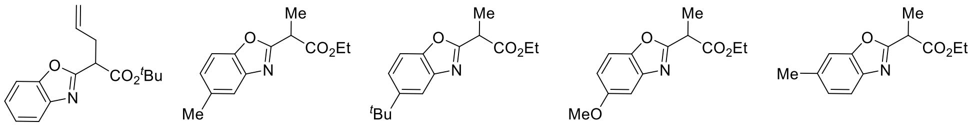

S1y **S1z** **S1aa** **S1ab** **S1ac**

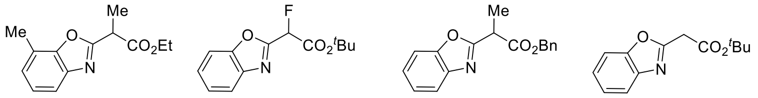

S1ad **S1ae** **S1af** **S1ag**

2.1.1 Preparation of benzoxazolyl acetates S1c, S1o-S1s [1-3]

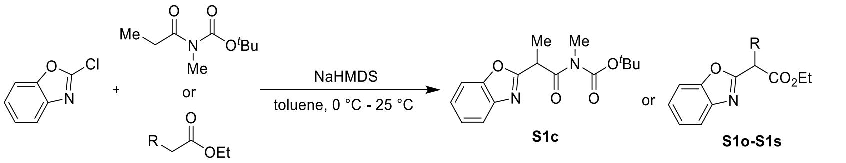

To a solution of NaHMDS (10 mL, 2 M in THF, 20 mmol, 2.0 equiv.) in dry toluene (20 mL) at 0 °C was added amide or ester (20 mmol, 2.0 equiv.) dropwise. After being stirred at 0 °C for 30 minutes, 2-chlorobenzoxazole (1.54 g, 10 mmol, 1.0 equiv.) was added at 0 °C. The reaction mixture was allowed to warm to room temperature and stirred for 3 hours. After completion, saturated aqueous NH_4Cl was added and the aqueous layer was extracted with EtOAc (20 mL × 3). The combined organic layers were washed with brine, dried over Na_2SO_4, filtered and concentrated in vacuo. The crude product was purified by flash chromatography to afford the desired product **S1c, S1o-S1s**.

Tert-butyl (2-(benzo[d]oxazol-2-yl)propanoyl)(methyl)carbamate (S1c)

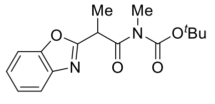

^{1}H NMR (400 MHz, $CDCl_3$) δ 7.76 – 7.64 (m, 1H), 7.56 – 7.45 (m, 1H), 7.35 – 7.22 (m, 2H), 5.25 (q, J = 7.0 Hz, 1H), 3.25 (s, 3H), 1.72 (d, J = 7.1 Hz, 3H), 1.43 (s, 9H). **^{13}C NMR (100 MHz, $CDCl_3$)** δ 173.08, 165.75, 153.06, 150.86, 141.19, 124.81, 124.27, 120.01, 110.60, 83.81, 42.16, 32.25, 27.94, 16.02. **ESI-MS**: calculated [$C_{16}H_{20}N_2O_4$ +

H]$^+$**:** 305.1496, found: 305.1497**.**

Ethyl 2-(benzo[d]oxazol-2-yl)-3-methylbutanoate (S1o)

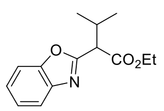

^{1}H NMR (400 MHz, $CDCl_3$) δ 7.79 – 7.65 (m, 1H), 7.61 – 7.48 (m, 1H), 7.43 – 7.30 (m, 2H), 4.32 – 4.12 (m, 2H), 3.80 (d, *J* = 9.0 Hz, 1H), 2.80 – 2.65 (m, 1H), 1.26 (t, *J* = 7.1 Hz, 3H), 1.11 (d, *J* = 6.7 Hz, 3H), 1.00 (d, *J* = 6.7 Hz, 3H). **^{13}C NMR (100 MHz, $CDCl_3$)** δ 169.39, 162.89, 150.96, 141.09, 125.12, 124.46, 120.23, 110.80, 61.65, 53.75, 30.36, 20.85, 20.46, 14.24. **ESI-MS**: calculated [$C_{14}H_{17}NO_3$ + H]$^+$: 248.1281, found: 248.1281**.**

Ethyl 2-(benzo[d]oxazol-2-yl)-2-cyclohexylacetate (S1p)

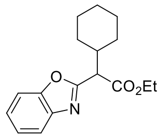

^{1}H NMR (400 MHz, $CDCl_3$) δ 7.79 – 7.66 (m, 1H), 7.59 – 7.48 (m, 1H), 7.38 – 7.27 (m, 2H), 4.29 – 4.12 (m, 2H), 3.82 (d, *J* = 9.5 Hz, 1H), 2.46 – 2.31 (m, 1H), 1.92 – 1.82 (m, 1H), 1.80 – 1.73 (m, 1H), 1.72 – 1.60 (m, 2H), 1.60 – 1.50 (m, 1H), 1.42 – 1.27 (m, 2H), 1.24 (t, *J* = 7.1 Hz, 3H), 1.21 – 1.03 (m, 3H). **^{13}C NMR (100 MHz, $CDCl_3$)** δ 169.32, 162.77, 150.98, 141.12, 125.08, 124.45, 120.21, 110.82, 61.64, 53.00, 39.45, 31.29, 30.75, 26.10, 26.02, 25.95, 14.26. **ESI-MS**: calculated [$C_{17}H_{21}NO_3$ + H]$^+$: 288.1594, found: 288.1594**.**

Ethyl 2-(benzo[d]oxazol-2-yl)-3,3-dimethylbutanoate (S1q)

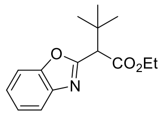

^{1}H NMR (400 MHz, $CDCl_3$) δ 7.80 – 7.68 (m, 1H), 7.62 – 7.49 (m, 1H), 7.40 – 7.29 (m, 2H), 4.31 – 4.15 (m, 2H), 3.95 (s, 1H), 1.26 (t, *J* = 7.1 Hz, 3H), 1.19 (s, 9H). **^{13}C NMR (100 MHz, $CDCl_3$)** δ 168.74, 162.60, 150.86, 141.04, 125.05, 124.41, 120.26, 110.78, 61.33, 56.48, 35.35, 28.30, 14.27. **ESI-MS**: calculated [$C_{15}H_{19}NO_3$ + H]$^+$: 262.1438, found: 262.1438**.**

Ethyl 2-(benzo[d]oxazol-2-yl)-2-phenylacetate (S1r)

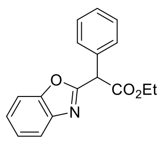

^{1}H NMR (400 MHz, $CDCl_3$) δ 7.81 – 7.69 (m, 1H), 7.58 – 7.49 (m, 3H), 7.45 – 7.36 (m, 3H), 7.35 – 7.29 (m, 2H), 5.34 (s, 1H), 4.36 – 4.21 (m, 2H), 1.26 (t, *J* = 7.1 Hz, 3H). **^{13}C NMR (100 MHz, $CDCl_3$)** δ 168.61, 162.42, 151.17, 141.12, 133.61, 129.23, 129.01, 128.56, 125.30, 124.53, 120.44, 110.85, 62.33, 52.46, 14.15. **ESI-MS**: calculated [$C_{17}H_{15}NO_3$ + H]$^+$: 282.1125, found: 282.1126**.**

Ethyl 2-(benzo[d]oxazol-2-yl)-2-(thiophen-2-yl)acetate (S1s)

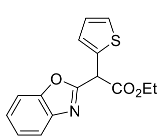

^{1}H NMR (400 MHz, $CDCl_3$) δ 7.78 – 7.71 (m, 1H), 7.56 – 7.48 (m, 1H), 7.38 – 7.30 (m, 3H), 7.24 – 7.20 (m, 1H), 7.05 – 6.99 (m, 1H), 5.63 (s, 1H), 4.28 (q, *J* = 7.1 Hz, 2H), 1.27 (t, *J* = 7.1 Hz, 3H). **^{13}C NMR (100 MHz, $CDCl_3$)** δ 167.70, 161.63, 151.15, 141.04, 134.28, 128.15, 126.95, 126.68, 125.48, 124.65, 120.55, 110.92, 62.71, 47.51, 14.10. **ESI-MS**: calculated [$C_{15}H_{13}NO_3S$ + H]$^+$: 288.0689, found: 288.0689**.**

2.1.2 Preparation of thiazole acetate S1m and benzoxazolyl acetates S1aa, S1ab[1-3]

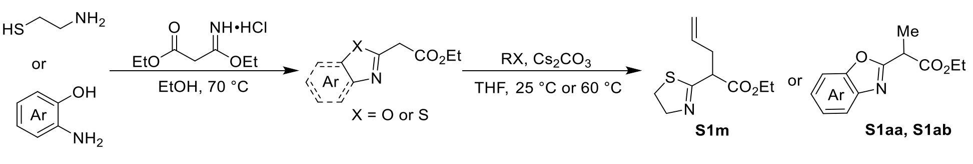

To a solution of cysteamine (20 mmol, 1.0 equiv.) or 1-amino-2-hydroxybenzene (20 mmol, 1.0 equiv.) and ethyl 3-ethoxy-3-iminopropanoate hydrochloride (20 mmol, 1.0 equiv.) in anhydrous ethanol

(40 mL) was stirred for 4 hours at 70 °C . After completion, saturated aqueous NH_4Cl was added and the aqueous layer was extracted with EtOAc (30 mL × 3). The combined organic layers were washed with brine, dried over Na_2SO_4, filtered and concentrated in vacuo. The crude product was purified by flash chromatography to afford the corresponding product.

To a solution of cesium carbonate (7.5 mmol, 1.5 equiv.) and ethyl 2-(benzo[d]oxazol-2-yl)acetate (5 mmol, 1.0 equiv.) in THF (20 mL) was added alkyl bromide or alkyl iodide (7.5 mmol, 1.5 equiv.) dropwise. The mixture was stirred at 25 °C or 60 °C until the reaction was complete (monitored by TLC). The mixture was quenched by adding H_2O at 0 °C and the aqueous layer was extracted with EtOAc (30 mL × 3). The combined organic layers were washed with brine, dried over Na_2SO_4, filtered and concentrated in vacuo. The crude product was purified by flash chromatography to afford the corresponding product **S1m**, **S1aa**, **S1ab**.

Ethyl 2-(4,5-dihydrothiazol-2-yl)pent-4-enoate (S1m)

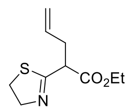

^{1}H NMR (400 MHz, CDCl$_3$) δ 5.89 – 5.71 (m, 1H), 5.14 (dd, *J* = 17.1, 1.3 Hz, 1H), 5.02 (dd, *J* = 17.2, 1.7 Hz, 1H), 4.24 (t, *J* = 8.5 Hz, 2H), 4.21 (q, *J* = 7.1 Hz, 1H), 3.66 (t, *J* = 7.6 Hz, 1H), 3.31 (t, *J* = 8.4 Hz, 2H), 2.80 – 2.68 (m, 1H), 2.66 – 2.54 (m, 1H), 1.27 (t, *J* = 7.0 Hz, 3H). **^{13}C NMR (100 MHz, CDCl$_3$)** δ 170.30, 168.28, 134.29, 117.68, 64.36, 59.28, 50.75, 34.96, 34.01, 14.26. **ESI-MS**: calculated $[C_{10}H_{15}NO_2S + H]^+$: 214.0896, found: 214.0896**.**

Ethyl 2-(5-(tert-butyl)benzo[d]oxazol-2-yl)propanoate (S1aa)

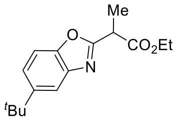

^{1}H NMR (600 MHz, CDCl$_3$) δ 7.76 – 7.73 (m, 1H), 7.45 – 7.36 (m, 2H), 4.28 – 4.17 (m, 2H), 4.10 (q, *J* = 7.3 Hz, 1H), 1.71 (d, *J* = 7.3 Hz, 3H), 1.37 (s, 9H), 1.24 (t, *J* = 7.1 Hz, 3H). **^{13}C NMR (150 MHz, CDCl$_3$)** δ 170.51, 164.31, 149.05, 148.03, 141.01, 122.85, 116.70, 109.86, 61.88, 40.77, 35.02, 31.88, 15.19, 14.20. **ESI-MS:** calculated $[C_{16}H_{21}NO_3 + H]^+$: 276.1594, found: 276.1601**.**

Ethyl 2-(5-methoxybenzo[d]oxazol-2-yl)propanoate (S1ab)

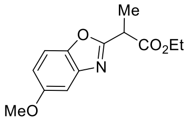

^{1}H NMR (600 MHz, CDCl$_3$) δ 7.37 (d, *J* = 8.9 Hz, 1H), 7.19 (d, *J* = 2.5 Hz, 1H), 6.91 (dd, *J* = 8.9, 2.6 Hz, 1H), 4.20 (qd, *J* = 7.1, 2.5 Hz, 2H), 4.08 (q, *J* = 7.3 Hz, 1H), 3.83 (s, 3H), 1.70 (d, *J* = 7.3 Hz, 3H), 1.23 (t, *J* = 7.1 Hz, 3H). **^{13}C NMR (150 MHz, CDCl$_3$)** δ 170.47, 164.91, 157.30, 145.65, 141.92, 113.68, 110.84, 103.14, 61.88, 56.03, 40.79, 15.13, 14.17. **ESI-MS**: calculated $[C_{13}H_{15}NO_4 + H]^+$: 250.1074, found: 250.1078**.**

2.1.3 Preparation of oxazole acetate S1n

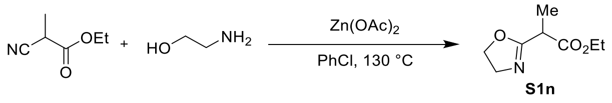

To a solution of ethyl 2-cyanopropanoate (10 mmol, 1.0 equiv.) and monoethanolamine (10 mmol, 1.0 equiv.) in PhCl (10 mL) was added $Zn(OAc)_2$ (0.4 mmol, 0.04 equiv.) at room temperature under N_2 atmosphere. The mixture was stirred vigorously and heated at 130 °C for 24 h. After cooling to rt, saturated aqueous NH_4Cl was added to the mixture and allowed to stir overnight. The organic phase was extracted twice with EtOAc. The combined organic layers were washed with brine, dried over Na_2SO_4, and concentrated in vacuo. The crude products were purified by flash column chromatography (petroleum ether/EtOAc) to afford oxazole acetates **S1n** (212 mg, 1.23 mmol, 12% yield).

Ethyl 2-(4,5-dihydrooxazol-2-yl)propanoate (S1n)

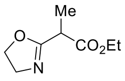

^{1}H NMR (400 MHz, $CDCl_3$) δ 4.26 (t, *J* = 9.5 Hz, 2H), 4.17 (q, *J* = 7.1 Hz, 2H), 3.84 (t, *J* = 9.5 Hz, 2H), 3.43 (q, *J* = 7.2 Hz, 1H), 1.42 (d, *J* = 7.3 Hz, 3H), 1.24 (t, *J* = 7.1 Hz, 3H). **^{13}C NMR (100 MHz, $CDCl_3$)** δ 170.97, 166.23, 67.99, 61.47, 54.38, 40.00, 14.45, 14.15. **ESI-MS**: calculated $[C_8H_{13}NO_3 + H]^+$: 194.0788, found: 194.0798.

2.1.4 Preparation of benzoxazolyl acetates S1i, S1u, S1v and benzimidazole acetate S1j[1-3]

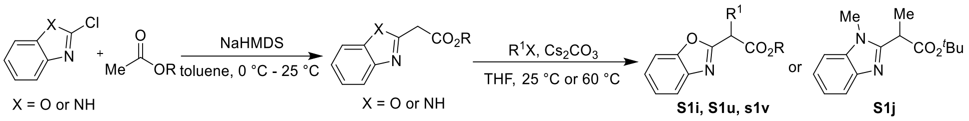

To a solution of NaHMDS (10 mL, 2 M in THF, 20 mmol, 2.0 equiv.) in dry toluene (20 mL) at 0 °C was added amide or ester (20 mmol, 2.0 equiv.) dropwise. After being stirred at 0 °C for 30 minutes, 2-chlorobenzoxazole (1.54 g, 10 mmol, 1.0 equiv.) was added at 0 °C. The reaction mixture was allowed to warm to room temperature and stirred for 3 hours. After completion, saturated aqueous NH_4Cl was added and the aqueous layer was extracted with EtOAc (20 mL × 3). The combined organic layers were washed with brine, dried over Na_2SO_4, filtered and concentrated in vacuo. The crude product was purified by flash chromatography to afford the corresponding product.

To a solution of cesium carbonate (7.5 mmol, 1.5 equiv.) and ethyl 2-(benzo[d]oxazol-2-yl)acetate (5 mmol, 1.0 equiv.) in THF (20 mL) was added alkyl bromide or alkyl iodide (7.5 mmol, 1.5 equiv.) dropwise. The mixture was stirred at 25 °C or 60 °C until the reaction was complete (monitored by TLC). The mixture was quenched by adding H_2O at 0 °C and the aqueous layer was extracted with EtOAc (30 mL × 3). The combined organic layers were washed with brine, dried over Na_2SO_4, filtered and concentrated in vacuo. The crude product was purified by flash chromatography to afford the corresponding product **S1i**, **S1j**, **S1u**, **S1v**.

2-phenylpropan-2-yl 2-(benzo[d]oxazol-2-yl)propanoate (S1i)

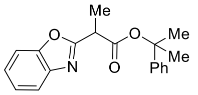

^{1}H NMR (400 MHz, $CDCl_3$) δ 7.77 – 7.70 (m, 1H), 7.57 – 7.50 (m, 1H), 7.36 – 7.32 (m, 2H), 7.28 – 7.20 (m, 5H), 4.11 (q, *J* = 7.3 Hz, 1H), 1.76 (s, 3H), 1.75 (s, 3H), 1.71 (d, *J* = 7.3 Hz, 3H). **^{13}C NMR (100 MHz, $CDCl_3$)** δ 168.77, 164.41, 151.03, 145.21, 141.16, 128.37, 127.31, 125.15, 124.48, 124.31, 120.18, 110.68, 83.48, 41.65, 28.50, 28.42, 14.98. **ESI-MS:** calculated $[C_{19}H_{19}NO_3 + H]^+$**:** 310.1438, found: 310.1439.

Tert-butyl 2-(1-methyl-1H-benzo[d]imidazol-2-yl)propanoate (S1j)

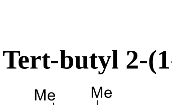

^{1}H NMR (400 MHz, $CDCl_3$) δ 7.84 – 7.74 (m, 1H), 7.36 – 7.30 (m, 1H), 7.29 – 7.24 (m, 2H), 4.01 (q, *J* = 7.2 Hz, 1H), 3.77 (s, 3H), 1.75 (d, *J* = 7.2 Hz, 3H), 1.42 (s, 9H). **^{13}C NMR (100 MHz, $CDCl_3$)** δ 170.64, 152.95, 142.49, 136.12, 122.58, 122.06, 119.89, 109.20, 82.12, 40.11, 30.16, 28.08, 15.45. **ESI-MS**: calculated $[C_{15}H_{20}N_2O_2 + H]^+$: 261.1598, found: 261.1598.

Tert-butyl 2-(benzo[d]oxazol-2-yl)pentanoate (S1u)

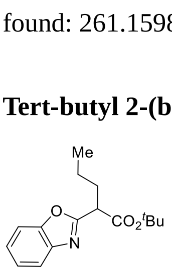

^{1}H NMR (400 MHz, $CDCl_3$) δ 7.78 – 7.66 (m, 1H), 7.57 – 7.48 (m, 1H), 7.37 – 7.26 (m, 2H), 3.94 (dd, *J* = 8.8, 6.5 Hz, 1H), 2.21 – 2.08 (m, 2H), 1.45 (s, 9H), 1.43 – 1.36 (m, 2H), 0.97 (t, *J* = 7.4 Hz, 3H). **^{13}C NMR (100 MHz, $CDCl_3$)** δ 169.03, 163.89,

150.93, 141.14, 124.89, 124.27, 120.05, 110.59, 82.18, 47.22, 32.24, 27.96, 20.64, 13.73. **ESI-MS**: calculated [$C_{16}H_{21}NO_3$ + Na]$^+$: 298.1414, found: 298.1424**.**

Tert-butyl 2-(benzo[d]oxazol-2-yl)hexanoate (S1v)

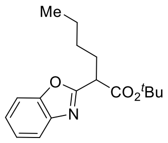

^{1}H NMR (400 MHz, $CDCl_3$) δ 7.75 – 7.67 (m, 1H), 7.54 – 7.47 (m, 1H), 7.35 – 7.28 (m, 2H), 3.90 (t, J = 7.7 Hz, 1H), 2.27 – 2.06 (m, 2H), 1.43 (s, 9H), 1.40 – 1.25 (m, 4H), 0.89 (t, J = 7.0 Hz, 3H). **^{13}C NMR (100 MHz, $CDCl_3$)** δ 169.10, 163.98, 150.98, 141.19, 124.94, 124.32, 120.11, 110.66, 82.24, 47.50, 29.98, 29.57, 28.01, 22.40, 13.91. **ESI-MS**: calculated [$C_{17}H_{23}NO_3$ + Na]$^+$: 312.1570, found: 312.1574**.**

2.2 Preparation of allenylmethylsilanes

2.2.1 Preparation of allenylmethylsilane 2a–2j[4,5]

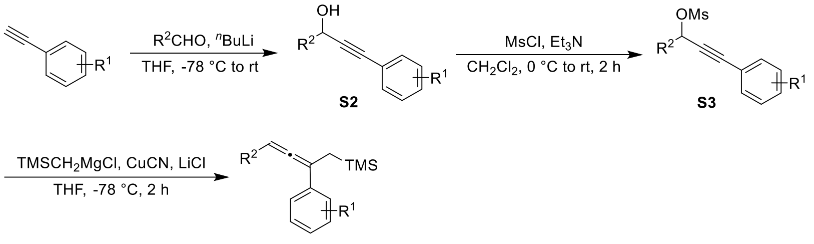

To a 100 mL round bottom flask equipped with a stir bar was added phenylacetylene (10.0 mmol, 1.0 equiv.) and THF (20 mL) and then cooled to -78 ℃. ^{n}BuLi (2.5 M in hexane, 1.0 equiv.) was added dropwise and the slightly yellow solution was stirred for 30 min. The corresponding aldehyde (10.0 mmol, 1.0 equiv.) was added and the mixture was allowed to warm to room temperature and further stirred for 1 h. After completion, the reaction was quenched with aqueous HCl (1.0 M) and then extracted with Et_2O (20 mL × 3). The combined organic layers were washed with brine (10 mL), dried over Na_2SO_4, and concentrated in vacuo. The crude product was purified by flash column chromatography (petroleum ether/EtOAc) to provide the corresponding propargylic alcohol **S2**.

To a solution of propargylic alcohol **S2** (1.0 equiv.) and triethylamine (2.0 equiv.) in CH_2Cl_2 (0.2 M) was added methanesulfonyl chloride (1.5 equiv.) dropwise at 0 ℃ under N_2 atmosphere. The reaction mixture was allowed to warm to room temperature and stirred for 2 h. After completion, saturated aqueous $NaHCO_3$ (15 mL) was added and the aqueous layer was extracted with CH_2Cl_2 (15 mL × 3). The combined organic extracts were washed with H_2O (20 mL) and brine (10 mL), dried over Na_2SO_4, and concentrated in vacuo. The crude product was purified by flash column chromatography (petroleum ether/EtOAc) to afford **S3**.

To an ice-cooled suspension of CuCN (3.0 equiv.) and LiCl (6.0 equiv.) in anhydrous THF (0.1 M) was added a solution of $TMSCH_2MgCl$ (1.3 M in THF, 2.5 equiv.). After being stirred for 25 min, the mixture was cooled to -78 ℃, and a solution of **S3** (1.0 equiv.) in THF (5 mL) was added. After 2 h, the reaction was quenched with saturated aqueous NH_4Cl (5 mL). The reaction mixture was extracted with Et_2O (20 mL × 3). The combined organic layers were washed with brine (10 mL), dried over Na_2SO_4, and concentrated in vacuo. The crude products were purified by flash column chromatography (petroleum ether/EtOAc) to afford allenylmethylsilane **2a-2j**.

(2,6-diphenylhexa-2,3-dien-1-yl)trimethylsilane (2a)

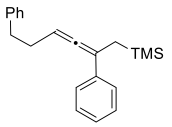

^{1}H NMR (400 MHz, CDCl$_3$) δ 7.33 – 7.27 (m, 4H), 7.26 – 7.12 (m, 6H), 5.50 – 5.36 (m, 1H), 2.86 – 2.72 (m, 2H), 2.50 – 2.38 (m, 2H), 1.75 (d, *J* = 2.4 Hz, 2H), -0.00 (s, 9H). **^{13}C NMR (100 MHz, CDCl$_3$)** δ 204.49, 141.95, 138.37, 128.74, 128.48, 128.20, 126.36, 126.28, 126.00, 103.25, 92.65, 35.88, 31.62, 19.13, -0.91. **ESI-MS**: calculated [$C_{21}H_{26}Si$ + H]$^+$: 307.1877, found: 307.1880.

Trimethyl(2-phenylpenta-2,3-dien-1-yl)silane (2b)

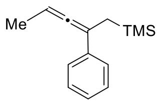

^{1}H NMR (400 MHz, CDCl$_3$) δ 7.44 – 7.38 (m, 2H), 7.33 – 7.27 (m, 2H), 7.21 – 7.15 (m, 1H), 5.47 – 5.34 (m, 1H), 1.81 (d, *J* = 2.6 Hz, 1H), 1.79 (d, *J* = 2.4 Hz, 1H), 1.77 (d, *J* = 7.0 Hz, 3H), 0.03 (s, 9H). **^{13}C NMR (100 MHz, CDCl$_3$)** δ 205.32, 138.77, 128.22, 126.37, 126.34, 102.35, 87.85, 19.10, 14.81, -1.00. **ESI-MS**: calculated [$C_{14}H_{20}Si$ + H]$^+$: 217.1407, found: 217.1405.

Trimethyl(2-phenylhepta-2,3-dien-1-yl)silane (2c)

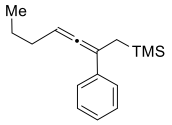

^{1}H NMR (400 MHz, CDCl$_3$) δ 7.45 – 7.40 (m, 2H), 7.36 – 7.27 (m, 3H), 7.21 – 7.15 (m, 1H), 5.50 – 5.36 (m, 1H), 2.15 – 2.08 (m, 2H), 1.88 – 1.76 (m, 2H), 1.58 – 1.47 (m, 2H), 0.99 (t, *J* = 7.4 Hz, 3H), 0.03 (s, 9H). **^{13}C NMR (100 MHz, CDCl$_3$)** δ 204.44, 138.72, 128.19, 126.28, 102.68, 93.25, 31.80, 22 .86, 19.24, 14.03, -0.90. **ESI-MS**: calculated [$C_{16}H_{24}Si$ + Na]$^+$: 267.1539, found: 267.1529.

Trimethyl(2-phenylocta-2,3-dien-1-yl)silane (2d)

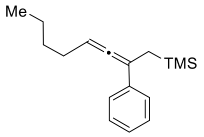

^{1}H NMR (400 MHz, CDCl$_3$) δ 7.44 – 7.38 (m, 2H), 7.33 – 7.27 (m, 2H), 7.20 – 7.14 (m, 1H), 5.52 – 5.35 (m, 1H), 2.18 – 2.09 (m, 2H), 1.88 – 1.76 (m, 2H), 1.53 – 1.44 (m, 2H), 1.43 – 1.35 (m, 2H), 0.93 (t, *J* = 7.2 Hz, 3H), 0.02 (s, 9H). **^{13}C NMR (100 MHz, CDCl$_3$)** δ 204.34, 138.74, 128.19, 126.27, 102.72, 93.44, 31.80, 29.40, 22.54, 19.24, 14.11, -0.90. **ESI-MS**: calculated [$C_{17}H_{26}Si$ + Na]$^+$: 281.1696, found: 281.1691.

(2,5-diphenylpenta-2,3-dien-1-yl)trimethylsilane (2e)

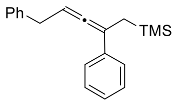

^{1}H NMR (400 MHz, CDCl$_3$) δ 7.46 – 7.40 (m, 2H), 7.37 – 7.28 (m, 6H), 7.26 – 7.18 (m, 2H), 5.67 – 5.54 (m, 1H), 3.49 (d, *J* = 7.3 Hz, 2H), 1.94 – 1.79 (m, 2H), 0.04 (s, 9H). **^{13}C NMR (100 MHz, CDCl$_3$)** δ 204.87, 140.71, 138.29, 128.65, 128.57, 128.25, 126.55, 126.42, 126.28, 103.55, 92.58, 36.40, 19.38, -0.89. **ESI-MS**: calculated [$C_{20}H_{24}Si$ + H]$^+$: 293.1720, found: 293.1726.

Trimethyl(2-phenylbuta-2,3-dien-1-yl)silane (2f)

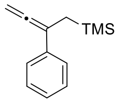

^{1}H NMR (600 MHz, CDCl$_3$) δ 7.44 – 7.40 (m, 2H), 7.34 – 7.29 (m, 2H), 7.22 – 7.18 (m, 1H), 5.03 (t, *J* = 2.6 Hz, 2H), 1.82 (t, *J* = 2.6 Hz, 2H), 0.03 (s, 9H). **^{13}C NMR (150 MHz, CDCl$_3$)** δ 209.39, 137.56, 128.30, 126.58, 126.34, 102.52, 77.38, 18.69, -0.88. **ESI-MS**: calculated [$C_{13}H_{18}Si$ + H]$^+$: 203.1251, found: 203.1262.

(2-(4-fluorophenyl)penta-2,3-dien-1-yl)trimethylsilane (2g)

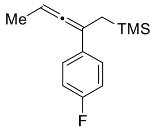

^{1}H NMR (400 MHz, CDCl$_3$) δ 7.40 – 7.32 (m, 2H), 7.04 – 6.92 (m, 2H), 5.41 (q, *J* = 6.8 Hz, 1H), 1.83 – 1.70 (m, 5H), 0.02 (s, 9H). **^{13}C NMR (100 MHz, CDCl$_3$)** δ 205.10 (d, *J* = 1.8 Hz), 161.71 (d, *J* = 245.3 Hz), 134.72 (d, *J* = 3.1 Hz), 127.81 (d, *J* = 7.9 Hz),

115.01 (d, J = 21.4 Hz), 101.58, 88.05, 19.39, 14.81, -1.03. **^{19}F NMR (471 MHz, $CDCl_3$)** δ -106.43. **ESI-MS**: calculated $[C_{14}H_{19}FSi + H]^+$: 235.1313, found:235.1321.

Trimethyl(2-(p-tolyl)penta-2,3-dien-1-yl)silane (2h)

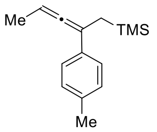

^{1}H NMR (400 MHz, $CDCl_3$) δ 7.34 – 7.27 (m, 2H), 7.15 – 7.09 (m, 2H), 5.46 – 5.35 (m, 1H), 2.35 (s, 3H), 1.81 – 1.78 (m, 2H), 1.77 (d, J = 7.0 Hz, 3H), 0.04 (s, 9H). **^{13}C NMR (100 MHz, $CDCl_3$)** δ 205.09, 135.99, 135.80, 128.95, 126.24, 102.19, 87.68, 21.17, 19.16, 14.87, -0.98. **ESI-MS**: calculated $[C_{15}H_{22}Si + H]^+$: 231.1564, found: 231.1559.

(2-(3-chlorophenyl)penta-2,3-dien-1-yl)trimethylsilane (2i)

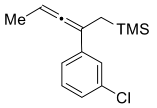

^{1}H NMR (400 MHz, $CDCl_3$) δ 7.40 – 7.35 (m, 1H), 7.29 – 7.25 (m, 1H), 7.24 – 7.19 (m, 1H), 7.17 – 7.11 (m, 1H), 5.53 – 5.36 (m, 1H), 1.82 – 1.70 (m, 5H), 0.03 (s, 9H). **^{13}C NMR (100 MHz, $CDCl_3$)** δ 205.46, 140.95, 134.25, 129.36, 126.45, 126.33, 124.39, 101.59, 88.51, 19.01, 14.68, -1.03. **ESI-MS**: calculated $[C_{14}H_{19}ClSi + H]^+$: 251.1017, found: 251.1021.

Trimethyl(2-(m-tolyl)penta-2,3-dien-1-yl)silane (2j)

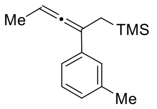

^{1}H NMR (400 MHz, $CDCl_3$) δ 7.24 – 7.16 (m, 3H), 7.02 – 6.96 (m, 1H), 5.48 – 5.34 (m, 1H), 2.35 (s, 3H), 1.81 – 1.78 (m, 2H), 1.76 (d, J = 7.0 Hz, 3H), 0.03 (s, 9H). **^{13}C NMR (100 MHz, $CDCl_3$)** δ 205.33, 138.73, 137.65, 128.11, 127.19, 127.11, 123.46, 102.36, 87.69, 21.67, 19.21, 14.84, -0.97. **ESI-MS**: calculated $[C_{15}H_{22}Si + H]^+$: 231.1564, found: 231.1571.

2.2.2 Preparation of allenylmethylsilane 2k[4,5]

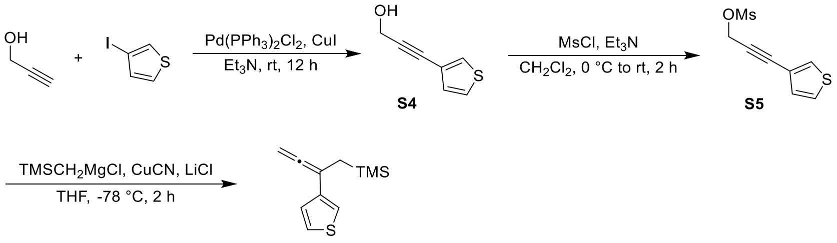

To a solution of aryl iodide (5 mmol, 1.0 equiv.) and propargyl alcohol (15 mmol, 3.0 equiv.) in triethylamine (25 mL, 0.2 M) was added $Pd(PPh_3)_2Cl_2$ (0.05 mmol, 1 mol%) and CuI (0.25 mmol, 5 mol%) under N_2 atmosphere. The resulting mixture was stirred at ambient temperature for 12 h. After completion of the reaction as monitored by TLC, the reaction mixture was diluted with EtOAc (20 mL) and filtered through a short pad of Celite. The filtrate was concentrated in vacuo and the crude residue was purified by flash column chromatography (petroleum ether/EtOAc) on silica gel to afford the propargylic alcohol **S4**.

To a solution of the individual propargylic alcohol **S4** (1.0 equiv.) and triethylamine (2.0 equiv.) in CH_2Cl_2 (0.2 M) was added methanesulfonyl chloride (1.5 equiv.) dropwise at 0 °C under N_2 atmosphere. The reaction mixture was allowed to warm to room temperature and stirred for 2 h. After completion, saturated aqueous $NaHCO_3$ (15 mL) was added and the aqueous layer was extracted with CH_2Cl_2 (15 mL × 3). The combined organic extracts were washed with H_2O (20 mL) and brine (10 mL), dried over Na_2SO_4, and concentrated in vacuo. The crude product was purified by flash column chromatography

(petroleum ether/EtOAc) to afford **S5**.

To an ice-cooled suspension of CuCN (3.0 equiv.) and LiCl (6.0 equiv.) in anhydrous THF (0.1 M) was added a solution of $TMSCH_2MgCl$ (1.3 M in THF, 2.5 equiv.). After being stirred for 25 min, the mixture was cooled to -78 °C, and a solution of the **S5** (1.0 equiv.) in THF (5 mL) was added. After 2 h, the reaction was quenched with saturated aqueous NH_4Cl (5 mL). The reaction mixture was extracted with Et_2O (20 mL × 3). The combined organic layers were washed with brine (10 mL), dried over Na_2SO_4, and concentrated in vacuo. The crude products were purified by flash column chromatography (petroleum ether/EtOAc) to afford allenylmethylsilane **2k**.

Trimethyl(2-(thiophen-3-yl)buta-2,3-dien-1-yl)silane (2k)

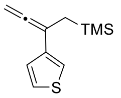

^{1}H NMR (400 MHz, CDCl$_3$) δ 7.23 (dd, *J* = 5.0, 2.9 Hz, 1H), 7.12 (dd, *J* = 5.0, 1.1 Hz, 1H), 7.01 (d, *J* = 1.8 Hz, 1H), 5.00 (t, *J* = 2.1 Hz, 2H), 1.74 (t, *J* = 2.5 Hz, 2H), 0.04 (s, 9H). **^{13}C NMR (100 MHz, CDCl$_3$)** δ 209.74, 139.53, 127.09, 125.28, 119.35, 98.78, 77.25, 19.51, -0.92. **ESI-MS**: calculated $[C_{11}H_{16}SSi + Na]^+$: 231.0634, found: 231.0617.

2.2.3 Preparation of allenylmethylsilanes 2l[4,5]

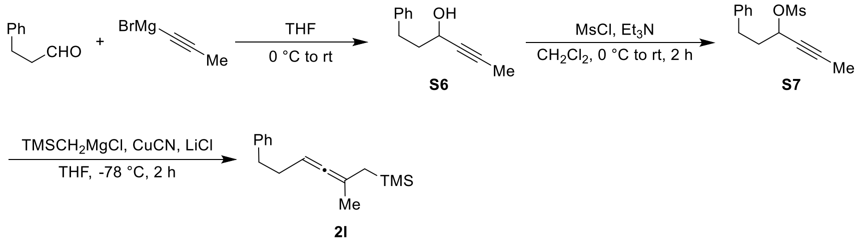

A round-bottom flask with a stir bar was dried in the oven and cooled under N_2 atmosphere. Ethynylmagnesium bromide in THF (0.5 M in THF, 1.2 equiv.) was transferred to the flask via a syringe. Then the reaction was cooled to 0 °C. Aldehyde (10.0 mmol, 1.0 equiv.) was dissolved in THF (0.5 M) and added dropwise via syringe to the above solution at 0 °C. After 30 min, the reaction mixture was allowed to warm to room temperature and stirred for 2 h. The mixture was stirred for a further 2 hours. The reaction was quenched with saturated aqueous NH_4Cl (5 mL). The reaction mixture was extracted with Et_2O (20 mL × 3). The combined organic layers were washed with brine (10 mL), dried over Na_2SO_4, and concentrated in vacuo. The crude products were purified by flash column chromatography (petroleum ether/EtOAc) to afford the corresponding propargylic alcohol **S6**.

To a solution of the individual propargylic alcohol **S6** (1.0 equiv.) and triethylamine (2.0 equiv.) in CH_2Cl_2 (0.2 M) was added methanesulfonyl chloride (1.5 equiv.) dropwise at 0 °Cunder N_2 atmosphere. The reaction mixture was allowed to warm to room temperature and stirred for 2 h. After completion, saturated aqueous $NaHCO_3$ (15 mL) was added and the aqueous layer was extracted with CH_2Cl_2 (15 mL × 3). The combined organic extracts were washed with H_2O (20 mL) and brine (10 mL), dried over Na_2SO_4, and concentrated in vacuo. The crude product was purified by flash column chromatography (petroleum ether/EtOAc) to afford **S7**.

To an ice-cooled suspension of CuCN (3.0 equiv.) and LiCl (6.0 equiv.) in anhydrous THF (0.1 M) was added a solution of $TMSCH_2MgCl$ (1.3 M in THF, 2.5 equiv.). After being stirred for 25 min, the mixture was cooled to -78 °C, and a solution of the **S7** (1.0 equiv.) in THF (5 mL) was added. After 2 h, the reaction was quenched with saturated aqueous NH_4Cl (5 mL). The reaction mixture was extracted with Et_2O (20 mL × 3). The combined organic layers were washed with brine (10 mL), dried over Na_2SO_4, and concentrated in vacuo. The crude products were purified by flash column chromatography

(petroleum ether/EtOAc) to afford allenylmethylsilane **2l**.

Trimethyl(2-methyl-6-phenylhexa-2,3-dien-1-yl)silane (2l)

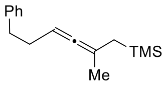

^{1}H NMR (400 MHz, $CDCl_3$) δ 7.32 – 7.24 (m, 2H), 7.22 – 7.14 (m, 3H), 5.03 – 4.94 (m, 1H), 2.71 (t, J = 7.8 Hz, 2H), 2.31 – 2.22 (m, 2H), 1.63 (d, J = 2.9 Hz, 3H), 1.30 – 1.28 (m, 2H), 0.04 (s, 9H). **^{13}C NMR (100 MHz, $CDCl_3$)** δ 202.02, 142.34, 128.68, 128.35, 125.82, 97.27, 88.56, 35.90, 31.79, 23.66, 22.14, -1.03. **ESI-MS**: calculated $[C_{16}H_{24}Si + H]^+$: 245.1720, found: 245.1727.

2.2.4 Preparation of allenylmethyl phenyl sulfone 2m[6]

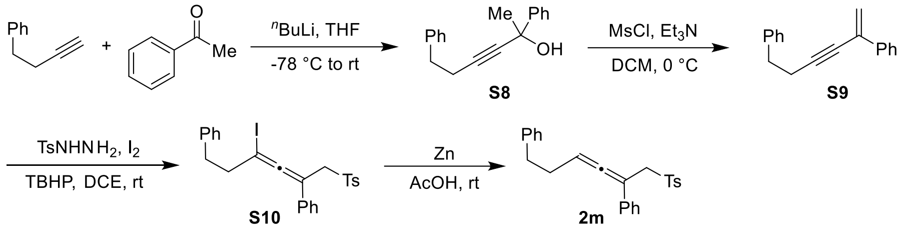

To a 50 mL round bottom flask equipped with a stir bar was added but-3-yn-1-ylbenzene (10.0 mmol, 1.0 equiv.) and THF (10 mL) and then cooled to -78 ℃. ^{n}BuLi (2.5 M in hexane, 1.0 equiv.) was added dropwise and the resulting solution was stirred for 30 min room temperature and cooled to −78 °C. The acetophenone (5.0 mmol, 1.0 equiv.) was added dropwise. The reaction mixture was allowed to warm to room temperature and stirred for 7 h. After completion, The reaction was quenched with saturated aqueous NH_4Cl (5 mL) and then extracted with EtOAc (20 mL × 3). The combined organic layers were washed with brine (10 mL), dried over Na_2SO_4. The reaction mixture was concentrated to afford the crude product **S8** without further purification.

To a solution of crude **S8** (1.0 equiv.) and triethylamine (5.0 equiv.) in CH_2Cl_2 (0.2 M) was added methanesulfonyl chloride (2.5 equiv.) dropwise at 0 ℃. The reaction mixture stirred for 30 min at 0 ℃. After completion, saturated aqueous NH_4Cl (15 mL) was added and the aqueous layer was extracted with CH_2Cl_2 (15 mL × 3). The combined organic extracts were washed with H_2O (20 mL) and brine (10 mL), dried over Na_2SO_4, and concentrated in vacuo. The crude product was purified by flash column chromatography (petroleum ether/EtOAc) to afford **S9**.

To a solution of 4-methylbenzenesulfonohydrazide (745 mg, 4.0 mmol) in 1,2-dichloroethane (DCE, 1.0 mL), **S9** (0.93 g, 4.0 mmol), I_2 (1.01, 4.0 mmol), and TBHP (17% in water, 0.7 mL, 4.8 mmol) were added in a Schlenck tube under air atmosphere.The reaction mixture was stirred at room temperature for 1 h. After the reaction was completed, the reaction mixture was purified using column chromatography to yield the corresponding product **S10** (1.17 g, 57% yield).

To a Schlenk tube were added **S10** (1.17 g, 2.28 mmol), Zn powder (1.8 g, 27.36 mmol), and AcOH (1.5 mL) under N_2 atmosphere. The resulting suspension was stirred for 4.5 h at room temperature and monitored using TLC. After filtration, Et_2O (10 mL) was added.The resulting solution was washed with water and brine, dried over Na_2SO_4, filtered, and concentrated under reduced pressure. The reaction mixture was purified using column chromatography to yield the corresponding product **2m** (193 mg, 21% yield).

(6-tosylhexa-3,4-diene-1,5-diyl)dibenzene (2m)

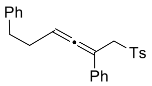

^{1}H NMR (400 MHz, $CDCl_3$) δ 7.70 – 7.62 (m, 2H), 7.30 – 7.26 (m, 2H), 7.25 – 7.18 (m, 8H), 7.13 – 7.09 (m, 2H), 5.24 (t, J = 6.9 Hz, 1H), 4.12 (dd, J = 14.4, 0.7 Hz, 1H),

3.96 (dd, J = 14.4, 1.7 Hz, 1H), 2.65 (t, J = 7.3 Hz, 2H), 2.34 (s, 3H), 2.26 – 2.18 (m, 2H). **^{13}C NMR (100 MHz, CDCl$_3$)** δ 208.51, 144.66, 141.10, 135.42, 134.36, 129.60, 128.90, 128.58, 128.51, 128.46, 127.10, 126.25, 126.18, 96.63, 94.23, 58.29, 35.11, 29.74, 21.65. **ESI-MS**: calculated [$C_{25}H_{24}O_2S$ + Na]$^+$: 411.1389, found: 411.1394.

2.3 Preparation of allylmethylsilanes

2.3.1 Preparation of allenylmethylsilane 4[7-9]

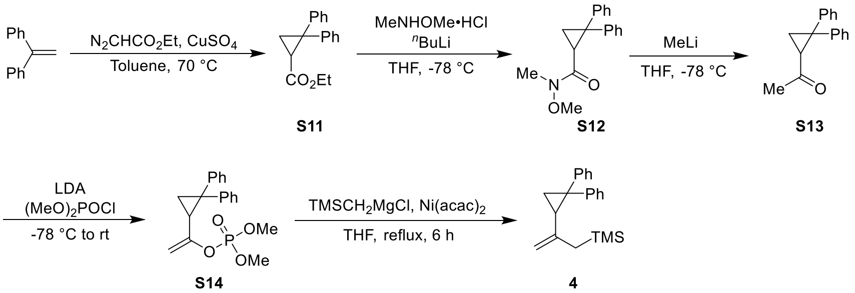

A mixture of 1,1-diphenylethylene (9.0 g, 50 mmol, 1.0 equiv.) and anhydrous $CuSO_4$ (0.54 g, 5 mmol, 0.1 equiv.) in 40 mL of toluene was heated at 70 °C, and a solution of ethyl diazoacetate (10.5 mL, 100 mmol, 2.0 equiv.) in 80 mL of toluene was added dropwise over 3 h. The mixture was heated at reflux for an additional 3 h and allowed to cool to room temperature. Stirring was continued overnight. The reaction mixture was treated with 40 mL of water and the resulting phases were separated. The aqueous phase was extracted with Et_2O (20 mL × 3). The combined organic layers were washed with brine, dried over Na_2SO_4, and concentrated in vacuo. The crude products were purified by flash column chromatography (petroleum ether/EtOAc) to afford **S11** (12.35 g, 46.4 mmol, 93% yield).

To a solution of *N,O*-dimethylhydroxylamine hydrochloride (2.92 g, 30.0 mmol, 3.0 equiv.) in THF (60 mL) was added ^{n}BuLi (2.5 M in THF, 60 mmol, 6.0 equiv.) at -78 °C under N_2 atmosphere. After stirring at room temperature for 10 min, the mixture was cooled to -78 °C, and a solution of **S11** (22.4 mmol) in THF (20 mL) was added. The reaction mixture was stirred at -78 °C for 2 h, and the reaction was quenched with saturated aqueous NH_4Cl and warmed to room temperature. The aqueous phase was extracted with Et_2O (20 mL × 3). The combined organic layers were washed with brine, dried over Na_2SO_4, and concentrated in vacuo. The crude products were purified by flash column chromatography (petroleum ether/EtOAc) to afford pure Weinreb amide **S12** (2.17 g, 7.7 mmol, 77% yield).

To a solution of the Weinreb amide **S12** (2.17 g, 7.7 mmol, 1.0 equiv.) in THF (20 mL) at -78 °C was added dropwise a solution of the methyllithium (1.3 M in THF, 23.0 mmol, 3.0 equiv.). After stirring at -78 °C for 0.5-2 h, the reaction mixture was quenched with methanol and saturated aqueous NH_4Cl, then warmed to room temperature and extracted with Et_2O (20 mL × 3). The combined organic layers were dried over Na_2SO_4 and concentrated in vacuo. The crude products were purified by flash column chromatography (petroleum ether/EtOAc) to afford **S13** (1.54 g, 6.5 mmol, 85% yield).

To a solution of the **S13** (1.54 g, 6.5 mmol, 1.0 equiv.) in THF (10 mL) at -78 °C was added dropwise a solution of the lithium diisopropylamide (2.0 M in THF, 7.5 mmol, 1.15 equiv.). The mixture was stirred at -78 °C for 30 min, dimethyl chlorophosphate (1.13 g, 7.8 mmol, 1.2 equiv.) was added, and the mixture was allowed to warm to room temperature for 1 h. After completion, the reaction mixture was diluted with Et_2O (20 mL), and the mixture was washed with water (20 mL), 1 N aq. HCl (20 mL), and sat. aq. $NaHCO_3$(20 mL). The combined organic layers were dried over Na_2SO_4 and concentrated in vacuo. The crude products were purified by flash column chromatography (petroleum ether/EtOAc) to

afford **S14** (1.47 g, 4.3 mmol, 66% yield).

To a suspension of $Ni(acac)_2$ (180 mg, 0.7 mmol, 0.1 equiv.) in anhydrous THF (20 mL) was added a solution of $TMSCH_2MgCl$ (1.3 M in THF, 14.0 mmol, 2.0 equiv.) dropwise at room temperature under N_2 atmosphere. After being stirred for 2 min, **S14** (2.4 g, 7.0 mmol, 1.0 equiv.) was added at room temperature. The mixture was stirred at room temperature for 15 h, then cooled to 0 °C with an ice bath, After completion, the reaction was quenched with 1 N aq. HCl (5 mL) at 0 °C. The reaction mixture was extracted with Et_2O (20 mL × 3). The combined organic layers were washed with brine (10 mL), dried over Na_2SO_4, and concentrated in vacuo. The crude products were purified by flash column chromatography (petroleum ether/EtOAc) to afford allenylmethylsilane **4** (1.1 g, 3.6 mmol, 51% yield).

(2-(2,2-diphenylcyclopropyl)allyl)trimethylsilane (4)

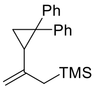

^{1}H NMR (600 MHz, $CDCl_3$) δ 7.31 – 7.26 (m, 5H), 7.26 – 7.21 (m, 3H), 7.20 – 7.14 (m, 2H), 4.46 (d, *J* = 0.8 Hz, 1H), 4.38 (d, *J* = 0.8 Hz, 1H), 2.23 (dd, *J* = 8.2, 7.0 Hz, 1H), 1.78 (dd, *J* = 6.5, 5.4 Hz, 1H), 1.52 – 1.43 (m, 2H), 1.40 (dd, *J* = 8.6, 5.2 Hz, 1H), 0.07 (s, 9H). **^{13}C NMR (150 MHz, $CDCl_3$)** δ 147.31, 143.44, 141.04, 130.41, 128.40, 128.05, 127.87, 126.24, 125.95, 108.49, 38.01, 34.33, 27.91, 19.32, -1.12. **ESI-MS**: calculated $[C_{21}H_{26}Si + H]^+$: 307.1877, found: 307.1879.

2.3.2 Preparation of allenylmethylsilanes 7[10.11]

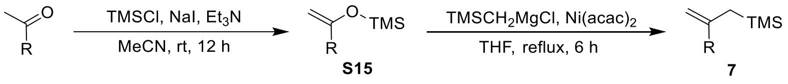

To a solution of ketone (10.0 mmol, 1.0 equiv.) in MeCN (1.5 M) was added sodium iodide (12.0 mmol, 1.2 equiv.) at room temperature under N_2 atmosphere. After being stirred for 5 min, triethylamine (15.0 mmol, 1.5 equiv.) was added, followed by chlorotrimethylsilane (12.0 mmol, 1.2 equiv.). The reaction mixture was stirred overnight at room temperature. After completion, the reaction was quenched with saturated aqueous NH_4Cl (50 mL) at 0 °C. The reaction mixture was extracted with petroleum ether (20 mL × 3). The combined organic layers were washed with brine (30 mL), dried over Na_2SO_4, and concentrated in vacuo. The residue was distilled under reduced pressure to provide pure silyl enol ethers **S15**.

To a suspension of $Ni(acac)_2$ (0.05 equiv.) in anhydrous THF (1.0 M) was added a solution of $TMSCH_2MgCl$ (1.3 M in THF, 2.0 equiv.) dropwise at room temperature under N_2 atmosphere. After being stirred for 2 min, silyl enol ethers **S15** (1.0 equiv.) was added at room temperature. The resulting black mixture was heated to reflux for 6 h. After completion, the reaction was quenched with saturated aqueous NH_4Cl (5 mL) at 0 °C. the reaction mixture was diluted with Et_2O (20 mL) and filtered through a short pad of Celite. The reaction mixture was extracted with Et_2O (20 mL × 3). The combined organic layers were washed with brine (10 mL), dried over Na_2SO_4, and concentrated in vacuo. The crude products were purified by flash column chromatography (petroleum ether/EtOAc) to afford allenylmethylsilane **7**.

Trimethyl(2-phenylallyl)silane (7a)

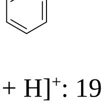

^{1}H NMR (600 MHz, $CDCl_3$) δ 7.40 (d, *J* = 7.5 Hz, 2H), 7.30 (t, *J* = 7.4 Hz, 2H), 7.27 – 7.21 (m, 1H), 5.13 (s, 1H), 4.87 (s, 1H), 2.03 (s, 2H), -0.10 (s, 9H). **^{13}C NMR (150 MHz, $CDCl_3$)** δ 146.78, 142.92, 128.21, 127.33, 126.47, 110.18, 26.28, -1.28. **ESI-MS**: calculated $[C_{12}H_{18}Si + H]^+$: 191.1251, found: 191.1241.

(2-(4-fluorophenyl)allyl)trimethylsilane (7b)

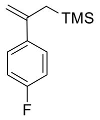

^{1}H NMR (400 MHz, $CDCl_3$) δ 7.41 – 7.32 (m, 2H), 7.03 – 6.95 (m, 2H), 5.07 (d, *J* = 1.5 Hz, 1H), 4.85 (d, *J* = 1.0 Hz, 1H), 2.04 – 1.96 (m, 2H), -0.10 (s, 9H). **^{13}C NMR (100 MHz, $CDCl_3$)** δ 162.29 (d, *J* = 245.8 Hz), 145.75, 138.97 (d, *J* = 3.3 Hz), 128.00 (d, *J* = 7.9 Hz), 115.00 (d, *J* = 21.2 Hz), 110.16 (d, *J* = 1.1 Hz), 26.47, -1.30. **^{19}F NMR (376 MHz, $CDCl_3$)** δ -115.67. **ESI-MS**: calculated $[C_{12}H_{17}FSi + H]^+$: 209.1156, found: 209.1156.

(2-(4-chlorophenyl)allyl)trimethylsilane (7c)

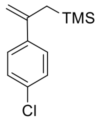

^{1}H NMR (400 MHz, $CDCl_3$) δ 7.36 – 7.31 (m, 2H), 7.29 – 7.23 (m, 2H), 5.11 (d, *J* = 1.5 Hz, 1H), 4.87 (d, *J* = 1.0 Hz, 1H), 1.99 (d, *J* = 0.7 Hz, 2H), -0.10 (s, 9H). **^{13}C NMR (100 MHz, $CDCl_3$)** δ 145.62, 141.33, 133.06, 128.36, 127.74, 110.74, 26.19, -1.28. **ESI-MS**: calculated $[C_{12}H_{17}ClSi + H]^+$: 225.0861, found: 225.0865.

Trimethyl(2-(p-tolyl)allyl)silane (7d)

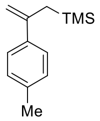

^{1}H NMR (400 MHz, $CDCl_3$) δ 7.36 – 7.29 (m, 2H), 7.16 – 7.07 (m, 2H), 5.13 (d, *J* = 1.7 Hz, 1H), 4.85 (d, *J* = 0.7 Hz, 1H), 2.36 (s, 3H), 2.03 (s, 2H), -0.07 (s, 9H). **^{13}C NMR (100 MHz, $CDCl_3$)** δ 146.49, 139.93, 137.00, 128.89, 126.30, 109.42, 26.16, 21.24, -1.23. **ESI-MS**: calculated $[C_{13}H_{20}Si + H]^+$: 205.1407, found: 205.1412.

(2-(4-methoxyphenyl)allyl)trimethylsilane (7e)

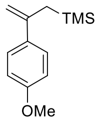

^{1}H NMR (600 MHz, $CDCl_3$) δ 7.36 – 7.32 (m, 2H), 6.85 – 6.81 (m, 2H), 5.06 (d, *J* = 1.7 Hz, 1H), 4.78 (d, *J* = 1.4 Hz, 1H), 3.81 (s, 3H), 1.98 (s, 2H), -0.10 (s, 9H). **^{13}C NMR (150 MHz, $CDCl_3$)** δ 159.00, 146.00, 135.31, 127.51, 113.48, 108.67, 55.37, 26.25, -1.24. **ESI-MS**: calculated $[C_{13}H_{20}OSi + Na]^+$:243.1176, found: 243.1186.

(2-([1,1'-biphenyl]-4-yl)allyl)trimethylsilane (7f)

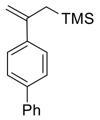

^{1}H NMR (400 MHz, $CDCl_3$) δ 7.65 – 7.59 (m, 2H), 7.58 – 7.53 (m, 2H), 7.51 – 7.47 (m, 2H), 7.47 – 7.40 (m, 2H), 7.38 – 7.31 (m, 1H), 5.21 (d, *J* = 1.5 Hz, 1H), 4.90 (d, *J* = 1.2 Hz, 1H), 2.06 (s, 2H), -0.06 (s, 9H). **^{13}C NMR (100 MHz, $CDCl_3$)** δ 146.17, 141.74, 140.88, 140.02, 128.89, 127.34, 127.07, 126.87, 126.82, 110.22, 26.10, -1.21. **ESI-MS**: calculated $[C_{18}H_{22}Si + H]^+$: 267.1564, found:267.1561.

(2-(3-chlorophenyl)allyl)trimethylsilane (7g)

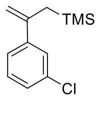

^{1}H NMR (600 MHz, $CDCl_3$) δ 7.40 – 7.38 (m, 1H), 7.29 – 7.27 (m, 1H), 7.24 – 7.22 (m, 2H), 5.14 (d, *J* = 1.3 Hz, 1H), 4.92 – 4.89 (m, 1H), 1.99 (d, *J* = 1.0 Hz, 2H), -0.08 (s, 9H). **^{13}C NMR (150 MHz, $CDCl_3$)** δ 145.55, 144.82, 134.15, 129.47, 127.34, 126.62, 124.62, 111.32, 26.15, -1.29. **ESI-MS**: calculated $[C_{12}H_{17}ClSi + H]^+$: 225.0861, found: 225.0870.

Trimethyl(2-(m-tolyl)allyl)silane (7h)

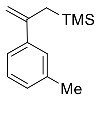

^{1}H NMR (600 MHz, $CDCl_3$) δ 7.24 – 7.22 (m, 1H), 7.22 – 7.18 (m, 2H), 7.09 – 7.06 (m, 1H), 5.13 (d, *J* = 1.8 Hz, 1H), 4.88 – 4.85 (m, 1H), 2.37 (s, 3H), 2.03 (d, *J* = 1.0 Hz, 2H), -0.08 (s, 9H). **^{13}C NMR (150 MHz, $CDCl_3$)** δ 146.83, 142.88, 137.62, 128.06, 127.20, 123.59, 109.98, 26.28, 21.65, -1.25. **ESI-MS**: calculated $[C_{13}H_{20}Si + Na]^+$: 227.1226, found: 227.1242.

(2-(3-methoxyphenyl)allyl)trimethylsilane (7i)

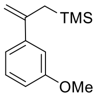

^{1}H NMR (600 MHz, CDCl$_3$) δ 7.25 – 7.21 (m, 1H), 7.03 – 6.99 (m, 1H), 6.97 – 6.94 (m, 1H), 6.82 – 6.79 (m, 1H), 5.15 (d, *J* = 1.7 Hz, 1H), 4.87 (d, *J* = 1.2 Hz, 1H), 3.83 (s, 3H), 2.02 (d, *J* = 0.8 Hz, 2H), -0.07 (s, 9H). **^{13}C NMR (150 MHz, CDCl$_3$)** δ 159.48, 146.63, 144.49, 129.13, 119.10, 112.48, 112.42, 110.34, 55.33, 26.30, -1.27. **ESI-MS**: calculated [$C_{13}H_{20}OSi$ + H]$^+$: 221.1356, found: 221.1362.

(2-(2-methoxyphenyl)allyl)trimethylsilane (7j)

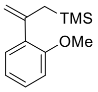

^{1}H NMR (600 MHz, CDCl$_3$) δ 7.26 – 7.21 (m, 1H), 7.18 – 7.15 (m, 1H), 6.92 – 6.88 (m, 1H), 6.87 – 6.84 (m, 1H), 4.97 – 4.94 (m, 1H), 4.89 (d, *J* = 2.2 Hz, 1H), 3.84 (s, 3H), 2.09 (d, *J* = 1.0 Hz, 2H), -0.14 (s, 9H). **^{13}C NMR (150 MHz, CDCl$_3$)** δ 156.62, 146.95, 133.55, 130.13, 128.39, 120.54, 112.57, 110.74, 55.47, 27.39, -1.36. **ESI-MS**: calculated [$C_{13}H_{20}OSi$ + H]$^+$: 221.1356, found: 221.1358.

(2-(furan-2-yl)allyl)trimethylsilane (7k)

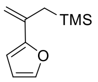

^{1}H NMR (600 MHz, CDCl$_3$) δ 7.34 (d, *J* = 1.6 Hz, 1H), 6.36 (dd, *J* = 3.3, 1.8 Hz, 1H), 6.25 (d, *J* = 3.3 Hz, 1H), 5.39 (d, *J* = 1.5 Hz, 1H), 4.74 (d, *J* = 0.8 Hz, 1H), 1.84 (d, *J* = 0.9 Hz, 2H), -0.00 (s, 9H). **^{13}C NMR (150 MHz, CDCl$_3$)** δ 155.61, 141.75, 135.16, 111.16, 107.23, 106.46, 23.53, -1.36. **ESI-MS**: calculated [$C_{10}H_{16}OSi$ + Na]$^+$: 203.0863, found: 203.0875.

Trimethyl(2-(naphthalen-2-yl)allyl)silane (7l)

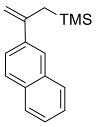

^{1}H NMR (400 MHz, CDCl$_3$) δ 7.85 – 7.78 (m, 4H), 7.66 – 7.61 (m, 1H), 7.50 – 7.45 (m, 2H), 5.32 (d, *J* = 1.3 Hz, 1H), 5.03 – 4.98 (m, 1H), 2.17 (s, 2H), -0.05 (s, 9H). **^{13}C NMR (100 MHz, CDCl$_3$)** δ 146.45, 140.01, 133.43, 132.88, 128.29, 127.75, 127.64, 126.15, 125.81, 125.04, 110.86, 26.17, -1.18. **ESI-MS**: calculated [$C_{16}H_{20}Si$ + H]$^+$: 241.1407, found: 241.1390.

(*E*)-trimethyl(2-methylene-4-phenylbut-3-en-1-yl)silane (7m)

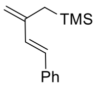

^{1}H NMR (600 MHz, CDCl$_3$) δ 7.47 – 7.40 (m, 2H), 7.37 – 7.31 (m, 2H), 7.26 – 7.21 (m, 1H), 6.83 (d, *J* = 16.1 Hz, 1H), 6.49 (d, *J* = 16.1 Hz, 1H), 5.06 (d, *J* = 1.6 Hz, 1H), 4.89 (d, *J* = 1.6 Hz, 1H), 1.86 (d, *J* = 0.5 Hz, 2H), 0.07 (s, 9H). **^{13}C NMR (150 MHz, CDCl$_3$)** δ 144.08, 137.59, 132.18, 128.88, 128.74, 127.45, 126.55, 114.99, 22.30, -1.00. **ESI-MS**: calculated [$C_{14}H_{20}Si$ + H]$^+$: 217.1407, found: 217.1421.

3. Photographic guide for electrochemical reactions

3.1 Overview of materials used

From left to right: 1) Electrochemical cell; 2) Carbon rod (d = 6 mm) anode and platinum plate (1.0 cm × 1.0 cm × 0.2 mm) cathode.

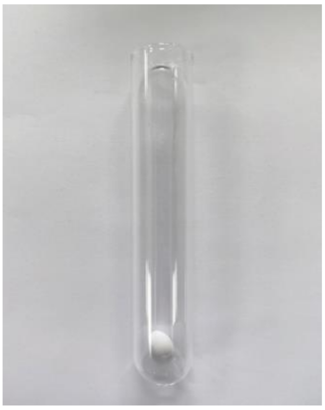

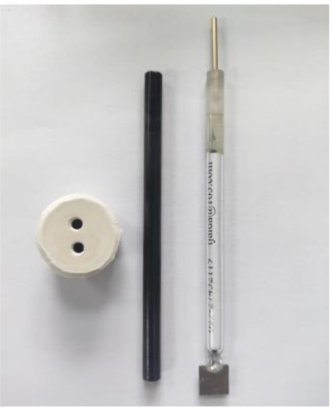

3.2 Assembling the cell

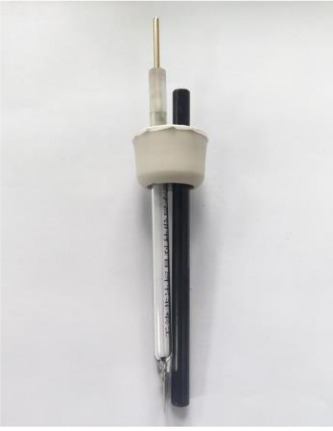

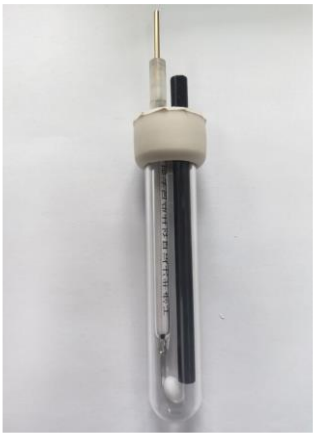

3.3 Electrolysis

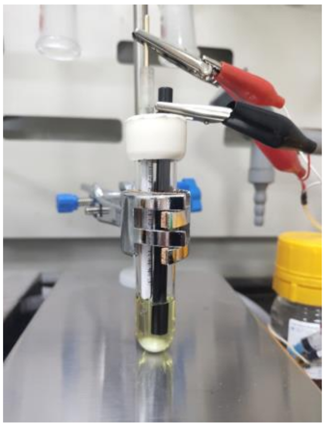

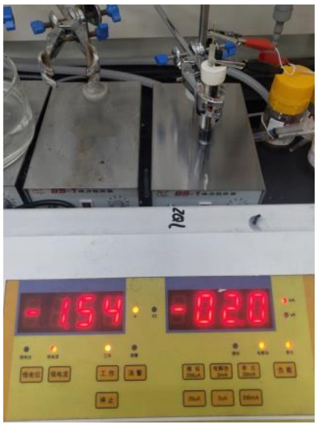

4. Optimization studies

***Table S1*: Survey of chiral ligands**[a]

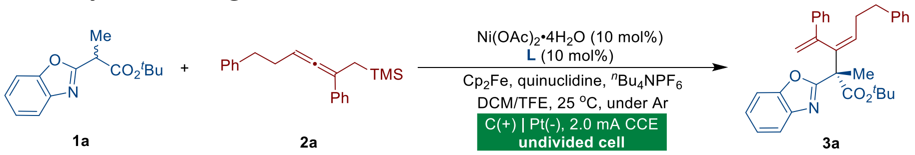

Entry	L	Yield (%)	e.e. (%) of **3a**
1	(*R*,*R*)-**L1**	13	*racemic*
2	(*R*)-**L2**	35	18
3	(*S*,*S*,*R*,*R*)-**L3**	38	52
4	(*S*,*S*,*R*,*R*)-**L4**	48	91
5	(*S*,*S*,*R*,*R*)-**L5**	44	71
6	(*R*,*R*)-**L6**	79	95
7	(*R*,*R*)-**L7**	55	90
8	(*R*,*R*)-**L8**	75	95
9	(*R*,*R*)-**L9**	79	96
10	(*R*,*R*)-**L10**	73	92

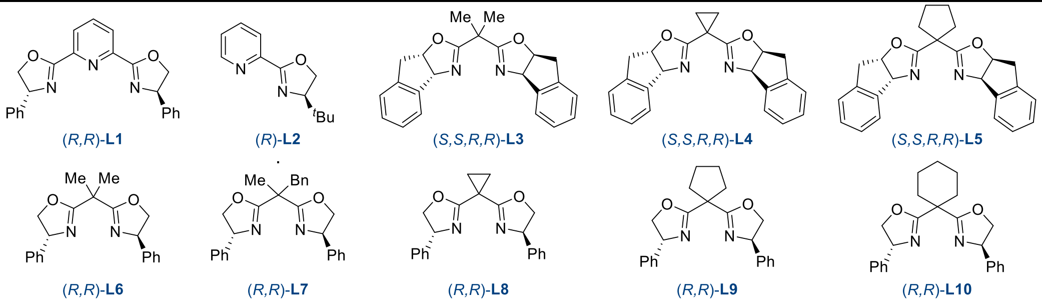

[a]Reactions were carried out with **1a** (0.1 mmol), **2a** (0.3 mmol), ^{n}Bu$_4$NPF$_6$ (0.15 mmol), quinuclidine (0.05 mmol), Ni(OAc)$_2$·4H$_2$O (10 mol%), **L** (10 mol%), Cp$_2$Fe (10 mol%) and DCM/TFE = 2:1 (3 mL) at 25 °C for 3 h in an undivided cell. TFE, 2,2,2-trifluoroethanol.

***Table S2*: Survey of substituent of benzoxazolyl acetates** [a]

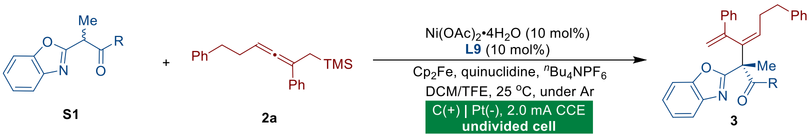

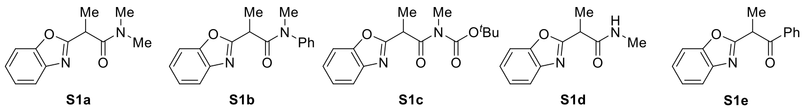

Entry	Substrates **S1**	**3**	Yield (%)	e.e. (%) of **3**
1	**S1a**	—	nr	—
2	**S1b**	—	nr	—
3	**S1c**	—	nr	—
4	**S1d**	**3aa**	36	92
5[b]	**S1d**	**3aa**	42	92
6[c]	**S1d**	**3aa**	37	92
7[d]	**S1d**	**3aa**	nr	—
8[e]	**S1d**	**3aa**	41	94
9[e]	**S1e**	**3z**	59	78

[a]Reactions were carried out with **S1** (0.1 mmol), **2a** (0.3 mmol), ^{n}Bu$_4$NPF$_6$ (0.15 mmol), quinuclidine (0.05 mmol), Ni(OAc)$_2$·4H$_2$O (10 mol%), **L9** (10 mol%), Cp$_2$Fe (10 mol%) and DCM/TFE = 2:1 (3 mL) at 25 °C in an undivided cell. [b]**2a** (0.6 mmol). [c]**2a** (0.6 mmol), 50 °C. [d]**2a** (0.6 mmol), HCOOH (20 mol%). [e]Ni(OAc)$_2$·4H$_2$O (20 mol%), **L9** (20 mol%), **2a** (0.6 mmol), 50 °C. TFE, 2,2,2-trifluoroethanol. nr = no reaction.

Table S3: Survey of esters[a]

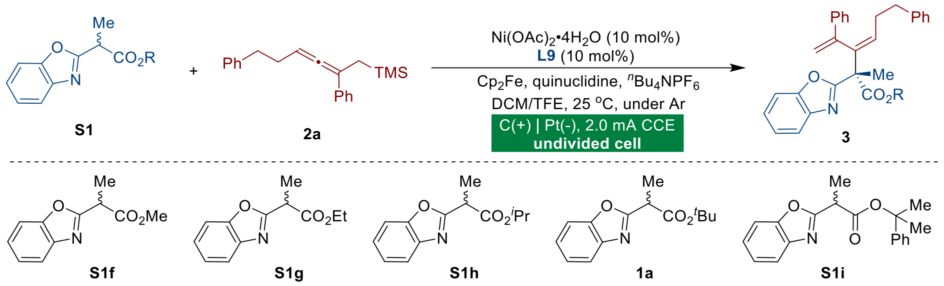

Entry	Substrates **S1**	**3**	Yield (%)	e.e. (%) of **3**
1	**S1f**	**3ab**	51	86
2	**S1g**	**3ac**	54	88
3	**S1h**	**3ad**	56	89
4	**1a**	**3a**	79	96
5	**S1i**	**3ae**	62	98

[a]Reactions were carried out with **S1** (0.1 mmol), **2a** (0.3 mmol), ^{n}Bu$_4$NPF$_6$ (0.15 mmol), quinuclidine (0.05 mmol), Ni(OAc)$_2$·4H$_2$O (10 mol%), **L9** (10 mol%), Cp$_2$Fe (10 mol%) and DCM/TFE = 2:1 (3 mL) at 25 °C in an undivided cell. TFE, 2,2,2-trifluoroethanol.

Table S4: Survey of benzoxazole[a]

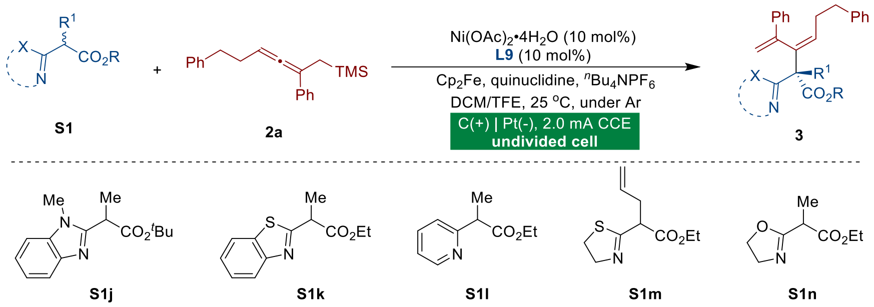

Entry	Substrates **S1**	**3**	Yield (%)	e.e. (%) of **3**
1	**S1j**	—	nr	—
2	**S1k**	—	nr	—
3	**S1l**	—	nr	—
4	**S1m**	—	nr	—
5	**S1n**	**3af**	56	28

[a]Reactions were carried out with **S1** (0.1 mmol), **2a** (0.3 mmol), ^{n}Bu$_4$NPF$_6$ (0.15 mmol), quinuclidine (0.05 mmol), Ni(OAc)$_2$·4H$_2$O (10 mol%), **L9** (10 mol%), Cp$_2$Fe (10 mol%) and DCM/TFE = 2:1 (3 mL) at 25 °C in an undivided cell. TFE, 2,2,2-trifluoroethanol. nr = no reaction.

Table S5**:** Survey of the effect of sterics around carbon for oxidative dienylation reactions[a]

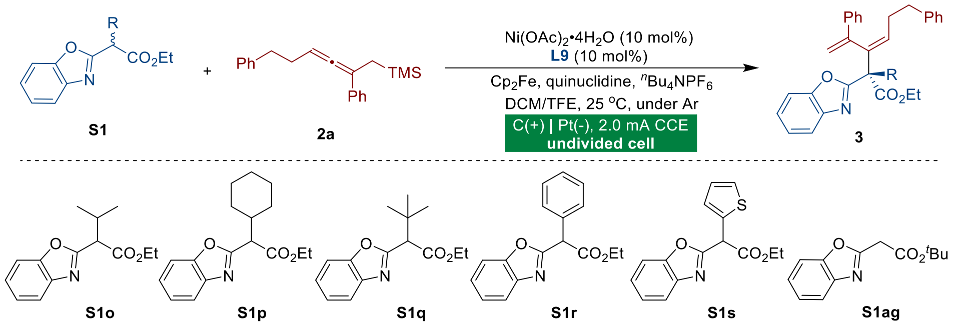

Entry	Substrates **S1**	**3**	Yield (%)	e.e. (%) of **3** (Z/E)
1	**S1o**	—	nr	—
2	**S1p**	—	nr	—
3	**S1q**	—	nr	—
4	**S1r**	—	nr	—
5	**S1s**	—	nr	—
6	**S1ag**	**3ag**	36 %	Racemic (> 20:1)

[a]Reactions were carried out with **S1** (0.1 mmol), **2a** (0.3 mmol), ^{n}Bu$_4$NPF$_6$ (0.15 mmol), quinuclidine (0.05 mmol), Ni(OAc)$_2$·4H$_2$O (10 mol%), **L9** (10 mol%), Cp$_2$Fe (10 mol%) and DCM/TFE = 2:1 (3 mL) at 25 °C in an undivided cell. TFE, 2,2,2-trifluoroethanol. nr = no reaction.

Table S6**:** Survey of the effect of sterics around carbon for oxidative allylation reactions[a]

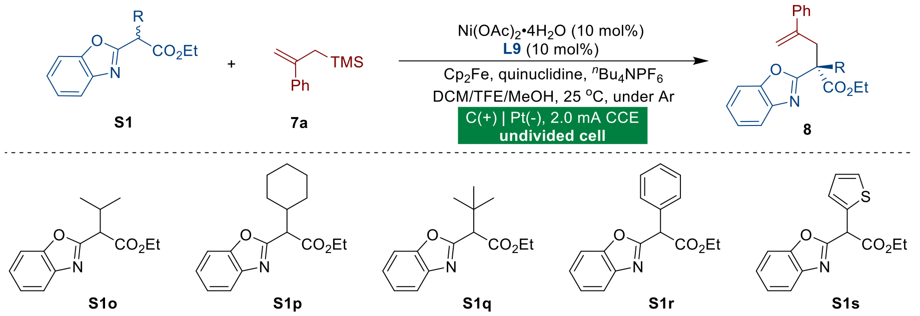

Entry	Substrates **S1**	**8**	Yield (%)	e.e. (%) of **8**
1[b]	**S1o**	**8w**	62	60
2[b]	**S1p**	**8x**	67	61
3	**S1q**	—	nr	—
4	**S1r**	—	nr	—
5	**S1s**	—	nr	—

[a]Reactions were carried out with **S1** (0.1 mmol), **7a** (0.3 mmol), ^{n}Bu$_4$NPF$_6$ (0.15 mmol), quinuclidine (0.05 mmol), Ni(OAc)$_2$·4H$_2$O (10 mol%), **L9** (10 mol%), Cp$_2$Fe (10 mol%) and DCM/TFE/MeOH = 3:2:1 (3 mL) at 25 °C in an undivided cell. [b]**7a** (0.6 mmol), 50 °C. TFE, 2,2,2-trifluoroethanol. nr = no reaction.

Table S7: Survey of the leaving group effect for oxidative dienylation reactions[a]

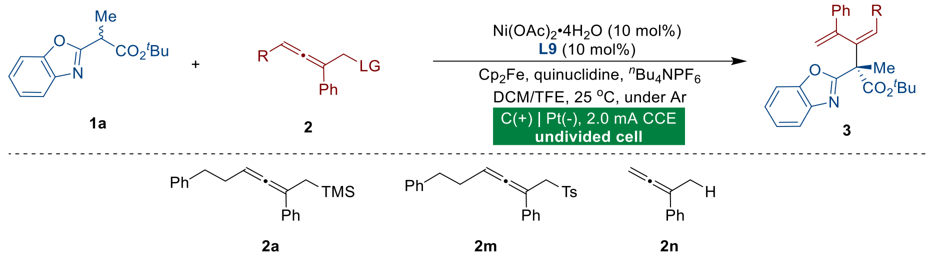

Entry	Substrates **2**	**3**	Yield (%)	e.e. (%) of **3**
1	**2a**	**3a**	79	96
2	**2m**	**3a**	< 5	91
3	**2n**	**3f**	42	94

^{a}Reactions were carried out with **1a** (0.1 mmol), **2** (0.3 mmol), ^{n}Bu$_4$NPF$_6$ (0.15 mmol), quinuclidine (0.05 mmol), Ni(OAc)$_2$·4H$_2$O (10 mol%), **L9** (10 mol%), Cp$_2$Fe (10 mol%) and DCM/TFE = 2:1 (3 mL) at 25 °C in an undivided cell. TFE, 2,2,2-trifluoroethanol.

Table S8: Survey of the leaving group effect for oxidative allylation reactions[a]

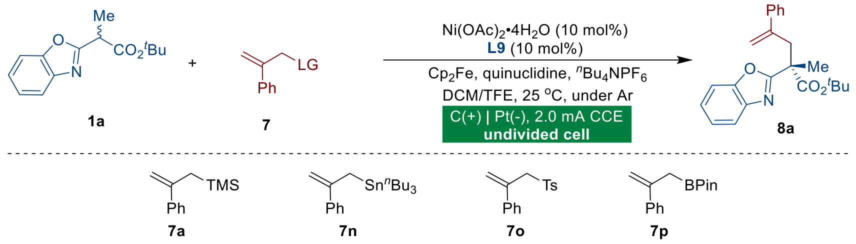

Entry	Substrates **7**	Yield (%)	e.e. (%) of **8a**
1	**7a**	84	94
2	**7n**	81	92
3	**7o**	52	92
4	**7p**	27	88

^{a}Reactions were carried out with **1a** (0.1 mmol), **7** (0.3 mmol), ^{n}Bu$_4$NPF$_6$ (0.15 mmol), quinuclidine (0.05 mmol), Ni(OAc)$_2$·4H$_2$O (10 mol%), **L9** (10 mol%), Cp$_2$Fe (10 mol%) and DCM/TFE/MeOH = 3:2:1 (3 mL) at 25 °C in an undivided cell. TFE, 2,2,2-trifluoroethanol.

Table S9: Survey of solvents[a]

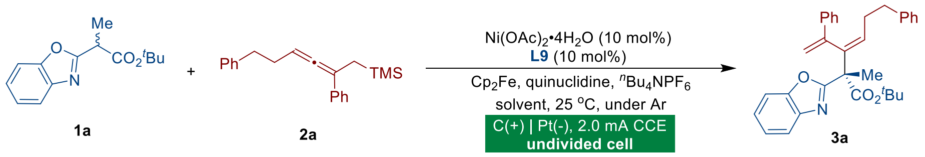

Entry	Solvent (3 mL)	Yield (%)	e.e. (%) of **3a**
1	DCM	16	53
2	DCM/MeOH = 1:1	26	68
3	THF/MeOH = 1:1	13	39
4	MeCN/MeOH = 1:1	8	36
5	DCM/TFE = 1:1	65	97
6	DCM/TFE = 2:1	79	96

^{a}Reactions were carried out with **1a** (0.1 mmol), **2a** (0.3 mmol), ^{n}Bu$_4$NPF$_6$ (0.15 mmol), quinuclidine (0.05 mmol), Ni(OAc)$_2$·4H$_2$O (10 mol%), **L9** (10 mol%) and Cp$_2$Fe (10 mol%) at 25 °C in an undivided cell. TFE, 2,2,2-trifluoroethanol.

***Table S10*: Survey of base**[a]

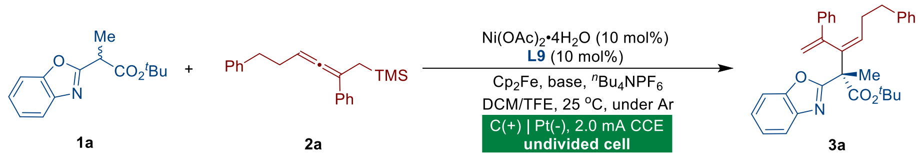

Entry	Base (0.05 mmol)	Yield (%)	e.e. (%) of **3a**
1	No base	51	96
2	2,6-dimethyoxypyridine	49	96
3	Et_3N	74	96
4	DMAP	57	95
5	quinuclidine	79	96
6	K_2CO_3	73	95

[a]Reactions were carried out with **1a** (0.1 mmol), **2a** (0.3 mmol), $^{n}Bu_4NPF_6$ (0.15 mmol), base (0.05 mmol), $Ni(OAc)_2·4H_2O$ (10 mol%), **L9** (10 mol%), Cp_2Fe (10 mol%) and DCM/TFE = 2:1 (3 mL) at 25 °C in an undivided cell. TFE, 2,2,2-trifluoroethanol.

***Table S11*: Survey of temperature**[a]

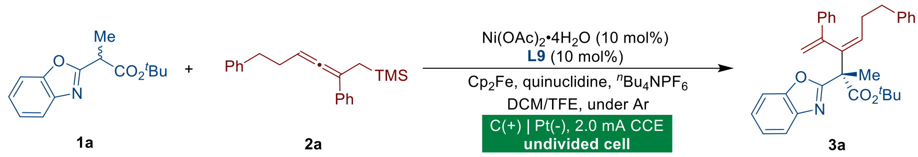

Entry	Temperature (°C)	Yield (%)	e.e. (%) of **3a**
1	25	79	96
2	35	76	96
3	10	53	96
4	0	27	97
5	-10	22	97

[a]Reactions were carried out with **1a** (0.1 mmol), **2a** (0.3 mmol), $^{n}Bu_4NPF_6$ (0.15 mmol), quinuclidine (0.05 mmol), $Ni(OAc)_2·4H_2O$ (10 mol%), **L9** (10 mol%), Cp_2Fe (10 mol%) and DCM/TFE = 2:1 (3 mL) in an undivided cell. TFE, 2,2,2-trifluoroethanol.

***Table S12*: Survey of electrolyte**[a]

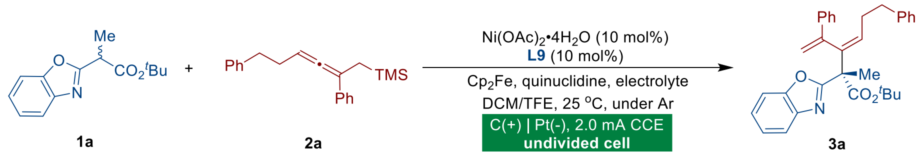

Entry	Electrolyte (0.15 mmol)	Yield (%)	e.e. (%) of **3a**
1	$^{n}Bu_4NPF_6$	79	96
2	$^{n}Bu_4NBF_6$	63	97
3	$^{n}Bu_4NBr$	69	96
4	$^{n}Bu_4NClO_4$	63	96
5	$^{n}Bu_4NOAc$	30	92

[a]Reactions were carried out with **1a** (0.1 mmol), **2a** (0.3 mmol), electrolyte (0.15 mmol), quinuclidine (0.05 mmol), $Ni(OAc)_2·4H_2O$ (10 mol%), **L9** (10 mol%), Cp_2Fe (10 mol%) and DCM/TFE = 2:1 (3 mL) at 25 °C in an undivided cell. TFE, 2,2,2-trifluoroethanol.

Table S13: Survey of anode[a]

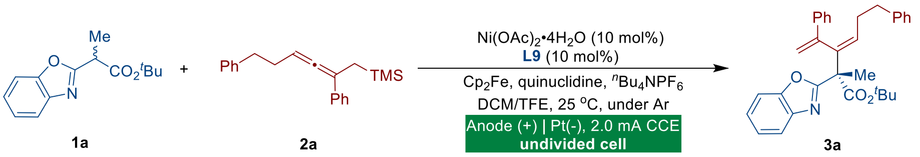

Entry	anode	Yield (%)	e.e. (%) of **3a**
1	Pt	67	96
2	C	79	96
3	BDD	65	96
4	RVC	55	97
5	Ni form	56	96

[a]Reactions were carried out with **1a** (0.1 mmol), **2a** (0.3 mmol), $^{n}Bu_4NPF_6$ (0.15 mmol), quinuclidine (0.05 mmol), $Ni(OAc)_2 \cdot 4H_2O$ (10 mol%), **L9** (10 mol%), Cp_2Fe (10 mol%) and DCM/TFE = 2:1 (3 mL) at 25 °C in an undivided cell. TFE, 2,2,2-trifluoroethanol.

Table S14: Survey of current[a]

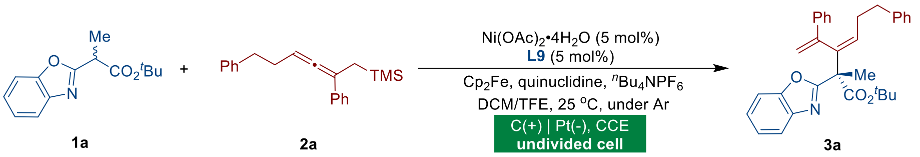

Entry	CCE	Yield (%)	e.e. (%) of **3a**
1	2.0 mA	76	96
2	4.0 mA	74	93
3	6.0 mA	51	92
4	8.0 mA	19	90
5	10.0 mA	16	88

[a]Reactions were carried out with **1a** (0.1 mmol), **2a** (0.3 mmol), $^{n}Bu_4NPF_6$ (0.15 mmol), quinuclidine (0.05 mmol), $Ni(OAc)_2 \cdot 4H_2O$ (5 mol%), **L9** (5 mol%), Cp_2Fe (10 mol%) and DCM/TFE = 2:1 (3 mL) at 25 °C under constant-current conditions in an undivided cell. TFE, 2,2,2-trifluoroethanol.

Table S15: Survey of voltage[a]

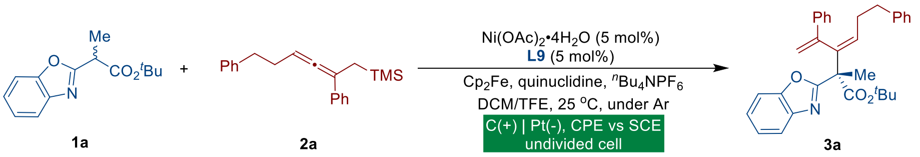

Entry	CPE (vs SCE)	Yield (%)	e.e. (%) of **3a**
1	0.4 V	67	96
2	1.0 V	72	95
3	2.5 V	12	92

[a]Reactions were carried out with **1a** (0.1 mmol), **2a** (0.3 mmol), $^{n}Bu_4NPF_6$ (0.15 mmol), quinuclidine (0.05 mmol), $Ni(OAc)_2 \cdot 4H_2O$ (5 mol%), **L9** (5 mol%), Cp_2Fe (10 mol%) and DCM/TFE = 2:1 (3 mL) at 25 °C under constant-voltage conditions in an undivided cell. TFE, 2,2,2-trifluoroethanol. CPE, constant potential electrolysis.

5. Synthesis and characterization of products

5.1 General procedure for the synthesis of products 3

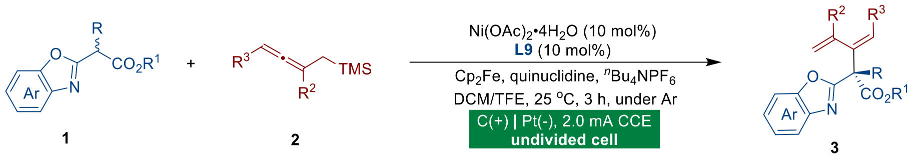

In a dried sealed tube, $Ni(OAc)_2{\cdot}4H_2O$ (2.48 mg, 0.01 mmol) and **L9** (3.6 mg, 0.01 mmol) were dissolved in DCM (1.0 mL) under N_2 atmosphere, and the mixture was stirred for 1 h at room temperature before use. A 10 mL flask equipped with a magnetic stir bar was charged with **1** (0.1 mmol), **2** (0.3 mmol), $^{n}Bu_4NPF_6$ (0.15 mmol), quinuclidine (0.05 mmol), Cp_2Fe (0.01 mmol). The flask was equipped with a carbon rod (d = 6 mm) as the anode and a platinum plate (1.0 cm × 1.0 cm × 0.2 mm) as the cathode. The reaction mixture was degassed via vacuum evacuation and backfilled with argon three times, followed by the addition via a syringe of nickel catalyst solution made in advance, DCM (1.0 mL) and TFE (1.0 mL). The constant current (2.0 mA) electrolysis was carried out at 25 ℃ for 3 h. After completion, The solution was diluted with ethyl acetate and washed with saturated NH_4Cl aqueous solution followed by distilled water washes. The aqueous phase was then extracted three times with ethyl acetate. The combined organic phase was dried over anhydrous $MgSO_4$ and concentrated under reduced pressure. The residue was purified by silica gel chromatography to afford the desired product **3**.

5.2 General procedure for the synthesis of products 8

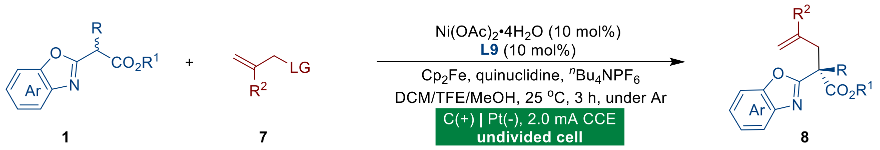

In a dried sealed tube, $Ni(OAc)_2{\cdot}4H_2O$ (2.5 mg, 0.01 mmol) and **L9** (3.6 mg, 0.01 mmol) were dissolved in DCM (1.0 mL) under N_2 atmosphere, and the mixture was stirred for 1 h at room temperature before use. A 10 mL flask equipped with a magnetic stir bar was charged with **1** (0.1 mmol), **7** (0.3 mmol), $^{n}Bu_4NPF_6$ (0.15 mmol), quinuclidine (0.05 mmol), Cp_2Fe (0.01 mmol). The flask was equipped with a carbon rod (d = 6 mm) as the anode and a platinum plate (1.0 cm × 1.0 cm × 0.2 mm) as the cathode. The reaction mixture was degassed via vacuum evacuation and backfilled with argon three times, followed by the addition via a syringe of nickel catalyst solution made in advance, DCM (0.5 mL), TFE (1.0 mL), and MeOH (0.5 mL). The constant current (2.0 mA) electrolysis was carried out at 25 ℃ for 3 h. After completion, The solution was diluted with ethyl acetate and washed with saturated NH_4Cl aqueous solution followed by distilled water washes. The aqueous phase was then extracted three times with ethyl acetate. The combined organic phase was dried over anhydrous $MgSO_4$ and concentrated under reduced pressure. The residue was purified by silica gel chromatography to afford the desired product **8**.

Analytical Data for the Products

Tert-butyl (*S*,*E*)-2-(benzo[d]oxazol-2-yl)-2-methyl-6-phenyl-3-(1-phenylvinyl)hex-3-enoate (3a)

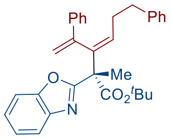

Reaction time: 3 h. **^{1}H NMR (400 MHz, CDCl$_3$)** δ 7.71 – 7.65 (m, 1H), 7.53 – 7.48 (m, 1H), 7.35 – 7.27 (m, 4H), 7.22 – 7.12 (m, 6H), 7.12 – 7.06 (m, 2H), 5.95 (t, *J* = 7.3 Hz, 1H), 5.60 (d, *J* = 1.4 Hz, 1H), 4.89 (d, *J* = 1.2 Hz, 1H), 2.70 (t, *J* = 7.5 Hz, 2H), 2.48 – 2.37 (m, 2H), 1.73 (s, 3H), 1.41 (s, 9H). **^{13}C NMR (100 MHz, CDCl$_3$)** δ 171.00,

166.64, 151.00, 144.62, 141.60, 141.07, 139.36, 138.61, 133.19, 128.70, 128.33, 128.28, 127.66, 126.23, 125.93, 124.82, 124.07, 120.16, 116.41, 110.62, 82.32, 55.83, 35.79, 32.04, 27.88, 22.69. **ESI-MS:** calculated $[C_{32}H_{33}NO_3 + Na]^+$: 502.2353, found: 502.2361. $[\alpha]^{20}_{D}$= +7.85 (c = 1.26, CH_2Cl_2). The product was analyzed by HPLC to determine the enantiomeric excess: 96% e.e. (CHIRALPAK IC, hexane/*i*-PrOH =95/5, detector: 254 nm, T = 25 °C, flow rate: 1 mL/min), t_1(major) = 4.60 min, t_2(minor) = 5.77 min.

Tert-butyl (*S*,*E*)-2-(benzo[d]oxazol-2-yl)-2-methyl-3-(1-phenylvinyl)pent-3-enoate (3b)

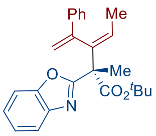

Reaction time: 3 h. **^{1}H NMR (400 MHz, CDCl3)** δ 7.70 – 7.64 (m, 1H), 7.51 – 7.45 (m, 1H), 7.42 – 7.36 (m, 2H), 7.33 – 7.26 (m, 2H), 7.23 – 7.11 (m, 3H), 5.99 (q, *J* = 6.7 Hz, 1H), 5.67 (d, *J* = 0.7 Hz, 1H), 5.06 (d, *J* = 0.8 Hz, 1H), 1.74 (s, 3H), 1.71 (d, *J* = 6.8 Hz, 3H), 1.42 (s, 9H). **^{13}C NMR (100 MHz, CDCl3)** δ 171.06, 166.80, 151.00, 144.72, 141.10, 139.42, 139.04, 128.37, 128.31, 127.61, 126.24, 124.80, 124.06, 120.16, 116.62, 110.63, 82.32, 56.02, 27.91, 22.76, 15.63. **ESI-MS:** calculated $[C_{25}H_{27}NO_3 + Na]^+$: 412.1883, found: 412.1893. $[\alpha]^{20}_{D}$= +6.65 (c = 0.89, CH_2Cl_2). The product was analyzed by HPLC to determine the enantiomeric excess: 95% e.e. (CHIRALPAK IC, hexane/*i*-PrOH =95/5, detector: 254 nm, T = 25 °C, flow rate: 1 mL/min), t_1(major) = 4.44 min, t_2(minor) = 5.69 min.

Tert-butyl (*S*,*E*)-2-(benzo[d]oxazol-2-yl)-2-methyl-3-(1-phenylvinyl)hept-3-enoate (3c)

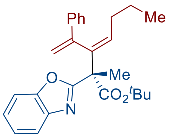

Reaction time: 3 h. **^{1}H NMR (400 MHz, CDCl3)** δ 7.71 – 7.62 (m, 1H), 7.50 – 7.44 (m, 1H), 7.43 – 7.36 (m, 2H), 7.33 – 7.26 (m, 2H), 7.22 – 7.10 (m, 3H), 5.91 (t, *J* = 7.3 Hz, 1H), 5.65 (d, *J* = 1.2 Hz, 1H), 5.01 (d, *J* = 0.9 Hz, 1H), 2.13 – 2.04 (m, 2H), 1.74 (s, 3H), 1.48 – 1.39 (m, 11H), 0.89 (t, *J* = 7.4 Hz, 3H). **^{13}C NMR (100 MHz, CDCl3)** δ 171.21, 166.76, 151.02, 144.82, 141.09, 139.46, 137.83, 134.38, 128.26, 127.63, 126.24, 124.78, 124.03, 120.14, 116.32, 110.61, 82.24, 55.91, 31.92, 27.92, 22.76, 22.64, 13.96. **ESI-MS:** calculated $[C_{27}H_{31}NO_3 + Na]^+$: 440.2196, found: 440.2204. $[\alpha]^{20}_{D}$= +6.13 (c = 0.98, CH_2Cl_2). The product was analyzed by HPLC to determine the enantiomeric excess: 96% e.e. (CHIRALPAK IC, hexane/*i*-PrOH =95/5, detector: 254 nm, T = 25 °C, flow rate: 1 mL/min), t_1(major) = 4.24 min, t_2(minor) = 5.26 min.

Tert-butyl (*S*,*E*)-2-(benzo[d]oxazol-2-yl)-2-methyl-3-(1-phenylvinyl)oct-3-enoate (3d)

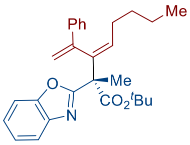

Reaction time: 3 h. **^{1}H NMR (400 MHz, CDCl3)** δ 7.69 – 7.63 (m, 1H), 7.51 – 7.44 (m, 1H), 7.42 – 7.36 (m, 2H), 7.32 – 7.26 (m, 2H), 7.22 – 7.11 (m, 3H), 5.90 (t, *J* = 7.3 Hz, 1H), 5.64 (d, *J* = 1.0 Hz, 1H), 5.00 (d, *J* = 0.9 Hz, 1H), 2.15 – 2.05 (m, 2H), 1.74 (s, 3H), 1.44 (s, 9H), 1.41 – 1.35 (m, 2H), 1.33 – 1.27 (m, 2H), 0.85 (t, *J* = 7.1 Hz, 3H). **^{13}C NMR (100 MHz, CDCl3)** δ 171.21, 166.78, 151.02, 144.85, 141.10, 139.50, 137.65, 134.55, 128.25, 127.62, 126.26, 124.78, 124.03, 120.15, 116.26, 110.61, 82.24, 55.90, 31.73, 29.67, 27.92, 22.65, 22.46, 14.12. **ESI-MS:** calculated $[C_{28}H_{33}NO_3 + Na]^+$: 454.2353, found: 454.2360. $[\alpha]^{20}_{D}$= +4.70 (c = 0.99, CH_2Cl_2). The product was analyzed by HPLC to determine the enantiomeric excess: 96% e.e. (CHIRALPAK IC, hexane/*i*-PrOH =95/5, detector: 254 nm, T = 25 °C, flow rate: 1 mL/min), t_1(major) = 4.18 min, t_2(minor) = 5.20 min.

Tert-butyl (*S*,*E*)-2-(benzo[d]oxazol-2-yl)-2-methyl-5-phenyl-3-(1-phenylvinyl)pent-3-enoate (3e)

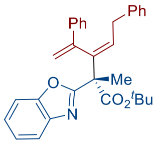

Reaction time: 3 h. **^{1}H NMR (400 MHz, $CDCl_3$)** δ 7.69 – 7.63 (m, 1H), 7.49 – 7.45 (m, 1H), 7.45 – 7.40 (m, 2H), 7.32 – 7.25 (m, 4H), 7.24 – 7.12 (m, 6H), 6.12 (t, *J* = 7.4 Hz, 1H), 5.70 (d, *J* = 1.0 Hz, 1H), 5.10 (d, *J* = 0.9 Hz, 1H), 3.48 (d, *J* = 7.4 Hz, 2H), 1.77 (s, 3H), 1.39 (s, 9H). **^{13}C NMR (100 MHz, $CDCl_3$)** δ 171.06, 166.54, 151.01, 144.47, 141.07, 140.48, 139.28, 138.94, 132.50, 128.56, 128.49, 128.37, 127.83, 126.27, 126.10, 124.89, 124.11, 120.19, 116.58, 110.61, 82.47, 55.97, 35.93, 27.87, 22.65. **ESI-MS:** calculated $[C_{31}H_{31}NO_3 + Na]^+$: 488.2196, found: 488.2191. $[\alpha]^{20}_{D}$= -6.49 (c = 0.93, CH_2Cl_2). The product was analyzed by HPLC to determine the enantiomeric excess: 97% e.e. (CHIRALPAK IC, hexane/*i*-PrOH =95/5, detector: 254 nm, T = 25 °C, flow rate: 1 mL/min), t_1(major) = 4.62 min, t_2(minor) = 5.79 min.

Tert-butyl (*S*)-2-(benzo[d]oxazol-2-yl)-2-methyl-3-methylene-4-phenylpent-4-enoate (3f)

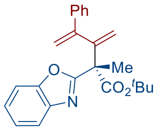

Reaction time: 3 h. **^{1}H NMR (400 MHz, $CDCl_3$)** δ 7.70 – 7.65 (m, 1H), 7.52 – 7.47 (m, 1H), 7.36 – 7.32 (m, 2H), 7.32 – 7.27 (m, 2H), 7.20 – 7.13 (m, 3H), 5.50 (d, *J* = 0.4 Hz, 1H), 5.41 (d, *J* = 0.4 Hz, 1H), 5.36 (d, *J* = 1.1 Hz, 1H), 5.22 (d, *J* = 1.1 Hz, 1H), 1.85 (s, 3H), 1.43 (s, 9H). **^{13}C NMR (100 MHz, $CDCl_3$)** δ 170.55, 166.19, 150.96, 148.57, 147.67, 140.99, 140.68, 128.07, 127.62, 127.37, 124.97, 124.18, 120.51, 120.23, 116.35, 110.68, 82.63, 55.38, 27.85, 23.21. **ESI-MS:** calculated $[C_{24}H_{25}NO_3 + Na]^+$: 398.1727, found: 398.1736. $[\alpha]^{20}_{D}$= +11.63 (c = 0.67, CH_2Cl_2). The product was analyzed by HPLC to determine the enantiomeric excess: 96% e.e. (CHIRALPAK IC, hexane/*i*-PrOH =99/1, detector: 254 nm, T = 25 °C, flow rate: 1 mL/min), t_1(major) = 7.18 min, t_2(minor) = 10.14 min.

Tert-butyl (*S*,*E*)-2-(benzo[d]oxazol-2-yl)-3-(1-(4-fluorophenyl)vinyl)-2-methylpent-3-enoate (3g)

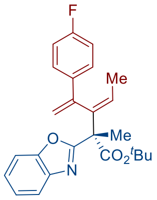

Reaction time: 3 h. **^{1}H NMR (400 MHz, $CDCl_3$)** δ 7.69 – 7.63 (m, 1H), 7.50 – 7.44 (m, 1H), 7.36 – 7.27 (m, 4H), 6.91 – 6.81 (m, 2H), 5.98 (q, *J* = 6.7 Hz, 1H), 5.60 (d, *J* = 0.8 Hz, 1H), 5.04 (d, *J* = 1.0 Hz, 1H), 1.74 (s, 3H), 1.70 (d, *J* = 6.8 Hz, 3H), 1.41 (s, 9H). **^{13}C NMR (100 MHz, $CDCl_3$)** δ 170.97, 166.62, 162.47 (d, *J* = 246.8 Hz), 150.94, 143.70, 141.01, 139.01, 135.52, 135.48, 128.46, 127.92 (d, *J* = 8.0 Hz), 124.90, 124.15, 120.16, 116.48, 115.11 (d, *J* = 21.5 Hz), 110.60, 82.43, 55.89, 27.91, 22.82, 15.60. **^{19}F NMR (376 MHz, $CDCl_3$)** δ -115.05. **ESI-MS:** calculated $[C_{25}H_{26}FNO_3 + Na]^+$: 430.1789, found: 430.1800. $[\alpha]^{20}_{D}$= +4.85 (c = 0.69, CH_2Cl_2). The product was analyzed by HPLC to determine the enantiomeric excess: 94% e.e. (CHIRALPAK IC, hexane/*i*-PrOH =95/5, detector: 254 nm, T = 25 °C, flow rate: 1 mL/min), t_1(major) = 4.13 min, t_2(minor) = 4.81 min.

Tert-butyl (*S*,*E*)-2-(benzo[d]oxazol-2-yl)-2-methyl-3-(1-(p-tolyl)vinyl)pent-3-enoate (3h)

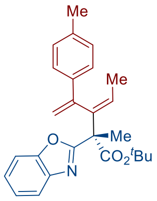

Reaction time: 3 h. **^{1}H NMR (400 MHz, $CDCl_3$)** δ 7.70 – 7.64 (m, 1H), 7.51 – 7.46 (m, 1H), 7.32 – 7.26 (m, 4H), 7.03 – 6.97 (m, 2H), 5.97 (q, *J* = 6.8 Hz, 1H), 5.63 (d, *J* = 1.1 Hz, 1H), 4.97 (d, *J* = 0.9 Hz, 1H), 2.26 (s, 3H), 1.73 (s, 3H), 1.70 (d, *J* = 6.8 Hz, 3H), 1.42 (s, 9H). **^{13}C NMR (100 MHz, $CDCl_3$)** δ 171.16, 166.85, 151.01, 144.47, 141.11, 139.10, 137.45, 136.61, 129.04, 128.19, 126.13, 124.78, 124.04, 120.14, 115.59, 110.66, 82.29, 56.04, 27.92, 22.64, 21.18, 15.63. **ESI-MS:** calculated $[C_{26}H_{29}NO_3 + H]^+$: 404.2220, found: 404.2221. $[\alpha]^{20}_{D}$= +7.60 (c = 0.54, CH_2Cl_2). The product was analyzed by HPLC to determine the enantiomeric excess: 96% e.e. (CHIRALPAK IC, hexane/*i*-PrOH =95/5, detector: 254 nm, T = 25 °C, flow rate: 1 mL/min), t_1(major) = 4.33 min, t_2(minor) = 5.56 min.

Tert-butyl (*S*,*E*)-2-(benzo[d]oxazol-2-yl)-3-(1-(3-chlorophenyl)vinyl)-2-methylpent-3-enoate (3i)

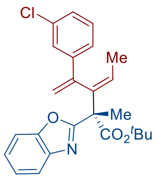

Reaction time: 3 h. **^{1}H NMR (400 MHz, $CDCl_3$)** δ 7.69 – 7.63 (m, 1H), 7.50 – 7.45 (m, 1H), 7.37 – 7.33 (m, 1H), 7.31 – 7.27 (m, 2H), 7.25 – 7.20 (m, 1H), 7.13 – 7.06 (m, 2H), 6.01 (q, *J* = 6.8 Hz, 1H), 5.68 (d, *J* = 0.9 Hz, 1H), 5.12 (d, *J* = 0.8 Hz, 1H), 1.75 (s, 3H), 1.70 (d, *J* = 6.8 Hz, 3H), 1.42 (s, 9H). **^{13}C NMR (100 MHz, $CDCl_3$)** δ 170.91, 166.48, 150.95, 143.60, 141.28, 140.98, 138.60, 134.32, 129.54, 128.85, 127.58, 126.41, 124.92, 124.40, 124.14, 120.15, 117.98, 110.68, 82.51, 55.84, 27.92, 22.86, 15.65. **ESI-MS:** calculated $[C_{25}H_{26}ClNO_3 + H]^+$: 424.1674, found: 424.1691. $[\alpha]^{20}_{D}$= +6.98 (c = 0.52, CH_2Cl_2). The product was analyzed by HPLC to determine the enantiomeric excess: 95% e.e. (CHIRALPAK IC, hexane/*i*-PrOH =95/5, detector: 254 nm, T = 25 °C, flow rate: 1 mL/min), t_1(major) = 4.11 min, t_2(minor) = 4.75 min.

Tert-butyl (*S*,*E*)-2-(benzo[d]oxazol-2-yl)-2-methyl-3-(1-(m-tolyl)vinyl)pent-3-enoate (3j)

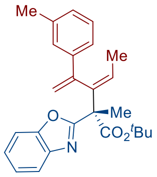

Reaction time: 3 h. **^{1}H NMR (400 MHz, $CDCl_3$)** δ 7.68 – 7.62 (m, 1H), 7.51 – 7.45 (m, 1H), 7.32 – 7.26 (m, 2H), 7.21 – 7.15 (m, 1H), 7.13 – 7.10 (m, 1H), 7.10 – 7.05 (m, 1H), 6.96 – 6.91 (m, 1H), 6.00 (q, *J* = 6.7 Hz, 1H), 5.65 (d, *J* = 1.3 Hz, 1H), 5.03 (d, *J* = 1.2 Hz, 1H), 2.18 (s, 3H), 1.76 – 1.70 (m, 6H), 1.42 (s, 9H). **^{13}C NMR (101 MHz, $CDCl_3$)** δ 171.18, 166.76, 151.01, 144.75, 141.10, 139.34, 139.18, 137.77, 128.36, 128.26, 128.22, 126.85, 124.78, 124.04, 123.47, 120.12, 116.60, 110.62, 82.28, 55.90, 27.92, 22.68, 21.48, 15.67. **ESI-MS:** calculated $[C_{26}H_{29}NO_3 + Na]^+$: 426.2040, found: 426.2038. $[\alpha]^{20}_{D}$= +13.57 (c = 0.67, CH_2Cl_2). The product was analyzed by HPLC to determine the enantiomeric excess: 95% e.e. (CHIRALPAK IC, hexane/*i*-PrOH =95/5, detector: 268 nm, T = 25 °C, flow rate: 1 mL/min), t_1(major) = 4.23 min, t_2(minor) = 5.19 min.

Tert-butyl (*S*)-2-(benzo[d]oxazol-2-yl)-2-methyl-3-methylene-4-(thiophen-3-yl)pent-4-enoate (3k)

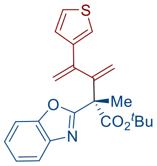

Reaction time: 3 h. **^{1}H NMR (400 MHz, $CDCl_3$)** δ 7.75 – 7.69 (m, 1H), 7.53 – 7.48 (m, 1H), 7.35 – 7.29 (m, 2H), 7.19 – 7.12 (m, 3H), 5.50 (d, *J* = 0.4 Hz, 1H), 5.45 (d, *J* = 0.5 Hz, 1H), 5.41 (d, *J* = 1.2 Hz, 1H), 5.09 (d, *J* = 1.2 Hz, 1H), 1.87 (s, 3H), 1.41 (s, 9H). **^{13}C NMR (100 MHz, $CDCl_3$)** δ 170.54, 166.37, 151.02, 147.49, 142.87, 142.39, 141.03, 126.23, 125.62, 125.03, 124.25, 123.00, 120.29, 119.93, 114.53, 110.68, 82.64, 55.50, 27.84, 23.02. **ESI-MS:** calculated $[C_{22}H_{23}NO_3S + Na]^+$: 404.1291, found: 404.1295. $[\alpha]^{20}_{D}$= -5.82 (c = 0.95, CH_2Cl_2). The product was analyzed by HPLC to determine the enantiomeric excess: 97% e.e. (CHIRALPAK IC, hexane/*i*-PrOH =95/5, detector: 254 nm, T = 25 °C, flow rate: 1 mL/min), t_1(major) = 4.83 min, t_2(minor) = 5.67 min.

Tert-butyl (*S*,*E*)-2-(benzo[d]oxazol-2-yl)-2-methyl-6-phenyl-3-(prop-1-en-2-yl)hex-3-enoate (3l)

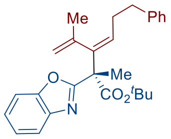

Reaction time: 3 h. **^{1}H NMR (400 MHz, $CDCl_3$)** δ 7.76 – 7.71 (m, 1H), 7.56 – 7.51 (m, 1H), 7.35 – 7.30 (m, 2H), 7.25 – 7.20 (m, 2H), 7.18 – 7.12 (m, 3H), 5.48 (t, *J* = 7.3 Hz, 1H), 5.02 – 4.96 (m, 1H), 4.59 (d, *J* = 1.5 Hz, 1H), 2.67 (t, *J* = 7.6 Hz, 2H), 2.46 – 2.37 (m, 2H), 1.84 (s, 3H), 1.63 (s, 3H), 1.42 (s, 9H). **^{13}C NMR (100 MHz, $CDCl_3$)** δ 170.88, 167.07, 151.00, 141.81, 141.69, 141.57, 141.16, 129.46, 128.66, 128.34, 125.93, 124.94, 124.20, 120.24, 116.75, 110.69, 82.33, 55.47, 36.09, 31.45, 27.91, 24.37, 22.73. **ESI-MS:** calculated $[C_{27}H_{31}NO_3 + Na]^+$: 440.2196, found: 440.2206. $[\alpha]^{20}_{D}$= -21.29 (c = 0.87, CH_2Cl_2). The product was analyzed by HPLC to determine the enantiomeric excess: 97% e.e. (CHIRALPAK IE, hexane/*i*-PrOH =99/1, detector: 254 nm, T = 25 °C, flow rate: 1 mL/min), t_1(major) = 7.45 min, t_2(minor)

= 9.57 min.

Tert-butyl (*S*)-2-(benzo[d]oxazol-2-yl)-2-ethyl-3-methylene-4-phenylpent-4-enoate (3m)

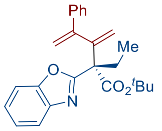

Reaction time: 3 h. **^{1}H NMR (400 MHz, CDCl$_3$)** δ 7.67 – 7.62 (m, 1H), 7.49 – 7.44 (m, 1H), 7.31 – 7.26 (m, 4H), 7.13 – 7.07 (m, 3H), 5.62 (d, *J* = 0.4 Hz, 1H), 5.40 (d, *J* = 0.4 Hz, 1H), 5.30 (d, *J* = 1.1 Hz, 1H), 5.18 (d, *J* = 1.1 Hz, 1H), 2.41 (dq, *J* = 14.6, 7.3 Hz, 1H), 2.27 (dq, *J* = 14.6, 7.3 Hz, 1H), 1.43 (s, 9H), 1.07 (t, *J* = 7.3 Hz, 3H). **^{13}C NMR (100 MHz, CDCl$_3$)** δ 169.43, 165.15, 150.64, 148.99, 147.19, 140.92, 140.58, 127.94, 127.49, 127.28, 124.83, 124.03, 120.72, 120.20, 116.27, 110.60, 82.48, 59.46, 29.72, 27.94, 9.96. **ESI-MS:** calculated [$C_{25}H_{27}NO_3$ + Na]$^+$: 412.1883, found: 412.1887. $[\alpha]^{20}_{D}$= +22.49 (c = 0.86, CH_2Cl_2). The product was analyzed by HPLC to determine the enantiomeric excess: 96% e.e. (CHIRALPAK IC, hexane/*i*-PrOH =99/1, detector: 254 nm, T = 25 °C, flow rate: 1 mL/min), t_1(major) = 4.99 min, t_2(minor) = 8.05 min.

Tert-butyl (*S*)-2-(benzo[d]oxazol-2-yl)-3-methylene-4-phenyl-2-propylpent-4-enoate (3n)

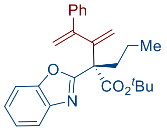

Reaction time: 3 h. **^{1}H NMR (400 MHz, CDCl$_3$)** δ 7.68 – 7.62 (m, 1H), 7.48 – 7.42 (m, 1H), 7.30 – 7.25 (m, 4H), 7.14 – 7.08 (m, 3H), 5.65 (d, *J* = 0.4 Hz, 1H), 5.40 (d, *J* = 0.4 Hz, 1H), 5.29 (d, *J* = 0.9 Hz, 1H), 5.19 (d, *J* = 1.0 Hz, 1H), 2.32 (td, *J* = 13.1, 4.6 Hz, 1H), 2.19 (td, *J* = 13.1, 4.6 Hz, 1H), 1.62 – 1.52 (m, 1H), 1.41 (s, 9H), 1.38 – 1.30 (m, 1H), 0.92 (t, *J* = 7.3 Hz, 3H). **^{13}C NMR (100 MHz, CDCl$_3$)** δ 169.42, 165.39, 150.58, 149.07, 147.06, 140.97, 140.72, 127.92, 127.46, 127.38, 124.80, 124.01, 120.84, 120.23, 116.28, 110.58, 82.40, 58.93, 38.68, 27.92, 18.63, 14.58. **ESI-MS:** calculated [$C_{26}H_{29}NO_3$ + Na]$^+$: 426.2040, found: 426.2044. $[\alpha]^{20}_{D}$= +14.17 (c = 0.61, CH_2Cl_2). The product was analyzed by HPLC to determine the enantiomeric excess: 95% e.e. (CHIRALPAK IF, hexane/*i*-PrOH =95/5, detector: 254 nm, T = 25 °C, flow rate: 1 mL/min), t_1(major) = 3.80 min, t_2(minor) = 6.07 min.

Tert-butyl (*S*)-2-(benzo[d]oxazol-2-yl)-2-(3-phenylbuta-1,3-dien-2-yl)hexanoate (3o)

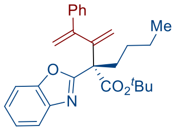

Reaction time: 3 h. **^{1}H NMR (400 MHz, CDCl$_3$)** δ 7.68 – 7.62 (m, 1H), 7.48 – 7.42 (m, 1H), 7.30 – 7.25 (m, 4H), 7.15 – 7.07 (m, 3H), 5.66 (d, *J* = 0.5 Hz, 1H), 5.41 (d, *J* = 0.5 Hz, 1H), 5.29 (d, *J* = 1.1 Hz, 1H), 5.19 (d, *J* = 1.2 Hz, 1H), 2.38 – 2.28 (m, 1H), 2.27 – 2.16 (m, 1H), 1.58 – 1.48 (m, 1H), 1.42 (s, 9H), 1.36 – 1.27 (m, 3H), 0.87 (t, *J* = 6.9 Hz, 3H). **^{13}C NMR (100 MHz, CDCl$_3$)** δ 169.41, 165.39, 150.57, 149.09, 147.04, 140.98, 140.72, 127.91, 127.46, 127.37, 124.79, 124.01, 120.86, 120.24, 116.32, 110.58, 82.37, 58.85, 36.30, 27.92, 27.34, 23.20, 14.06. **ESI-MS:** calculated [$C_{27}H_{31}NO_3$ + Na]$^+$: 440.2196, found: 440.2202. $[\alpha]^{20}_{D}$= +18.70 (c = 0.57, CH_2Cl_2). The product was analyzed by HPLC to determine the enantiomeric excess: 95% e.e. (CHIRALPAK IF, hexane/*i*-PrOH =95/5, detector: 254 nm, T = 25 °C, flow rate: 1 mL/min), t_1(major) = 3.75 min, t_2(minor) = 4.62 min.

Tert-butyl (*S*)-2-(benzo[d]oxazol-2-yl)-3-methylene-2-phenethyl-4-phenylpent-4-enoate (3p)

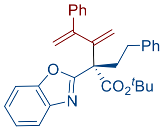

Reaction time: 6 h. **^{1}H NMR (400 MHz, CDCl$_3$)** δ 7.70 – 7.63 (m, 1H), 7.49 – 7.44 (m, 1H), 7.31 – 7.25 (m, 6H), 7.22 – 7.16 (m, 3H), 7.13 – 7.07 (m, 3H), 5.67 (d, *J* = 0.5 Hz, 1H), 5.44 (d, *J* = 0.5 Hz, 1H), 5.31 (d, *J* = 0.7 Hz, 1H), 5.25 (d, *J* = 0.7 Hz, 1H), 2.95 – 2.86 (m, 1H), 2.73 – 2.63 (m, 2H), 2.59 – 2.50 (m, 1H), 1.46 (s, 9H). **^{13}C NMR (100 MHz, CDCl$_3$)** δ 169.25, 165.00, 150.63, 148.95, 146.87, 141.96, 140.99, 140.61, 128.59, 128.50, 127.96, 127.52, 127.42, 126.05, 124.91, 124.10, 121.14, 120.32, 116.46, 110.62, 82.74, 58.80, 38.50,

31.69, 27.98. **ESI-MS:** calculated [$C_{31}H_{31}NO_3$ + Na]$^+$: 488.2196, found: 488.2205. $[\alpha]^{20}_D$= -6.53 (c = 0.85, CH_2Cl_2). The product was analyzed by HPLC to determine the enantiomeric excess: 95% e.e. (CHIRALPAK IF, hexane/*i*-PrOH =95/5, detector: 254 nm, T = 25 °C, flow rate: 1 mL/min), t_1(major) = 4.12 min, t_2(minor) = 5.06 min.

Tert-butyl (*S*)-2-(benzo[d]oxazol-2-yl)-2-(3-((tert-butyldimethylsilyl)oxy)propyl)-3-methylene-4-phenylpent-4-enoate (3q)

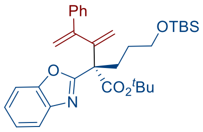

Reaction time: 3 h. **^{1}H NMR (400 MHz, CDCl$_3$)** δ 7.67 – 7.60 (m, 1H), 7.47 – 7.41 (m, 1H), 7.29 – 7.24 (m, 4H), 7.12 – 7.07 (m, 3H), 5.63 (d, *J* = 0.5 Hz, 1H), 5.39 (d, *J* = 0.4 Hz, 1H), 5.29 (d, *J* = 1.1 Hz, 1H), 5.19 (d, *J* = 1.0 Hz, 1H), 3.67 – 3.53 (m, 2H), 2.39 – 2.25 (m, 2H), 1.84 – 1.71 (m, 1H), 1.64 – 1.52 (m, 1H), 1.41 (s, 9H), 0.86 (s, 9H), 0.00 (s, 6H). **^{13}C NMR (100 MHz, CDCl$_3$)** δ 169.38, 165.20, 150.61, 148.91, 147.04, 140.99, 140.65, 127.95, 127.49, 127.36, 124.81, 124.02, 120.87, 120.25, 116.28, 110.60, 82.47, 63.22, 58.65, 33.13, 28.63, 27.91, 26.09, 18.45, -5.16, -5.20. **ESI-MS:** calculated [$C_{32}H_{43}NO_4Si$ + H]$^+$: 534.3034, found: 534.3051. $[\alpha]^{20}_D$= +12.45 (c = 1.14, CH_2Cl_2). The product was analyzed by HPLC to determine the enantiomeric excess: 96% e.e. (CHIRALPAK IF, hexane/*i*-PrOH =95/5, detector: 254 nm, T = 25 °C, flow rate: 1 mL/min), t_1(major) = 3.54 min, t_2(minor) = 7.42 min.

Tert-butyl (*S*)-2-allyl-2-(benzo[d]oxazol-2-yl)-3-methylene-4-phenylpent-4-enoate (3r)

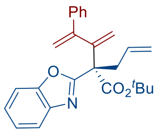

Reaction time: 3 h. **^{1}H NMR (400 MHz, CDCl$_3$)** δ 7.66 – 7.61 (m, 1H), 7.49 – 7.44 (m, 1H), 7.31 – 7.25 (m, 4H), 7.14 – 7.07 (m, 3H), 6.11 – 5.98 (m, 1H), 5.62 (d, *J* = 0.4 Hz, 1H), 5.43 (d, *J* = 0.4 Hz, 1H), 5.33 (d, *J* = 1.1 Hz, 1H), 5.21 (d, *J* = 1.1 Hz, 1H), 5.11 (dd, *J* = 17.0, 1.1 Hz, 1H), 5.05 (dd, *J* = 10.0, 1.4 Hz, 1H), 3.15 (dd, *J* = 14.1, 5.9 Hz, 1H), 2.95 (dd, *J* = 14.1, 8.1 Hz, 1H), 1.40 (s, 9H). **^{13}C NMR (100 MHz, CDCl$_3$)** δ 168.93, 164.88, 150.73, 148.80, 146.69, 140.89, 140.42, 133.63, 127.97, 127.56, 127.30, 124.90, 124.07, 121.02, 120.24, 118.91, 116.55, 110.62, 82.72, 58.79, 40.93, 27.96. **ESI-MS:** calculated [$C_{26}H_{27}NO_3$ + Na]$^+$: 424.1883, found: 424.1894. $[\alpha]^{20}_D$= +38.37 (c = 0.85, CH_2Cl_2). The product was analyzed by HPLC to determine the enantiomeric excess: 92% e.e. (CHIRALPAK IF, hexane/*i*-PrOH =95/5, detector: 254 nm, T = 25 °C, flow rate: 1 mL/min), t_1(major) = 3.91 min, t_2(minor) = 5.21 min.

Ethyl (*S*)-2-(benzo[d]oxazol-2-yl)-2-methyl-3-methylene-4-phenylpent-4-enoate (3s)

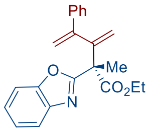

Reaction time: 3 h. **^{1}H NMR (400 MHz, CDCl$_3$)** δ 7.71 – 7.64 (m, 1H), 7.52 – 7.46 (m, 1H), 7.36 – 7.28 (m, 4H), 7.21 – 7.13 (m, 3H), 5.45 (d, *J* = 0.4 Hz, 1H), 5.44 (d, *J* = 0.4 Hz, 1H), 5.36 (d, *J* = 1.1 Hz, 1H), 5.24 (d, *J* = 1.0 Hz, 1H), 4.16 (q, *J* = 7.1 Hz, 2H), 1.89 (s, 3H), 1.21 (t, *J* = 7.1 Hz, 3H). **^{13}C NMR (100 MHz, CDCl$_3$)** δ 171.39, 165.77, 150.95, 148.64, 147.48, 140.94, 140.41, 128.07, 127.67, 127.30, 125.09, 124.28, 120.55, 120.29, 116.74, 110.76, 62.11, 54.71, 23.21, 14.03. **ESI-MS:** calculated [$C_{22}H_{21}NO_3$ + H]$^+$: 348.1594, found: 348.1600. $[\alpha]^{20}_D$= +8.70 (c = 0.59, CH_2Cl_2). The product was analyzed by HPLC to determine the enantiomeric excess: 88% e.e. (CHIRALPAK IB N-5, hexane/*i*-PrOH =95/5, detector: 254 nm, T = 25 °C, flow rate: 1 mL/min), t_1(minor) = 5.10 min, t_2(major) = 7.44 min.

Isopropyl (*S*)-2-(benzo[d]oxazol-2-yl)-2-methyl-3-methylene-4-phenylpent-4-enoate (3t)

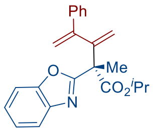

Reaction time: 3 h. **^{1}H NMR (400 MHz, $CDCl_3$)** δ 7.71 – 7.64 (m, 1H), 7.52 – 7.46 (m, 1H), 7.37 – 7.28 (m, 4H), 7.21 – 7.11 (m, 3H), 5.48 (d, *J* = 0.5 Hz, 1H), 5.42 (d, *J* = 0.5 Hz, 1H), 5.36 (d, *J* = 1.0 Hz, 1H), 5.23 (d, *J* = 0.8 Hz, 1H), 5.12 – 5.00 (m, 1H), 1.87 (s, 3H), 1.23 (d, *J* = 6.3 Hz, 3H), 1.19 (d, *J* = 6.3 Hz, 3H). **^{13}C NMR (100 MHz, $CDCl_3$)** δ 170.96, 165.92, 150.96, 148.63, 147.46, 140.97, 140.57, 128.06, 127.64, 127.34, 125.03, 124.23, 120.61, 120.27, 116.55, 110.71, 69.83, 54.73, 23.15, 21.63, 21.52. **ESI-MS:** calculated $[C_{23}H_{23}NO_3 + H]^+$: 362.1751, found: 362.1751. $[\alpha]^{20}_D$= +27.73 (c = 0.67, CH_2Cl_2). The product was analyzed by HPLC to determine the enantiomeric excess: 86% e.e. (CHIRALPAK IF, hexane/*i*-PrOH =99/1, detector: 254 nm, T = 25 °C, flow rate: 1 mL/min), t_1(major) = 8.10 min, t_2(minor) = 8.77 min.

Ethyl (*S*)-2-methyl-2-(5-methylbenzo[d]oxazol-2-yl)-3-methylene-4-phenylpent-4-enoate (3u)

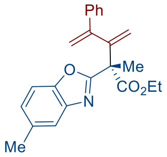

Reaction time: 3 h. **^{1}H NMR (400 MHz, $CDCl_3$)** δ 7.49 – 7.44 (m, 1H), 7.38 – 7.31 (m, 3H), 7.22 – 7.16 (m, 3H), 7.14 – 7.10 (m, 1H), 5.43 (d, *J* = 0.4 Hz, 1H), 5.41 (d, *J* = 0.4 Hz, 1H), 5.35 (d, *J* = 1.0 Hz, 1H), 5.21 (d, *J* = 1.0 Hz, 1H), 4.14 (q, *J* = 7.1 Hz, 2H), 2.45 (s, 3H), 1.87 (s, 3H), 1.20 (t, *J* = 7.1 Hz, 3H). **^{13}C NMR (100 MHz, $CDCl_3$)** δ 171.44, 165.87, 149.22, 148.64, 147.53, 141.18, 140.55, 134.08, 128.10, 127.69, 127.36, 126.18, 120.55, 120.19, 116.64, 110.12, 62.07, 54.78, 23.21, 21.61, 14.03. **ESI-MS:** calculated $[C_{23}H_{23}NO_3 + H]^+$: 362.1751, found: 362.1758. $[\alpha]^{20}_D$= +6.25 (c = 0.73, CH_2Cl_2). The product was analyzed by HPLC to determine the enantiomeric excess: 90% e.e. (CHIRALPAK IC, hexane/*i*-PrOH =95/5, detector: 254 nm, T = 25 °C, flow rate: 1 mL/min), t_1(major) = 7.20 min, t_2(minor) = 12.43 min.

Ethyl (*S*)-2-(5-(tert-butyl)benzo[d]oxazol-2-yl)-2-methyl-3-methylene-4-phenylpent-4-enoate (3v)

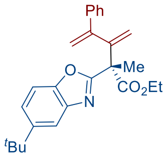

Reaction time: 3 h. **^{1}H NMR (400 MHz, $CDCl_3$)** δ 7.75 – 7.70 (m, 1H), 7.43 – 7.33 (m, 4H), 7.23 – 7.14 (m, 3H), 5.44 (d, *J* = 0.5 Hz, 1H), 5.41 (d, *J* = 0.5 Hz, 1H), 5.36 (d, *J* = 1.1 Hz, 1H), 5.23 (d, *J* = 1.1 Hz, 1H), 4.15 (q, *J* = 7.1 Hz, 2H), 1.87 (s, 3H), 1.37 (s, 9H), 1.21 (t, *J* = 7.1 Hz, 3H). **^{13}C NMR (100 MHz, $CDCl_3$)** δ 171.40, 165.97, 148.99, 148.60, 147.81, 147.52, 140.83, 140.58, 128.09, 127.64, 127.35, 122.78, 120.60, 116.84, 116.64, 109.88, 62.06, 54.78, 35.02, 31.90, 23.26, 14.04. **ESI-MS:** calculated $[C_{26}H_{29}NO_3 + H]^+$: 404.2220, found: 404.2230. $[\alpha]^{20}_D$= +1.01 (c = 0.82, CH_2Cl_2). The product was analyzed by HPLC to determine the enantiomeric excess: 90% e.e. (CHIRALPAK IC, hexane/*i*-PrOH =95/5, detector: 254 nm, T = 25 °C, flow rate: 1 mL/min), t_1(major) = 4.99 min, t_2(minor) = 8.48 min.

Ethyl (*S*)-2-(5-methoxybenzo[d]oxazol-2-yl)-2-methyl-3-methylene-4-phenylpent-4-enoate (3w)

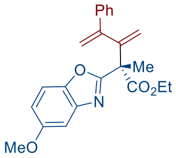

Reaction time: 3 h. **^{1}H NMR (400 MHz, $CDCl_3$)** δ 7.38 – 7.30 (m, 3H), 7.21 – 7.13 (m, 4H), 6.94 – 6.88 (m, 1H), 5.43 (d, *J* = 0.4 Hz, 1H), 5.42 (d, *J* = 0.5 Hz, 1H), 5.35 (d, *J* = 0.9 Hz, 1H), 5.22 (d, *J* = 0.8 Hz, 1H), 4.15 (q, *J* = 7.1 Hz, 2H), 3.83 (s, 3H), 1.86 (s, 3H), 1.21 (t, *J* = 7.1 Hz, 3H). **^{13}C NMR (100 MHz, $CDCl_3$)** δ 171.41, 166.52, 157.19, 148.66, 147.54, 145.62, 141.75, 140.46, 128.08, 127.68, 127.33, 120.50, 116.68, 113.77, 110.91, 103.16, 62.09, 56.04, 54.78, 23.18, 14.04. **ESI-MS:** calculated $[C_{23}H_{23}NO_4 + H]^+$: 378.1700, found: 378.1704. $[\alpha]^{20}_D$= +6.99 (c = 1.13, CH_2Cl_2). The product was analyzed by HPLC to determine the enantiomeric excess: 85% e.e. (CHIRALPAK IC, hexane/*i*-PrOH =85/15, detector: 254 nm, T = 25 °C, flow rate: 1 mL/min), t_1(major) = 5.84 min, t_2(minor) = 8.21 min.

Ethyl (*S*)-2-methyl-2-(6-methylbenzo[d]oxazol-2-yl)-3-methylene-4-phenylpent-4-enoate (3x)

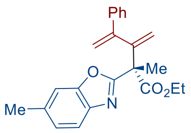

Reaction time: 3 h. **^{1}H NMR (400 MHz, CDCl$_3$)** δ 7.58 – 7.52 (m, 1H), 7.38 – 7.29 (m, 3H), 7.23 – 7.15 (m, 3H), 7.14 – 7.08 (m, 1H), 5.43 (d, *J* = 0.5 Hz, 1H), 5.41 (d, *J* = 0.5 Hz, 1H), 5.35 (d, *J* = 1.0 Hz, 1H), 5.22 (d, *J* = 1.1 Hz, 1H), 4.14 (q, *J* = 7.1 Hz, 2H), 2.48 (s, 3H), 1.87 (s, 3H), 1.21 (t, *J* = 7.1 Hz, 3H). **^{13}C NMR (100 MHz, CDCl$_3$)** δ 171.45, 165.23, 151.27, 148.64, 147.54, 140.55, 138.75, 135.47, 128.09, 127.67, 127.35, 125.49, 120.53, 119.61, 116.62, 110.92, 62.05, 54.72, 23.21, 21.88, 14.03. **ESI-MS:** calculated [$C_{23}H_{23}NO_3$ + H]$^+$: 362.1751, found: 362.1758. $[\alpha]^{20}_{D}$= +10.70 (c = 0.69, CH_2Cl_2).The product was analyzed by HPLC to determine the enantiomeric excess: 89% e.e. (CHIRALPAK IC, hexane/*i*-PrOH =95/5, detector: 254 nm, T = 25 °C, flow rate: 1 mL/min), t_1(major) = 8.55 min, t_2(minor) = 9.92 min.

Ethyl (*S*)-2-methyl-2-(7-methylbenzo[d]oxazol-2-yl)-3-methylene-4-phenylpent-4-enoate (3y)

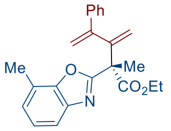

Reaction time: 3 h. **^{1}H NMR (400 MHz, CDCl$_3$)** δ 7.53 – 7.48 (m, 1H), 7.37 – 7.31 (m, 2H), 7.22 – 7.13 (m, 4H), 7.12 – 7.07 (m, 1H), 5.47 (d, *J* = 0.4 Hz, 1H), 5.42 (d, *J* = 0.5 Hz, 1H), 5.36 (d, *J* = 1.1 Hz, 1H), 5.26 (d, *J* = 1.1 Hz, 1H), 4.15 (q, *J* = 7.1 Hz, 2H), 2.48 (s, 3H), 1.91 (s, 3H), 1.21 (t, *J* = 7.1 Hz, 3H). **^{13}C NMR (100 MHz, CDCl$_3$)** δ 171.40, 165.46, 150.16, 148.76, 147.69, 140.50, 140.41, 128.00, 127.60, 127.28, 126.00, 124.14, 121.19, 120.46, 117.55, 116.64, 61.99, 54.69, 23.44, 15.22, 14.03. **ESI-MS:** calculated [$C_{23}H_{23}NO_3$ + H]$^+$: 362.1751, found: 362.1753. $[\alpha]^{20}_{D}$= +4.13 (c = 0.75, CH_2Cl_2). The product was analyzed by HPLC to determine the enantiomeric excess: 92% e.e. (CHIRALPAK IC, hexane/*i*-PrOH =95/5, detector: 254 nm, T = 25 °C, flow rate: 1 mL/min), t_1(major) = 7.15 min, t_2(minor) = 10.76 min.

(*S*,*E*)-2-(benzo[d]oxazol-2-yl)-2-methyl-1,6-diphenyl-3-(1-phenylvinyl)hex-3-en-1-one (3z)

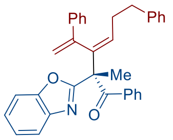

Reaction time: 3 h. **^{1}H NMR (400 MHz, CDCl$_3$)** δ 7.88 – 7.83 (m, 2H), 7.62 – 7.58 (m, 1H), 7.51 – 7.45 (m, 2H), 7.40 – 7.34 (m, 2H), 7.34 – 7.27 (m, 2H), 7.21 – 7.13 (m, 3H), 7.11 – 7.07 (m, 2H), 7.07 – 7.03 (m, 3H), 7.02 – 6.97 (m, 2H), 6.06 (t, *J* = 7.2 Hz, 1H), 5.59 (d, *J* = 1.3 Hz, 1H), 5.03 (d, *J* = 1.1 Hz, 1H), 2.77 – 2.66 (m, 2H), 2.62 – 2.50 (m, 2H), 1.82 (s, 3H). **^{13}C NMR (100 MHz, CDCl$_3$)** δ 198.60, 166.78, 150.91, 145.35, 141.54, 141.08, 139.54, 138.28, 135.93, 135.56, 132.56, 130.06, 128.70, 128.42, 128.24, 127.72, 126.40, 126.02, 124.88, 124.16, 120.15, 117.89, 110.76, 59.59, 35.80, 32.52, 22.99. **ESI-MS:** calculated [$C_{34}H_{29}NO_2$ + H]$^+$: 484.2271, found: 484.2285. $[\alpha]^{20}_{D}$ = +33.40 (c = 0.59, CH_2Cl_2). The product was analyzed by HPLC to determine the enantiomeric excess: 78% e.e. (CHIRALPAK IC, hexane/*i*-PrOH =80/20, detector: 254 nm, T = 25 °C, flow rate: 1 mL/min), t_1(major) = 4.87 min, t_2(minor) = 9.52 min.

(*S*,*E*)-2-(benzo[d]oxazol-2-yl)-*N*,2-dimethyl-6-phenyl-3-(1-phenylvinyl)hex-3-enamide (3aa)

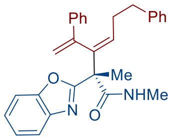

Reaction time: 3 h. **^{1}H NMR (400 MHz, CDCl$_3$)** δ 8.39 (q, *J* = 4.1 Hz, 1H), 7.62 – 7.52 (m, 1H), 7.42 – 7.35 (m, 1H), 7.32 – 7.26 (m, 3H), 7.25 – 7.20 (m, 2H), 7.19 – 7.14 (m, 1H), 7.14 – 7.08 (m, 2H), 7.06 – 7.00 (m, 4H), 5.94 (t, *J* = 7.2 Hz, 1H), 5.41 (d, *J* = 1.4 Hz, 1H), 4.68 (d, *J* = 1.5 Hz, 1H), 2.82 – 2.65 (m, 5H), 2.49 – 2.37 (m, 2H), 1.82 (s, 3H). **^{13}C NMR (100 MHz, CDCl$_3$)** δ 170.26, 166.98, 149.98, 145.09, 141.62, 140.62, 140.04, 138.92, 131.25, 128.82, 128.38, 127.96, 127.38, 126.10, 126.01, 125.11, 124.39, 119.78, 117.11, 110.66, 54.29, 35.93, 31.93, 26.66, 22.23. **ESI-MS:** calculated [$C_{29}H_{28}N_2O_2$ + H]$^+$: 437.2224, found: 437.2228. $[\alpha]^{20}_{D}$ = -20.80 (c = 0.52, CH_2Cl_2). The product was analyzed by HPLC to determine the enantiomeric excess: 94% e.e. (CHIRALPAK IE, hexane/*i*-PrOH =80/20, detector: 254 nm, T = 25 °C, flow rate: 1 mL/min), t_1(major) = 8.80 min, t_2(minor) = 9.93 min.

Methyl (*S*,*E*)-2-(benzo[d]oxazol-2-yl)-2-methyl-6-phenyl-3-(1-phenylvinyl)hex-3-enoate (3ab)

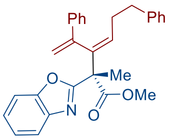

Reaction time: 3 h. **^{1}H NMR (400 MHz, $CDCl_3$)** δ 7.73 – 7.63 (m, 1H), 7.54 – 7.46 (m, 1H), 7.37 – 7.30 (m, 2H), 7.29 – 7.26 (m, 1H), 7.25 – 7.24 (m, 1H), 7.23 – 7.18 (m, 2H), 7.19 – 7.12 (m, 4H), 7.11 – 7.04 (m, 2H), 5.83 (t, *J* = 7.3 Hz, 1H), 5.59 (d, *J* = 0.9 Hz, 1H), 4.91 (d, *J* = 1.1 Hz, 1H), 3.65 (s, 3H), 2.70 (t, *J* = 7.4 Hz, 2H), 2.52 – 2.38 (m, 2H), 1.76 (s, 3H). **^{13}C NMR (100 MHz, $CDCl_3$)** δ 172.21, 166.05, 150.94, 144.59, 141.55, 140.97, 139.22, 138.52, 133.24, 128.78, 128.33, 128.29, 127.70, 126.25, 125.97, 125.03, 124.24, 120.27, 117.20, 110.75, 55.24, 52.88, 35.82, 32.07, 22.84. **ESI-MS:** calculated $[C_{29}H_{27}NO_3 + H]^+$: 438.2064, found: 438.2081. $[\alpha]^{20}_D$= +11.90 (c = 0.74, CH_2Cl_2). The product was analyzed by HPLC to determine the enantiomeric excess: 86% e.e. (CHIRALPAK IC, hexane/*i*-PrOH =95/5, detector: 254 nm, T = 25 °C, flow rate: 1 mL/min), t_1(major) = 7.70 min, t_2(minor) = 16.78 min.

Ethyl (*S*,*E*)-2-(benzo[d]oxazol-2-yl)-2-methyl-6-phenyl-3-(1-phenylvinyl)hex-3-enoate (3ac)

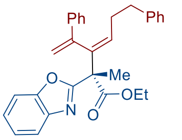

Reaction time: 3 h. **^{1}H NMR (400 MHz, $CDCl_3$)** δ 7.73 – 7.62 (m, 1H), 7.54 – 7.44 (m, 1H), 7.35 – 7.29 (m, 2H), 7.28 – 7.25 (m, 2H), 7.22 – 7.11 (m, 6H), 7.10 – 7.03 (m, 2H), 5.87 (t, *J* = 7.3 Hz, 1H), 5.59 (d, *J* = 1.0 Hz, 1H), 4.91 (d, *J* = 1.1 Hz, 1H), 4.13 (q, *J* = 7.1 Hz, 2H), 2.69 (t, *J* = 7.5 Hz, 2H), 2.48 – 2.35 (m, 2H), 1.75 (s, 3H), 1.18 (t, *J* = 7.1 Hz, 3H). **^{13}C NMR (100 MHz, $CDCl_3$)** δ 171.78, 166.25, 150.97, 144.62, 141.58, 141.00, 139.27, 138.48, 133.29, 128.74, 128.33, 128.28, 127.67, 126.24, 125.96, 124.96, 124.18, 120.24, 117.00, 110.70, 61.92, 55.18, 35.81, 32.07, 22.79, 14.06. **ESI-MS:** calculated $[C_{30}H_{29}NO_3 + H]^+$: 452.2220, found: 452.2228. $[\alpha]^{20}_D$= +12.69 (c = 0.81, CH_2Cl_2). The product was analyzed by HPLC to determine the enantiomeric excess: 88% e.e. (CHIRALPAK IC, hexane/*i*-PrOH =95/5, detector: 254 nm, T = 25 °C, flow rate: 1 mL/min), t_1(major) = 7.02 min, t_2(minor) = 16.20 min.

Isopropyl (*S*,*E*)-2-(benzo[d]oxazol-2-yl)-2-methyl-6-phenyl-3-(1-phenylvinyl)hex-3-enoate (3ad)

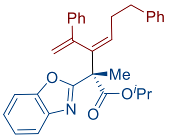

Reaction time: 3 h. **^{1}H NMR (400 MHz, $CDCl_3$)** δ 7.73 – 7.62 (m, 1H), 7.54 – 7.44 (m, 1H), 7.36 – 7.28 (m, 4H), 7.23 – 7.11 (m, 6H), 7.11 – 7.04 (m, 2H), 5.92 (t, *J* = 7.3 Hz, 1H), 5.60 (d, *J* = 1.2 Hz, 1H), 5.13 – 4.99 (m, 1H), 4.92 (d, *J* = 0.8 Hz, 1H), 2.69 (t, *J* = 7.5 Hz, 2H), 2.50 – 2.34 (m, 2H), 1.74 (s, 3H), 1.24 – 1.14 (m, 6H). **^{13}C NMR (100 MHz, $CDCl_3$)** δ 171.38, 166.39, 150.98, 144.63, 141.60, 141.03, 139.34, 138.41, 133.37, 128.70, 128.33, 128.28, 127.66, 126.23, 125.94, 124.90, 124.13, 120.20, 116.76, 110.65, 69.61, 55.16, 35.80, 32.09, 22.65, 21.65, 21.57. **ESI-MS:** calculated $[C_{31}H_{31}NO_3 + H]^+$: 466.2377, found: 466.2393. $[\alpha]^{20}_D$= +15.26 (c = 0.87, CH_2Cl_2). The product was analyzed by HPLC to determine the enantiomeric excess: 89% e.e. (CHIRALPAK IC, hexane/*i*-PrOH =95/5, detector: 235 nm, T = 25 °C, flow rate: 1 mL/min), t_1(major) = 5.88 min, t_2(minor) = 13.21 min.

2-phenylpropan-2-yl (*S*,*E*)-2-(benzo[d]oxazol-2-yl)-2-methyl-6-phenyl-3-(1-phenylvinyl)hex-3-enoate (3ae)

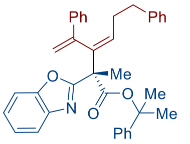

Reaction time: 3 h. **^{1}H NMR (400 MHz, $CDCl_3$)** δ 7.71 – 7.61 (m, 1H), 7.53 – 7.45 (m, 1H), 7.34 – 7.26 (m, 7H), 7.26 – 7.21 (m, 2H), 7.19 – 7.10 (m, 6H), 7.10 – 7.04 (m, 2H), 5.94 (t, *J* = 7.3 Hz, 1H), 5.57 (d, *J* = 1.0 Hz, 1H), 4.83 (d, *J* = 0.9 Hz, 1H), 2.68 (t, *J* = 7.5 Hz, 2H), 2.48 – 2.33 (m, 2H), 1.76 (s, 3H), 1.74 – 1.67 (m, 6H). **^{13}C NMR (100 MHz, $CDCl_3$)** δ 170.27, 166.41, 151.02, 145.35, 144.53, 141.60, 141.07, 139.35, 138.37, 133.56, 128.70, 128.35, 128.32, 128.31, 127.69, 127.31, 126.21, 125.95, 124.91, 124.47, 124.14, 120.18,

116.52, 110.62, 83.62, 55.68, 35.79, 32.08, 28.19, 28.02, 22.63. **ESI-MS:** calculated [$C_{37}H_{35}NO_3$ + H]$^+$: 542.2690, found: 542.2703. $[\alpha]^{20}_D$= +7.14 (c = 1.12, CH_2Cl_2). The product was analyzed by HPLC to determine the enantiomeric excess: 98% e.e. (CHIRALPAK IC, hexane/*i*-PrOH =95/5, detector: 254 nm, T = 25 °C, flow rate: 1 mL/min), t_1(major) = 5.44 min, t_2(minor) = 7.04 min.

Ethyl (*S*,*E*)-2-(4,5-dihydrooxazol-2-yl)-2-methyl-6-phenyl-3-(1-phenylvinyl)hex-3-enoate (3af)

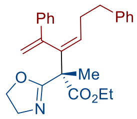

Reaction time: 4 h. **^{1}H NMR (400 MHz, CDCl3)** δ 7.37 – 7.31 (m, 2H), 7.28 – 7.21 (m, 5H), 7.20 – 7.14 (m, 1H), 7.13 – 7.07 (m, 2H), 5.84 (t, *J* = 7.3 Hz, 1H), 5.62 (d, *J* = 1.5 Hz, 1H), 5.02 (d, *J* = 1.5 Hz, 1H), 4.20 – 4.06 (m, 3H), 4.06 – 3.94 (m, 1H), 3.80 – 3.68 (m, 1H), 3.66 – 3.53 (m, 1H), 2.75 – 2.62 (m, 2H), 2.45 – 2.34 (m, 2H), 1.48 (s, 3H), 1.21 (t, *J* = 7.1 Hz, 3H). **^{13}C NMR (100 MHz, CDCl3)** δ 172.09, 168.08, 144.95, 141.78, 139.68, 137.82, 132.52, 128.75, 128.32, 128.27, 127.67, 126.41, 125.93, 116.74, 67.72, 61.56, 54.37, 54.24, 35.88, 31.97, 22.40, 14.08. **ESI-MS:** calculated [$C_{26}H_{29}NO_3$ + Na]$^+$: 426.2040, found: 426.2054. $[\alpha]^{20}_D$= +8.97 (c = 1.00, CH_2Cl_2). The product was analyzed by HPLC to determine the enantiomeric excess: 28% e.e. (CHIRALPAK IC, hexane/*i*-PrOH =80/20, detector: 254 nm, T = 25 °C, flow rate: 1 mL/min), t_1(major) = 8.38 min, t_2(minor) = 10.55 min.

Tert-butyl (Z)-2-(benzo[d]oxazol-2-yl)-6-phenyl-3-(1-phenylvinyl)hex-3-enoate (3ag) (Z/E >20:1)

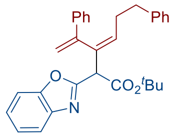

Reaction time: 3 h. **^{1}H NMR (400 MHz, CDCl3)** δ 7.76 – 7.69 (m, 1H), 7.56 – 7.48 (m, 1H), 7.37 – 7.30 (m, 4H), 7.29 – 7.24 (m, 3H), 7.20 – 7.11 (m, 3H), 7.08 – 7.01 (m, 2H), 5.94 (t, *J* = 7.2 Hz, 1H), 5.56 (d, *J* = 1.2 Hz, 1H), 5.03 (d, *J* = 1.1 Hz, 1H), 4.62 (s, 1H), 2.75 – 2.61 (m, 2H), 2.55 – 2.40 (m, 2H), 1.43 (s, 9H). **^{13}C NMR (100 MHz, CDCl3)** δ 167.50, 162.39, 151.14, 145.62, 141.52, 141.34, 138.28, 134.57, 133.61, 128.68, 128.57, 128.36, 128.06, 126.78, 125.94, 124.99, 124.30, 120.36, 116.42, 110.75, 82.50, 52.86, 35.82, 31.50, 27.99. **ESI-MS:** calculated [$C_{31}H_{31}NO_3$ + H]$^+$: 466.2377, found: 466.2375. The product was analyzed by HPLC to determine the enantiomeric excess: racemic (CHIRALPAK IE, hexane/*i*-PrOH =95/5, detector: 254 nm, T = 25 °C, flow rate: 1 mL/min), t_1 = 8.19 min, t_2 = 11.80 min.

Tert-butyl (*S*)-2-(benzo[d]oxazol-2-yl)-2-methyl-4-phenylpent-4-enoate (8a)

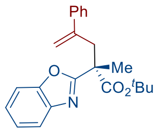

Reaction time: 3 h. **^{1}H NMR (400 MHz, CDCl3)** δ 7.58 – 7.53 (m, 1H), 7.37 – 7.31 (m, 1H), 7.25 – 7.21 (m, 2H), 7.20 – 7.15 (m, 2H), 7.05 – 6.98 (m, 2H), 6.96 – 6.90 (m, 1H), 5.22 (d, *J* = 1.6 Hz, 1H), 5.08 (d, *J* = 1.6 Hz, 1H), 3.55 (d, *J* = 13.8 Hz, 1H), 3.29 (d, *J* = 13.9 Hz, 1H), 1.60 (s, 3H), 1.33 (s, 9H). **^{13}C NMR (100 MHz, CDCl3)** δ 171.39, 166.69, 150.57, 144.57, 141.47, 140.75, 127.84, 126.98, 126.56, 124.69, 124.01, 119.92, 118.50, 110.43, 82.24, 49.51, 41.96, 27.85, 21.13. **ESI-MS:** calculated [$C_{23}H_{25}NO_3$ + H]$^+$: 364.1907, found: 364.1913. $[\alpha]^{20}_D$= +64.33 (c = 0.99, CH_2Cl_2). The product was analyzed by HPLC to determine the enantiomeric excess: 94% e.e. (CHIRALPAK IC, hexane/*i*-PrOH =99/1, detector: 254 nm, T = 25 °C, flow rate: 1 mL/min), t_1(major) = 6.22 min, t_2(minor) = 7.71 min.

Tert-butyl (*S*)-2-(benzo[d]oxazol-2-yl)-4-(4-fluorophenyl)-2-methylpent-4-enoate (8b)

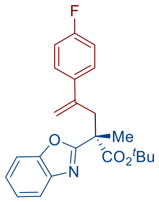

Reaction time: 3 h. **^{1}H NMR (500 MHz, CDCl3)** δ 7.58 – 7.53 (m, 1H), 7.35 – 7.30 (m, 1H), 7.26 – 7.22 (m, 2H), 7.13 – 7.08 (m, 2H), 6.68 – 6.63 (m, 2H), 5.17 (d, *J* = 1.5 Hz, 1H), 5.08 (d, *J* = 1.6 Hz, 1H), 3.52 (d, *J* = 13.9 Hz, 1H), 3.22 (d, *J* = 14.0 Hz, 1H), 1.62 (s, 3H), 1.34 (s, 9H). **^{13}C NMR (100 MHz, CDCl3)** δ 171.38, 166.47, 162.00 (d, *J* = 246.1 Hz), 150.54, 143.63, 140.71, 137.47, 137.43, 128.20 (d, *J* = 8.0 Hz), 124.83,

124.15, 119.93, 118.62, 114.59 (d, J = 21.4 Hz), 110.38, 82.33, 49.33, 42.31, 27.88, 21.09. ^{19}**F NMR (376 MHz, CDCl$_3$)** δ -115.80. **ESI-MS:** calculated [$C_{23}H_{24}FNO_3$ + Na]$^+$: 404.1632, found: 404.1645. $[\alpha]^{20}_D$= +89.61 (c = 0.83, CH_2Cl_2). The product was analyzed by HPLC to determine the enantiomeric excess: 94% e.e. (CHIRALPAK IG, hexane/*i*-PrOH =95/5, detector: 254 nm, T = 25 °C, flow rate: 1 mL/min), t_1(major) = 4.73 min, t_2(minor) = 5.38 min.

Tert-butyl (*S*)-2-(benzo[d]oxazol-2-yl)-4-(4-chlorophenyl)-2-methylpent-4-enoate (8c)

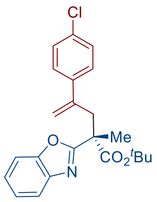

Reaction time: 6 h. ^{1}**H NMR (400 MHz, CDCl$_3$)** δ 7.58 – 7.52 (m, 1H), 7.35 – 7.30 (m, 1H), 7.26 – 7.23 (m, 2H), 7.08 – 7.03 (m, 2H), 6.95 – 6.89 (m, 2H), 5.19 (d, J = 1.3 Hz, 1H), 5.11 (d, J = 1.4 Hz, 1H), 3.52 (d, J = 13.9 Hz, 1H), 3.20 (d, J = 13.9 Hz, 1H), 1.62 (s, 3H), 1.34 (s, 9H). ^{13}**C NMR (100 MHz, CDCl$_3$)** δ 171.36, 166.29, 150.44, 143.55, 140.59, 139.79, 132.87, 127.90, 127.87, 124.92, 124.20, 119.92, 119.06, 110.40, 82.37, 49.26, 42.19, 27.87, 21.05. **ESI-MS:** calculated [$C_{23}H_{24}ClNO_3$ + Na]$^+$: 420.1337, found: 420.1346. $[\alpha]^{20}_D$= +100.81 (c = 0.77, CH_2Cl_2). The product was analyzed by HPLC to determine the enantiomeric excess: 94% e.e. (CHIRALPAK IG, hexane/*i*-PrOH =95/5, detector: 254 nm, T = 25 °C, flow rate: 1 mL/min), t_1(major) = 4.71 min, t_2(minor) = 5.20 min.

Tert-butyl (*S*)-2-(benzo[d]oxazol-2-yl)-2-methyl-4-(p-tolyl)pent-4-enoate (8d)

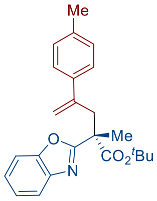

Reaction time: 3 h. ^{1}**H NMR (400 MHz, CDCl$_3$)** δ 7.58 – 7.53 (m, 1H), 7.37 – 7.31 (m, 1H), 7.25 – 7.20 (m, 2H), 7.08 – 7.02 (m, 2H), 6.82 – 6.76 (m, 2H), 5.18 (d, J = 1.6 Hz, 1H), 5.04 (d, J = 1.6 Hz, 1H), 3.53 (d, J = 13.8 Hz, 1H), 3.24 (d, J = 13.9 Hz, 1H), 2.08 (s, 3H), 1.61 (s, 3H), 1.34 (s, 9H). ^{13}**C NMR (100 MHz, CDCl$_3$)** δ 171.48, 166.67, 150.58, 144.46, 140.79, 138.55, 136.78, 128.49, 126.43, 124.59, 123.93, 119.90, 117.66, 110.45, 82.17, 49.47, 42.05, 27.86, 21.08, 20.96. **ESI-MS:** calculated [$C_{24}H_{27}NO_3$ + Na]$^+$: 400.1883, found: 400.1894. $[\alpha]^{20}_D$= +108.15 (c = 0.81, CH_2Cl_2). The product was analyzed by HPLC to determine the enantiomeric excess: 94% e.e. (CHIRALPAK IG, hexane/*i*-PrOH =95/5, detector: 254 nm, T = 25 °C, flow rate: 1 mL/min), t_1(major) = 4.69 min, t_2(minor) = 5.74 min.

Tert-butyl (*S*)-2-(benzo[d]oxazol-2-yl)-4-(4-methoxyphenyl)-2-methylpent-4-enoate (8e)

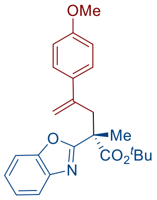

Reaction time: 3 h. ^{1}**H NMR (500 MHz, CDCl$_3$)** δ 7.58 – 7.52 (m, 1H), 7.37 – 7.31 (m, 1H), 7.26 – 7.21 (m, 2H), 7.10 – 7.06 (m, 2H), 6.53 – 6.47 (m, 2H), 5.15 (d, J = 1.6 Hz, 1H), 5.01 (d, J = 1.6 Hz, 1H), 3.59 (s, 3H), 3.53 (d, J = 13.8 Hz, 1H), 3.22 (d, J = 13.9 Hz, 1H), 1.62 (s, 3H), 1.35 (s, 9H). ^{13}**C NMR (150 MHz, CDCl$_3$)** δ 171.52, 166.67, 158.66, 150.60, 143.97, 140.82, 133.86, 127.67, 124.58, 123.93, 119.90, 117.15, 113.13, 110.43, 82.21, 55.18, 49.39, 42.18, 27.88, 21.06. **ESI-MS:** calculated [$C_{24}H_{27}NO_4$+ Na]$^+$: 416.1832, found: 416.1835. $[\alpha]^{20}_D$= +103.93 (c = 0.64, CH_2Cl_2). The product was analyzed by HPLC to determine the enantiomeric excess: 92% e.e. (CHIRALPAK IG, hexane/*i*-PrOH =95/5, detector: 254 nm, T = 25 °C, flow rate: 1 mL/min), t_1(major) = 6.79 min, t_2(minor) = 8.75 min.

Tert-butyl (*S*)-4-([1,1'-biphenyl]-4-yl)-2-(benzo[d]oxazol-2-yl)-2-methylpent-4-enoate (8f)

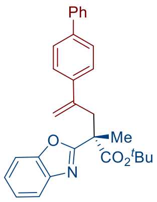

Reaction time: 3 h. **^{1}H NMR (400 MHz, $CDCl_3$)** δ 7.56 – 7.52 (m, 1H), 7.40 – 7.30 (m, 6H), 7.24 – 7.15 (m, 6H), 5.29 (d, *J* = 1.5 Hz, 1H), 5.14 (d, *J* = 1.5 Hz, 1H), 3.62 (d, *J* = 13.9 Hz, 1H), 3.28 (d, *J* = 13.9 Hz, 1H), 1.68 (s, 3H), 1.35 (s, 9H). **^{13}C NMR (100 MHz, $CDCl_3$)** δ 171.48, 166.53, 150.55, 144.19, 140.82, 140.75, 140.29, 140.00, 128.63, 127.19, 127.15, 126.94, 126.59, 124.76, 124.05, 119.90, 118.53, 110.39, 82.27, 49.34, 42.13, 27.87, 21.11. **ESI-MS:** calculated $[C_{29}H_{29}NO_3 + Na]^+$: 462.2040, found: 462.2044. $[\alpha]^{20}_{D}$= +101.36 (c = 0.91, CH_2Cl_2). The product was analyzed by HPLC to determine the enantiomeric excess: 95% e.e. (CHIRALPAK IC, hexane/*i*-PrOH =99/1, detector: 292 nm, T = 25 °C, flow rate: 1 mL/min), t_1(major) = 8.59 min, t_2(minor) = 11.12 min.

Tert-butyl (*S*)-2-(benzo[d]oxazol-2-yl)-4-(3-chlorophenyl)-2-methylpent-4-enoate (8g)

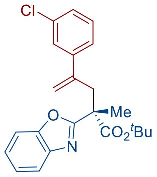

Reaction time: 6 h. **^{1}H NMR (500 MHz, $CDCl_3$)** δ 7.57 – 7.52 (m, 1H), 7.35 – 7.31 (m, 1H), 7.25 – 7.20 (m, 2H), 7.15 – 7.12 (m, 1H), 7.03 – 6.99 (m, 1H), 6.90 – 6.86 (m, 1H), 6.85 – 6.81 (m, 1H), 5.23 (d, *J* = 1.3 Hz, 1H), 5.14 (d, *J* = 1.2 Hz, 1H), 3.53 (d, *J* = 13.9 Hz, 1H), 3.21 (d, *J* = 14.0 Hz, 1H), 1.64 (s, 3H), 1.34 (s, 9H). **^{13}C NMR (125 MHz, $CDCl_3$)** δ 171.32, 166.24, 150.50, 143.44, 143.05, 140.65, 133.71, 128.98, 126.84, 126.77, 124.84, 124.81, 124.11, 119.91, 119.67, 110.46, 82.38, 49.20, 42.13, 27.86, 21.07. **ESI-MS:** calculated $[C_{23}H_{24}ClNO_3 + Na]^+$: 420.1337, found: 420.1341. $[\alpha]^{20}_{D}$= +70.85 (c = 0.89, CH_2Cl_2). The product was analyzed by HPLC to determine the enantiomeric excess: 91% e.e. (CHIRALPAK IG, hexane/*i*-PrOH =95/5, detector: 254 nm, T = 25 °C, flow rate: 1 mL/min), t_1(major) = 4.76 min, t_2(minor) = 5.71 min.

Tert-butyl (*S*)-2-(benzo[d]oxazol-2-yl)-2-methyl-4-(m-tolyl)pent-4-enoate (8h)

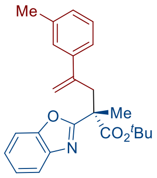

Reaction time: 6 h. **^{1}H NMR (400 MHz, $CDCl_3$)** δ 7.59 – 7.52 (m, 1H), 7.36 – 7.29 (m, 1H), 7.25 – 7.19 (m, 2H), 7.02 – 6.96 (m, 1H), 6.95 – 6.88 (m, 2H), 6.75 – 6.68 (m, 1H), 5.20 (d, *J* = 1.6 Hz, 1H), 5.07 (d, *J* = 1.7 Hz, 1H), 3.55 (d, *J* = 13.8 Hz, 1H), 3.25 (d, *J* = 13.9 Hz, 1H), 2.08 (s, 3H), 1.62 (s, 3H), 1.34 (s, 9H). **^{13}C NMR (100 MHz, $CDCl_3$)** δ 171.49, 166.67, 150.60, 144.71, 141.37, 140.82, 137.34, 127.77, 127.66, 127.23, 124.64, 123.99, 123.79, 119.89, 118.26, 110.37, 82.20, 49.41, 42.10, 27.87, 21.33, 21.07. **ESI-MS:** calculated $[C_{24}H_{27}NO_3 + Na]^+$: 400.1883, found: 400.1888. $[\alpha]^{20}_{D}$= +98.77 (c = 0.42, CH_2Cl_2). The product was analyzed by HPLC to determine the enantiomeric excess: 94% e.e. (CHIRALPAK IG, hexane/*i*-PrOH =95/5, detector: 254 nm, T = 25 °C, flow rate: 1 mL/min), t_1(major) = 4.66 min, t_2(minor) = 5.51 min.

Tert-butyl (*S*)-2-(benzo[d]oxazol-2-yl)-4-(3-methoxyphenyl)-2-methylpent-4-enoate (8i)

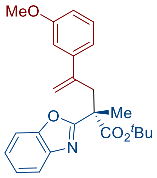

Reaction time: 3 h. **^{1}H NMR (400 MHz, $CDCl_3$)** δ 7.59 – 7.53 (m, 1H), 7.37 – 7.31 (m, 1H), 7.25 – 7.19 (m, 2H), 6.97 – 6.90 (m, 1H), 6.81 – 6.75 (m, 1H), 6.71 – 6.66 (m, 1H), 6.50 – 6.43 (m, 1H), 5.23 (d, *J* = 1.6 Hz, 1H), 5.08 (d, *J* = 1.7 Hz, 1H), 3.65 (s, 3H), 3.53 (d, *J* = 13.8 Hz, 1H), 3.26 (d, *J* = 13.9 Hz, 1H), 1.62 (s, 3H), 1.34 (s, 9H). **^{13}C NMR (100 MHz, $CDCl_3$)** δ 171.41, 166.69, 159.11, 150.64, 144.50, 142.96, 140.83, 128.84, 124.66, 123.99, 119.91, 119.24, 118.51, 112.51, 112.24, 110.43, 82.23, 55.14, 49.52, 42.11, 27.88, 21.12. **ESI-MS:** calculated $[C_{24}H_{27}NO_4 + Na]^+$: 416.1832, found: 416.1840. $[\alpha]^{20}_{D}$= +95.83 (c = 0.96, CH_2Cl_2). The product was analyzed by HPLC to determine the enantiomeric excess: 91% e.e. (CHIRALPAK IG, hexane/*i*-PrOH =95/5, detector: 254 nm, T = 25 °C, flow rate: 1 mL/min), t_1(major) = 6.14 min, t_2(minor)

= 7.13 min.

Tert-butyl (*S*)-2-(benzo[d]oxazol-2-yl)-4-(2-methoxyphenyl)-2-methylpent-4-enoate (8j)

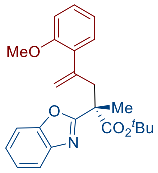

Reaction time: 3 h. **^{1}H NMR (500 MHz, CDCl$_3$)** δ 7.58 – 7.52 (m, 1H), 7.30 – 7.26 (m, 1H), 7.23 – 7.18 (m, 2H), 6.89 – 6.86 (m, 1H), 6.86 – 6.80 (m, 1H), 6.57 – 6.49 (m, 2H), 5.20 – 5.17 (m, 1H), 5.07 (d, *J* = 2.1 Hz, 1H), 3.79 (s, 3H), 3.71 (d, *J* = 13.4 Hz, 1H), 3.23 (d, *J* = 13.9 Hz, 1H), 1.65 (s, 3H), 1.32 (s, 9H). **^{13}C NMR (125 MHz, CDCl$_3$)** δ 171.64, 166.86, 156.10, 150.61, 143.98, 140.89, 130.69, 130.31, 128.13, 124.47, 123.85, 120.24, 120.14, 119.90, 110.34, 109.96, 81.96, 55.41, 49.36, 42.50, 27.85, 20.98. **ESI-MS:** calculated [$C_{24}H_{27}NO_4$ + H]$^+$: 416.1832, found: 416.1828. $[\alpha]^{20}_D$= +53.28 (c = 1.03, CH_2Cl_2). The product was analyzed by HPLC to determine the enantiomeric excess: 92% e.e. (CHIRALPAK IG, hexane/*i*-PrOH =95/5, detector: 254 nm, T = 25 °C, flow rate: 1 mL/min), t_1(major) = 5.29 min, t_2(minor) = 6.39 min.

Tert-butyl (*S*)-2-(benzo[d]oxazol-2-yl)-4-(furan-2-yl)-2-methylpent-4-enoate (8k)

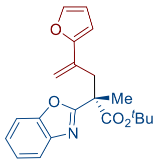

Reaction time: 3 h. **^{1}H NMR (600 MHz, CDCl$_3$)** δ 7.70 – 7.66 (m, 1H), 7.51 – 7.47 (m, 1H), 7.33 – 7.28 (m, 2H), 7.16 (d, *J* = 1.4 Hz, 1H), 6.21 (d, *J* = 3.3 Hz, 1H), 6.17 (dd, *J* = 3.4, 1.9 Hz, 1H), 5.55 (d, *J* = 0.6 Hz, 1H), 4.84 (d, *J* = 0.6 Hz, 1H), 3.35 (d, *J* = 14.5 Hz, 1H), 3.23 (d, *J* = 14.5 Hz, 1H), 1.67 (s, 3H), 1.38 (s, 9H). **^{13}C NMR (150 MHz, CDCl$_3$)** δ 171.34, 167.07, 154.65, 150.84, 141.96, 140.96, 132.65, 124.97, 124.27, 120.15, 114.15, 111.10, 110.51, 106.66, 82.42, 50.07, 38.86, 27.93, 21.17. **ESI-MS:** calculated [$C_{21}H_{23}NO_4$ + Na]$^+$: 376.1519, found: 376.1530. $[\alpha]^{20}_D$= +77.35 (c = 0.67, CH_2Cl_2). The product was analyzed by HPLC to determine the enantiomeric excess: 93% e.e. (CHIRALPAK IG, hexane/*i*-PrOH =95/5, detector: 254 nm, T = 25 °C, flow rate: 0.7 mL/min), t_1(major) = 9.01 min, t_2(minor) = 9.73 min.

Tert-butyl (*S*)-2-(benzo[d]oxazol-2-yl)-2-methyl-4-(naphthalen-2-yl)pent-4-enoate (8l)

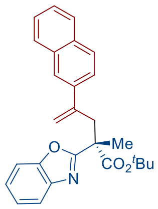

Reaction time: 3 h. **^{1}H NMR (400 MHz, CDCl$_3$)** δ 7.65 – 7.55 (m, 3H), 7.54 – 7.49 (m, 1H), 7.46 – 7.41 (m, 1H), 7.38 – 7.28 (m, 3H), 7.20 – 7.14 (m, 1H), 7.10 – 6.99 (m, 2H), 5.35 (d, *J* = 1.5 Hz, 1H), 5.18 (d, *J* = 1.4 Hz, 1H), 3.67 (d, *J* = 13.9 Hz, 1H), 3.38 (d, *J* = 13.9 Hz, 1H), 1.64 (s, 3H), 1.32 (s, 9H). **^{13}C NMR (100 MHz, CDCl$_3$)** δ 171.48, 166.59, 150.40, 144.52, 140.58, 138.85, 132.99, 132.46, 128.03, 127.53, 127.37, 125.91, 125.62, 125.24, 125.10, 124.64, 123.94, 119.70, 118.96, 110.21, 82.28, 49.59, 42.09, 27.87, 21.18. **ESI-MS:** calculated [$C_{27}H_{27}NO_3$ + Na]$^+$: 436.1883, found: 436.1891. $[\alpha]^{20}_D$= +78.81 (c = 0.38, CH_2Cl_2). The product was analyzed by HPLC to determine the enantiomeric excess: 95% e.e. (CHIRALPAK IC, hexane/*i*-PrOH =99/1, detector: 247 nm, T = 25 °C, flow rate: 1 mL/min), t_1(major) = 7.86 min, t_2(minor) = 10.54 min.

Tert-butyl (*S*,*E*)-2-(benzo[d]oxazol-2-yl)-2-methyl-4-methylene-6-phenylhex-5-enoate (8m)

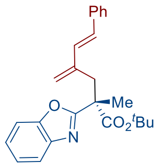

Reaction time: 3 h. **^{1}H NMR (600 MHz, CDCl$_3$)** δ 7.70 – 7.66 (m, 1H), 7.50 – 7.46 (m, 1H), 7.29 – 7.26 (m, 2H), 7.22 – 7.19 (m, 2H), 7.17 – 7.13 (m, 3H), 6.63 (d, *J* = 16.3 Hz, 1H), 6.46 (d, *J* = 16.3 Hz, 1H), 5.24 (d, *J* = 0.8 Hz, 1H), 4.91 (d, *J* = 0.7 Hz, 1H), 3.31 (d, *J* = 14.3 Hz, 1H), 3.20 (d, *J* = 14.3 Hz, 1H), 1.73 (s, 3H), 1.40 (s, 9H). **^{13}C NMR (150 MHz, CDCl$_3$)** δ 171.56, 167.20, 150.81, 141.20, 140.98, 137.06, 131.20, 128.82, 128.51, 127.48, 126.41, 125.01, 124.32, 120.15, 119.86, 110.55, 82.39, 49.85, 37.94, 27.95, 21.36. **ESI-MS:** calculated [$C_{25}H_{27}NO_3$ + Na]$^+$: 412.1883, found: 412.1888. $[\alpha]^{20}_D$= +70.75 (c = 0.84,

CH_2Cl_2). The product was analyzed by HPLC to determine the enantiomeric excess: 96% e.e. (CHIRALPAK IG, hexane/*i*-PrOH =95/5, detector: 254 nm, T = 25 °C, flow rate: 1 mL/min), t_1(major) = 5.36 min, t_2(minor) = 6.50 min.

Tert-butyl (*S*)-2-(benzo[d]oxazol-2-yl)-2-methylpent-4-enoate (8n)

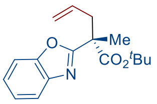

Reaction time: 6 h. **^{1}H NMR (400 MHz, $CDCl_3$)** δ 7.76 – 7.69 (m, 1H), 7.54 – 7.47 (m, 1H), 7.36 – 7.28 (m, 2H), 5.80 – 5.65 (m, 1H), 5.14 (dd, *J* = 16.9, 1.1 Hz, 1H), 5.08 (dd, *J* = 10.2, 1.3 Hz, 1H), 3.01 – 2.82 (m, 2H), 1.67 (s, 3H), 1.39 (s, 9H). **^{13}C NMR (100 MHz, $CDCl_3$)** δ 171.20, 167.30, 150.98, 141.02, 132.63, 124.99, 124.32, 120.18, 119.49, 110.62, 82.23, 49.45, 41.33, 27.97, 21.12. **ESI-MS:** calculated $[C_{17}H_{21}NO_3 + Na]^+$: 310.1414, found: 310.1419. $[\alpha]^{20}_D$= +13.05 (c = 0.60, CH_2Cl_2). The product was analyzed by HPLC to determine the enantiomeric excess: 87% e.e. (CHIRALPAK IC, hexane/*i*-PrOH =99/1, detector: 254 nm, T = 25 °C, flow rate: 0.5 mL/min), t_1(major) = 10.05 min, t_2(minor) = 11.92 min.

Tert-butyl (*R*)-2-(benzo[d]oxazol-2-yl)-2-fluoro-4-phenylpent-4-enoate (8o)

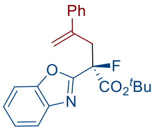

Reaction time: 3 h. **^{1}H NMR (400 MHz, $CDCl_3$)** δ 7.81 – 7.75 (m, 1H), 7.57 – 7.51 (m, 1H), 7.44 – 7.35 (m, 4H), 7.31 – 7.26 (m, 2H), 7.25 – 7.19 (m, 1H), 5.50 (d, *J* = 0.7 Hz, 1H), 5.41 (d, *J* = 0.7 Hz, 1H), 3.81 – 3.79 (m, 1H), 3.79 – 3.68 (m, 1H), 1.33 (s, 9H). **^{13}C NMR (100 MHz, $CDCl_3$)** δ 164.90 (d, *J* = 27.4 Hz), 160.81 (d, *J* = 25.4 Hz), 150.87, 141.24, 141.10, 140.44, 128.32, 127.74, 126.68, 126.28, 124.99, 121.02, 119.02, 111.23, 91.42 (d, *J* = 194.1 Hz), 84.54, 39.91 (d, *J* = 20.8 Hz), 27.75. **^{19}F NMR (376 MHz, $CDCl_3$)** δ -156.80. **ESI-MS:** calculated $[C_{22}H_{22}FNO_3 + Na]^+$: 390.1476, found: 390.1483. $[\alpha]^{20}_D$= -1.33 (c = 0.86, CH_2Cl_2). The product was analyzed by HPLC to determine the enantiomeric excess: 91% e.e. (CHIRALPAK IG, hexane/*i*-PrOH =90/10, detector: 254 nm, T = 25 °C, flow rate: 1 mL/min), t_1(minor) = 5.94 min, t_2(major) = 7.57 min.

Tert-butyl (*S*)-2-(benzo[d]oxazol-2-yl)-2-ethyl-4-phenylpent-4-enoate (8p)

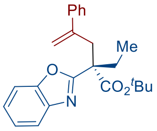

Reaction time: 3 h. **^{1}H NMR (500 MHz, $CDCl_3$)** δ 7.54 – 7.50 (m, 1H), 7.33 – 7.29 (m, 1H), 7.23 – 7.18 (m, 2H), 7.15 – 7.10 (m, 2H), 6.97 – 6.92 (m, 2H), 6.89 – 6.84 (m, 1H), 5.17 (d, *J* = 1.6 Hz, 1H), 5.07 (d, *J* = 1.5 Hz, 1H), 3.50 (d, *J* = 14.2 Hz, 1H), 3.30 (d, *J* = 14.2 Hz, 1H), 2.27 – 2.18 (m, 1H), 2.18 – 2.09 (m, 1H), 1.34 (s, 9H), 0.88 (t, *J* = 7.5 Hz, 3H). **^{13}C NMR (100 MHz, $CDCl_3$)** δ 170.55, 166.22, 150.34, 144.82, 141.44, 140.68, 127.68, 126.81, 126.50, 124.59, 123.91, 119.90, 118.28, 110.37, 82.17, 53.64, 38.71, 27.94, 25.96, 8.66. **ESI-MS:** calculated $[C_{24}H_{27}NO_3 + Na]^+$: 400.1883, found: 400.1888. $[\alpha]^{20}_D$= +109.63 (c = 0.83, CH_2Cl_2). The product was analyzed by HPLC to determine the enantiomeric excess: 90% e.e. (CHIRALPAK IG, hexane/*i*-PrOH =95/5, detector: 254 nm, T = 25 °C, flow rate: 1 mL/min), t_1(major) = 4.64 min, t_2(minor) = 7.91 min.

Tert-butyl (*S*)-2-(benzo[d]oxazol-2-yl)-4-phenyl-2-propylpent-4-enoate (8q)

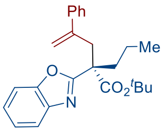

Reaction time: 3 h. **^{1}H NMR (500 MHz, $CDCl_3$)** δ 7.57 – 7.52 (m, 1H), 7.35 – 7.30 (m, 1H), 7.24 – 7.20 (m, 2H), 7.17 – 7.13 (m, 2H), 6.99 – 6.95 (m, 2H), 6.92 – 6.87 (m, 1H), 5.16 (d, *J* = 1.6 Hz, 1H), 5.03 (d, *J* = 1.6 Hz, 1H), 3.50 (d, *J* = 14.2 Hz, 1H), 3.32 (d, *J* = 14.2 Hz, 1H), 2.16 – 2.02 (m, 2H), 1.34 (s, 9H), 1.27 – 1.20 (m, 2H), 0.81 (t, *J* = 7.3 Hz, 3H). **^{13}C NMR (100 MHz, $CDCl_3$)** δ 170.58, 166.38, 150.36, 144.94, 141.56, 140.68, 127.73, 126.84, 126.52, 124.61, 123.94, 119.93, 118.22, 110.38, 82.14, 53.28, 38.77, 34.71, 27.92, 17.46, 14.28.

ESI-MS: calculated $[C_{25}H_{29}NO_3 + Na]^+$: 414.2040, found: 414.2047. $[\alpha]^{20}_D$= +92.95 (c = 0.90, CH_2Cl_2). The product was analyzed by HPLC to determine the enantiomeric excess: 90% e.e. (CHIRALPAK IG, hexane/*i*-PrOH =95/5, detector: 272 nm, T = 25 °C, flow rate: 1 mL/min), t_1(major) = 4.30 min, t_2(minor) = 8.52 min.

Tert-butyl (*S*)-2-(benzo[d]oxazol-2-yl)-2-(2-phenylallyl)hexanoate (8r)

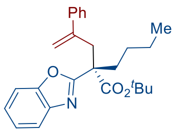

Reaction time: 3 h. **^{1}H NMR (400 MHz, CDCl₃)** δ 7.58 – 7.51 (m, 1H), 7.36 – 7.31 (m, 1H), 7.24 – 7.19 (m, 2H), 7.17 – 7.11 (m, 2H), 7.00 – 6.94 (m, 2H), 6.92 – 6.85 (m, 1H), 5.16 (d, *J* = 1.6 Hz, 1H), 5.03 (d, *J* = 1.6 Hz, 1H), 3.50 (d, *J* = 14.1 Hz, 1H), 3.31 (d, *J* = 14.2 Hz, 1H), 2.17 – 2.03 (m, 2H), 1.34 (s, 9H), 1.24 – 1.13 (m, 4H), 0.82 (t, *J* = 6.8 Hz, 3H). **^{13}C NMR (100 MHz, $CDCl_3$)** δ 170.60, 166.46, 150.40, 144.98, 141.65, 140.75, 127.74, 126.87, 126.57, 124.62, 123.95, 119.97, 118.22, 110.40, 82.12, 53.31, 38.69, 32.26, 27.94, 26.20, 22.89, 14.01. **ESI-MS:** calculated $[C_{26}H_{31}NO_3 + Na]^+$: 428.2196, found: 428.2192. $[\alpha]^{20}_D$= +86.24 (c = 0.84, CH_2Cl_2). The product was analyzed by HPLC to determine the enantiomeric excess: 90% e.e. (CHIRALPAK IG, hexane/*i*-PrOH =95/5, detector: 254 nm, T = 25 °C, flow rate: 1 mL/min), t_1(major) = 4.09 min, t_2(minor) = 5.85 min.

Tert-butyl (*S*)-2-(benzo[d]oxazol-2-yl)-2-phenethyl-4-phenylpent-4-enoate (8s)

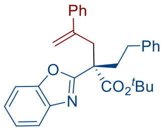

Reaction time: 3 h. **^{1}H NMR (500 MHz, $CDCl_3$)** δ 7.59 – 7.54 (m, 1H), 7.38 – 7.34 (m, 1H), 7.26 – 7.19 (m, 6H), 7.17 – 7.12 (m, 1H), 7.05 – 7.01 (m, 2H), 6.98 – 6.94 (m, 3H), 5.20 (d, *J* = 1.5 Hz, 1H), 5.09 (d, *J* = 1.4 Hz, 1H), 3.60 (d, *J* = 14.2 Hz, 1H), 3.45 (d, *J* = 14.3 Hz, 1H), 2.53 – 2.35 (m, 4H), 1.39 (s, 9H). **^{13}C NMR (150 MHz, $CDCl_3$)** δ 170.35, 166.01, 150.39, 144.84, 141.68, 141.37, 140.71, 128.47, 128.40, 127.93, 127.02, 126.65, 126.05, 124.74, 124.05, 120.01, 118.53, 110.45, 82.44, 53.43, 38.73, 34.75, 30.69, 28.00. **ESI-MS:** calculated $[C_{30}H_{31}NO_3 + Na]^+$: 476.2196, found: 476.2201. $[\alpha]^{20}_D$= +43.89 (c = 0.94, CH_2Cl_2). The product was analyzed by HPLC to determine the enantiomeric excess: 90% e.e. (CHIRALPAK IG, hexane/*i*-PrOH =99/1, detector: 273 nm, T = 25 °C, flow rate: 1 mL/min), t_1(major) = 7.23 min, t_2(minor) = 10.75 min.

Tert-butyl (*S*)-2-(benzo[d]oxazol-2-yl)-2-(3-((tert-butyldimethylsilyl)oxy)propyl)-4-phenylpent-4-enoate (8t)

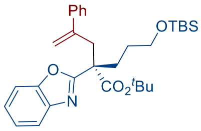

Reaction time: 3 h. **^{1}H NMR (400 MHz, $CDCl_3$)** δ 7.56 – 7.50 (m, 1H), 7.34 – 7.28 (m, 1H), 7.24 – 7.18 (m, 2H), 7.16 – 7.11 (m, 2H), 7.00 – 6.93 (m, 2H), 6.91 – 6.85 (m, 1H), 5.18 (d, *J* = 1.5 Hz, 1H), 5.09 (d, *J* = 1.6 Hz, 1H), 3.55 – 3.45 (m, 3H), 3.33 (d, *J* = 14.2 Hz, 1H), 2.21 – 2.08 (m, 2H), 1.51 – 1.42 (m, 2H), 1.33 (s, 9H), 0.87 (s, 9H), 0.03 – -0.01 (m, 6H). **^{13}C NMR (100 MHz, $CDCl_3$)** δ 170.45, 166.23, 150.40, 144.76, 141.56, 140.75, 127.76, 126.89, 126.55, 124.62, 123.94, 119.96, 118.44, 110.40, 82.22, 63.16, 52.98, 39.04, 29.25, 27.93, 27.72, 26.11, 18.47, -5.17. **ESI-MS:** calculated $[C_{31}H_{43}NO_4Si + Na]^+$: 544.2854, found: 544.2853. $[\alpha]^{20}_D$= +61.97 (c = 1.09, CH_2Cl_2). The product was analyzed by HPLC to determine the enantiomeric excess: 88% e.e. (CHIRALPAK IA, hexane/*i*-PrOH =95/5, detector: 254 nm, T = 25 °C, flow rate: 1 mL/min), t_1(major) = 3.42 min, t_2(minor) = 4.13 min.

Tert-butyl (*S*)-2-(benzo[d]oxazol-2-yl)-2-(3-methoxypropyl)-4-phenylpent-4-enoate (8u)

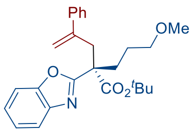

Reaction time: 3 h. **^{1}H NMR (500 MHz, $CDCl_3$)** δ 7.56 – 7.51 (m, 1H), 7.34 – 7.30 (m, 1H), 7.24 – 7.20 (m, 2H), 7.16 – 7.13 (m, 2H), 7.00 – 6.95 (m, 2H), 6.93 – 6.87 (m, 1H), 5.17 (d, J = 1.5 Hz, 1H), 5.07 (d, J = 1.5 Hz, 1H), 3.51 (d, J = 14.2 Hz, 1H), 3.32 (d, J = 14.2 Hz, 1H), 3.25 (s, 3H), 3.24 – 3.17 (m, 2H), 2.21 – 2.08 (m, 2H), 1.55 – 1.47 (m, 2H), 1.34 (s, 9H). **^{13}C NMR (100 MHz, $CDCl_3$)** δ 170.35, 166.11, 150.38, 144.71, 141.54, 140.66, 127.78, 126.89, 126.55, 124.68, 123.98, 119.95, 118.48, 110.42, 82.36, 72.58, 58.59, 53.00, 38.90, 29.26, 27.92, 24.36. **ESI-MS:** calculated $[C_{26}H_{31}NO_4 + Na]^+$: 444.2145, found: 444.2151. $[\alpha]^{20}_D$= +86.22 (c = 0.85, CH_2Cl_2). The product was analyzed by HPLC to determine the enantiomeric excess: 90% e.e. (CHIRALPAK IA, hexane/*i*-PrOH =95/5, detector: 254 nm, T = 25 °C, flow rate: 1 mL/min), t_1(major) = 4.78 min, t_2(minor) = 6.27 min.

Tert-butyl (*S*)-2-allyl-2-(benzo[d]oxazol-2-yl)-4-phenylpent-4-enoate (8v)

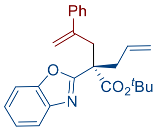

Reaction time: 3 h. **^{1}H NMR (500 MHz, $CDCl_3$)** δ 7.55 – 7.50 (m, 1H), 7.33 – 7.28 (m, 1H), 7.24 – 7.20 (m, 2H), 7.15 – 7.10 (m, 2H), 6.97 – 6.92 (m, 2H), 6.89 – 6.83 (m, 1H), 5.79 – 5.69 (m, 1H), 5.20 (d, J = 1.4 Hz, 1H), 5.11 (d, J = 1.2 Hz, 1H), 5.10 – 5.03 (m, 2H), 3.01 – 2.88 (m, 2H), 1.33 (s, 9H). **^{13}C NMR (100 MHz, $CDCl_3$)** δ 169.97, 165.69, 150.36, 144.62, 141.34, 140.60, 132.43, 127.68, 126.84, 126.58, 124.67, 123.97, 119.93, 119.55, 118.74, 110.40, 82.43, 52.93, 39.03, 37.36, 27.95. **ESI-MS:** calculated $[C_{25}H_{27}NO_3 + Na]^+$: 412.1883, found: 412.1888. $[\alpha]^{20}_D$= +96.15 (c = 0.88, CH_2Cl_2). The product was analyzed by HPLC to determine the enantiomeric excess: 85% e.e. (CHIRALPAK IG, hexane/*i*-PrOH =95/5, detector: 254 nm, T = 25 °C, flow rate: 1 mL/min), t_1(major) = 4.61 min, t_2(minor) = 6.34 min.

Ethyl (*R*)-2-(benzo[d]oxazol-2-yl)-2-isopropyl-4-phenylpent-4-enoate (8w)

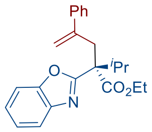

Reaction time: 5 h. **^{1}H NMR (400 MHz, $CDCl_3$)** δ 7.66 – 7.59 (m, 1H), 7.41 – 7.35 (m, 1H), 7.27 – 7.24 (m, 2H), 7.22 – 7.18 (m, 2H), 7.10 – 7.04 (m, 2H), 7.03 – 6.96 (m, 1H), 5.22 (d, J = 1.1 Hz, 1H), 5.16 (d, J = 1.2 Hz, 1H), 3.94 (q, J = 7.1 Hz, 2H), 3.51 (s, 2H), 2.75 – 2.62 (m, 1H), 1.14 (t, J = 7.1 Hz, 3H), 1.10 (d, J = 6.8 Hz, 3H), 0.97 (d, J = 6.8 Hz, 3H). **^{13}C NMR (100 MHz, $CDCl_3$)** δ 170.51, 165.19, 150.30, 144.62, 141.72, 140.57, 127.80, 127.01, 126.57, 124.71, 124.02, 120.03, 118.48, 110.56, 61.23, 57.46, 40.21, 32.77, 18.87, 18.70, 14.15. **ESI-MS:** calculated $[C_{23}H_{25}NO_3 + H]^+$: 364.1907, found: 364.1921. $[\alpha]^{20}_D$= +4.69 (c = 0.51, CH_2Cl_2). The product was analyzed by HPLC to determine the enantiomeric excess: 60% e.e. (CHIRALPAK IC, hexane/*i*-PrOH =95/5, detector: 254 nm, T = 25 °C, flow rate: 1 mL/min), t_1(major) = 5.48 min, t_2(minor) = 6.20 min.

Ethyl (*R*)-2-(benzo[d]oxazol-2-yl)-2-cyclohexyl-4-phenylpent-4-enoate (8x)

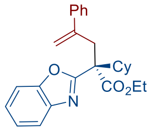

Reaction time: 5 h. **^{1}H NMR (400 MHz, $CDCl_3$)** δ 7.69 – 7.61 (m, 1H), 7.43 – 7.36 (m, 1H), 7.28 – 7.23 (m, 4H), 7.16 – 7.09 (m, 2H), 7.09 – 7.01 (m, 1H), 5.20 (d, J = 1.2 Hz, 1H), 5.09 (d, J = 0.8 Hz, 1H), 4.03 – 3.88 (m, 2H), 3.59 – 3.45 (m, 2H), 2.31 – 2.18 (m, 1H), 2.07 – 1.90 (m, 2H), 1.79 – 1.63 (m, 2H), 1.61 – 1.54 (m, 1H), 1.24 – 1.10 (m, 5H), 1.10 – 0.94 (m, 2H), 0.82 – 0.67 (m, 1H). **^{13}C NMR (100 MHz, $CDCl_3$)** δ 170.48, 165.38, 150.29, 144.77, 142.02, 140.63, 127.87, 127.06, 126.66, 124.69, 124.04, 120.07, 118.25, 110.60, 61.16, 57.65, 43.25, 39.78, 29.16, 28.70, 26.90, 26.67, 26.46, 14.16. **ESI-MS:** calculated $[C_{26}H_{29}NO_3 + H]^+$: 404.2220, found: 404.2229. $[\alpha]^{20}_D$= +3.75 (c = 0.58, CH_2Cl_2). The product was analyzed by HPLC to determine the enantiomeric excess: 61% e.e. (CHIRALPAK IC, hexane/*i*-PrOH =95/5, detector: 254 nm, T = 25 °C,

flow rate: 1 mL/min), t_1(major) = 5.04 min, t_2(minor) = 6.00 min.

Methyl (*S*)-2-(benzo[d]oxazol-2-yl)-2-methyl-4-phenylpent-4-enoate (8y)

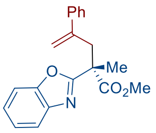

Reaction time: 3 h. **^{1}H NMR (500 MHz, CDCl$_3$)** δ 7.63 – 7.59 (m, 1H), 7.39 – 7.35 (m, 1H), 7.27 – 7.25 (m, 2H), 7.23 – 7.19 (m, 2H), 7.11 – 7.06 (m, 2H), 7.03 – 6.98 (m, 1H), 5.26 (d, *J* = 1.6 Hz, 1H), 5.12 (d, *J* = 1.4 Hz, 1H), 3.50 (s, 2H), 3.49 (s, 3H), 1.65 (s, 3H). **^{13}C NMR (150 MHz, CDCl$_3$)** δ 172.55, 166.34, 150.66, 144.30, 141.15, 140.71, 127.93, 127.19, 126.69, 124.98, 124.24, 120.06, 118.86, 110.65, 52.74, 48.63, 42.27, 21.18. **ESI-MS:** calculated [$C_{20}H_{19}NO_3$ + H]$^+$: 322.1438, found: 322.1445. $[\alpha]^{20}_D$= +30.05 (c = 0.54, CH_2Cl_2). The product was analyzed by HPLC to determine the enantiomeric excess: 91% e.e. (CHIRALPAK IC, hexane/*i*-PrOH =95/5, detector: 254 nm, T = 25 °C, flow rate: 1 mL/min), t_1(major) = 7.57 min, t_2(minor) = 10.83 min.

Ethyl (*S*)-2-(benzo[d]oxazol-2-yl)-2-methyl-4-phenylpent-4-enoate (8z)

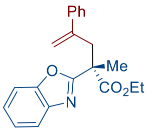

Reaction time: 3 h. **^{1}H NMR (500 MHz, CDCl$_3$)** δ 7.61 – 7.57 (m, 1H), 7.38 – 7.34 (m, 1H), 7.26 – 7.23 (m, 2H), 7.22 – 7.18 (m, 2H), 7.09 – 7.03 (m, 2H), 7.01 – 6.96 (m, 1H), 5.24 (d, *J* = 1.5 Hz, 1H), 5.11 (d, *J* = 1.4 Hz, 1H), 4.06 – 3.97 (m, 1H), 3.96 – 3.88 (m, 1H), 3.53 (d, *J* = 13.8 Hz, 1H), 3.44 (d, *J* = 13.9 Hz, 1H), 1.64 (s, 3H), 1.11 (t, *J* = 7.1 Hz, 3H). **^{13}C NMR (150 MHz, CDCl$_3$)** δ 172.17, 166.41, 150.64, 144.37, 141.22, 140.71, 127.87, 127.12, 126.64, 124.88, 124.16, 120.02, 118.76, 110.58, 61.81, 48.70, 42.20, 21.15, 14.01. **ESI-MS:** calculated [$C_{21}H_{21}NO_3$ + H]$^+$: 336.1594, found: 336.1602. $[\alpha]^{20}_D$= +45.68 (c = 0.73, CH_2Cl_2). The product was analyzed by HPLC to determine the enantiomeric excess: 91% e.e. (CHIRALPAK IC, hexane/*i*-PrOH =95/5, detector: 254 nm, T = 25 °C, flow rate: 1 mL/min), t_1(major) = 7.42 min, t_2(minor) = 11.15 min.

Benzyl (*S*)-2-(benzo[d]oxazol-2-yl)-2-methyl-4-phenylpent-4-enoate (8aa)

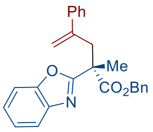

Reaction time: 3 h. **^{1}H NMR (500 MHz, CDCl$_3$)** δ 7.61 – 7.57 (m, 1H), 7.35 – 7.32 (m, 1H), 7.26 – 7.23 (m, 5H), 7.21 – 7.18 (m, 2H), 7.16 – 7.13 (m, 2H), 7.07 – 7.02 (m, 2H), 7.00 – 6.96 (m, 1H), 5.23 (d, *J* = 1.5 Hz, 1H), 5.08 (d, *J* = 1.5 Hz, 1H), 5.01 (d, *J* = 12.5 Hz, 1H), 4.90 (d, *J* = 12.5 Hz, 1H), 3.55 (d, *J* = 13.8 Hz, 1H), 3.46 (d, *J* = 13.9 Hz, 1H), 1.66 (s, 3H). **^{13}C NMR (150 MHz, CDCl$_3$)** δ 171.95, 166.19, 150.62, 144.22, 141.15, 140.68, 135.51, 128.52, 128.23, 127.91, 127.82, 127.16, 126.65, 124.95, 124.21, 120.04, 118.83, 110.56, 67.15, 48.85, 42.19, 21.10. **ESI-MS:** calculated [$C_{26}H_{23}NO_3$ + H]$^+$: 398.1751, found: 398.1761. $[\alpha]^{20}_D$= +32.68 (c = 0.86, CH_2Cl_2). The product was analyzed by HPLC to determine the enantiomeric excess: 85% e.e. (CHIRALPAK IC, hexane/*i*-PrOH =95/5, detector: 254 nm, T = 25 °C, flow rate: 1 mL/min), t_1(major) = 7.19 min, t_2(minor) = 11.25 min.

6. Large-scale reaction and transformation of products

6.1 Large-scale reaction

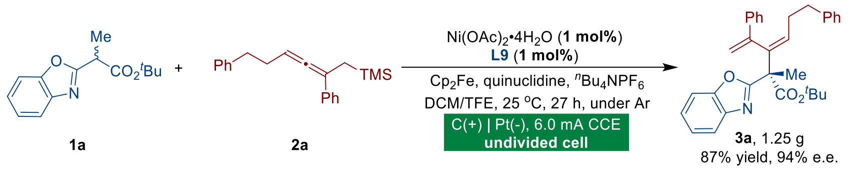

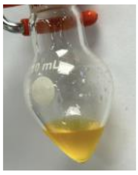

In a dried sealed tube, $Ni(OAc)_2·4H_2O$ (7.4 mg, 0.03 mmol) and **L9** (10.8 mg, 0.03 mmol) were

dissolved in DCM (3.0 mL) under N_2 atmosphere, and the mixture was stirred for 1 h at room temperature before use. A 30 mL flask equipped with a magnetic stir bar was charged with **1a** (0.74 g, 3.0 mmol), **2a** (1.37 g, 4.5 mmol), $^{n}Bu_4NPF_6$ (0.35 g, 0.9 mmol), quinuclidine (33.6 mg, 0.3 mmol), Cp_2Fe (5.4 mg, 0.03 mmol). The flask was equipped with a carbon plate (2.0 cm × 2.0 cm × 2.0 mm) as the anode and a platinum plate (2.0 cm × 2.0 cm × 0.2 mm) as the cathode. The reaction mixture was degassed via vacuum evacuation and backfilled with argon three times, followed by the addition via a syringe of nickel catalyst solution made in advance, DCM (9.0 mL) and TFE (6.0 mL). The constant current (6.0 mA) electrolysis was carried out at 25 °C for 27 h. After completion, The solution was diluted with ethyl acetate and washed with saturated NH_4Cl aqueous solution followed by distilled water washes. The aqueous phase was then extracted three times with ethyl acetate. The combined organic phase was dried over anhydrous $MgSO_4$ and concentrated under reduced pressure. The residue was purified by silica gel chromatography to afford the desired product **3a** (1.25 g, 87% yield, 94% e.e.).

6.2 Transformation of product 3f

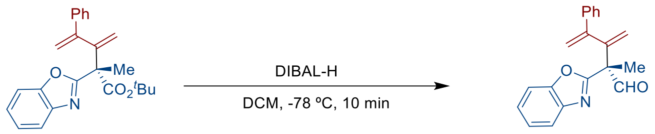

3f (96% e.e.) **9**, 68% yield, 96% e.e.

To a solution of **3f** (101.9 mg, 0.27 mmol, 96% e.e.) in DCM (1.5 mL) was added DIBAL-H (1.0 M in hexane, 0.59 mmol, 2.2 equiv.) dropwise via syringe at -78 °C under N_2 atmosphere. The reaction mixture was stirred at -78 °C for 10 min before pouring into a saturated aqueous potassium sodium tartrate solution (5 mL) with rapid stirring. The aqueous phase was extracted with Et_2O (20 mL × 3). The combined organic layers were washed with brine, dried over Na_2SO_4, and concentrated in vacuo. The crude products were purified by flash column chromatography (petroleum ether/EtOAc) to afford **9** (55.7 mg, 0.184 mmol, 68% yield, 96% e.e.).

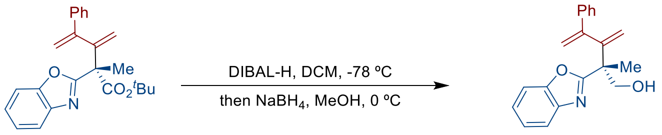

3f (96% e.e.) **10**, 69% yield, 96% e.e.

To a solution of **3f** (75.2 mg, 0.2 mmol, 96% e.e.) in DCM (1.0 mL) was added DIBAL-H (1.0 M in hexane, 0.44 mmol, 2.2 equiv.) dropwise via syringe at -78 °C under N_2 atmosphere. The reaction mixture was stirred at -78 °C for 10 min before pouring into a saturated aqueous potassium sodium tartrate solution (5 mL) with rapid stirring. The aqueous phase was extracted with Et_2O (20 mL × 3). The combined organic layers were washed with brine, dried over Na_2SO_4, and concentrated in vacuo. The crude product **9** was carried on without further purification. To a solution of crude product **9** in MeOH (1.0 mL) was added $NaBH_4$ (9.08 mg, 0.24 mmol, 1.2 equiv.) at 0 °C. The mixture was allowed to warm to room temperature and stirred for 1 h until complete consumption of **9** (monitored by TLC). The reaction was quenched with H_2O, and diluted with EtOAc. The aqueous phase was separated and extracted twice with EtOAc. The combined organic phase dried over anhydrous Na_2SO_4 and concentrated in vacuo. The crude products were purified by flash column chromatography (petroleum ether/EtOAc) to afford **10** (42.1 mg, 0.138 mmol, 69% yield, 96% e.e.).

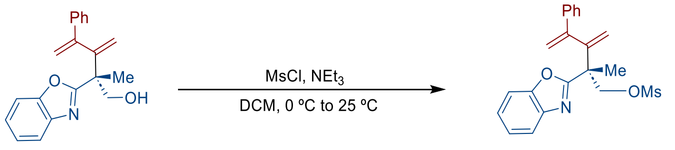

10 (96% e.e.) **11**, 94% yield, 96% e.e.

To a solution of **10** (188.3 mg, 0.62 mmol, 96% e.e.) in DCM (6.0 mL) was added Et_3N (103 uL, 0.74 mmol, 1.2 equiv.) and MsCl (79 uL, 0.74 mmol, 1.2 equiv.) at 0 °C. The mixture was allowed to warm to room temperature and stirred for 2 h until complete consumption of **10** (monitored by TLC). The reaction was quenched with H_2O, diluted with CH_2Cl_2. The aqueous phase was separated and extracted twice with CH_2Cl_2. The combined organic phase dried over anhydrous Na_2SO_4 and concentrated in vacuo. The crude products were purified by flash column chromatography (petroleum ether/EtOAc) to afford **11** (222.1 mg, 0.58 mmol, 94% yield, 96% e.e.).

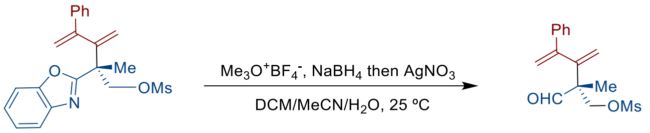

11 (96% e.e.) **12**, 73% yield, 96% e.e.

To a solution of **11** (38.4 mg, 0.1 mmol, 96% e.e.), activated 4 Å molecular sieves powders (200 mg), and anhydrous DCM (1.0 mL) were stirred at rt for 10 min, and then $Me_3O^+BF_4^-$ (74 mg, 0.5 mmol, 5.0 equiv.) was added. After stirring at rt for 2 h, another batch of $Me_3O^+BF_4^-$ (74 mg, 0.5 mmol, 5.0 equiv.) was added to the suspension, which was continued to react until **11** was consumed as determined by TLC. The reaction was concentrated without filtering off the molecular sieves to give the crude N-methylbenzothiazolium salt. The residue was redissolved in MeOH (1.5 mL), which was then cooled to 0 °C and added $NaBH_4$ (4.6 mg, 0.12 mmol, 1.2 equiv.). The mixture was allowed to warm to room temperature and stirred for 1 h until the starting material had been consumed as determined by TLC. The mixture was diluted with acetone, filtered through a pad of Celite, and concentrated to give the crude benzoxazolines. To a vigorously stirred solution of the crude benzoxazolines in CH_2Cl_2 (0.4 mL) and CH_3CN (2.0 mL) was added H_2O (0.24 mL) followed by $AgNO_3$ (51 mg, 0.3 mmol, 3.0 equiv.). The mixture was stirred at rt (monitored by TLC), and then diluted with 1 M phosphate buffer at pH 7 (0.2 mL). After stirring for 15 min, the reaction mixture was diluted with 1 M phosphate buffer at pH 7 (5 mL) and partially concentrated to remove CH_3CN. The suspension was extracted twice with EtOAc, The combined organic phase dried over anhydrous Na_2SO_4 and concentrated in vacuo. The crude products were purified by flash column chromatography (petroleum ether/EtOAc) to afford **12** (21.4 mg, 0.073 mmol, 73% yield, 96% e.e.).

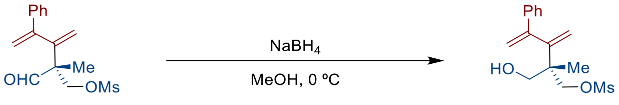

12 (96% e.e.) **13**, 92% yield, 96% e.e.

To a solution of crude product **12** (11.5 mg, 0.039 mmol, 96% e.e.) in MeOH (0.5 mL) was added $NaBH_4$ (1.77 mg, 0.047 mmol, 1.2 equiv.) at 0 °C. The mixture was allowed to warm to room temperature and stirred for 1 h until complete consumption of **12** (monitored by TLC). The reaction was quenched with H_2O, and diluted with EtOAc. The aqueous phase was separated and extracted twice with EtOAc. The combined organic phase dried over anhydrous Na_2SO_4 and concentrated in vacuo. The crude products were purified by flash column chromatography (petroleum ether/EtOAc) to afford **13** (10.6 mg, 0.036 mmol, 92% yield, 96% e.e.).

(*S*)-2-(benzo[d]oxazol-2-yl)-2-methyl-3-methylene-4-phenylpent-4-enal (9)

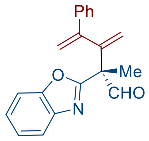

^{1}H NMR (400 MHz, $CDCl_3$) δ 10.02 (s, 1H), 7.67 – 7.62 (m, 1H), 7.49 – 7.43 (m, 1H), 7.34 – 7.29 (m, 2H), 7.25 – 7.20 (m, 2H), 7.19 – 7.13 (m, 3H), 5.51 (d, *J* = 0.9 Hz, 1H), 5.37 – 5.32 (m, 2H), 5.17 (d, *J* = 1.0 Hz, 1H), 1.68 (s, 3H). **^{13}C NMR (100 MHz, $CDCl_3$)**

δ 196.01, 164.74, 150.47, 148.59, 146.77, 140.69, 139.32, 128.26, 128.03, 127.14, 125.27, 124.55, 120.81, 120.21, 117.15, 110.74, 57.33, 18.83. **ESI-MS**: calculated $[C_{20}H_{17}NO_2 + H]^+$: 304.1332, found: 304.1335. $[\alpha]^{20}_{D}$= +22.40 (c = 0.57, CH_2Cl_2). The product was analyzed by HPLC to determine the enantiomeric excess: 96% e.e. (CHIRALPAK IC, hexane/*i*-PrOH =95/5, detector: 254 nm, T = 25 °C, flow rate: 1 mL/min), t_1(major) = 5.72 min, t_2(minor) = 6.61 min.

(*R*)-2-(benzo[d]oxazol-2-yl)-2-methyl-3-methylene-4-phenylpent-4-en-1-ol (10)

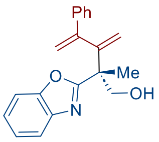

^{1}H NMR (400 MHz, CDCl$_3$) δ 7.62 – 7.54 (m, 1H), 7.46 – 7.39 (m, 1H), 7.33 – 7.26 (m, 4H), 7.17 – 7.04 (m, 3H), 5.34 (d, *J* = 1.4 Hz, 1H), 5.32 (d, *J* = 0.9 Hz, 1H), 5.22 (d, *J* = 0.9 Hz, 1H), 5.14 (d, *J* = 1.4 Hz, 1H), 4.14 (d, *J* = 11.3 Hz, 1H), 3.72 (d, *J* = 11.3 Hz, 1H), 3.43 (s, 1H), 1.52 (s, 3H). **^{13}C NMR (100 MHz, CDCl$_3$)** δ 168.99, 150.27, 149.72, 149.61, 140.39, 139.84, 128.08, 127.64, 126.73, 124.89, 124.32, 119.77, 118.53, 116.59, 110.67, 68.47, 48.40, 20.98. **ESI-MS**: calculated $[C_{20}H_{19}NO_2 + H]^+$: 306.1489, found: 306.1496. $[\alpha]^{20}_{D}$= -20.02 (c = 0.53, CH_2Cl_2). The product was analyzed by HPLC to determine the enantiomeric excess: 96% e.e. (CHIRALPAK IC, hexane/*i*-PrOH =90/0, detector: 254 nm, T = 25 °C, flow rate: 1 mL/min), t_1(major) = 6.72 min, t_2(minor) = 12.58 min.

(*R*)-2-(benzo[d]oxazol-2-yl)-2-methyl-3-methylene-4-phenylpent-4-en-1-yl methanesulfonate (11)

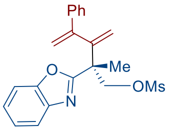

^{1}H NMR (500 MHz, CDCl$_3$) δ 7.56 – 7.51 (m, 1H), 7.39 – 7.34 (m, 1H), 7.30 – 7.26 (m, 1H), 7.26 – 7.22 (m, 1H), 7.21 – 7.16 (m, 2H), 7.08 – 6.99 (m, 3H), 5.44 (d, *J* = 0.5 Hz, 1H), 5.40 (d, *J* = 0.5 Hz, 1H), 5.34 (d, *J* = 1.5 Hz, 1H), 5.12 (d, *J* = 1.4 Hz, 1H), 4.79 (d, *J* = 9.5 Hz, 1H), 4.49 (d, *J* = 9.5 Hz, 1H), 2.93 (s, 3H), 1.72 (s, 3H). **^{13}C NMR (100 MHz, CDCl$_3$)** δ 166.10, 150.44, 148.98, 148.39, 140.71, 139.17, 128.05, 127.70, 126.60, 125.04, 124.34, 119.94, 119.19, 117.32, 110.61, 73.24, 46.09, 37.30, 21.09. **ESI-MS**: calculated $[C_{21}H_{21}NO_4S + H]^+$: 384.1264, found: 384.1274. $[\alpha]^{20}_{D}$= -43.19 (c = 0.56, CH_2Cl_2). The product was analyzed by HPLC to determine the enantiomeric excess: 96% e.e. (CHIRALPAK IC, hexane/*i*-PrOH =70/30, detector: 254 nm, T = 25 °C, flow rate: 1 mL/min), t_1(major) = 8.56 min, t_2(minor) = 9.34 min.

(*R*)-2-formyl-2-methyl-3-methylene-4-phenylpent-4-en-1-yl methanesulfonate (12)

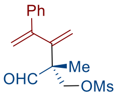

^{1}H NMR (400 MHz, CDCl$_3$) δ 9.29 (s, 1H), 7.37 – 7.30 (m, 5H), 5.48 (d, *J* = 0.4 Hz, 1H), 5.44 (d, *J* = 1.4 Hz, 1H), 5.34 (d, *J* = 0.4 Hz, 1H), 5.17 (d, *J* = 1.4 Hz, 1H), 4.37 (d, *J* = 9.8 Hz, 1H), 4.16 (d, *J* = 9.8 Hz, 1H), 2.93 (s, 3H), 1.26 (s, 3H). **^{13}C NMR (100 MHz, CDCl$_3$)** δ 198.47, 148.69, 145.50, 139.29, 128.81, 128.63, 126.96, 121.05, 117.32, 70.81, 54.79, 37.19, 16.95. **ESI-MS**: calculated $[C_{15}H_{18}O_4S + Na]^+$: 317.0818, found: 317.0822. $[\alpha]^{20}_{D}$= +34.29 (c = 0.45, CH_2Cl_2). The product was analyzed by HPLC to determine the enantiomeric excess: 96% e.e. (CHIRALPAK IE, hexane/*i*-PrOH =70/30, detector: 254 nm, T = 25 °C, flow rate: 1 mL/min), t_1(minor) = 8.62 min, t_2(major) = 9.05 min.

(*S*)-2-(hydroxymethyl)-2-methyl-3-methylene-4-phenylpent-4-en-1-yl methanesulfonate (13)

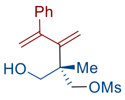

^{1}H NMR (500 MHz, CDCl$_3$) δ 7.44 – 7.39 (m, 2H), 7.37 – 7.28 (m, 3H), 5.42 (d, *J* = 1.7 Hz, 1H), 5.34 (d, *J* = 0.8 Hz, 1H), 5.29 (d, *J* = 0.6 Hz, 1H), 5.13 (d, *J* = 1.7 Hz, 1H), 4.18 (d, *J* = 9.6 Hz, 1H), 4.13 (d, *J* = 9.6 Hz, 1H), 3.52 (d, *J* = 11.4 Hz, 1H), 3.43 (d, *J* = 11.4 Hz, 1H), 2.93 (s, 3H), 0.99 (s, 3H). **^{13}C NMR (125 MHz, CDCl$_3$)** δ 150.03, 149.90, 140.25, 128.67, 128.24, 126.56, 117.87, 116.64, 72.30, 65.27, 44.81, 37.10, 19.78. **ESI-MS**: calculated $[C_{15}H_{20}O_4S +$

Na]$^+$: 319.0975, found: 319.0981. $[\alpha]^{20}_{D}$= -14.60 (c = 0.53, CH_2Cl_2). The product was analyzed by HPLC to determine the enantiomeric excess: 96% e.e. (CHIRALPAK IC, hexane/*i*-PrOH =70/30, detector: 254 nm, T = 25 °C, flow rate: 1 mL/min), t_1(major) = 7.61 min, t_2(minor) = 8.89 min.

6.3 Synthesis of chiral Boox ligands (*R*,*R*)-16 and (*R*,*S*)-16[12,13]

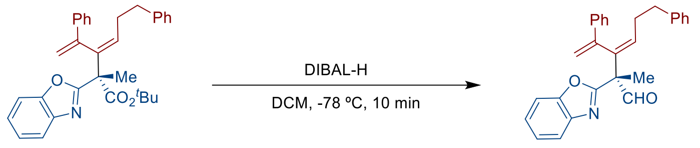

3a (94% e.e.) **14**, 77% yield, 94% e.e.

To a solution of **3a** (240 mg, 0.5 mmol, 94% e.e.) in DCM (3.0 mL) was added DIBAL-H (1.0 M in hexane, 1.1 mmol, 2.2 equiv.) dropwise via syringe at -78 °C under N_2 atmosphere. The reaction mixture was stirred at -78 °C for 10 min before pouring into a saturated aqueous potassium sodium tartrate solution (10 mL) with rapid stirring. The aqueous phase was extracted with Et_2O (20 mL × 3). The combined organic layers were washed with brine, dried over Na_2SO_4, and concentrated in vacuo. The crude products were purified by flash column chromatography (petroleum ether/EtOAc) to afford **14** (157.6 mg, 0.385 mmol, 77% yield, 94% e.e.).

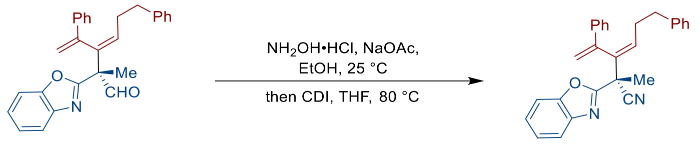

14 (94% e.e.) **15**, 80% yield, 94% e.e.

To a solution of **14** (396.9 mg, 0.975 mmol, 94% e.e.) in EtOH (10 mL) was added sodium acetate (224 mg, 2.73 mmol, 2.8 equiv.) and NH_2OH•HCl (190 mg, 2.73 mmol, 2.8 equiv.) at room temperature. The mixture was stirred at room temperature for 2 h and concentrated in vacuo. The residue was diluted with ethyl acetate, washed with water, saturated aqueous sodium chloride solution, dried over Na_2SO_4, filtered, and concentrated in vacuo. The crude product was then dissolved in anhydrous tetrahydrofuran (10 mL) and treated with 1,1-carbonyldiimidazole (1.26 g, 7.8 mmol, 8.0 equiv.) before being heated at reflux for 2 h under N_2 atmosphere. The reaction was then cooled down to 0 °C and water (5.0 mL) was added slowly before the resulting mixture was stirred for an additional hour. The reaction was diluted with ethyl acetate and washed with water, saturated with aqueous sodium chloride solution, dried over Na_2SO_4, and concentrated in vacuo. The residue was purified through flash column chromatography (petroleum ether/EtOAc) to afford **15** (316.2 mg, 0.777 mmol, 80% yield, 94% e.e.).

Synthesis of chiral Boox ligands (*R*,*R*)-16

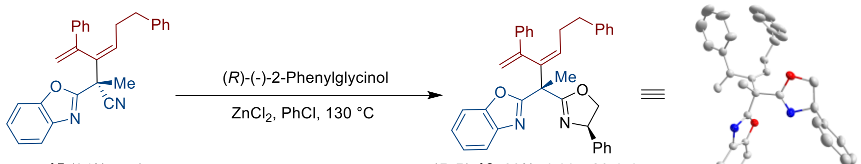

15 (94% e.e.) (*R*,*R*)-**16**, 60% yield, >20:1 d.r. CCDC (**2343662**)

To a solution of **15** (43.2 mg, 0.107 mmol, 94% e.e.) and (*R*)-(-)-2-Phenylglycinol (16.1 mg, 0.118 mmol, 1.1 equiv.) in PhCl (1.0 mL) was added $ZnCl_2$ (43.6 mg, 0.32 mmol, 3.0 equiv.) at room temperature under N_2 atmosphere. The mixture was stirred vigorously and heated at 130 °C for 24 h.

After cooling to rt, saturated aqueous NH_4Cl was added to the mixture and allowed to stir overnight. The organic phase was extracted twice with EtOAc. The combined organic layers were washed with brine, dried over Na_2SO_4, and concentrated in vacuo. The crude products were purified by flash column chromatography (petroleum ether/EtOAc) to afford chiral Boox ligands (*R*,*R*)-**16** (33.6 mg, 0.064 mmol, 60% yield, >20:1 d.r.).

Synthesis of chiral Boox ligands (*R*,*S*)-16

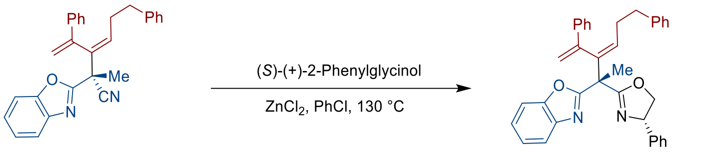

15 (94% e.e.)   (*R*,*S*)-**16**, 51% yield, >20:1 d.r.

To a solution of **15** (49.8 mg, 0.123 mmol, 94% e.e.) and (*S*)-(+)-2-Phenylglycinol (18.6 mg, 0.135 mmol, 1.1 equiv.) in PhCl (1.0 mL) was added $ZnCl_2$ (50.3 mg, 0.369 mmol, 3.0 equiv.) at room temperature under N_2 atmosphere. The mixture was stirred vigorously and heated at 130 °C for 72 h. After cooling to rt, saturated aqueous NH_4Cl was added to the mixture and allowed to stir overnight. The organic phase was extracted twice with EtOAc. The combined organic layers were washed with brine, dried over Na_2SO_4, and concentrated in vacuo. The crude products were purified by flash column chromatography (petroleum ether/EtOAc) to afford chiral Boox ligands (*R*,*S*)-**16** (33.0 mg, 0.063 mmol, 51% yield, >20:1 d.r.).

(*S*,*E*)-2-(benzo[d]oxazol-2-yl)-2-methyl-6-phenyl-3-(1-phenylvinyl)hex-3-enal (14)

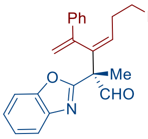

^{1}H NMR (400 MHz, CDCl$_3$) δ 9.92 (s, 1H), 7.70 – 7.64 (m, 1H), 7.50 – 7.44 (m, 1H), 7.36 – 7.29 (m, 2H), 7.21 – 7.10 (m, 8H), 7.07 – 7.00 (m, 2H), 5.70 (t, *J* = 7.3 Hz, 1H), 5.56 (d, *J* = 1.0 Hz, 1H), 4.76 (d, *J* = 1.0 Hz, 1H), 2.74 – 2.61 (m, 2H), 2.50 – 2.41 (m, 2H), 1.54 (s, 3H). **^{13}C NMR (100 MHz, CDCl$_3$)** δ 195.96, 165.24, 150.37, 144.06, 141.14, 140.62, 138.53, 137.70, 134.59, 128.74, 128.45, 128.39, 128.01, 126.16, 126.10, 125.19, 124.49, 120.15, 117.35, 110.71, 57.55, 35.83, 32.03, 18.01. **ESI-MS**: calculated $[C_{28}H_{25}NO_2+ H]^+$: 408.1958, found: 408.1970. $[\alpha]^{20}_D$= +70.55 (c = 1.15, CH_2Cl_2). The product was analyzed by HPLC to determine the enantiomeric excess: 94% e.e. (CHIRALPAK IG, hexane/*i*-PrOH =95/5, detector: 300 nm, T = 25 °C, flow rate: 1 mL/min), t_1(major) = 7.79 min, t_2(minor) = 8.65 min.

(*R*,*E*)-2-(benzo[d]oxazol-2-yl)-2-methyl-6-phenyl-3-(1-phenylvinyl)hex-3-enenitrile (15)

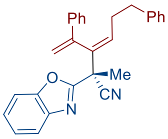

^{1}H NMR (400 MHz, CDCl$_3$) δ 7.58 – 7.53 (m, 1H), 7.41 – 7.36 (m, 1H), 7.33 – 7.20 (m, 4H), 7.19 – 7.14 (m, 1H), 7.13 – 7.08 (m, 2H), 7.03 – 6.91 (m, 5H), 6.39 (t, *J* = 7.3 Hz, 1H), 5.54 (d, *J* = 0.8 Hz, 1H), 4.70 (d, *J* = 0.8 Hz, 1H), 2.83 – 2.71 (m, 2H), 2.54 – 2.43 (m, 2H), 1.92 (s, 3H). **^{13}C NMR (100 MHz, CDCl$_3$)** δ 161.41, 150.77, 142.51, 141.06, 140.37, 137.89, 136.54, 133.07, 128.79, 128.45, 128.12, 127.70, 126.16, 125.87, 125.66, 124.64, 120.38, 119.25, 118.92, 110.87, 44.32, 35.66, 31.91, 23.93. **ESI-MS**: calculated $[C_{28}H_{24}N_2O + H]^+$: 405.1961, found: 405.1962. $[\alpha]^{20}_D$= +37.72 (c = 1.15, CH_2Cl_2). The product was analyzed by HPLC to determine the enantiomeric excess: 94% e.e. (CHIRALPAK IA, hexane/*i*-PrOH =95/5, detector: 285 nm, T = 25 °C, flow rate: 1 mL/min), t_1(major) = 5.15 min, t_2(minor) = 6.05 min.

2-((*R*,*E*)-6-phenyl-2-((*R*)-4-phenyl-4,5-dihydrooxazol-2-yl)-3-(1-phenylvinyl)hex-3-en-2-

yl)benzo[d]oxazole ((*R*,*R*)-16)

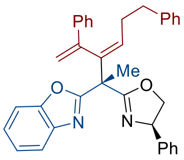

^{1}H NMR (500 MHz, CDCl$_3$) δ 7.64 – 7.59 (m, 1H), 7.46 – 7.41 (m, 1H), 7.26 – 7.19 (m, 6H), 7.18 – 7.14 (m, 1H), 7.13 – 7.03 (m, 6H), 7.03 – 6.95 (m, 4H), 5.76 (t, *J* = 7.3 Hz, 1H), 5.52 (d, *J* = 1.4 Hz, 1H), 5.11 (dd, *J* = 10.0, 8.6 Hz, 1H), 4.86 (d, *J* = 1.2 Hz, 1H), 4.56 (dd, *J* = 10.2, 8.4 Hz, 1H), 3.92 (dd, *J* = 8.6, 8.2 Hz, 1H), 2.67 – 2.55 (m, 2H), 2.43 – 2.32 (m, 2H), 1.79 (s, 3H). **^{13}C NMR (100 MHz, CDCl$_3$)** δ 168.60, 166.43, 150.90, 144.53, 142.01, 141.55, 140.97, 139.24, 139.00, 132.76, 128.77, 128.70, 128.31, 128.29, 127.72, 127.60, 126.77, 126.31, 125.95, 124.99, 124.22, 120.33, 117.51, 110.84, 75.38, 69.55, 49.71, 35.84, 32.04, 23.79. **ESI-MS**: calculated [$C_{36}H_{32}N_2O_2$ + H]$^+$: 525.2537, found: 525.2541. $[\alpha]^{20}_D$= +10.60 (c = 0.62, CH_2Cl_2).

2-((*R*,*E*)-6-phenyl-2-((*S*)-4-phenyl-4,5-dihydrooxazol-2-yl)-3-(1-phenylvinyl)hex-3-en-2-yl)benzo[d]oxazole ((*R*,*S*)-16)

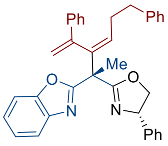

^{1}H NMR (500 MHz, CDCl$_3$) δ 7.74 – 7.70 (m, 1H), 7.55 – 7.50 (m, 1H), 7.36 – 7.28 (m, 6H), 7.26 – 7.16 (m, 8H), 7.15 – 7.11 (m, 1H), 7.09 – 7.06 (m, 2H), 5.80 (t, *J* = 7.3 Hz, 1H), 5.59 (d, *J* = 1.4 Hz, 1H), 5.02 – 4.93 (m, 2H), 4.41 (dd, *J* = 10.1, 8.5 Hz, 1H), 4.08 (dd, *J* = 8.6, 8.0 Hz, 1H), 2.73 – 2.65 (m, 2H), 2.51 – 2.41 (m, 2H), 1.86 (s, 3H). **^{13}C NMR (125 MHz, CDCl$_3$)** δ 168.44, 166.53, 150.94, 144.64, 142.22, 141.57, 141.07, 139.48, 139.19, 132.68, 128.80, 128.73, 128.32, 128.24, 127.70, 127.63, 126.89, 126.46, 125.95, 125.03, 124.25, 120.40, 117.57, 110.81, 75.35, 69.59, 49.64, 35.89, 32.03, 23.91. **ESI-MS**: calculated [$C_{36}H_{32}N_2O_2$ + H]$^+$: 525.2537, found: 525.2546. $[\alpha]^{20}_D$= -43.99 (c = 0.60, CH_2Cl_2).

6.4 Synthesis of chiral Boox ligands (*R*,*R*)-19 and (*R*,*S*)-19[12,13]

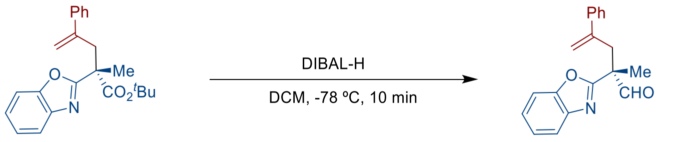

8a (94% e.e.) **17**, 67% yield, 94% e.e.

To a solution of **8a** (135.1 mg, 0.372 mmol, 94% e.e.) in DCM (2.0 mL) was added DIBAL-H (1.0 M in hexane, 0.82 mmol, 2.2 equiv.) dropwise via syringe at -78 °C under N_2 atmosphere. The reaction mixture was stirred at -78 °C for 10 min before pouring into a saturated aqueous potassium sodium tartrate solution (10 mL) with rapid stirring. The aqueous phase was extracted with Et_2O (20 mL × 3). The combined organic layers were washed with brine, dried over Na_2SO_4, and concentrated in vacuo. The crude products were purified by flash column chromatography (petroleum ether/EtOAc) to afford **17** (72.2 mg, 0.248 mmol, 67% yield, 94% e.e.).

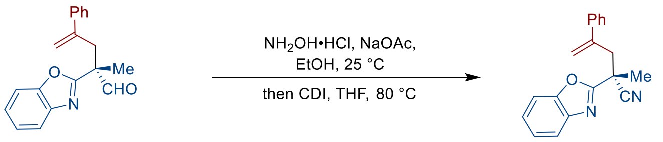

17 (94% e.e.) **18**, 83% yield, 94% e.e.

To a solution of **17** (258.3 mg, 0.887 mmol, 94% e.e.) in EtOH (10 mL) was added sodium acetate (203.7 mg, 2.48 mmol, 2.8 equiv.) and NH_2OH•HCl (172.6 mg, 2.48 mmol, 2.8 equiv.) at room temperature. The mixture was stirred at room temperature for 2 h and concentrated in vacuo. The residue

was diluted with ethyl acetate, washed with water, saturated aqueous sodium chloride solution, dried over Na_2SO_4, filtered, and concentrated in vacuo. The crude product was then dissolved in anhydrous tetrahydrofuran (10 mL) and treated with 1,1-carbonyldiimidazole (196 mg, 7.1 mmol, 8.0 equiv.) before being heated at reflux for 2 h under N_2 atmosphere. The reaction was then cooled down to 0 °C and water (5.0 mL) was added slowly before the resulting mixture was stirred for an additional hour. The reaction was diluted with ethyl acetate and washed with water, saturated with aqueous sodium chloride solution, dried over Na_2SO_4, and concentrated in vacuo. The residue was purified through flash column chromatography (petroleum ether/EtOAc) to afford **18** (212.7 mg, 0.737 mmol, 83% yield, 94% e.e.).

chiral Boox ligands (*R*,*R*)-19

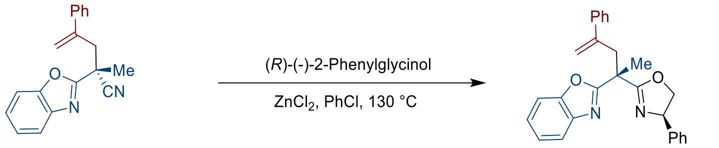

18 (94% e.e.) (*R*,*R*)-**19**, 59% yield, >20:1 d.r.

To a solution of **18** (51.5 mg, 0.179 mmol, 94% e.e.) and (*R*)-(-)-2-Phenylglycinol (27.0 mg, 0.197 mmol, 1.1 equiv.) in PhCl (1.0 mL) was added $ZnCl_2$ (73.2 mg, 0.537 mmol, 3.0 equiv.) at room temperature under N_2 atmosphere. The mixture was stirred vigorously and heated at 130 ℃ for 24 h. After cooling to rt, saturated aqueous NH_4Cl was added to the mixture and allowed to stir overnight. The organic phase was extracted twice with EtOAc. The combined organic layers were washed with brine, dried over Na_2SO_4, and concentrated in vacuo. The crude products were purified by flash column chromatography (petroleum ether/EtOAc) to afford chiral Boox ligands (*R*,*R*)-**19** (43 mg, 0.105 mmol, 59% yield, >20:1 d.r.).

Synthesis of chiral Boox ligands (*R*,*S*)-19

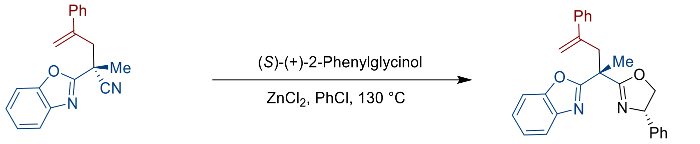

18 (94% e.e.) (*R*,*S*)-**19**, 55% yield, 18:1 d.r.

To a solution of **18** (51.5 mg, 0.179 mmol, 94% e.e.) and (*S*)-(+)-2-Phenylglycinol (27.0 mg, 0.197 mmol, 1.1 equiv.) in PhCl (1.0 mL) was added $ZnCl_2$ (73.2 mg, 0.537 mmol, 3.0 equiv.) at room temperature under N_2 atmosphere. The mixture was stirred vigorously and heated at 130 ℃ for 24 h. After cooling to rt, saturated aqueous NH_4Cl was added to the mixture and allowed to stir overnight. The organic phase was extracted twice with EtOAc. The combined organic layers were washed with brine, dried over Na_2SO_4, and concentrated in vacuo. The crude products were purified by flash column chromatography (petroleum ether/EtOAc) to afford chiral Boox ligands (*R*,*S*)-**19** (40.4 mg, 0.099 mmol, 55% yield, 18:1 d.r.).

(*S*)-2-(benzo[d]oxazol-2-yl)-2-methyl-4-phenylpent-4-enal (17)

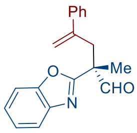

^{1}H NMR (400 MHz, $CDCl_3$) δ 9.72 (s, 1H), 7.55 – 7.48 (m, 1H), 7.32 – 7.26 (m, 1H), 7.22 – 7.17 (m, 2H), 7.12 – 7.07 (m, 2H), 7.00 – 6.94 (m, 2H), 6.93 – 6.86 (m, 1H), 5.17 (d, *J* = 1.2 Hz, 1H), 5.02 (d, *J* = 1.2 Hz, 1H), 3.35 (d, *J* = 14.0 Hz, 1H), 3.15 (d, *J* = 14.0 Hz, 1H), 1.47 (s, 3H). **^{13}C NMR (100 MHz, $CDCl_3$)** δ 198.39, 164.91, 150.52, 143.81,

140.75, 140.73, 128.05, 127.38, 126.51, 125.09, 124.41, 120.00, 118.49, 110.61, 52.76, 41.51, 17.78. **ESI-MS**: calculated $[C_{19}H_{17}NO_2 + H]^+$: 292.1332, found: 292.1339. $[\alpha]^{20}_D$= +28.65 (c = 0.51, CH_2Cl_2). The product was analyzed by HPLC to determine the enantiomeric excess: 94% e.e. (CHIRALPAK IC, hexane/*i*-PrOH =95/5, detector: 254 nm, T = 25 °C, flow rate: 1 mL/min), t_1(major) = 6.27 min, t_2(minor) = 7.28 min.

(*R*)-2-(benzo[d]oxazol-2-yl)-2-methyl-4-phenylpent-4-enenitrile (18)

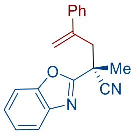

^{1}H NMR (500 MHz, $CDCl_3$) δ 7.61 – 7.55 (m, 1H), 7.39 – 7.33 (m, 1H), 7.31 – 7.25 (m, 2H), 7.22 – 7.16 (m, 2H), 7.07 – 7.00 (m, 2H), 7.00 – 6.92 (m, 1H), 5.41 (d, *J* = 0.8 Hz, 1H), 5.31 (d, *J* = 0.8 Hz, 1H), 3.45 – 3.31 (m, 2H), 1.86 (s, 3H). **^{13}C NMR (125 MHz, $CDCl_3$)** δ 162.33, 150.66, 142.56, 140.43, 139.89, 128.09, 127.53, 126.45, 125.62, 124.75, 120.37, 119.75, 119.62, 110.84, 45.19, 39.36, 24.17. **ESI-MS**: calculated $[C_{19}H_{16}N_2O + H]^+$: 289.1335, found: 289.1343. $[\alpha]^{20}_D$= -15.62 (c = 0.67, CH_2Cl_2). The product was analyzed by HPLC to determine the enantiomeric excess: 94% e.e. (CHIRALPAK IG, hexane/*i*-PrOH =95/5, detector: 254 nm, T = 25 °C, flow rate: 1 mL/min), t_1(major) = 9.82 min, t_2(minor) = 13.14 min.

2-((*R*)-4-phenyl-2-((*R*)-4-phenyl-4,5-dihydrooxazol-2-yl)pent-4-en-2-yl)benzo[d]oxazole ((*R,R*)-19)

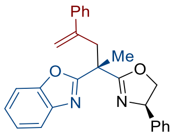

^{1}H NMR (500 MHz, $CDCl_3$) δ 7.63 – 7.59 (m, 1H), 7.43 – 7.39 (m, 1H), 7.36 – 7.32 (m, 2H), 7.30 – 7.25 (m, 5H), 7.19 – 7.16 (m, 2H), 7.08 – 7.02 (m, 2H), 7.00 – 6.93 (m, 1H), 5.29 (d, *J* = 1.5 Hz, 1H), 5.19 – 5.10 (m, 2H), 4.48 (dd, *J* = 10.2, 8.4 Hz, 1H), 3.87 (dd, *J* = 8.5 Hz, 1H), 3.66 (d, *J* = 13.9 Hz, 1H), 3.57 (d, *J* = 13.8 Hz, 1H), 1.75 (s, 3H). **^{13}C NMR (125 MHz, $CDCl_3$)** δ 169.09, 166.75, 150.75, 144.40, 141.94, 141.30, 140.81, 128.75, 127.93, 127.70, 127.13, 126.88, 126.62, 124.93, 124.18, 120.17, 118.97, 110.61, 75.28, 69.74, 43.33, 42.74, 21.84. **ESI-MS**: calculated $[C_{27}H_{24}N_2O_2 + H]^+$: 409.1911, found: 409.1917. $[\alpha]^{20}_D$= +155.58 (c = 0.62, CH_2Cl_2).

2-((*R*)-4-phenyl-2-((*S*)-4-phenyl-4,5-dihydrooxazol-2-yl)pent-4-en-2-yl)benzo[d]oxazole ((*R,S*)-19)

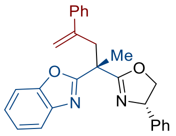

^{1}H NMR (500 MHz, $CDCl_3$) δ 7.70 – 7.64 (m, 1H), 7.46 – 7.42 (m, 1H), 7.36 – 7.26 (m, 7H), 7.21 – 7.15 (m, 4H), 7.11 – 7.07 (m, 1H), 5.31 (d, *J* = 1.6 Hz, 1H), 5.21 (d, *J* = 1.5 Hz, 1H), 4.74 (dd, *J* = 10.2, 7.8 Hz, 1H), 4.09 (dd, *J* = 10.3, 8.4 Hz, 1H), 3.89 (dd, *J* = 8.1 Hz, 1H), 3.70 (d, *J* = 13.7 Hz, 1H), 3.51 (d, *J* = 13.7 Hz, 1H), 1.81 (s, 3H). **^{13}C NMR (125 MHz, $CDCl_3$)** δ 168.32, 167.12, 150.80, 144.43, 142.15, 141.61, 140.85, 128.78, 127.89, 127.70, 127.15, 126.97, 126.75, 125.02, 124.28, 120.20, 119.17, 110.66, 75.14, 69.43, 42.84, 42.50, 22.08. **ESI-MS**: calculated $[C_{27}H_{24}N_2O_2 + H]^+$: 409.1911, found: 409.1919. $[\alpha]^{20}_D$= -106.07 (c = 0.57, CH_2Cl_2).

7. Evaluation of chiral Boox ligands

***Table S16*: Evaluation of chiral Boox ligands in the enantioselective anodic oxidative dienylation reaction[a]**

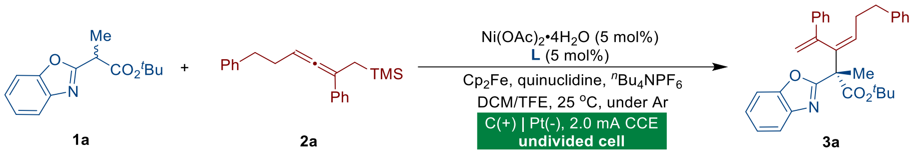

Entry	L	Yield (%)	e.e. (%) of **3a**
1	(*R*,*R*)-**16**	52	93 (*S*)
2	(*R*,*S*)-**16**	53	68 (*R*)
3	(*R*,*R*)-**19**	43	81 (*S*)
4	(*R*,*S*)-**19**	48	46 (*R*)

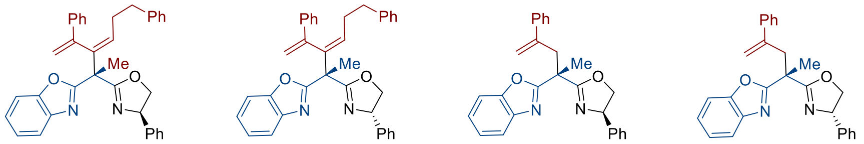

[a]Reactions were carried out with **1a** (0.1 mmol), **2a** (0.15 mmol), $^nBu_4NPF_6$ (0.15 mmol), quinuclidine (0.05 mmol), $Ni(OAc)_2{\cdot}4H_2O$ (5 mol%), **L** (5 mol%), Cp_2Fe (10 mol%) and DCM/TFE = 2:1 (3 mL) at 25 °C for 3 h in an undivided cell. TFE, 2,2,2-trifluoroethanol.

8. Cyclic voltammetry studies

Unless otherwise noted, the cyclic voltammograms were recorded on a CHI 760E instrument using a glassy carbon disk working electrode (diameter, 3 mm), a Pt wire auxiliary electrode, an SCE reference electrode, and a scan rate of 100 mV/s.

Definitions

Onset Potential (E_{onset}) - The potential at which the extrapolation of the linear portion of the CV baseline and the extrapolation of the linear portion of the electrochemical wave intersect one another[14].

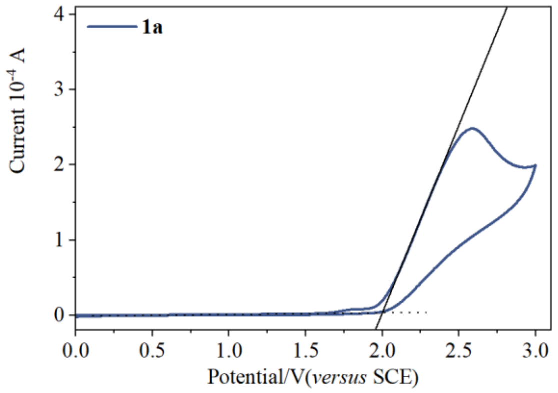

Figure S1. Cyclic voltammogram of **1a** (33 mM) in an electrolyte of $^nBu_4NPF_6$ (0.05 M) in DCM/TFE = 2:1 (3 mL). The oxidation peak of **1a** was observed at +2.58 V (*versus* SCE). The onset potential of **1a** was measured approximately at +1.98 V (*versus* SCE).

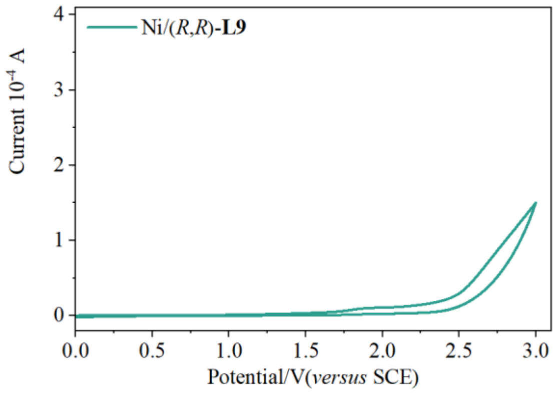

Figure S2. Cyclic voltammogram of Ni/(*R*,*R*)-**L9** (3.3 mM) in an electrolyte of $^{n}Bu_4NPF_6$ (0.05 M) in DCM/TFE = 2:1 (3 mL). The oxidation peak of Ni/(*R*,*R*)-**L9** was observed at +1.91 V (*versus* SCE).

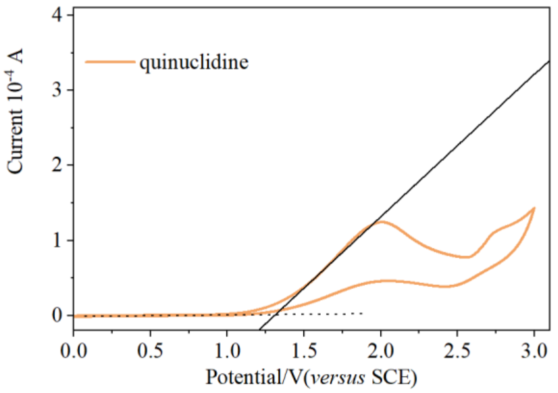

Figure S3. Cyclic voltammogram of quinuclidine (16.7 mM) in an electrolyte of $^{n}Bu_4NPF_6$ (0.05 M) in DCM/TFE = 2:1 (3 mL). The oxidation peak of quinuclidine was observed at +2.01 V (*versus* SCE). The onset potential of quinuclidine was measured approximately at +1.3 V (*versus* SCE).

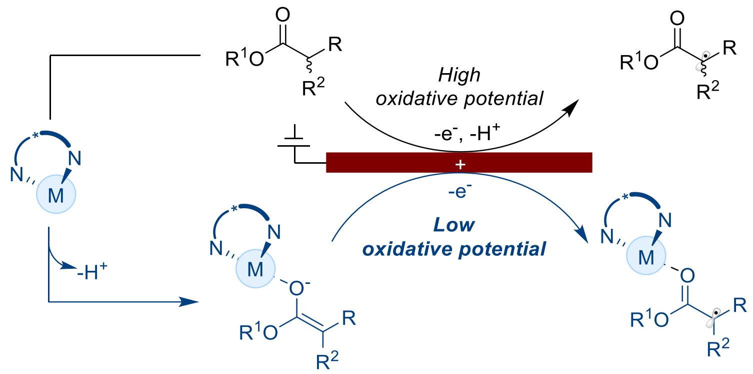

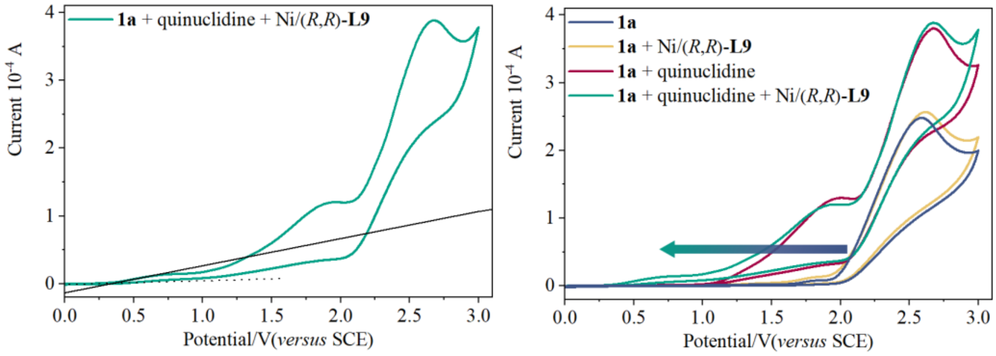

Figure S4. Cyclic voltammogram of [**1a** (33 mM) + quinuclidine (16.7 mM) + Ni/(*R*,*R*)-**L9** (3.3 mM)] in an electrolyte of ^{n}Bu$_4$NPF$_6$ (0.05 M) in DCM/TFE = 2:1 (3 mL). The onset potential of [**1a** + quinuclidine + Ni/(*R*,*R*)-**L9**] was measured approximately at +0.36 V (*versus* SCE). Cyclic voltammetry (CV) experiments show that the onset potential of nickel–bound–enolate intermediate is significantly decreased.

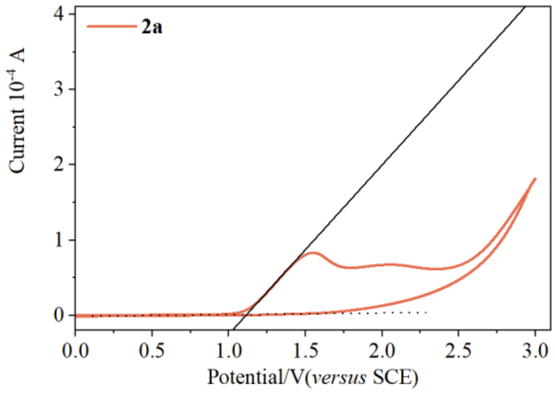

Figure S5. Cyclic voltammogram of **2a** (50 mM) in an electrolyte of ^{n}Bu$_4$NPF$_6$ (0.05 M) in DCM/TFE = 2:1 (3 mL). The oxidation peak of **2a** was observed at +1.56 V and 2.10 V (*versus* SCE). The onset potential of **2a** was measured approximately at +1.11 V (*versus* SCE).

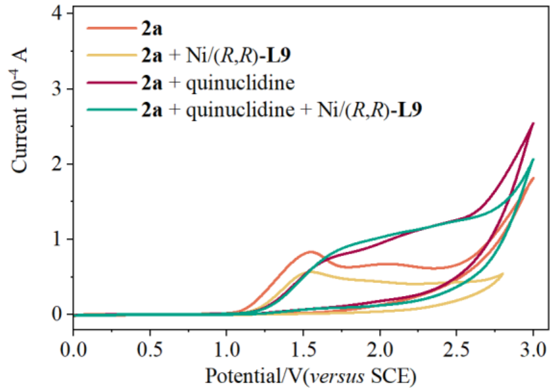

Figure S6. Cyclic voltammogram of [**2a** (50 mM) + Ni/(*R*,*R*)-**L9** (3.3 mM)], [**2a** (50 mM) + quinuclidine (16.7 mM)] and [**2a** (50 mM) + quinuclidine (16.7 mM) + Ni/(*R*,*R*)-**L9** (3.3 mM)] in an electrolyte of ^{n}Bu$_4$NPF$_6$ (0.05 M) in DCM/TFE = 2:1 (3 mL). Indicates that there is no interaction between the Ni/(*R*,*R*)-**L9** and **2a**.

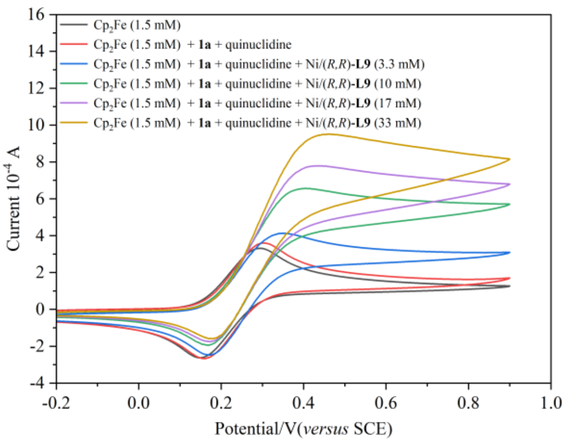

Figure S7. Titration of Ni/(*R*,*R*)-**L9** (3.3 mM to 33 mM) to Cp_2Fe (1.5 mM) monitored by cyclic voltammetry in the presence of **1a** (33 mM), quinuclidine (17 mM), $^nBu_4NPF_6$ (50 mM) in DCM/TFE = 2:1 (3 mL). A decrease in ferrocene's reduction peak were observed with increasing concentrations of Ni/(*R*,*R*)-**L9**.

9. Controlled experiments

9.1 Electric current turned on and off experiments.

In a dried sealed tube, $Ni(OAc)_2 \cdot 4H_2O$ (12.4 mg, 0.05 mmol) and **L9** (18.0 mg, 0.05 mmol) were dissolved in CD_2Cl_2 (3.0 mL) under N_2 atmosphere, and the mixture was stirred for 1 h at room temperature before use. A 30 mL flask equipped with a magnetic stir bar was charged with **1a** (124.5 mg, 0.5 mmol), **2a** (455 mg, 1.5 mmol), $^nBu_4NPF_6$ (290 mg, 0.75 mmol), quinuclidine (28 mg, 0.25 mmol), Cp_2Fe (9.3 mg, 0.05 mmol) and 1,1,2,2-Tetrachloroethane (42 mg, 0.25 mmol, as an internal standard). The flask was equipped with a carbon plate (2.0 cm × 2.0 cm × 2.0 mm) as the anode and a platinum plate (2.0 cm × 2.0 cm × 0.2 mm) as the cathode. The reaction mixture was degassed via vacuum evacuation and backfilled with argon three times, followed by the addition via a syringe of nickel catalyst solution made in advance, CD_2Cl_2 (7.0 mL) and TFE (5.0 mL). The constant current (8.0 mA) electrolysis was carried out at 25 °C. The electric current was switched off after half an hour at which point a reaction solution (0.5 mL) was taken and analyzed by ^1H NMR spectroscopy. The electric current was switched on after half an hour at which point a reaction solution (0.5 mL) was taken and analyzed by ^1H NMR spectroscopy. Repeated the above steps, The electric current was switched on/off every half an hour at which point a reaction solution (0.5 mL) was taken and analyzed by ^1H NMR spectroscopy. Yields were determined by ^1H NMR using 1,1,2,2-Tetrachloroethane as an internal standard (*Table S17* and *Figure S8*).

Table S17: Electric current turned on and off experiments.

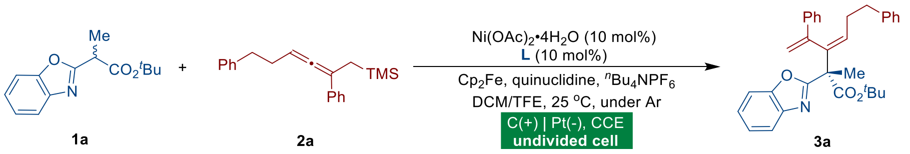

Entry	Time (h)	Yield (%)
1	0	0
2	0.5	21
3	1	21
4	1.5	39
5	2	39
6	2.5	56
7	3	56
8	3.5	72
9	4	72

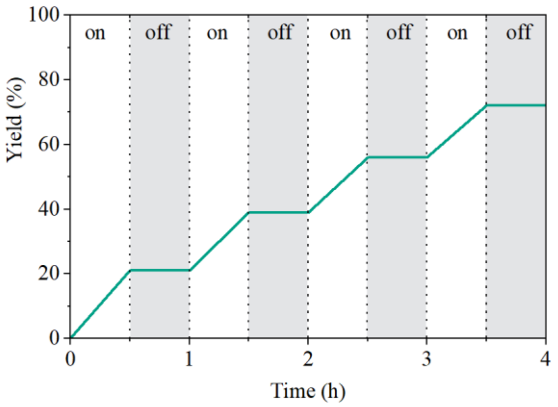

Figure S8. Profile of the current on-off interval experiment. The current-switched on-off experiment revealed that coupling product **3a** was formed only upon exposure of the system to electric conditions.

9.2 Kinetic studies to investigate the racemic background reaction

With Ni/(*R,R*)-L9: In a dried sealed tube, $Ni(OAc)_2 \cdot 4H_2O$ (12.4 mg, 0.05 mmol) and **L9** (18 mg, 0.05 mmol) were dissolved in CD_2Cl_2 (3.0 mL) under N_2 atmosphere, and the mixture was stirred for 1 h at room temperature before use. A 30 mL flask equipped with a magnetic stir bar was charged with **1a** (124.5 mg, 0.5 mmol), **2a** (455 mg, 1.5 mmol), ^{n}Bu$_4$NPF$_6$ (290 mg, 0.75 mmol), quinuclidine (28 mg, 0.25 mmol), Cp_2Fe (9.3 mg, 0.05 mmol) and 1,1,2,2-Tetrachloroethane (42 mg, 0.25 mmol, as an internal standard). The flask was equipped with a carbon plate (2.0 cm × 2.0 cm × 2.0 mm) as the anode and a platinum plate (2.0 cm × 2.0 cm × 0.2 mm) as the cathode. The reaction mixture was degassed via vacuum evacuation and backfilled with argon three times, followed by the addition via a syringe of nickel catalyst solution made in advance, CD_2Cl_2 (7.0 mL) and TFE (5.0 mL). The constant current (8.0 mA) electrolysis was carried out at 25 °C. A reaction solution (0.5 mL) was taken and analyzed by ^{1}H NMR spectroscopy every half an hour. Yields were determined by ^{1}H NMR using 1,1,2,2-Tetrachloroethane as an internal standard (*Table S18*).

Table S18: With Ni/(*R*,*R*)-**L9** reaction monitoring

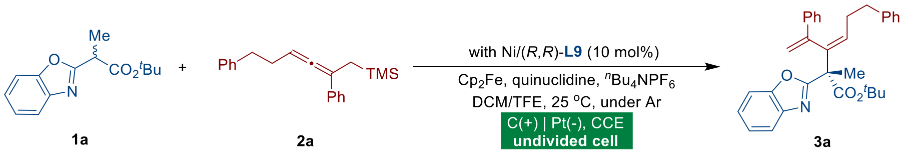

Entry	Time (h)	Yield (%)
1	0	0
2	0.5	21
3	1	39
4	1.5	56
5	2	72
6	2.5	86
7	3	95

Without Ni/(*R*,*R*)-L9: A 30 mL flask equipped with a magnetic stir bar was charged with **1a** (124.5 mg, 0.5 mmol), **2a** (455 mg, 1.5 mmol), $^{n}Bu_4NPF_6$ (290 mg, 0.75 mmol), quinuclidine (28 mg, 0.25 mmol), Cp_2Fe (9.3 mg, 0.05 mmol) and 1,1,2,2-tetrachloroethane (42 mg, 0.25 mmol, as an internal standard). The flask was equipped with a carbon plate (2.0 cm × 2.0 cm × 2.0 mm) as the anode and a platinum plate (2.0 cm × 2.0 cm × 0.2 mm) as the cathode. The reaction mixture was degassed via vacuum evacuation and backfilled with argon three times, followed by the addition via a syringe of CD_2Cl_2 (10.0 mL) and TFE (5.0 mL). The constant current (8.0 mA) electrolysis was carried out at 25 °C. A reaction solution (0.5 mL) was taken and analyzed by ^{1}H NMR spectroscopy every half an hour. Yields were determined by ^{1}H NMR using 1,1,2,2-Tetrachloroethane as an internal standard (*Table S19*).

Table S19: Without Ni/(*R*,*R*)-**L9** reaction monitoring

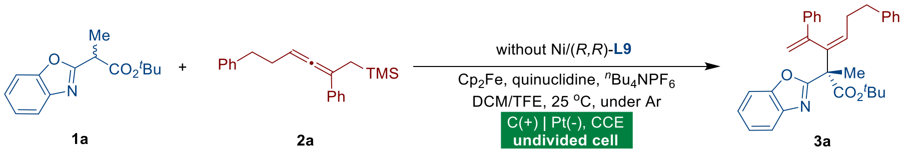

Entry	Time (h)	Yield (%)
1	0	0
2	0.5	4.5
3	1	10
4	1.5	16.5
5	2	22.6
6	2.5	26.5
7	3	31

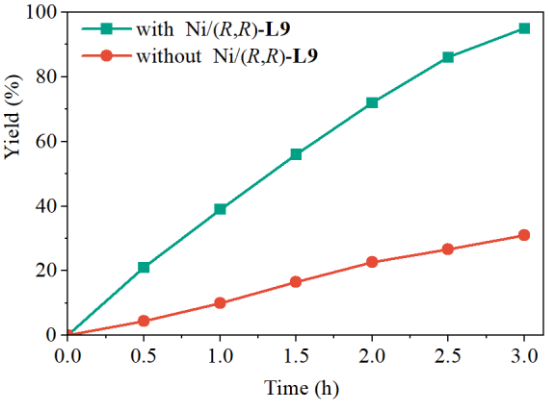

Figure S9. Kinetic studies to investigate the racemic background reaction. The investigations showed that the inclusion of a chiral nickel catalyst enhanced the catalytic efficiency of the process, although the improvement was not of significant magnitude.

9.3 Investigation of the catalyst loading

(a) Investigation of the catalyst loading under the standard condition

In a dried sealed tube, $Ni(OAc)_2 \cdot 4H_2O$ (9.92 mg, 0.04 mmol) and **L9** (14.4 mg, 0.04 mmol) were dissolved in DCM (2.0 mL) under N_2 atmosphere, and the mixture was stirred for 1 h at room temperature before use.

Ni/(*R,R*)-L9 (0.5 mol%): A 10 mL flask equipped with a magnetic stir bar was charged with **1a** (0.2 mmol), **2a** (0.6 mmol), $^{n}Bu_4NPF_6$ (0.225 mmol), quinuclidine (0.05 mmol), Cp_2Fe (0.001 mmol). The flask was equipped with a carbon rod (d = 6 mm) as the anode and a platinum plate (1.0 cm × 1.0 cm × 0.2 mm) as the cathode. The reaction mixture was degassed via vacuum evacuation and backfilled with argon three times, followed by the addition via a syringe of nickel catalyst solution made in advance (50 uL), DCM (3.0 mL), and TFE (1.5 mL). The constant current electrolysis was carried out at 25 ℃ until complete consumption of the substrate (monitored by TLC). The solution was diluted with ethyl acetate and washed with saturated NH_4Cl aqueous solution followed by distilled water washes. The aqueous phase was then extracted three times with ethyl acetate. The combined organic phase was dried over anhydrous $MgSO_4$ and concentrated under reduced pressure. The residue was purified by silica gel chromatography to afford the desired product **3a**.

Ni/(*R,R*)-L9 (1 mol%): A 10 mL flask equipped with a magnetic stir bar was charged with **1a** (0.2 mmol), **2a** (0.6 mmol), $^{n}Bu_4NPF_6$ (0.225 mmol), quinuclidine (0.05 mmol), Cp_2Fe (0.002 mmol). The flask was equipped with a carbon rod (d = 6 mm) as the anode and a platinum plate (1.0 cm × 1.0 cm × 0.2 mm) as the cathode. The reaction mixture was degassed via vacuum evacuation and backfilled with argon three times, followed by the addition via a syringe of nickel catalyst solution made in advance (100 uL), DCM (3.0 mL), and TFE (1.5 mL). The constant current electrolysis was carried out at 25 ℃ until complete consumption of the substrate (monitored by TLC). The solution was diluted with ethyl acetate and washed with saturated NH_4Cl aqueous solution followed by distilled water washes. The aqueous phase was then extracted three times with ethyl acetate. The combined organic phase was dried over anhydrous $MgSO_4$ and concentrated under reduced pressure. The residue was purified by silica gel chromatography to afford the desired product **3a**.

Ni/(*R,R*)-L9 (2 mol%): A 10 mL flask equipped with a magnetic stir bar was charged with **1a** (0.2 mmol), **2a** (0.6 mmol), $^{n}Bu_4NPF_6$ (0.225 mmol), quinuclidine (0.05 mmol), Cp_2Fe (0.004 mmol). The flask was equipped with a carbon rod (d = 6 mm) as the anode and a platinum plate (1.0 cm × 1.0 cm × 0.2 mm) as the cathode. The reaction mixture was degassed via vacuum evacuation and backfilled with argon three times, followed by the addition via a syringe of nickel catalyst solution made in advance (200 uL), DCM (3.0 mL), and TFE (1.5 mL). The constant current electrolysis was carried out at 25 ℃ until complete consumption of the substrate (monitored by TLC). The solution was diluted with ethyl acetate and washed with saturated NH_4Cl aqueous solution followed by distilled water washes. The aqueous phase was then extracted three times with ethyl acetate. The combined organic phase was dried over anhydrous $MgSO_4$ and concentrated under reduced pressure. The residue was purified by silica gel chromatography to afford the desired product **3a**.

Ni/(*R,R*)-L9 (5 mol%): A 10 mL flask equipped with a magnetic stir bar was charged with **1a** (0.2 mmol), **2a** (0.6 mmol), $^{n}Bu_4NPF_6$ (0.225 mmol), quinuclidine (0.05 mmol), Cp_2Fe (0.01 mmol). The flask was equipped with a carbon rod (d = 6 mm) as the anode and a platinum plate (1.0 cm × 1.0 cm × 0.2 mm) as the cathode. The reaction mixture was degassed via vacuum evacuation and backfilled with argon three times, followed by the addition via a syringe of nickel catalyst solution made in advance (0.5 mL), DCM (2.5 mL), and TFE (1.5 mL). The constant current electrolysis was carried out at 25 ℃ until

complete consumption of the substrate (monitored by TLC). The solution was diluted with ethyl acetate and washed with saturated NH_4Cl aqueous solution followed by distilled water washes. The aqueous phase was then extracted three times with ethyl acetate. The combined organic phase was dried over anhydrous $MgSO_4$ and concentrated under reduced pressure. The residue was purified by silica gel chromatography to afford the desired product **3a**.

Ni/(*R,R*)-L9 (10 mol%): A 10 mL flask equipped with a magnetic stir bar was charged with **1a** (0.2 mmol), **2a** (0.6 mmol), $^nBu_4NPF_6$ (0.225 mmol), quinuclidine (0.05 mmol), Cp_2Fe (0.02 mmol). The flask was equipped with a carbon rod (d = 6 mm) as the anode and a platinum plate (1.0 cm × 1.0 cm × 0.2 mm) as the cathode. The reaction mixture was degassed via vacuum evacuation and backfilled with argon three times, followed by the addition via a syringe of nickel catalyst solution made in advance (1.0 mL), DCM (2.0 mL), and TFE (1.5 mL). The constant current electrolysis was carried out at 25 °C until complete consumption of the substrate (monitored by TLC). The solution was diluted with ethyl acetate and washed with saturated NH_4Cl aqueous solution followed by distilled water washes. The aqueous phase was then extracted three times with ethyl acetate. The combined organic phase was dried over anhydrous $MgSO_4$ and concentrated under reduced pressure. The residue was purified by silica gel chromatography to afford the desired product **3a**.

Table S20**:** Investigation of the catalyst loading under the standard condition

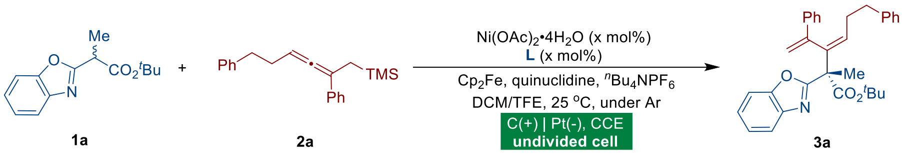

Entry	Ni/(*R*,*R*)-**L9** (mol %)	Yield (%)	e.e. (%) of **3a**
1	0.5	21	93
2	1	73	94
3	2	78	95
4	5	84	96
5	10	91	96

(b) Investigation of the catalyst loading with Ag_2O instead of electric current under the standard condition

In a dried sealed tube, $Ni(OAc)_2·4H_2O$ (4.96 mg, 0.02 mmol) and **L9** (7.2 mg, 0.02 mmol) were dissolved in DCM (2.0 mL) under N_2 atmosphere, and the mixture was stirred for 1 h at room temperature before use.

Ni/(*R,R*)-L9 (0.5 mol%): A 10 mL flask equipped with a magnetic stir bar was charged with **1a** (0.1 mmol), **2a** (0.3 mmol), $^nBu_4NPF_6$ (0.15 mmol), quinuclidine (0.05 mmol), Cp_2Fe (0.0005 mmol) and Ag_2O (0.3 mmol). The reaction mixture was degassed via vacuum evacuation and backfilled with argon three times, followed by the addition via a syringe of nickel catalyst solution made in advance (50 uL), DCM (2.0 mL), and TFE (1.0 mL). The mixture was stirred at 25 °C until complete consumption of the substrate (monitored by TLC). The solution was diluted with ethyl acetate and washed with saturated NH_4Cl aqueous solution followed by distilled water washes. The aqueous phase was then extracted three times with ethyl acetate. The combined organic phase was dried over anhydrous $MgSO_4$ and concentrated under reduced pressure. The residue was purified by silica gel chromatography to afford the desired product **3a**.

Ni/(*R,R*)-L9 (1 mol%): A 10 mL flask equipped with a magnetic stir bar was charged with **1a** (0.1

mmol), **2a** (0.3 mmol), ^{n}Bu$_4$NPF$_6$ (0.15 mmol), quinuclidine (0.05 mmol), Cp_2Fe (0.001 mmol) and Ag_2O (0.3 mmol). The reaction mixture was degassed via vacuum evacuation and backfilled with argon three times, followed by the addition via a syringe of nickel catalyst solution made in advance (100 uL), DCM (2.0 mL), and TFE (1.0 mL). The mixture was stirred at 25 °C until complete consumption of the substrate (monitored by TLC). The solution was diluted with ethyl acetate and washed with saturated NH_4Cl aqueous solution followed by distilled water washes. The aqueous phase was then extracted three times with ethyl acetate. The combined organic phase was dried over anhydrous $MgSO_4$ and concentrated under reduced pressure. The residue was purified by silica gel chromatography to afford the desired product **3a**.

Ni/(*R*,*R*)-L9 (2 mol%): A 10 mL flask equipped with a magnetic stir bar was charged with **1a** (0.1 mmol), **2a** (0.3 mmol), ^{n}Bu$_4$NPF$_6$ (0.15 mmol), quinuclidine (0.05 mmol), Cp_2Fe (0.002 mmol) and Ag_2O (0.3 mmol). The reaction mixture was degassed via vacuum evacuation and backfilled with argon three times, followed by the addition via a syringe of nickel catalyst solution made in advance (200 uL), DCM (2.0 mL), and TFE (1.0 mL). The mixture was stirred at 25 °C until complete consumption of the substrate (monitored by TLC). The solution was diluted with ethyl acetate and washed with saturated NH_4Cl aqueous solution followed by distilled water washes. The aqueous phase was then extracted three times with ethyl acetate. The combined organic phase was dried over anhydrous $MgSO_4$ and concentrated under reduced pressure. The residue was purified by silica gel chromatography to afford the desired product **3a**.

Ni/(*R*,*R*)-L9 (5 mol%): A 10 mL flask equipped with a magnetic stir bar was charged with **1a** (0.1 mmol), **2a** (0.3 mmol), ^{n}Bu$_4$NPF$_6$ (0.15 mmol), quinuclidine (0.05 mmol), Cp_2Fe (0.005 mmol) and Ag_2O (0.3 mmol). The reaction mixture was degassed via vacuum evacuation and backfilled with argon three times, followed by the addition via a syringe of nickel catalyst solution made in advance (0.5 mL), DCM (1.5 mL), and TFE (1.0 mL). The mixture was stirred at 25 °C until complete consumption of the substrate (monitored by TLC). The solution was diluted with ethyl acetate and washed with saturated NH_4Cl aqueous solution followed by distilled water washes. The aqueous phase was then extracted three times with ethyl acetate. The combined organic phase was dried over anhydrous $MgSO_4$ and concentrated under reduced pressure. The residue was purified by silica gel chromatography to afford the desired product **3a**.

Ni/(*R*,*R*)-L9 (10 mol%): A 10 mL flask equipped with a magnetic stir bar was charged with **1a** (0.1 mmol), **2a** (0.3 mmol), ^{n}Bu$_4$NPF$_6$ (0.15 mmol), quinuclidine (0.05 mmol), Cp_2Fe (0.01 mmol) and Ag_2O (0.3 mmol). The reaction mixture was degassed via vacuum evacuation and backfilled with argon three times, followed by the addition via a syringe of nickel catalyst solution made in advance (1.0 mL), DCM (1.0 mL), and TFE (1.0 mL). The mixture was stirred at 25 °C until complete consumption of the substrate (monitored by TLC). The solution was diluted with ethyl acetate and washed with saturated NH_4Cl aqueous solution followed by distilled water washes. The aqueous phase was then extracted three times with ethyl acetate. The combined organic phase was dried over anhydrous $MgSO_4$ and concentrated under reduced pressure. The residue was purified by silica gel chromatography to afford the desired product **3a**.

***Table S21*:** Investigation of the catalyst loading with Ag_2O instead of electric current under the standard condition

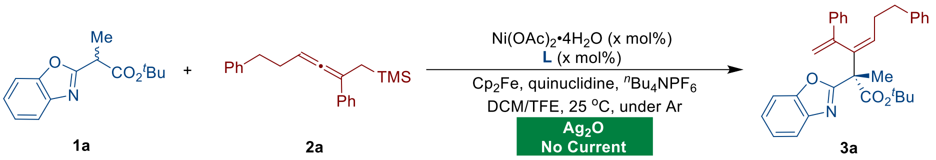

Entry	Ni/(*R*,*R*)-**L9** (mol %)	Yield (%)	e.e. (%) of **3a**
1	0.5	30	11
2	1	38	32
3	2	41	49
4	5	52	79
5	10	60	87

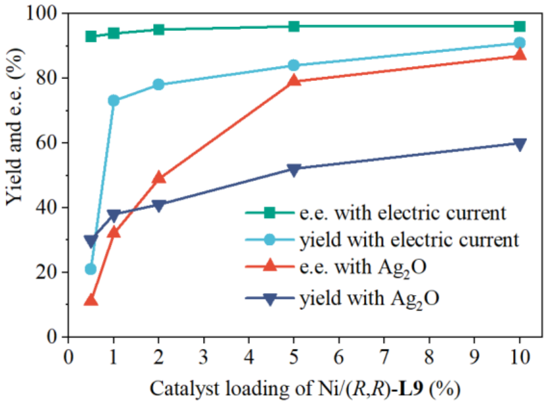

Figure S10. Investigation of the catalyst loading. Catalyst loadings varying from 0.5 mol% to 10 mol% produced coupling adduct **3a** with a similar enantiomeric excess in each case (green line). The utilization of silver oxide as a chemical oxidant instead of electrical input revealed a noticeable decline in the enantiomeric excess of the coupling adduct **3a** as the catalyst loading decreased from 10 mol% to 0.5 mol% (red line). In comparison to conventional processes that employ stoichiometric chemical oxidants, the tunable oxidation potential and current density yielded by our anodic oxidative coupling methodology offer the most prominent advantages.

9.4 Constant potential experiments

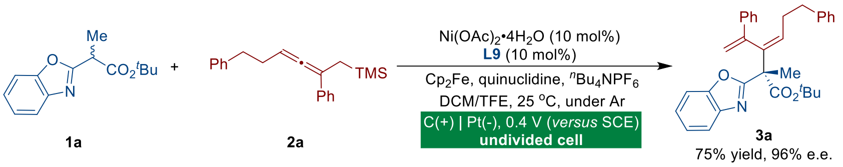

In a dried sealed tube, $Ni(OAc)_2{\cdot}4H_2O$ (2.48 mg, 0.01 mmol) and **L9** (3.6 mg, 0.01 mmol) were dissolved in DCM (1.0 mL) under N_2 atmosphere, and the mixture was stirred for 1 h at room temperature before use. A 10 mL flask equipped with a magnetic stir bar was charged with **1a** (0.1 mmol), **2a** (0.3 mmol), $^nBu_4NPF_6$ (0.15 mmol), quinuclidine (0.05 mmol), Cp_2Fe (0.01 mmol). The flask was equipped with a carbon rod (d = 6 mm) as the anode, a platinum plate (1.0 cm × 1.0 cm × 0.2 mm) as the cathode, and a saturated calomel electrode (SCE) as the reference electrode. The reaction mixture was degassed via vacuum evacuation and backfilled with argon three times, followed by the addition via a syringe of nickel catalyst solution made in advance, DCM (1.0 mL) and TFE (1.0 mL). The constant potential +0.4 V (*versus* SCE) electrolysis was carried out at 25 °C until complete consumption of the substrate (monitored by TLC). The solution was diluted with ethyl acetate and washed with saturated NH_4Cl aqueous solution followed by distilled water washes. The aqueous phase was then extracted three times

with ethyl acetate. The combined organic phase was dried over anhydrous $MgSO_4$ and concentrated under reduced pressure. The residue was purified by silica gel chromatography to afford the desired product **3a** (75% yield, 96% e.e.).

9.5 Radical clock experiment

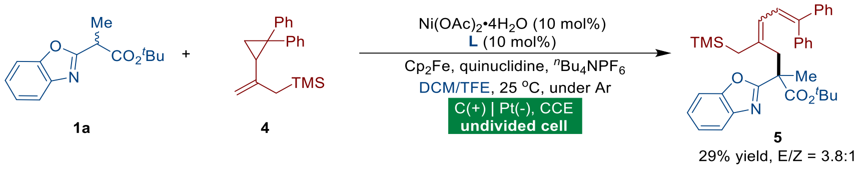

In a dried sealed tube, $Ni(OAc)_2 \cdot 4H_2O$ (2.48 mg, 0.01 mmol) and **L9** (3.6 mg, 0.01 mmol) were dissolved in DCM (1.0 mL) under N_2 atmosphere, and the mixture was stirred for 1 h at room temperature before use. A 10 mL flask equipped with a magnetic stir bar was charged with **1a** (0.1 mmol), **4** (0.15 mmol), $^nBu_4NPF_6$ (0.15 mmol), quinuclidine (0.05 mmol), Cp_2Fe (0.01 mmol). The flask was equipped with a carbon rod (d = 6 mm) as the anode and a platinum plate (1.0 cm × 1.0 cm × 0.2 mm) as the cathode. The reaction mixture was degassed via vacuum evacuation and backfilled with argon three times, followed by the addition via a syringe of nickel catalyst solution made in advance, DCM (1.0 mL) and TFE (1.0 mL). The constant current (2.0 mA) electrolysis was carried out at 25 °C until complete consumption of the substrate (monitored by TLC). The solution was diluted with ethyl acetate and washed with saturated NH_4Cl aqueous solution followed by distilled water washes. The aqueous phase was then extracted three times with ethyl acetate. The combined organic phase was dried over anhydrous $MgSO_4$ and concentrated under reduced pressure. The residue was purified by silica gel chromatography to afford the desired product **5** (29% yield, E/Z = 3.8:1).

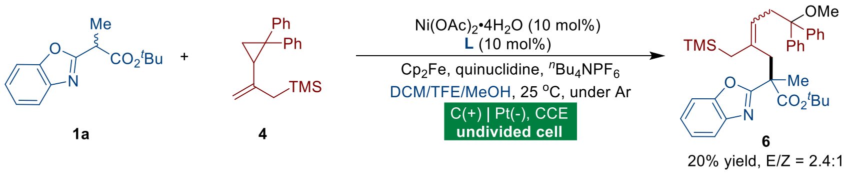

In a dried sealed tube, $Ni(OAc)_2 \cdot 4H_2O$ (2.5 mg, 0.01 mmol) and **L9** (3.6 mg, 0.01 mmol) were dissolved in DCM (1.0 mL) under N_2 atmosphere, and the mixture was stirred for 1 h at room temperature before use. A 10 mL flask equipped with a magnetic stir bar was charged with **1a** (0.1 mmol), **4** (0.15 mmol), $^nBu_4NPF_6$ (0.15 mmol), quinuclidine (0.05 mmol), Cp_2Fe (0.01 mmol). The flask was equipped with a carbon rod (d = 6 mm) as the anode and a platinum plate (1.0 cm × 1.0 cm × 0.2 mm) as the cathode. The reaction mixture was degassed via vacuum evacuation and backfilled with argon three times, followed by the addition via a syringe of nickel catalyst solution made in advance, DCM (0.5 mL), TFE (1.0 mL), and MeOH (0.5 mL). The constant current (2.0 mA) electrolysis was carried out at 25 °C until complete consumption of the substrate (monitored by TLC). The solution was diluted with ethyl acetate and washed with saturated NH_4Cl aqueous solution followed by distilled water washes. The aqueous phase was then extracted three times with ethyl acetate. The combined organic phase was dried over anhydrous $MgSO_4$ and concentrated under reduced pressure. The residue was purified by silica gel chromatography to afford the desired product **6** (20% yield, E/Z = 2.4:1).

Tert-butyl 2-(benzo[d]oxazol-2-yl)-2-methyl-7,7-diphenyl-4-((trimethylsilyl)methyl)hepta-4,6-dienoate (5) (E/Z = 3.8:1)

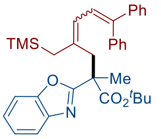

Major isomer: **^{1}H NMR (600 MHz, $CDCl_3$)** (major) δ 7.70 – 7.68 (m, 1H), 7.33 – 7.30 (m, 3H), 7.29 – 7.26 (m, 2H), 7.24 – 7.18 (m, 4H), 7.12 – 7.09 (m, 2H), 7.07 – 7.04 (m, 2H), 6.72 (d, *J* = 11.4 Hz, 1H), 5.82 (d, *J* = 11.4 Hz, 1H), 3.26 (d, *J* = 13.8 Hz, 1H), 3.19 (d, *J* = 13.7 Hz, 1H), 1.78 (s, 3H), 1.40 (s, 9H), 1.36 (d, *J* = 13.3 Hz, 1H), 1.08 (d, *J* = 13.3 Hz, 1H), -0.08 (s, 9H). **^{13}C NMR (150 MHz, $CDCl_3$)** (major) δ 171.92, 167.31, 150.78, 142.80, 140.96, 140.20, 139.61, 137.78, 130.76, 128.13, 128.06, 127.52, 127.05, 126.87, 126.42, 125.07, 124.23, 124.05, 120.06, 110.46, 82.38, 49.93, 38.37, 29.41, 27.95, 21.53, -1.16. **ESI-MS**: calculated $[C_{35}H_{41}NO_3Si + H]^+$: 552.2928, found: 552.2928.

Tert-butyl 2-(benzo[d]oxazol-2-yl)-7-methoxy-2-methyl-7,7-diphenyl-4-((trimethylsilyl)methyl)-hept-4-enoate (6) (E/Z = 2.4:1)

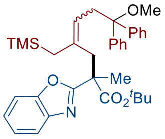

Major isomer: **^{1}H NMR (400 MHz, $CDCl_3$)** δ 7.80 – 7.73 (m, 1H), 7.58 – 7.52 (m, 1H), 7.40 – 7.33 (m, 2H), 7.25 – 7.18 (m, 8H), 7.18 – 7.12 (m, 2H), 5.01 (t, *J* = 6.3 Hz, 1H), 2.98 (s, 3H), 2.91 (dd, *J* = 26.6, 13.9 Hz, 2H), 2.85 (dd, *J* = 15.7, 7.5 Hz, 1H), 2.70 (dd, *J* = 15.6, 6.0 Hz, 1H), 1.69 (s, 3H), 1.40 (s, 9H), 1.20 (d, *J* = 13.8 Hz, 1H), 0.91 (d, *J* = 13.7 Hz, 1H), -0.17 (s, 9H). **^{13}C NMR (100 MHz, $CDCl_3$)** δ 172.08, 167.80, 150.87, 145.52, 145.46, 141.15, 133.56, 128.02, 127.96, 126.93, 126.89, 126.67, 126.60, 125.12, 124.48, 122.20, 120.28, 110.60, 82.24, 82.13, 50.15, 49.76, 38.50, 33.92, 27.94, 27.82, 20.96, -1.43. **ESI-MS**: calculated $[C_{36}H_{45}NO_4Si + Na]^+$: 606.3010, found: 606.3014.

9.6 Tracking and monitoring experiment of TMS-OCH_2CF_3

In a dried sealed tube, $Ni(OAc)_2·4H_2O$ (12.4 mg, 0.05 mmol) and **L9** (18.0 mg, 0.05 mmol) were dissolved in CD_2Cl_2 (3.0 mL) under N_2 atmosphere, and the mixture was stirred for 1 h at room temperature before use. A 30 mL flask equipped with a magnetic stir bar was charged with **1a** (124.5 mg, 0.5 mmol), **2a** (455 mg, 1.5 mmol), ^{n}Bu_4NPF_6 (290 mg, 0.75 mmol), quinuclidine (28 mg, 0.25 mmol), Cp_2Fe (9.3 mg, 0.05 mmol) and 1,1,2,2-Tetrachloroethane (42 mg, 0.25 mmol, as an internal standard). The flask was equipped with a carbon plate (2.0 cm × 2.0 cm × 2.0 mm) as the anode and a platinum plate (2.0 cm × 2.0 cm × 0.2 mm) as the cathode. The reaction mixture was degassed via vacuum evacuation and backfilled with argon three times, followed by the addition via a syringe of nickel catalyst solution made in advance, CD_2Cl_2 (7.0 mL) and TFE (5.0 mL). The constant current (8.0 mA) electrolysis was carried out at 25 °C. A reaction solution (0.5 mL) was taken every half an hour and analyzed by ^{1}H NMR spectroscopy. Yields determined by ^{1}H NMR using 1,1,2,2-Tetrachloroethane as an internal standard.

^{1}H NMR (400 MHz, CD_2Cl_2:TFE = 2:1) of reaction mixture

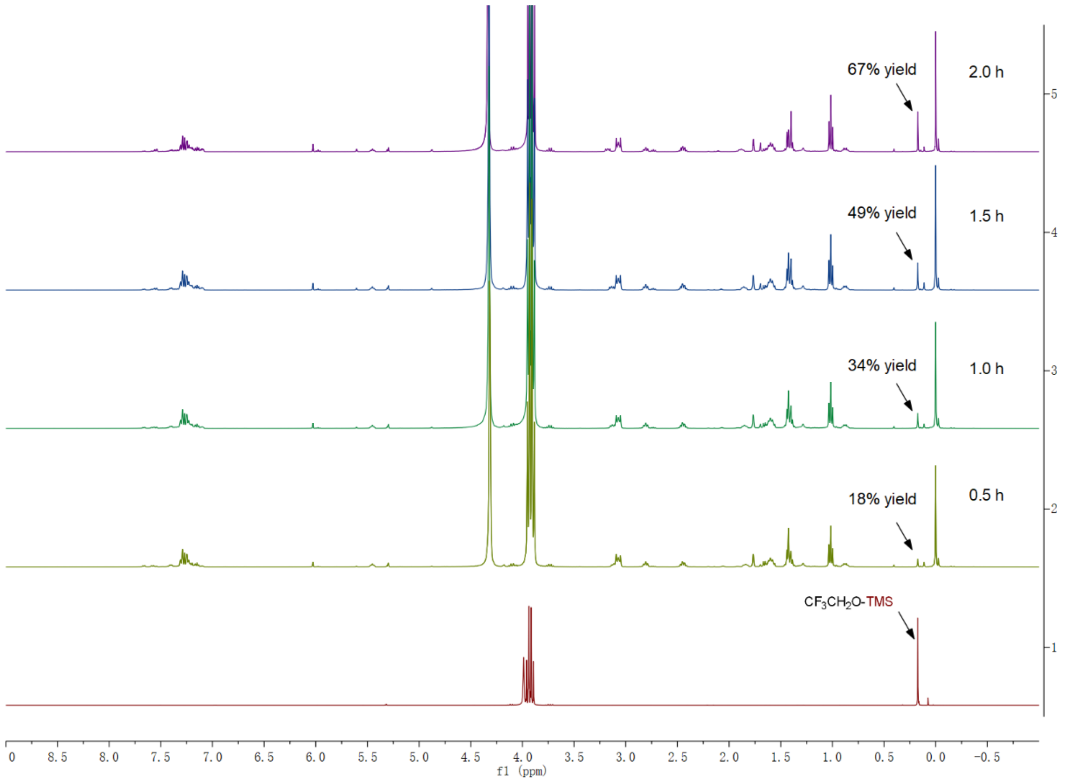

^{1}H NMR spectrum of **TMS-OCH$_2$CF$_3$** (after purification)

^{1}H NMR (400 MHz, CD$_2$Cl$_2$) δ 3.93 (q, *J* = 8.8 Hz, 1H), 0.17 (s, 1H).

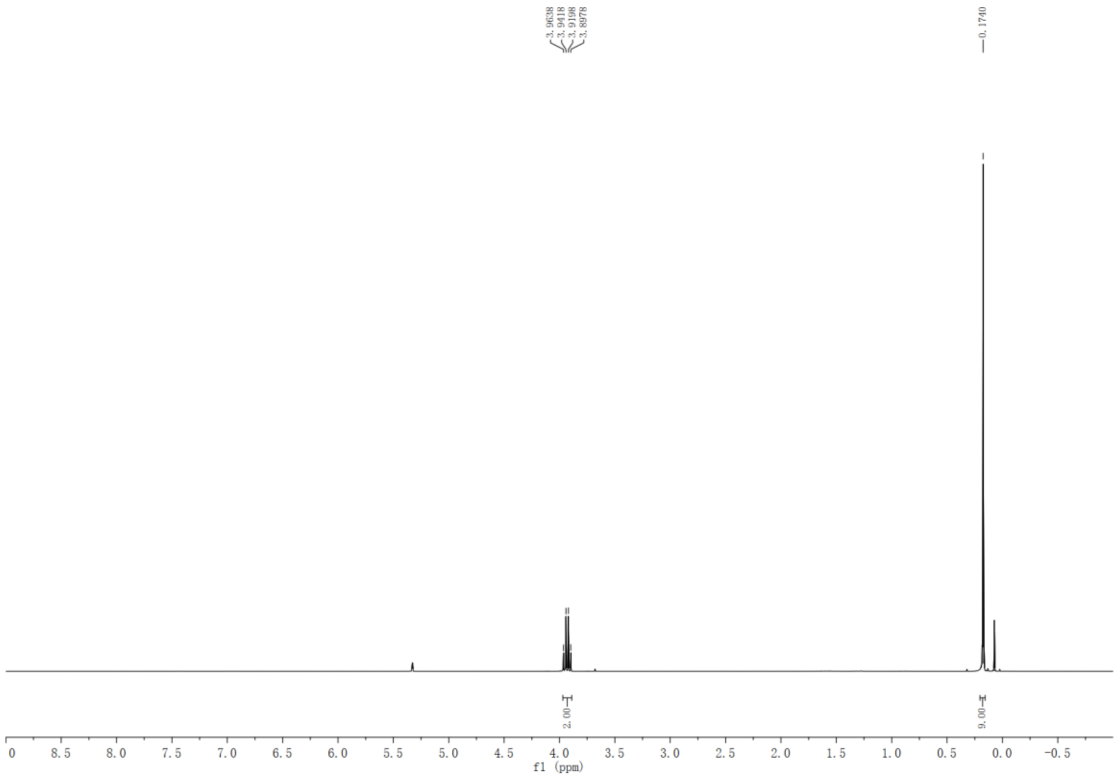

High resolution mass spectrometry (HRMS) of reaction mixture

EI-MS: calculated $[C_5H_{11}F_3OSi]^+$: 172.0526, found: 172.0518

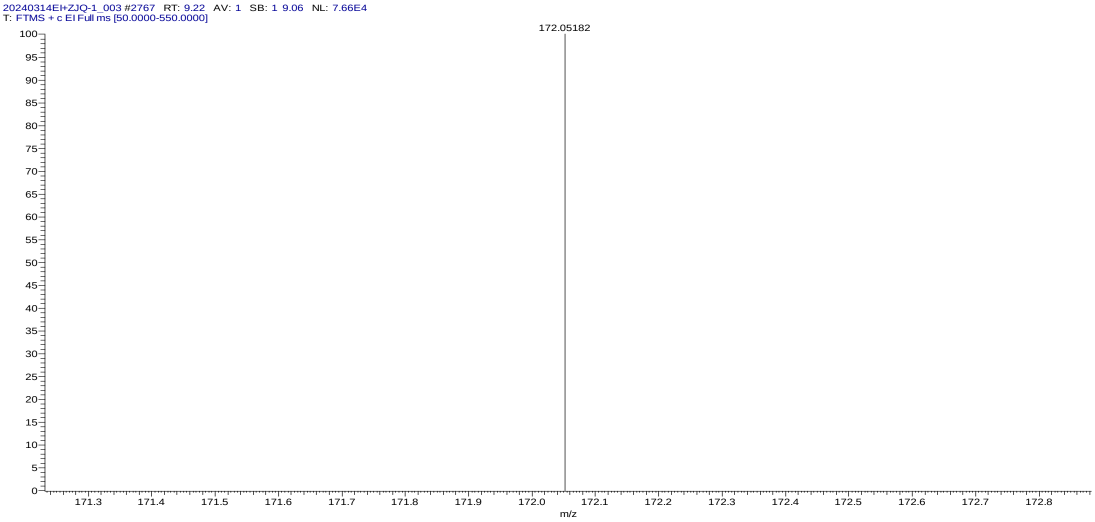

10. Computational Study

We investigated the process of removing TMS from the radical intermediate through DFT calculations. We considered two possible reaction pathways: Path 1) TMS leaves as a radical and forms the product, followed by TMS radical undergoing electrode oxidation and combining with TFE (red pathway). Path 2) The radical intermediate undergoes oxidation to form a cation, followed by nucleophilic attack of TFE on TMS to generate the product (black pathway). The process through Path 1 is thermodynamically unfavorable (ΔG = 25.4 kcal/mol), suggesting that Path 1 is unlikely to occur at room temperature. In Path 2, we calculated the process of oxidation of the radical intermediate at a potential of 0.4 V (versus SCE) and found that this process is energetically favorable (ΔG = -17.6 kcal/mol). Subsequently, the nucleophilic attack of TFE on TMS has a low energy barrier (ΔG =12.4 kcal/mol) and is relatively facile. Therefore, we suggested that the radical intermediate is initially oxidized to a cation and then undergoes TMS removal to yield the target product.

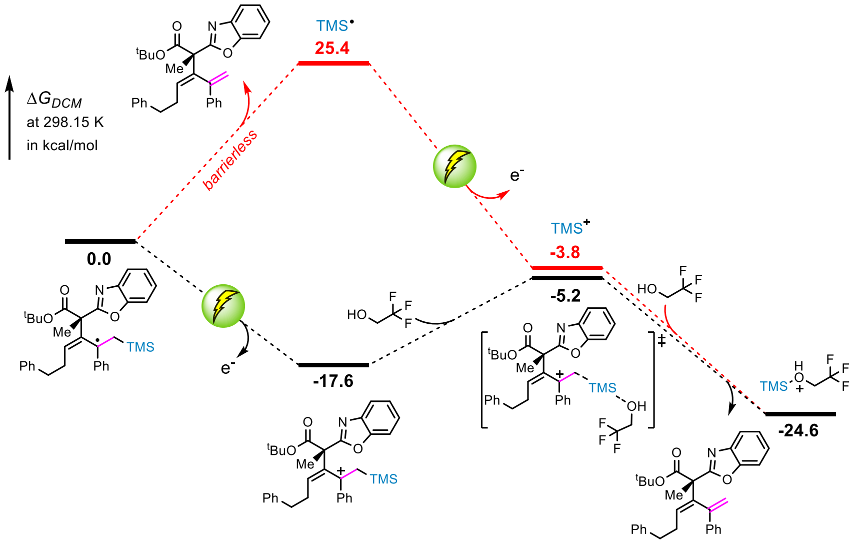

Figure S11. Gibbs free energy profiles of TMS leaving. Computed at the M06-2X-D3(zero)/def2-TZVP/SMD(DCM)//B3LYP-D3(BJ)/def2-SVP/IEFPCM(DCM) level. Paths with an electricity icon refers to an electrochemical process under the external potential of 0.4 V(versus SCE). The 'barrierless' refers to the absence of electronic barrier that makes the transition state coordinates difficult to locate.

All calculations were performed with Gaussian 16, Rev. C01 package[15]. The DFT functional of B3LYP[16], associated with the Grimme's dispersion correction D3(BJ)[17] and def2-SVP basis set[18] were used for geometry optimization of all structures. The solvent effects were taken into account in geometry optimizations by employing the IEFPCM solvation model (solvent = dichloromethane)[19]. Harmonic frequency calculations at the same level were performed to validate that the optimized structures are local minima or transition states. The Gibbs free energy were calculated using Grimme's QRRHO[20] by Shermo (Version 2.5)[21]. Single-point energy calculations were conducted with the M06-2X functional[22] and def2-TZVP basis set[18], including dispersion correction D3(zero)[23] and SMD solvation model[24] (solvent = dichloromethane).

Gibbs free energy of electrochemical processes were calculated according to suggestions from Konezny, S.J. et al[25]. We use ferrocene/ferrocenium as a link between experiments and calculations. The potentials are obtained according to the following equations:

$$E_{calc}(vs. Fc^{+}/Fc) = E_{calc}(abs) - E_{calc,Fc^{+}/Fc}(abs)$$

$$E_{exp}(vs.Fc^{+}/Fc) = E_{exp}(vs.SCE) - E_{exp,Fc^{+}/Fc}(vs.SCE)$$

Thus, the calculated and experimental potentials are comparable. Next, the Gibbs free energy of electrochemical processes can be calculated using:

$$\Delta G = -nFE = -nF\left[E_{exp}^{external}(vs.Fc^{+}/Fc) - E_{calc}(vs.Fc^{+}/Fc)\right]$$

where $E_{exp}^{external}$ is the external potential used in our reaction systems.

Optimized Coordinates

Radical intermediate

C	-3.63341500	1.85751600	1.68880600
C	-2.71148800	1.96399000	0.65585300
C	-3.02263700	2.47312000	-0.61700000
C	-4.32495400	2.89667500	-0.90292900
C	-5.27248300	2.78918600	0.11907500
C	-4.93338900	2.28177400	1.38947800
H	-3.36519400	1.46473000	2.66963000
H	-4.58347500	3.29023800	-1.88728800
H	-6.30083000	3.10616100	-0.06749900
H	-5.70378700	2.21655600	2.16075300
N	-1.86591200	2.43360500	-1.39685200
O	-1.38729700	1.61856100	0.63621400
C	-0.95397700	1.93396800	-0.62487600
C	0.45767100	1.57342500	-0.99367200
C	0.77020600	2.13304400	-2.40292600
H	1.76803900	1.80067500	-2.71203500
H	0.02496700	1.79269300	-3.12989500
H	0.74251200	3.23254200	-2.38941500
C	1.45038900	2.28124900	-0.05076700
O	1.13219300	3.12205100	0.75998900
O	2.68697600	1.88896800	-0.32815700
C	3.89031400	2.43840500	0.32211600
C	5.02031300	1.66946800	-0.35693200
H	5.02237500	1.86194000	-1.44005300
H	5.98911700	1.98579000	0.05688300
H	4.89989700	0.58856500	-0.19677400
C	3.98237500	3.93475600	0.02727600
H	4.94540600	4.32050900	0.39412800
H	3.93227800	4.11331400	-1.05771000
H	3.17178500	4.48849000	0.51711600
C	3.85376900	2.13993300	1.81951200
H	2.99726900	2.62592000	2.30298900
H	3.79748900	1.05567600	1.99158300
H	4.77892600	2.51249300	2.28419600
C	0.59764400	0.01794700	-0.97136300
C	-0.08141500	-0.67645400	-1.90644100
H	-0.65228600	-0.10832400	-2.64714700
C	-0.16142500	-2.16522200	-2.05680000
H	0.61899900	-2.50334200	-2.76384900
H	0.06647300	-2.65844300	-1.10034000
C	-1.52627600	-2.63931700	-2.59575600
H	-1.46095600	-3.71677900	-2.81147100
H	-1.71528100	-2.13564300	-3.55890600
C	-2.68722700	-2.38771300	-1.66075600
C	-3.15353800	-3.40643800	-0.81574400

C	-3.31471000	-1.13176300	-1.59485300
C	-4.21697200	-3.18396300	0.06349400
H	-2.67559300	-4.38950800	-0.85065700
C	-4.37399000	-0.90244600	-0.71283200
H	-2.97568900	-0.32013900	-2.24226800
C	-4.83037000	-1.92951100	0.11844000
H	-4.56431400	-3.99315500	0.71088300
H	-4.84088500	0.08333400	-0.67259400
H	-5.65791300	-1.74952400	0.80877100
C	1.44899500	-0.60724300	0.05855000
C	1.12467800	-0.37403800	1.49534000
C	2.62367100	-1.34432500	-0.32128200
C	3.06547000	-1.40921300	-1.67426200
C	3.45239300	-1.97813400	0.64914200
C	4.23818700	-2.06565600	-2.02641100
H	2.48204600	-0.90520500	-2.44400300
C	4.62534100	-2.63414300	0.28854600
H	3.17158800	-1.95236900	1.70088300
C	5.03153900	-2.68882500	-1.05137100
H	4.54812200	-2.08488000	-3.07450300
H	5.23364200	-3.10857500	1.06295500
H	5.95373500	-3.20284000	-1.33127700
H	0.52093800	0.53586800	1.62556400
H	2.03172700	-0.24903000	2.10579400
Si	0.06587600	-1.73564800	2.34583500
C	-1.74185500	-1.44853500	1.93184300
H	-1.90494700	-1.36922600	0.84811300
H	-2.36889500	-2.27245500	2.30611400
H	-2.09498700	-0.51434100	2.39234400
C	0.33787500	-1.53691300	4.20424300
H	-0.28463000	-2.24809300	4.77229700
H	1.39172100	-1.71858200	4.47256300
H	0.07368500	-0.51792900	4.53223300
C	0.57736100	-3.47472100	1.82244900
H	-0.12361300	-4.20736100	2.25597900
H	0.55500800	-3.59253500	0.72841900
H	1.59124600	-3.72933100	2.16626800

Cation intermediate

C	-3.70492300	1.86102300	1.65739600
C	-2.78685200	1.94610200	0.61930500
C	-3.11892700	2.35696100	-0.68299300
C	-4.43875800	2.69774400	-0.99726500
C	-5.38207100	2.60952900	0.02934600
C	-5.02204200	2.20170800	1.32986400
H	-3.42394000	1.54534000	2.66162100
H	-4.71202500	3.01450000	-2.00480100
H	-6.42395300	2.86344900	-0.17631100
H	-5.79065700	2.14957500	2.10367100
N	-1.96019400	2.32158900	-1.46152100
O	-1.44354100	1.66162900	0.61885000
C	-1.02952000	1.91891000	-0.66072000
C	0.40131800	1.61946300	-1.00949900
C	0.71572900	2.20142800	-2.41026200
H	1.73491600	1.92272000	-2.70436600
H	-0.00062800	1.83893600	-3.15544900
H	0.63443700	3.29677200	-2.38022700

C	1.35829500	2.37007100	-0.06090100
O	1.01970200	3.29756600	0.63256100
O	2.58989000	1.89270800	-0.20414200
C	3.78697700	2.50268600	0.42620000
C	4.92193000	1.61664600	-0.07609900
H	4.96563600	1.62591400	-1.17477300
H	5.88046000	1.98561700	0.31620700
H	4.78451900	0.57895900	0.25935000
C	3.93283600	3.93425400	-0.08306500
H	4.88619700	4.34946400	0.27570800
H	3.94427600	3.94998800	-1.18329000
H	3.11614800	4.57308600	0.27563300
C	3.66142600	2.42957600	1.94573900
H	2.80341500	3.01036000	2.30543500
H	3.55613700	1.38591500	2.27648100
H	4.57664900	2.83590200	2.40083600
C	0.62925900	0.07985100	-1.01190600
C	0.03710600	-0.65075500	-1.98162900
H	-0.57461700	-0.08845600	-2.69399100
C	0.02794500	-2.12653900	-2.21366600
H	0.72089900	-2.33771800	-3.04810000
H	0.41092100	-2.68115400	-1.34771900
C	-1.36595300	-2.64296800	-2.63379100
H	-1.27098400	-3.71224400	-2.87416100
H	-1.65362600	-2.13424000	-3.56825100
C	-2.45844600	-2.45098100	-1.60529000
C	-2.79935700	-3.48678300	-0.72166400
C	-3.16941800	-1.24174400	-1.51829800
C	-3.82650400	-3.32710100	0.21309200
H	-2.26513100	-4.43890500	-0.78046500
C	-4.19677900	-1.07751700	-0.58480300
H	-2.93418900	-0.42109600	-2.20004300
C	-4.53106200	-2.12240500	0.28137600
H	-4.07920000	-4.14935300	0.88686900
H	-4.73845200	-0.13144700	-0.53630400
H	-5.33456200	-1.99401100	1.01009400
C	1.45572800	-0.50863600	0.05406000
C	1.10154000	-0.27492500	1.42473500
C	2.62026600	-1.28908200	-0.29885900
C	3.12353600	-1.26400600	-1.62427000
C	3.34198200	-2.02122300	0.68024200
C	4.27574900	-1.96301700	-1.96069300
H	2.62249200	-0.65861900	-2.37677300
C	4.48813500	-2.72296300	0.33813000
H	3.00297800	-2.04773700	1.71418400
C	4.95679900	-2.69839200	-0.98407000
H	4.65558300	-1.92471300	-2.98286600
H	5.02612500	-3.28958500	1.09972100
H	5.86423500	-3.24565600	-1.24818400
H	0.42201100	0.57305300	1.57024300
H	1.96167700	-0.22101600	2.09994400
Si	0.01177600	-1.73664600	2.27902200
C	-1.76202400	-1.17166600	2.21386800
H	-2.09990100	-0.99075700	1.18522400
H	-2.40713500	-1.95212100	2.64675800
H	-1.89192900	-0.24950000	2.79780800
C	0.68176200	-1.74780200	4.03104800
H	0.08562900	-2.44685000	4.64067300

H	1.73248400	-2.07568200	4.06940800
H	0.60946800	-0.74774400	4.48684700
C	0.24555900	-3.39716000	1.44565100
H	-0.22471800	-4.15392600	2.09581500
H	-0.26762500	-3.43701700	0.47544400
H	1.29662300	-3.68503600	1.30642500

TS-Cation

C	1.36642600	3.75594900	-1.83719700
C	0.47955500	3.37696900	-0.83791700
C	0.61146500	3.75075200	0.51011600
C	1.69111600	4.54458100	0.91323800
C	2.60153300	4.93227800	-0.07287600
C	2.44223800	4.54843000	-1.42035000
H	1.23516600	3.45145300	-2.87616700
H	1.81138300	4.84176600	1.95616500
H	3.45867200	5.55018700	0.20282400
H	3.17680600	4.87753000	-2.15830300
N	-0.44254400	3.18042500	1.22397200
O	-0.63198800	2.58040400	-0.92745900
C	-1.12699300	2.51669000	0.34886600
C	-2.29457100	1.60510100	0.63643400
C	-3.08017200	2.17980600	1.84325600
H	-3.86573500	1.47493500	2.14233600
H	-2.41021100	2.37233100	2.68815600
H	-3.53999900	3.13691100	1.55962900
C	-3.31315700	1.62588800	-0.52088400
O	-3.39135300	2.52613700	-1.32143600
O	-4.13003000	0.58244000	-0.42880100
C	-5.28132700	0.38280700	-1.34144400
C	-5.88937500	-0.92834900	-0.85506600
H	-6.16736300	-0.85761500	0.20629800
H	-6.79352100	-1.15346600	-1.43905600
H	-5.18063700	-1.75905300	-0.97435400
C	-6.26172400	1.54042000	-1.16583200
H	-7.16647500	1.34109900	-1.75918500
H	-6.55778200	1.63315500	-0.10993200
H	-5.82573900	2.48968000	-1.50000800
C	-4.77684800	0.24949900	-2.77715700
H	-4.31869000	1.18114700	-3.13019800
H	-4.04075400	-0.56462500	-2.85148800
H	-5.62455700	0.00374600	-3.43394500
C	-1.75729500	0.17858000	0.95254200
C	-1.20927800	-0.05870000	2.16020400
H	-1.17610900	0.78361700	2.85641900
C	-0.63807600	-1.33219000	2.70866700
H	-1.45248600	-1.85788700	3.23892800
H	-0.31895500	-2.01307500	1.90991300
C	0.49883000	-1.11664400	3.72877500
H	0.69197500	-2.08023400	4.22349900
H	0.13906200	-0.42876900	4.51093200
C	1.79720400	-0.58997500	3.15985400
C	2.80404300	-1.47801500	2.74451900
C	2.03763400	0.78831200	3.04310000
C	4.01767200	-1.00577600	2.23209900
H	2.64616100	-2.55499600	2.85055200
C	3.24878400	1.26666600	2.53423700

H	1.26766900	1.50039500	3.34971800
C	4.24440600	0.37241700	2.12835200
H	4.80124300	-1.71038400	1.93997300
H	3.41167700	2.34311300	2.44848400
H	5.19610500	0.74526600	1.74285500
C	-1.83338200	-0.85695000	-0.11652600
C	-1.23896500	-0.62101700	-1.34269000
C	-2.57949600	-2.09481200	0.16299800
C	-3.42490000	-2.17741600	1.28894300
C	-2.53533900	-3.19939900	-0.71655600
C	-4.17681300	-3.32255400	1.53887600
H	-3.51779500	-1.31979800	1.95232600
C	-3.28484700	-4.34448400	-0.46479600
H	-1.90965600	-3.17170000	-1.60772400
C	-4.10695600	-4.41223400	0.66671400
H	-4.83099400	-3.35934200	2.41207600
H	-3.23063600	-5.18855000	-1.15495900
H	-4.69728700	-5.31022700	0.86115100
H	-0.91786800	0.39437700	-1.58584900
H	-1.50417400	-1.24855100	-2.19418900
Si	1.04679000	-1.27763400	-1.15425800
C	1.51736900	0.29699400	-0.28424700
H	0.71274400	0.67897100	0.35067000
H	2.38225800	0.13906000	0.36458700
H	1.77671000	1.06187000	-1.02912000
C	1.15885600	-1.37400100	-3.01908000
H	2.17247100	-1.67853000	-3.31954700
H	0.46052700	-2.13233900	-3.40375800
H	0.92158200	-0.40710900	-3.48665800
C	0.93748600	-2.91047200	-0.25453900
H	1.51019400	-3.65695400	-0.82545000
H	1.37701600	-2.82731300	0.74931100
H	-0.08875200	-3.28190900	-0.14641200
C	5.67940100	-1.53095700	-1.60463800
C	4.34218600	-0.82608200	-1.46712500
H	4.45853500	0.01710700	-0.76909100
H	4.04271500	-0.43749100	-2.44865100
O	3.35236000	-1.74264200	-1.03555100
H	3.55150500	-2.01163100	-0.12154500
F	6.63500400	-0.65965100	-1.94414300
F	5.64461900	-2.50018700	-2.52536800
F	6.02978700	-2.10079000	-0.43167100

Product

C	3.94211500	-1.33613900	1.63399400
C	3.01775800	-1.39325300	0.60034800
C	3.34511600	-1.71665600	-0.72704000
C	4.67456100	-1.98539100	-1.07166000
C	5.62627000	-1.92216800	-0.04994600
C	5.26731500	-1.60800500	1.27701900
H	3.65467500	-1.07871000	2.65365300
H	4.94983300	-2.23224100	-2.09838100
H	6.67451800	-2.12421600	-0.28083500
H	6.04328400	-1.57230800	2.04473900
N	2.17328200	-1.68556100	-1.48430800
O	1.67232700	-1.15587800	0.63399100
C	1.23844100	-1.36042000	-0.64894000

C	-0.22210000	-1.13656500	-0.95056000
C	-0.49328000	-1.54570900	-2.41597100
H	-1.53303100	-1.31446300	-2.67455900
H	0.18311200	-1.01938200	-3.09845200
H	-0.31892900	-2.62315500	-2.54759700
C	-1.07809400	-2.06907500	-0.06807100
O	-0.62041600	-2.88596500	0.69726500
O	-2.36714100	-1.87937400	-0.32901200
C	-3.44100800	-2.61519800	0.36644100
C	-4.71065500	-2.07131000	-0.28163400
H	-4.70333200	-2.26166400	-1.36537300
H	-5.59126200	-2.56620600	0.15351200
H	-4.79679800	-0.98839300	-0.11860000
C	-3.29521900	-4.11310600	0.09916800
H	-4.17452100	-4.63944800	0.50002900
H	-3.24657000	-4.30339900	-0.98400900
H	-2.39353100	-4.51957700	0.57193400
C	-3.40091500	-2.27507700	1.85532600
H	-2.47262200	-2.63043800	2.31957800
H	-3.47757400	-1.18776100	1.99925700
H	-4.25322200	-2.75431000	2.36013600
C	-0.57042500	0.35366800	-0.69189500
C	-0.17064500	1.28122300	-1.57613800
H	0.37294400	0.94871900	-2.46664900
C	-0.32374600	2.76650000	-1.45411500
H	-1.01060900	3.13331200	-2.23842800
H	-0.77296300	3.03660000	-0.48869000
C	1.03360100	3.48508700	-1.60938200
H	0.86759300	4.57150000	-1.52679900
H	1.43146000	3.29808600	-2.61992500
C	2.04117500	3.03307600	-0.57847400
C	1.93213500	3.45272200	0.75621500
C	3.06992700	2.14021900	-0.91486700
C	2.83162600	3.00100600	1.72552400
H	1.13211700	4.14302600	1.03871800
C	3.97462500	1.68862400	0.04947100
H	3.16120200	1.79145700	-1.94697200
C	3.85802200	2.11867900	1.37397700
H	2.73136100	3.34002800	2.75978900
H	4.76383100	0.98796300	-0.23059700
H	4.55988600	1.75937300	2.12972000
C	-1.31429600	0.72076800	0.55705600
C	-0.82778000	0.40555200	1.77248600
C	-2.62250900	1.41524900	0.39609400
C	-3.38049800	1.26070700	-0.77852900
C	-3.14201500	2.24020500	1.41222900
C	-4.61990900	1.88680800	-0.92398700
H	-2.99799300	0.62808600	-1.57877600
C	-4.38004700	2.86733500	1.26822400
H	-2.55864100	2.41147500	2.31859500
C	-5.12830000	2.69204700	0.09911500
H	-5.19318700	1.74134800	-1.84283400
H	-4.75765700	3.50739700	2.06939600
H	-6.09522500	3.18736300	-0.01629300
H	0.13430100	-0.09159500	1.88559200
H	-1.39106500	0.62176700	2.68247700

TMS-Radical

Si	-0.00022700	0.00039300	-0.43016100
C	1.40229400	1.12051400	0.17816800
H	2.38274800	0.76459300	-0.17679400
H	1.26736900	2.15773600	-0.16809800
H	1.42386400	1.13077900	1.28351200
C	-1.67201900	0.65327500	0.17824800
H	-1.85328500	1.68206300	-0.17202700
H	-2.50237500	0.01962500	-0.17252200
H	-1.69365900	0.66121800	1.28354500
C	0.26996300	-1.77416400	0.17793300
H	-0.53236900	-2.44463900	-0.16935100
H	1.23227300	-2.17805200	-0.17543900
H	0.27718000	-1.79658100	1.28333000

TMS-Cation

Si	-0.00093000	-0.00001900	-0.00320200
C	0.96973000	-1.55640400	-0.00298600
H	0.80341100	-2.06077700	0.96721100
H	0.58802900	-2.23539600	-0.78377300
H	2.04536500	-1.38074900	-0.14013200
C	0.86461400	1.61700400	0.00420800
H	1.41597600	1.71730000	-0.94871600
H	0.17594100	2.46518500	0.11815300
H	1.61866900	1.62515200	0.80981200
C	-1.83396900	-0.06114200	0.00058100
H	-2.20337500	0.45389500	0.90529000
H	-2.21361900	0.50796300	-0.86615100
H	-2.21963300	-1.08904400	-0.02768300

TFE

C	-0.41724800	0.01681700	0.00296200
C	0.90186500	0.76460000	-0.08813700
H	0.91671400	1.28265600	-1.06477800
H	0.90562400	1.52991100	0.70201400
O	1.99548200	-0.08408100	0.11396800
H	2.04970200	-0.69496900	-0.63503700
F	-1.45303700	0.83924800	-0.23262900
F	-0.60476500	-0.54328600	1.20748700
F	-0.46926500	-0.97745700	-0.90851300

TFE-TMS

Si	-1.75452800	0.04619600	0.10830600
C	-1.44722900	-0.97461500	1.62352000
H	-1.39752400	-2.05193300	1.40343100
H	-0.54067900	-0.65957500	2.16020200
H	-2.30490900	-0.81468600	2.29851000
C	-1.61806000	1.87972400	0.33420100
H	-0.68965300	2.14370700	0.86165400
H	-1.65476100	2.41694500	-0.62642900
H	-2.47095100	2.22146300	0.94414800
C	-3.10500100	-0.54344800	-1.01355000
H	-3.09013100	-0.01532800	-1.97963100
H	-3.03016000	-1.62688500	-1.19087500
H	-4.07613900	-0.33748100	-0.53346400

C	2.00877800	-0.01223500	-0.03335500
C	0.99580400	-1.02600400	-0.54305600
H	1.39559800	-1.56120700	-1.41237000
H	0.74204400	-1.74081700	0.24541000
O	-0.22161100	-0.35224600	-0.93445100
H	-0.13199400	0.09786900	-1.79654700
F	3.13909100	-0.63349300	0.29378000
F	1.54284600	0.63121200	1.05071200
F	2.27137300	0.90879400	-0.96907700

Fc

Fe	-0.00000900	0.00009100	-0.00029200
C	-1.67137500	-0.26482500	-1.18655500
C	-1.66977900	-1.21081900	-0.11495800
H	-1.64539200	-2.29469400	-0.21831200
C	-1.67064500	-0.48386500	1.11567700
H	-1.64724600	-0.91678700	2.11476700
C	-1.67166200	1.04682200	-0.61811800
H	-1.64845300	1.98431200	-1.17184800
C	-1.67067500	0.91131600	0.80479400
H	-1.64708700	1.72765000	1.52526300
C	1.67139700	0.48376900	-1.11546800
C	1.67098600	-0.91140700	-0.80454700
H	1.64783300	-1.72773700	-1.52503400
C	1.67109200	-1.04687400	0.61836500
H	1.64732600	-1.98435700	1.17208300
C	1.66992900	1.21075400	0.11515100
H	1.64579800	2.29464100	0.21843100
C	1.67070900	0.26478500	1.18676800
H	1.64791300	0.50162000	2.24958600
H	-1.64923700	-0.50161100	-2.24940000
H	1.64892800	0.91666700	-2.11459200

Fc+

Fe	0.00000500	-0.00055400	0.00010400
C	1.68845800	-1.15222100	-0.39078000
C	1.68787700	0.01552400	-1.21651100
H	1.64382500	0.02941900	-2.30404600
C	1.68789200	1.16189100	-0.36129400
H	1.64425800	2.20051100	-0.68403700
C	1.68839600	-0.72759600	0.97486100
H	1.64512700	-1.37793700	1.84665000
C	1.68821900	0.70245300	0.99314400
H	1.64553400	1.33044900	1.88121700
C	-1.68916600	-1.16147700	0.36116200
C	-1.68875900	-0.70199200	-0.99324300
H	-1.64657100	-1.33001400	-1.88132300
C	-1.68744300	0.72805800	-0.97491800
H	-1.64333200	1.37837700	-1.84668200
C	-1.68812800	-0.01516600	1.21643100
H	-1.64432500	-0.02917800	2.30397500
C	-1.68735200	1.15262600	0.39075600
H	-1.64348900	2.18257700	0.74019200
H	1.64556000	-2.18223300	-0.74015500
H	-1.64668100	-2.20015900	0.68386700

11. X-ray crystallography data

11.1 Characterize of 3f by X-ray crystallography

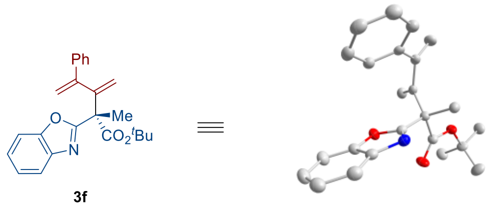

3f

3f
CCDC (**2268498**)

Chemical formula	$C_{24}H_{25}NO_3$
Formula weight	375.45
Space group	$P3_1$
Z	3
a, Å	17.0714(2)
b, Å	17.0714(2)
c, Å	6.21280(10)
α, °	90
β, °	90
γ, °	120
V, $Å^3$	1568.04(4)
Flack parameter	0.03(9)

11.2 Characterize of 8l by X-ray crystallography

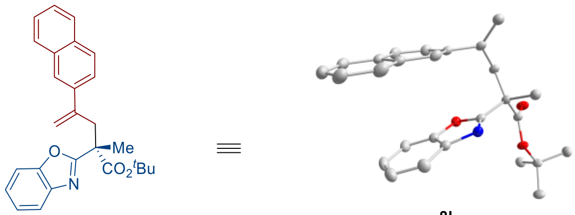

8l

8l
CCDC (**2268499**)

Chemical formula	$C_{27}H_{27}NO_3$
Formula weight	413.49
Space group	$P2_1$
Z	2
a, Å	9.67380(10)
b, Å	6.35940(10)
c, Å	18.1306(3)
α, °	90
β, °	99.6740(10)
γ, °	90
V, Å^3	1099.53(3)
Flack parameter	0.00(7)

11.3 Characterize of (*R*,*R*)-16 by X-ray crystallography

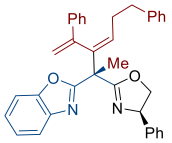

(*R*,*R*)-**16**

≡

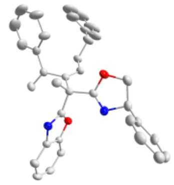

(*R*,*R*)-**16**
CCDC (**2343662**)

Chemical formula	$C_{36}H_{32}N_2O_2$
Formula weight	524.63
Space group	$P2_1$
Z	2
a, Å	9.1775(6)
b, Å	6.1626(4)
c, Å	25.7551(18)
α, °	90
β, °	92.233(3)
γ, °	90
V, Å^3	1455.53(17)
Flack parameter	0.05(13)

12. NMR spectra

^{1}H NMR spectrum of crude **3a**

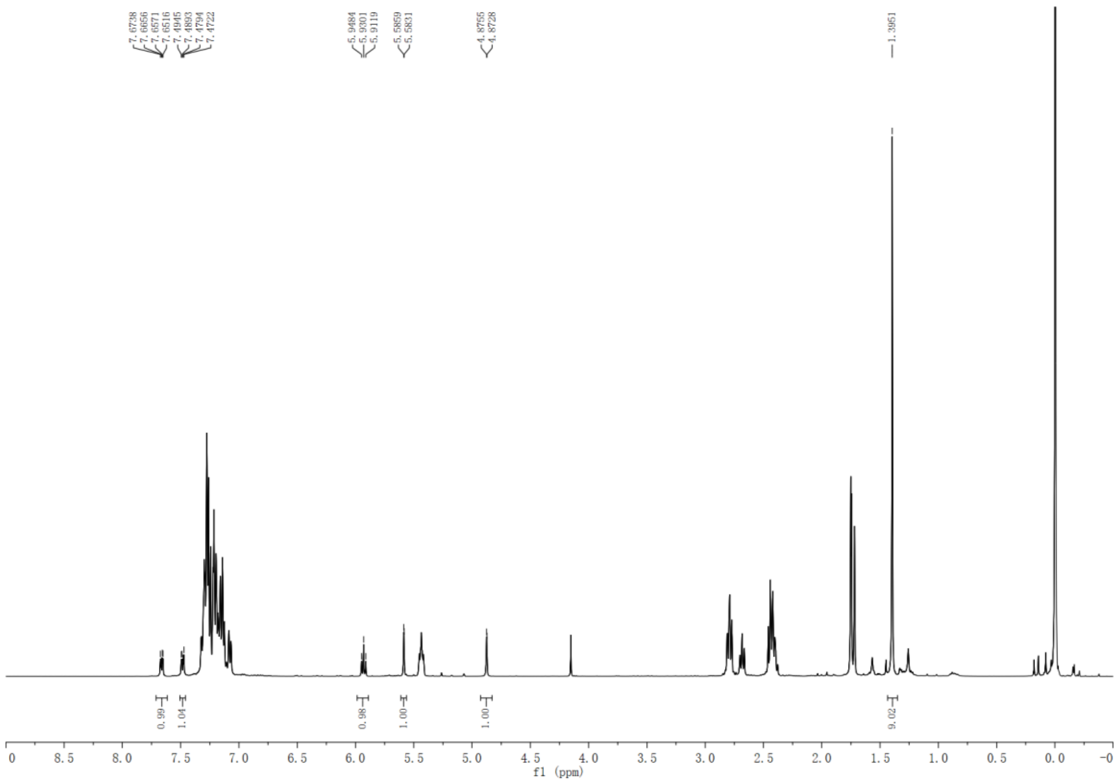

^{1}H NMR spectrum of **3a**

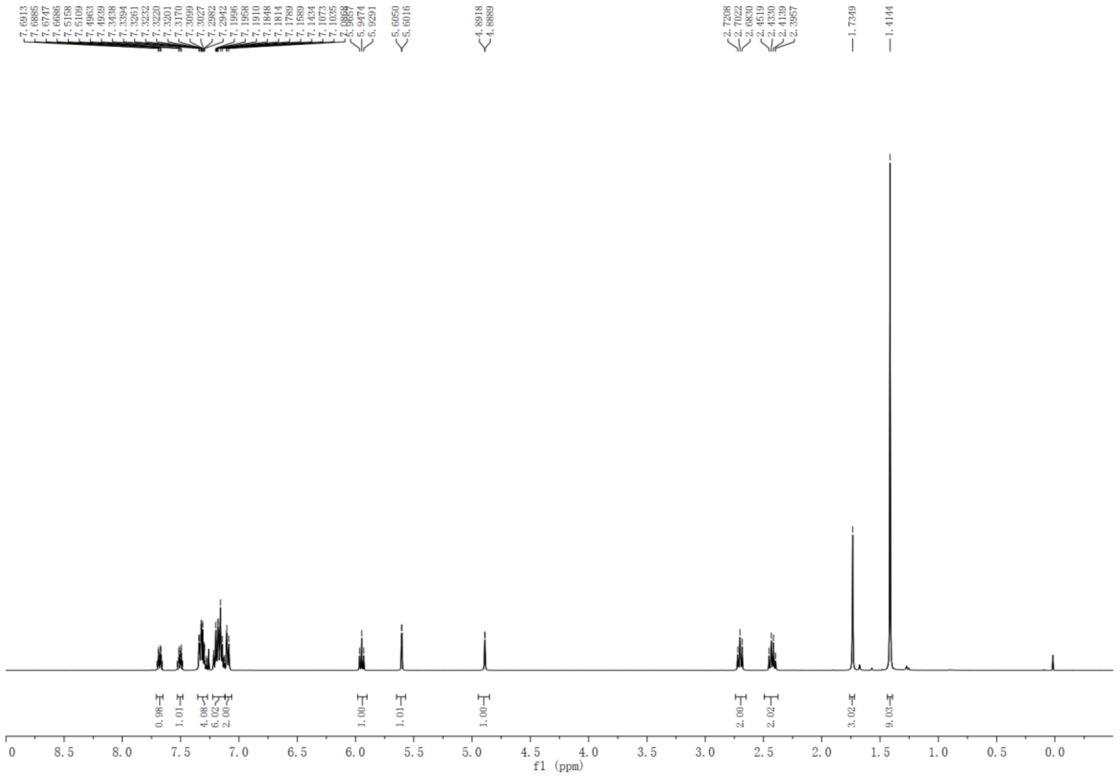

Comparison of ^{1}H NMR spectrum of **3a** (blue) and crude **3a** (rad)

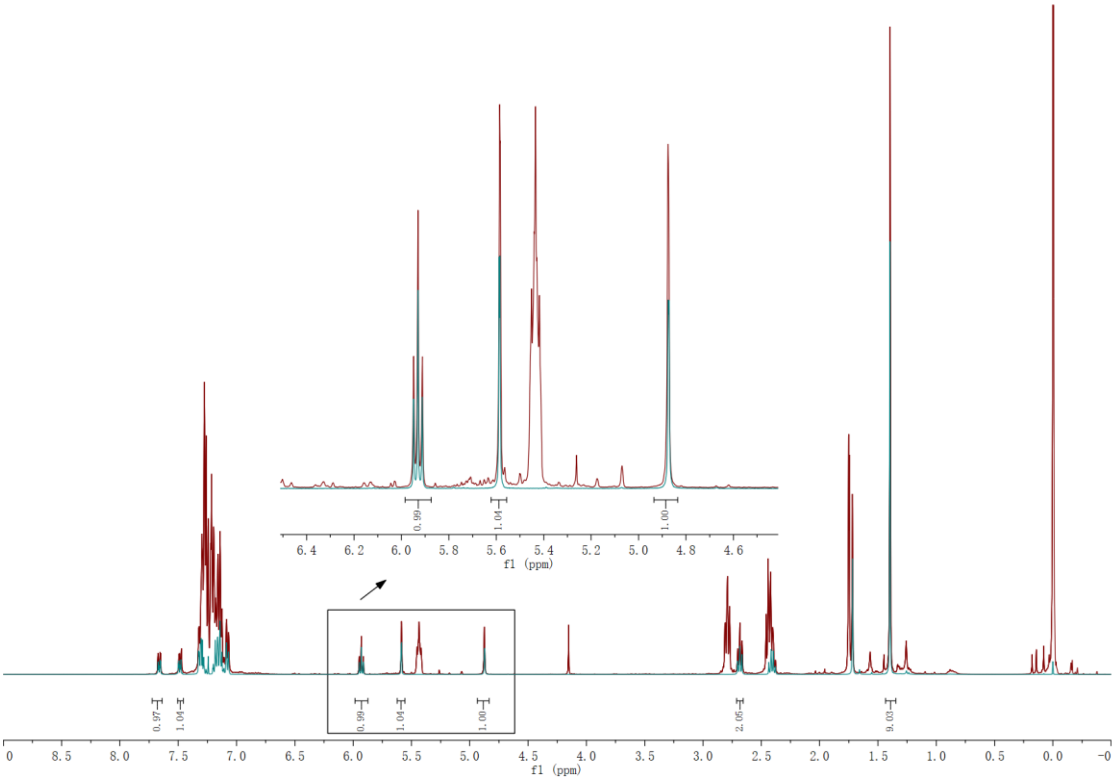

NOESY spectrum of **3a**

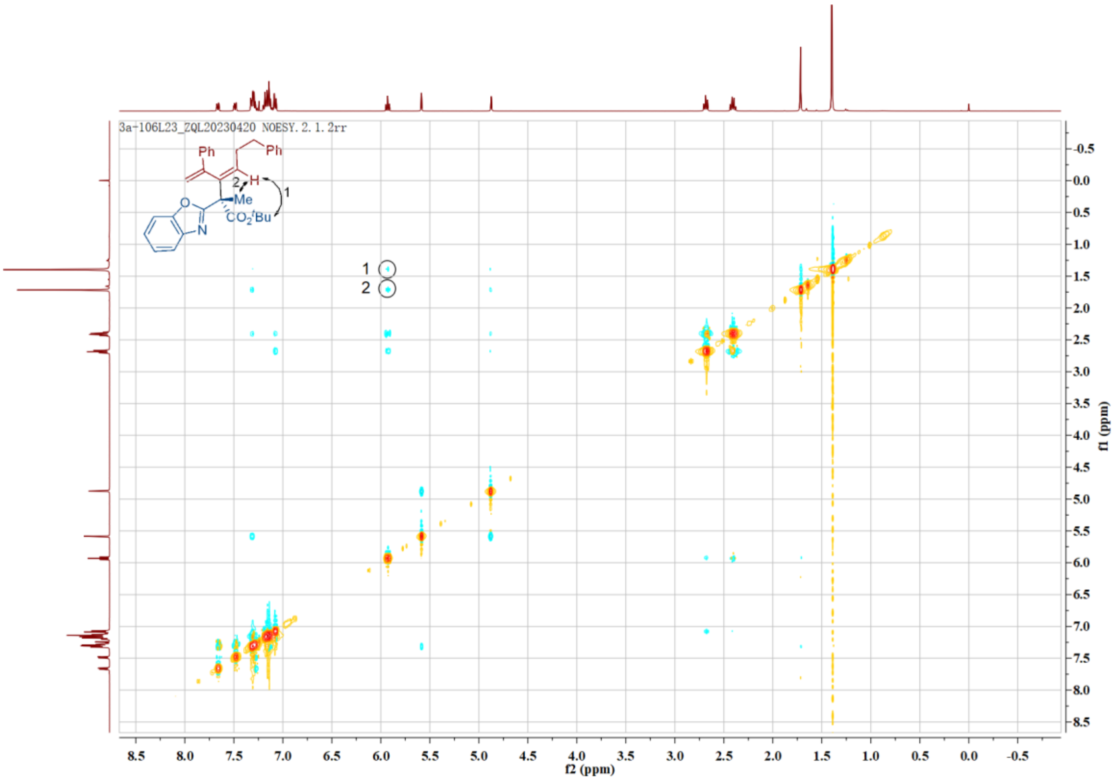

The NOESY spectrum of **3a** suggests that product **3a** is in the *E* configuration.

^{13}C NMR spectrum of **3a**

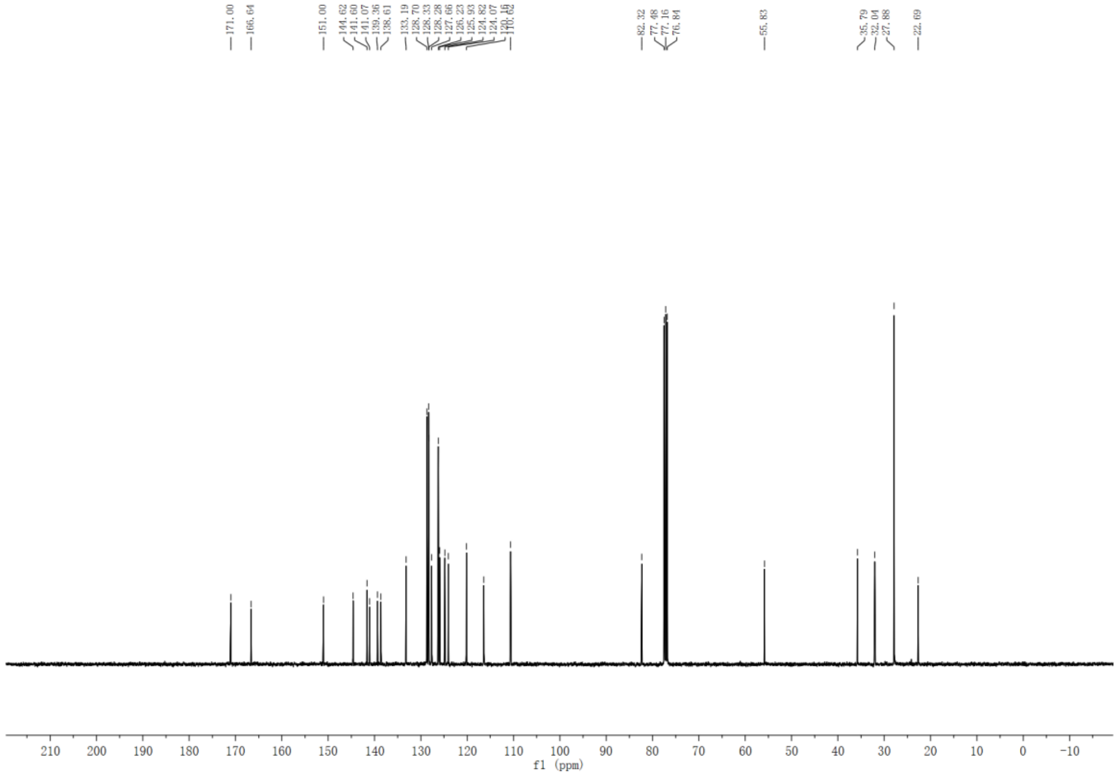

^{1}H NMR spectrum of **3b**

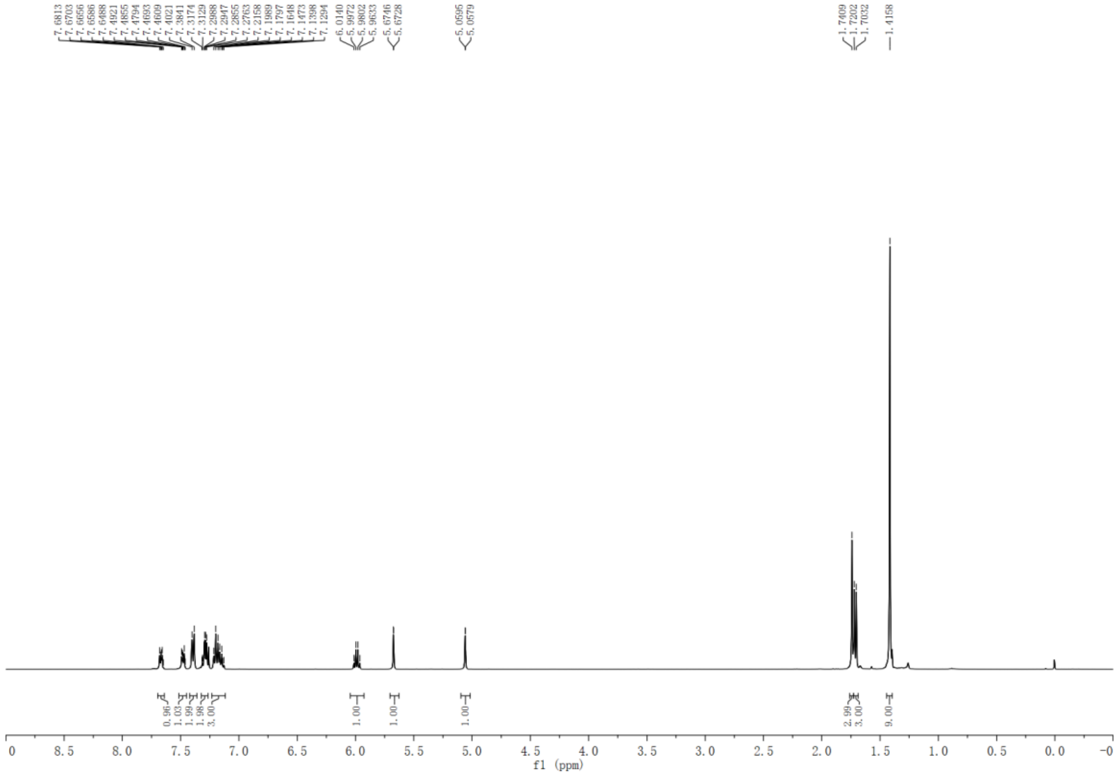

^{13}C NMR spectrum of **3b**

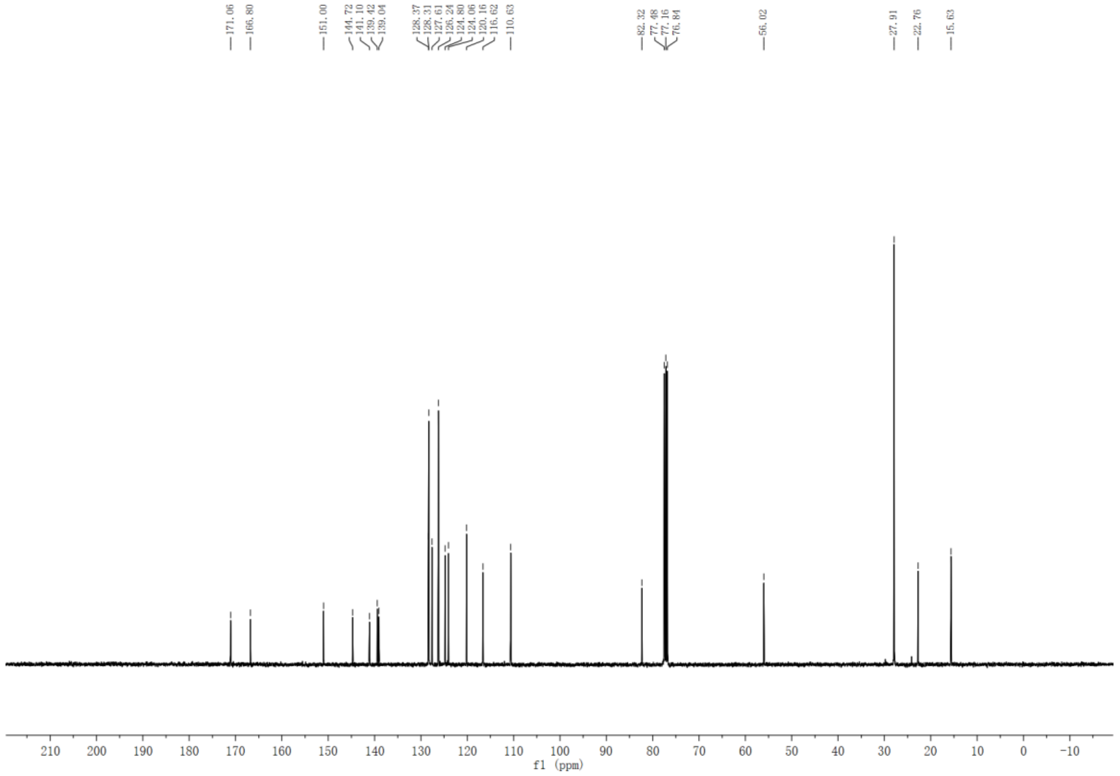

^{1}H NMR spectrum of **3c**

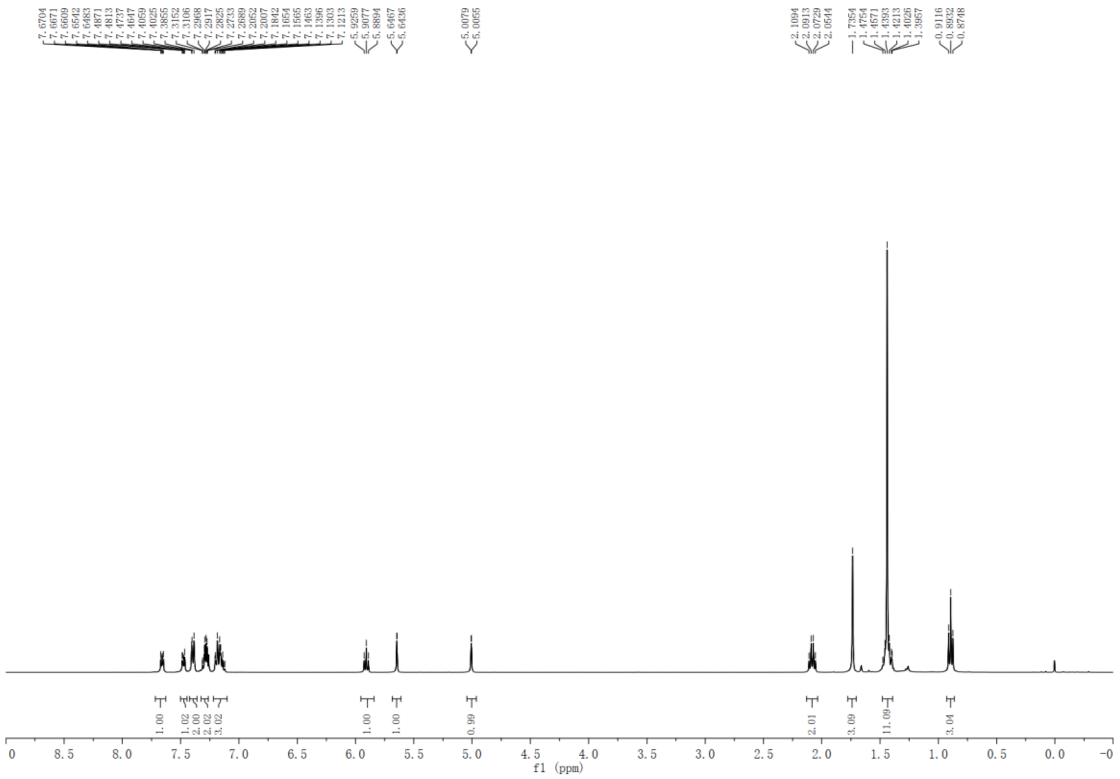

^{13}C NMR spectrum of **3c**

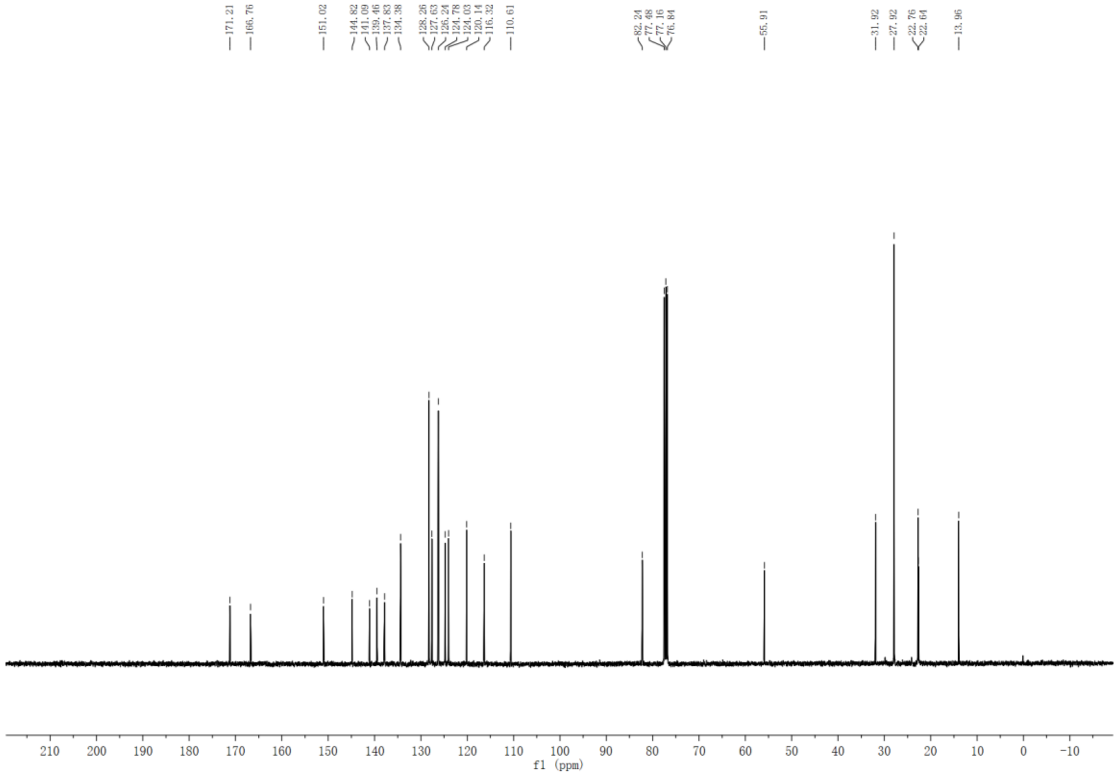

^{1}H NMR spectrum of **3d**

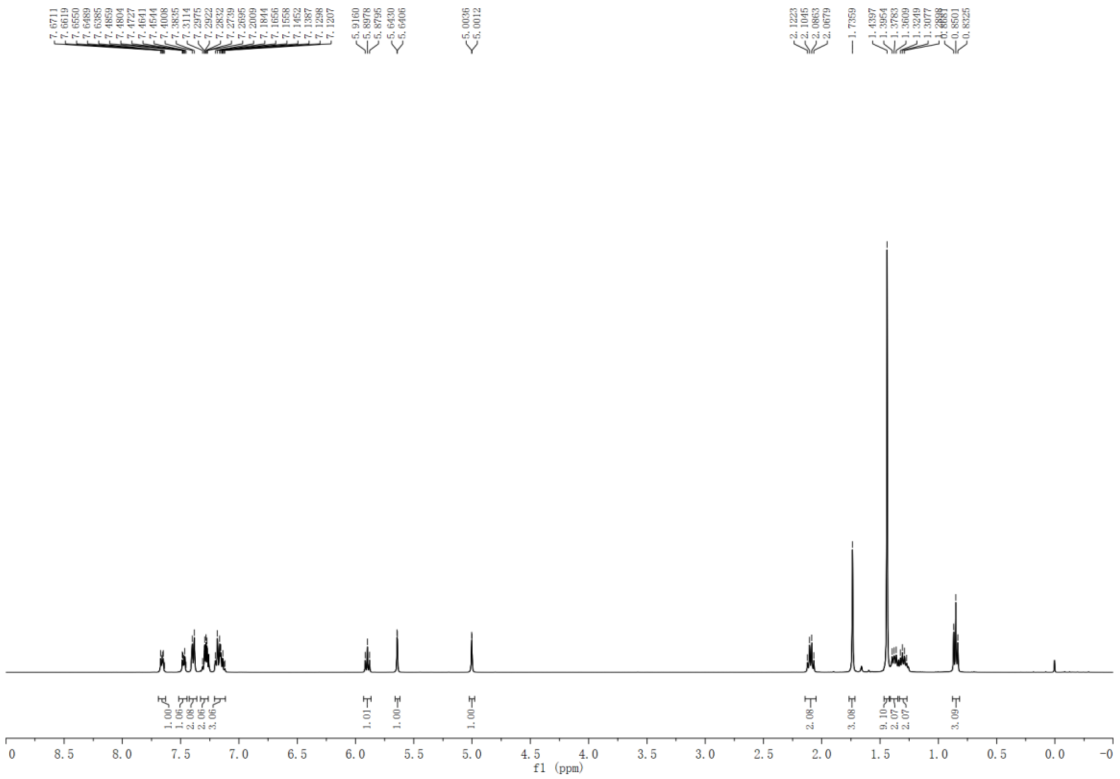

^{13}C NMR spectrum of **3d**

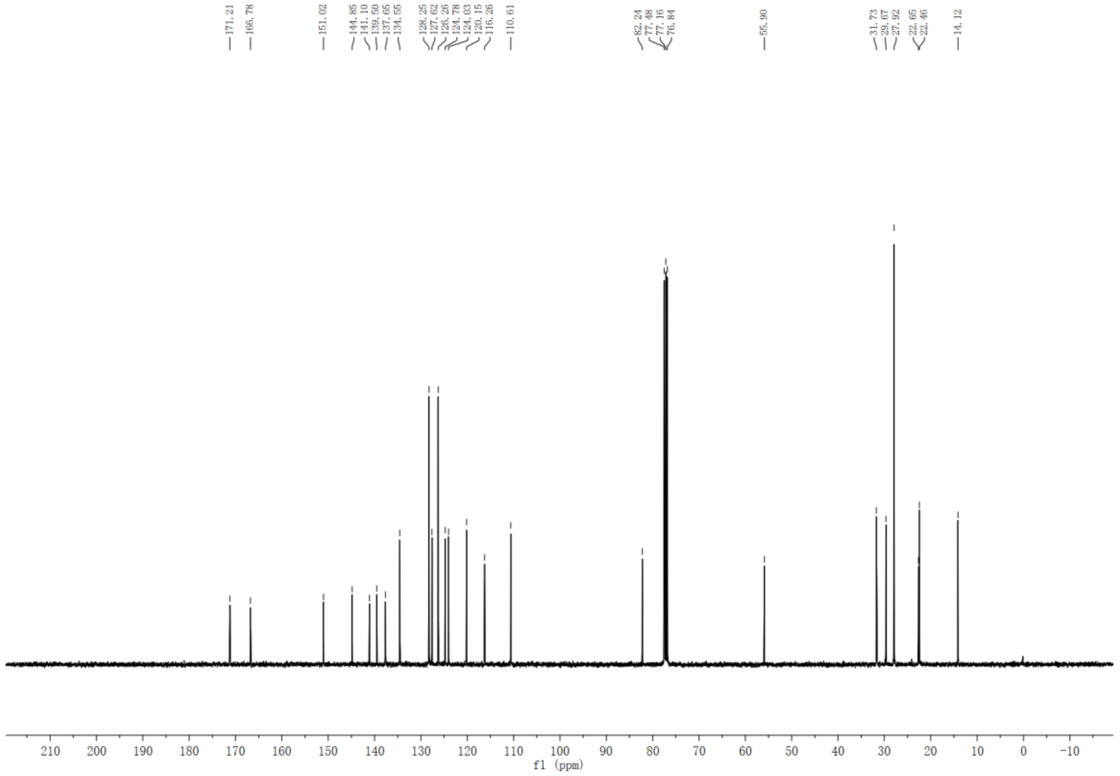

^{1}H NMR spectrum of **3e**

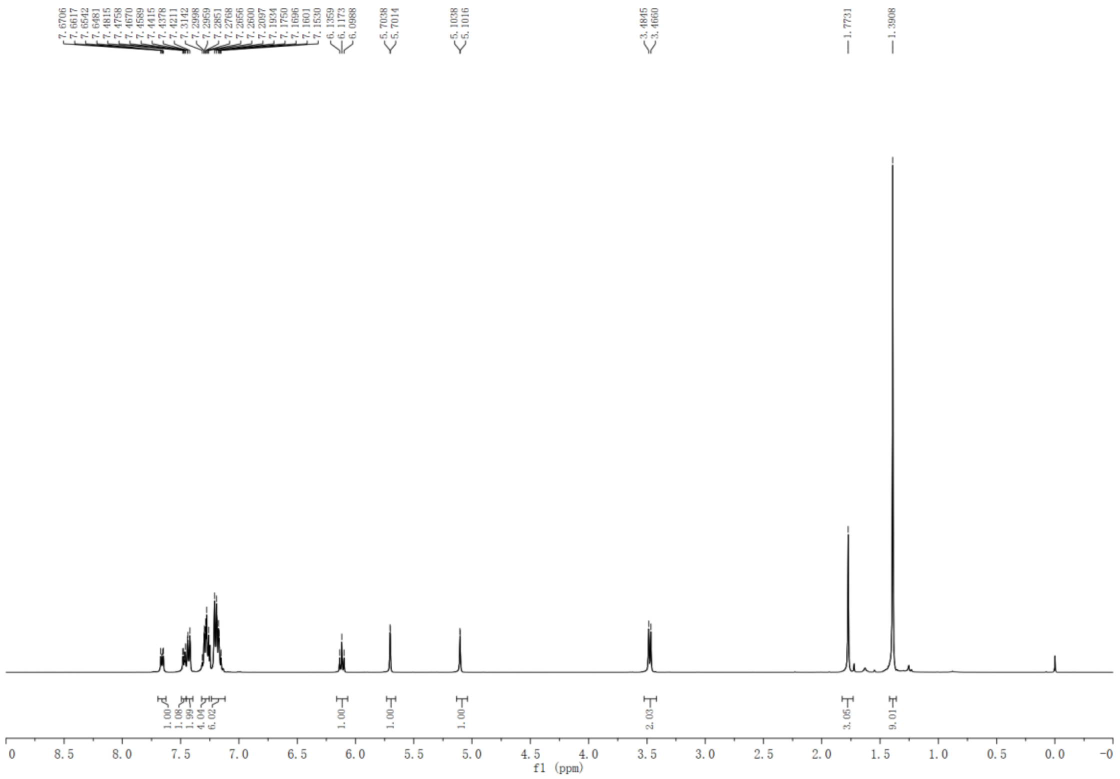

^{13}C NMR spectrum of **3e**

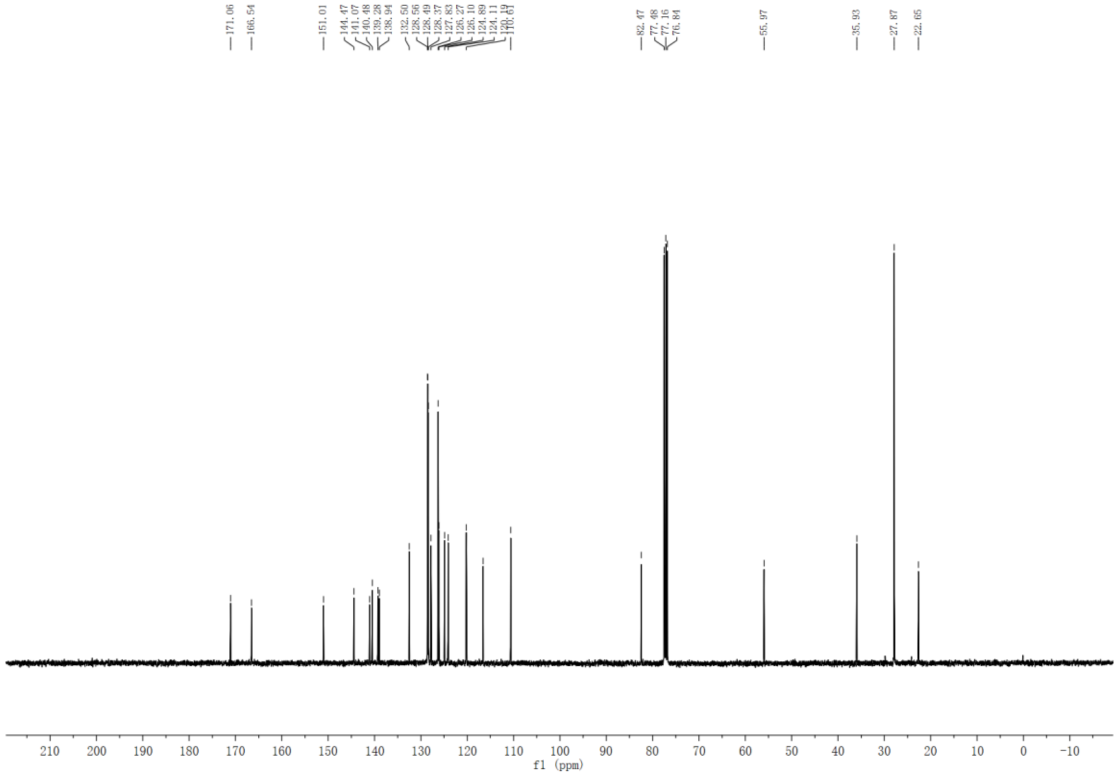

^{1}H NMR spectrum of **3f**

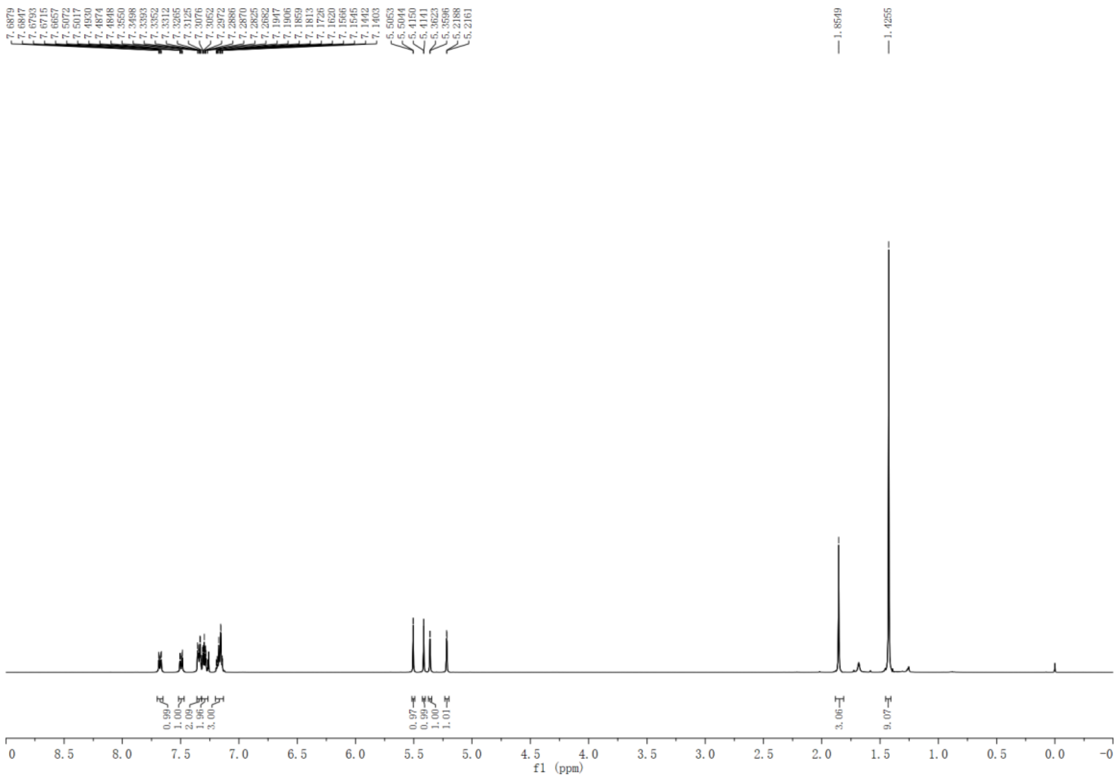

^{13}C NMR spectrum of **3f**

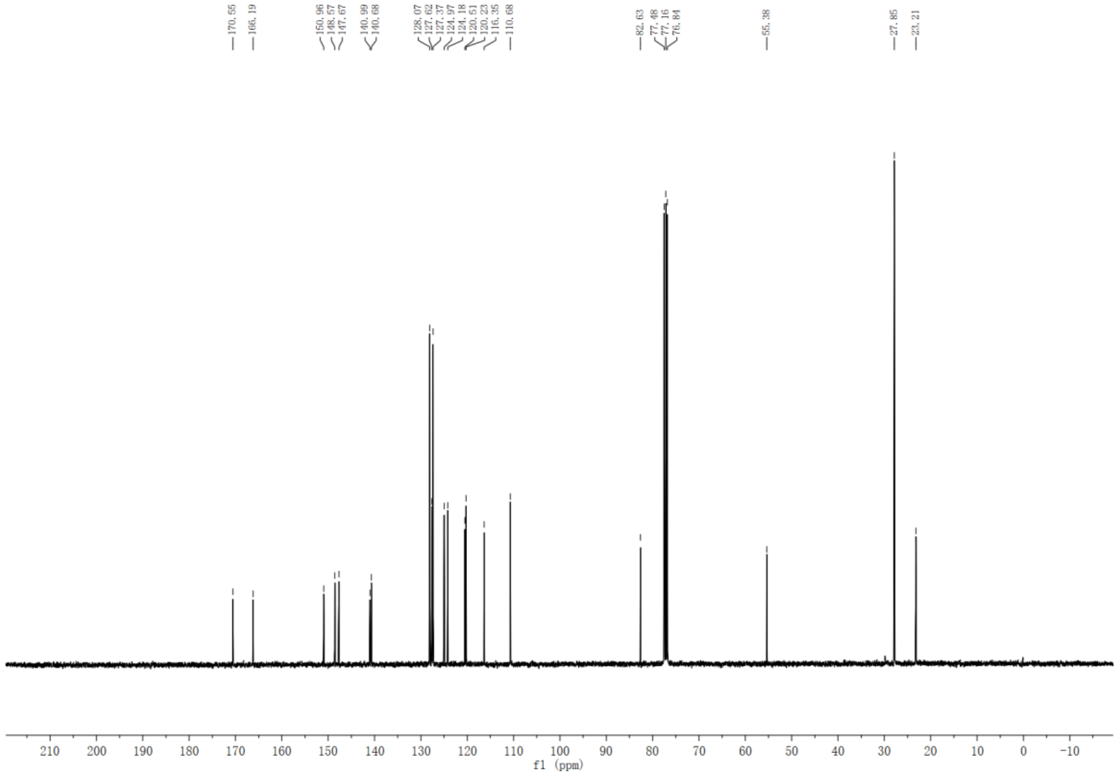

^{1}H NMR spectrum of crude **3g**

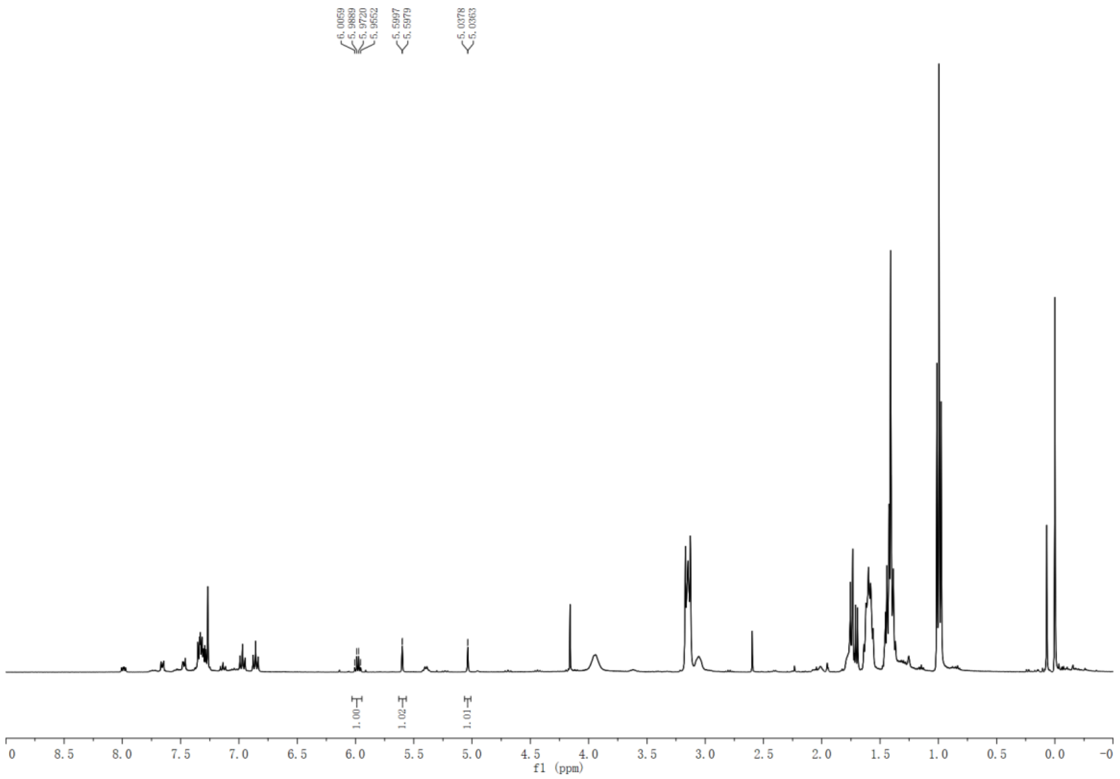

^{1}H NMR spectrum of **3g**

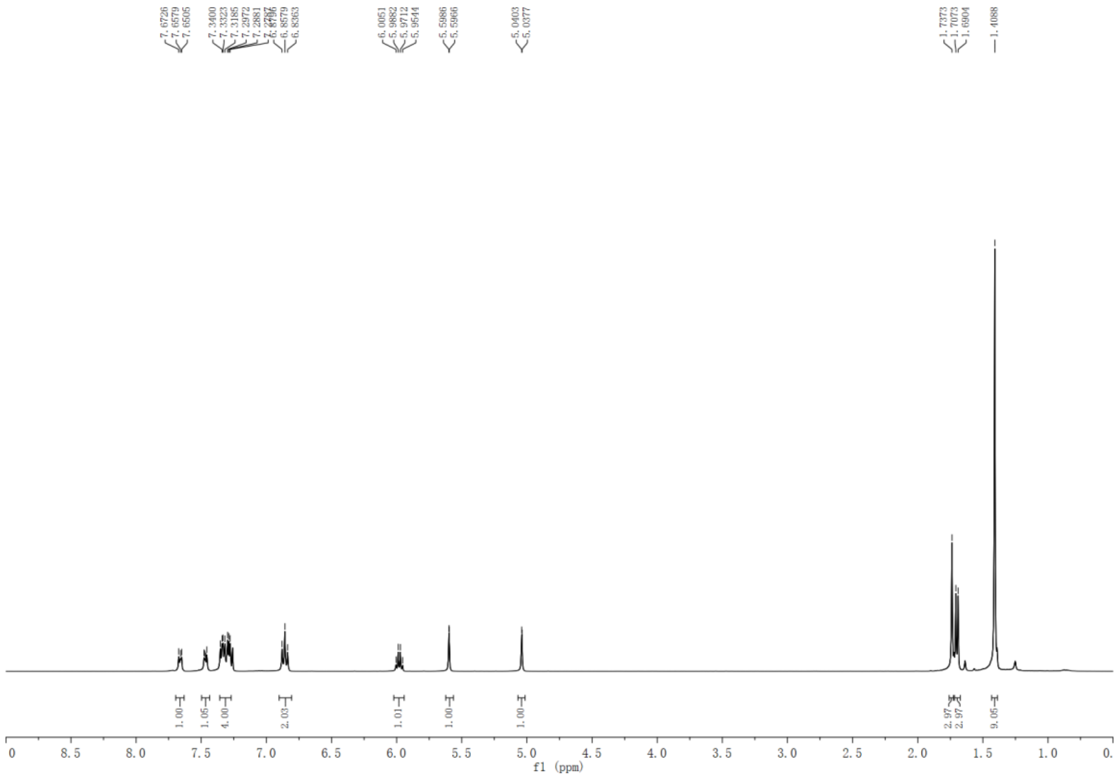

Comparison of ^{1}H NMR spectrum of **3g** (blue) and crude **3g** (red)

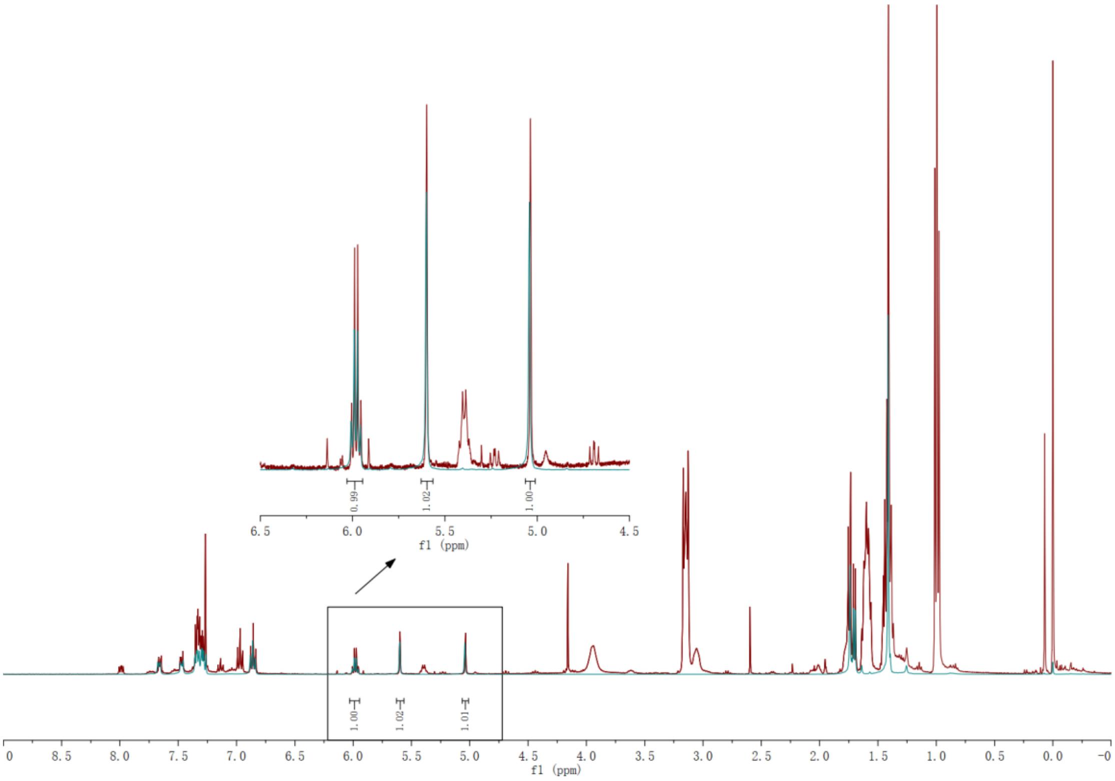

NOESY spectrum of **3g**

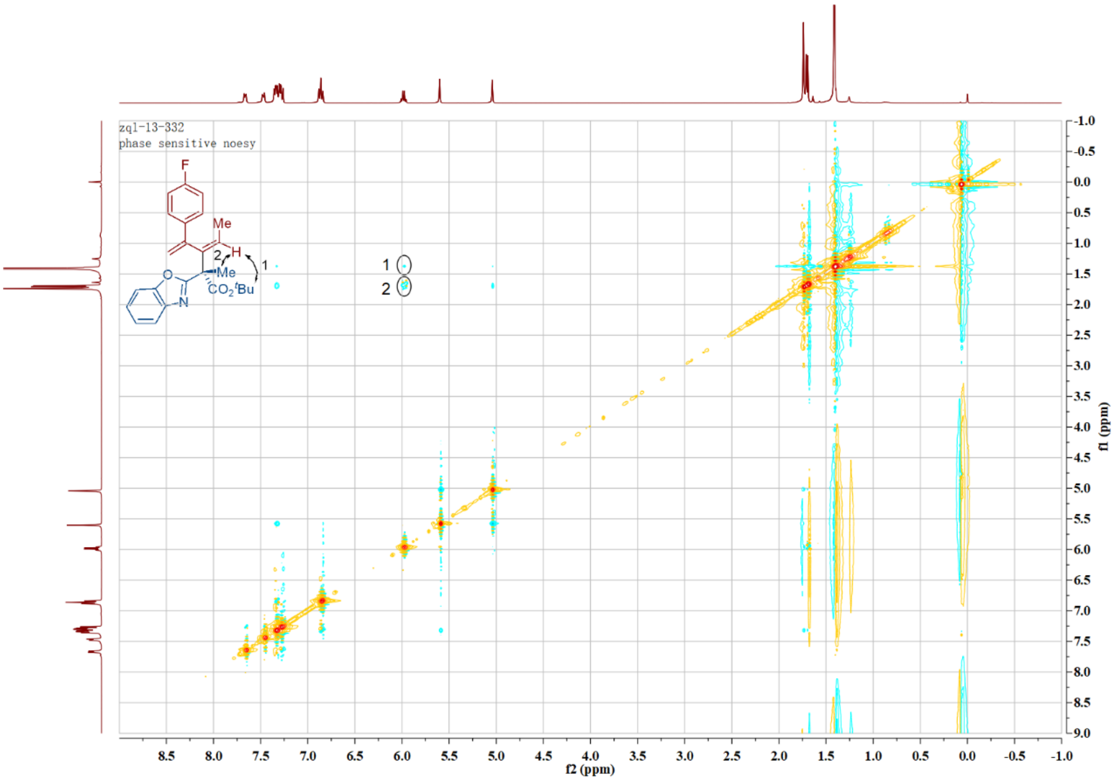

The NOESY spectrum of **3g** suggests that product **3g** is in the *E* configuration.

^{13}C NMR spectrum of **3g**

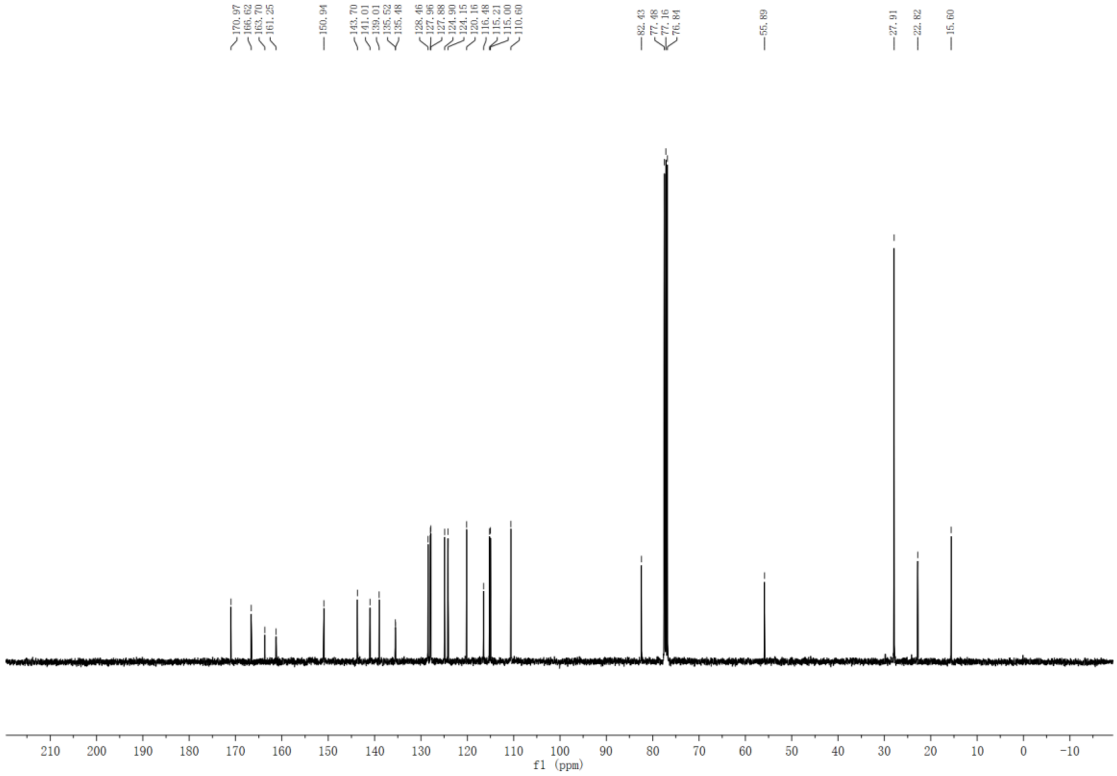

^{19}F NMR spectrum of **3g**

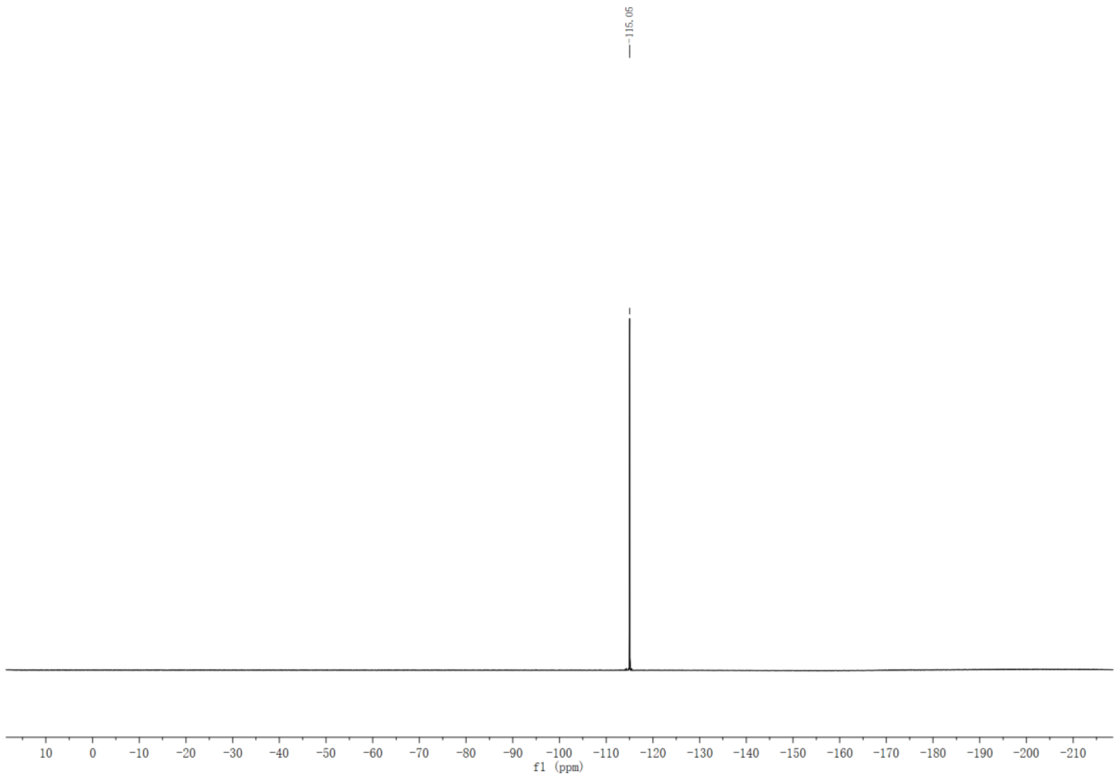

^{1}H NMR spectrum of **3h**

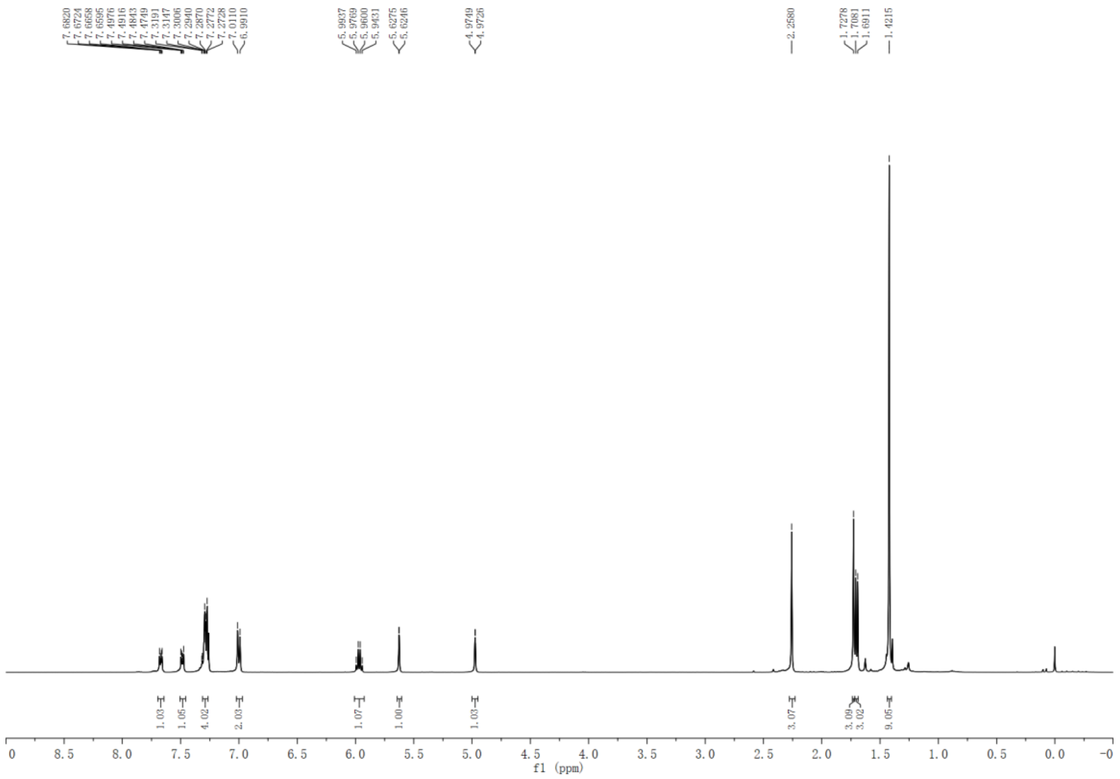

^{13}C NMR spectrum of **3h**

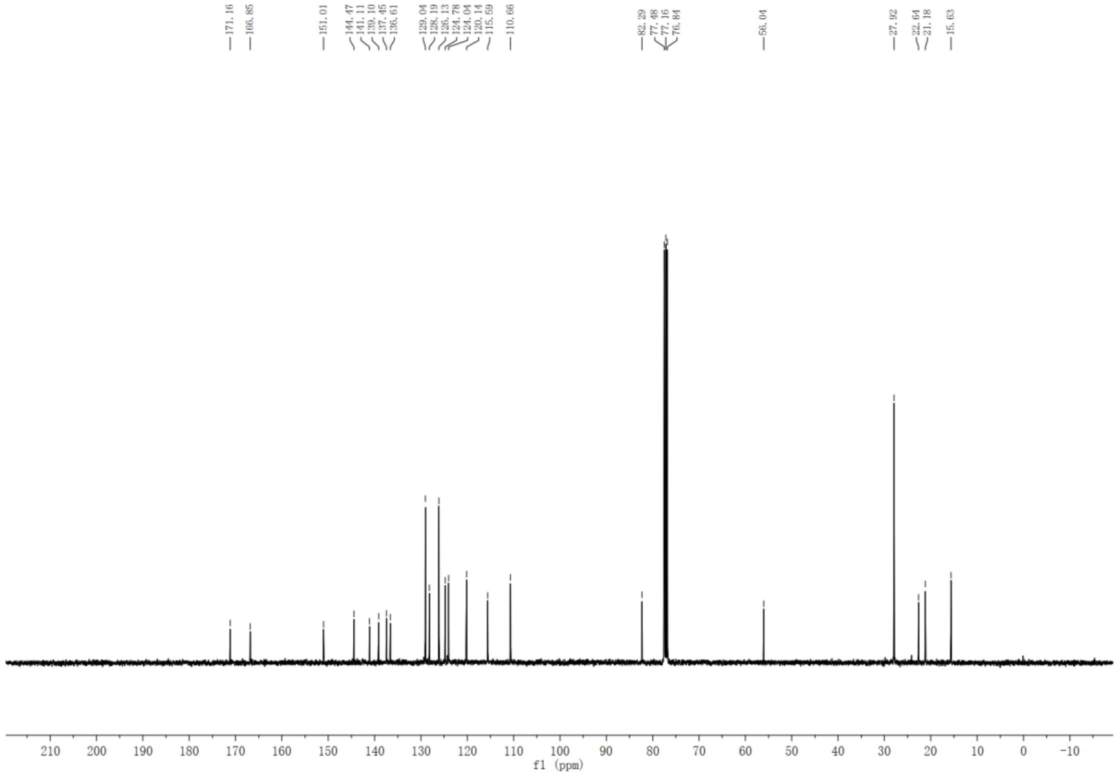

^{1}H NMR spectrum of **3i**

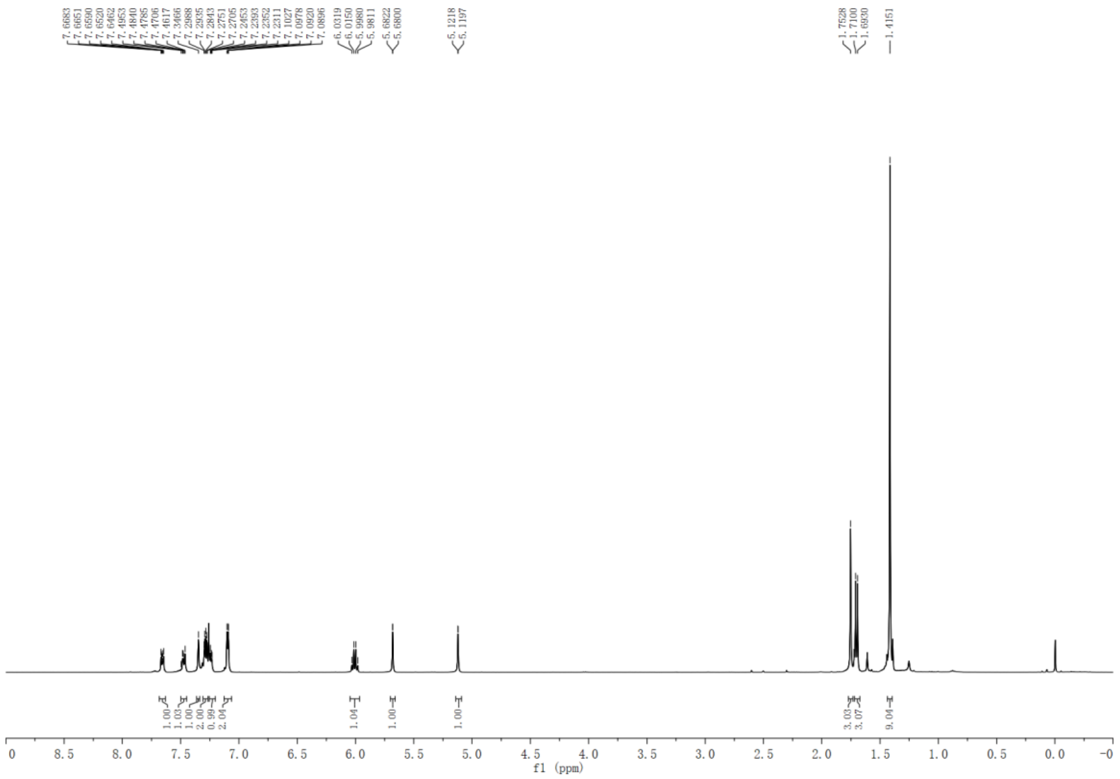

^{13}C NMR spectrum of **3i**

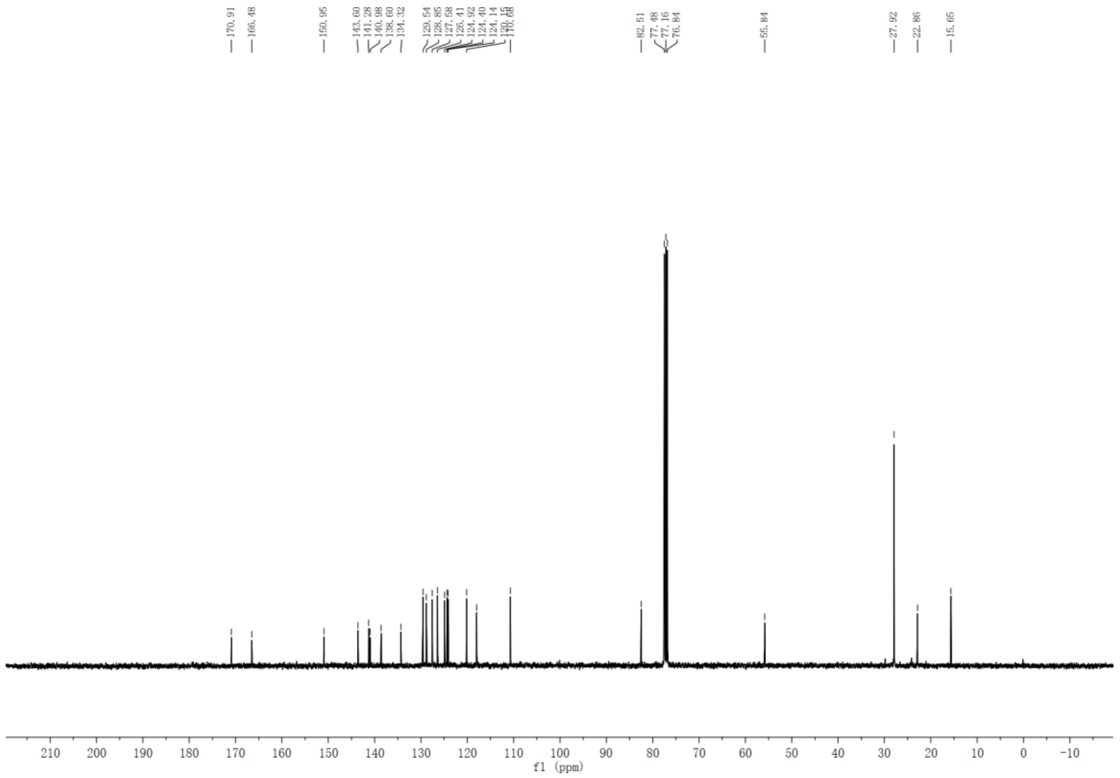

^{1}H NMR spectrum of crude **3j**

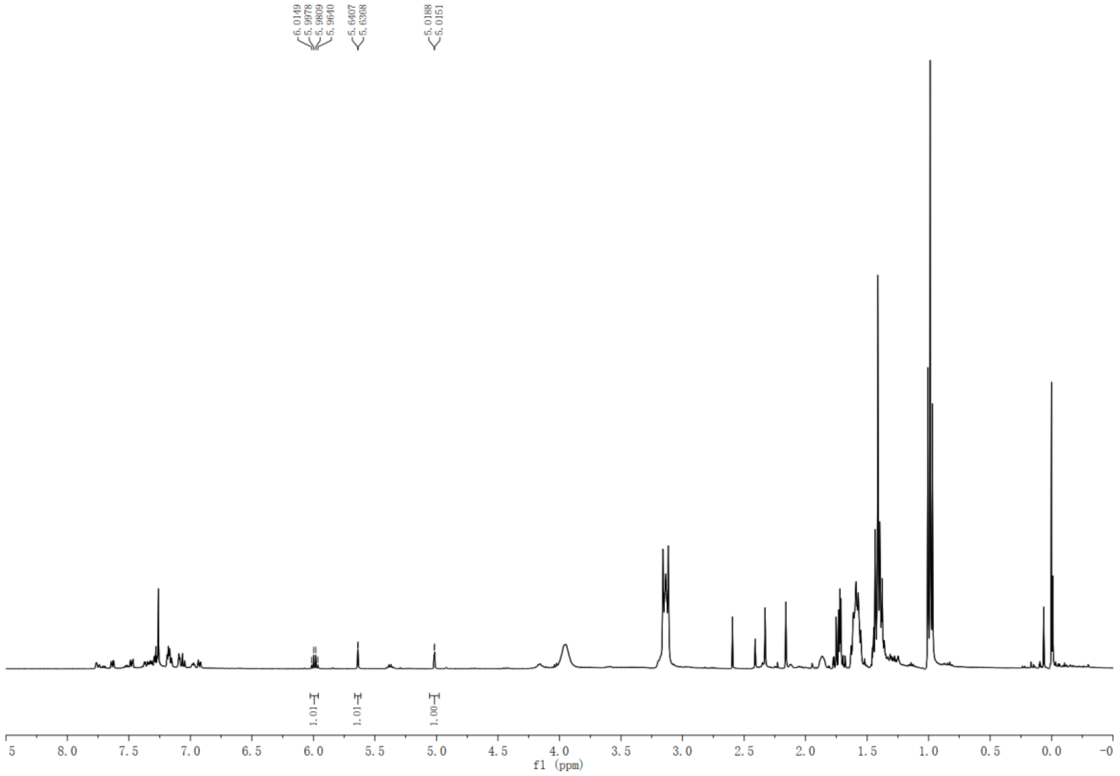

^{1}H NMR spectrum of **3j**

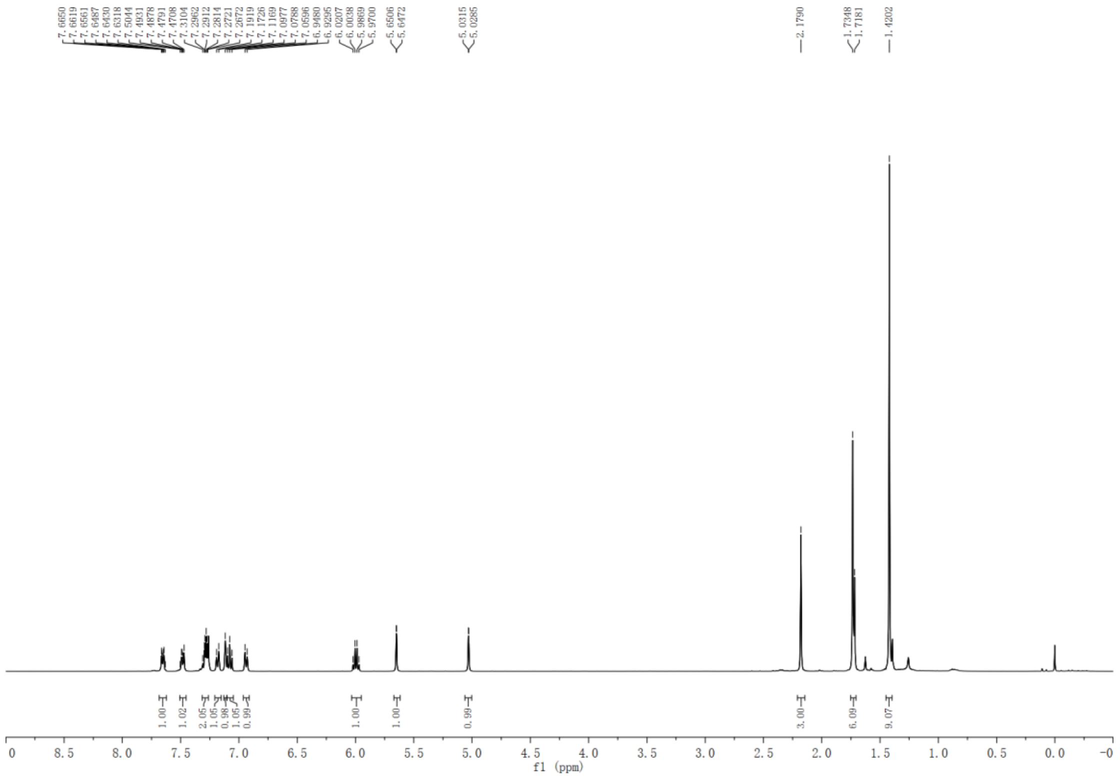

Comparison of ^{1}H NMR spectrum of **3j** (blue) and crude **3j** (red)

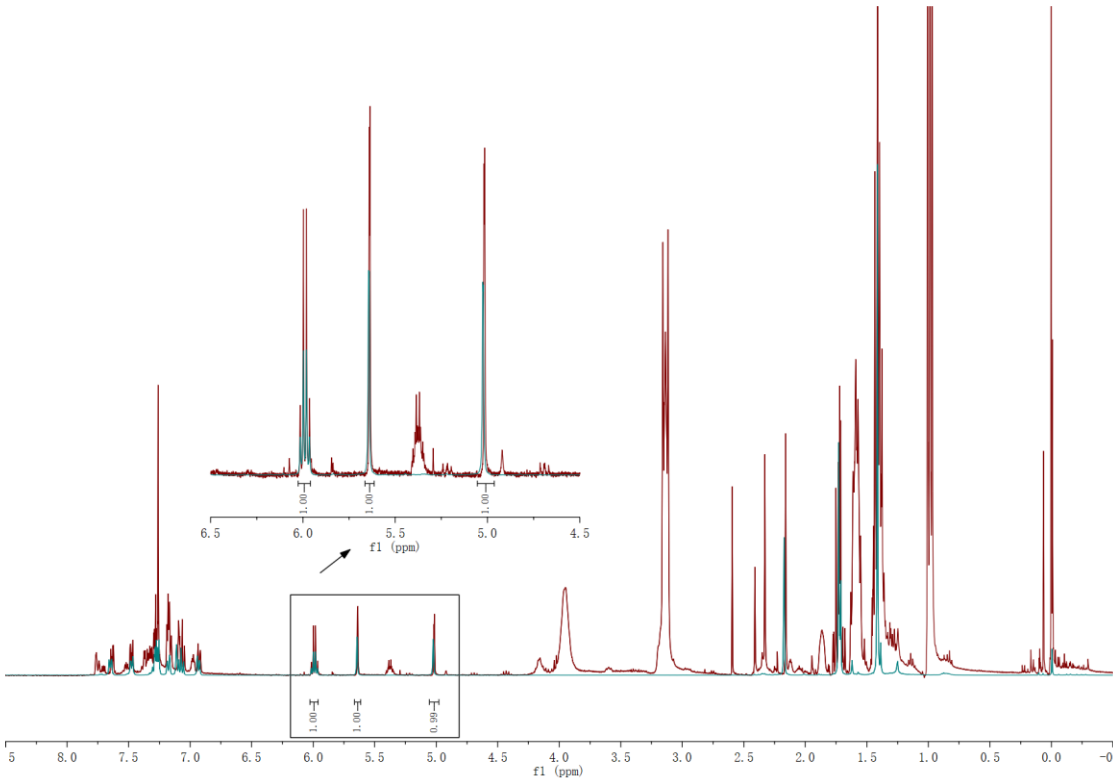

NOESY spectrum of **3j**

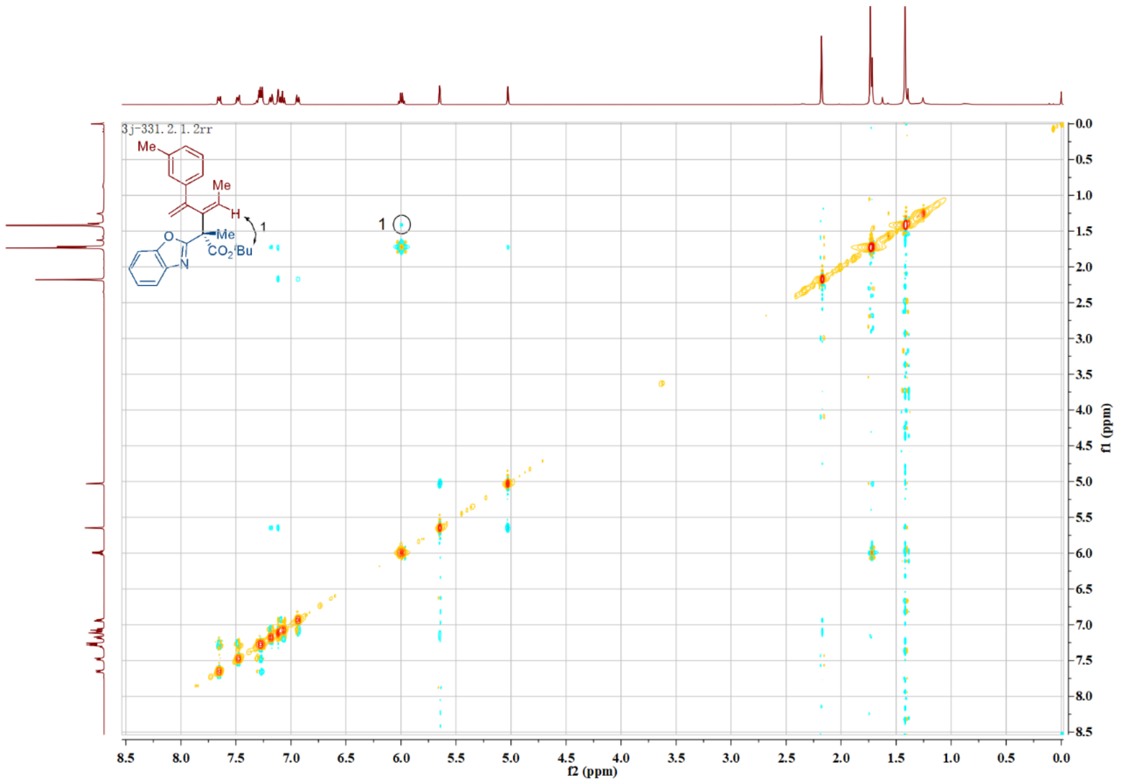

The NOESY spectrum of **3j** suggests that product **3j** is in the *E* configuration.

^{13}C NMR spectrum of **3j**

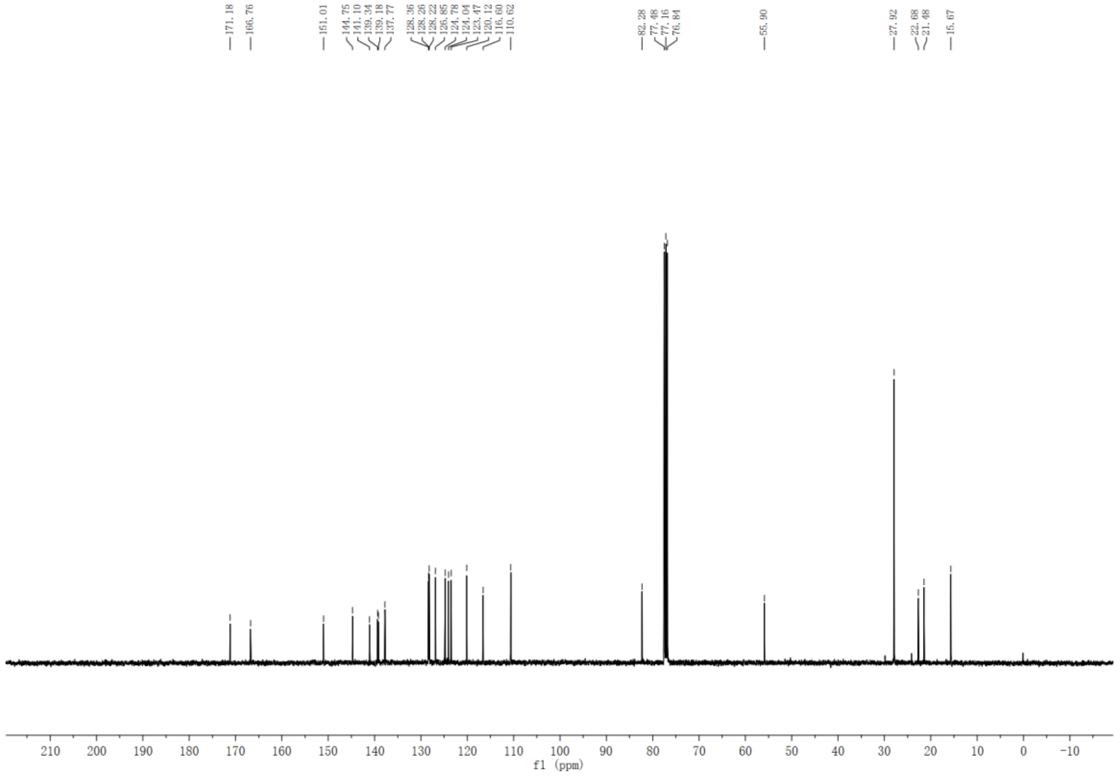

^{1}H NMR spectrum of **3k**

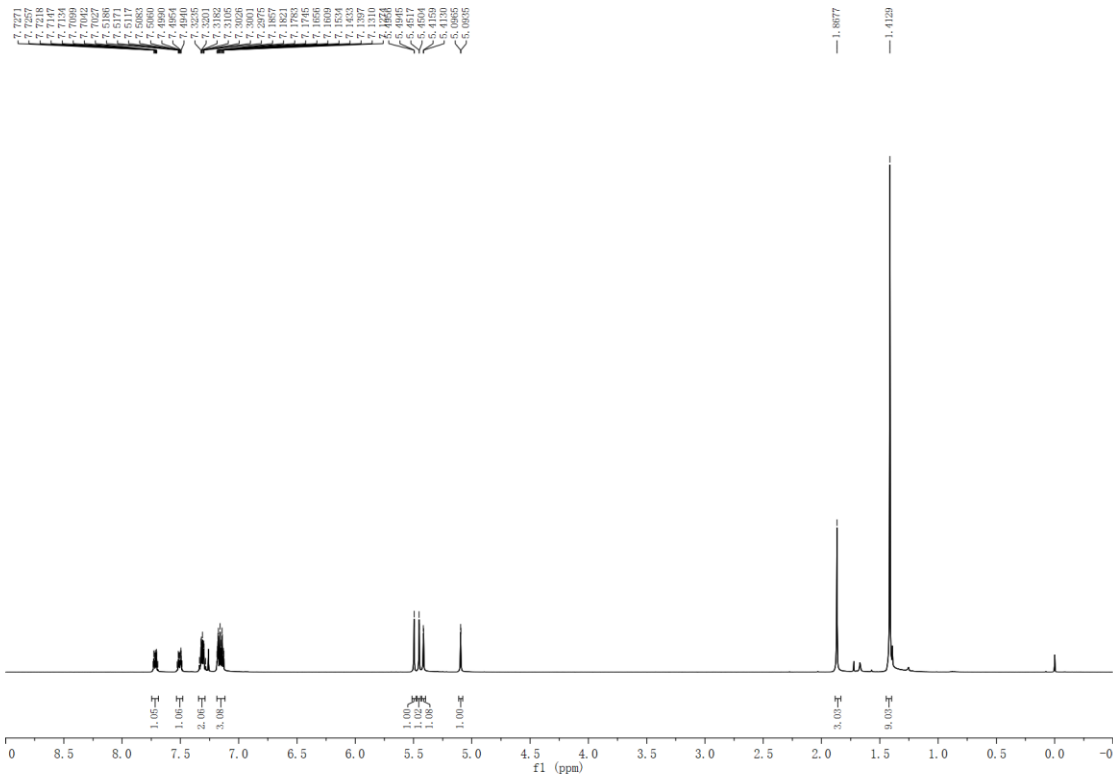

^{13}C NMR spectrum of **3k**

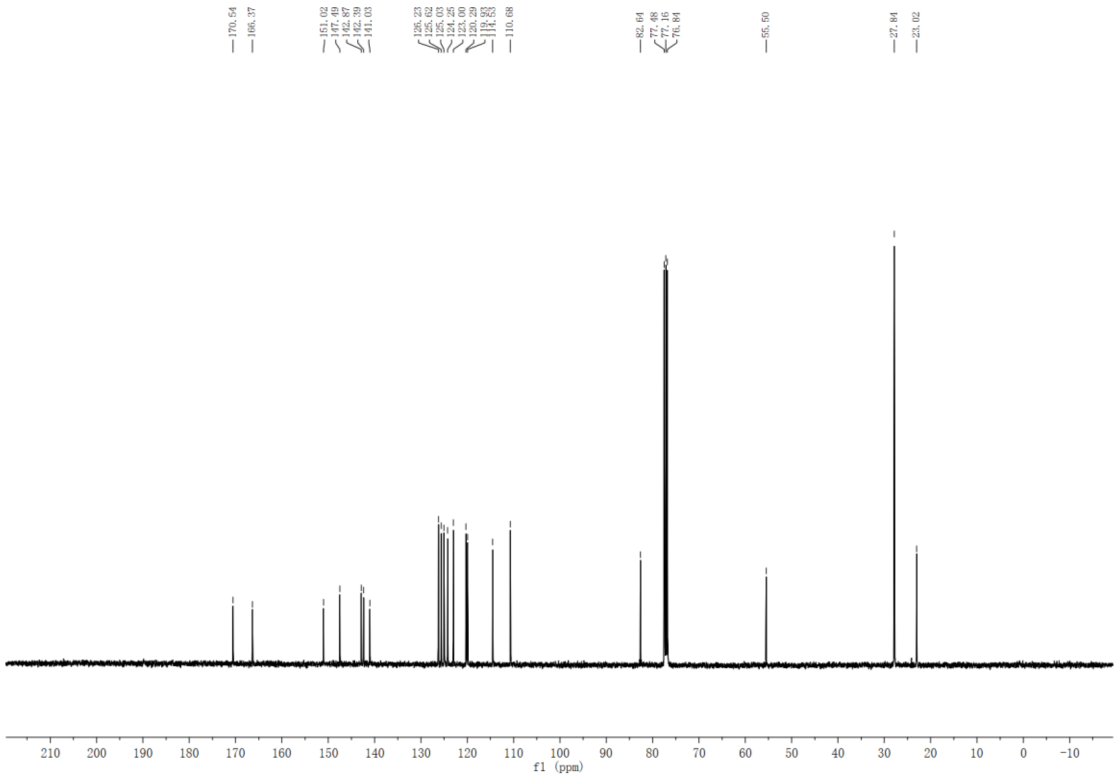

^{1}H NMR spectrum of **3l**

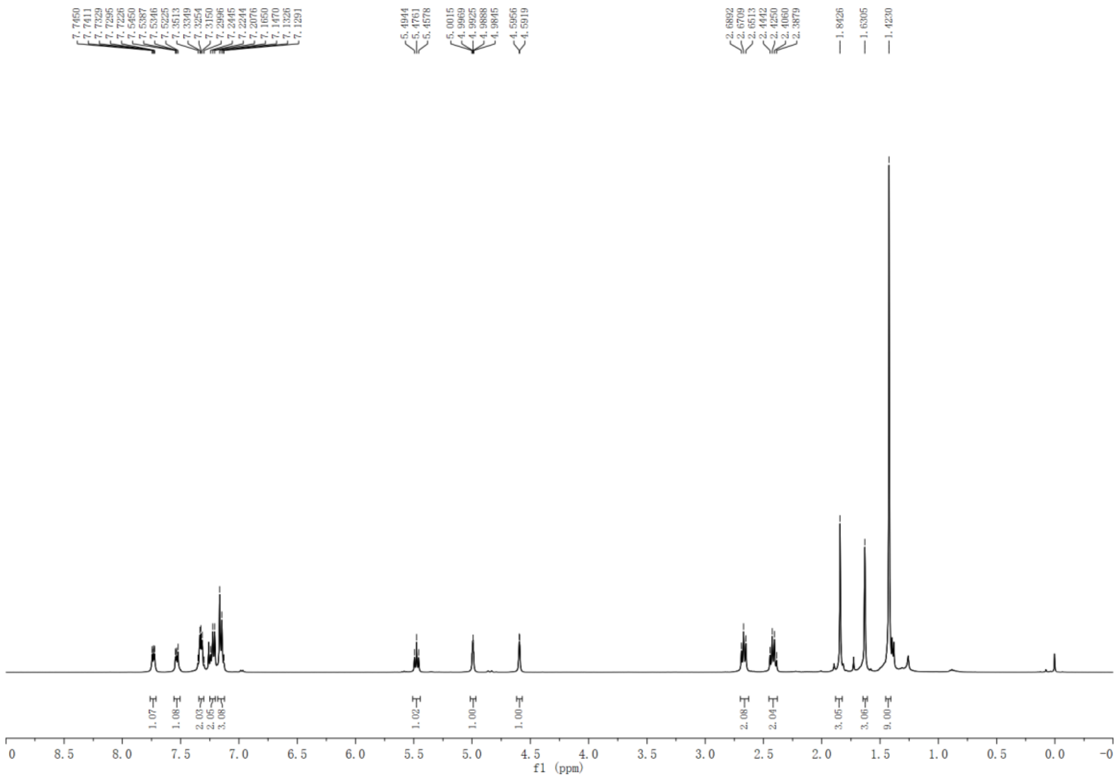

^{13}C NMR spectrum of **3l**

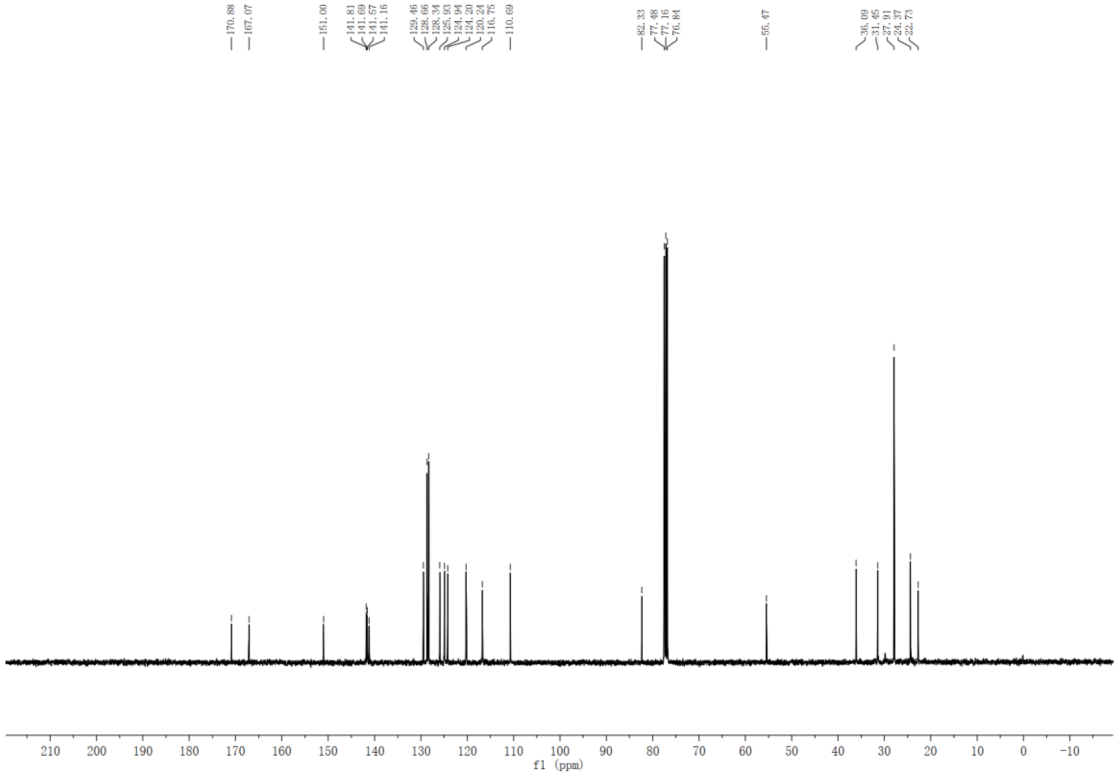

^{1}H NMR spectrum of **3m**

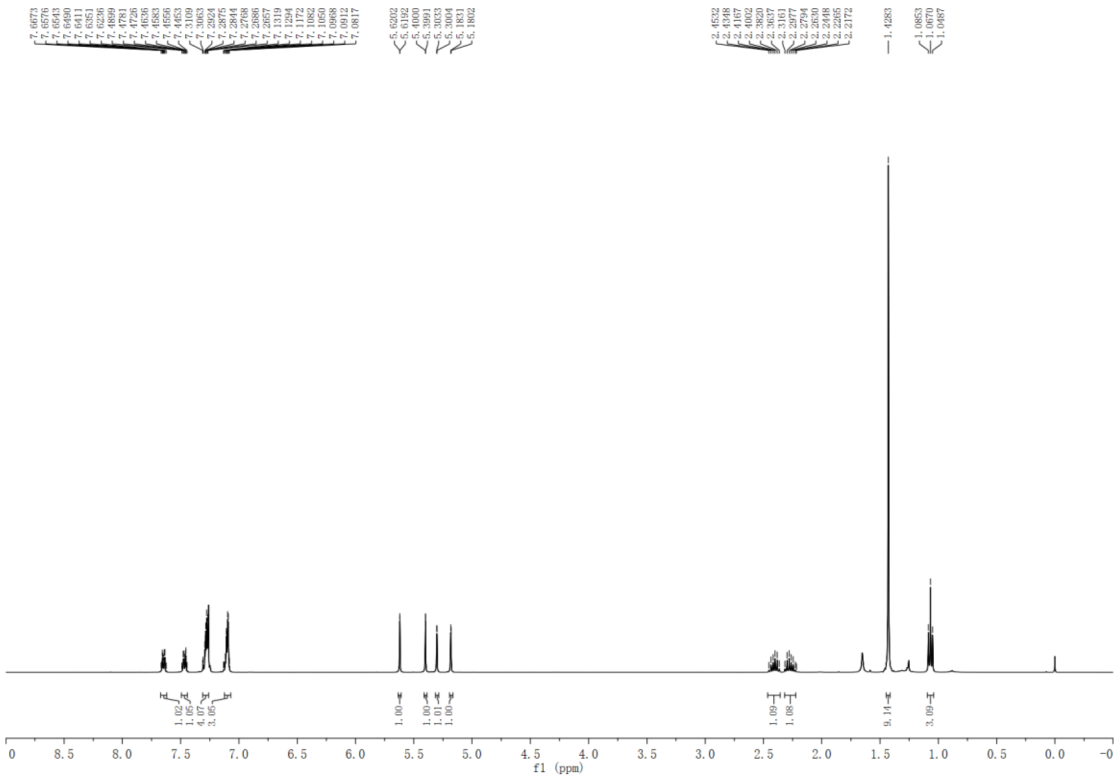

^{13}C NMR spectrum of **3m**

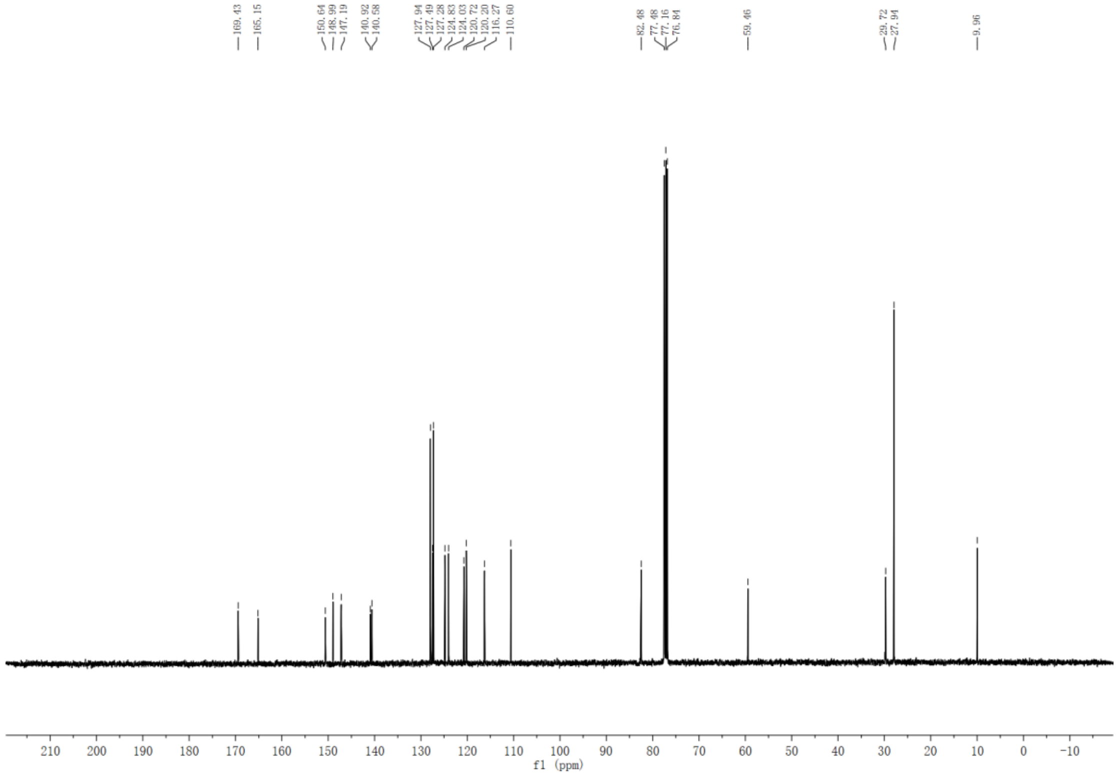

^{1}H NMR spectrum of **3n**

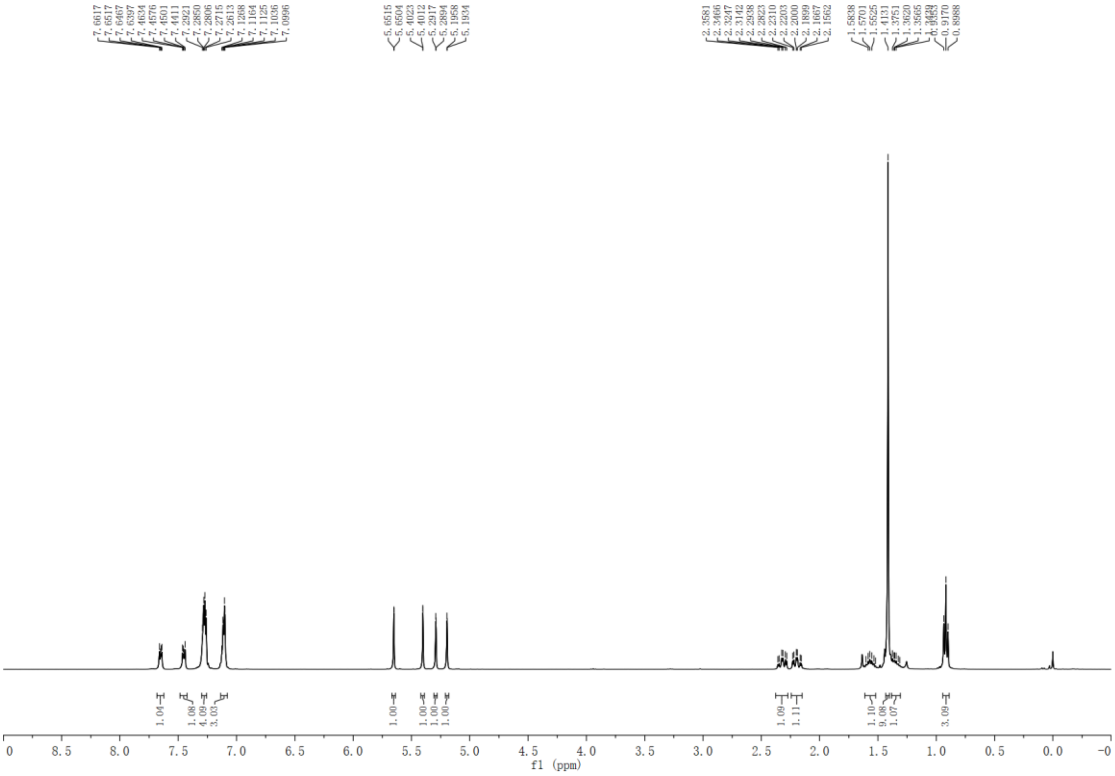

^{13}C NMR spectrum of **3n**

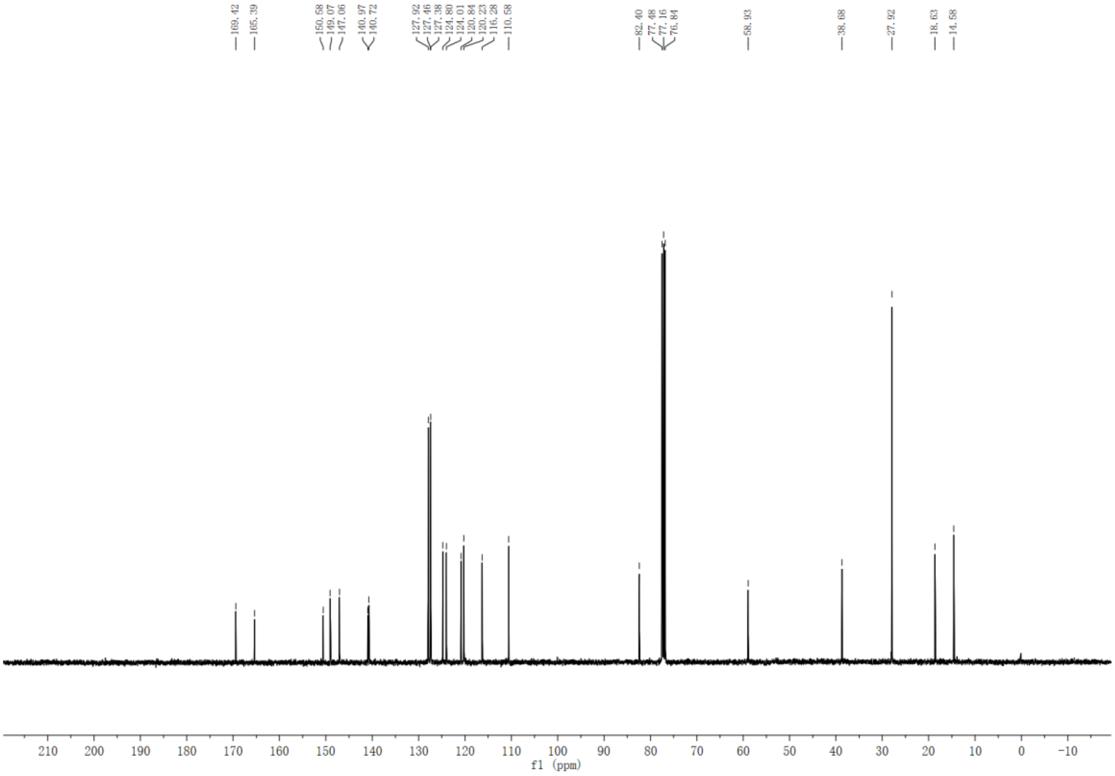

^{1}H NMR spectrum of **3o**

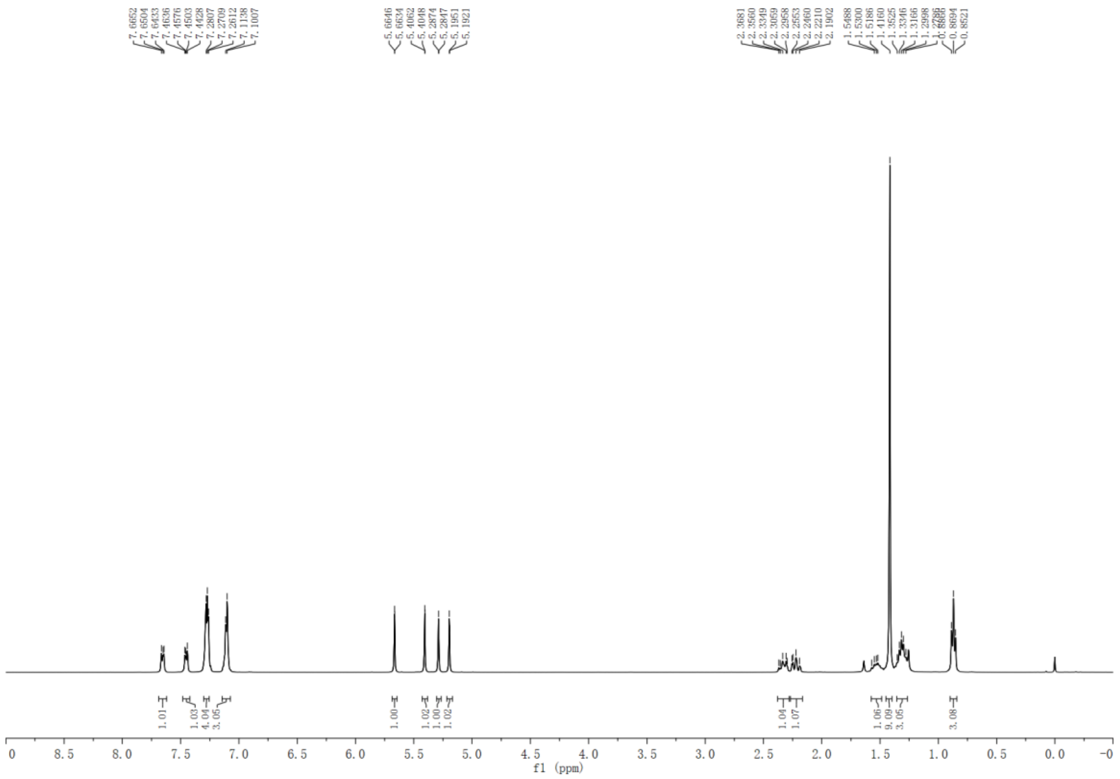

^{13}C NMR spectrum of **3o**

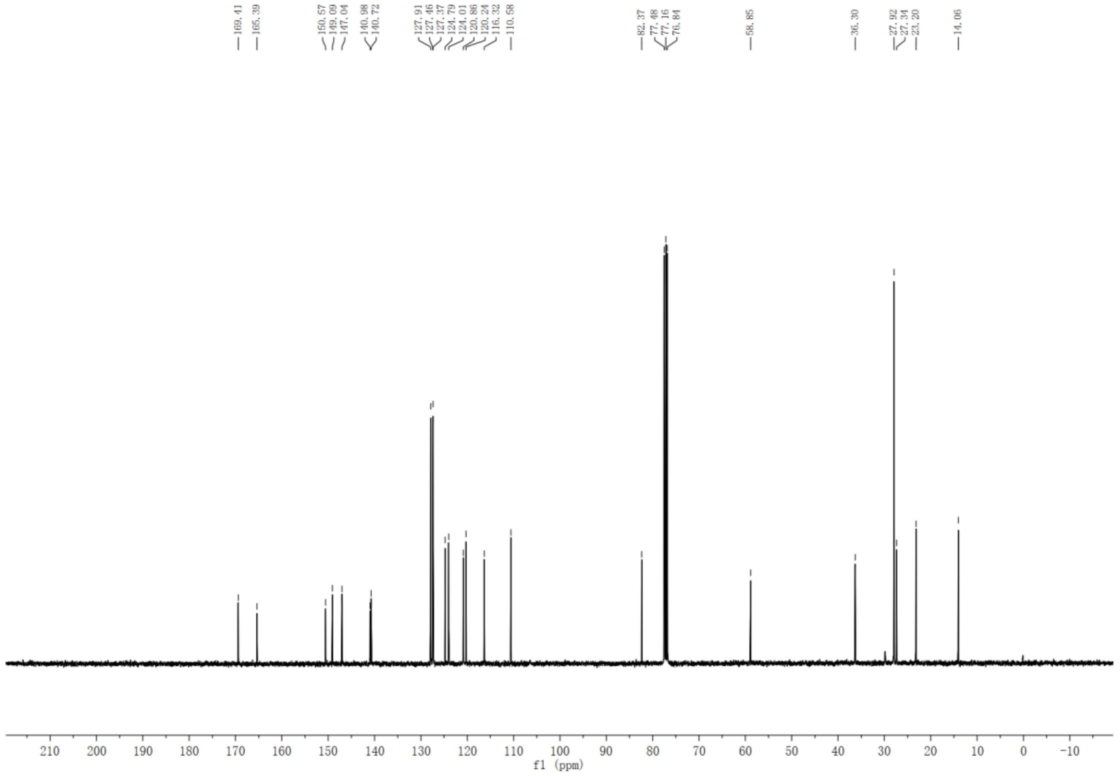

^{1}H NMR spectrum of **3p**

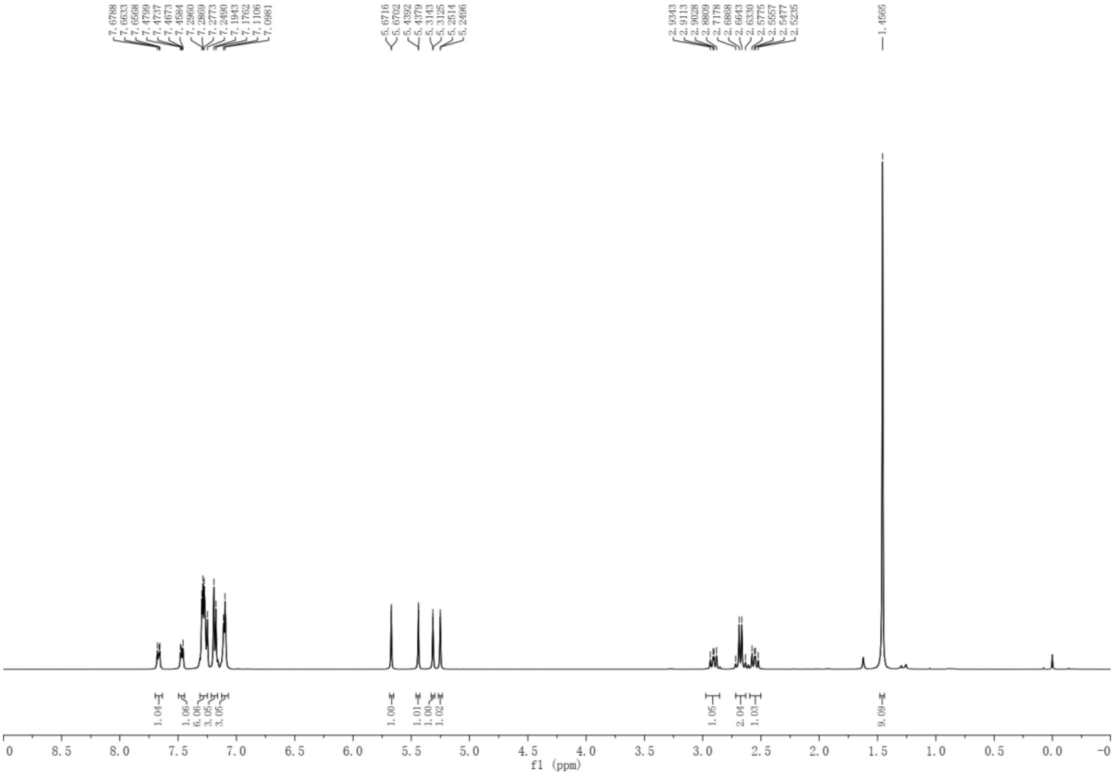

^{13}C NMR spectrum of **3p**

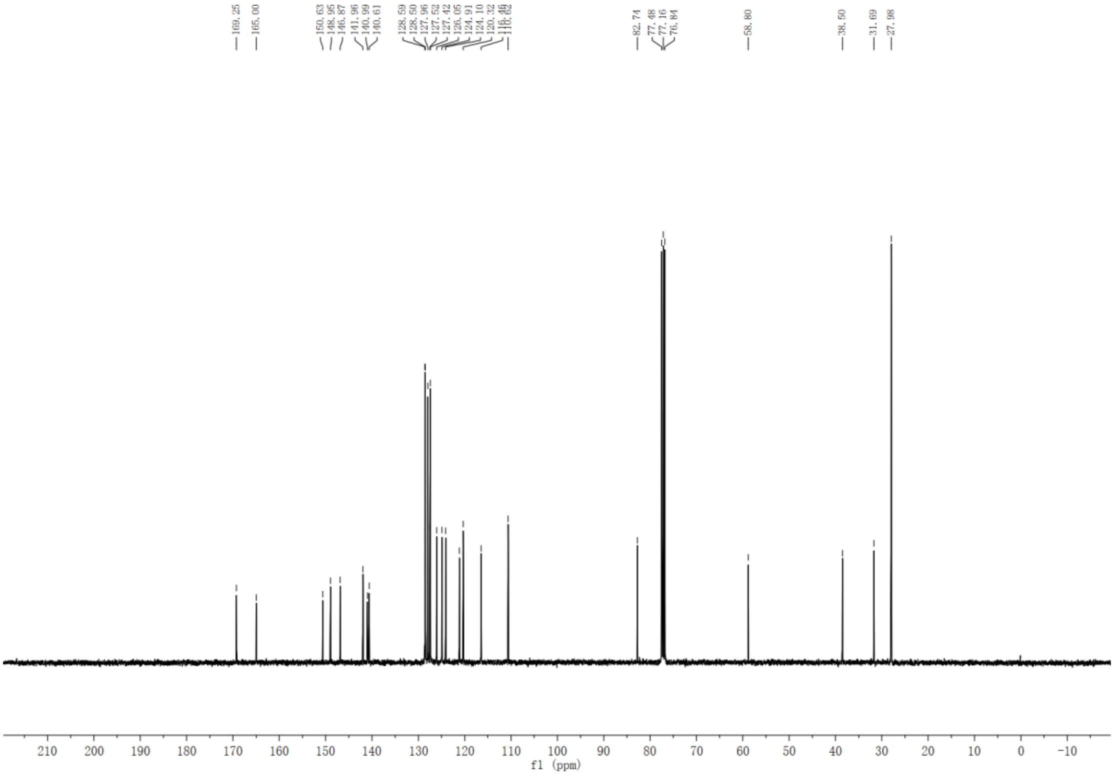

^{1}H NMR spectrum of **3q**

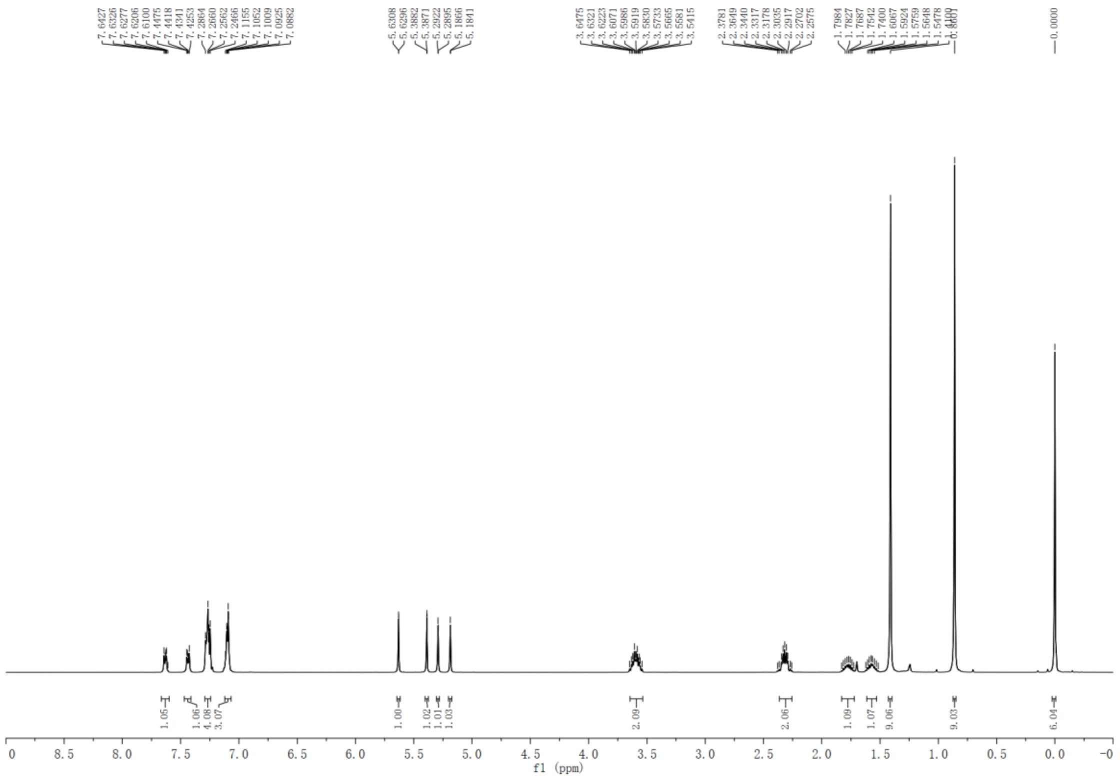

^{13}C NMR spectrum of **3q**

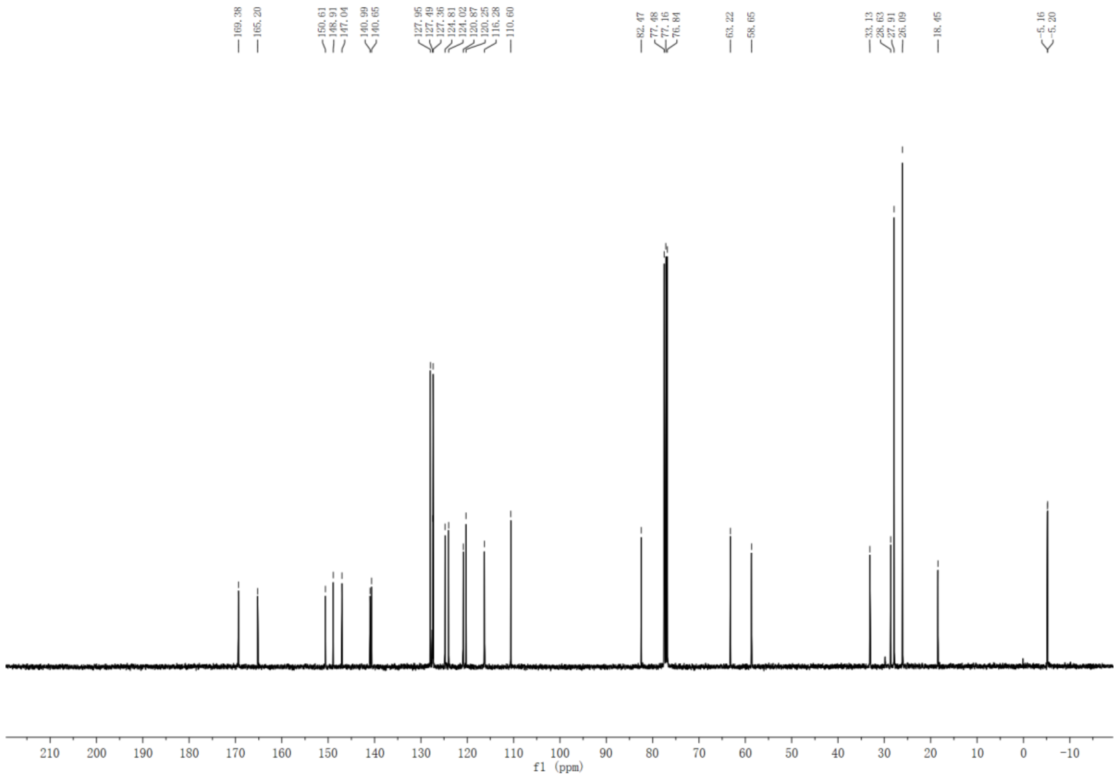

^{1}H NMR spectrum of **3r**

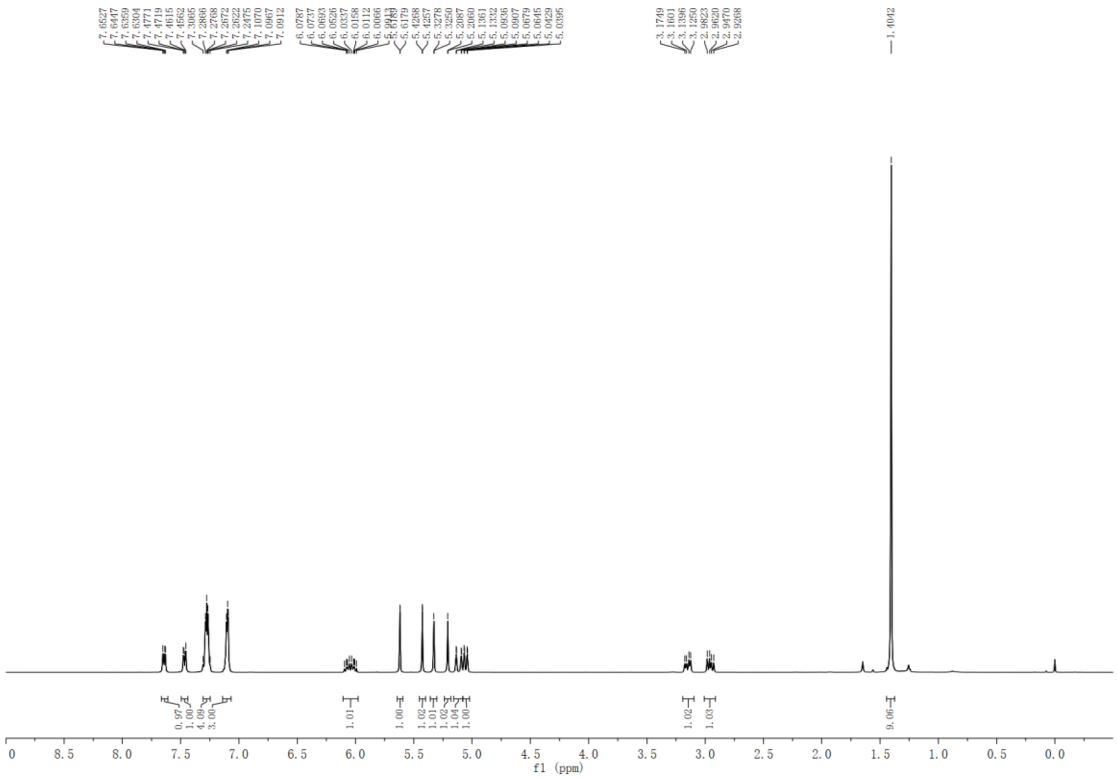

^{13}C NMR spectrum of **3r**

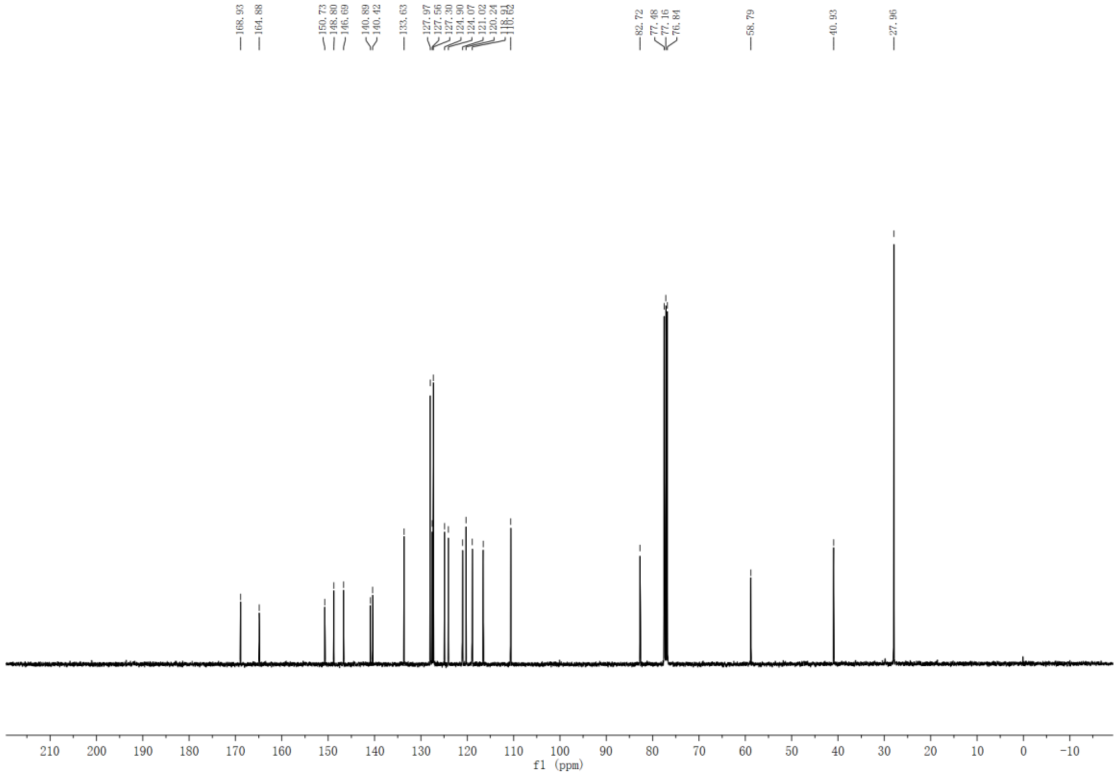

^{1}H NMR spectrum of **3s**

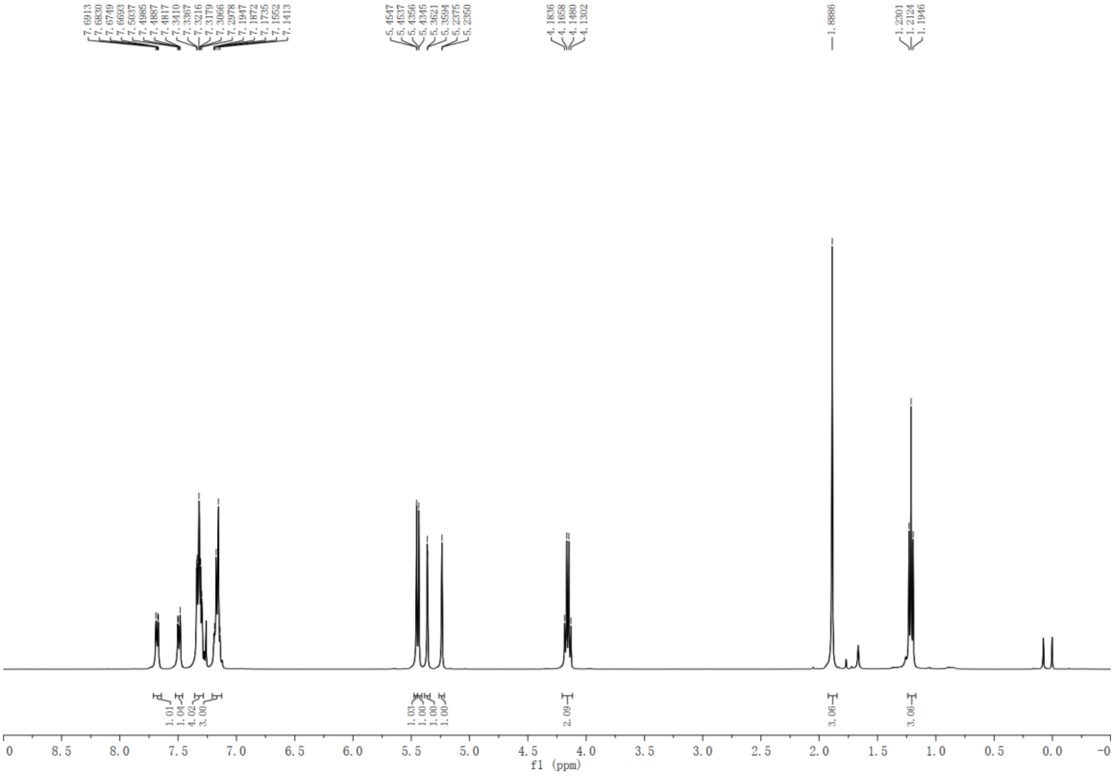

^{13}C NMR spectrum of **3s**

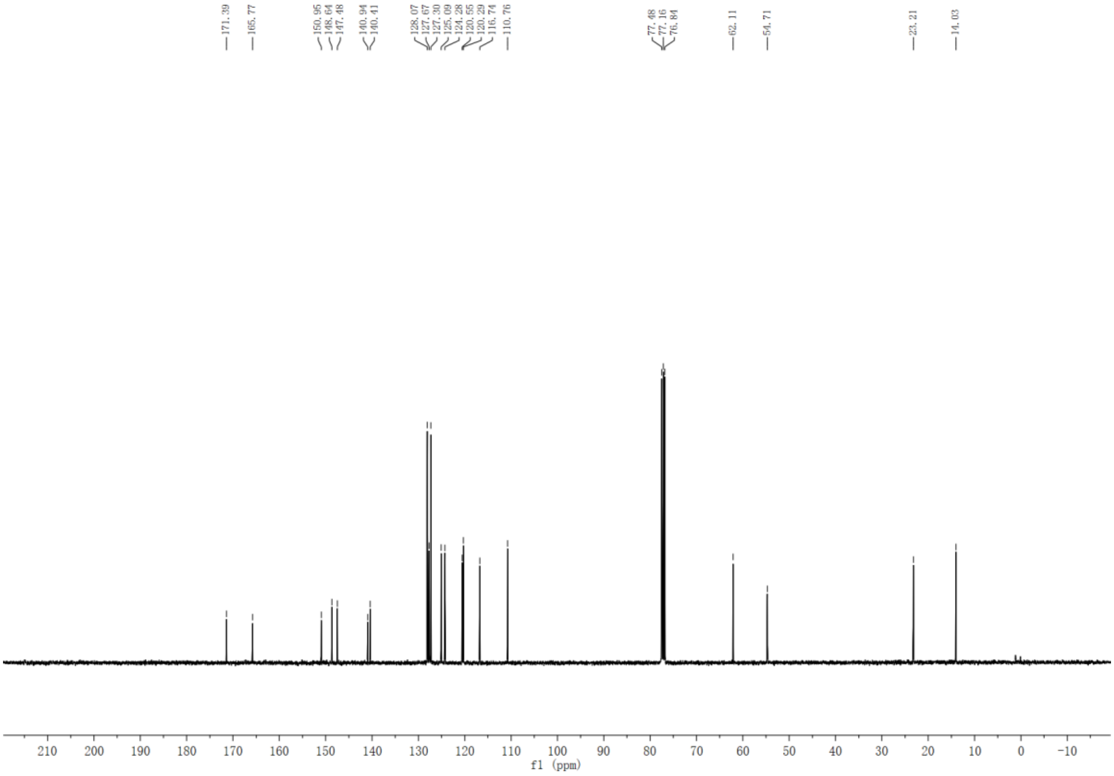

^{1}H NMR spectrum of **3t**

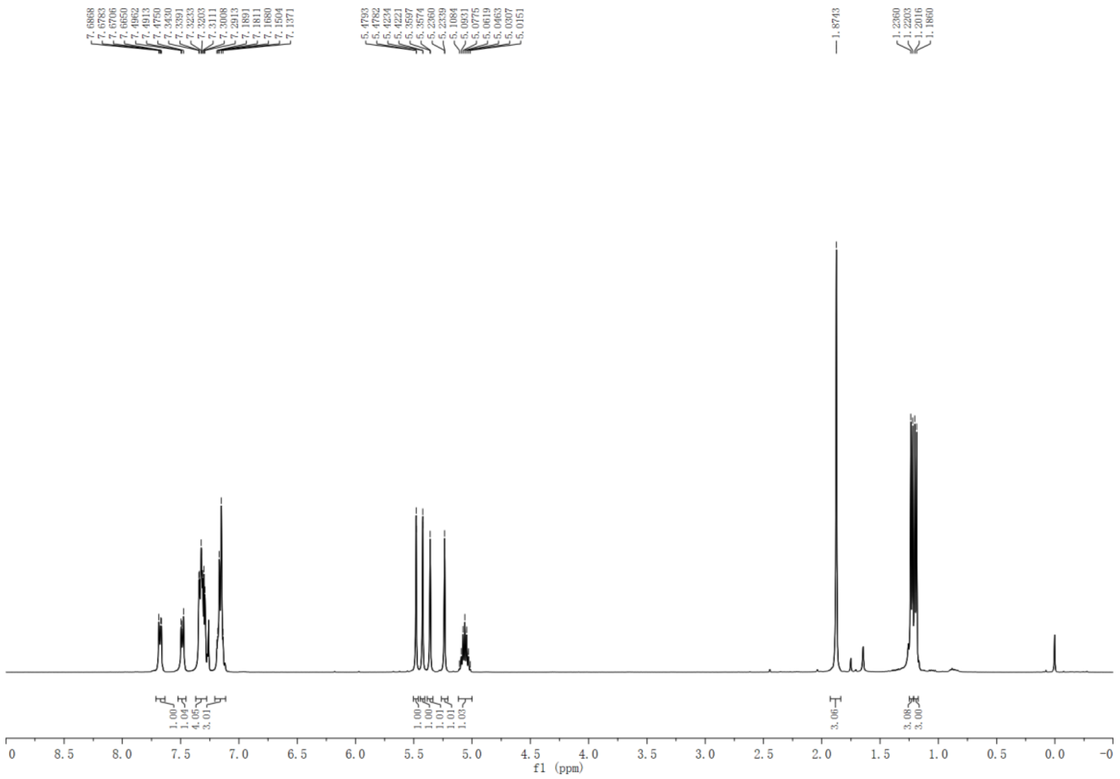

^{13}C NMR spectrum of **3t**

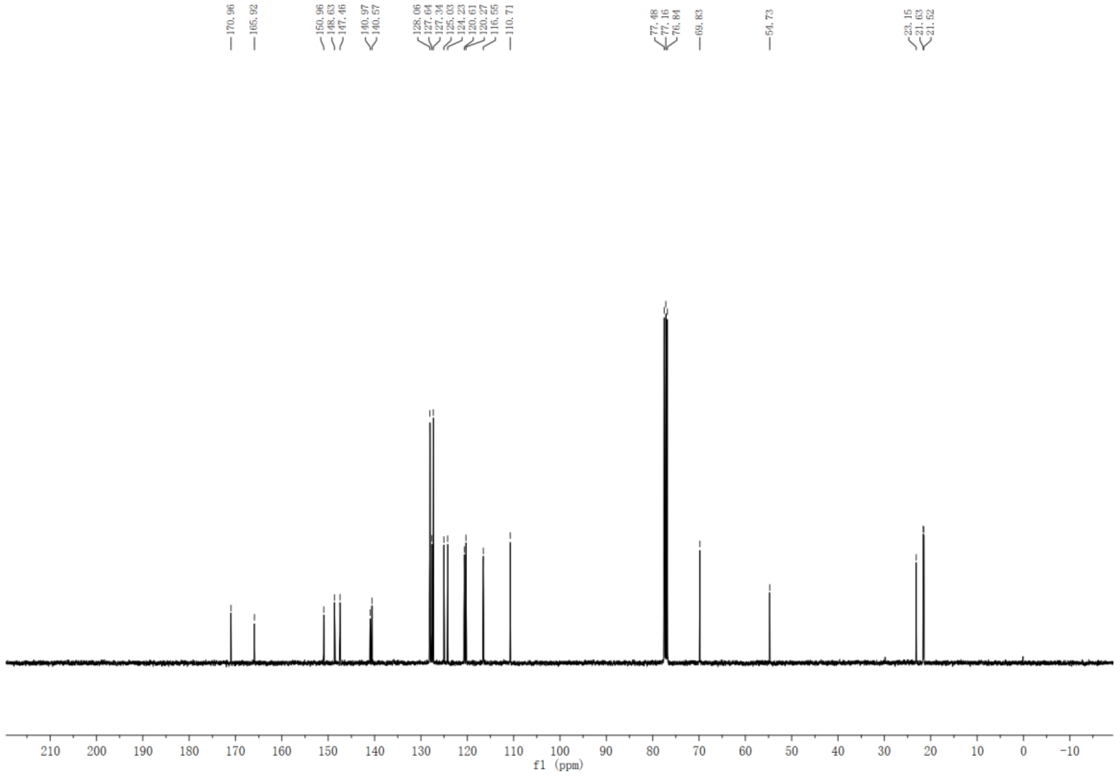

^{1}H NMR spectrum of **3u**

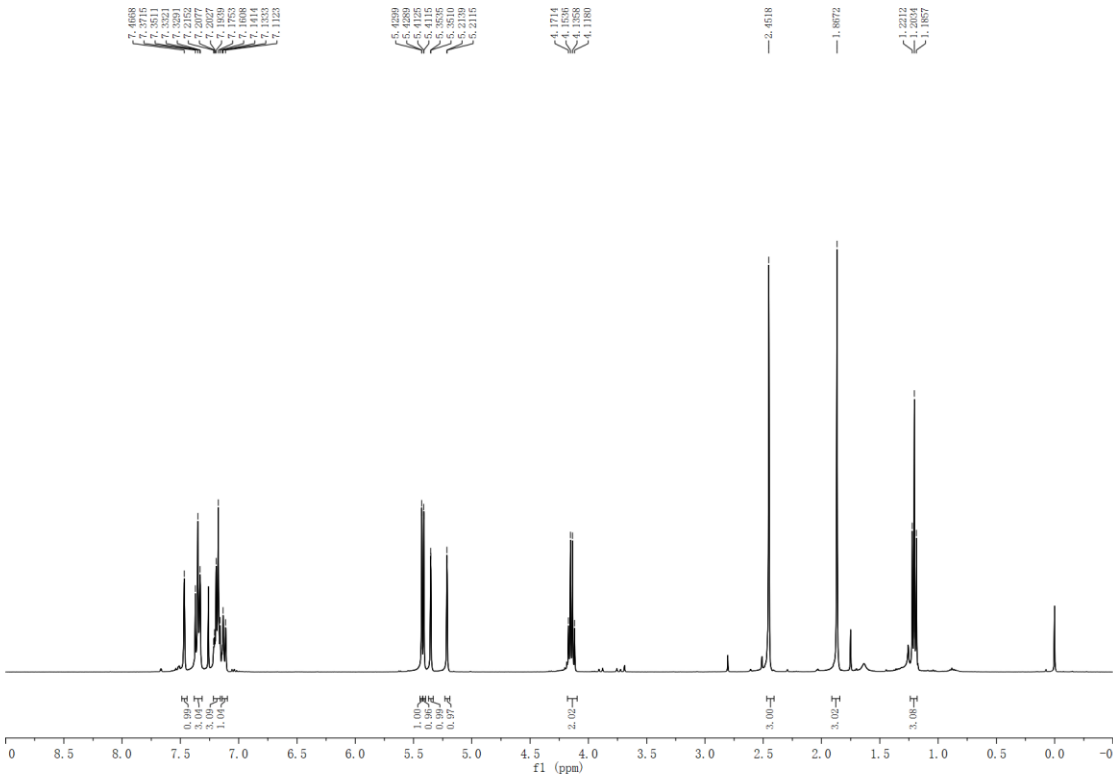

^{13}C NMR spectrum of **3u**

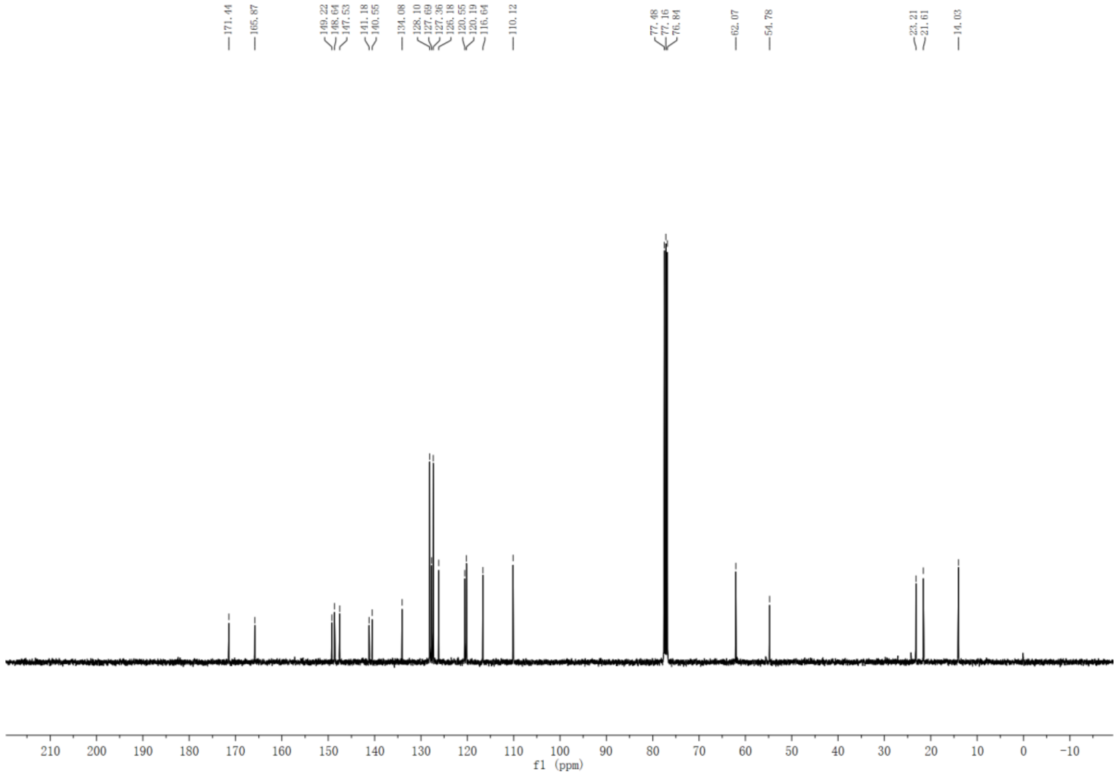

^{1}H NMR spectrum of **3v**

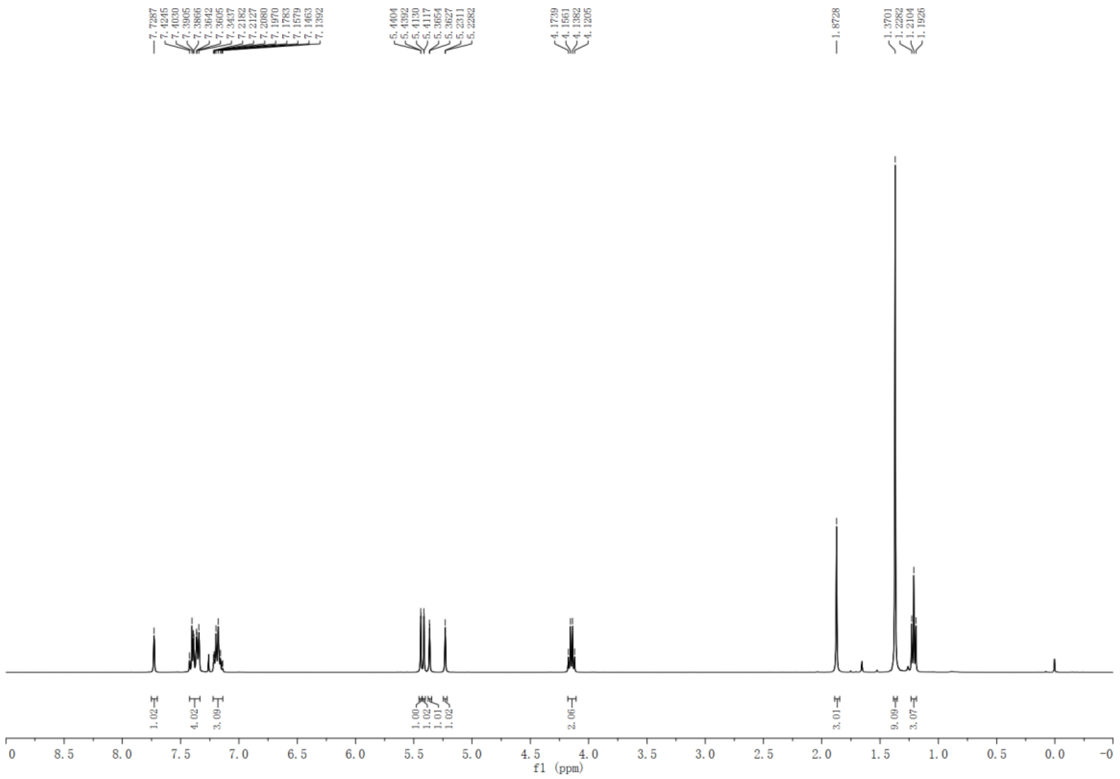

^{13}C NMR spectrum of **3v**

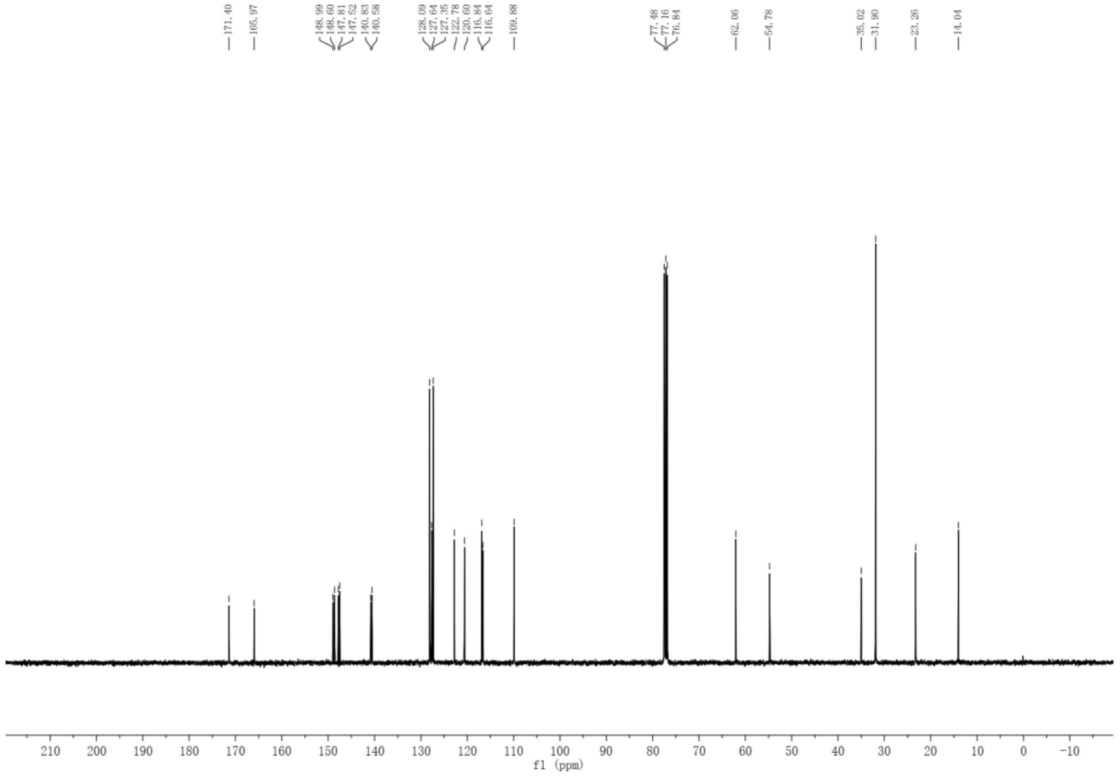

^{1}H NMR spectrum of **3w**

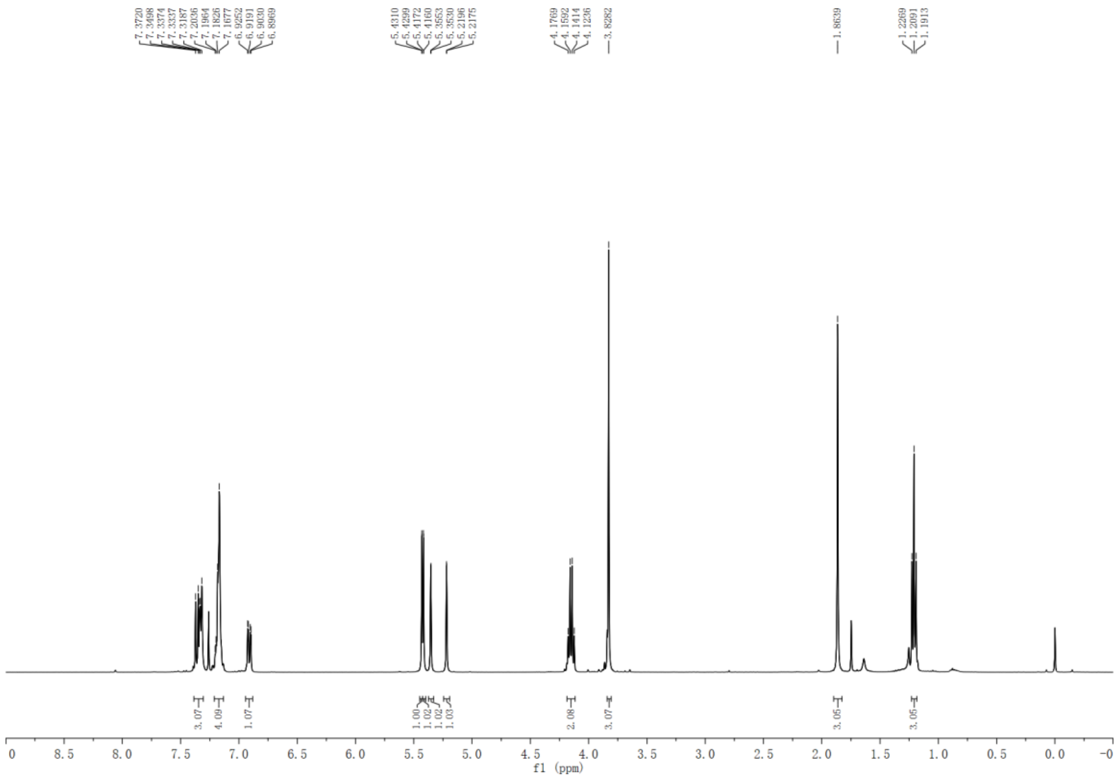

^{13}C NMR spectrum of **3w**

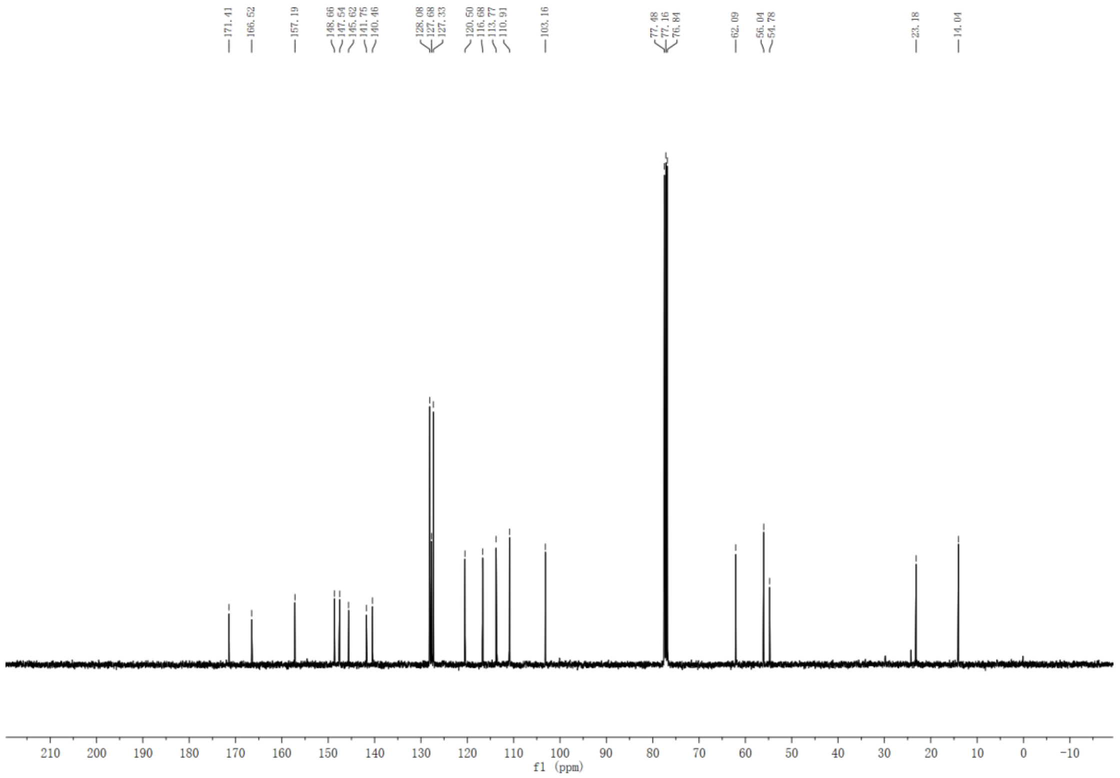

^{1}H NMR spectrum of **3x**

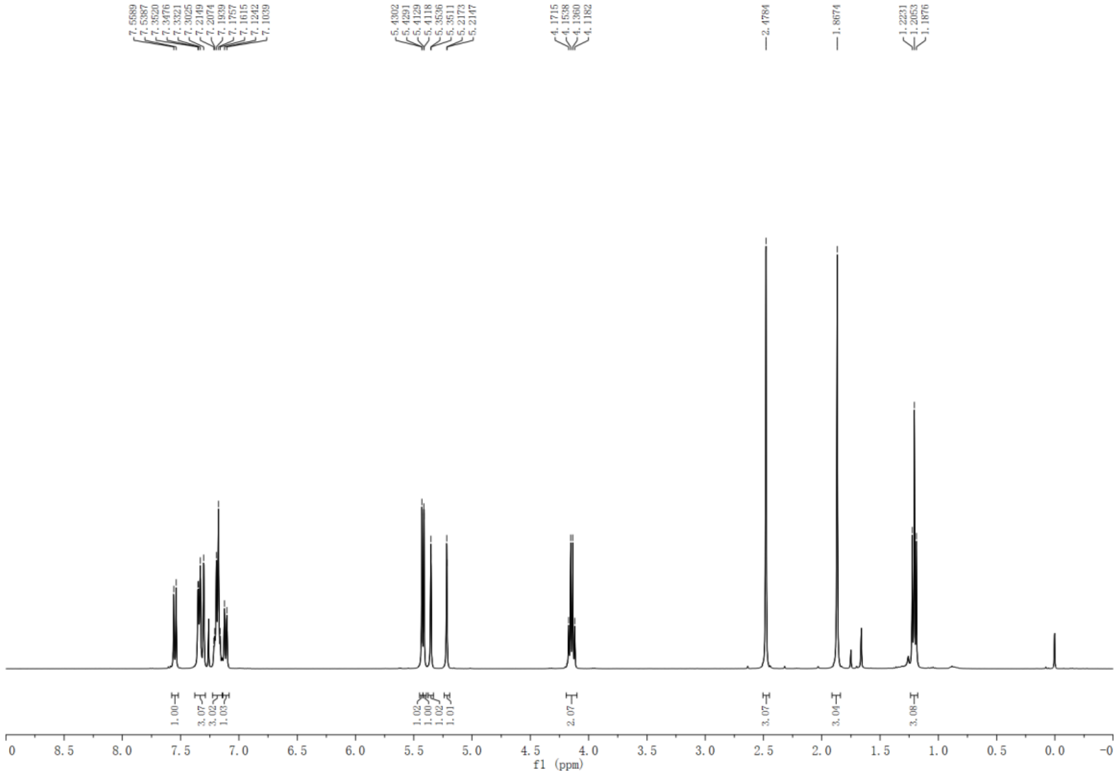

^{13}C NMR spectrum of **3x**

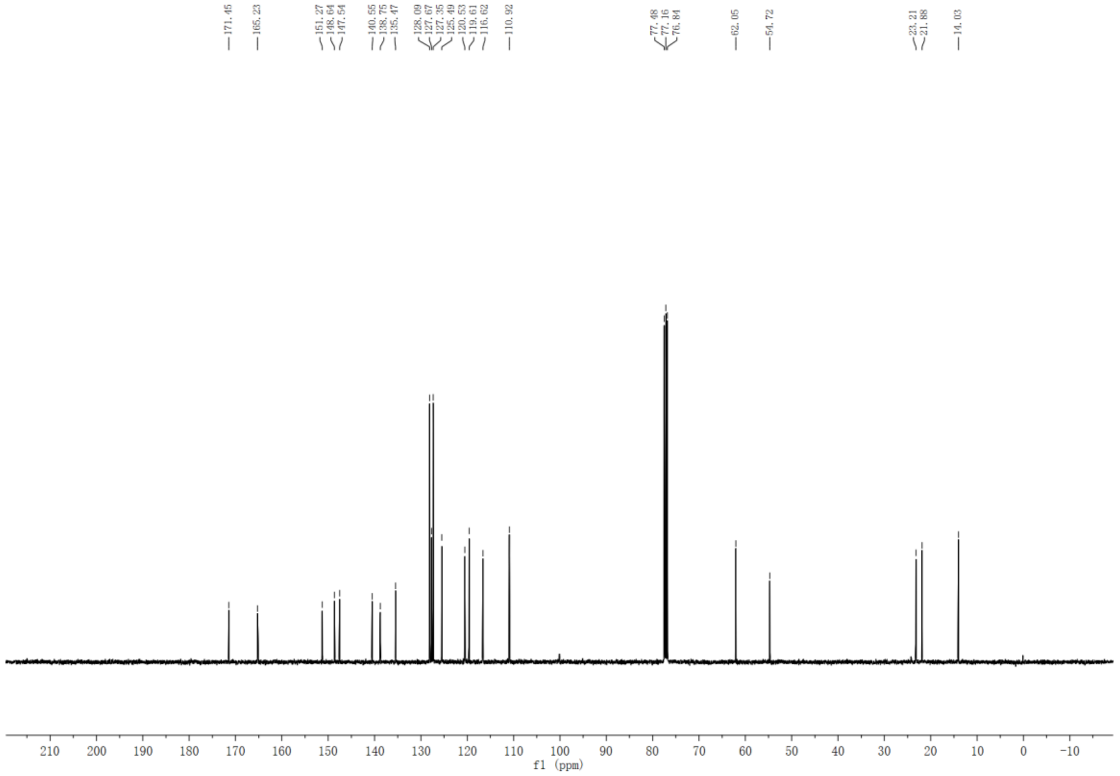

^{1}H NMR spectrum of **3y**

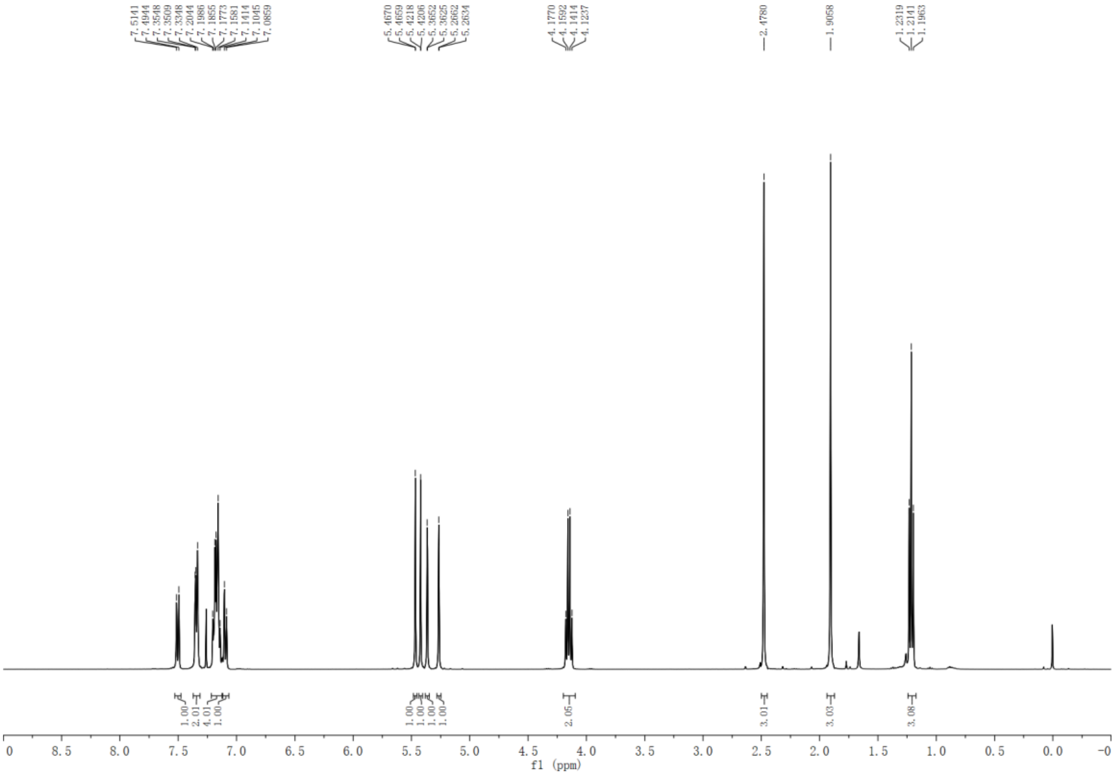

^{13}C NMR spectrum of **3y**

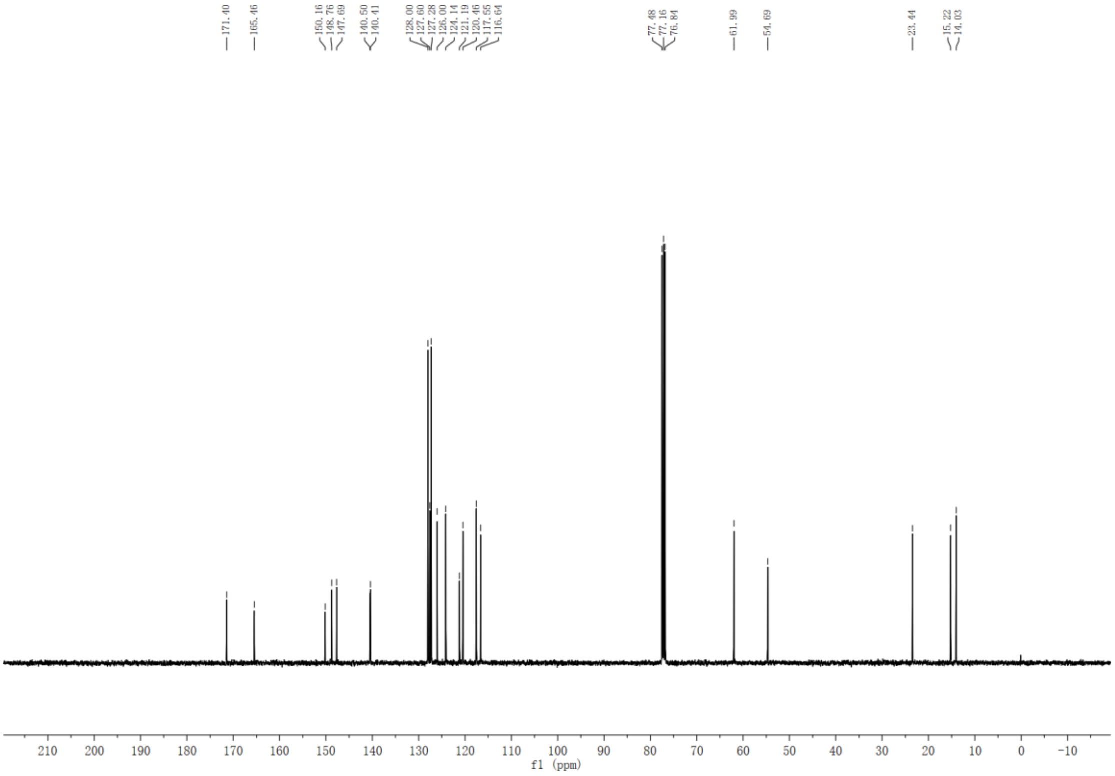

^{1}H NMR spectrum of **3z**

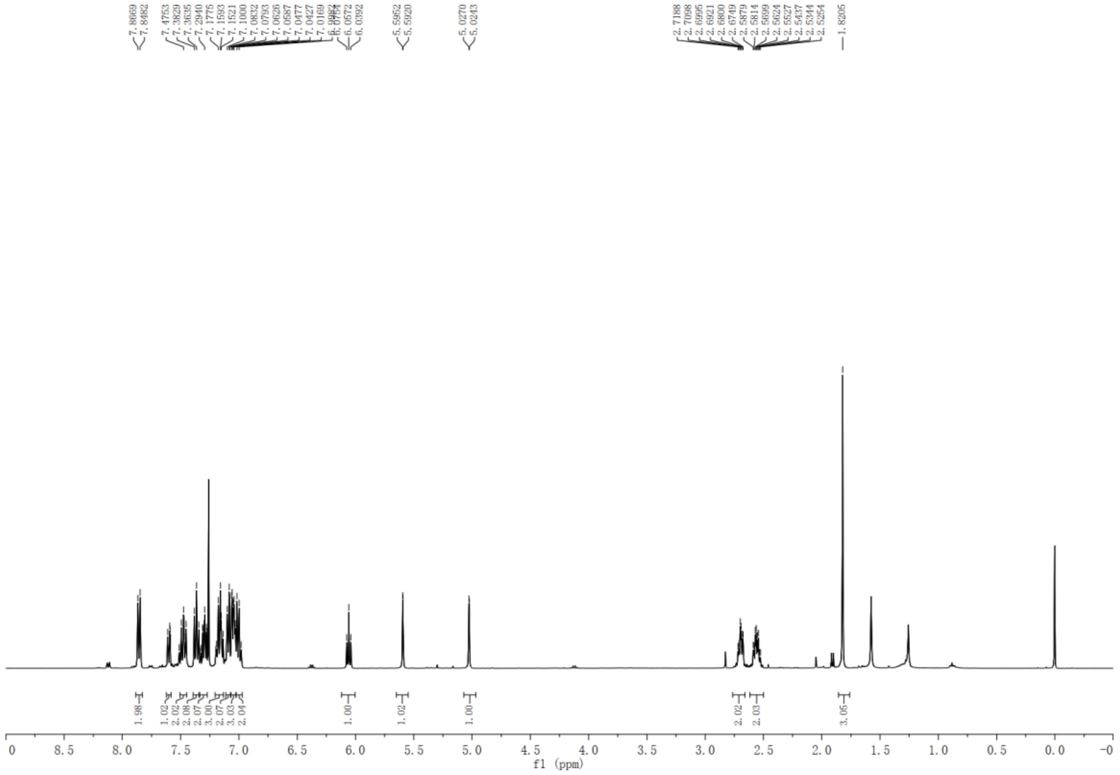

^{13}C NMR spectrum of **3z**

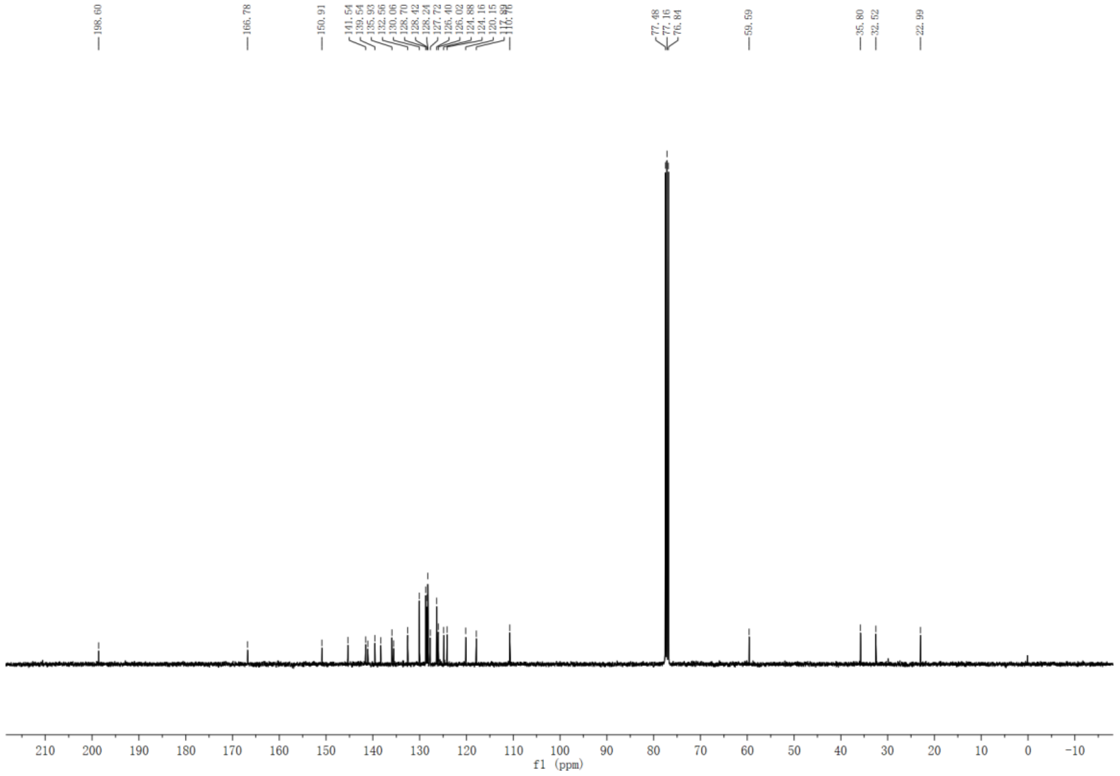

^{1}H NMR spectrum of **3aa**

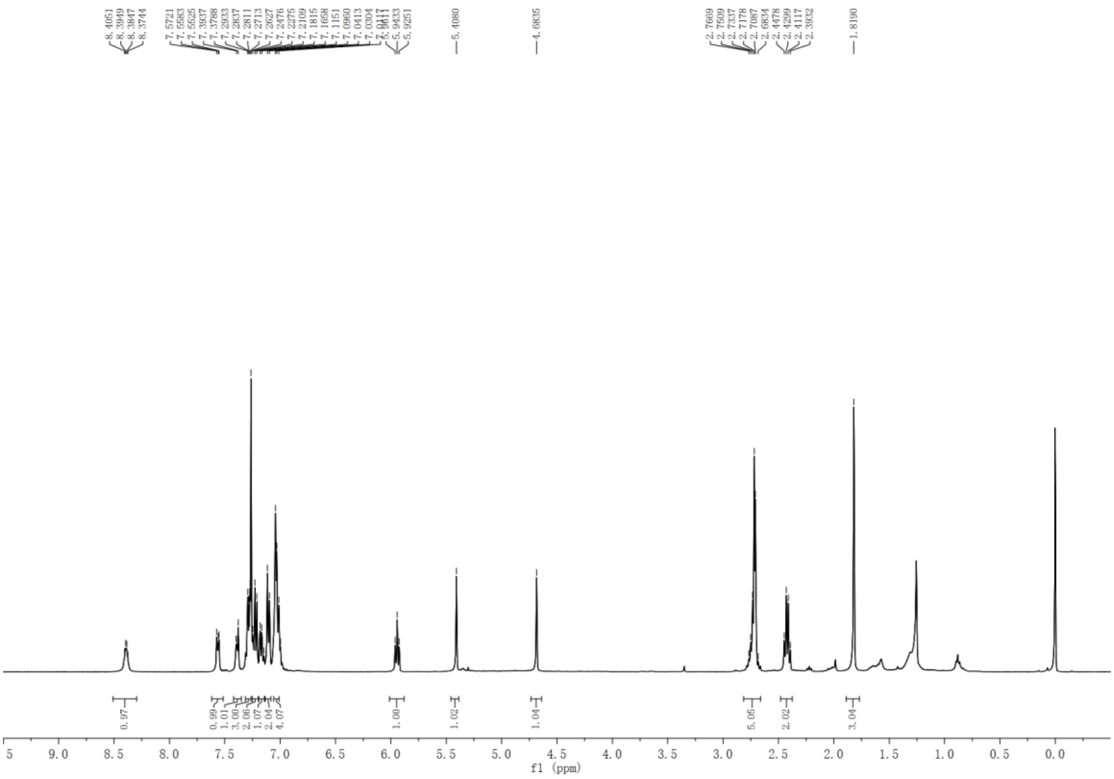

^{13}C NMR spectrum of **3aa**

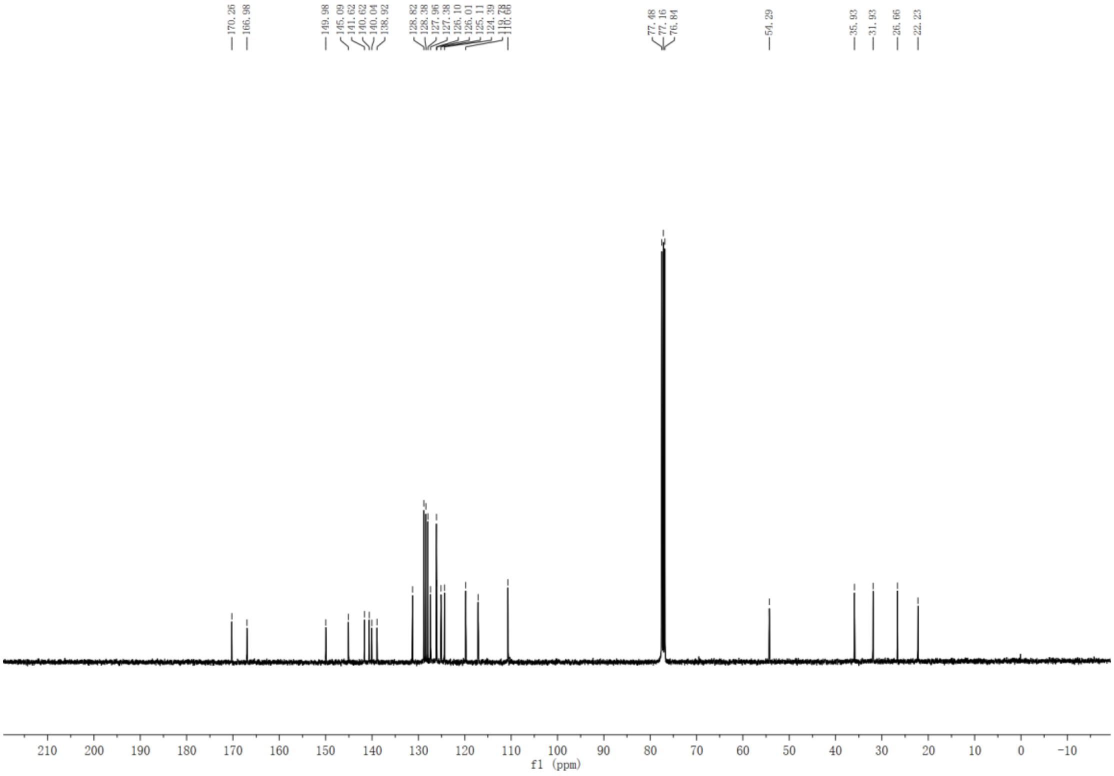

^{1}H NMR spectrum of **3ab**

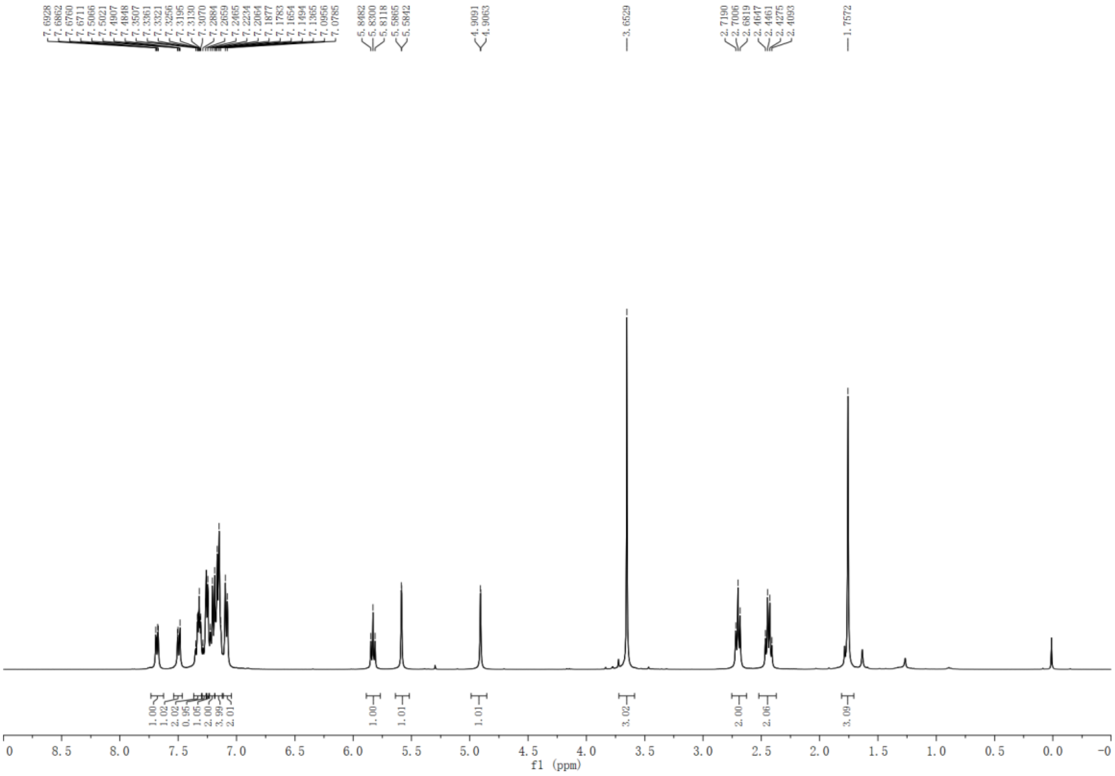

^{13}C NMR spectrum of **3ab**

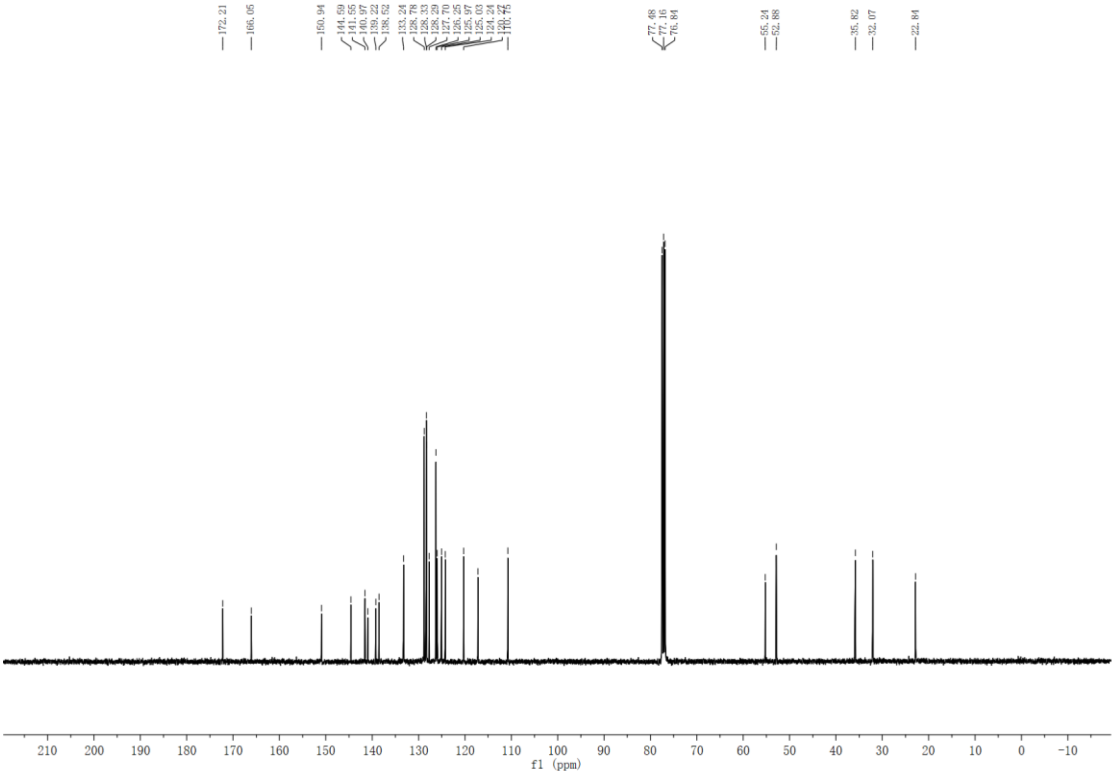

^{1}H NMR spectrum of **3ac**

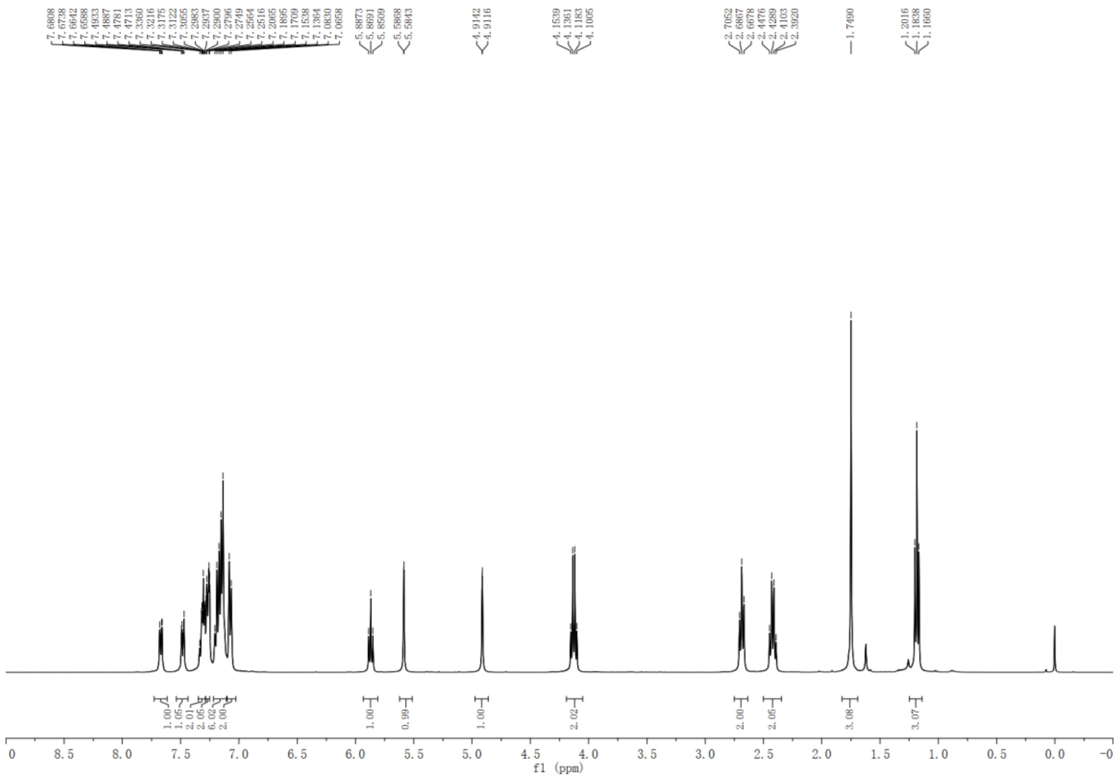

^{13}C NMR spectrum of **3ac**

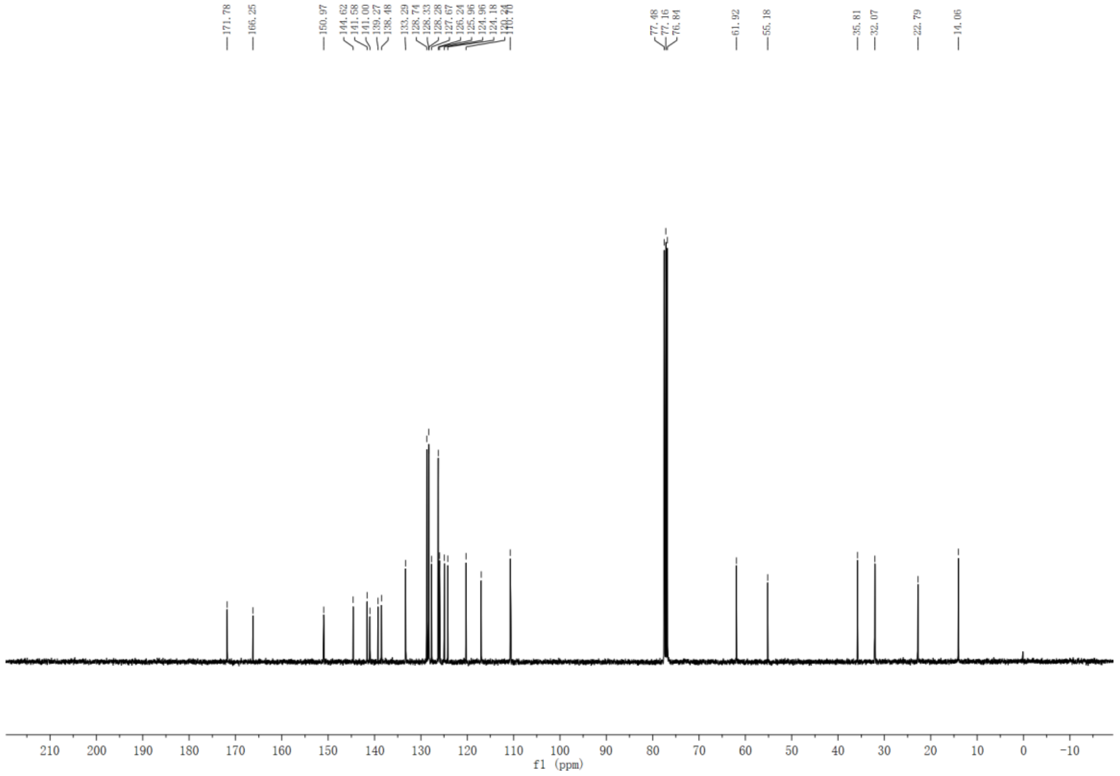

^{1}H NMR spectrum of **3ad**

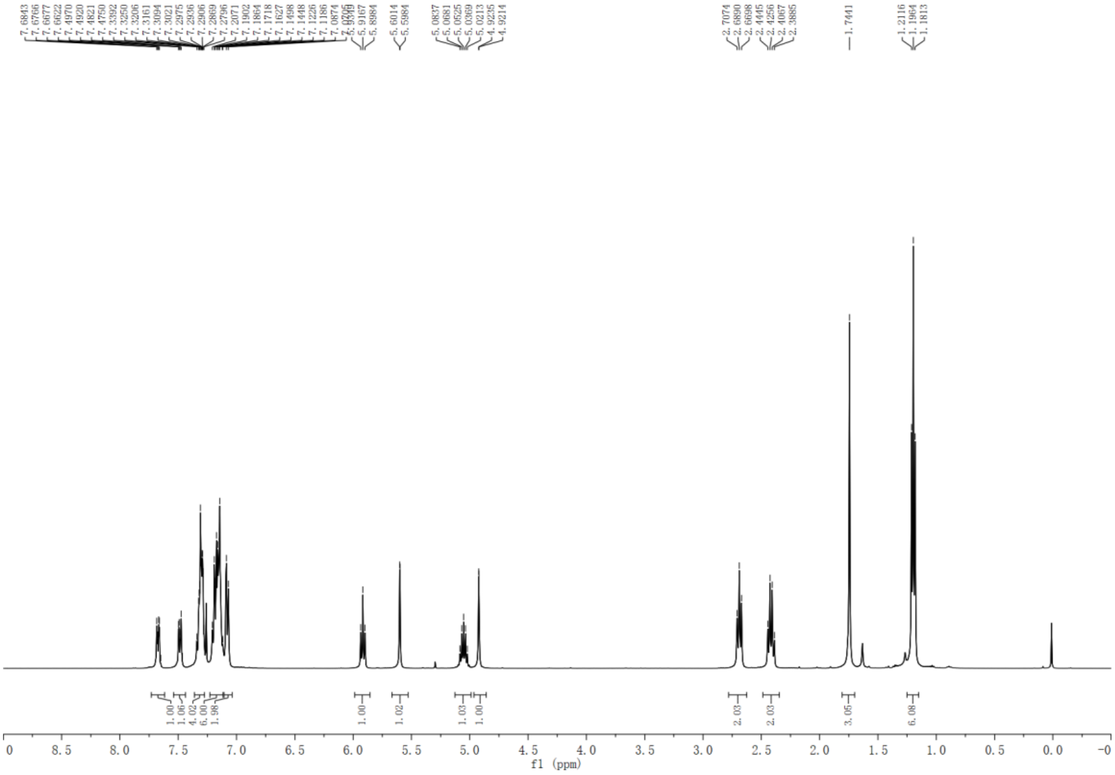

^{13}C NMR spectrum of **3ad**

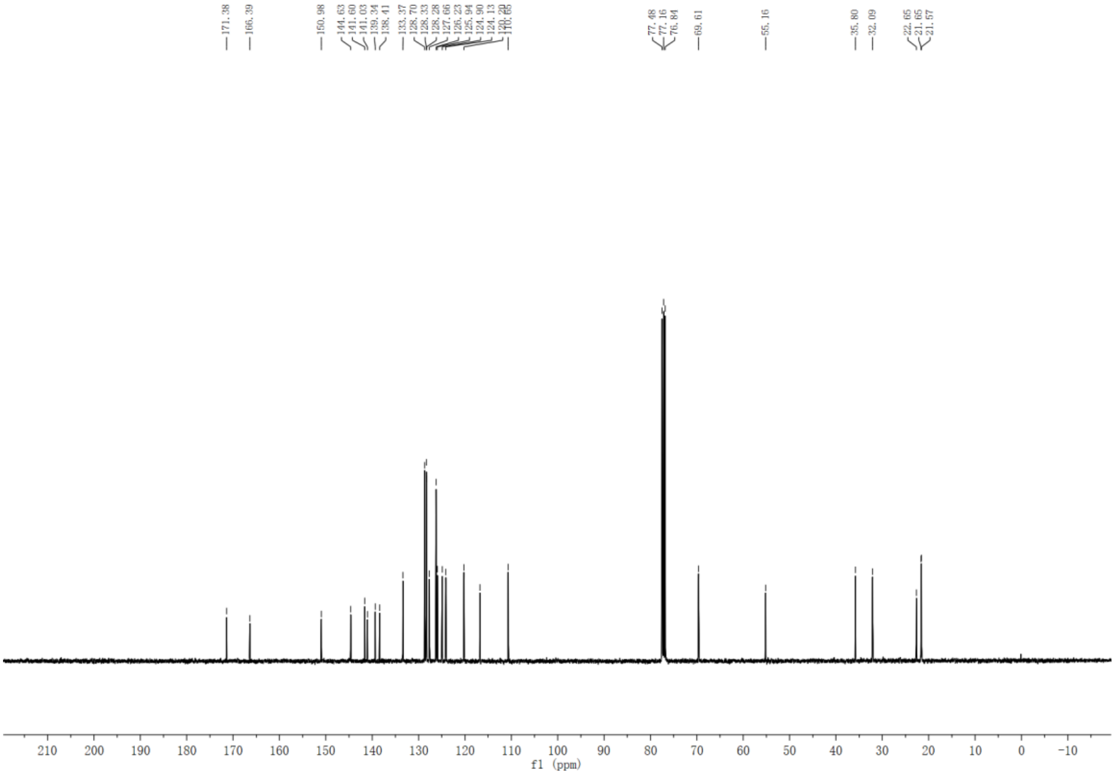

^{1}H NMR spectrum of **3ae**

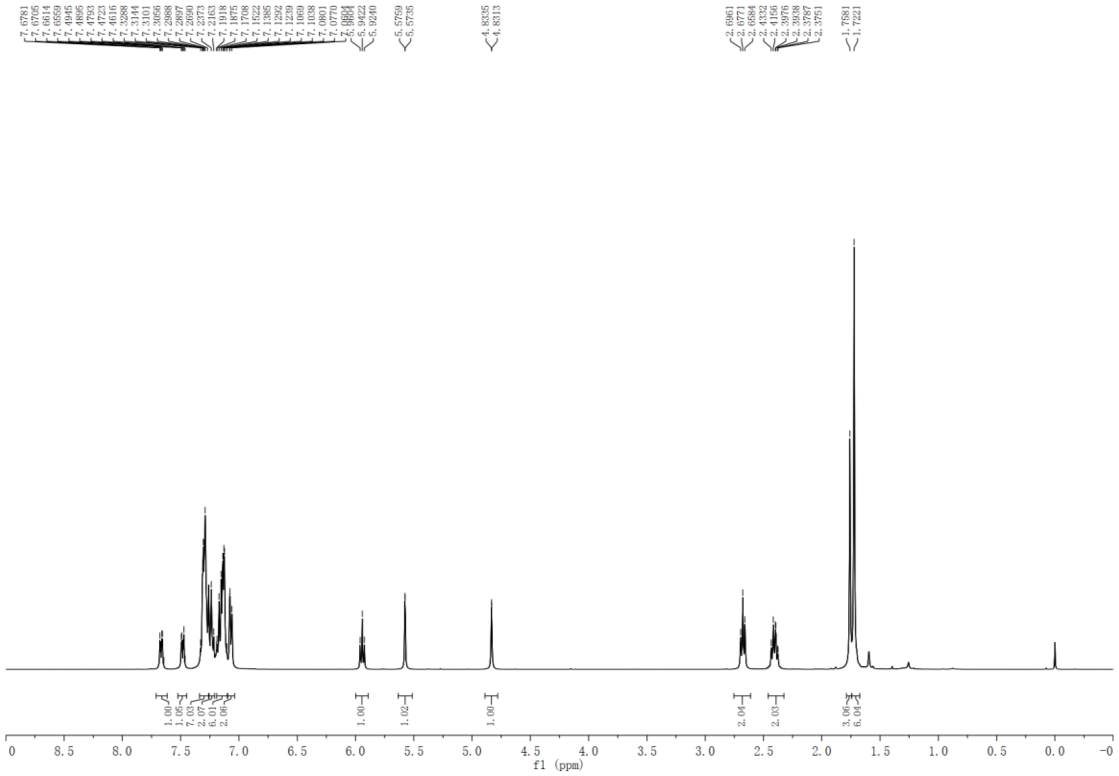

^{13}C NMR spectrum of **3ae**

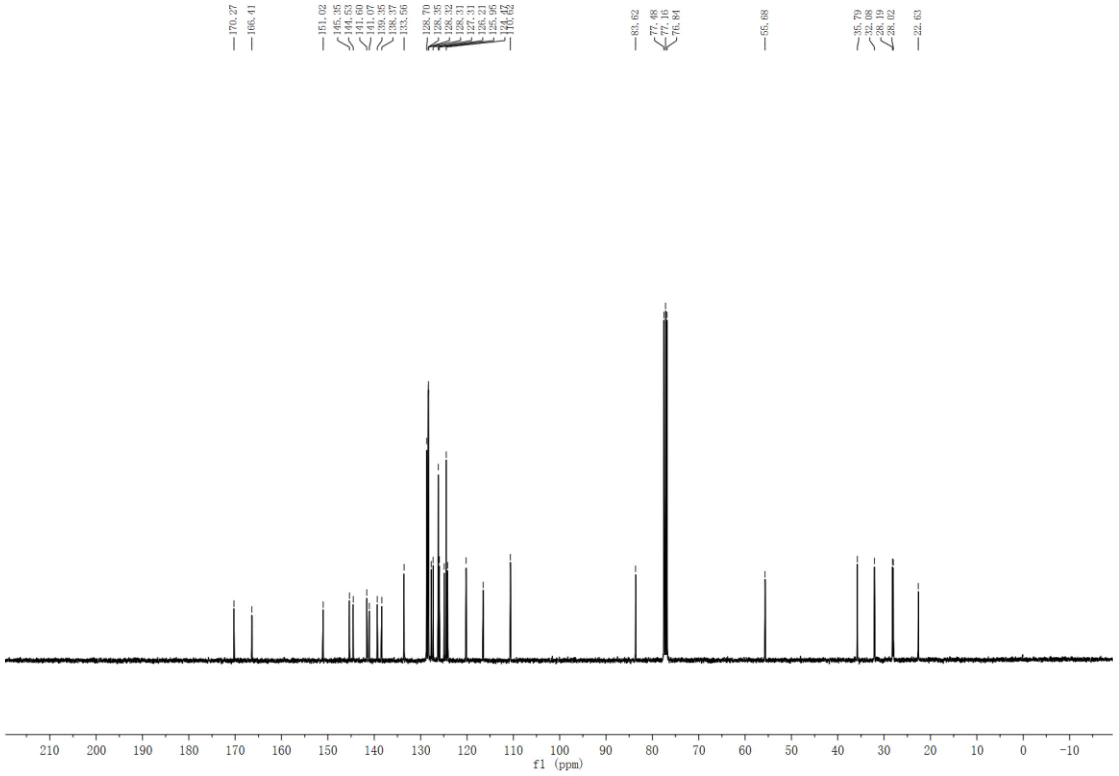

^{1}H NMR spectrum of **3af**

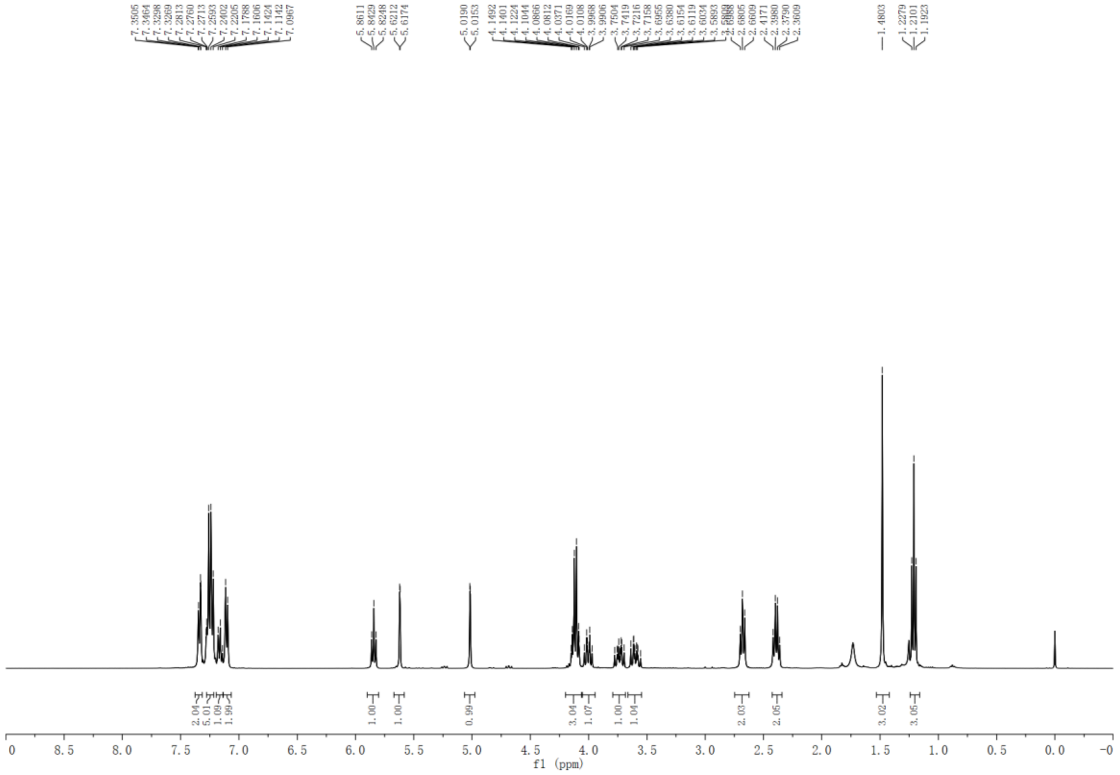

^{13}C NMR spectrum of **3af**

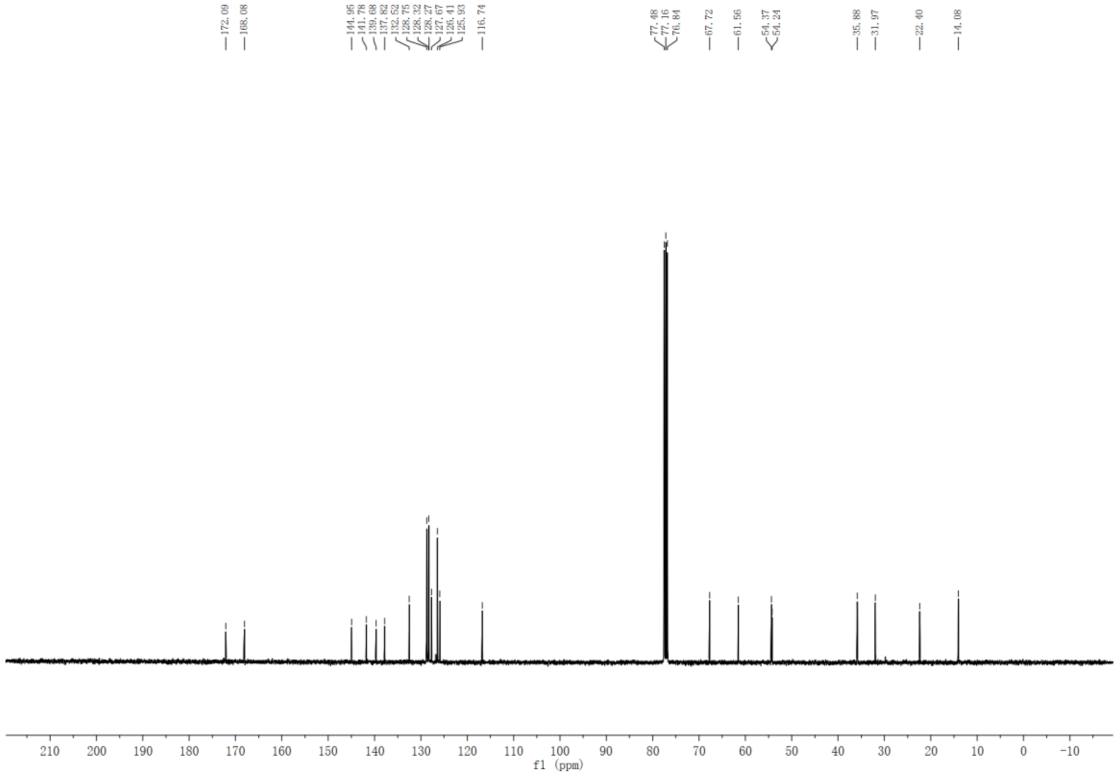

^{1}H NMR spectrum of crude **3ag** (Z/E = 25:1)

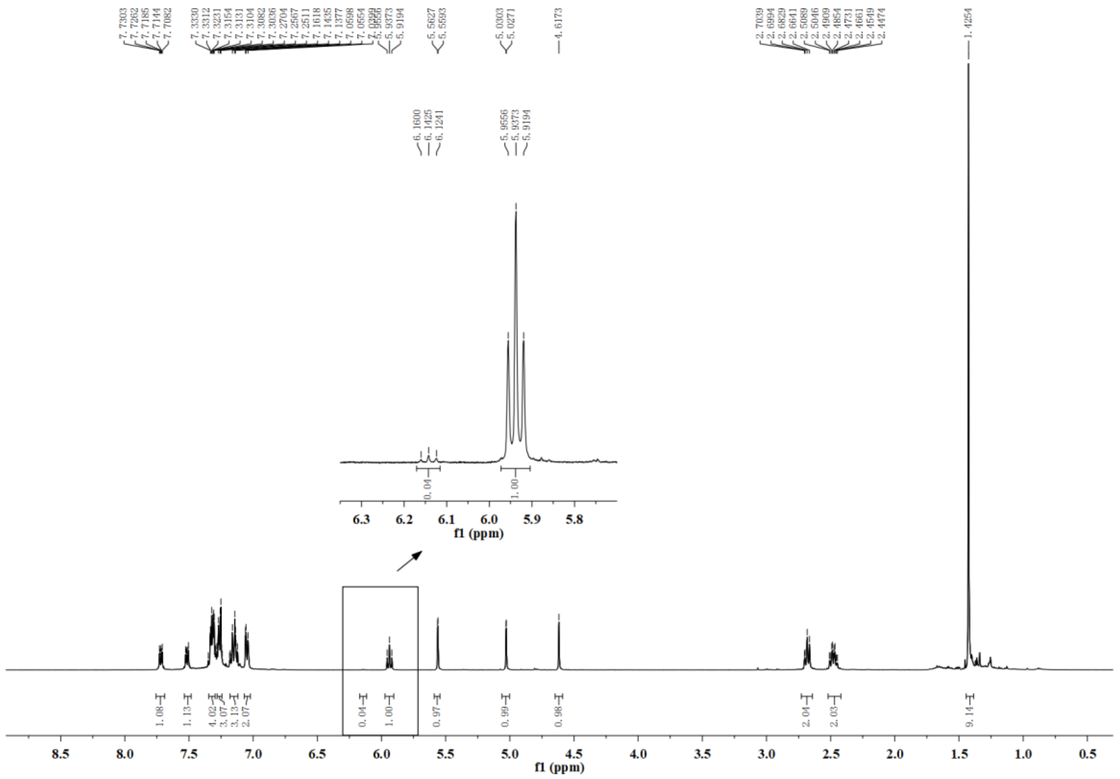

^{1}H NMR spectrum of **3ag**

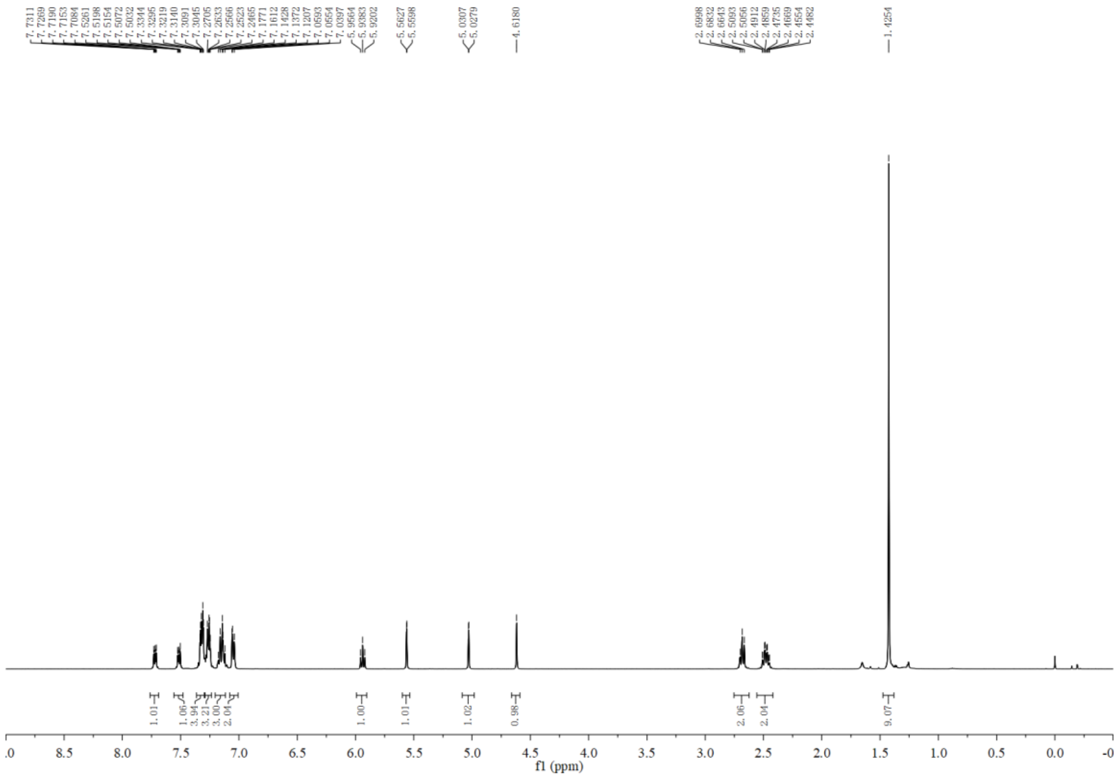

^{13}C NMR spectrum of **3ag**

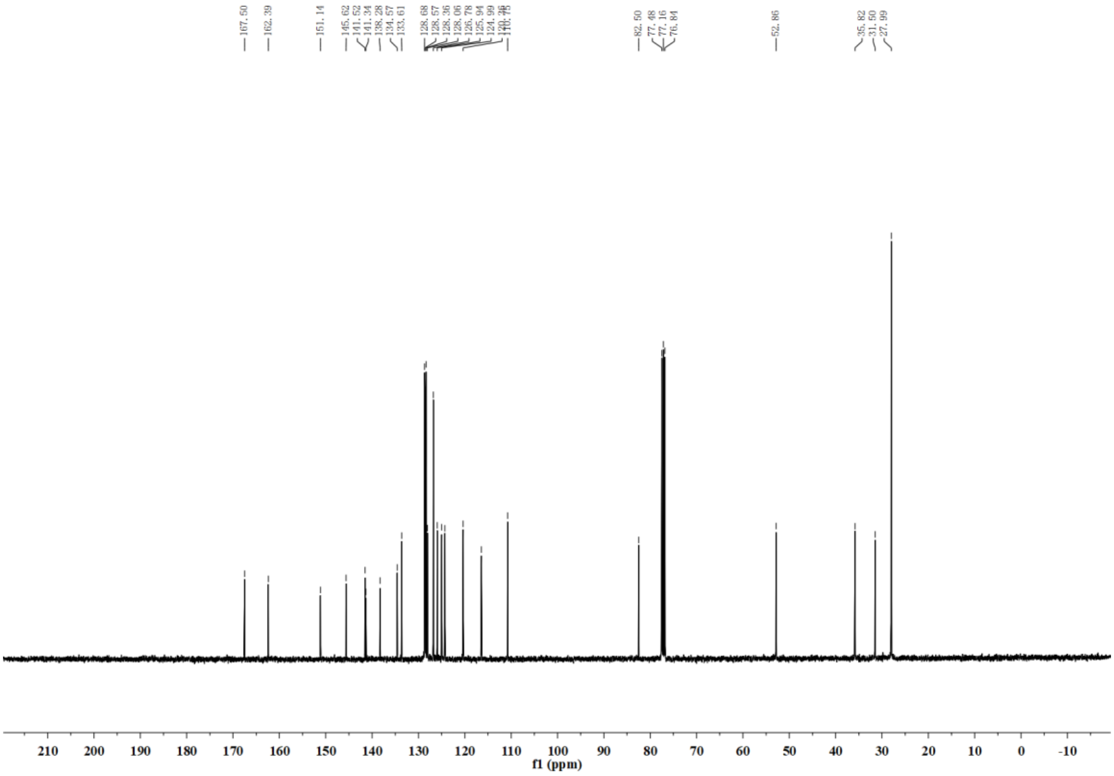

NOESY spectrum of **3ag**

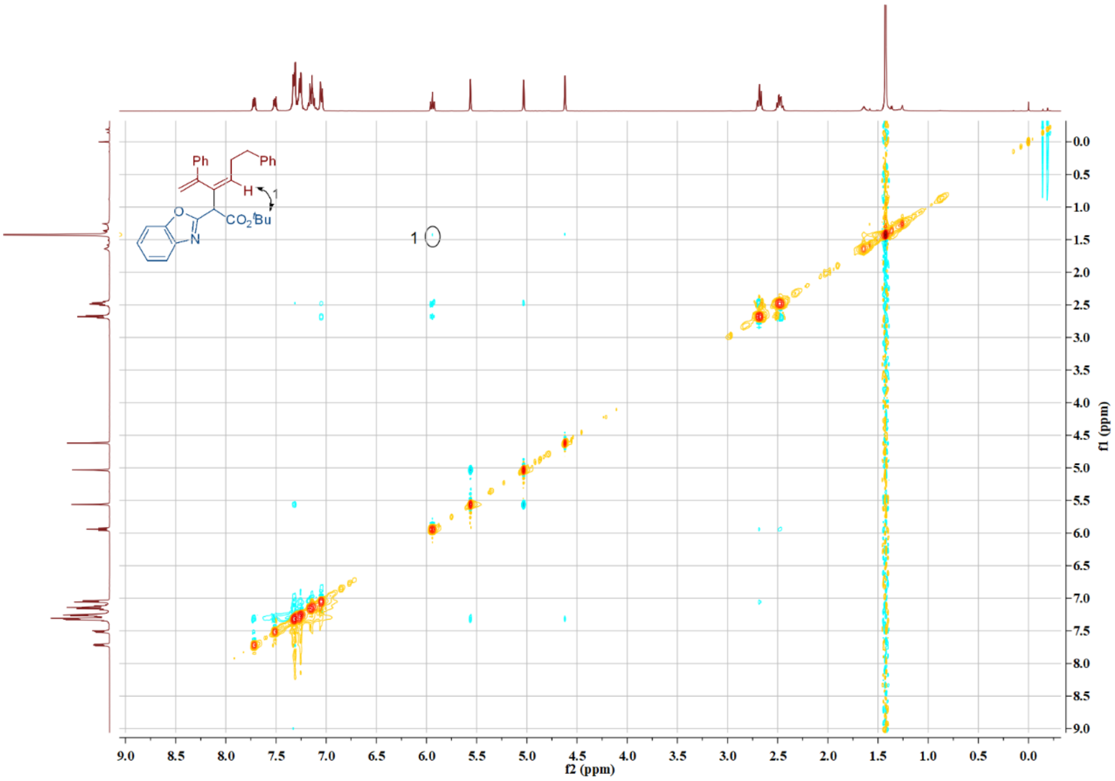

^{1}H NMR spectrum of **5** (E/Z = 3.8:1)

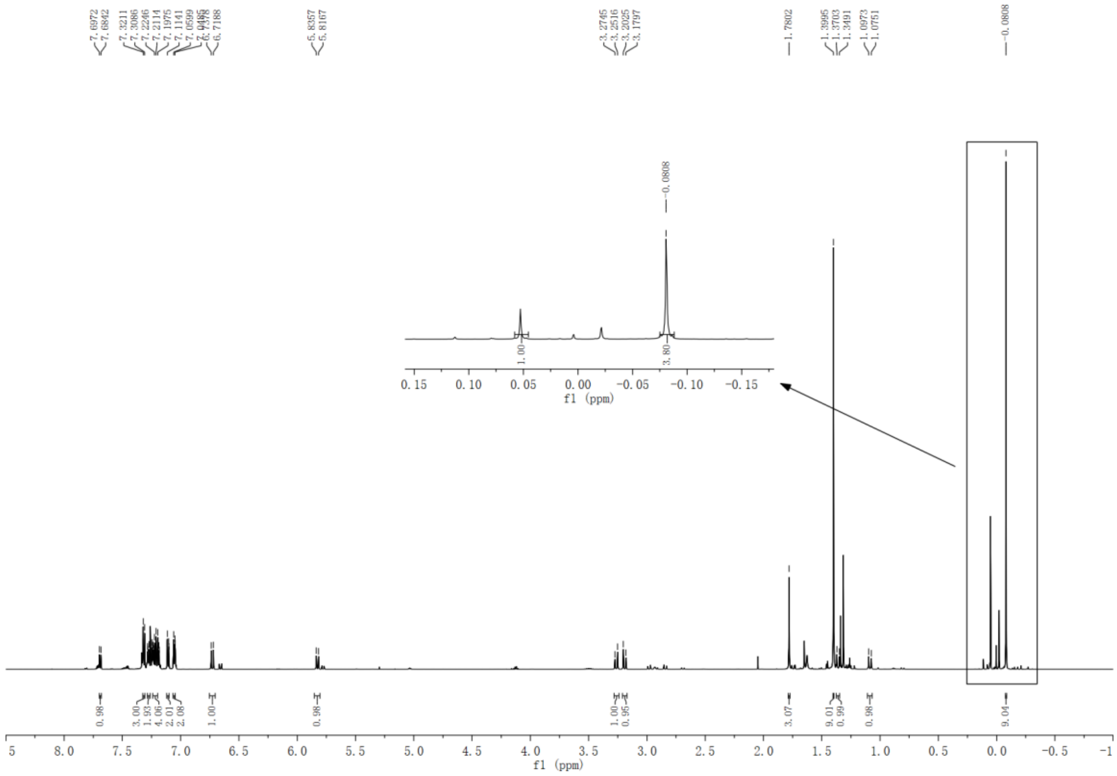

^{1}H NMR spectrum of **5** (major)

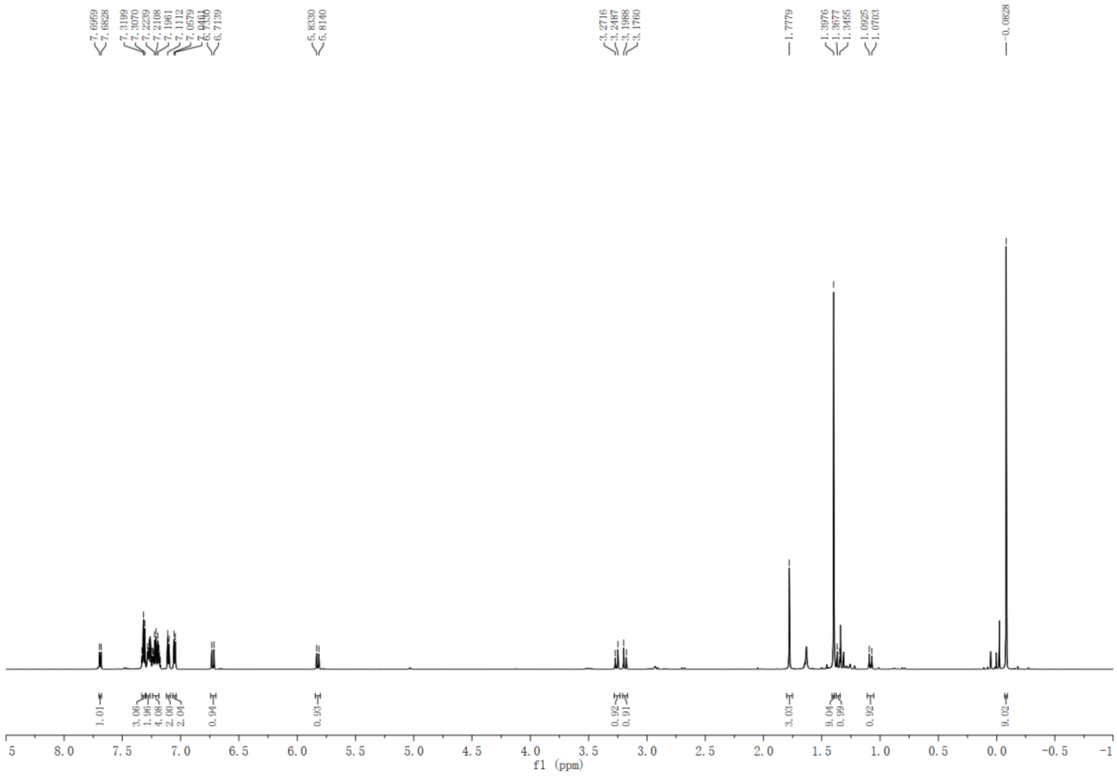

^{13}C NMR spectrum of **5** (major)

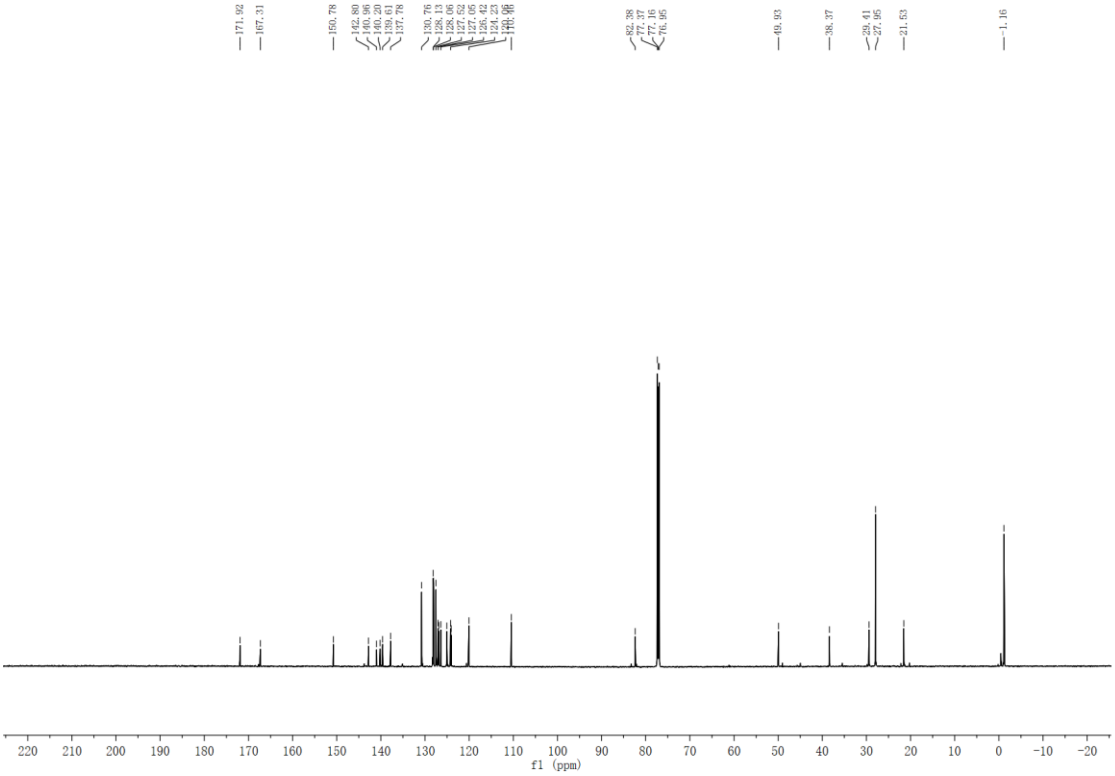

NOESY spectrum of **5** (major)

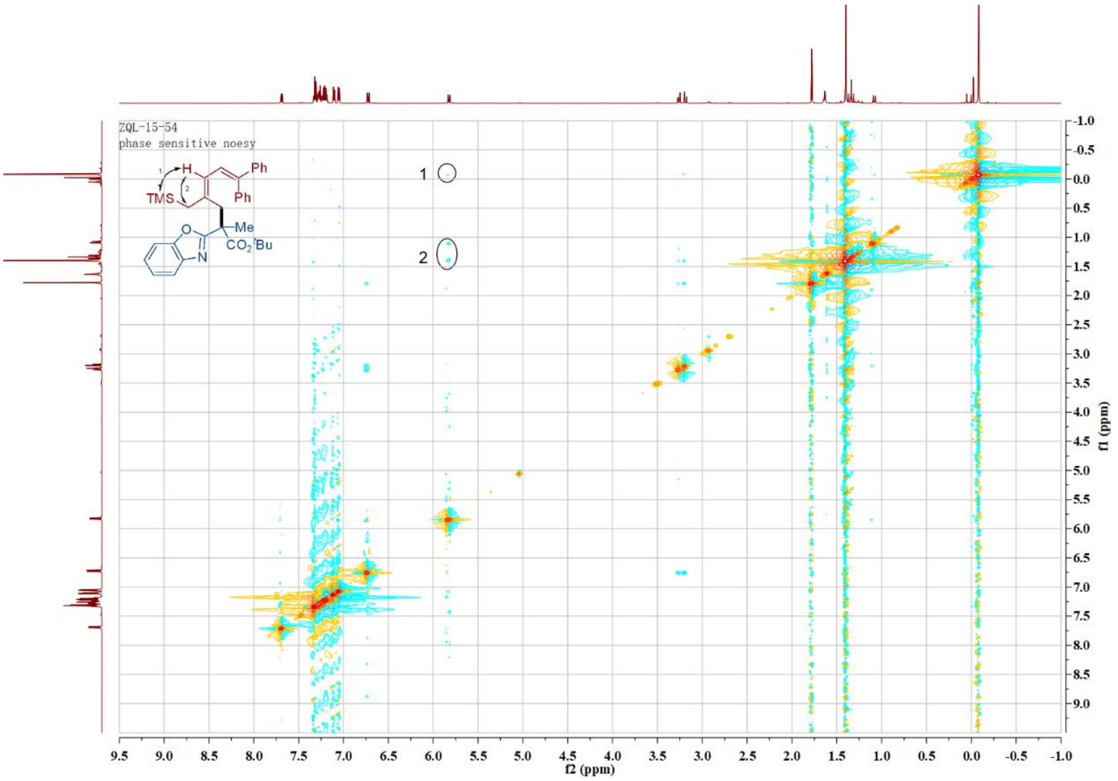

The NOESY spectrum of **5** (major) suggests that major product of **5** is in the *E* configuration.

^{1}H NMR spectrum of **6** (E/Z = 2.4:1)

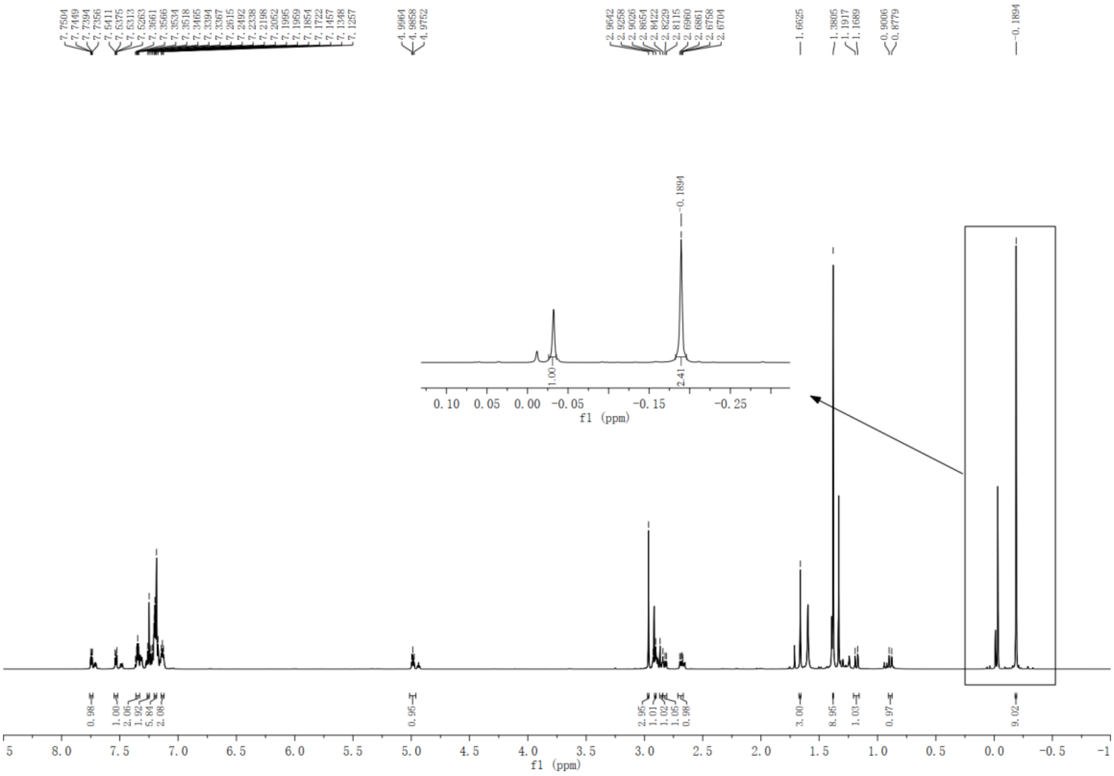

^{1}H NMR spectrum of **6** (major)

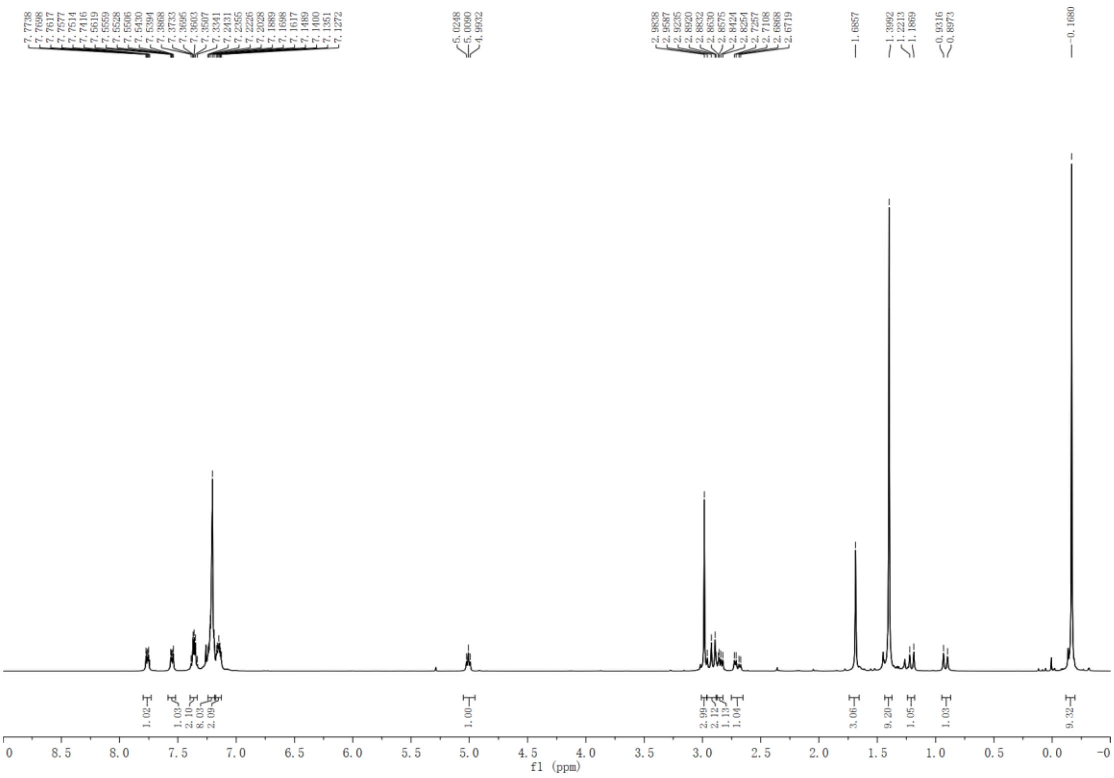

^{13}C NMR spectrum of **6** (major)

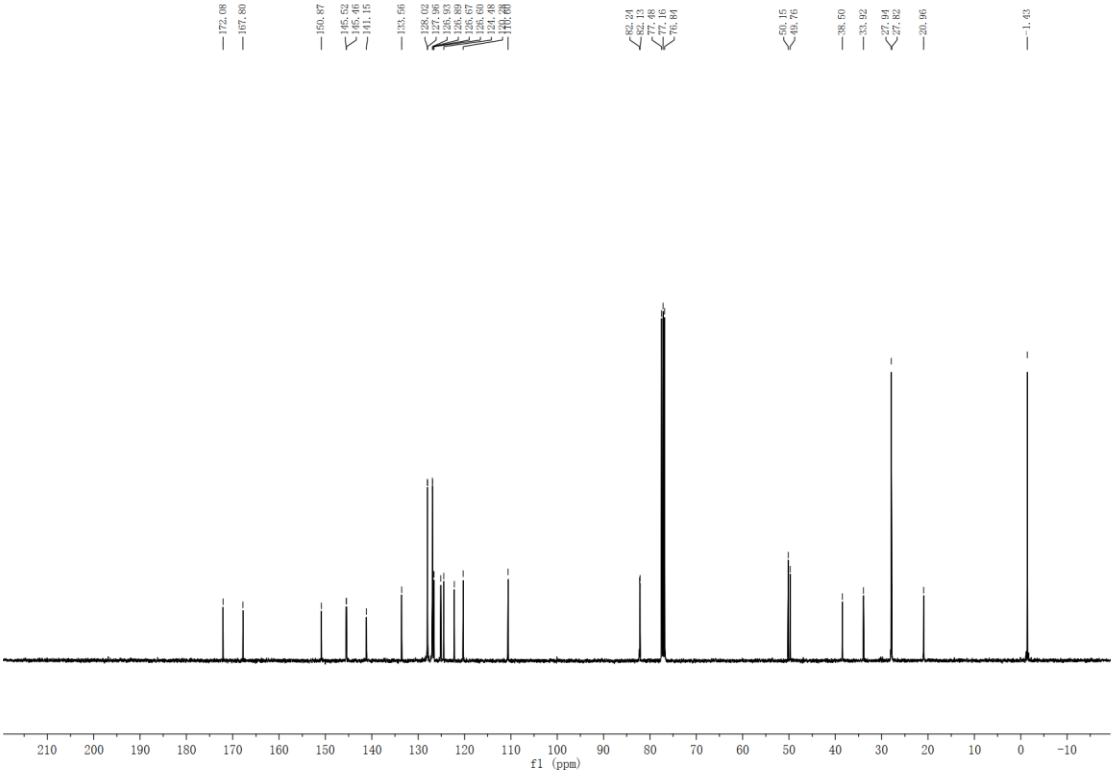

NOESY spectrum of **6** (major)

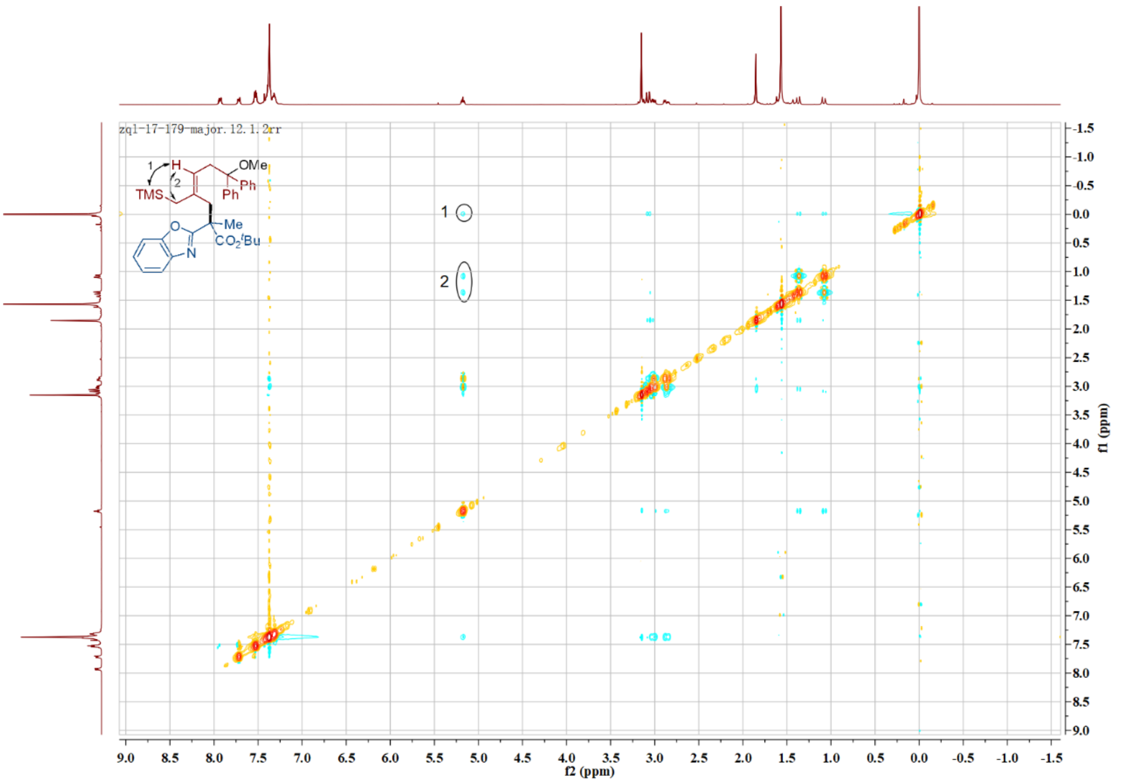

The NOESY spectrum of **6** (major) suggests that major product of **6** is in the *E* configuration.

^{1}H NMR spectrum of **8a**

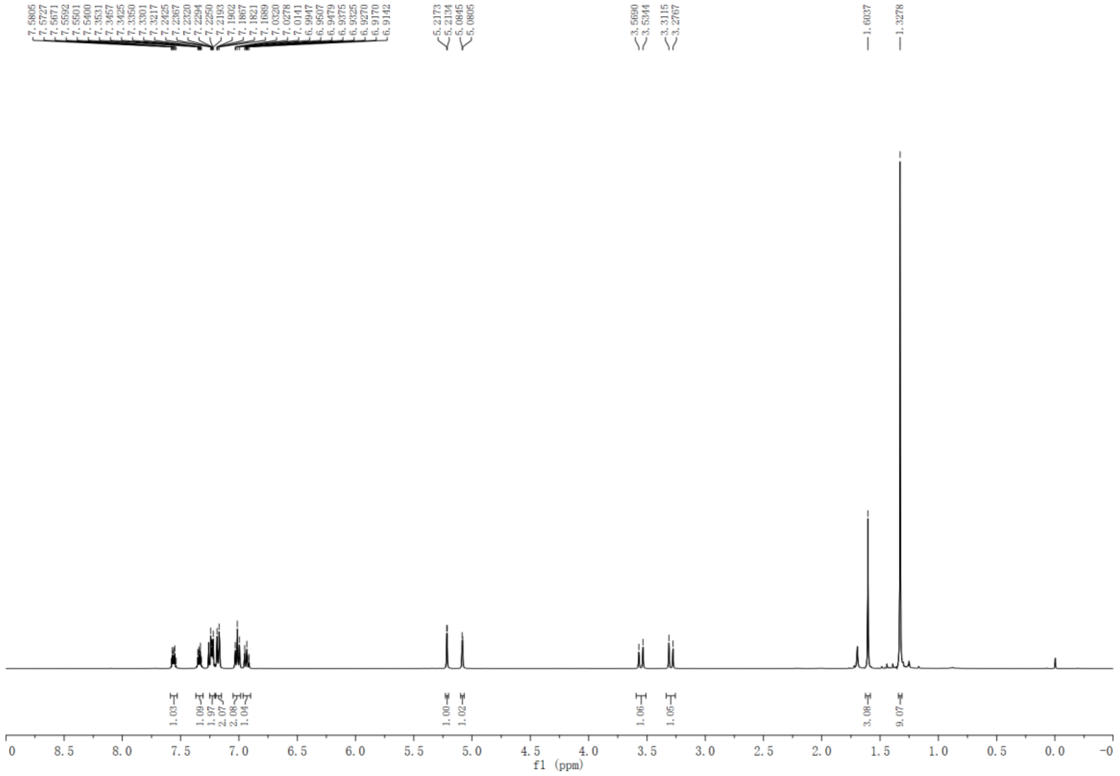

^{13}C NMR spectrum of **8a**

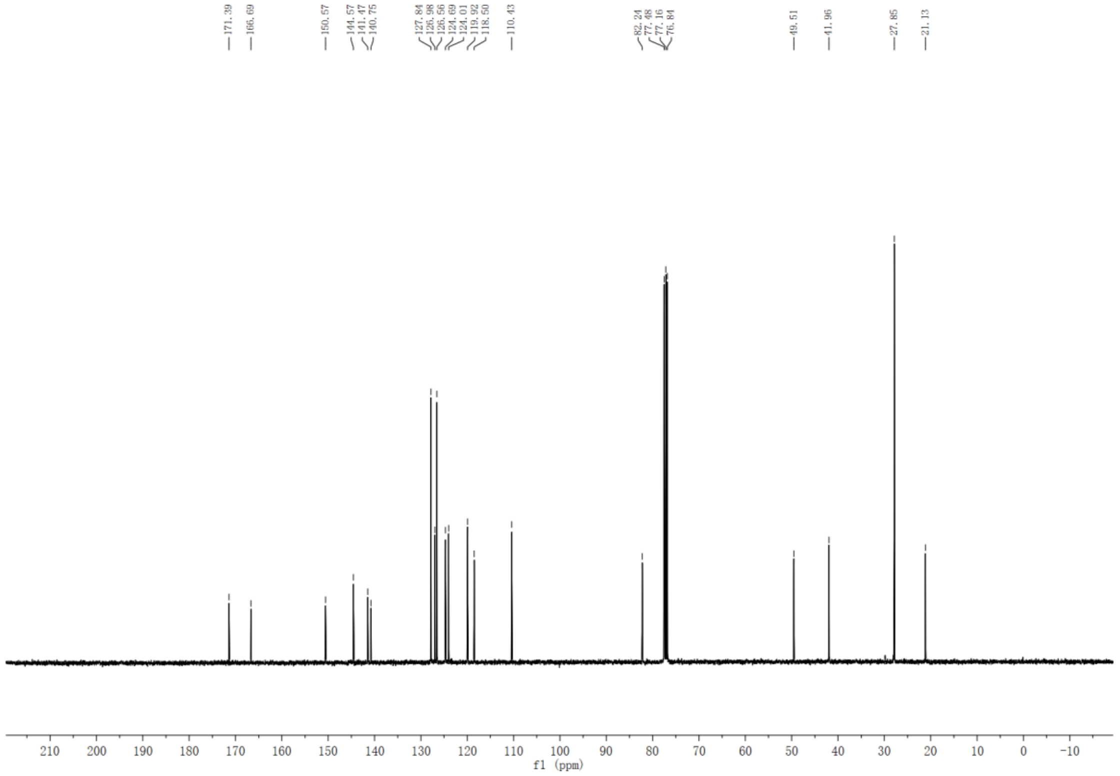

^{1}H NMR spectrum of **8b**

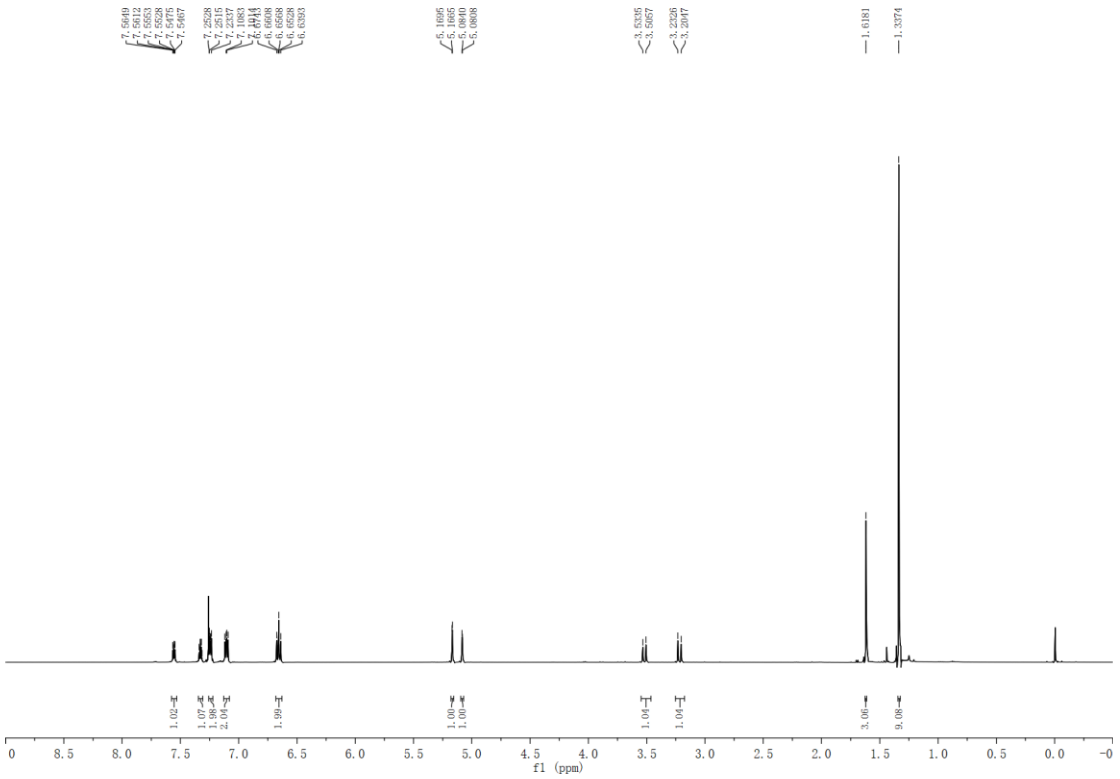

^{13}C NMR spectrum of **8b**

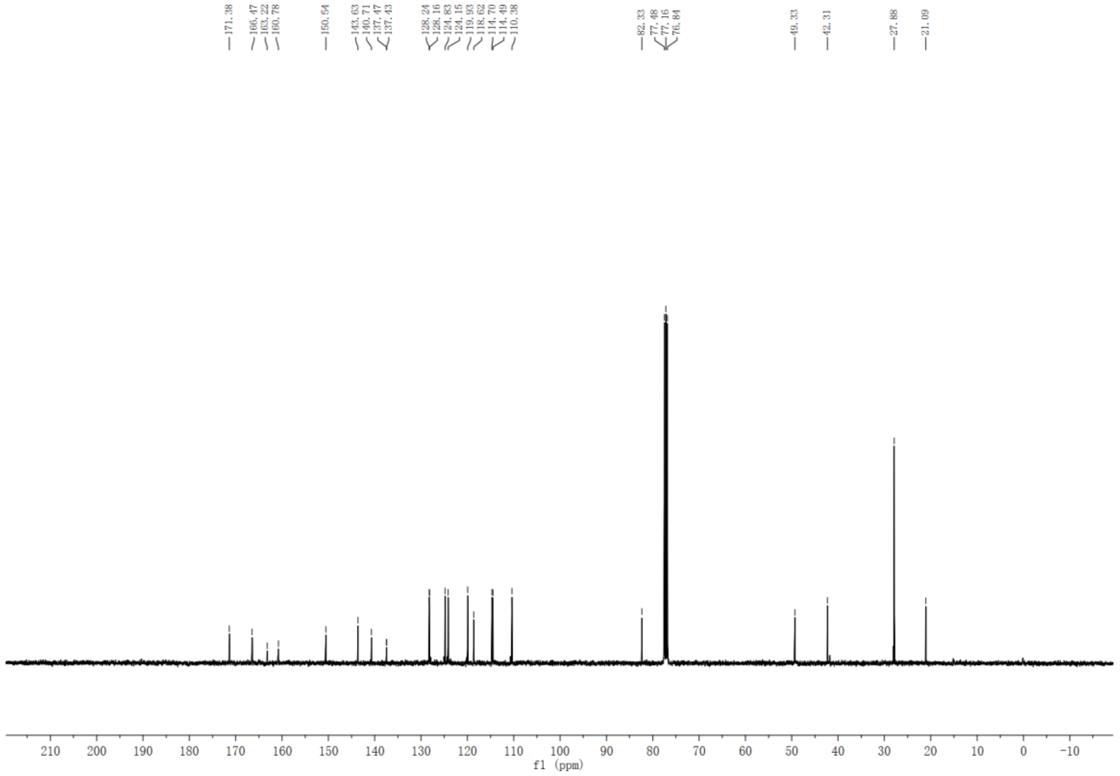

^{19}F NMR spectrum of **8b**

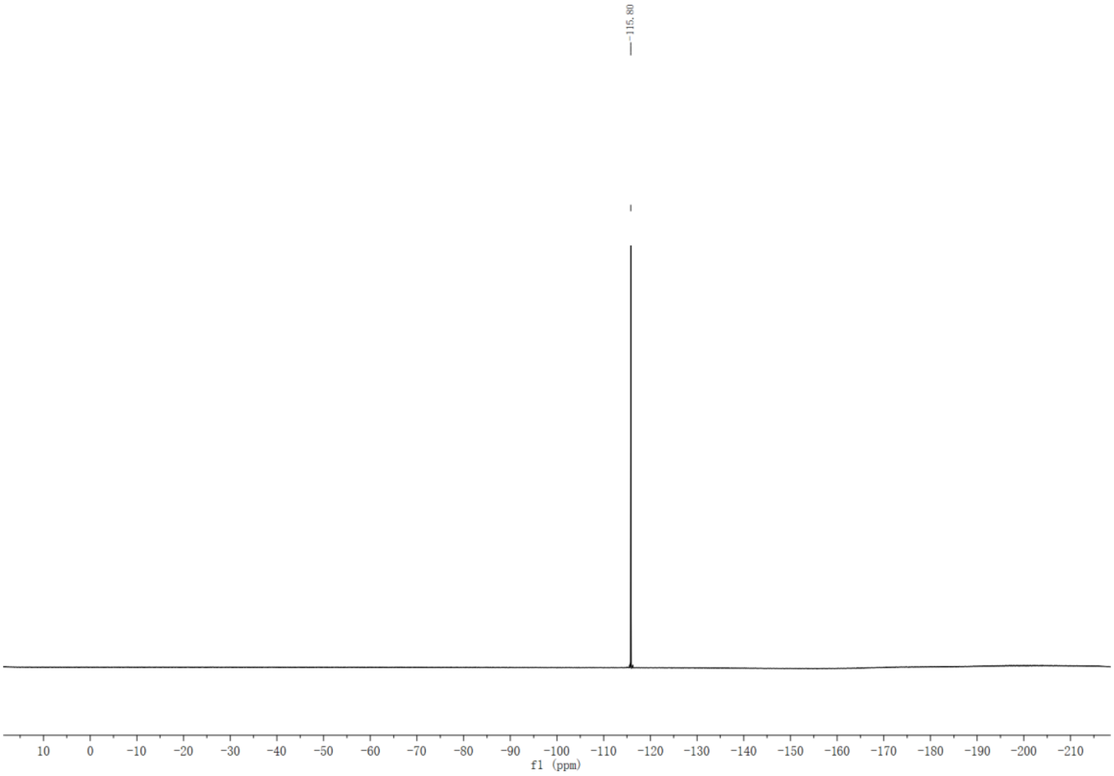

^{1}H NMR spectrum of **8c**

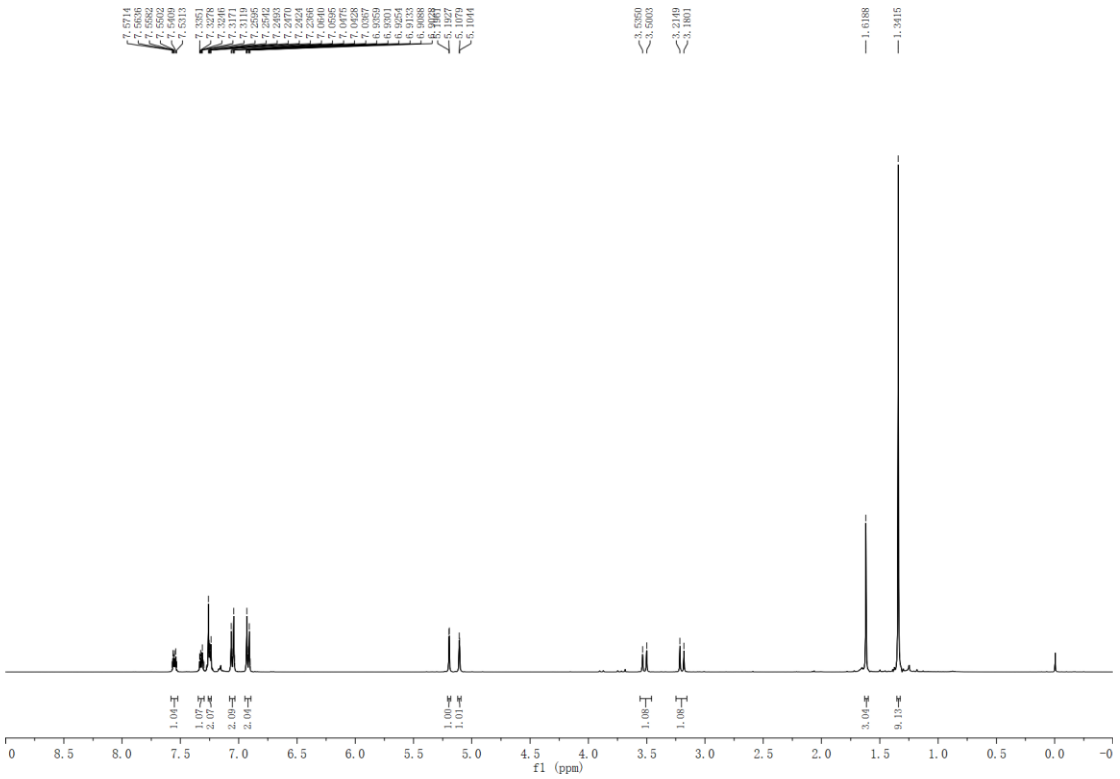

^{13}C NMR spectrum of **8c**

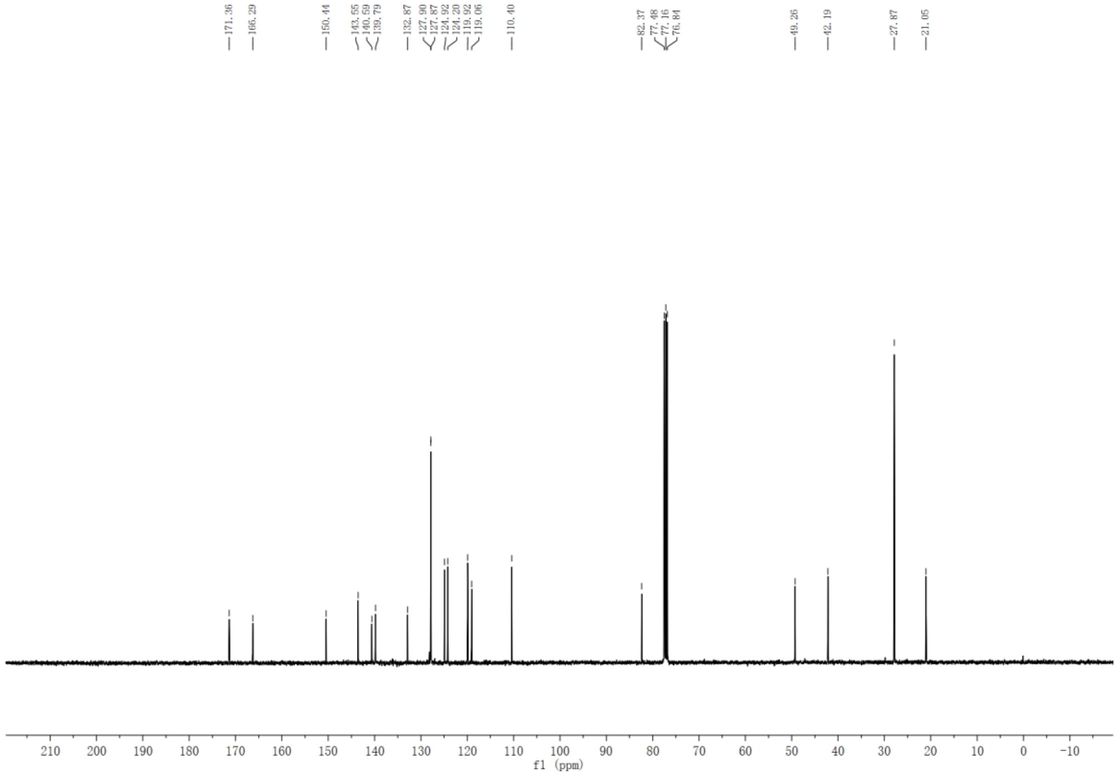

^{1}H NMR spectrum of **8d**

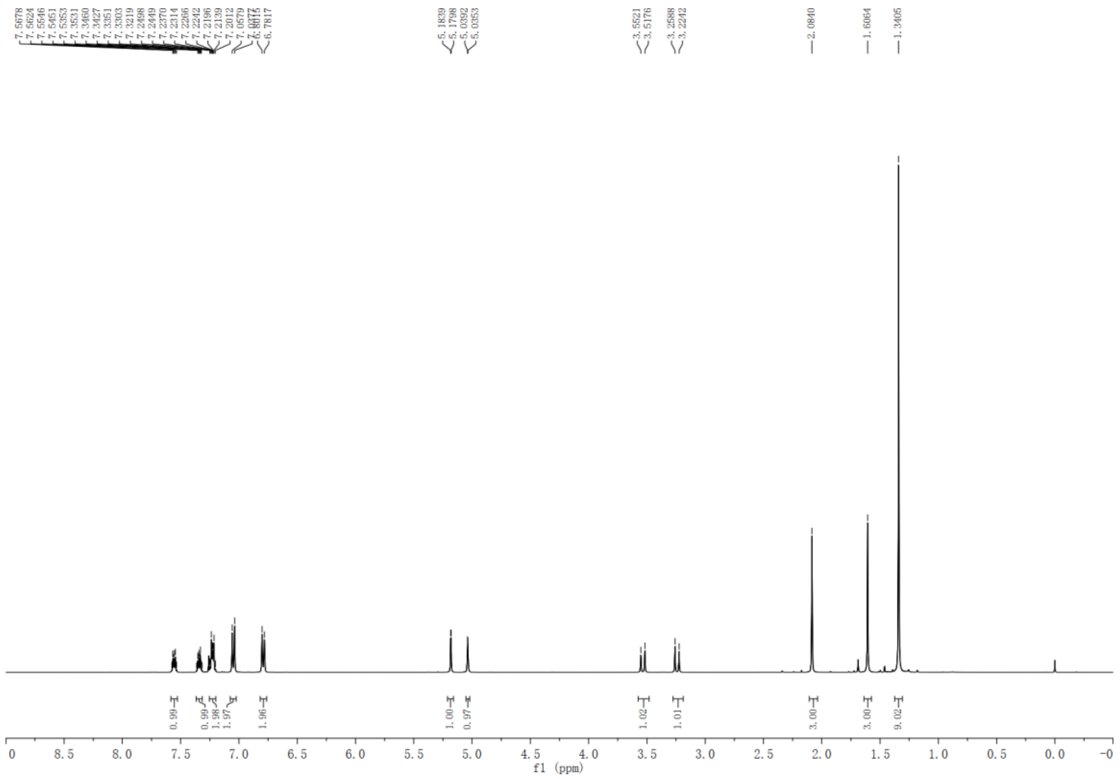

^{13}C NMR spectrum of **8d**

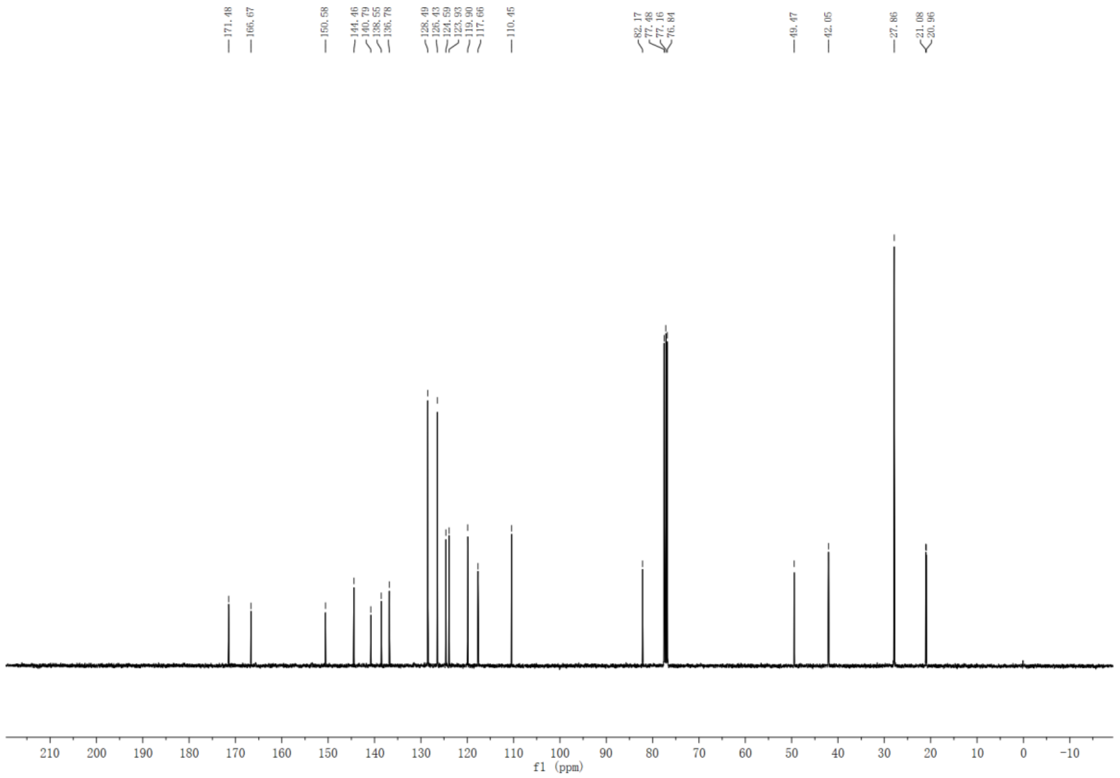

^{1}H NMR spectrum of **8e**

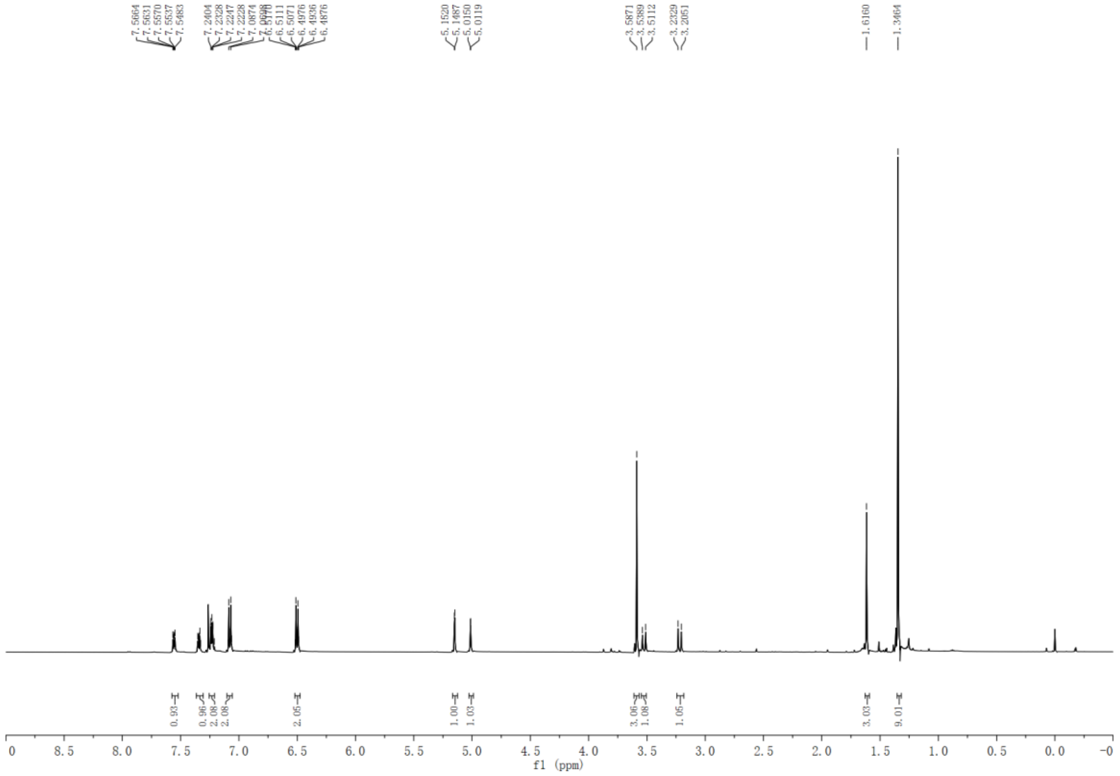

^{13}C NMR spectrum of **8e**

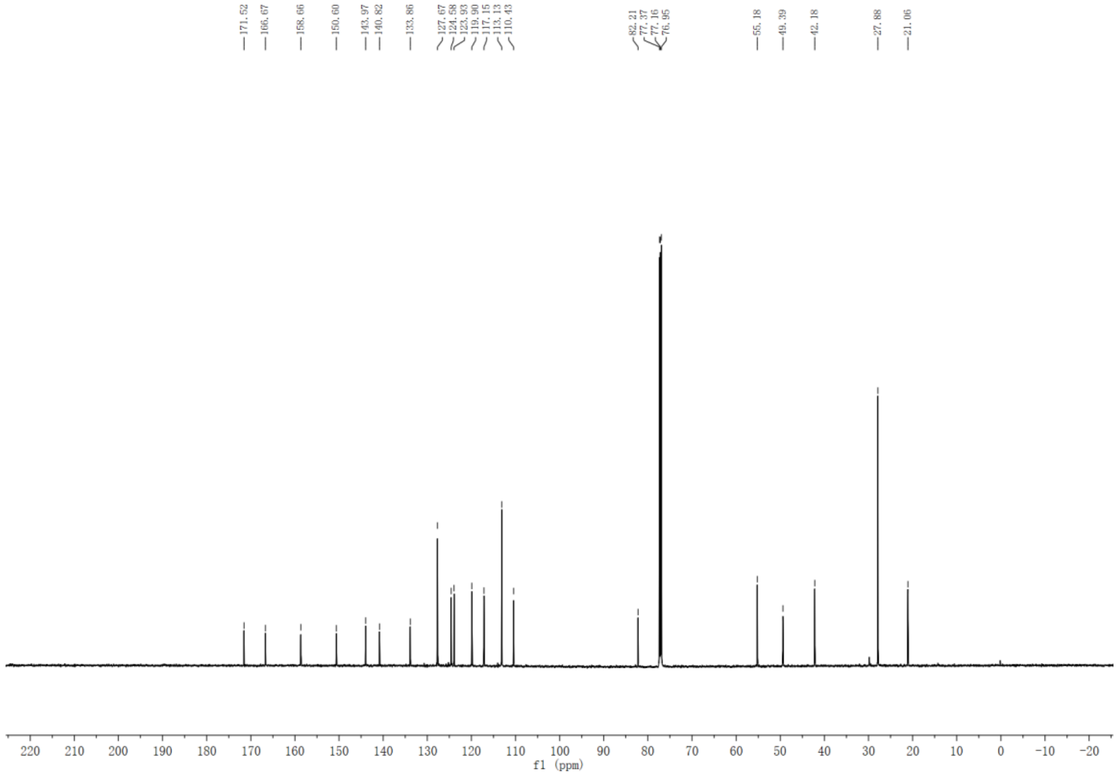

^{1}H NMR spectrum of **8f**

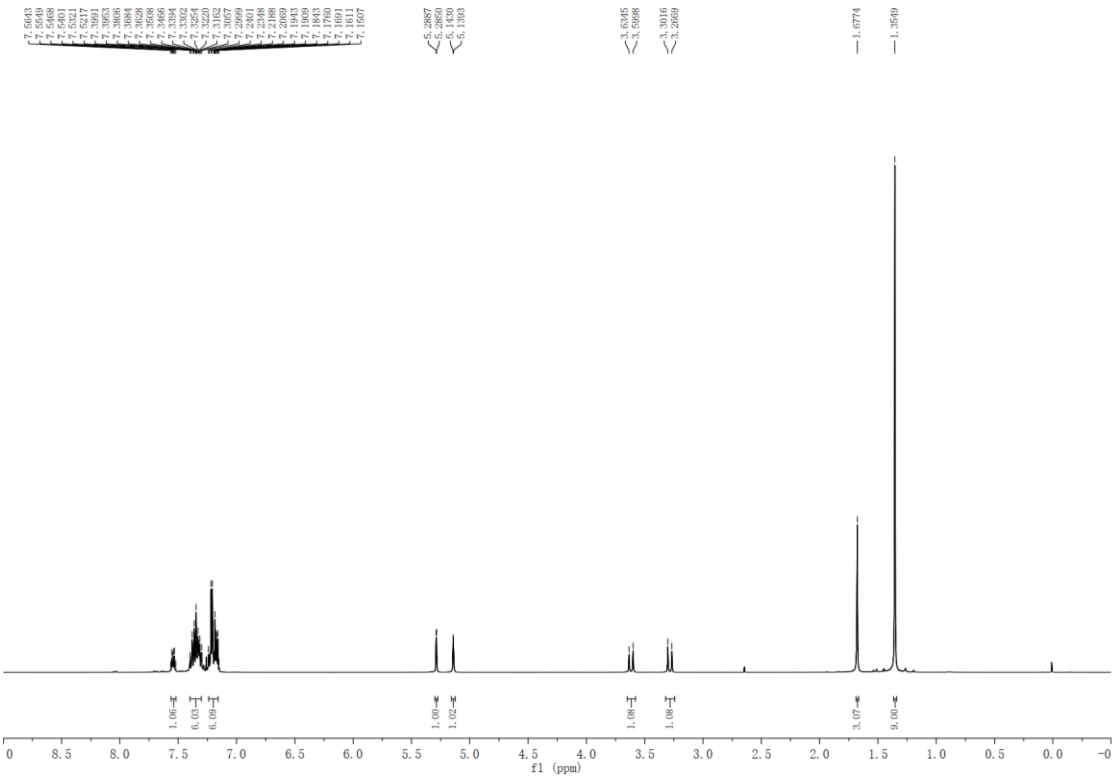

^{13}C NMR spectrum of **8f**

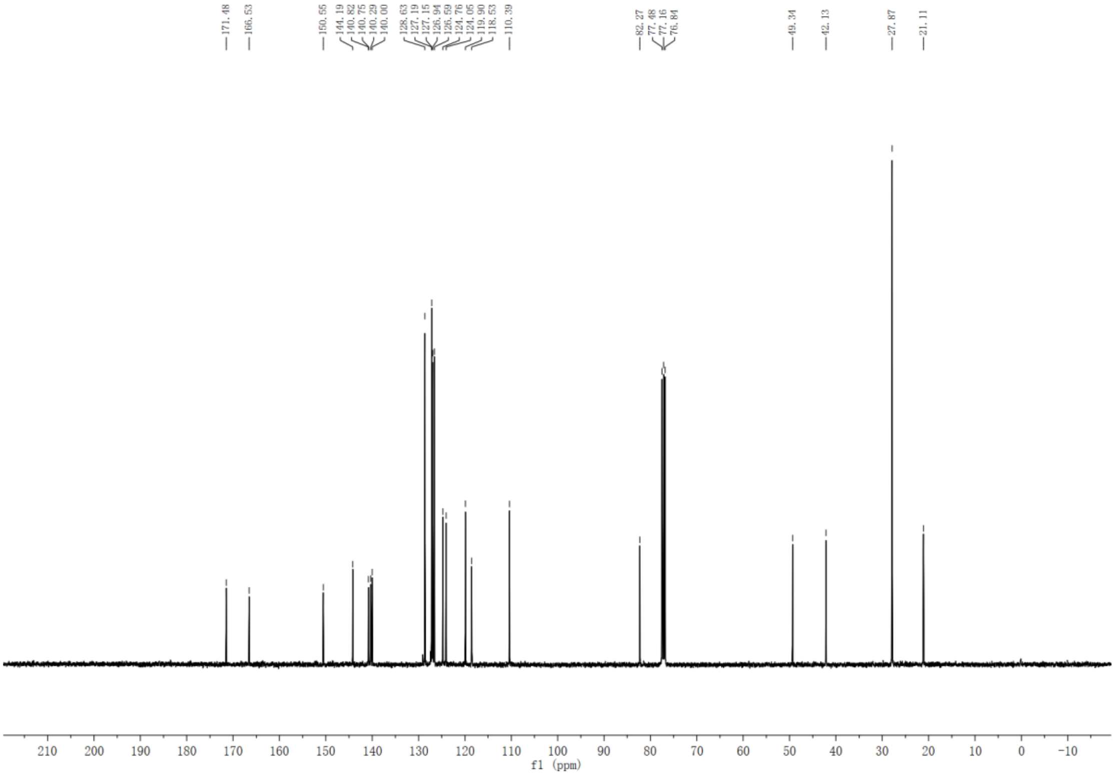

^{1}H NMR spectrum of **8g**

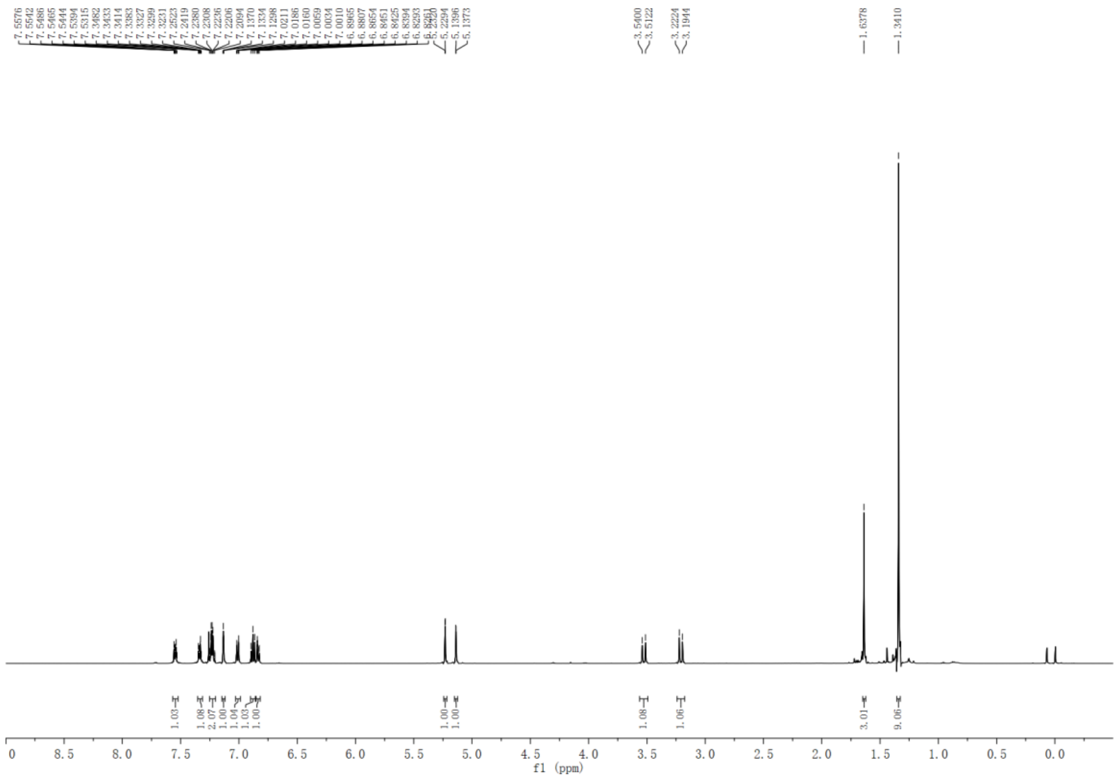

^{13}C NMR spectrum of **8g**

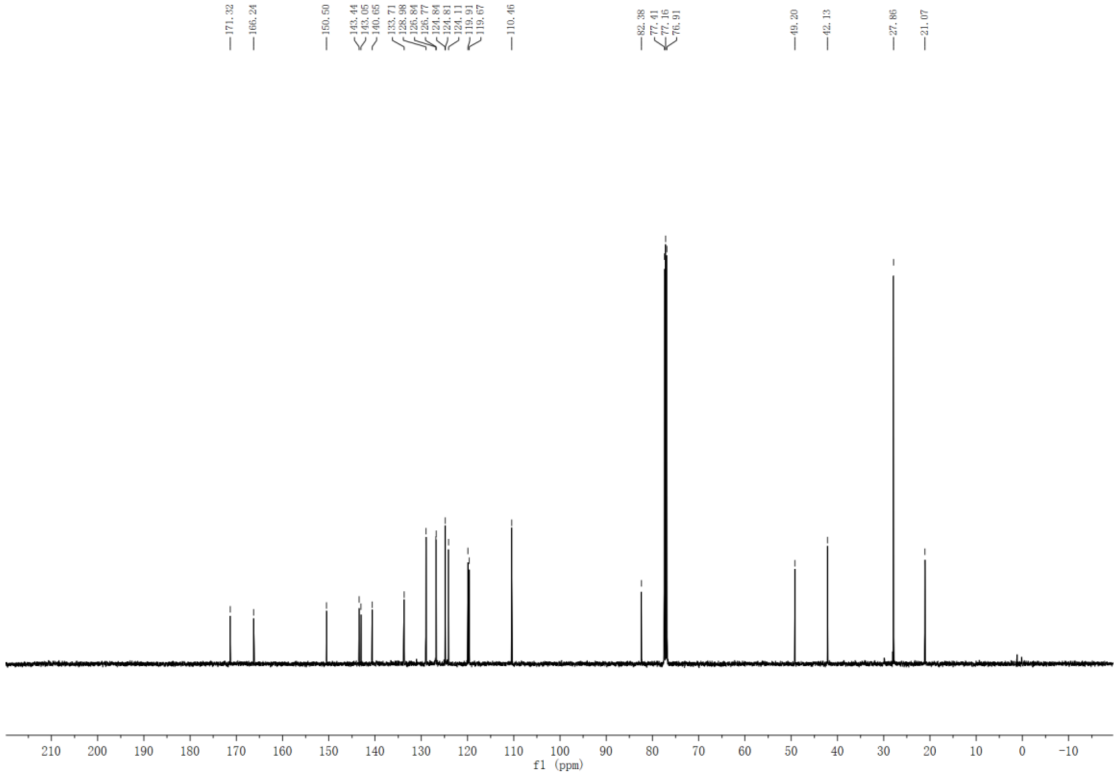

^{1}H NMR spectrum of **8h**

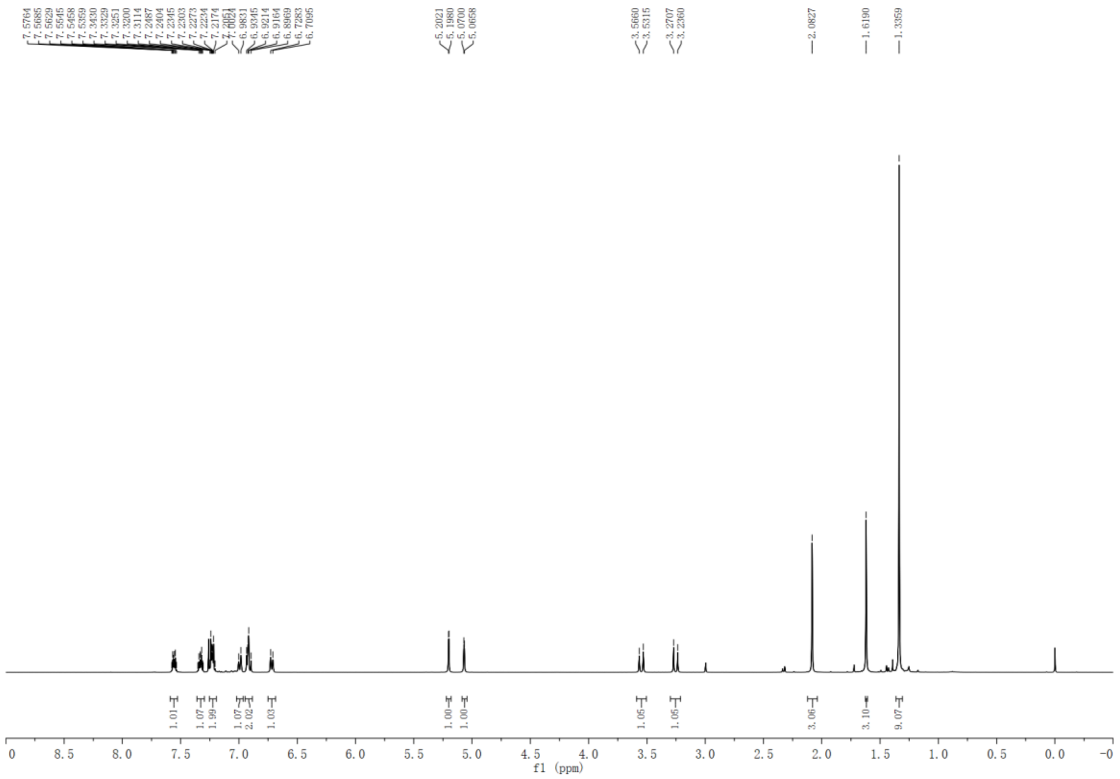

^{13}C NMR spectrum of **8h**

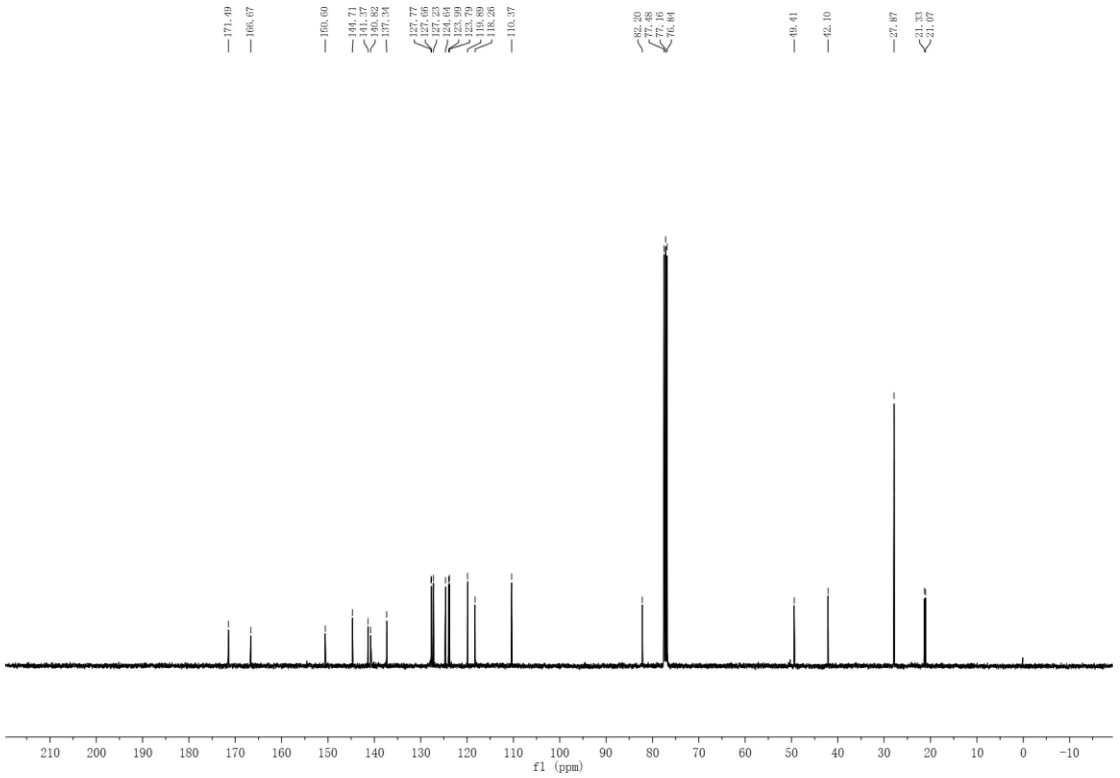

^{1}H NMR spectrum of **8i**

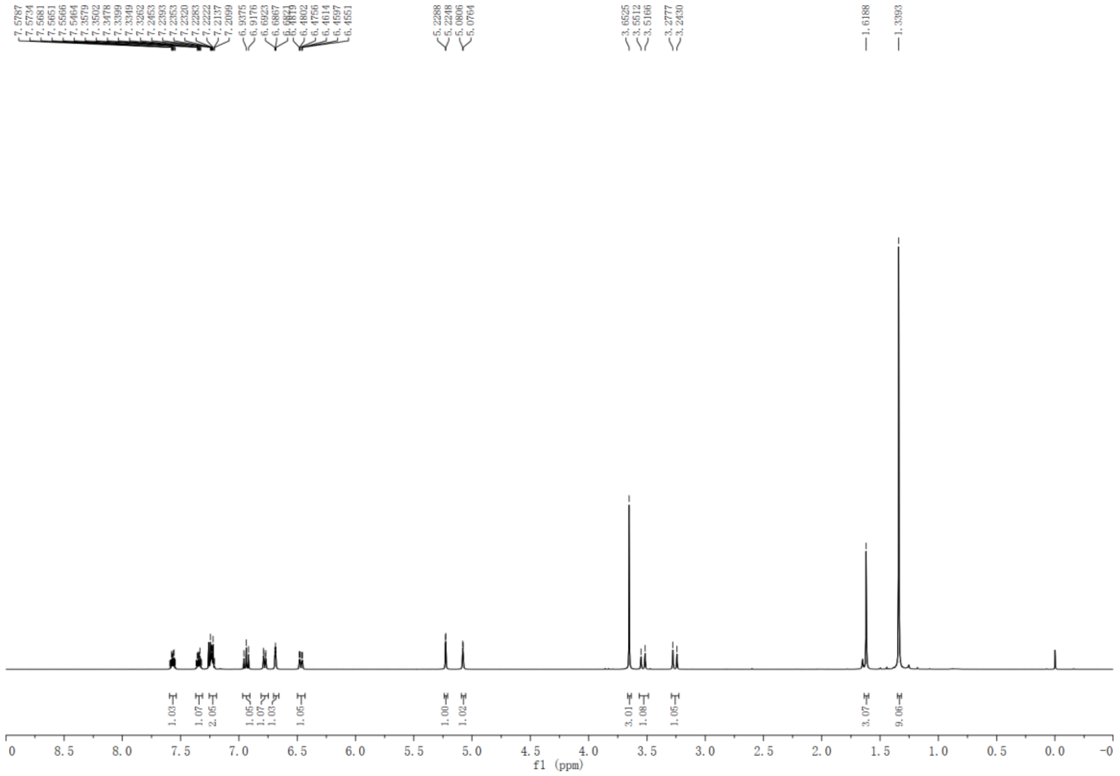

^{13}C NMR spectrum of **8i**

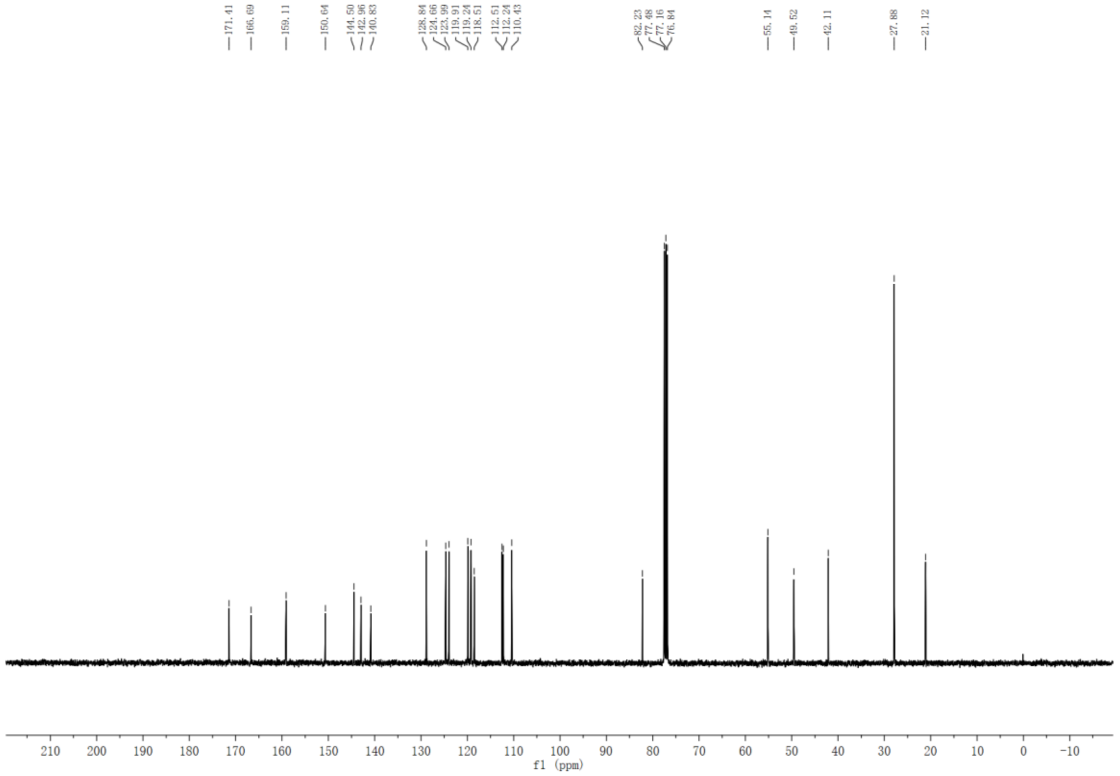

^{1}H NMR spectrum of **8j**

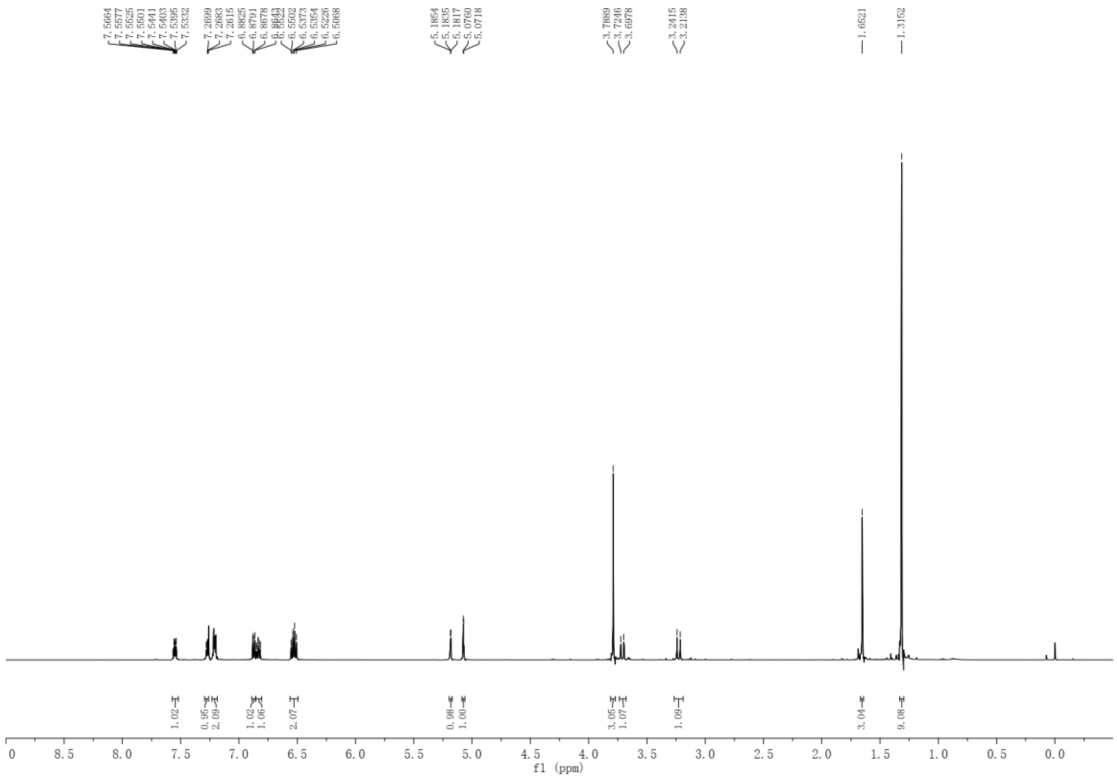

^{13}C NMR spectrum of **8j**

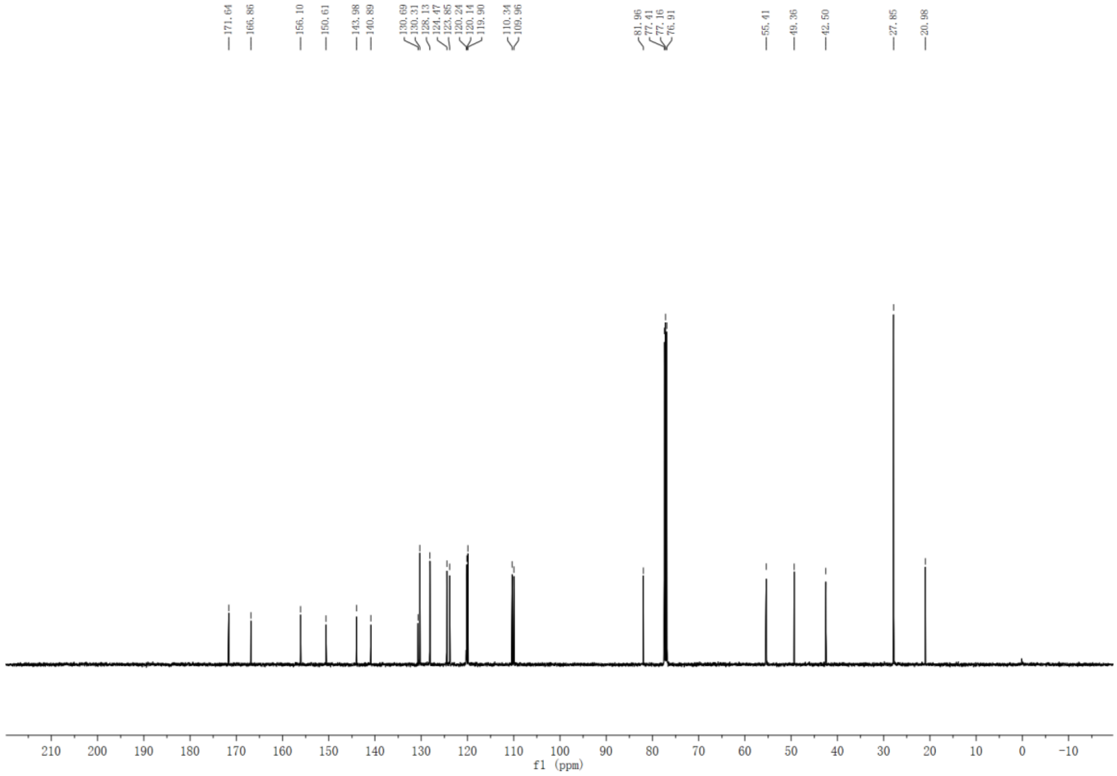

^{1}H NMR spectrum of **8k**

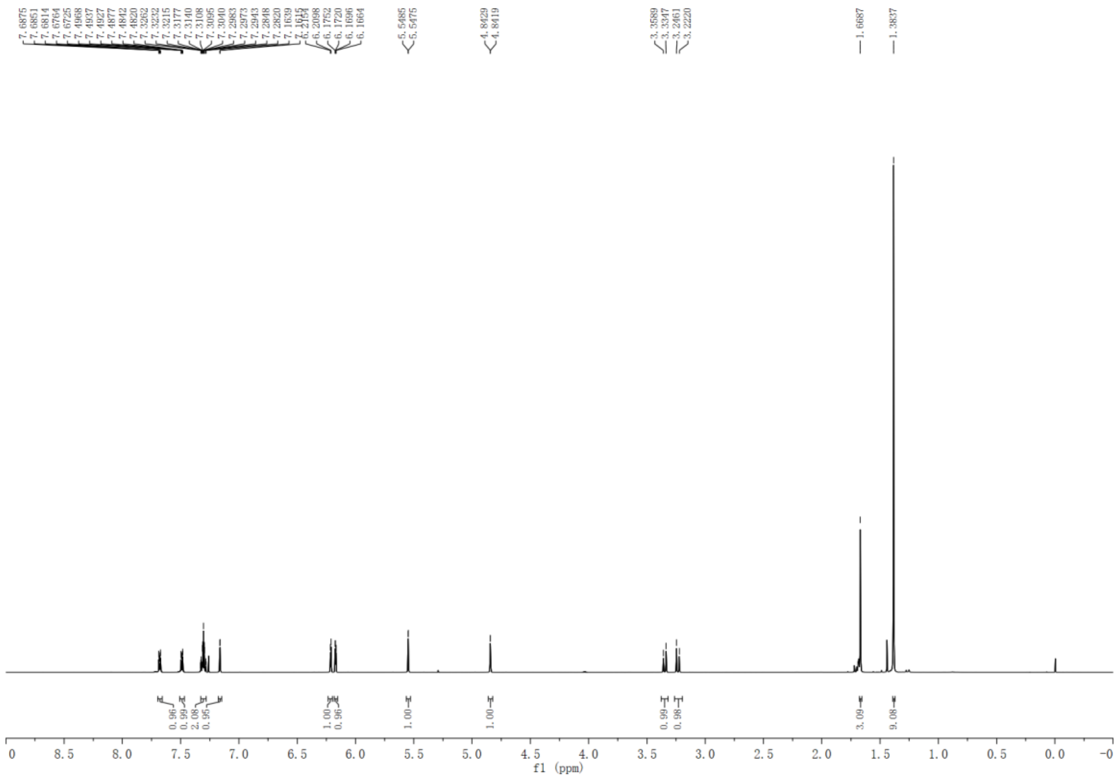

^{13}C NMR spectrum of **8k**

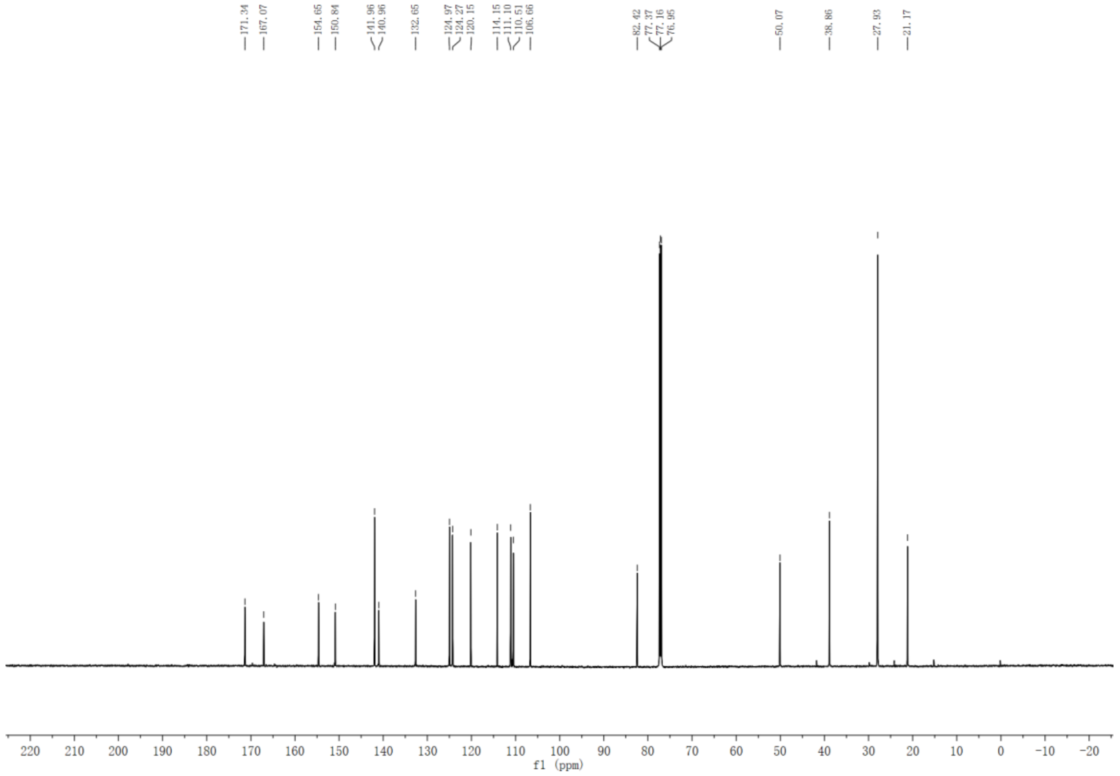

^{1}H NMR spectrum of **8l**

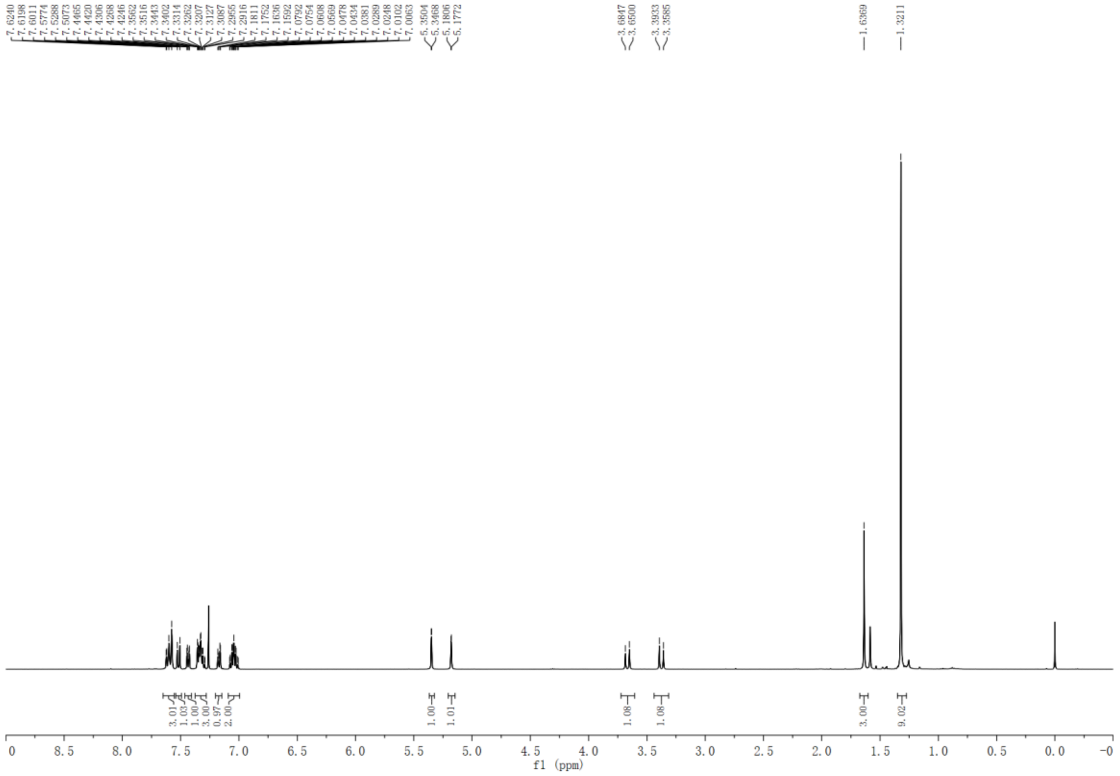

^{13}C NMR spectrum of **8l**

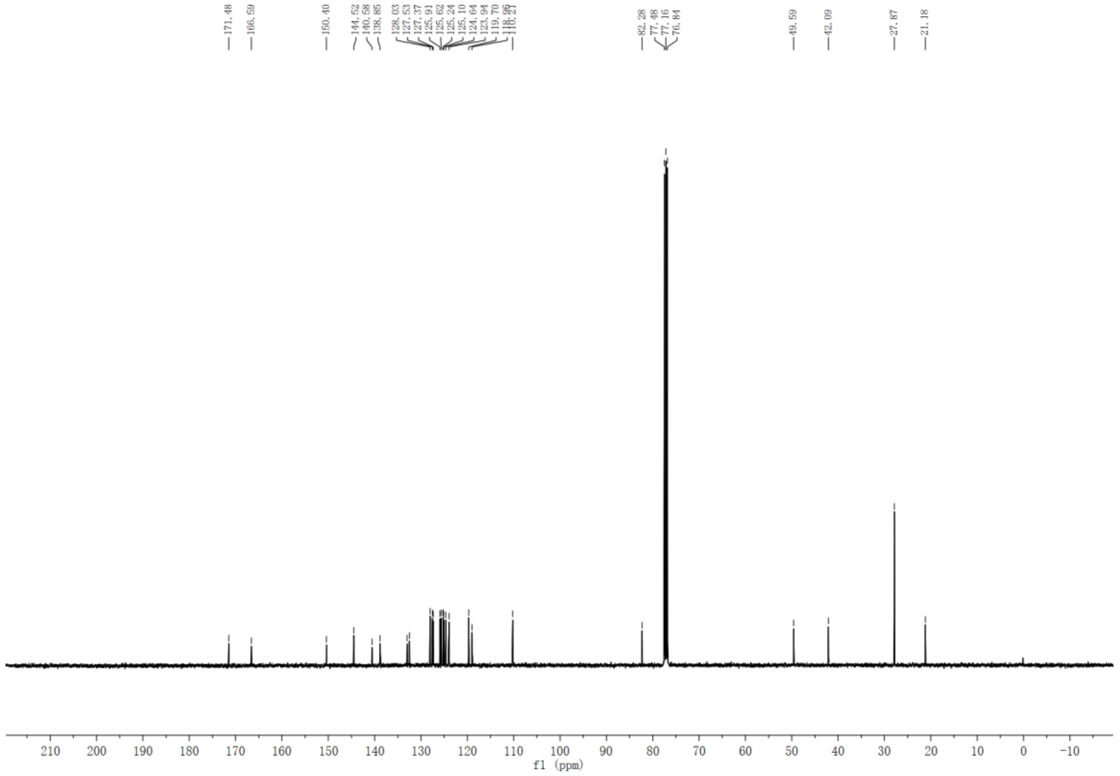

^{1}H NMR spectrum of **8m**

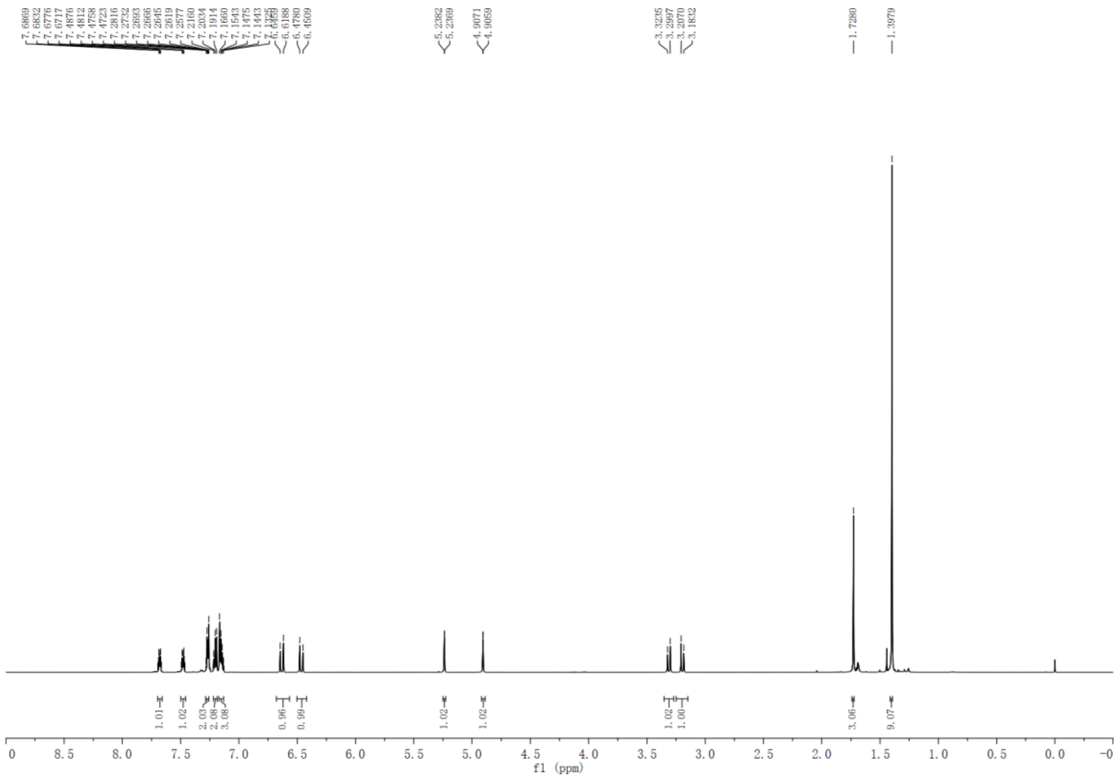

^{13}C NMR spectrum of **8m**

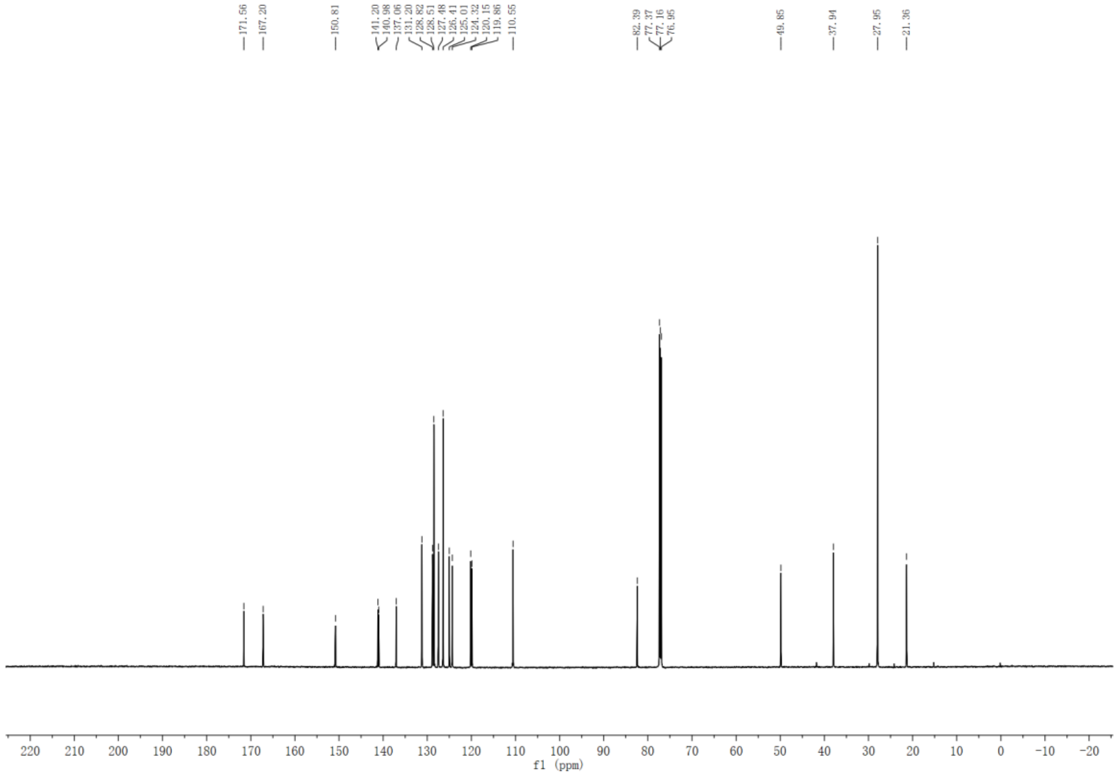

^{1}H NMR spectrum of **8n**

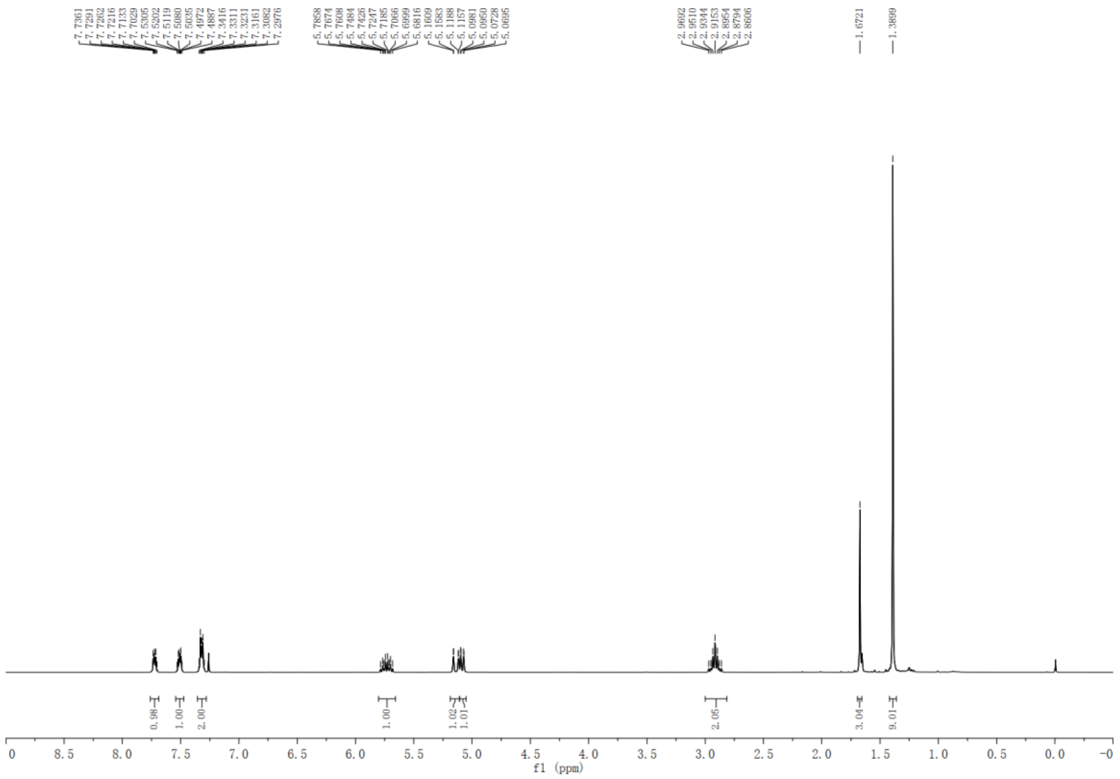

^{13}C NMR spectrum of **8n**

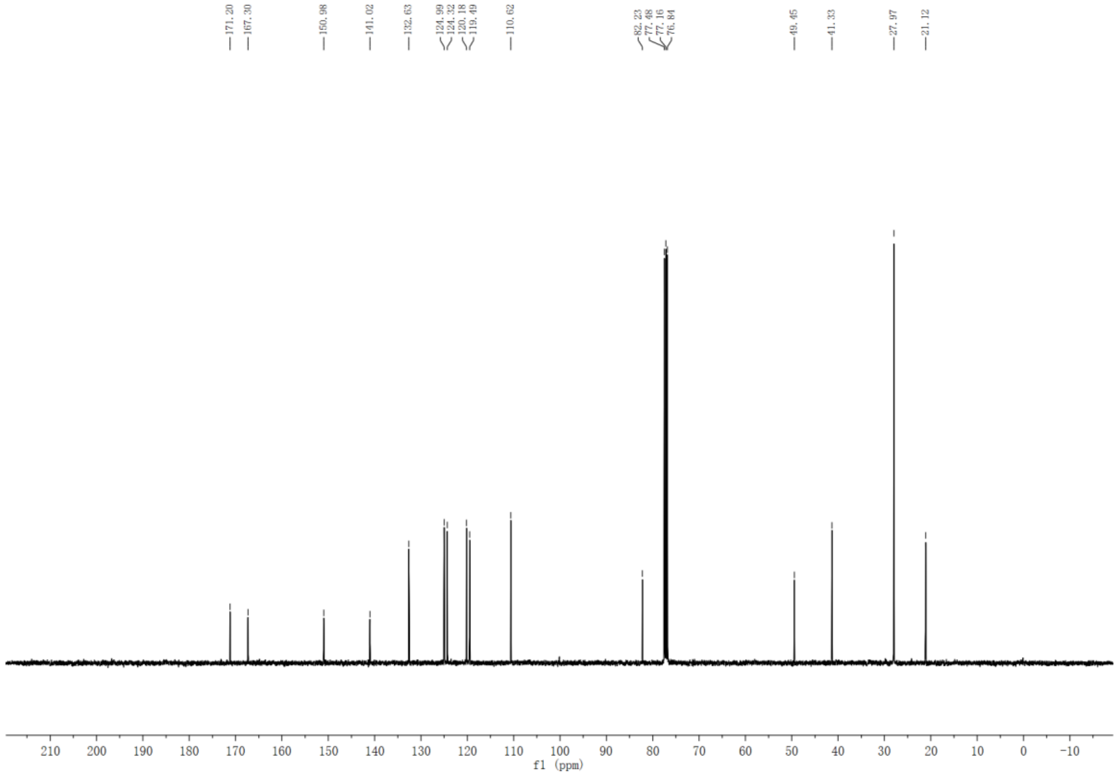

^{1}H NMR spectrum of **8o**

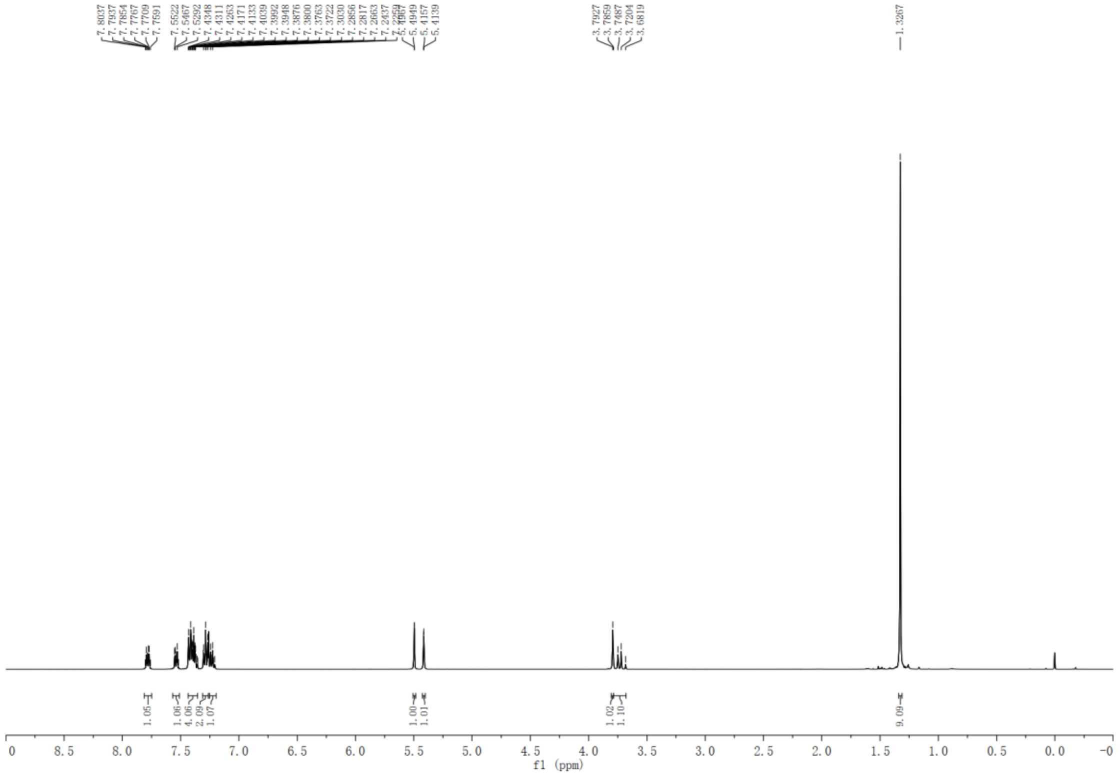

^{13}C NMR spectrum of **8o**

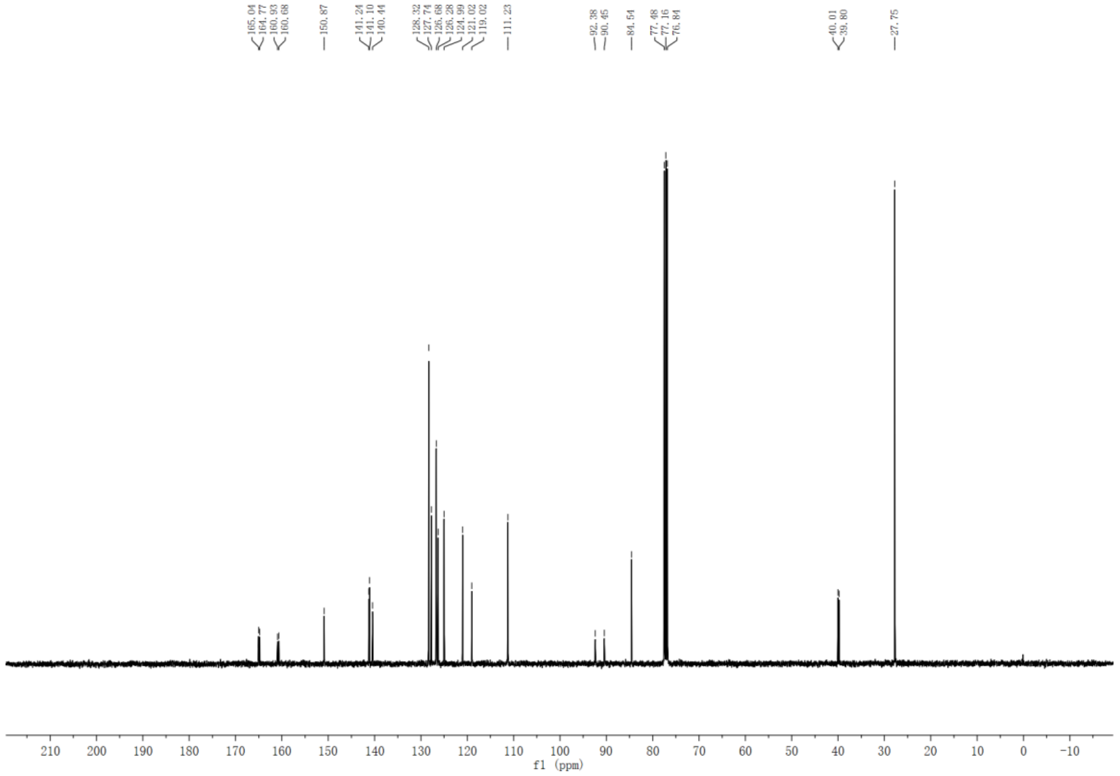

^{19}F NMR spectrum of **8o**

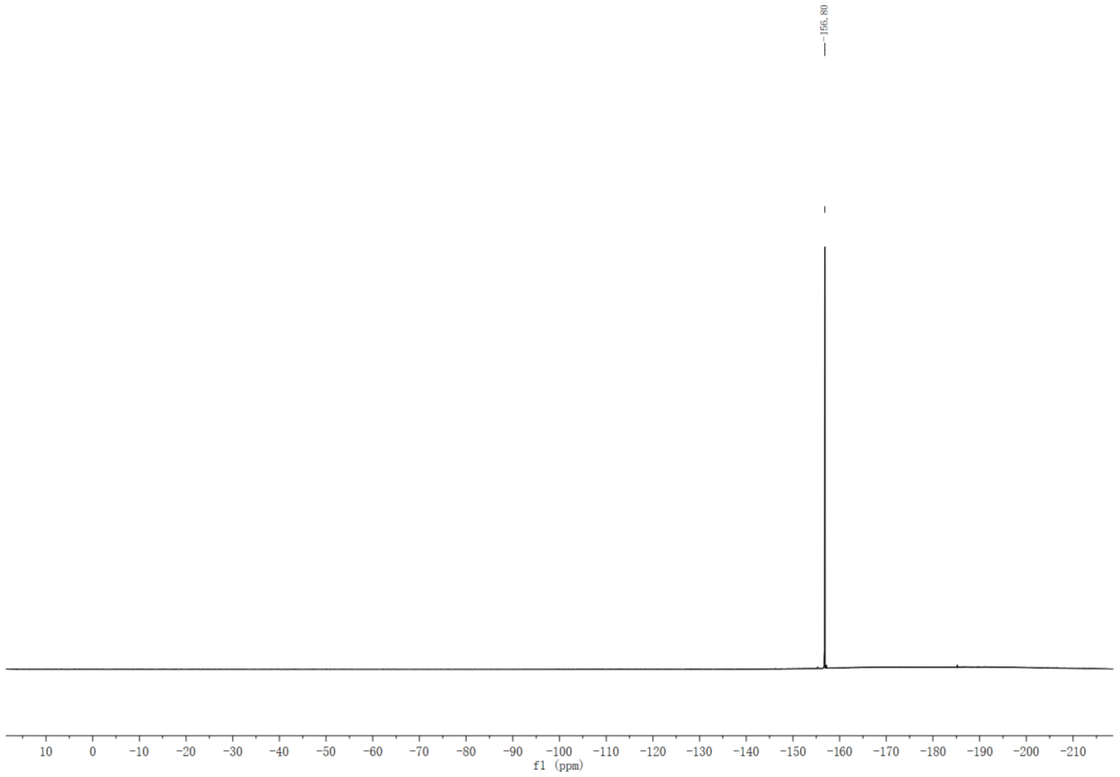

^{1}H NMR spectrum of **8p**

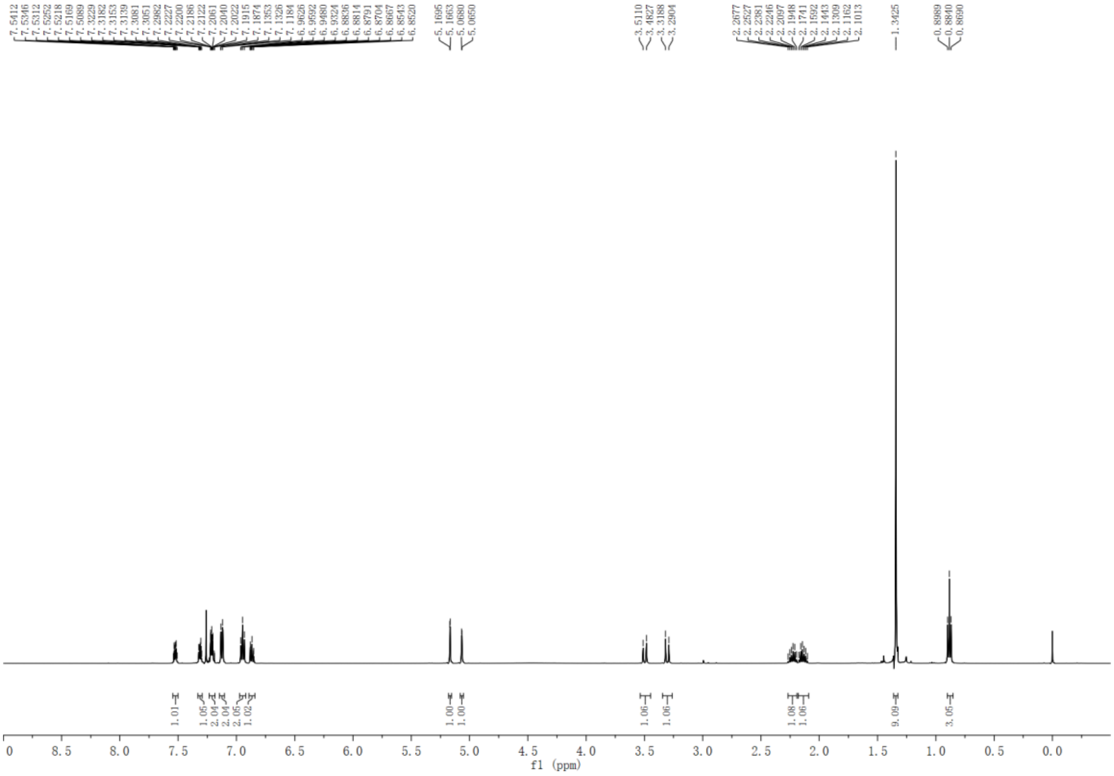

^{13}C NMR spectrum of **8p**

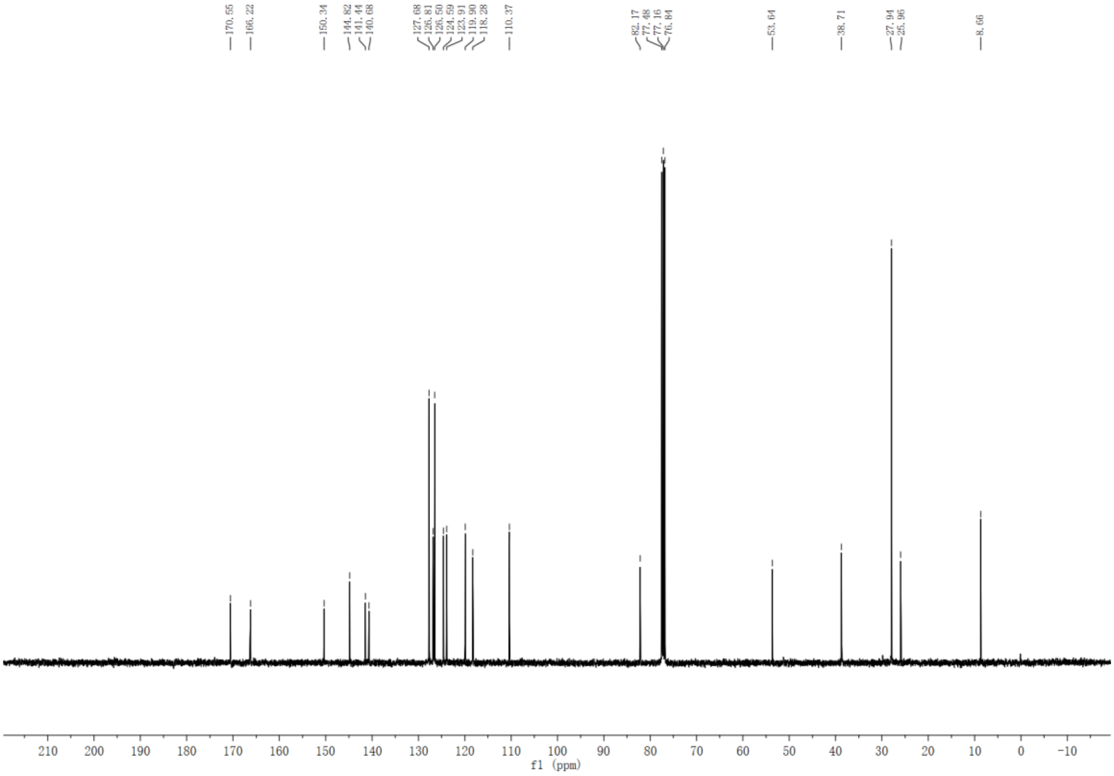

^{1}H NMR spectrum of **8q**

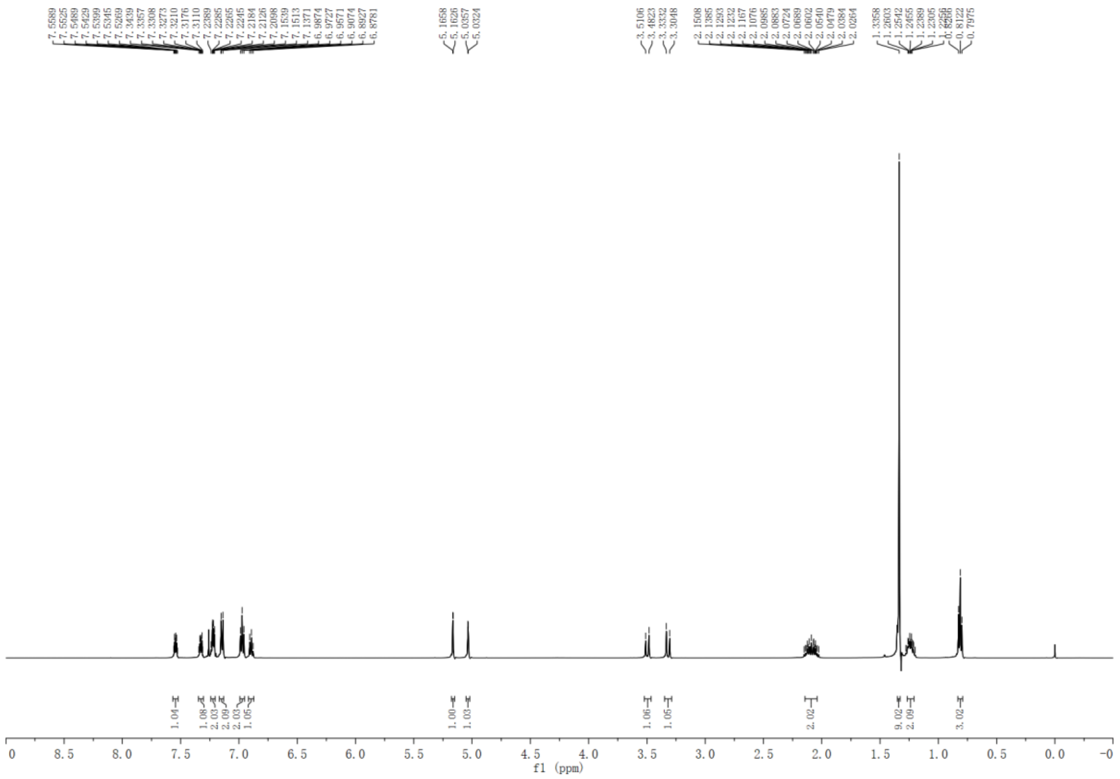

^{13}C NMR spectrum of **8q**

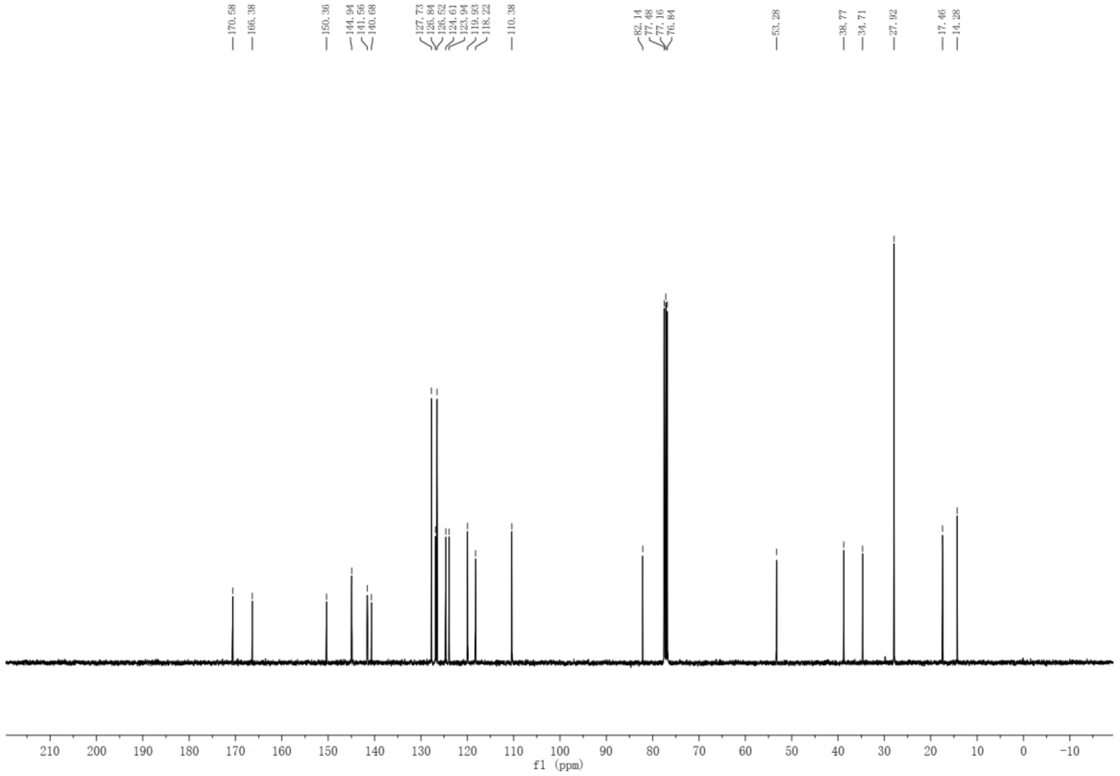

^{1}H NMR spectrum of **8r**

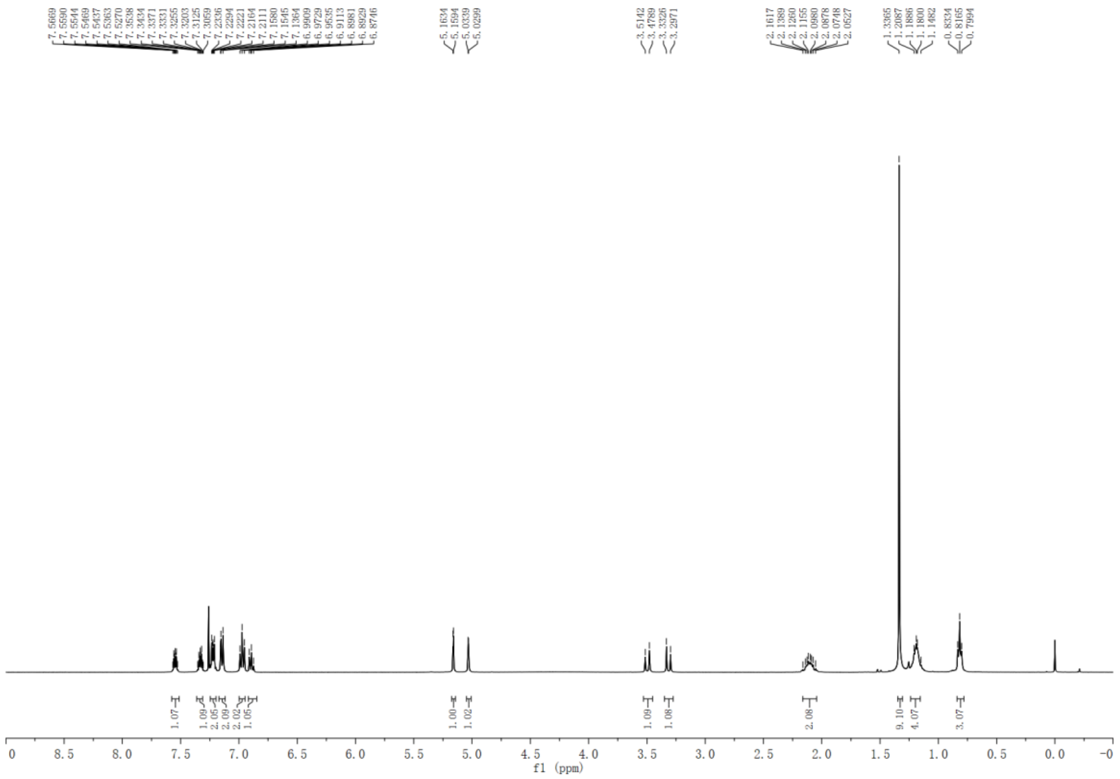

^{13}C NMR spectrum of **8r**

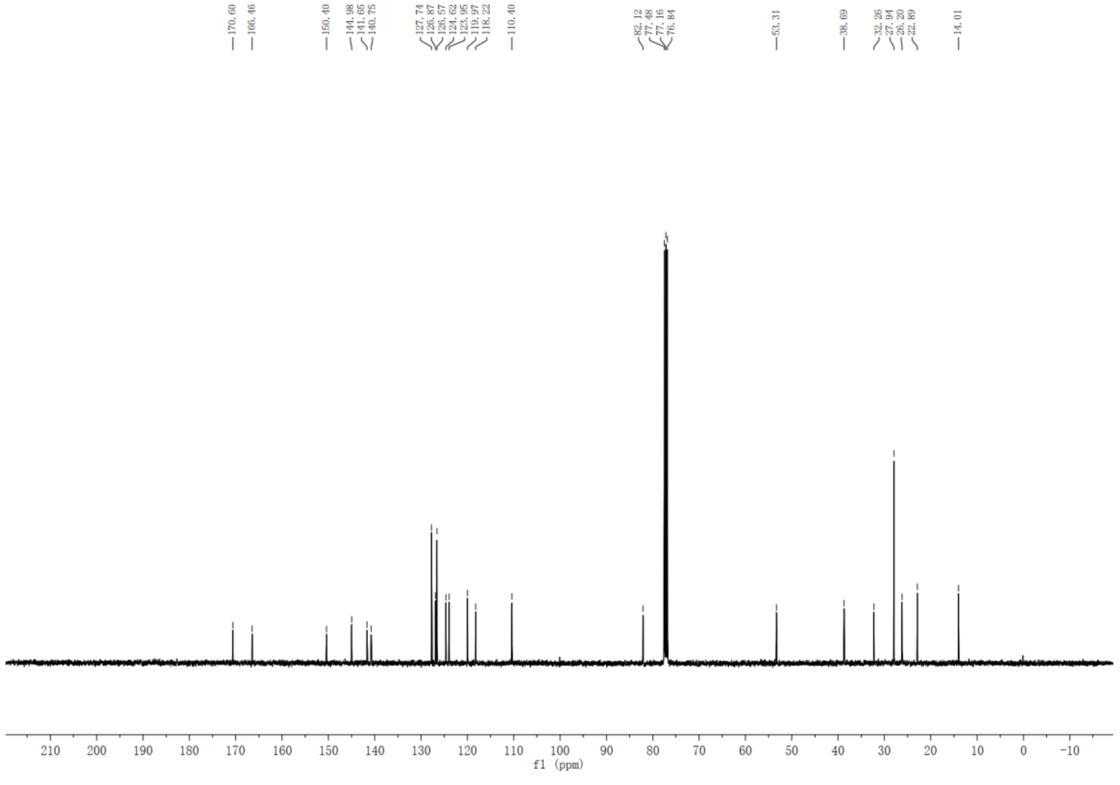

^{1}H NMR spectrum of **8s**

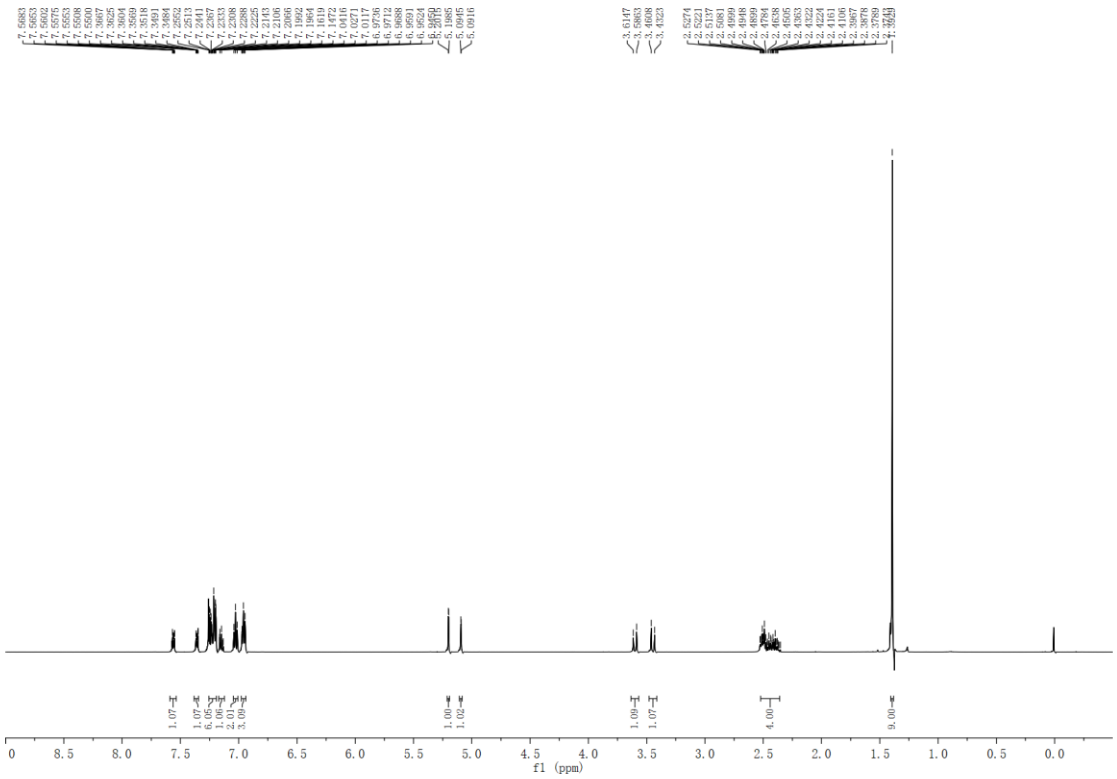

^{13}C NMR spectrum of **8s**

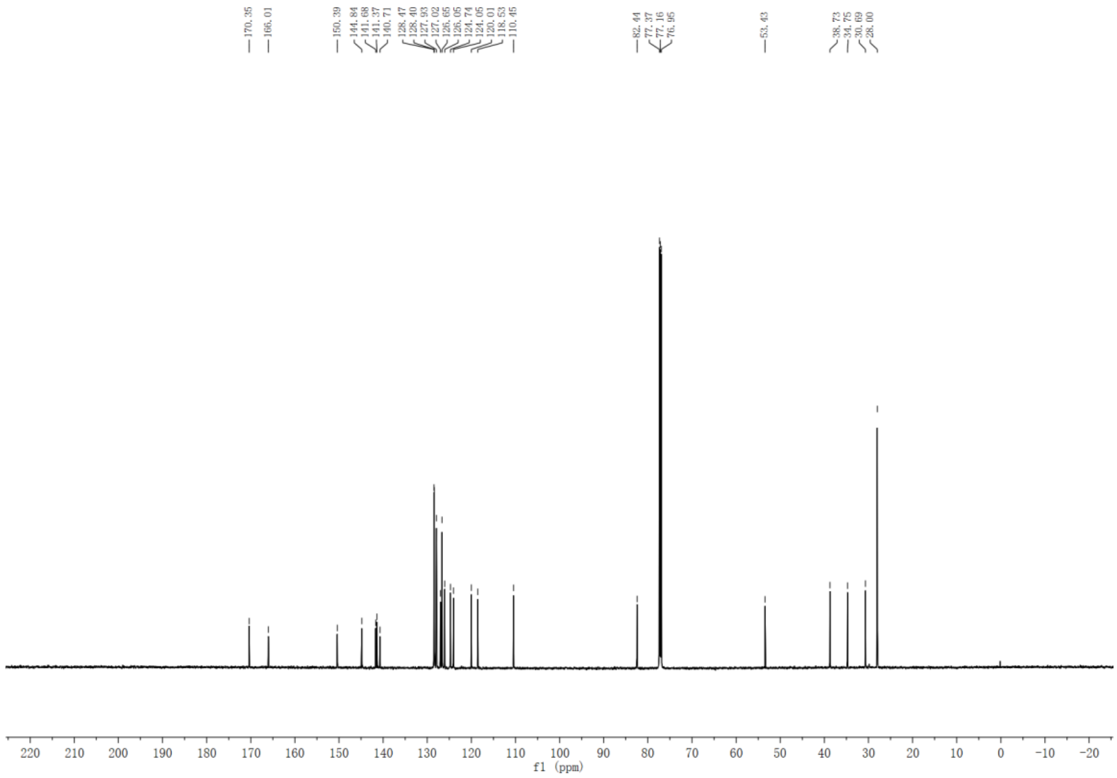

^{1}H NMR spectrum of **8t**

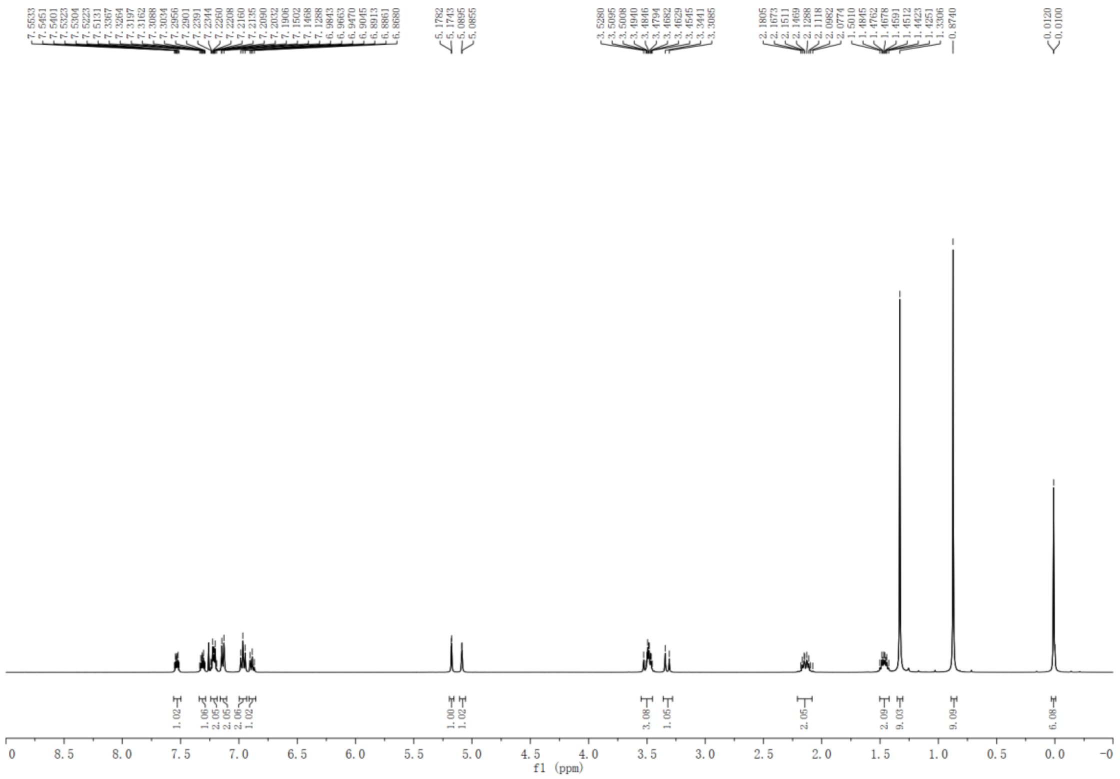

^{13}C NMR spectrum of **8t**

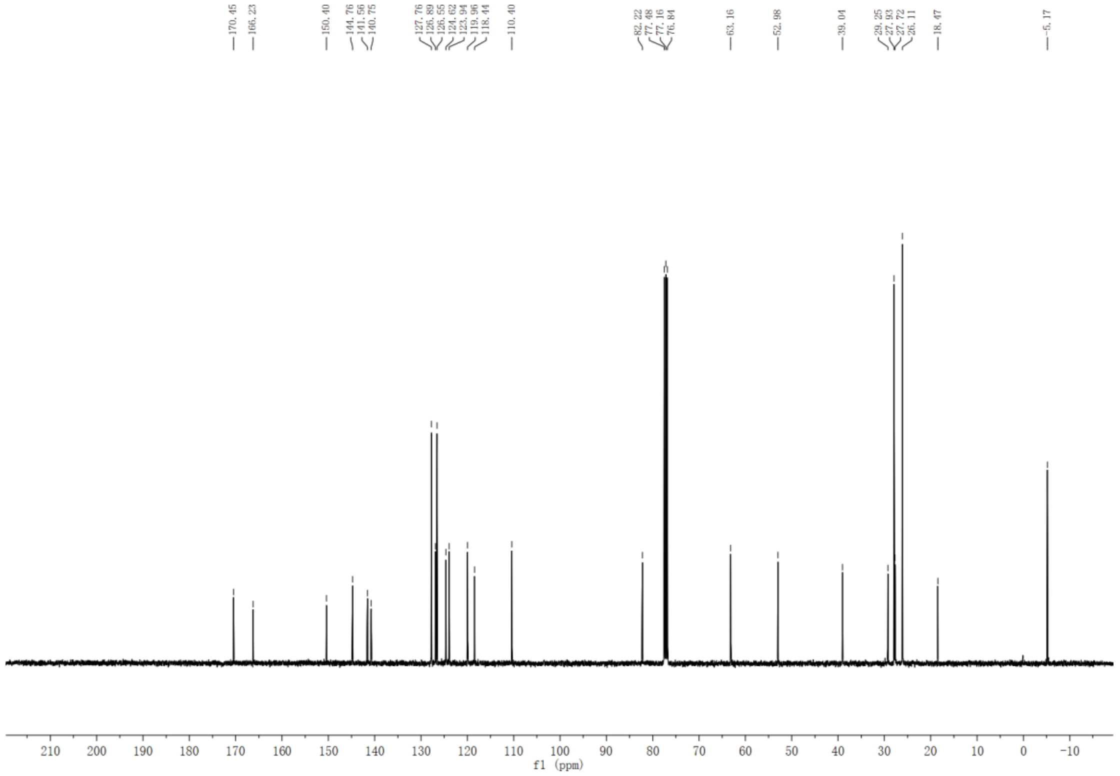

^{1}H NMR spectrum of **8u**

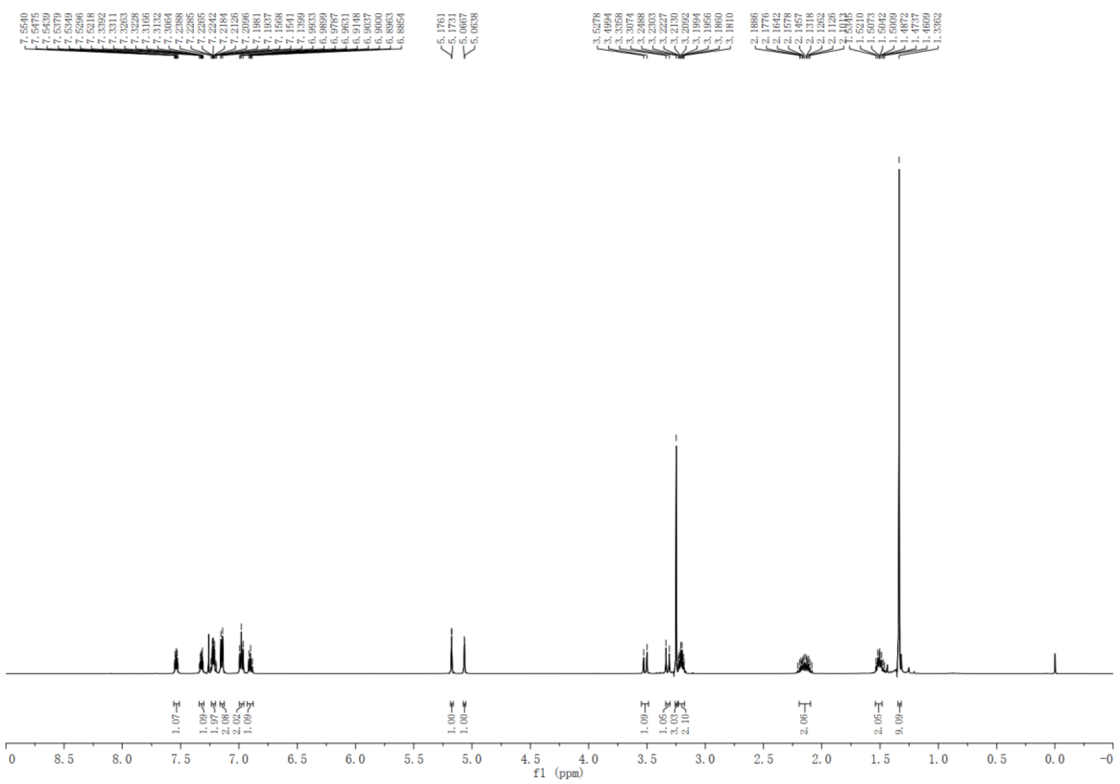

^{13}C NMR spectrum of **8u**

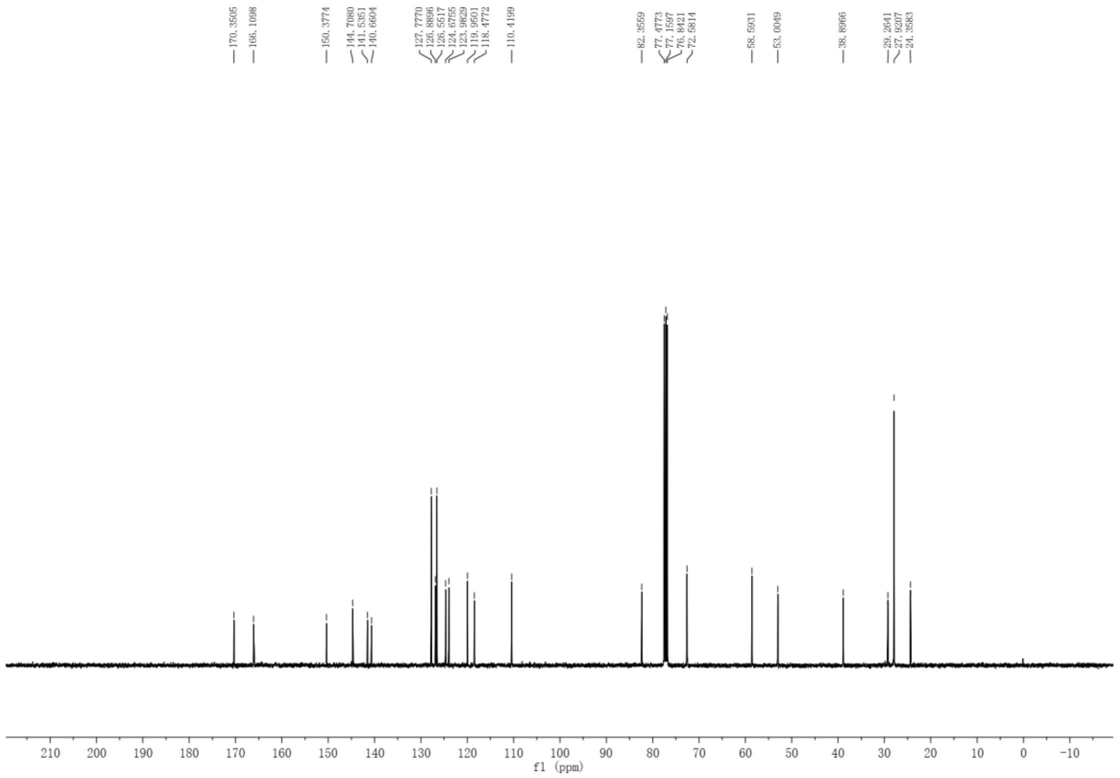

^{1}H NMR spectrum of **8v**

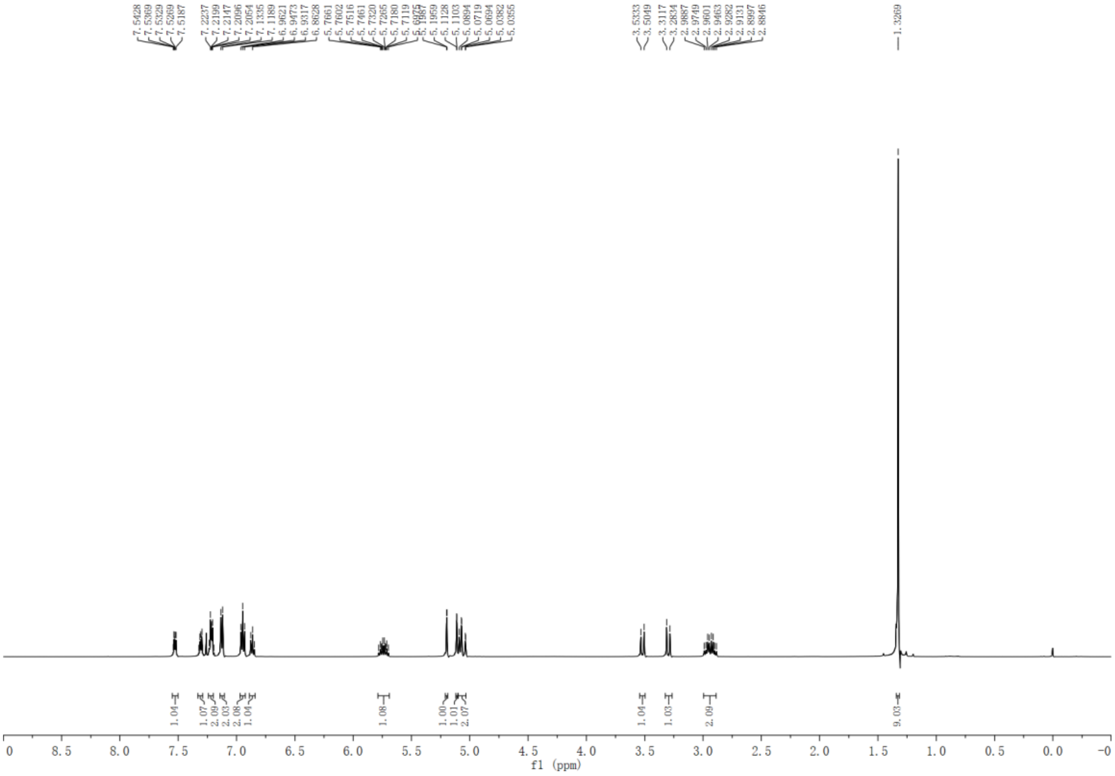

^{13}C NMR spectrum of **8v**

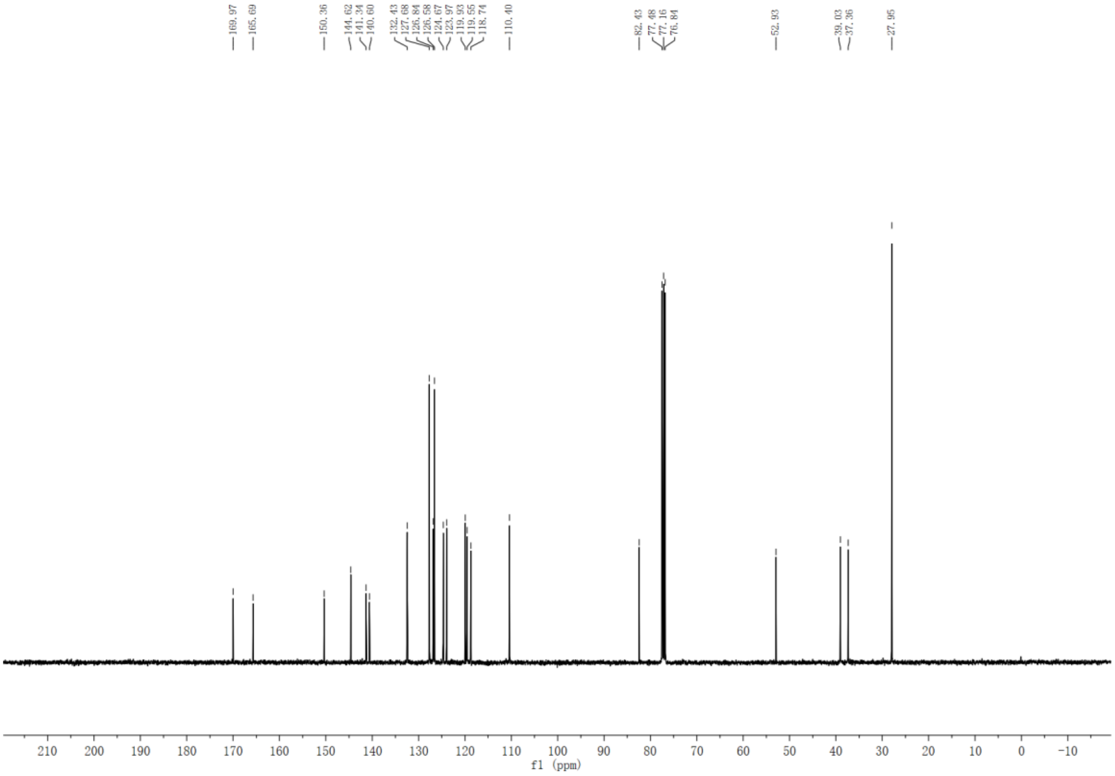

^{1}H NMR spectrum of **8w**

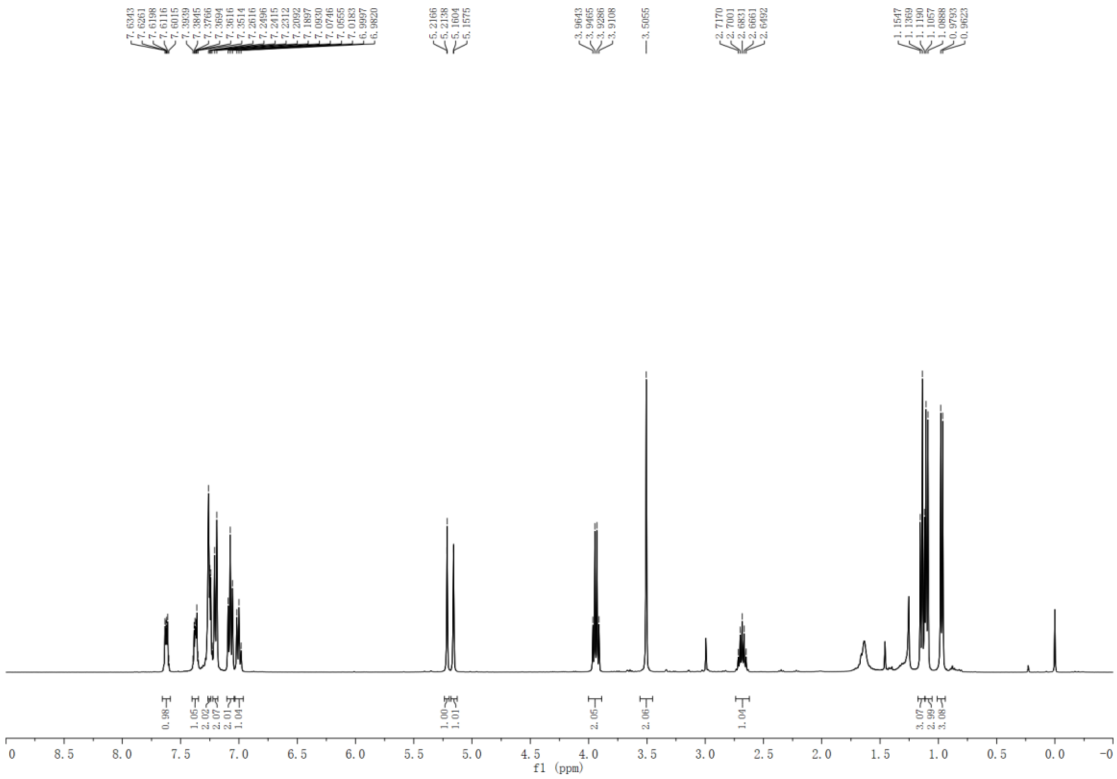

^{13}C NMR spectrum of **8w**

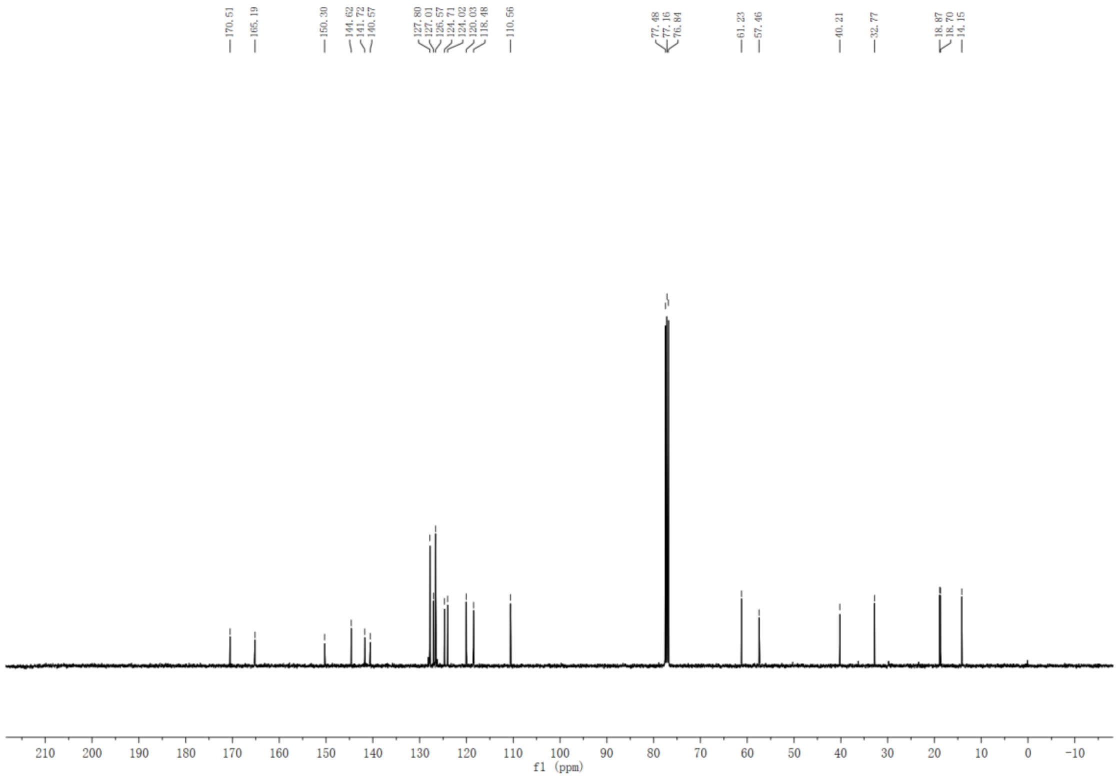

^{1}H NMR spectrum of **8x**

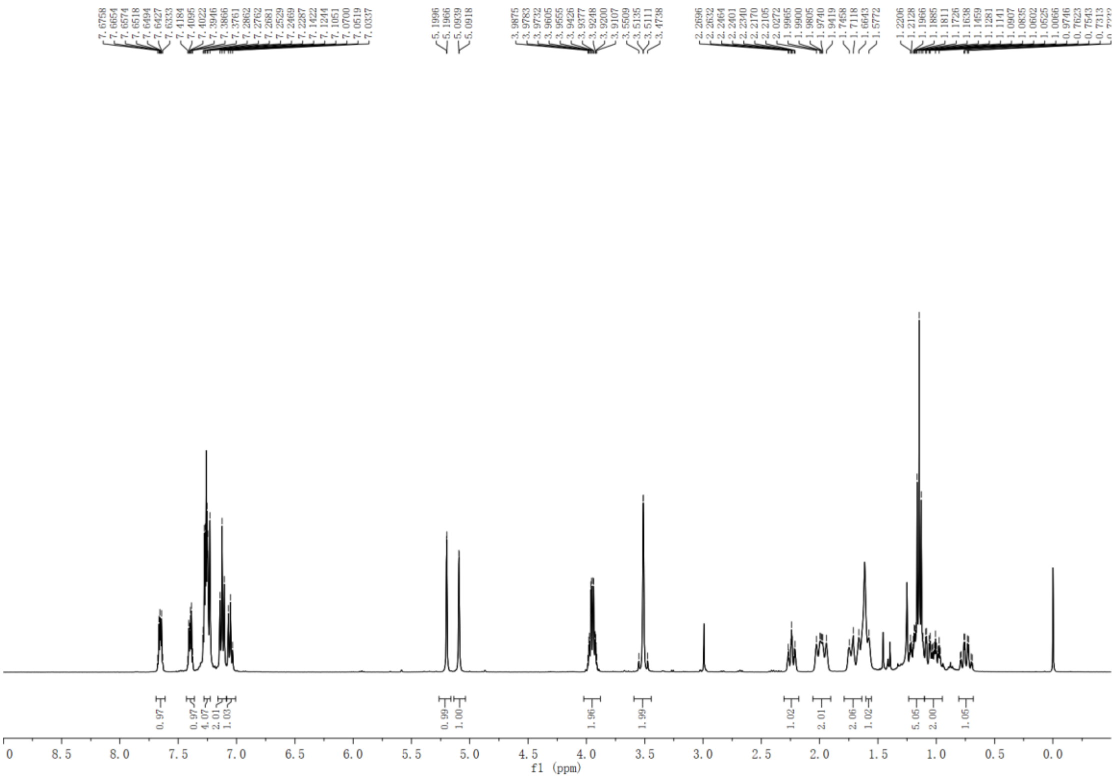

^{13}C NMR spectrum of **8x**

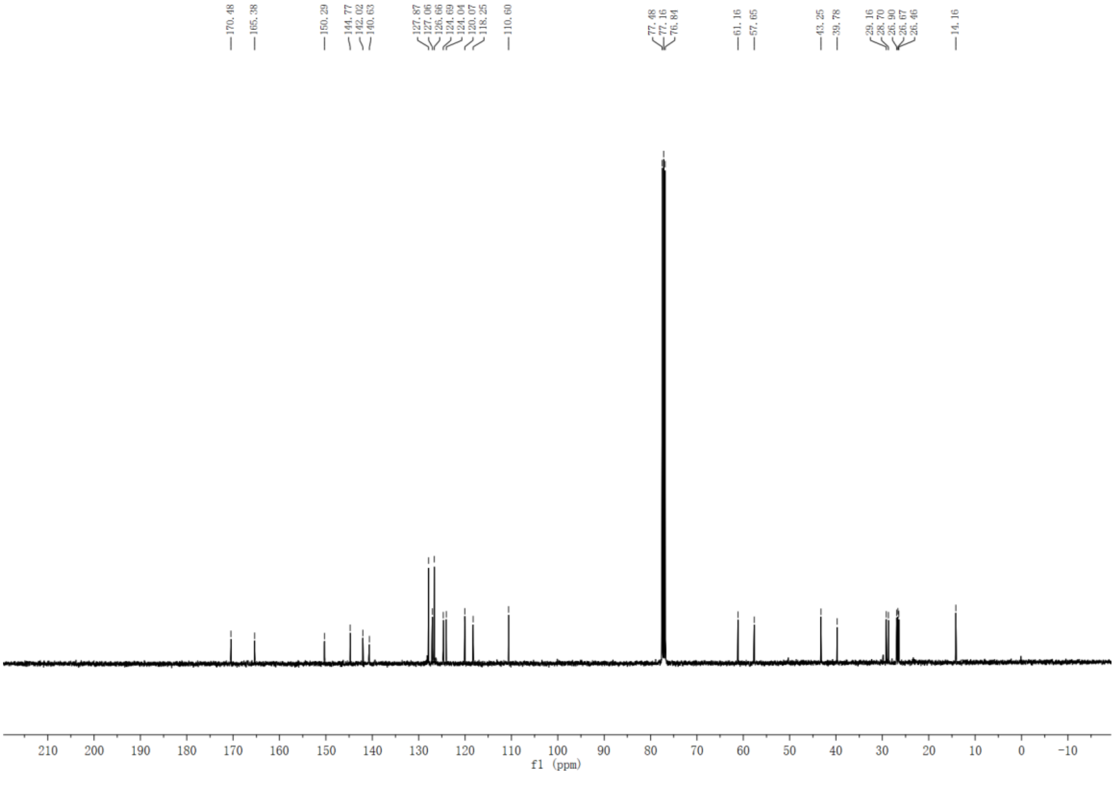

^{1}H NMR spectrum of **8y**

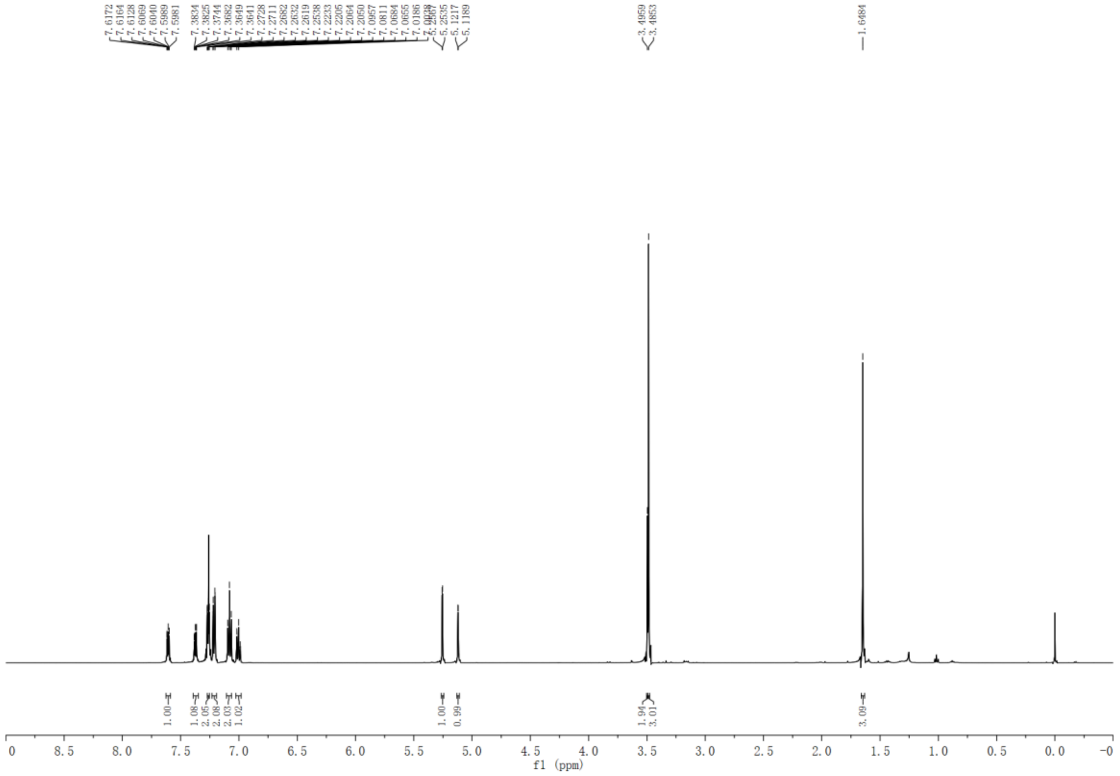

^{13}C NMR spectrum of **8y**

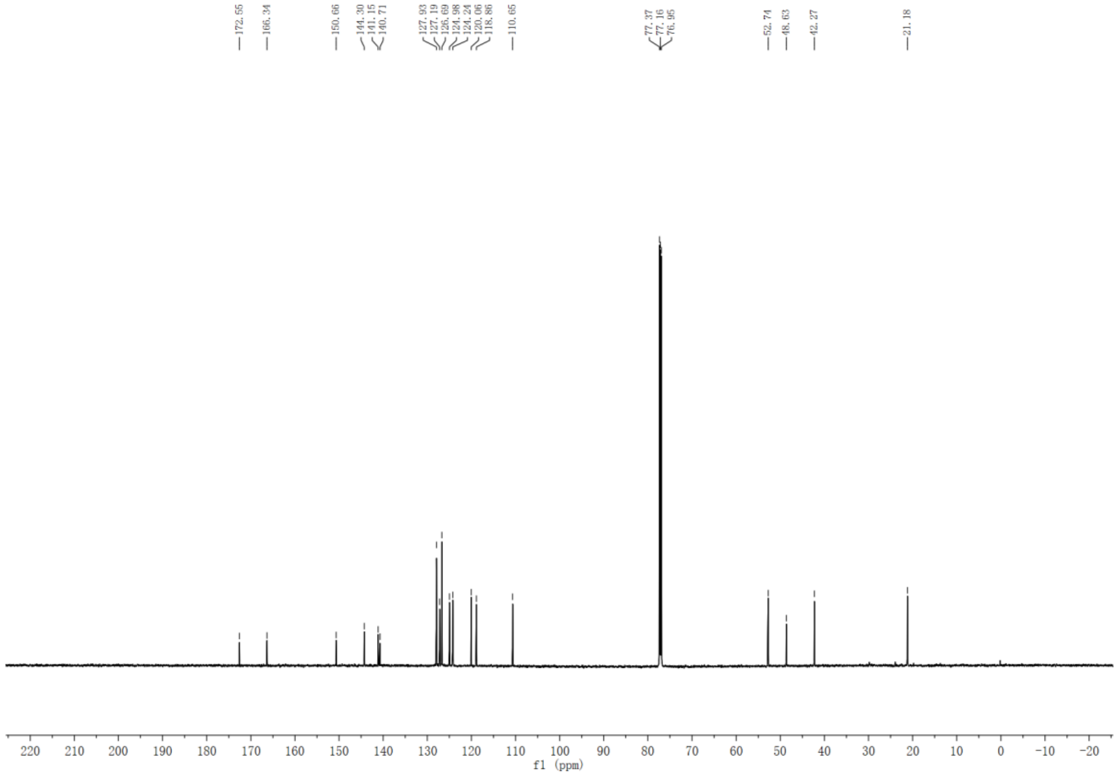

^{1}H NMR spectrum of **8z**

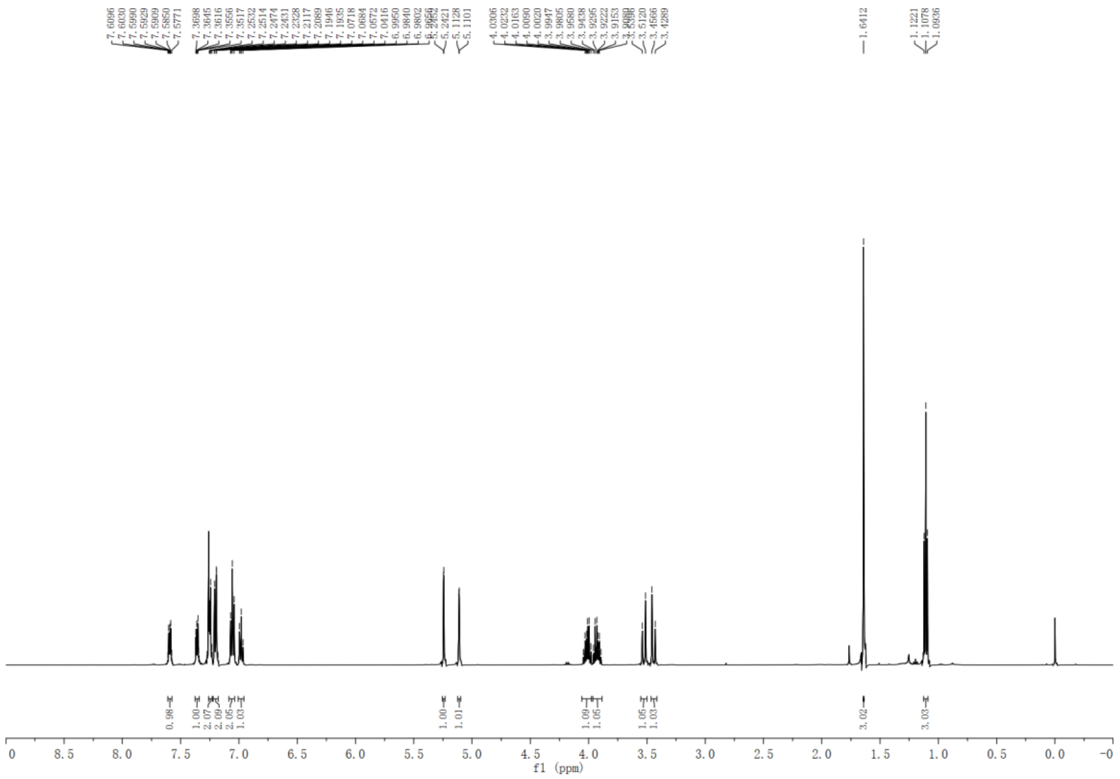

^{13}C NMR spectrum of **8z**

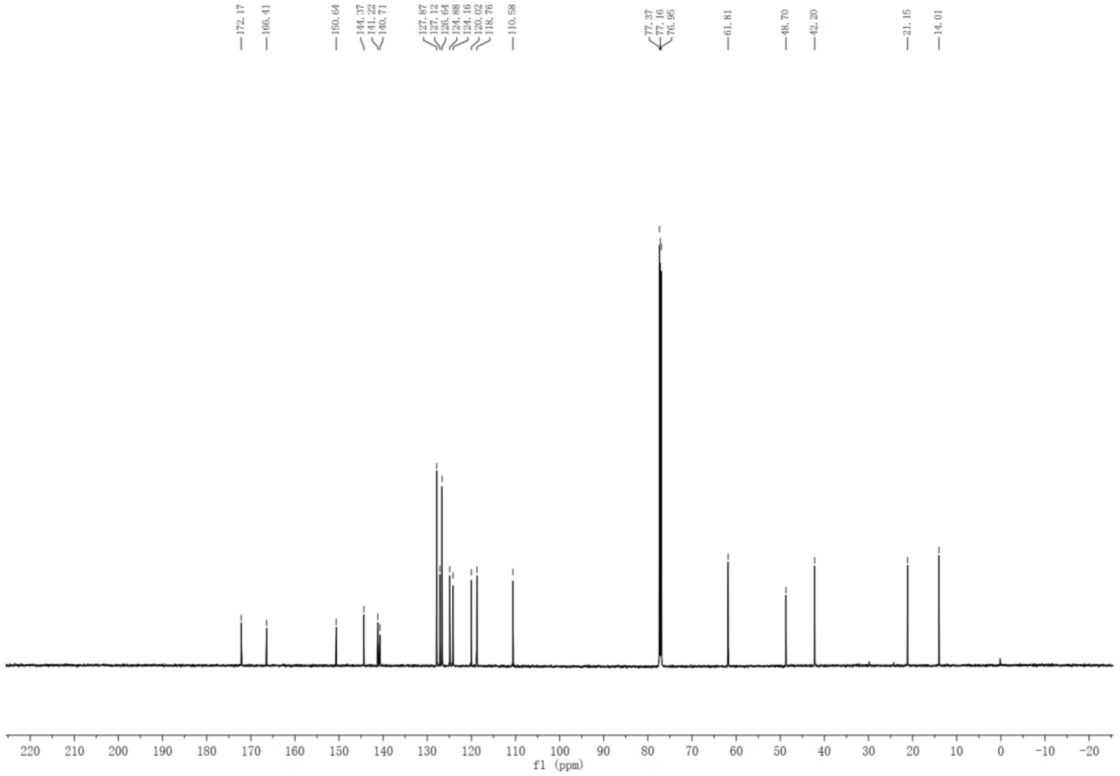

^{1}H NMR spectrum of **8aa**

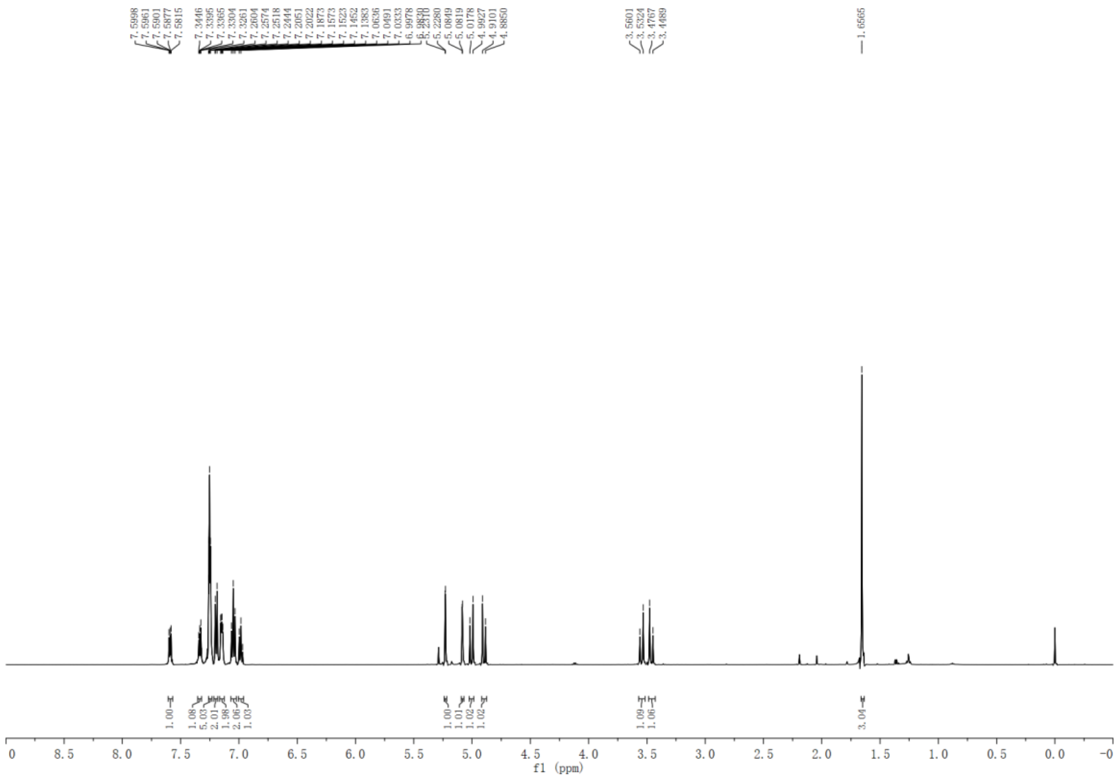

^{13}C NMR spectrum of **8aa**

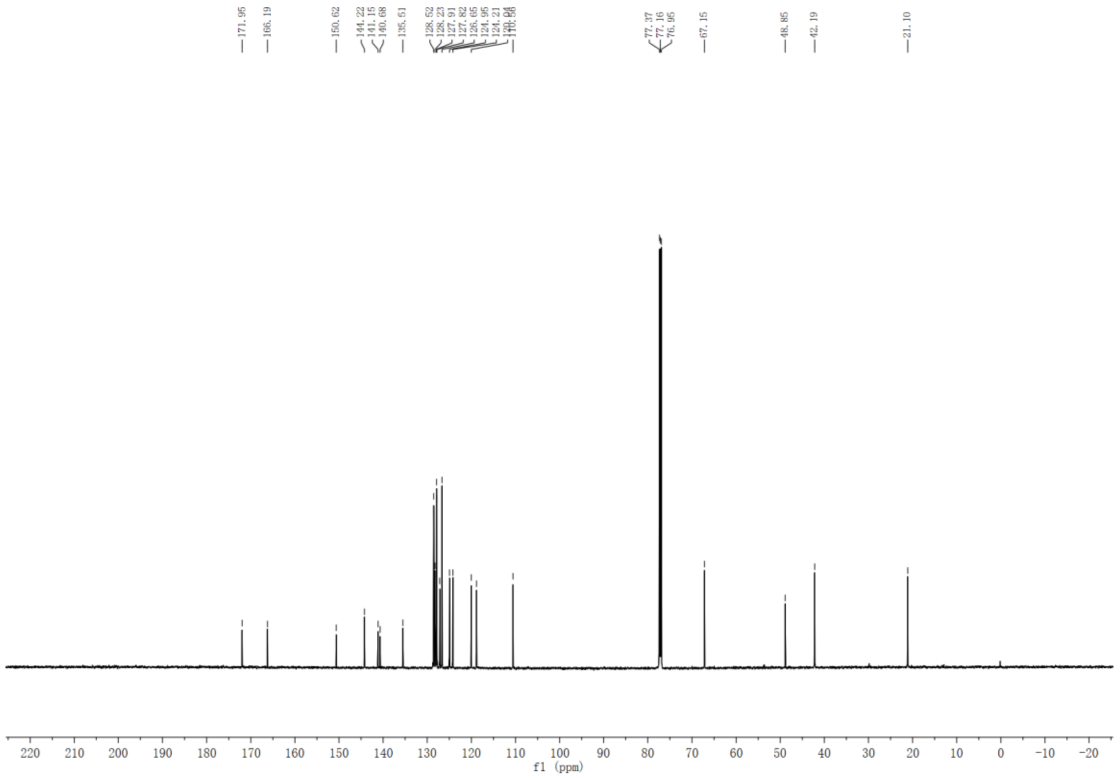

^{1}H NMR spectrum of **9**

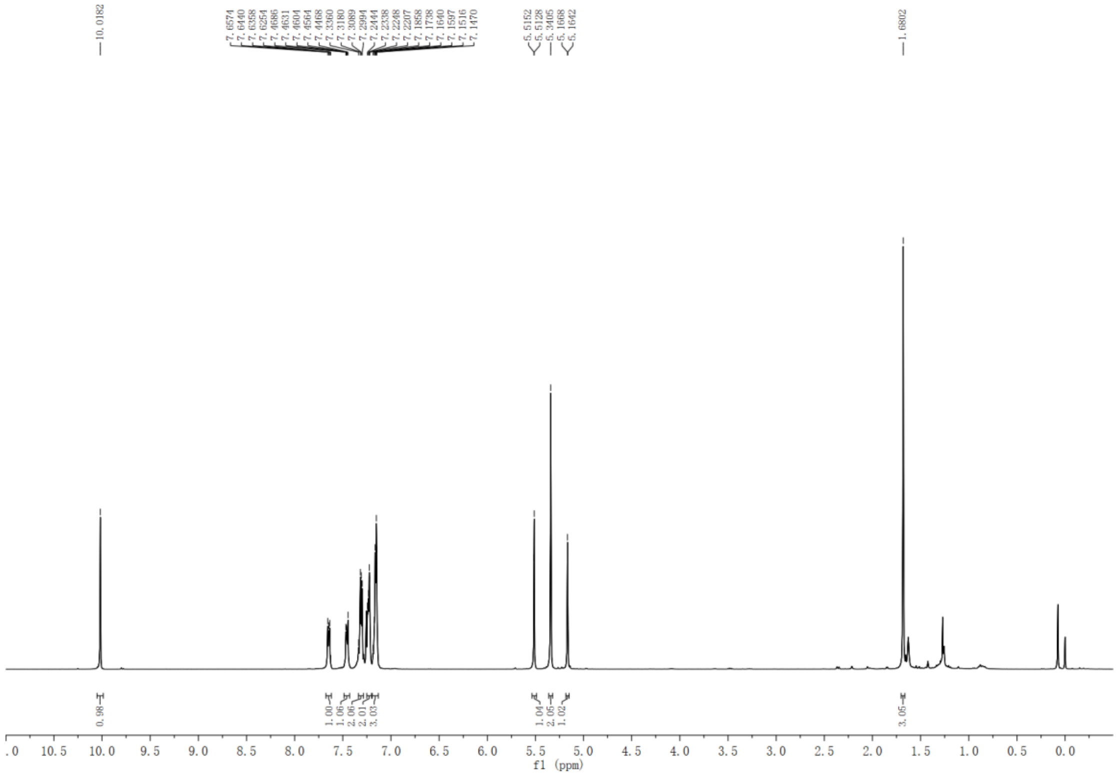

^{13}C NMR spectrum of **9**

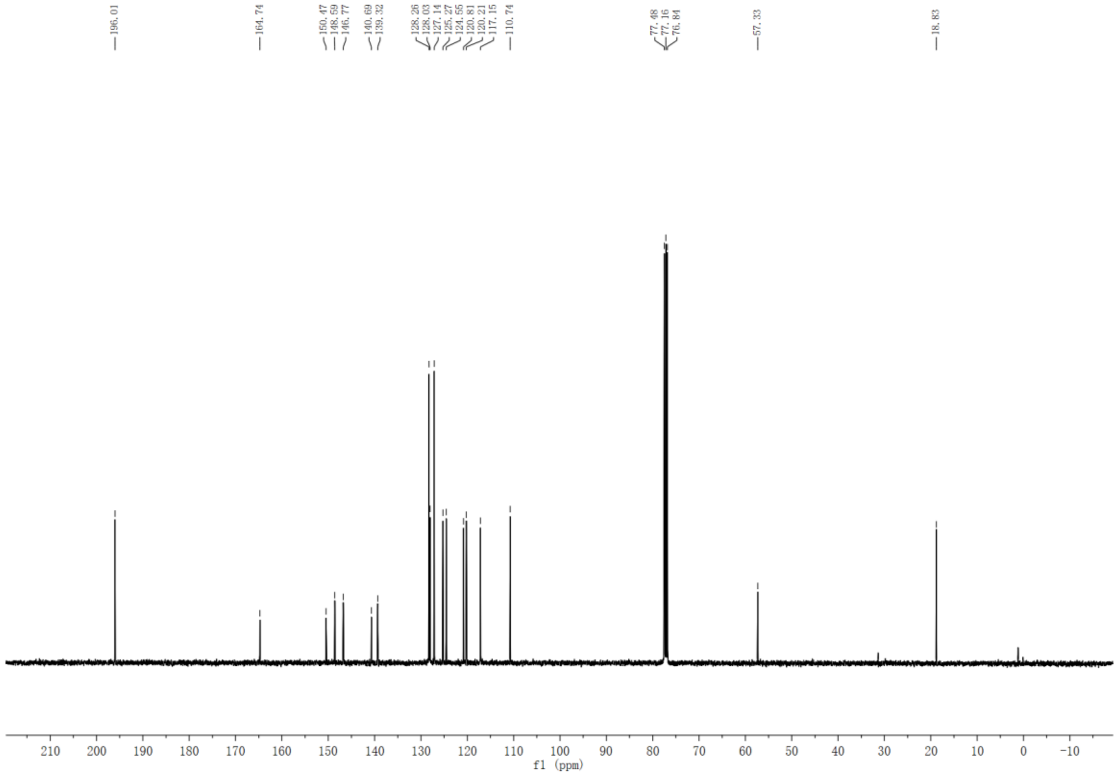

^{1}H NMR spectrum of **10**

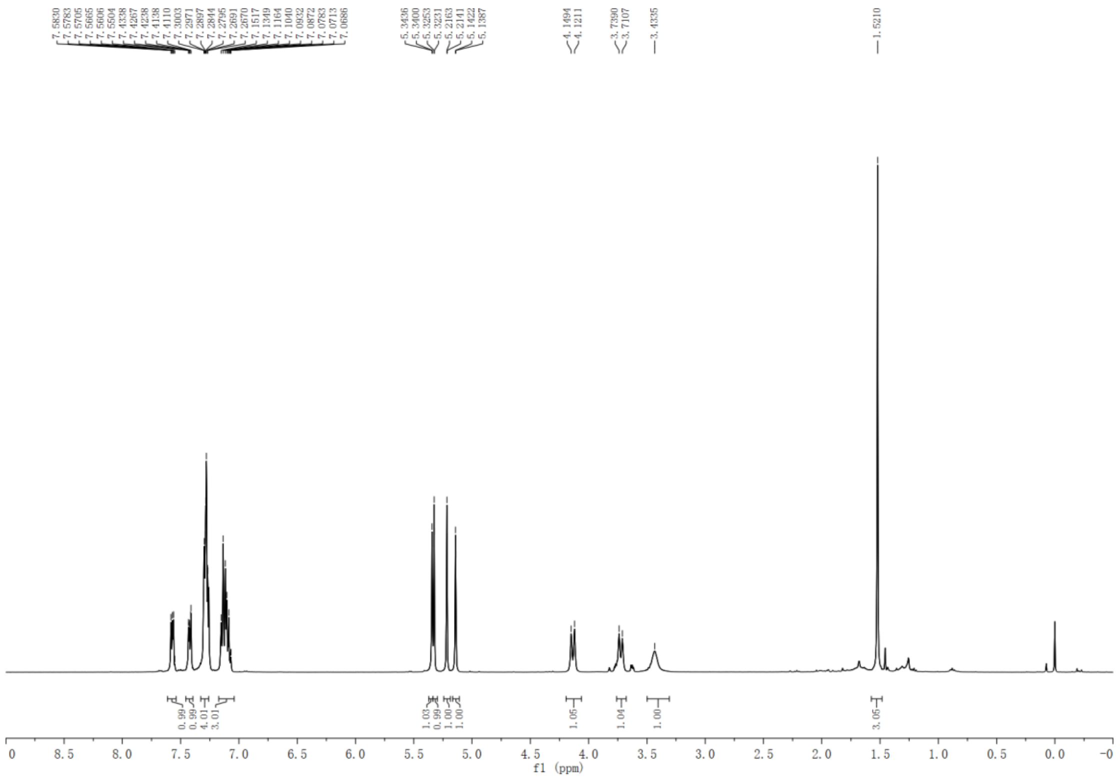

^{13}C NMR spectrum of **10**

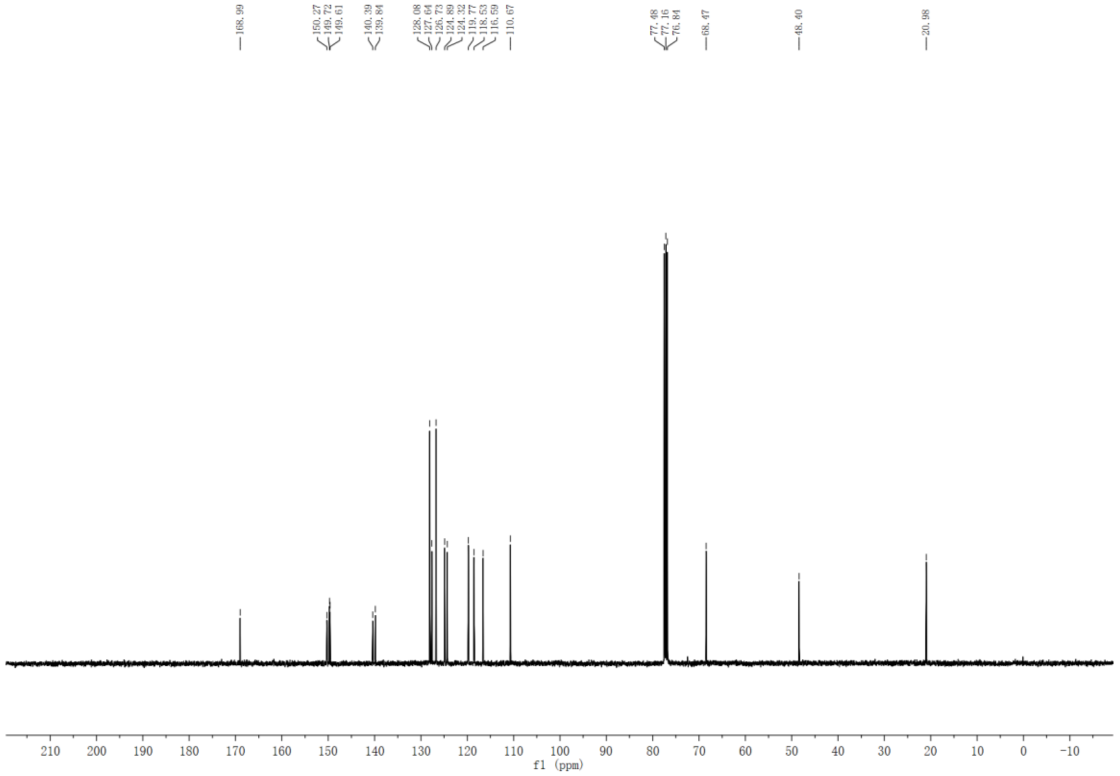

^{1}H NMR spectrum of **11**

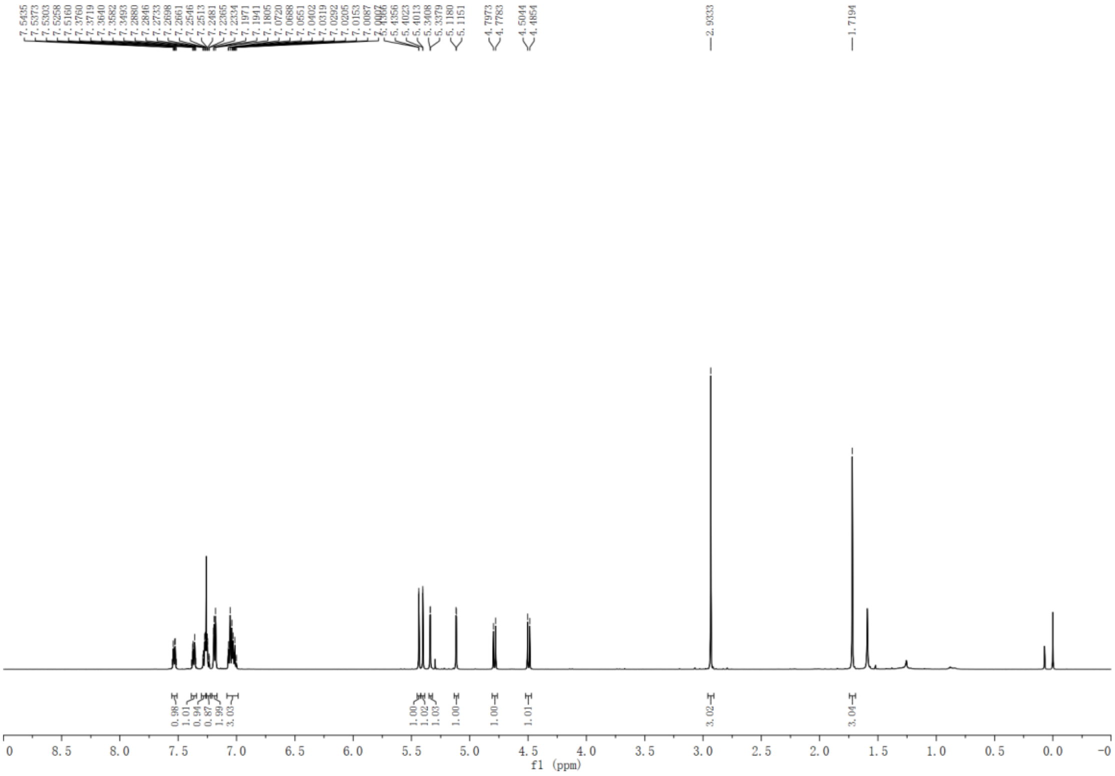

^{13}C NMR spectrum of **11**

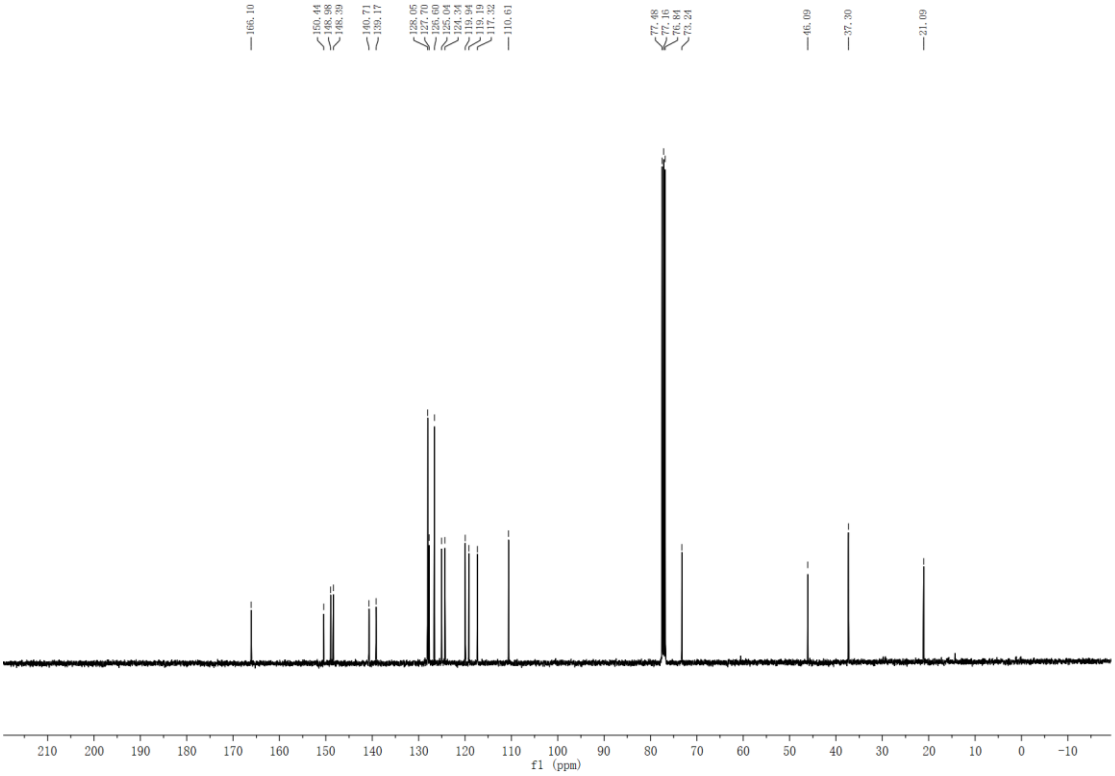

^{1}H NMR spectrum of **12**

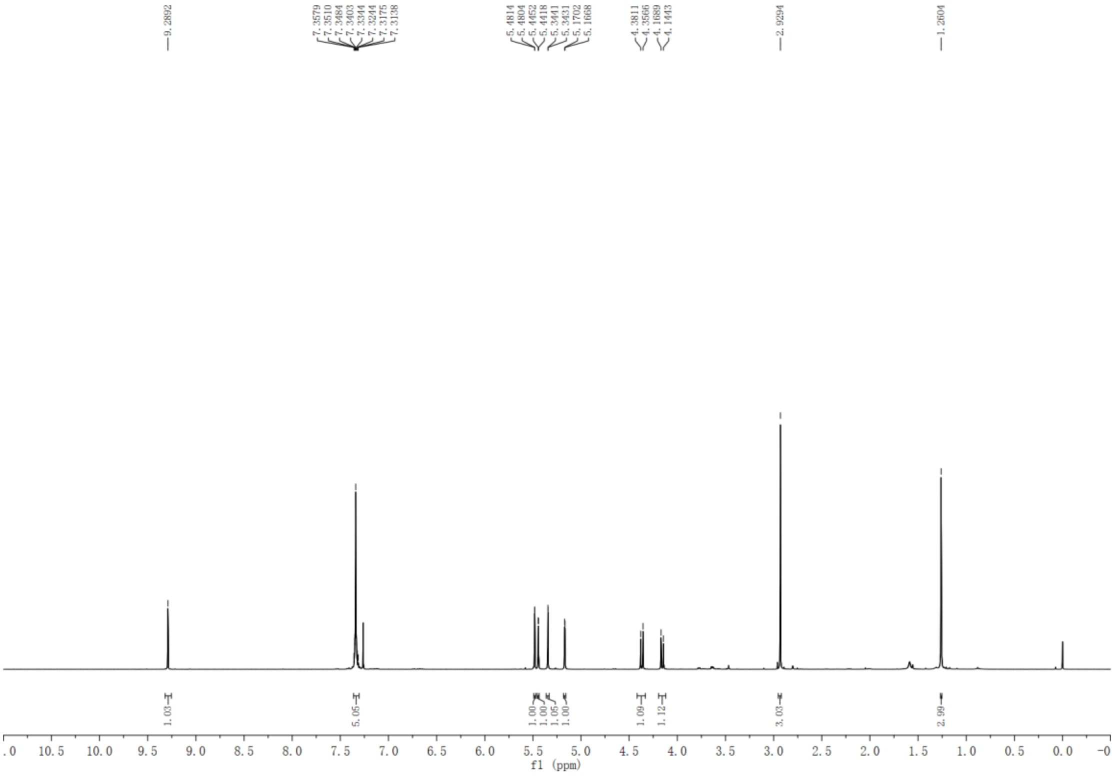

^{13}C NMR spectrum of **12**

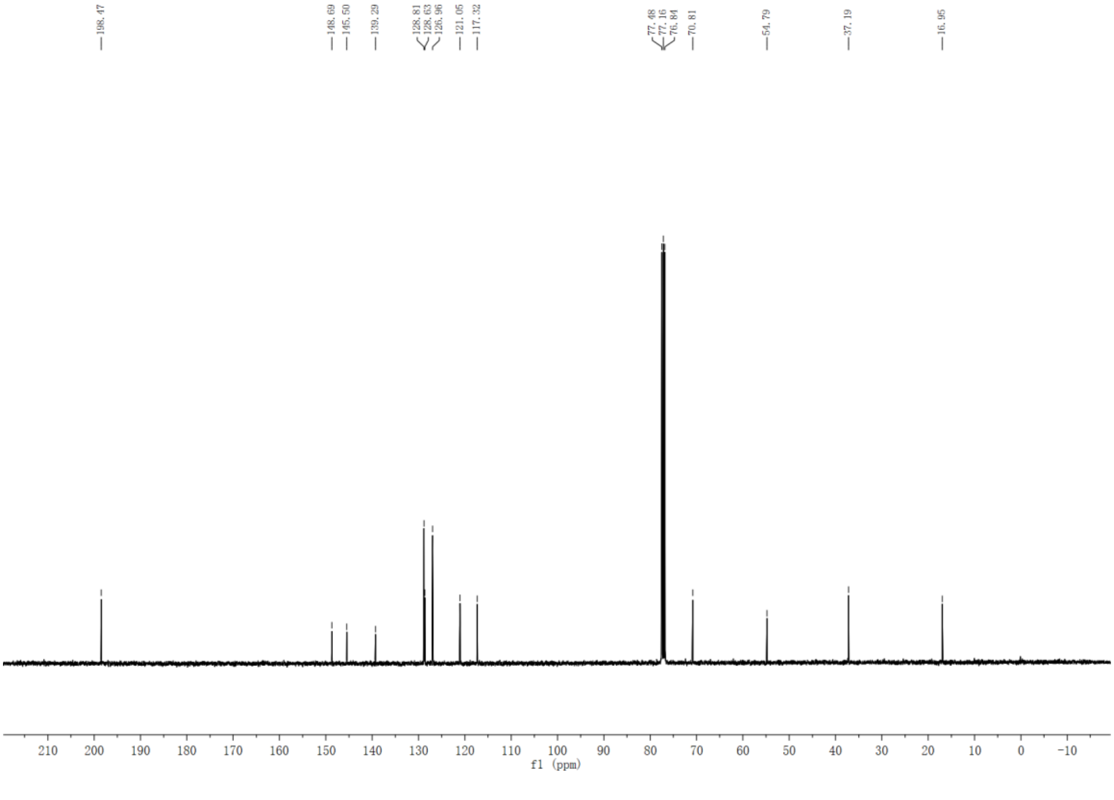

^{1}H NMR spectrum of **13**

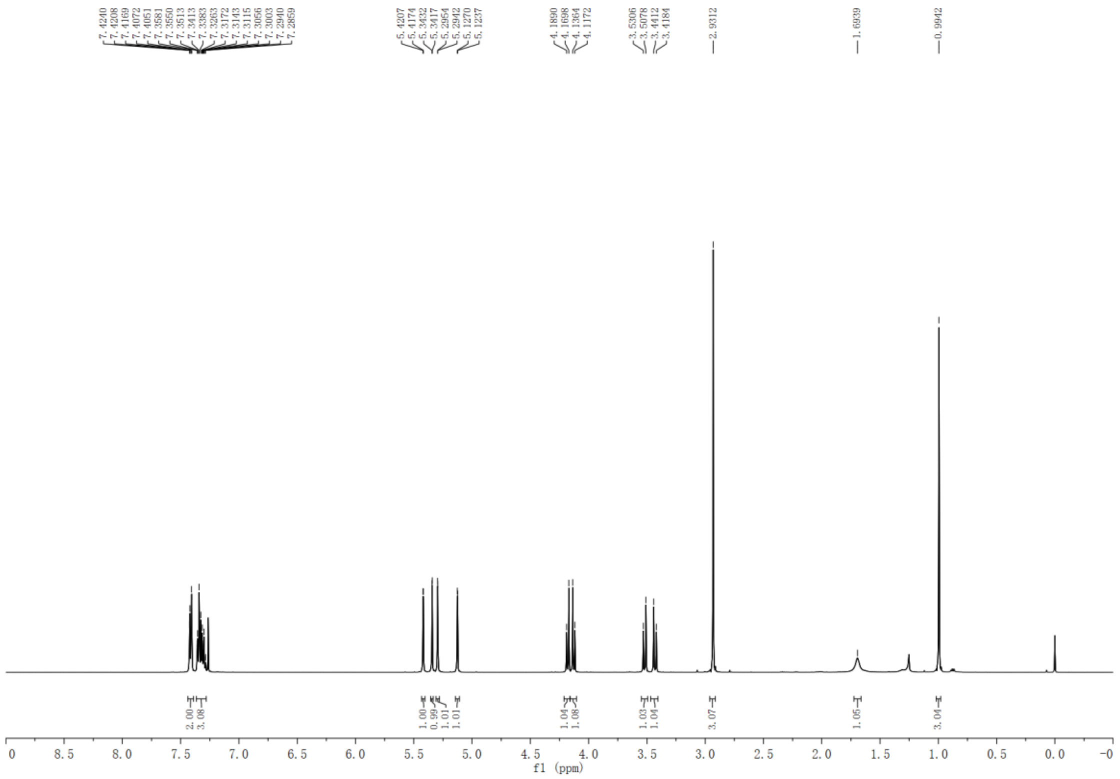

^{13}C NMR spectrum of **13**

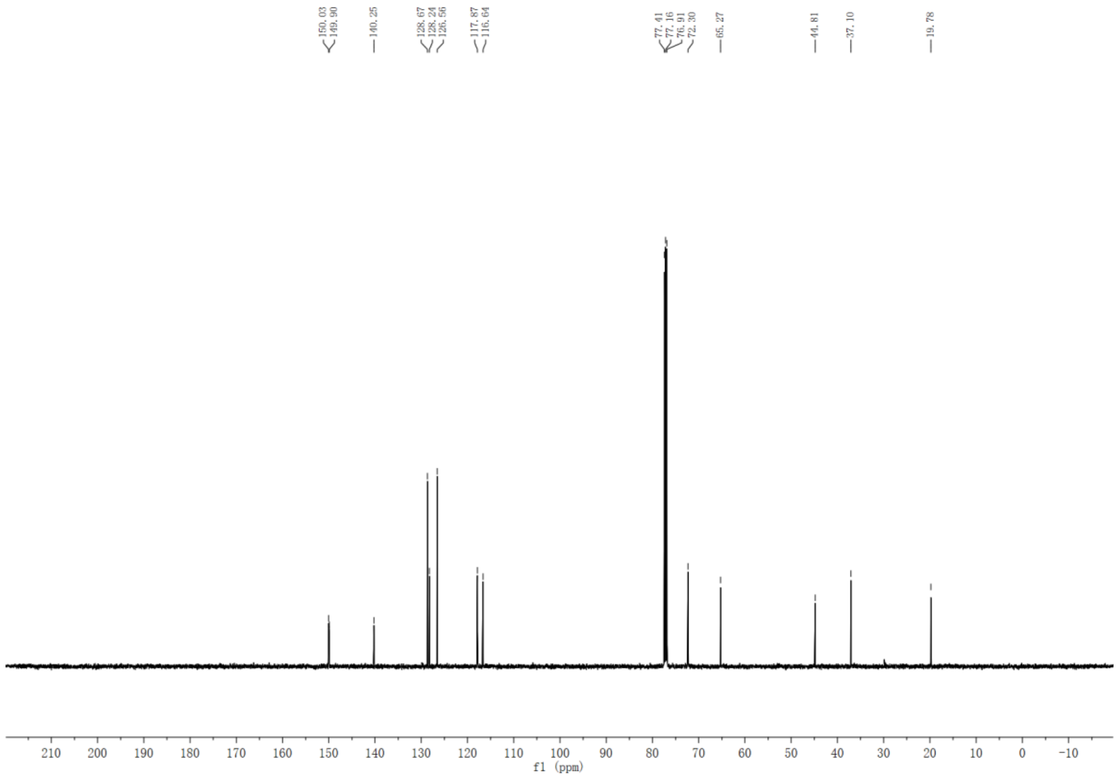

^{1}H NMR spectrum of **14**

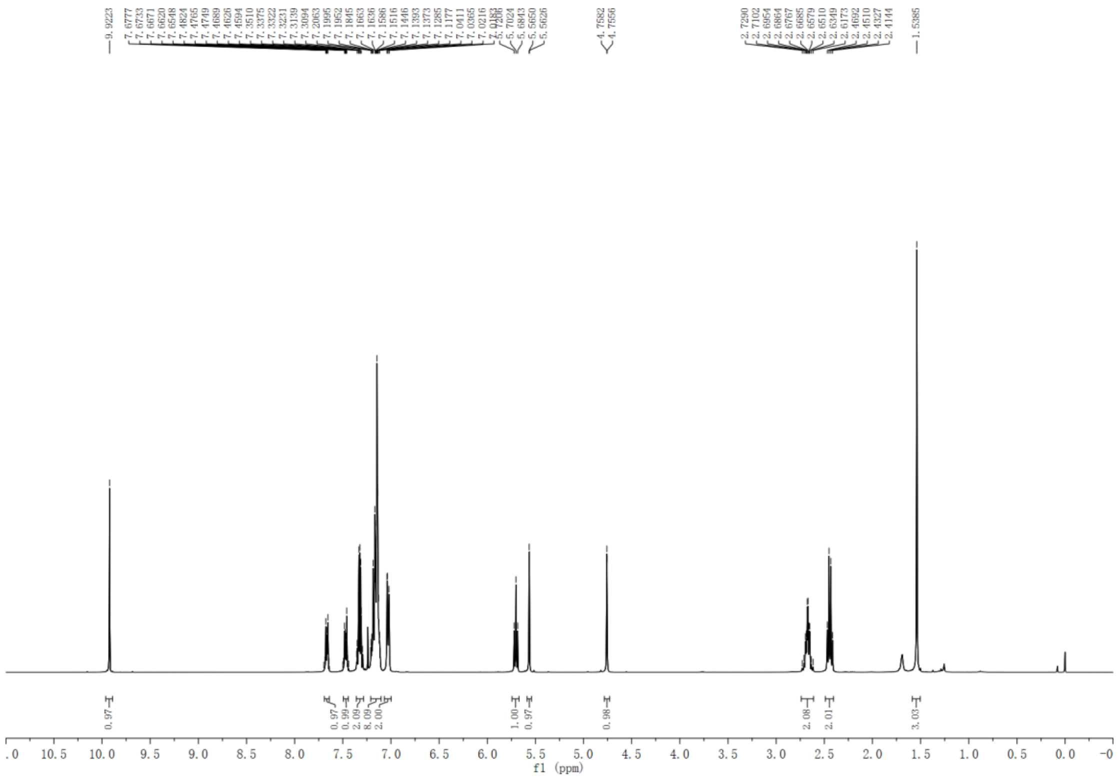

^{13}C NMR spectrum of **14**

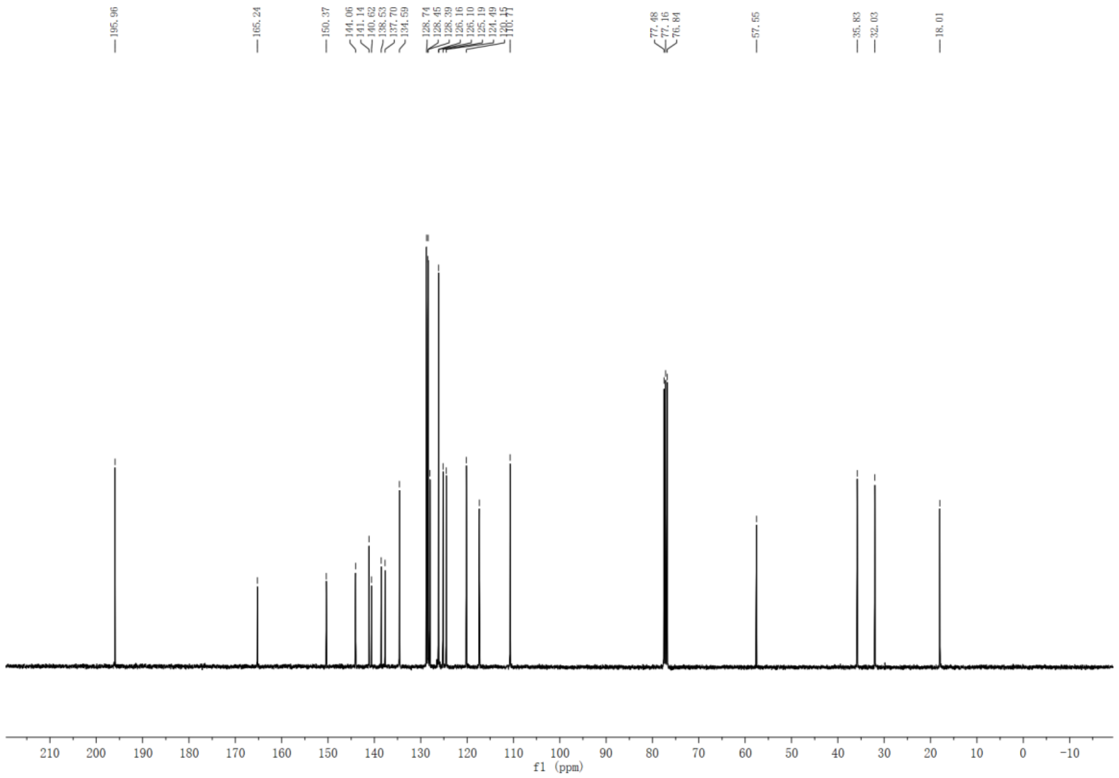

^{1}H NMR spectrum of **15**

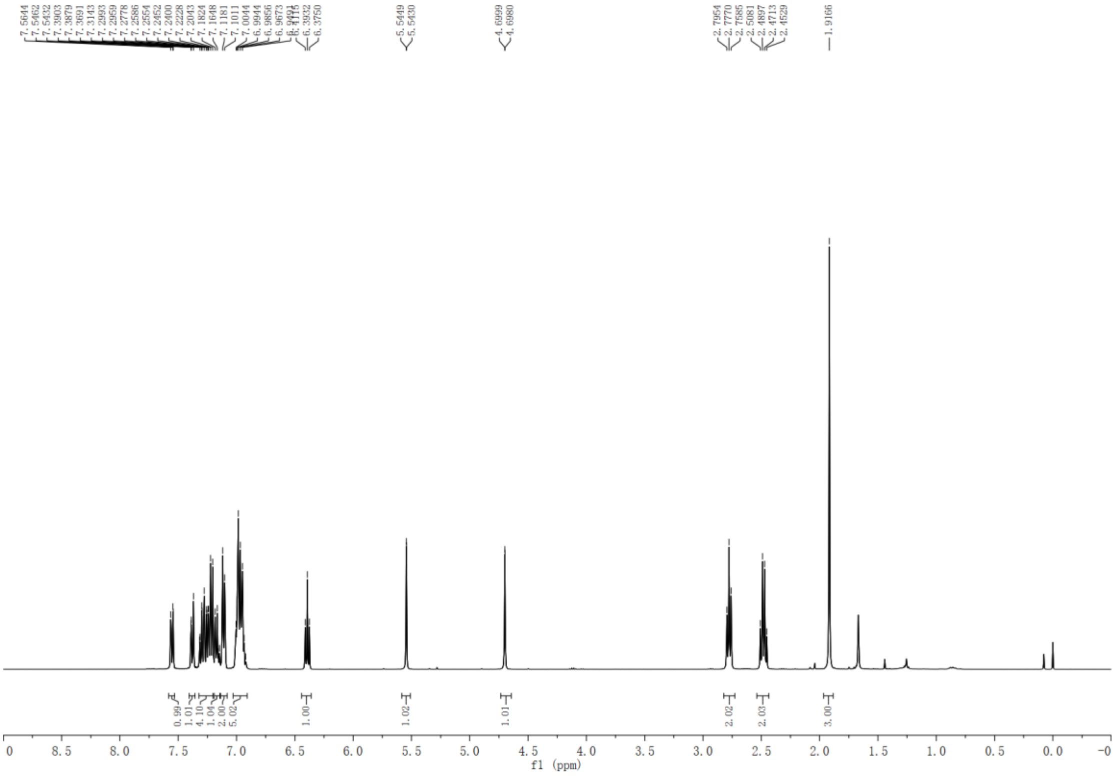

^{13}C NMR spectrum of **15**

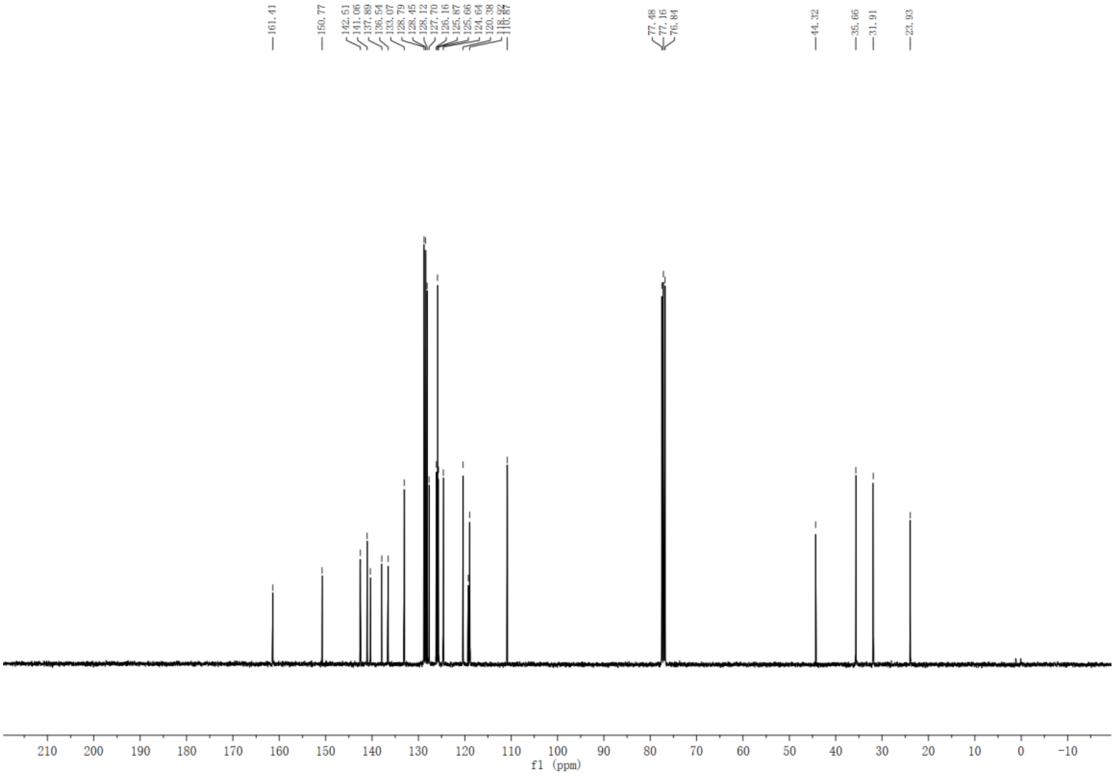

^{1}H NMR spectrum of ***(R,R)*-16** (74:1 dr)

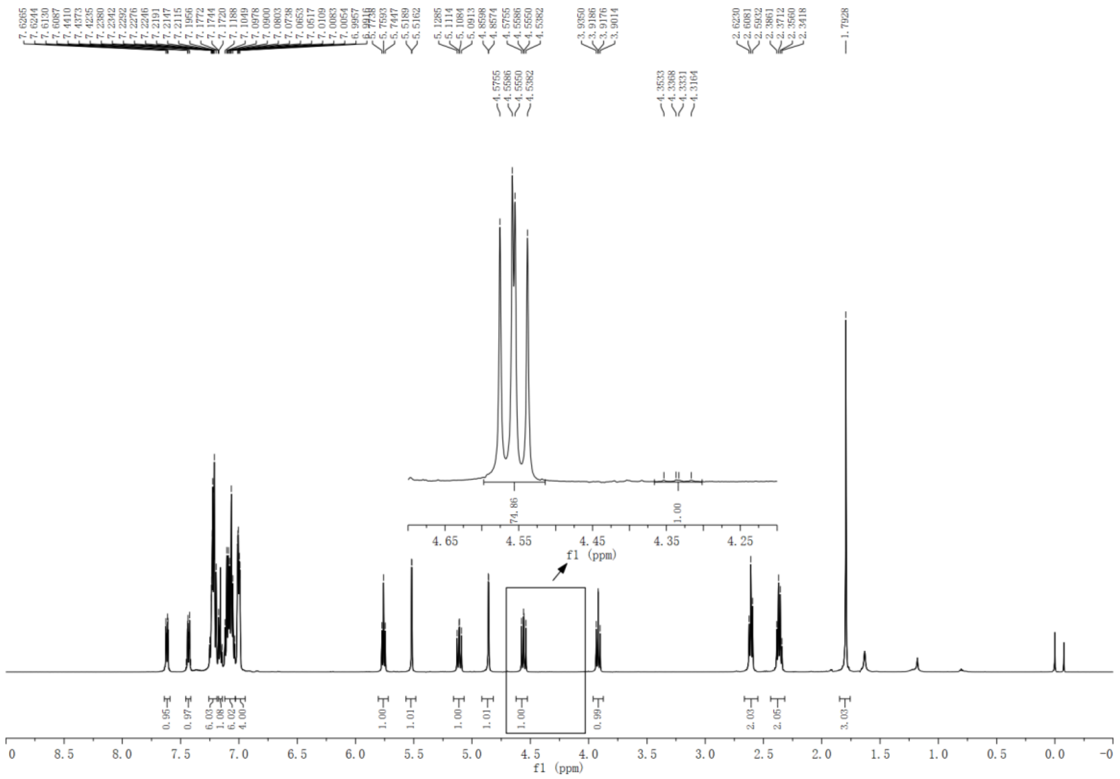

^{13}C NMR spectrum of ***(R,R)*-16**

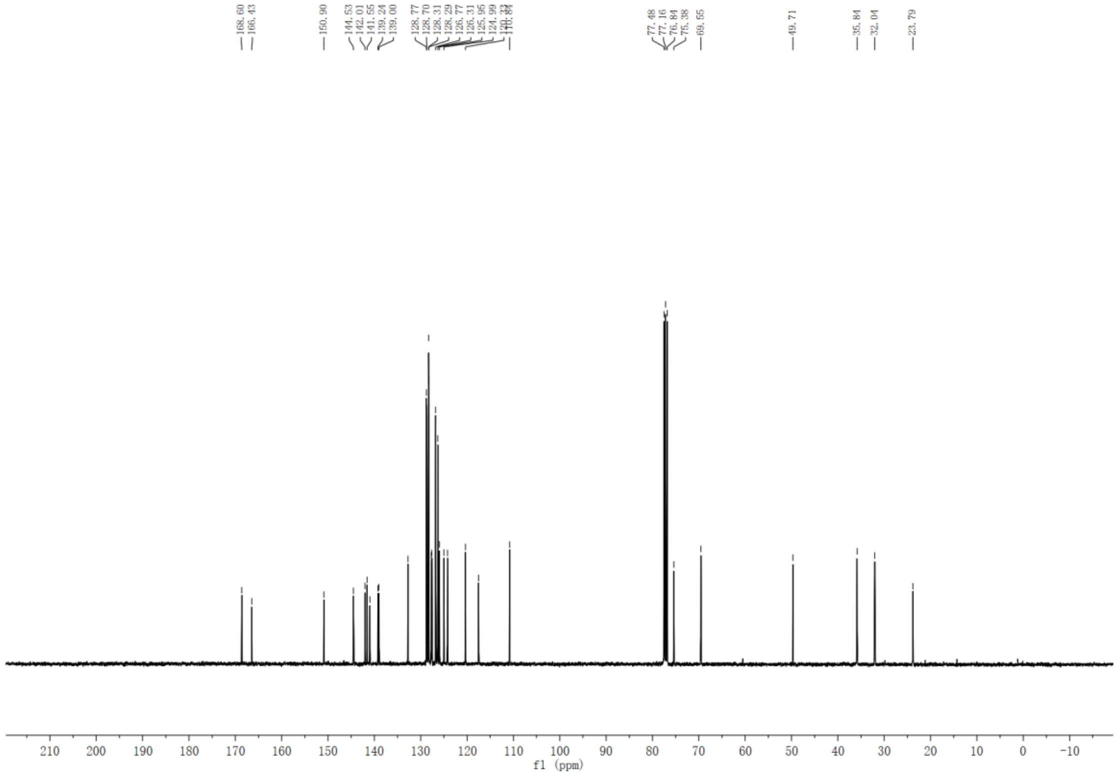

^{1}H NMR spectrum of ***(R,S)*-16** (21:1 dr)

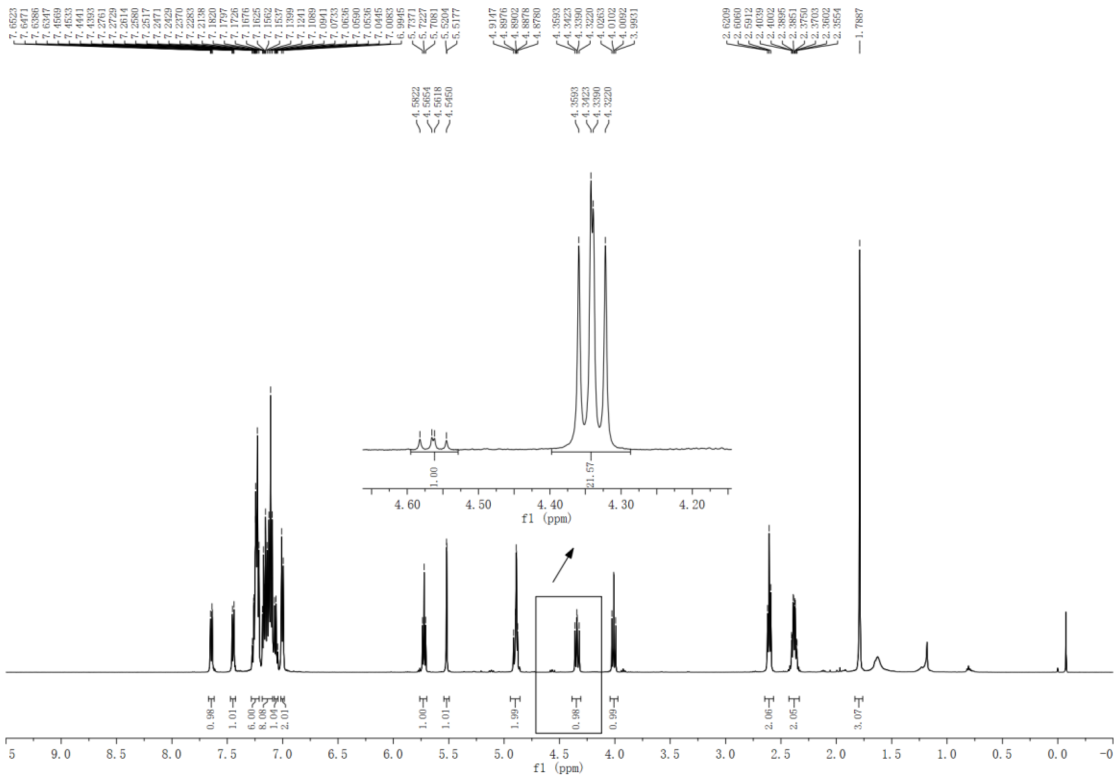

^{13}C NMR spectrum of ***(R,S)*-16**

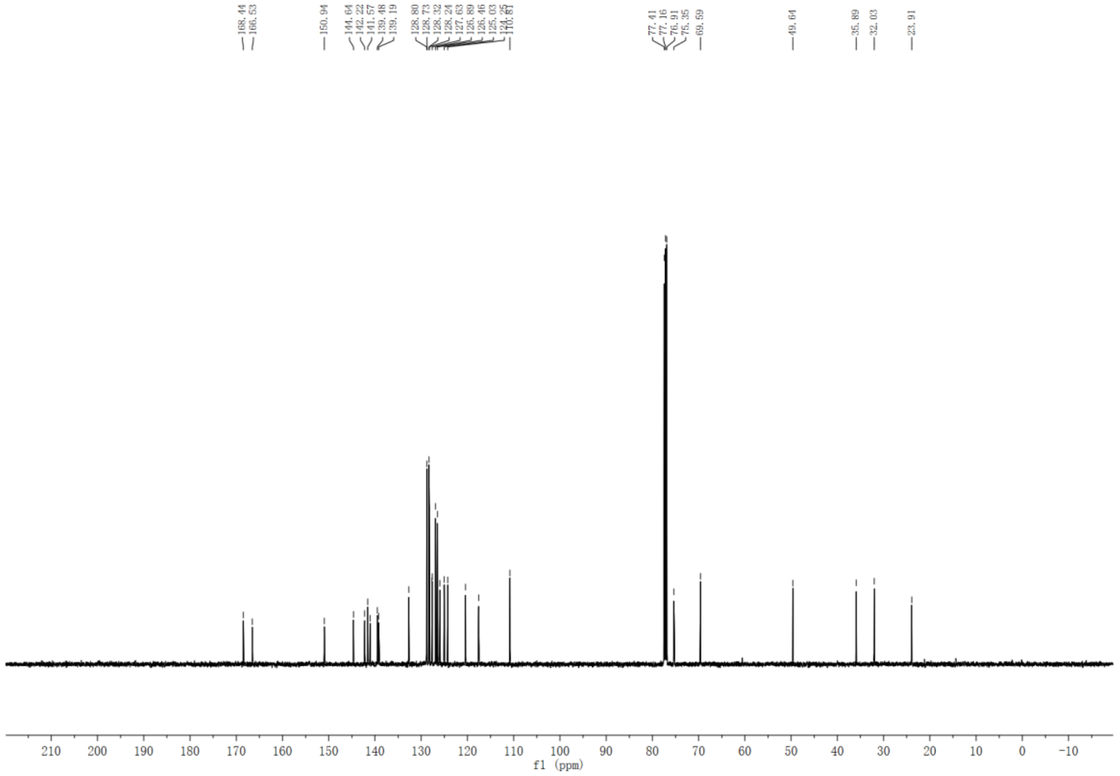

^{1}H NMR spectrum of **17**

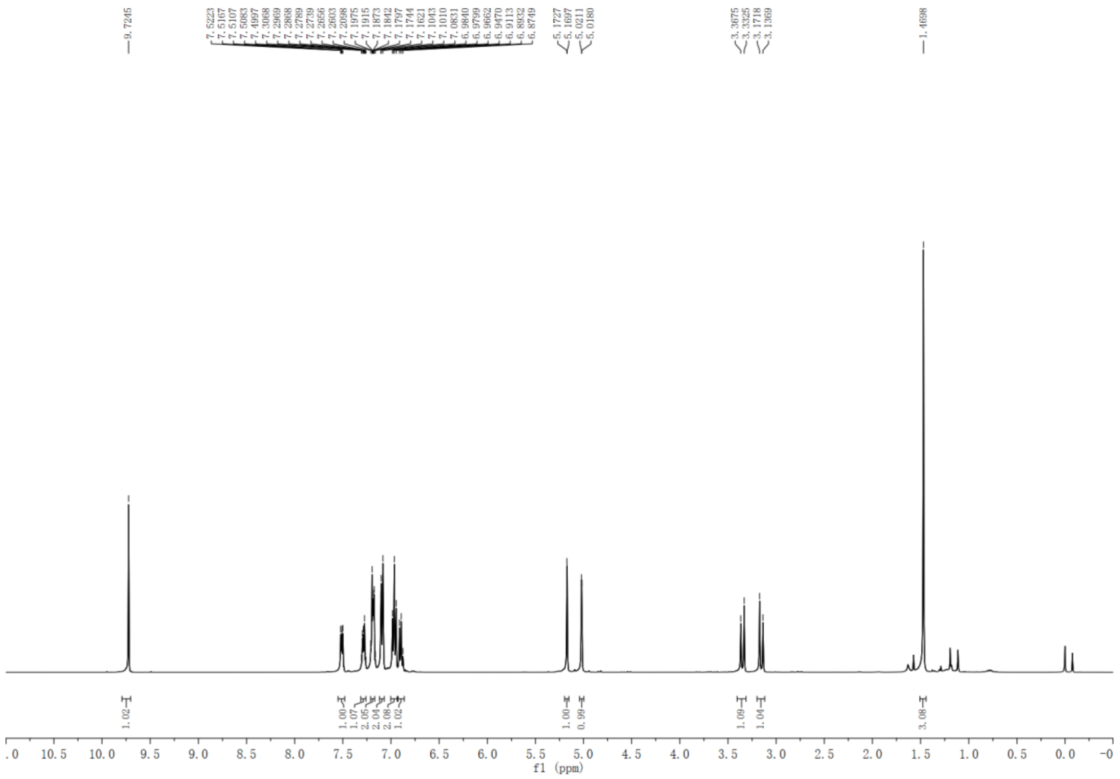

^{13}C NMR spectrum of **17**

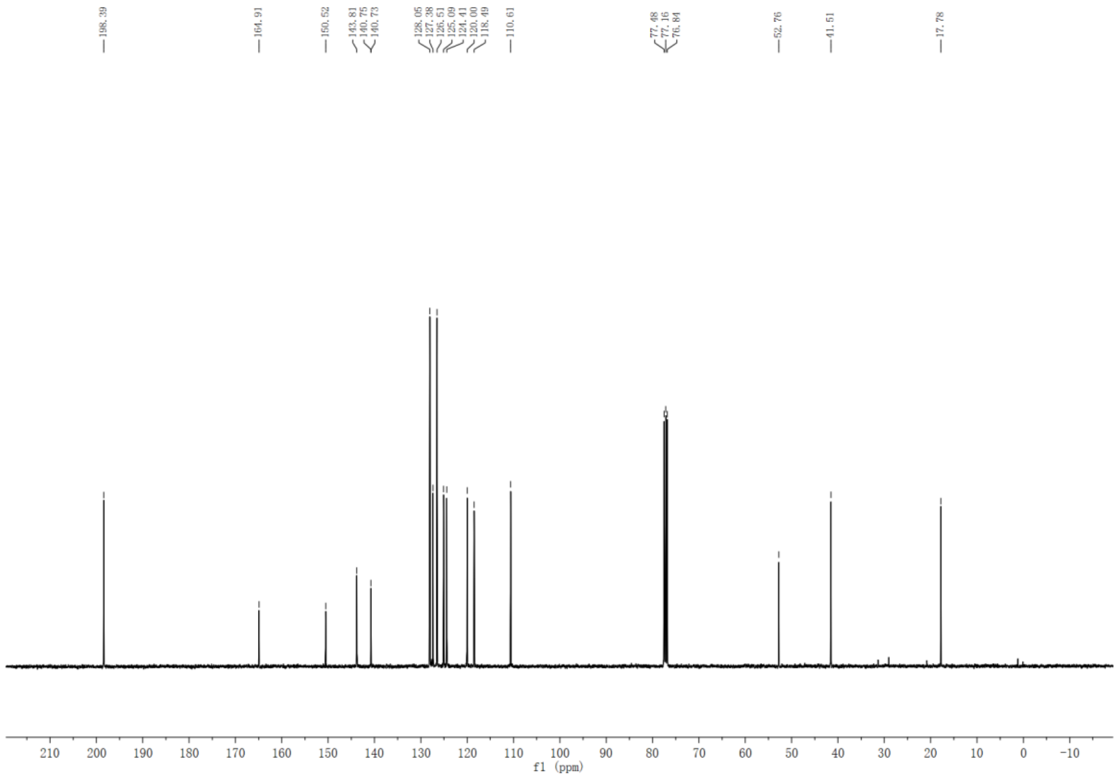

^{1}H NMR spectrum of **18**

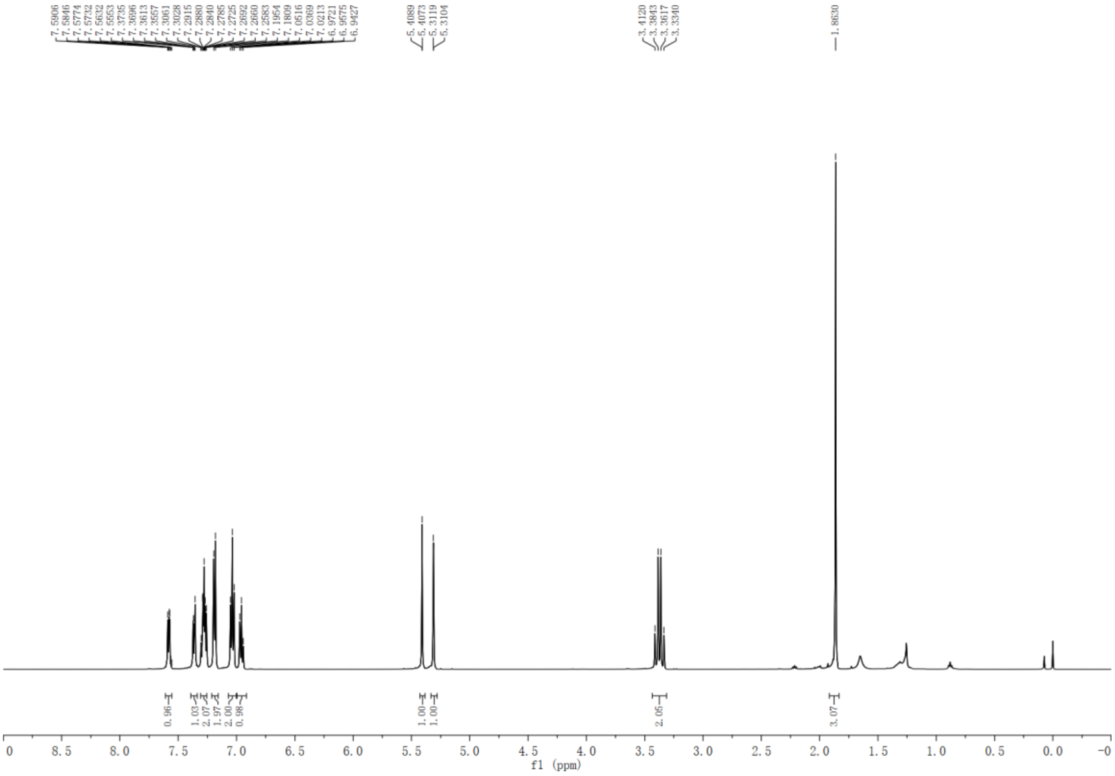

^{13}C NMR spectrum of **18**

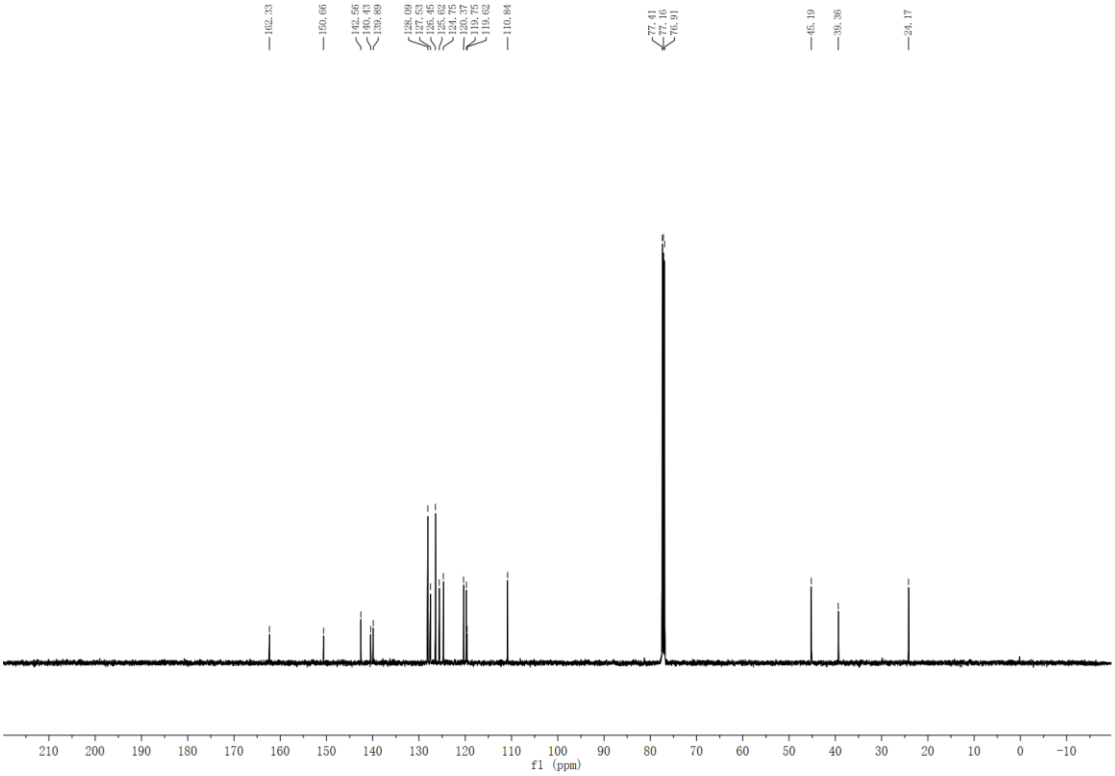

^{1}H NMR spectrum of ***(R,R)*-19** (29:1 dr)

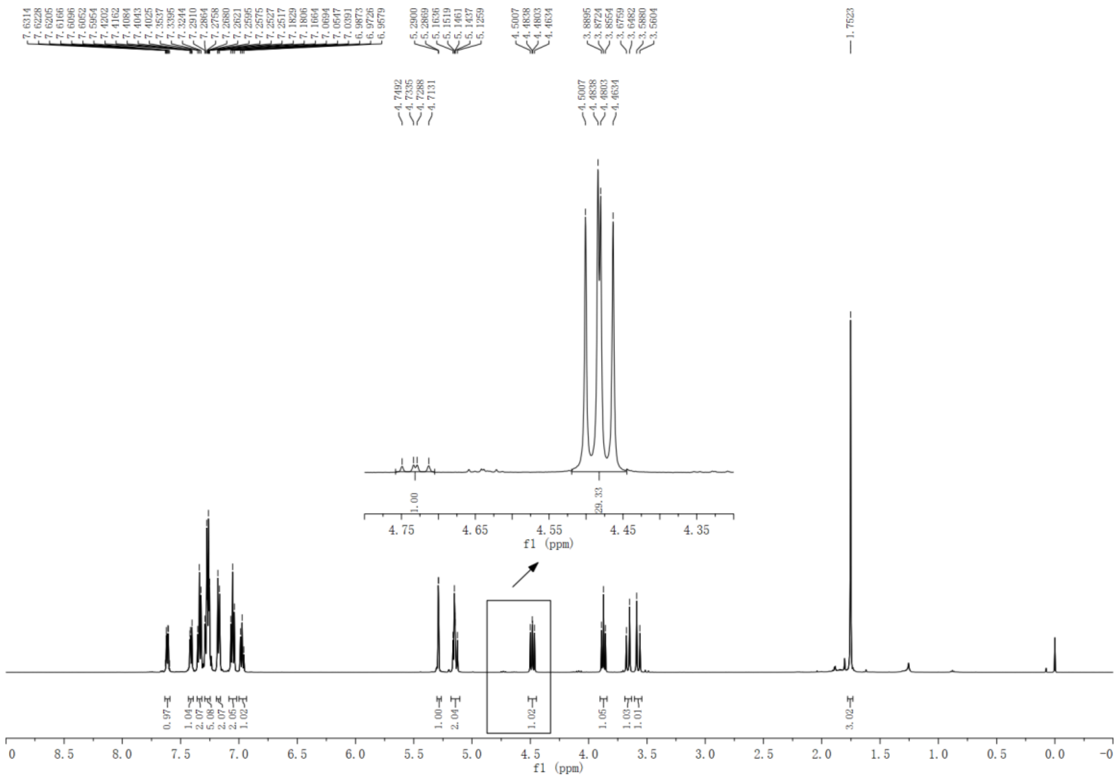

^{13}C NMR spectrum of ***(R,R)*-19**

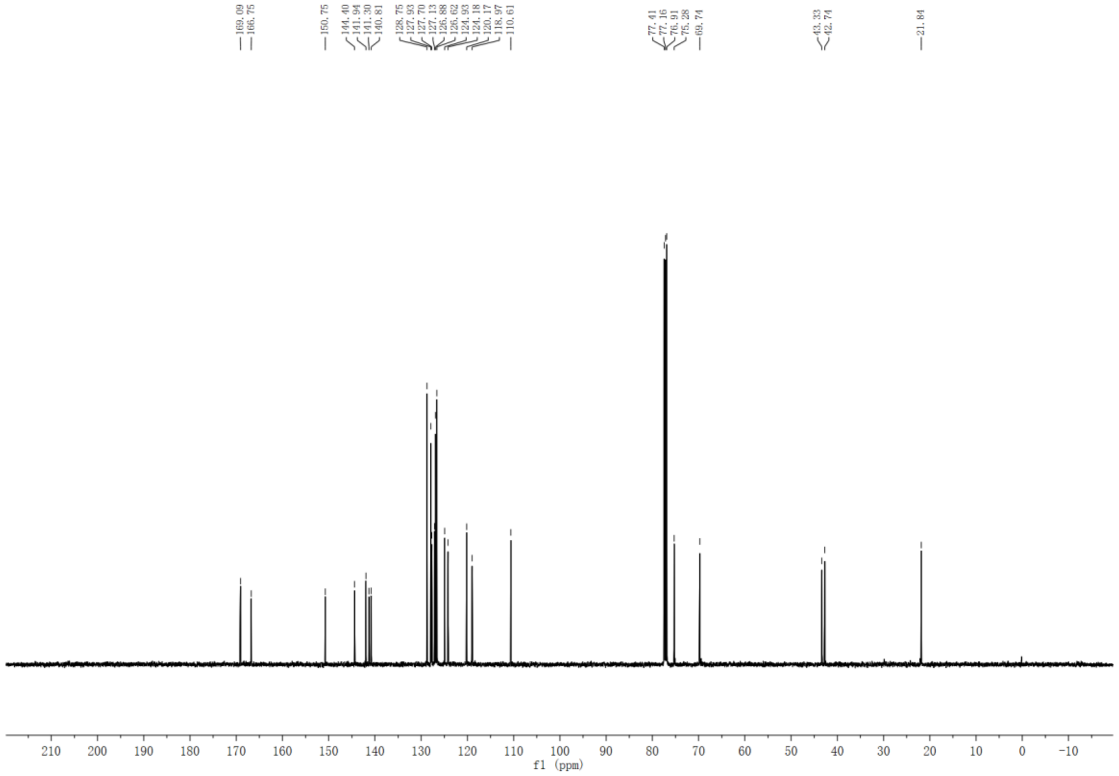

^{1}H NMR spectrum of ***(R,S)*-19** (18:1 dr)

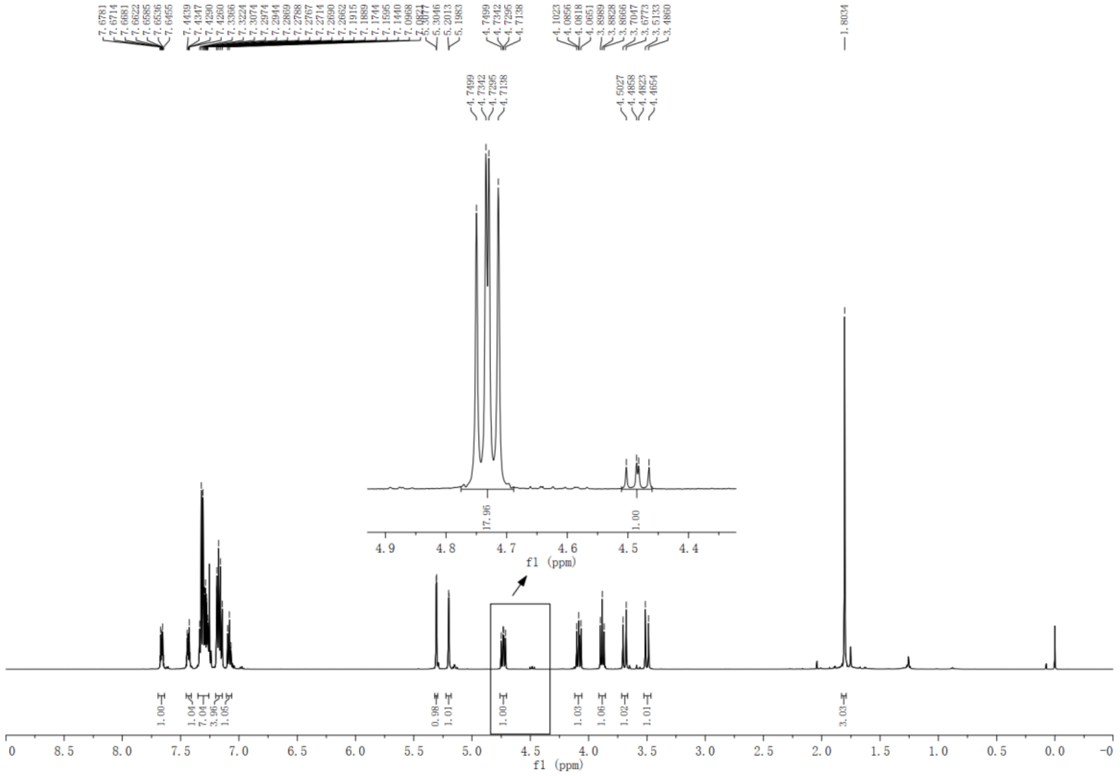

^{13}C NMR spectrum of ***(R,S)*-19**

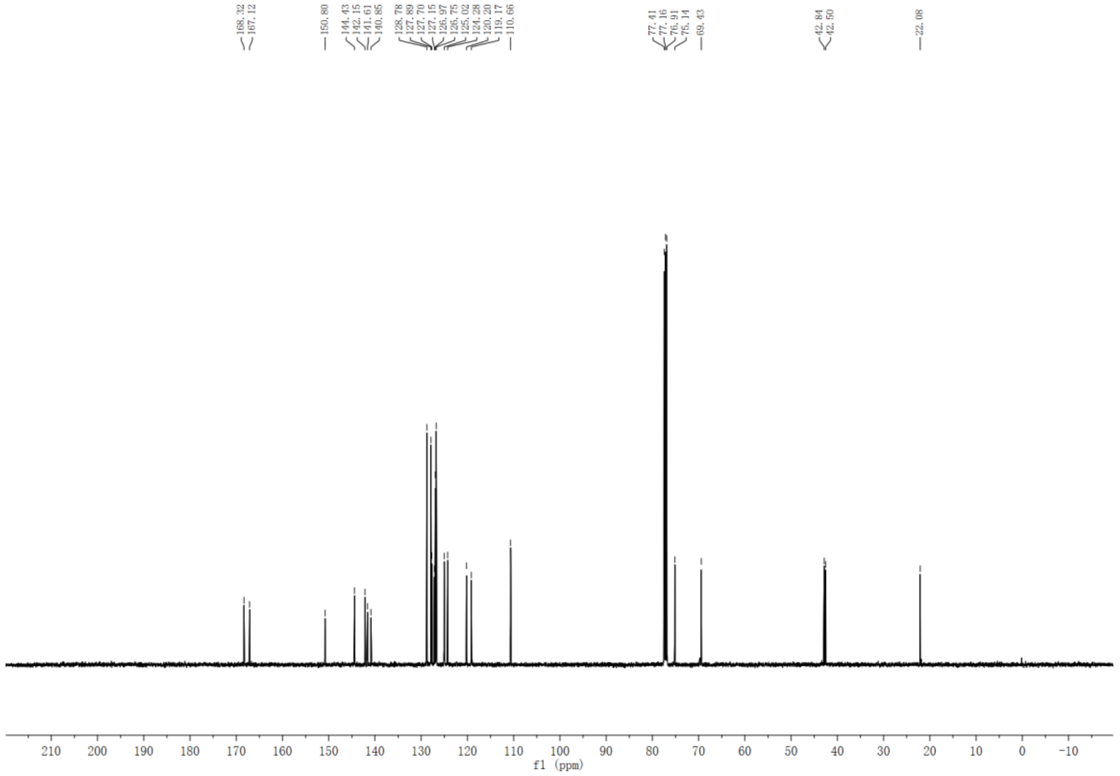

13. HPLC traces

Rac-**3a**

SAMPLE INFORMATION

Sample Name:	zql-13-283-RAC-IC-5%	Acquired By:	System
Sample Type:	Unknown	Sample Set Name:	0
Vial:	13	Acq. Method Set:	5%210400
Injection #:	1	Processing Method:	133391
Injection Volume:	10.00 ul	Channel Name:	254.0nm
Run Time:	8.0 Minutes	Proc. Chnl. Descr.:	2998 PDA 254.0 nm (2998
Date Acquired:	1/6/2023 9:08:26 PM CST		
Date Processed:	1/6/2023 9:56:54 PM CST		

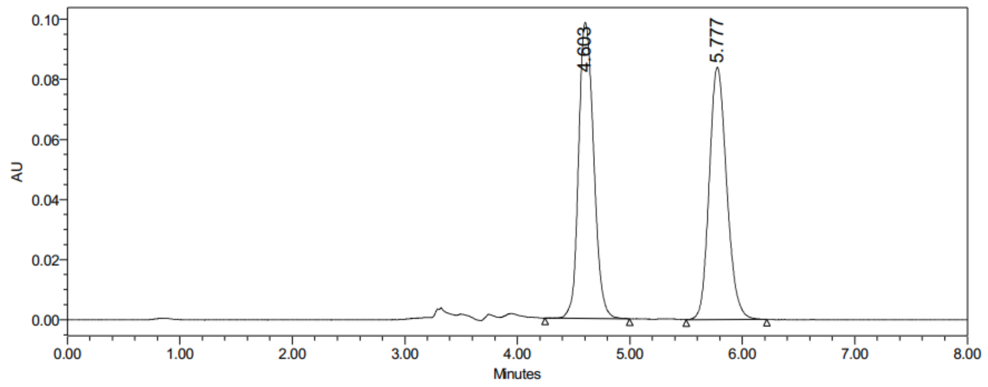

	RT	Area	% Area	Height
1	4.603	917852	50.20	98570
2	5.777	910503	49.80	84005

Asy-**3a**

SAMPLE INFORMATION

Sample Name:	zql-13-339-ASY-IC-5%	Acquired By:	System
Sample Type:	Unknown	Sample Set Name:	0
Vial:	17	Acq. Method Set:	5%210400
Injection #:	1	Processing Method:	13392
Injection Volume:	10.00 ul	Channel Name:	254.0nm
Run Time:	8.0 Minutes	Proc. Chnl. Descr.:	2998 PDA 254.0 nm (2998
Date Acquired:	1/6/2023 9:43:10 PM CST		
Date Processed:	1/6/2023 9:58:04 PM CST		

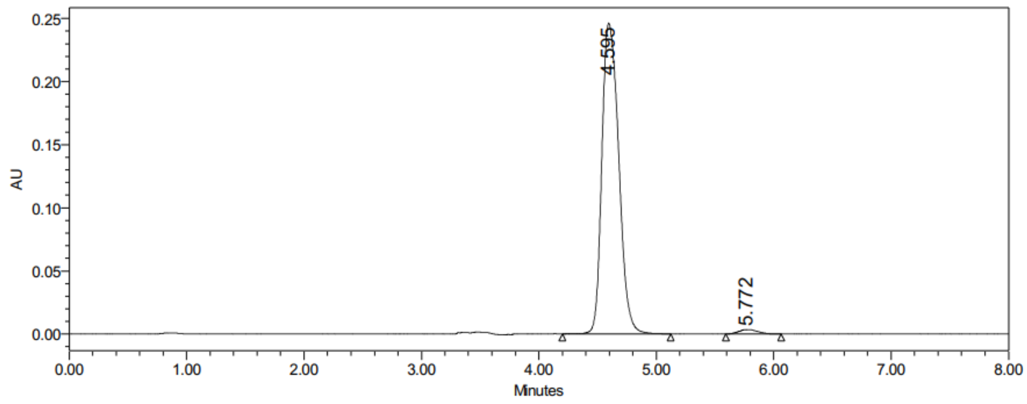

	RT	Area	% Area	Height
1	4.595	2450746	98.41	246053
2	5.772	39548	1.59	3494

Rac-**3b**

SAMPLE INFORMATION

Sample Name:	zql-13-277-RAC-IC-5%	Acquired By:	System
Sample Type:	Unknown	Sample Set Name	0
Vial:	12	Acq. Method Set:	5%210400
Injection #:	1	Processing Method	133081
Injection Volume:	10.00 ul	Channel Name:	254.0nm
Run Time:	10.0 Minutes	Proc. Chnl. Descr.:	2998 PDA 254.0 nm (2998
Date Acquired:	12/23/2022 19:20:19 CST		
Date Processed:	1/4/2023 20:23:41 CST		

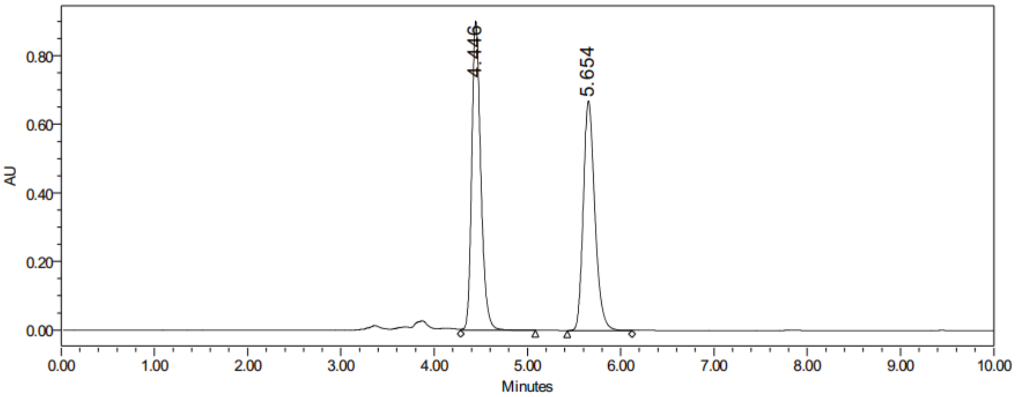

	RT	Area	% Area	Height
1	4.446	6063534	51.13	901579
2	5.654	5794743	48.87	669581

Asy-**3b**

SAMPLE INFORMATION

Sample Name:	zql-13-308-IC-5%	Acquired By:	System
Sample Type:	Unknown	Sample Set Name	0
Vial:	47	Acq. Method Set:	5%210400
Injection #:	1	Processing Method	133082
Injection Volume:	10.00 ul	Channel Name:	254.0nm
Run Time:	10.0 Minutes	Proc. Chnl. Descr.:	2998 PDA 254.0 nm (2998
Date Acquired:	12/23/2022 19:42:11 CST		
Date Processed:	1/4/2023 20:24:58 CST		

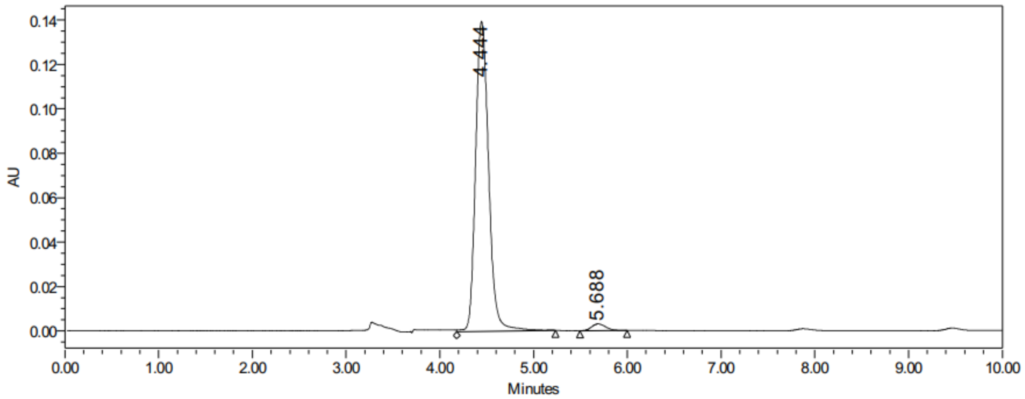

	RT	Area	% Area	Height
1	4.444	1350816	97.73	139536
2	5.688	31324	2.27	2981

Rac-**3c**

SAMPLE INFORMATION

Sample Name:	zql-13-268-RAC-IC-5%	Acquired By:	System
Sample Type:	Unknown	Sample Set Name:	0
Vial:	105	Acq. Method Set:	5%210400
Injection #:	1	Processing Method:	133361
Injection Volume:	10.00 ul	Channel Name:	254.0nm
Run Time:	8.0 Minutes	Proc. Chnl. Descr.:	2998 PDA 254.0 nm (2998
Date Acquired:	1/6/2023 8:42:06 PM CST		
Date Processed:	1/6/2023 9:34:15 PM CST		

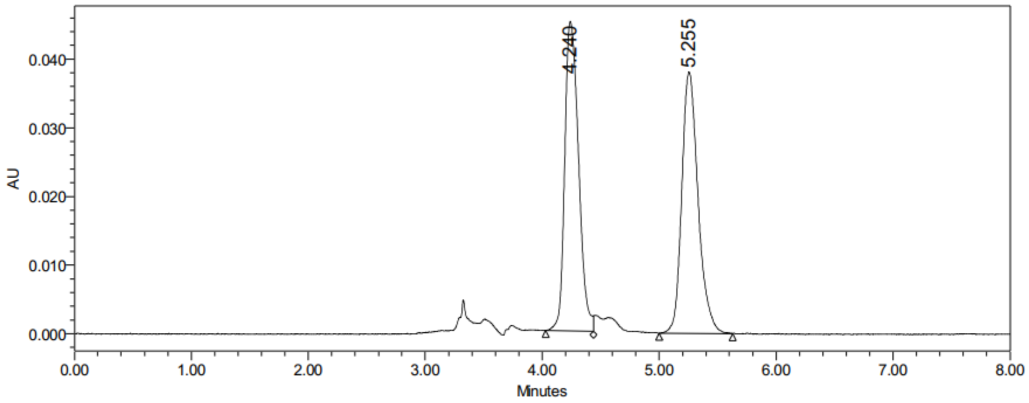

	RT	Area	% Area	Height
1	4.240	370126	50.09	45051
2	5.255	368854	49.91	38100

Asy-**3c**

SAMPLE INFORMATION

Sample Name:	zql-13-336-ASY-IC-5%	Acquired By:	System
Sample Type:	Unknown	Sample Set Name:	0
Vial:	14	Acq. Method Set:	5%210400
Injection #:	1	Processing Method:	133362
Injection Volume:	10.00 ul	Channel Name:	254.0nm
Run Time:	8.0 Minutes	Proc. Chnl. Descr.:	2998 PDA 254.0 nm (2998
Date Acquired:	1/6/2023 9:17:06 PM CST		
Date Processed:	1/6/2023 9:33:20 PM CST		

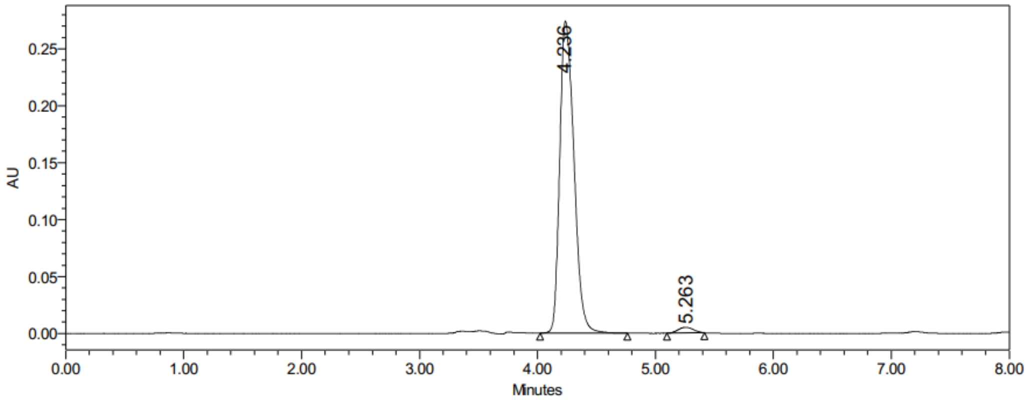

	RT	Area	% Area	Height
1	4.236	2296942	98.07	274025
2	5.263	45125	1.93	4925

Rac-**3d**

SAMPLE INFORMATION

Sample Name:	zql-13-278-RAC-IC-5%	Acquired By:	System
Sample Type:	Unknown	Sample Set Name:	0
Vial:	11	Acq. Method Set:	5%210400
Injection #:	1	Processing Method:	133371
Injection Volume:	10.00 ul	Channel Name:	254.0nm
Run Time:	8.0 Minutes	Proc. Chnl. Descr.:	2998 PDA 254.0 nm (2998
Date Acquired:	1/6/2023 8:51:03 PM CST		
Date Processed:	1/6/2023 9:35:52 PM CST		

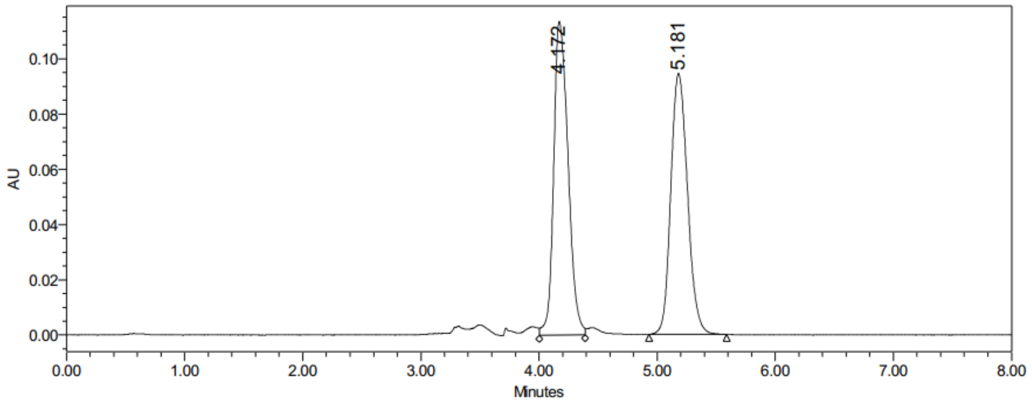

	RT	Area	% Area	Height
1	4.172	959328	50.85	113483
2	5.181	927435	49.15	94484

Asy-**3d**

SAMPLE INFORMATION

Sample Name:	zql-13-337-ASY-IC-5%	Acquired By:	System
Sample Type:	Unknown	Sample Set Name:	0
Vial:	15	Acq. Method Set:	5%210400
Injection #:	1	Processing Method:	133372
Injection Volume:	10.00 ul	Channel Name:	254.0nm
Run Time:	8.0 Minutes	Proc. Chnl. Descr.:	2998 PDA 254.0 nm (2998
Date Acquired:	1/6/2023 9:25:48 PM CST		
Date Processed:	1/6/2023 9:35:00 PM CST		

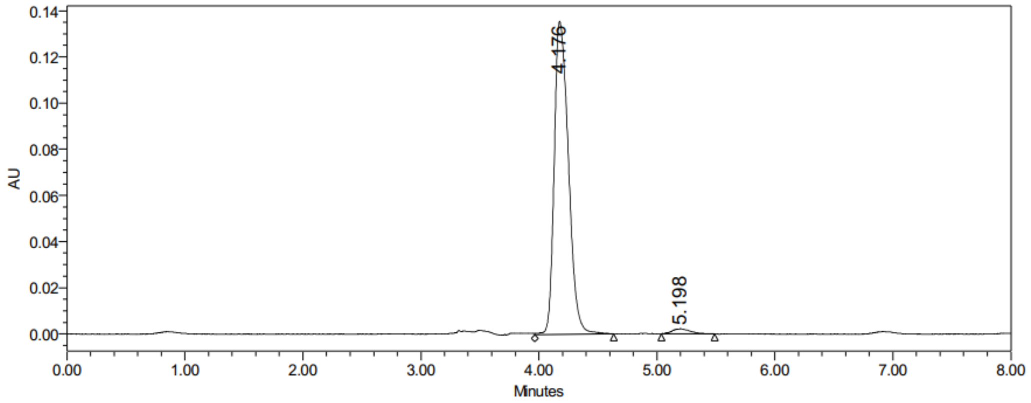

	RT	Area	% Area	Height
1	4.176	1134302	98.07	135676
2	5.198	22314	1.93	2121

Rac-**3e**

SAMPLE INFORMATION

Sample Name:	zql-13-282-RAC-IC-5%	Acquired By:	System
Sample Type:	Unknown	Sample Set Name:	0
Vial:	12	Acq. Method Set:	5%210400
Injection #:	1	Processing Method:	133381
Injection Volume:	10.00 ul	Channel Name:	254.0nm
Run Time:	8.0 Minutes	Proc. Chnl. Descr.:	2998 PDA 254.0 nm (2998
Date Acquired:	1/6/2023 8:59:45 PM CST		
Date Processed:	1/6/2023 9:55:39 PM CST		

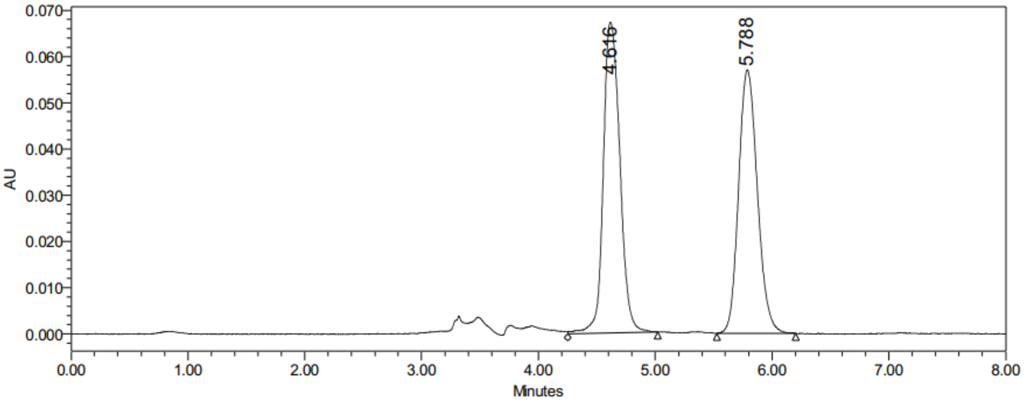

	RT	Area	% Area	Height
1	4.616	663161	51.01	67217
2	5.788	637010	48.99	56980

Asy-**3e**

SAMPLE INFORMATION

Sample Name:	zql-13-338-ASY-IC-5%	Acquired By:	System
Sample Type:	Unknown	Sample Set Name:	0
Vial:	16	Acq. Method Set:	5%210400
Injection #:	1	Processing Method:	133382
Injection Volume:	10.00 ul	Channel Name:	254.0nm
Run Time:	8.0 Minutes	Proc. Chnl. Descr.:	2998 PDA 254.0 nm (2998
Date Acquired:	1/6/2023 9:34:29 PM CST		
Date Processed:	1/6/2023 9:54:51 PM CST		

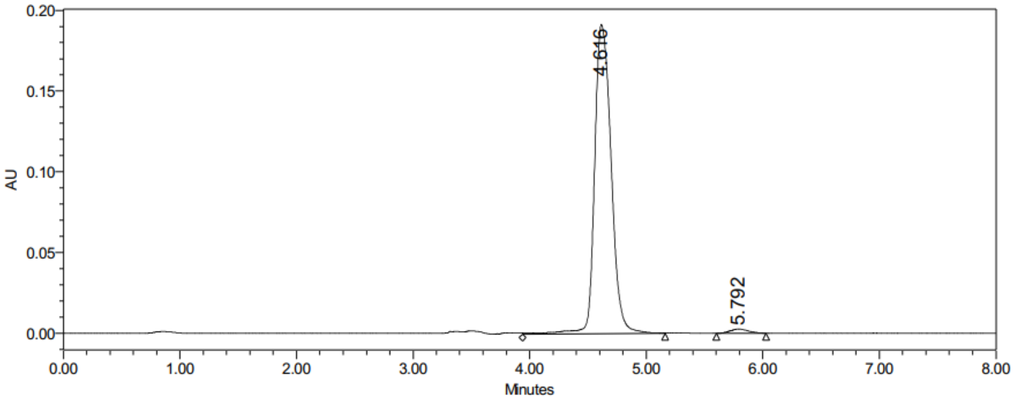

	RT	Area	% Area	Height
1	4.616	1890553	98.56	191355
2	5.792	27560	1.44	2461

Rac-**3f**

SAMPLE INFORMATION

Sample Name:	zql-12-197-RAC-IC-1%	Acquired By:	System
Sample Type:	Unknown	Sample Set Name	
Vial:	48	Acq. Method Set:	1%210400
Injection #:	1	Processing Method	122561
Injection Volume:	10.00 ul	Channel Name:	254.0nm
Run Time:	30.0 Minutes	Proc. Chnl. Descr.:	2998 PDA 254.0 nm (2998
Date Acquired:	7/22/2022 20:08:03 CST		
Date Processed:	1/7/2023 22:04:26 CST		

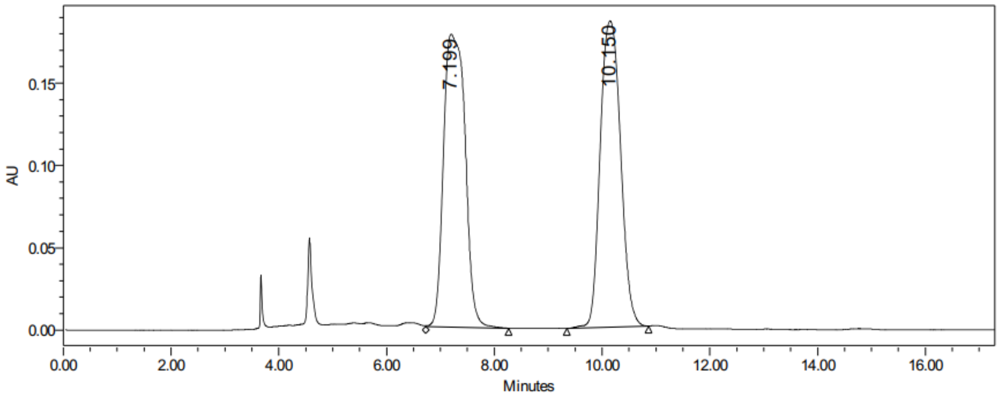

	RT	Area	% Area	Height
1	7.199	4940431	50.08	177848
2	10.150	4924243	49.92	185983

Asy-**3f**

SAMPLE INFORMATION

Sample Name:	zql-14-146-IC-1%	Acquired By:	System
Sample Type:	Unknown	Sample Set Name	0
Vial:	63	Acq. Method Set:	1%210400
Injection #:	1	Processing Method	3f
Injection Volume:	10.00 ul	Channel Name:	254.0nm
Run Time:	15.0 Minutes	Proc. Chnl. Descr.:	2998 PDA 254.0 nm (2998
Date Acquired:	2/25/2023 18:02:31 CST		
Date Processed:	10/6/2023 16:54:04 CST		

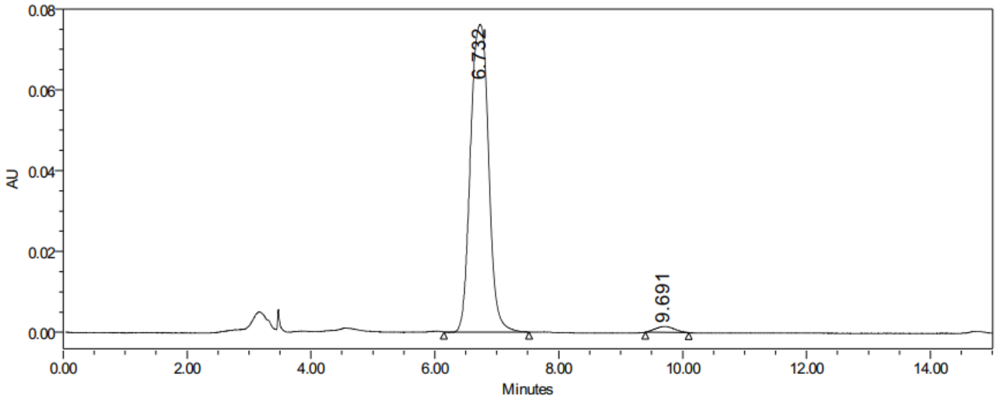

	RT	Area	% Area	Height
1	6.732	1551309	98.06	76140
2	9.691	30631	1.94	1430

Rac-**3g**

SAMPLE INFORMATION

Sample Name:	zql-13-326-RAC-IC-5%	Acquired By:	System
Sample Type:	Unknown	Sample Set Name	0
Vial:	2	Acq. Method Set:	5%210400
Injection #:	1	Processing Method	133321
Injection Volume:	10.00 ul	Channel Name:	254.0nm
Run Time:	8.0 Minutes	Proc. Chnl. Descr.:	2998 PDA 254.0 nm (2998
Date Acquired:	1/5/2023 20:38:32 CST		
Date Processed:	1/5/2023 21:41:00 CST		

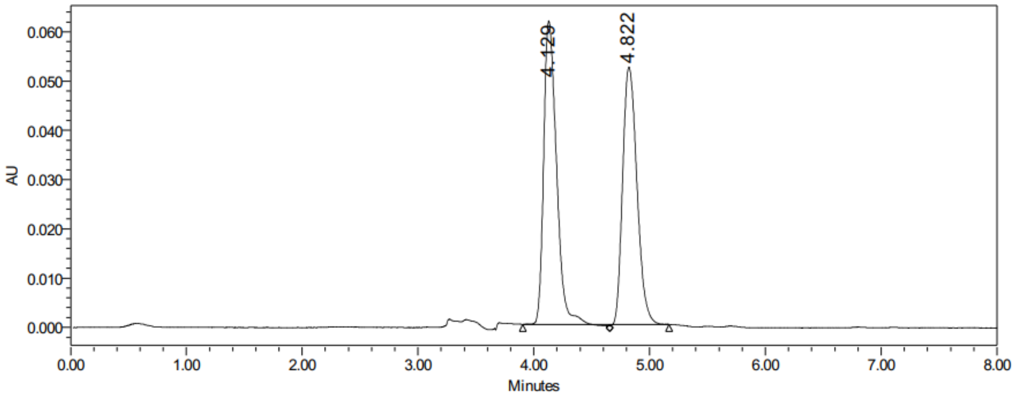

	RT	Area	% Area	Height
1	4.129	473993	51.34	61581
2	4.822	449261	48.66	52212

Asy-**3g**

SAMPLE INFORMATION

Sample Name:	zql-17-212-IC-5%	Acquired By:	System
Sample Type:	Unknown	Sample Set Name:	
Vial:	78	Acq. Method Set:	5%210400
Injection #:	1	Processing Method:	3g
Injection Volume:	10.00 ul	Channel Name:	254.0nm
Run Time:	10.0 Minutes	Proc. Chnl. Descr.:	2998 PDA 254.0 nm (2998
Date Acquired:	3/27/2024 10:25:30 PM CST		
Date Processed:	4/2/2024 9:38:49 PM CST		

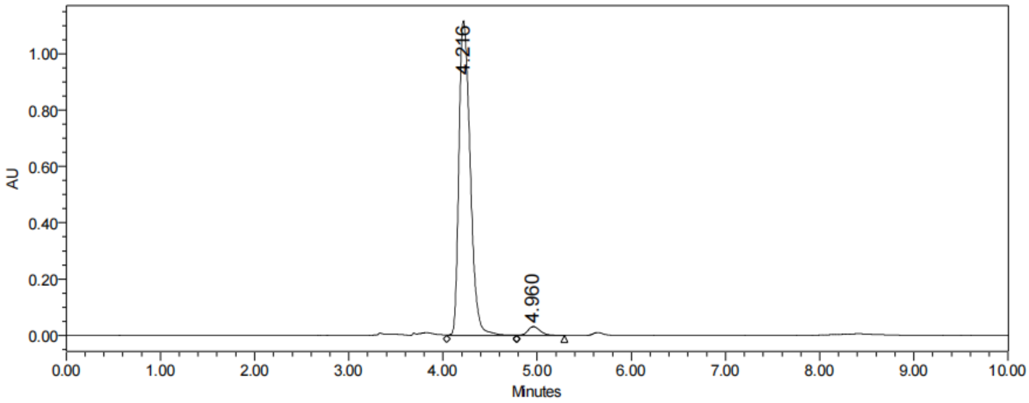

	RT	Area	% Area	Height
1	4.216	9299760	97.05	1116547
2	4.960	282998	2.95	31075

Rac-**3h**

SAMPLE INFORMATION

Sample Name:	zql-13-324-RAC-IC-5%	Acquired By:	System
Sample Type:	Unknown	Sample Set Name	
Vial:	60	Acq. Method Set:	5%210400
Injection #:	2	Processing Method	133301
Injection Volume:	10.00 ul	Channel Name:	254.0nm
Run Time:	8.0 Minutes	Proc. Chnl. Descr.:	2998 PDA 254.0 nm (2998
Date Acquired:	1/5/2023 20:15:13 CST		
Date Processed:	1/5/2023 21:36:45 CST		

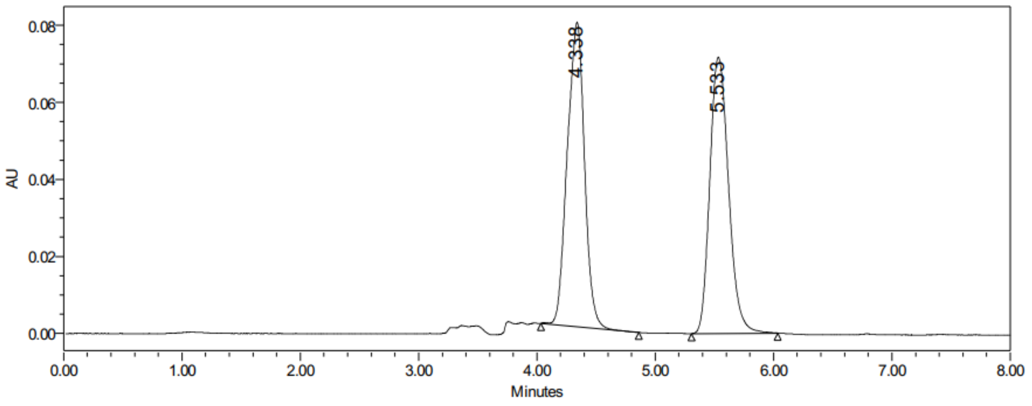

	RT	Area	% Area	Height
1	4.338	800919	50.39	79073
2	5.533	788532	49.61	71738

Asy-**3h**

SAMPLE INFORMATION

Sample Name:	zql-17-213-IC-5%	Acquired By:	System
Sample Type:	Unknown	Sample Set Name:	0
Vial:	58	Acq. Method Set:	5%210400
Injection #:	1	Processing Method:	3h
Injection Volume:	10.00 ul	Channel Name:	254.0nm
Run Time:	10.0 Minutes	Proc. Chnl. Descr.:	2998 PDA 254.0 nm (2998
Date Acquired:	3/28/2024 10:44:45 PM CST		
Date Processed:	4/2/2024 9:46:51 PM CST		

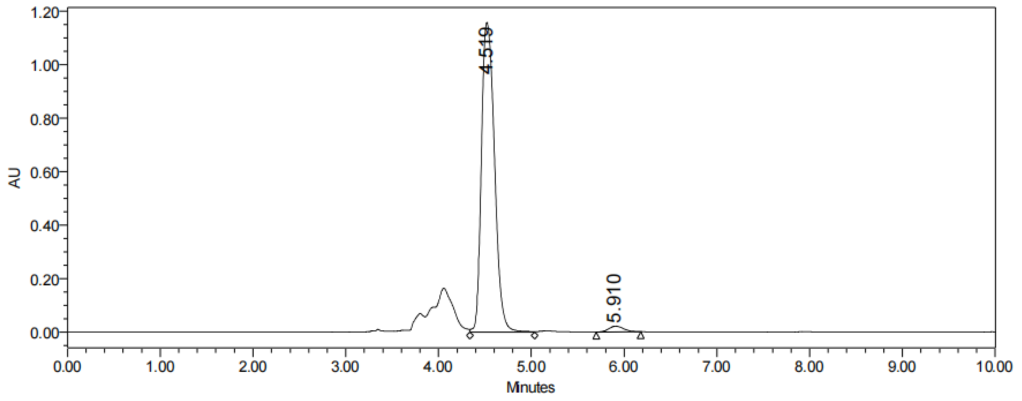

	RT	Area	% Area	Height
1	4.519	11374710	97.96	1156452
2	5.910	236579	2.04	21688

Rac-**3i**

SAMPLE INFORMATION

Sample Name:	zql-13-327-RAC-IC-5%	Acquired By:	System
Sample Type:	Unknown	Sample Set Name	0
Vial:	3	Acq. Method Set:	5%210400
Injection #:	1	Processing Method	133331
Injection Volume:	10.00 ul	Channel Name:	254.0nm
Run Time:	8.0 Minutes	Proc. Chnl. Descr.:	2998 PDA 254.0 nm (2998
Date Acquired:	1/5/2023 20:47:14 CST		
Date Processed:	1/5/2023 21:43:59 CST		

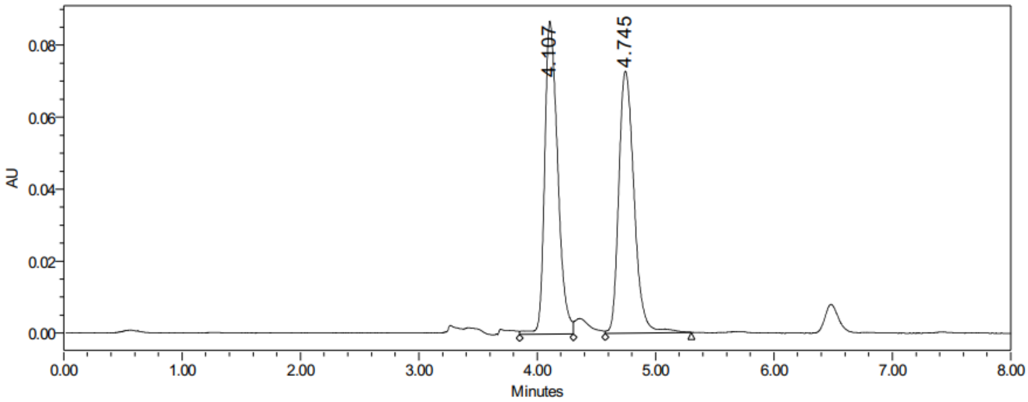

	RT	Area	% Area	Height
1	4.107	666171	50.41	86929
2	4.745	655246	49.59	72839

Asy-**3i**

SAMPLE INFORMATION

Sample Name:	zql-17-188-IC-5%	Acquired By:	System
Sample Type:	Unknown	Sample Set Name:	0
Vial:	84	Acq. Method Set:	5%210400
Injection #:	1	Processing Method:	3j
Injection Volume:	10.00 ul	Channel Name:	254.0nm
Run Time:	10.0 Minutes	Proc. Chnl. Descr.:	2998 PDA 254.0 nm (2998
Date Acquired:	3/20/2024 8:41:29 PM CST		
Date Processed:	4/2/2024 9:49:14 PM CST		

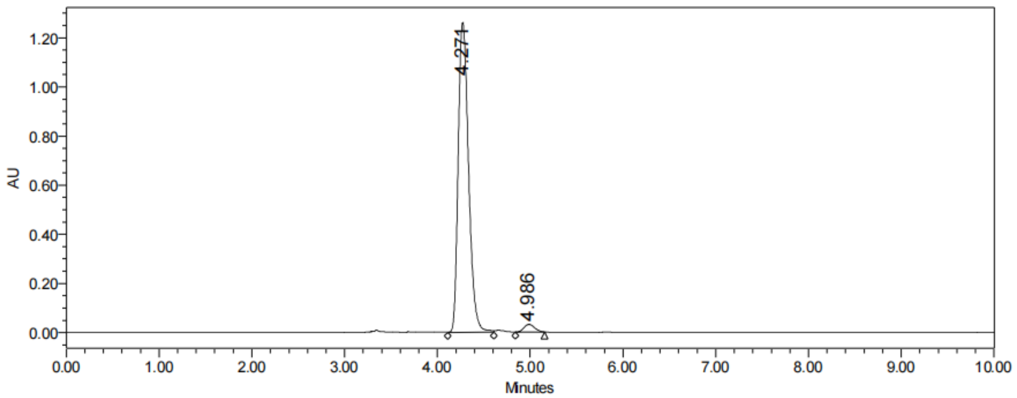

	RT	Area	% Area	Height
1	4.271	9752174	97.50	1260200
2	4.986	250483	2.50	31250

Rac-**3j**

SAMPLE INFORMATION

Sample Name:	zql-13-325-RAC-IC-5%	Acquired By:	System
Sample Type:	Unknown	Sample Set Name	0
Vial:	1	Acq. Method Set:	5%210400
Injection #:	1	Processing Method	133311
Injection Volume:	10.00 ul	Channel Name:	268.0nm
Run Time:	8.0 Minutes	Proc. Chnl. Descr.:	2998 PDA 268.0 nm (2998
Date Acquired:	1/5/2023 20:29:50 CST		
Date Processed:	1/5/2023 21:38:57 CST		

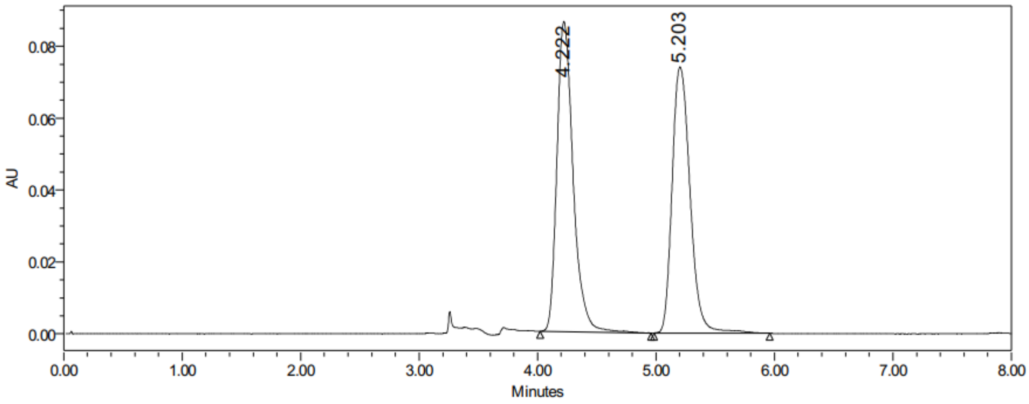

	RT	Area	% Area	Height
1	4.222	821629	51.26	86310
2	5.203	781371	48.74	74082

Asy-**3j**

SAMPLE INFORMATION

Sample Name:	zql-17-180-IC-5%	Acquired By:	System
Sample Type:	Unknown	Sample Set Name	0
Vial:	57	Acq. Method Set:	5%210400
Injection #:	1	Processing Method	3j
Injection Volume:	10.00 ul	Channel Name:	254.0nm
Run Time:	8.0 Minutes	Proc. Chnl. Descr.:	2998 PDA 254.0 nm (2998
Date Acquired:	4/2/2024 22:30:51 CST		
Date Processed:	4/2/2024 22:50:33 CST		

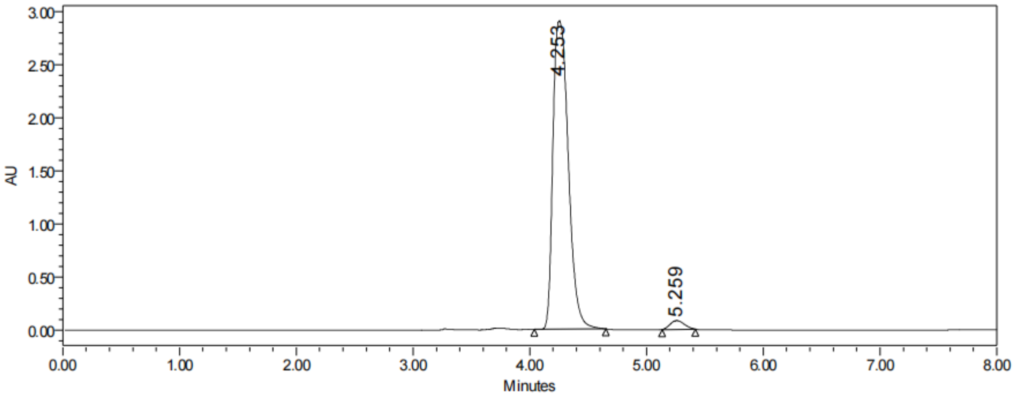

	RT	Area	% Area	Height
1	4.253	26547212	97.48	2903483
2	5.259	684958	2.52	81678

Rac-**3k**

SAMPLE INFORMATION

Sample Name:	zql-14-19-RAC-IC-5%	Acquired By:	System
Sample Type:	Unknown	Sample Set Name:	0
Vial:	33	Acq. Method Set:	5%210400
Injection #:	1	Processing Method:	14211
Injection Volume:	10.00 ul	Channel Name:	254.0nm
Run Time:	8.0 Minutes	Proc. Chnl. Descr.:	2998 PDA 254.0 nm (2998
Date Acquired:	1/12/2023 7:51:47 PM CST		
Date Processed:	1/12/2023 8:26:09 PM CST		

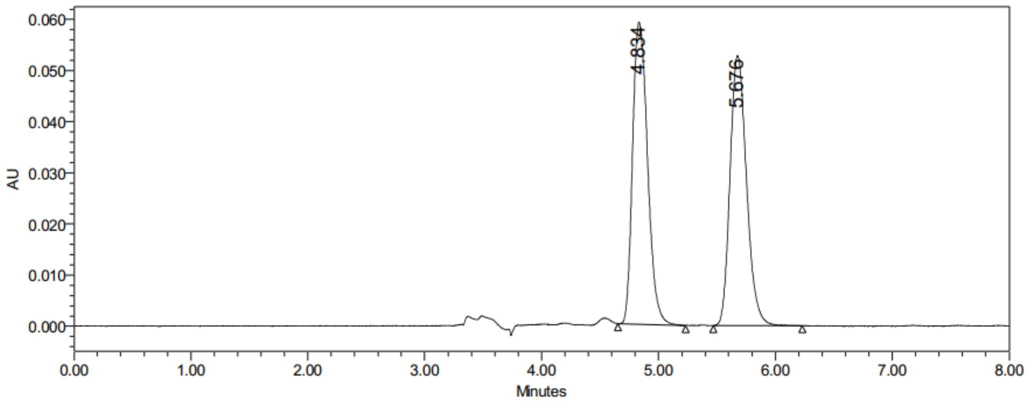

	RT	Area	% Area	Height
1	4.834	526127	50.36	59121
2	5.676	518531	49.64	52850

Asy-**3k**

SAMPLE INFORMATION

Sample Name:	zql-14-21-ASY-IC-5%	Acquired By:	System
Sample Type:	Unknown	Sample Set Name:	0
Vial:	49	Acq. Method Set:	5%210400
Injection #:	1	Processing Method:	14212
Injection Volume:	10.00 ul	Channel Name:	254.0nm
Run Time:	8.0 Minutes	Proc. Chnl. Descr.:	2998 PDA 254.0 nm (2998
Date Acquired:	1/12/2023 8:00:56 PM CST		
Date Processed:	1/12/2023 8:24:50 PM CST		

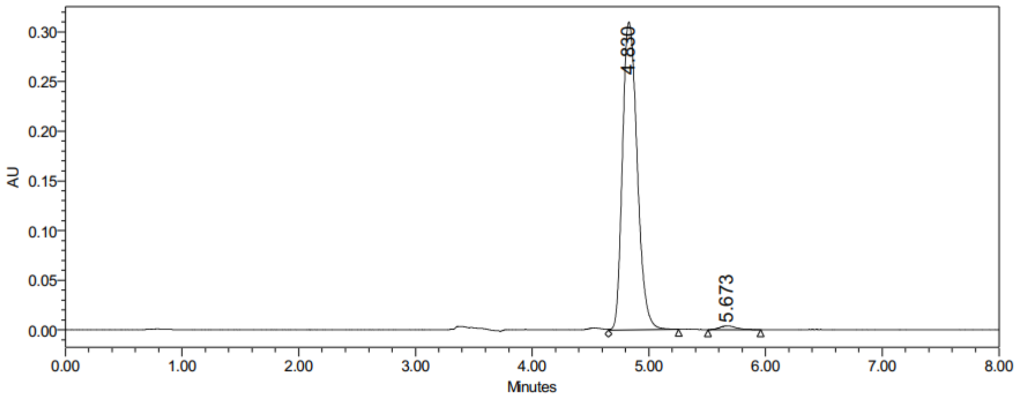

	RT	Area	% Area	Height
1	4.830	2718690	98.62	310078
2	5.673	37996	1.38	3891

Rac-**3l**

SAMPLE INFORMATION

Sample Name:	zql-14-58-rac-IE-1%	Acquired By:	System
Sample Type:	Unknown	Sample Set Name	0
Vial:	31	Acq. Method Set:	1%210400
Injection #:	1	Processing Method	14541
Injection Volume:	10.00 ul	Channel Name:	245.0nm
Run Time:	14.0 Minutes	Proc. Chnl. Descr.:	2998 PDA 245.0 nm (2998
Date Acquired:	4/15/2023 20:11:40 CST		
Date Processed:	4/15/2023 20:40:00 CST		

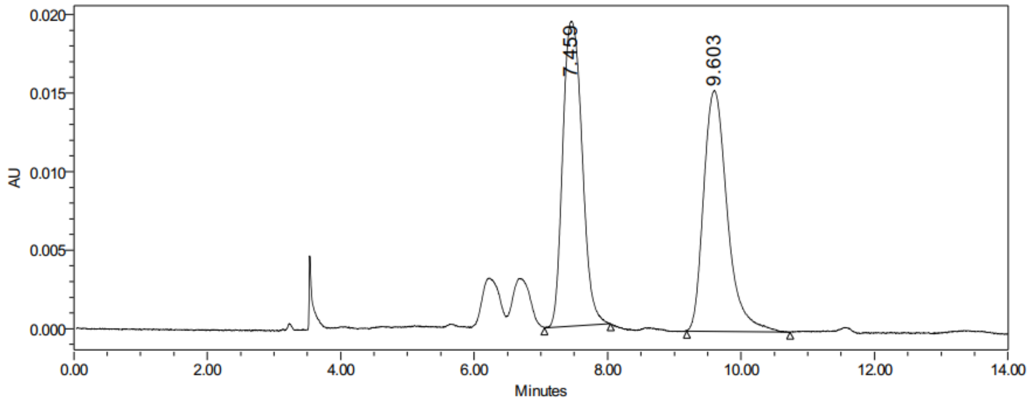

	RT	Area	% Area	Height
1	7.459	391659	50.90	19411
2	9.603	377768	49.10	15346

Asy-**3l**

SAMPLE INFORMATION

Sample Name:	zql-14-54-asy-IE-1%	Acquired By:	System
Sample Type:	Unknown	Sample Set Name	0
Vial:	70	Acq. Method Set:	1%210400
Injection #:	1	Processing Method	14542
Injection Volume:	10.00 ul	Channel Name:	254.0nm
Run Time:	14.0 Minutes	Proc. Chnl. Descr.:	2998 PDA 254.0 nm (2998
Date Acquired:	4/15/2023 20:26:41 CST		
Date Processed:	4/15/2023 20:41:28 CST		

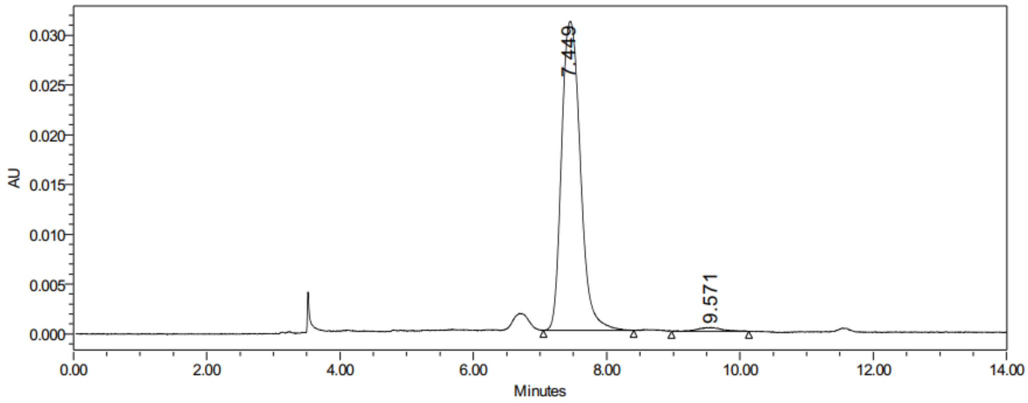

	RT	Area	% Area	Height
1	7.449	609990	98.54	31021
2	9.571	9029	1.46	383

Rac-**3m**

SAMPLE INFORMATION			
Sample Name:	zql-12-260-RAC-IC-1%	Acquired By:	System
Sample Type:	Unknown	Sample Set Name	0
Vial:	68	Acq. Method Set:	1%210400
Injection #:	1	Processing Method	141372
Injection Volume:	10.00 ul	Channel Name:	254.0nm
Run Time:	10.0 Minutes	Proc. Chnl. Descr.:	2998 PDA 254.0 nm (2998
Date Acquired:	2/23/2023 21:28:38 CST		
Date Processed:	2/23/2023 21:42:21 CST		

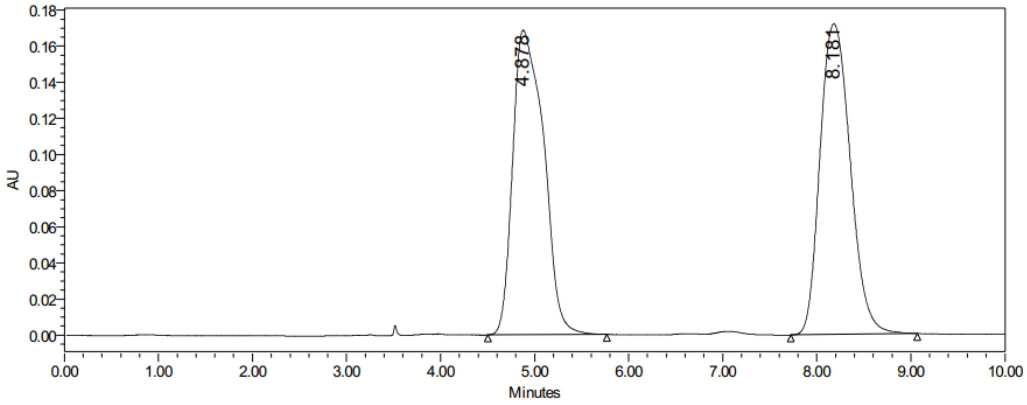

	RT	Area	% Area	Height
1	4.878	3851040	49.96	168762
2	8.181	3857663	50.04	172017

Asy-**3m**

SAMPLE INFORMATION			
Sample Name:	zql-14-137-IC-1%	Acquired By:	System
Sample Type:	Unknown	Sample Set Name	0
Vial:	1	Acq. Method Set:	1%210400
Injection #:	1	Processing Method	141371
Injection Volume:	10.00 ul	Channel Name:	254.0nm
Run Time:	10.0 Minutes	Proc. Chnl. Descr.:	2998 PDA 254.0 nm (2998
Date Acquired:	2/23/2023 20:21:45 CST		
Date Processed:	2/23/2023 21:28:14 CST		

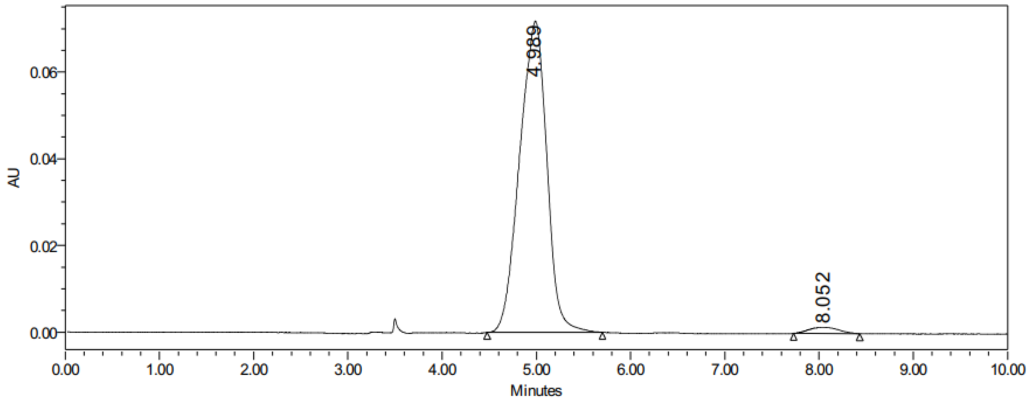

	RT	Area	% Area	Height
1	4.989	1438760	97.91	71825
2	8.052	30661	2.09	1401

Rac-**3n**

SAMPLE INFORMATION

Sample Name:	zql-14-144-rac-IF-5%	Acquired By:	System
Sample Type:	Unknown	Sample Set Name	
Vial:	44	Acq. Method Set:	5%210400
Injection #:	1	Processing Method	141381
Injection Volume:	10.00 ul	Channel Name:	254.0nm
Run Time:	15.0 Minutes	Proc. Chnl. Descr.:	2998 PDA 254.0 nm (2998
Date Acquired:	2/24/2023 21:11:14 CST		
Date Processed:	2/24/2023 22:03:20 CST		

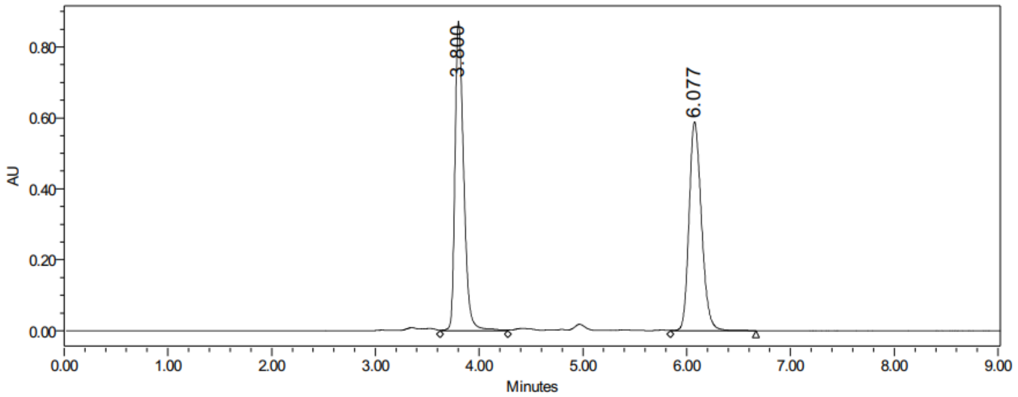

	RT	Area	% Area	Height
1	3.800	5076925	50.85	872009
2	6.077	4908167	49.15	588862

Asy-**3n**

SAMPLE INFORMATION

Sample Name:	zql-17-184-IF-5%	Acquired By:	System
Sample Type:	Unknown	Sample Set Name:	0
Vial:	26	Acq. Method Set:	5%210400
Injection #:	1	Processing Method:	3n
Injection Volume:	10.00 ul	Channel Name:	254.0nm
Run Time:	10.0 Minutes	Proc. Chnl. Descr.:	2998 PDA 254.0 nm (2998
Date Acquired:	3/20/2024 10:42:16 AM CST		
Date Processed:	4/2/2024 9:52:15 PM CST		

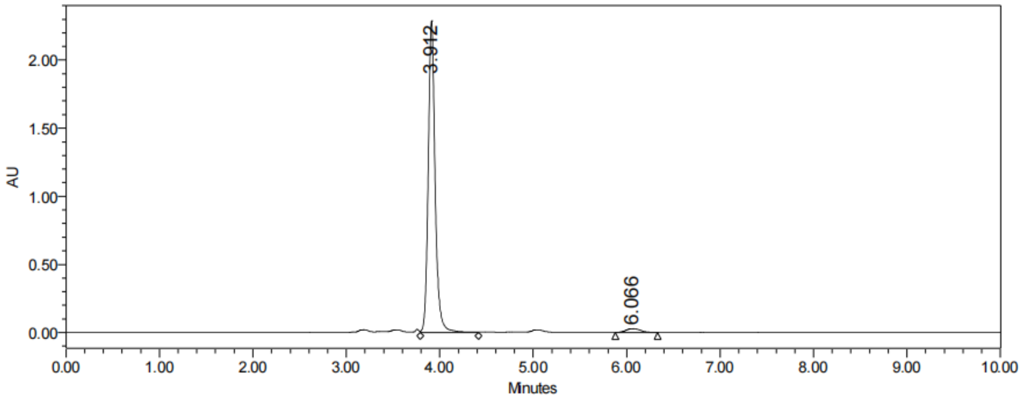

	RT	Area	% Area	Height
1	3.912	11756989	97.50	2286853
2	6.066	301096	2.50	28375

Rac-**3o**

SAMPLE INFORMATION

Sample Name:	zql-14-145-rac-IF-5%	Acquired By:	System
Sample Type:	Unknown	Sample Set Name	
Vial:	1	Acq. Method Set:	5%210400
Injection #:	1	Processing Method	131391
Injection Volume:	10.00 ul	Channel Name:	254.0nm
Run Time:	30.0 Minutes	Proc. Chnl. Descr.:	2998 PDA 254.0 nm (2998
Date Acquired:	2/24/2023 21:00:51 CST		
Date Processed:	2/24/2023 22:05:56 CST		

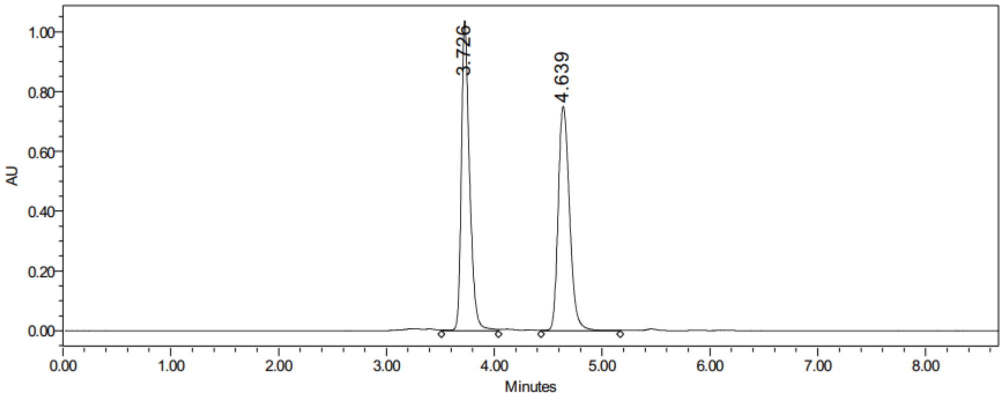

	RT	Area	% Area	Height
1	3.726	5534983	50.18	1035929
2	4.639	5494564	49.82	750931

Asy-**3o**

SAMPLE INFORMATION

Sample Name:	zql-14-139-IF-5%	Acquired By:	System
Sample Type:	Unknown	Sample Set Name	0
Vial:	2	Acq. Method Set:	5%210400
Injection #:	1	Processing Method	131392
Injection Volume:	10.00 ul	Channel Name:	254.0nm
Run Time:	8.5 Minutes	Proc. Chnl. Descr.:	2998 PDA 254.0 nm (2998
Date Acquired:	2/24/2023 21:42:40 CST		
Date Processed:	2/24/2023 22:06:39 CST		

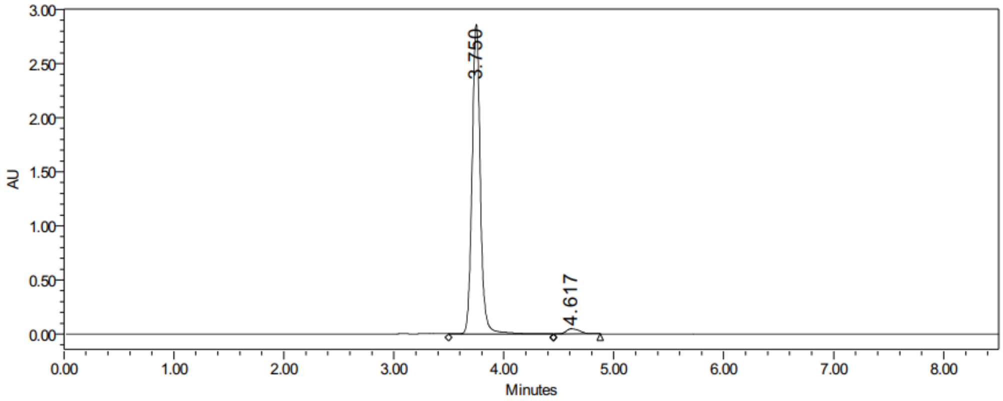

	RT	Area	% Area	Height
1	3.750	14881475	97.37	2859790
2	4.617	401433	2.63	47841

Rac-**3p**

SAMPLE INFORMATION

Sample Name:	zql-14-123-IF-5%	Acquired By:	System
Sample Type:	Unknown	Sample Set Name:	0
Vial:	75	Acq. Method Set:	5%210400
Injection #:	1	Processing Method:	141271
Injection Volume:	10.00 ul	Channel Name:	254.0nm
Run Time:	7.0 Minutes	Proc. Chnl. Descr.:	2998 PDA 254.0 nm (2998
Date Acquired:	2/21/2023 10:11:06 PM CST		
Date Processed:	2/23/2023 8:24:15 PM CST		

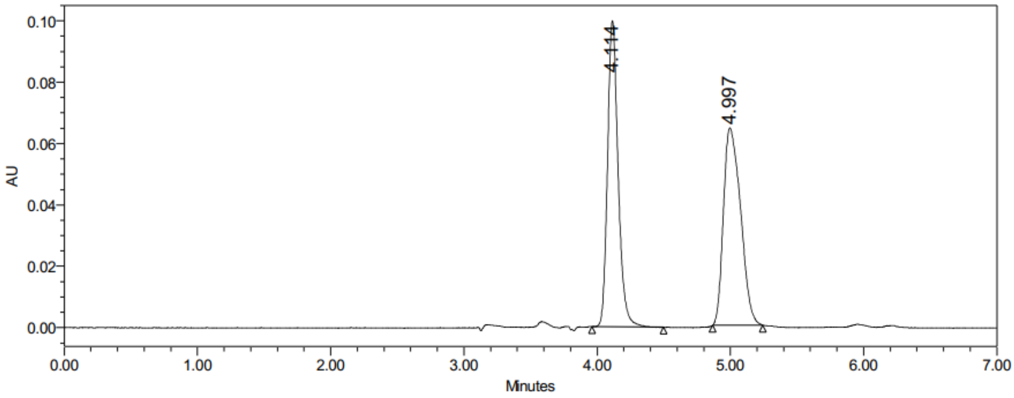

	RT	Area	% Area	Height
1	4.114	552160	48.98	99764
2	4.997	575184	51.02	64321

Asy-**3p**

SAMPLE INFORMATION

Sample Name:	zql-17-183-IF-5%	Acquired By:	System
Sample Type:	Unknown	Sample Set Name:	0
Vial:	25	Acq. Method Set:	5%210400
Injection #:	1	Processing Method:	3p
Injection Volume:	10.00 ul	Channel Name:	254.0nm
Run Time:	10.0 Minutes	Proc. Chnl. Descr.:	2998 PDA 254.0 nm (2998
Date Acquired:	3/20/2024 10:31:35 AM CST		
Date Processed:	4/2/2024 10:02:00 PM CST		

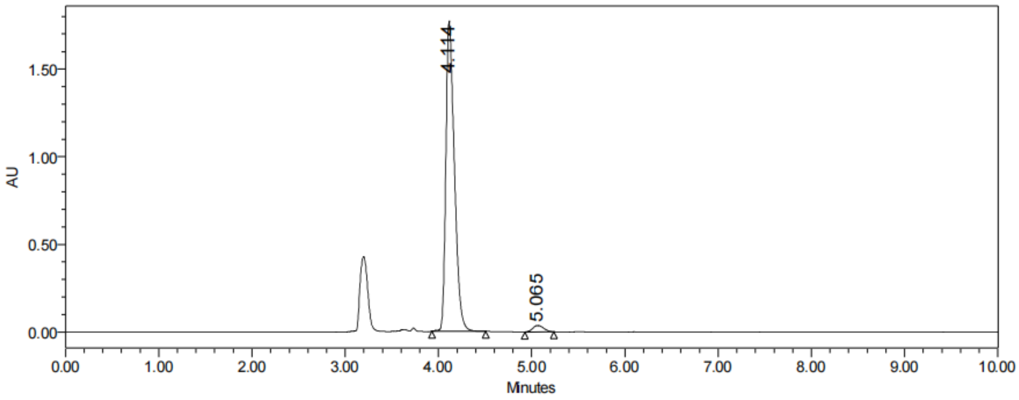

	RT	Area	% Area	Height
1	4.114	11254988	97.42	1768235
2	5.065	298204	2.58	36867

Rac-**3q**

SAMPLE INFORMATION

Sample Name:	zql-14-143-rac-IF-5%	Acquired By:	System
Sample Type:	Unknown	Sample Set Name	
Vial:	105	Acq. Method Set:	5%210400
Injection #:	1	Processing Method	141421
Injection Volume:	10.00 ul	Channel Name:	254.0nm
Run Time:	30.0 Minutes	Proc. Chnl. Descr.:	2998 PDA 254.0 nm (2998
Date Acquired:	2/24/2023 20:49:14 CST		
Date Processed:	2/24/2023 22:09:47 CST		

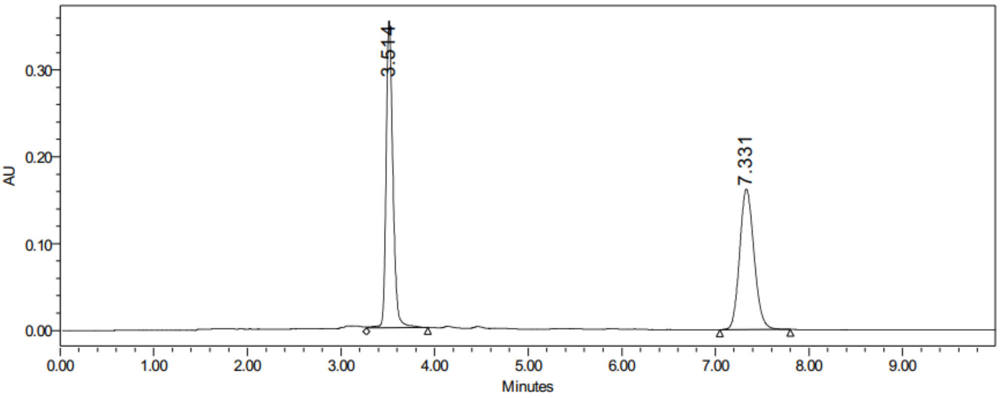

	RT	Area	% Area	Height
1	3.514	1778519	50.76	353271
2	7.331	1725513	49.24	161818

Asy-**3q**

SAMPLE INFORMATION

Sample Name:	zql-14-142-IF-5%	Acquired By:	System
Sample Type:	Unknown	Sample Set Name	
Vial:	4	Acq. Method Set:	5%210400
Injection #:	1	Processing Method	141422
Injection Volume:	2.00 ul	Channel Name:	254.0nm
Run Time:	10.0 Minutes	Proc. Chnl. Descr.:	2998 PDA 254.0 nm (2998
Date Acquired:	2/24/2023 22:13:37 CST		
Date Processed:	2/24/2023 22:27:51 CST		

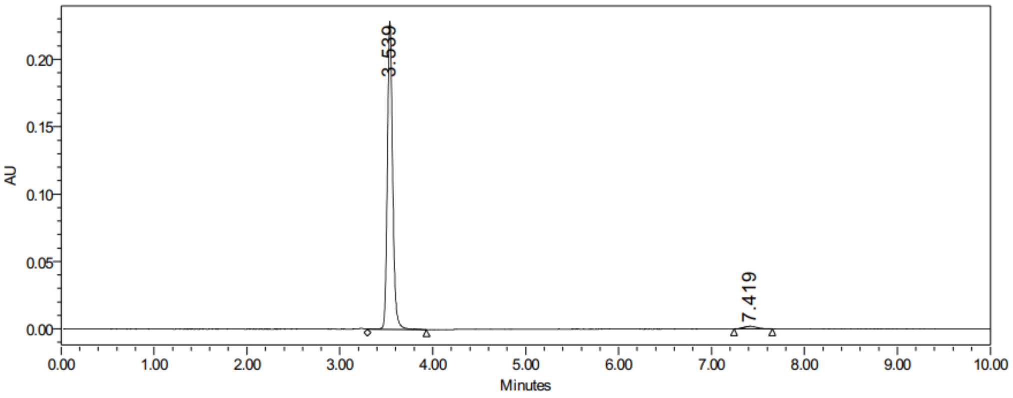

	RT	Area	% Area	Height
1	3.539	907432	97.93	228653
2	7.419	19161	2.07	2025

Rac-**3r**

SAMPLE INFORMATION

Sample Name:	zql-12-296-rac-IF-5%	Acquired By:	System
Sample Type:	Unknown	Sample Set Name	
Vial:	61	Acq. Method Set:	5%210400
Injection #:	1	Processing Method	141411
Injection Volume:	10.00 ul	Channel Name:	254.0nm
Run Time:	15.0 Minutes	Proc. Chnl. Descr.:	2998 PDA 254.0 nm (2998
Date Acquired:	2/24/2023 21:21:36 CST		
Date Processed:	2/24/2023 22:07:58 CST		

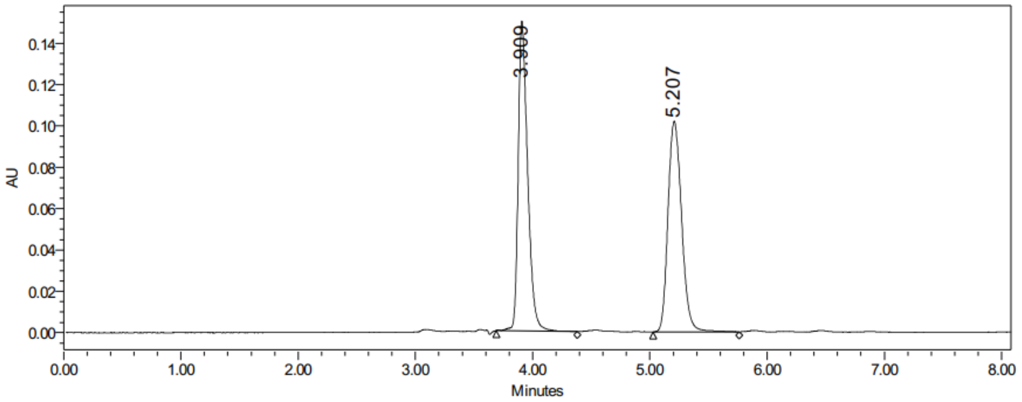

	RT	Area	% Area	Height
1	3.909	830589	50.32	149705
2	5.207	819981	49.68	101913

Asy-**3r**

SAMPLE INFORMATION

Sample Name:	zql-14-141-IF-5%	Acquired By:	System
Sample Type:	Unknown	Sample Set Name	0
Vial:	3	Acq. Method Set:	5%210400
Injection #:	1	Processing Method	141412
Injection Volume:	10.00 ul	Channel Name:	254.0nm
Run Time:	8.0 Minutes	Proc. Chnl. Descr.:	2998 PDA 254.0 nm (2998
Date Acquired:	2/24/2023 21:51:51 CST		
Date Processed:	2/24/2023 22:08:48 CST		

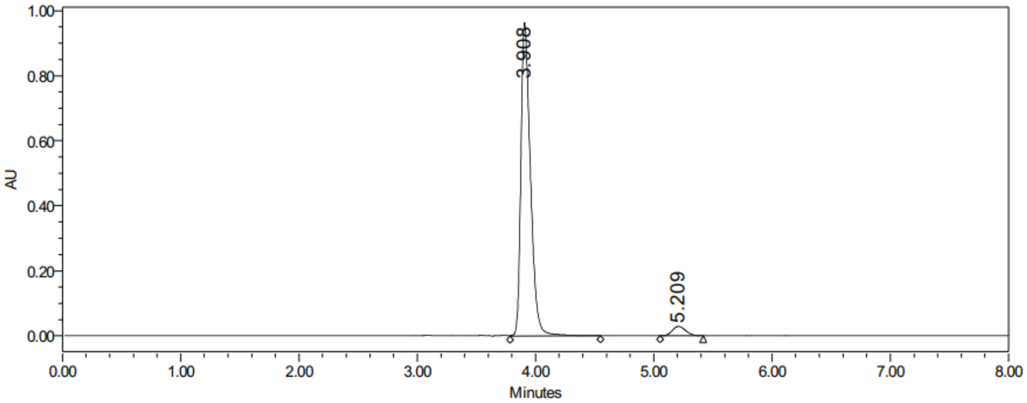

	RT	Area	% Area	Height
1	3.908	5357031	95.97	963211
2	5.209	224710	4.03	28599

Rac-**3s**

SAMPLE INFORMATION

Sample Name:	zql-12-87-RAC-IBN-5-5%	Acquired By:	System
Sample Type:	Unknown	Sample Set Name	0
Vial:	106	Acq. Method Set:	5%210400
Injection #:	1	Processing Method	132551
Injection Volume:	10.00 ul	Channel Name:	254.0nm
Run Time:	10.0 Minutes	Proc. Chnl. Descr.:	2998 PDA 254.0 nm (2998
Date Acquired:	4/15/2023 18:37:29 CST		
Date Processed:	4/15/2023 19:01:11 CST		

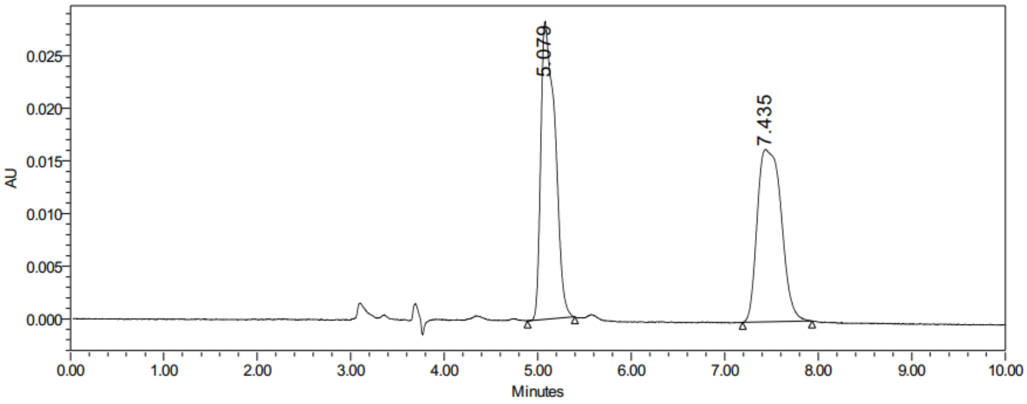

	RT	Area	% Area	Height
1	5.079	298256	50.35	28314
2	7.435	294128	49.65	16383

Asy-**3s**

SAMPLE INFORMATION

Sample Name:	zql-13-255-asy-IBN-5-5%	Acquired By:	System
Sample Type:	Unknown	Sample Set Name	0
Vial:	107	Acq. Method Set:	5%210400
Injection #:	1	Processing Method	132552
Injection Volume:	10.00 ul	Channel Name:	254.0nm
Run Time:	10.0 Minutes	Proc. Chnl. Descr.:	2998 PDA 254.0 nm (2998
Date Acquired:	4/15/2023 18:48:11 CST		
Date Processed:	4/15/2023 19:00:02 CST		

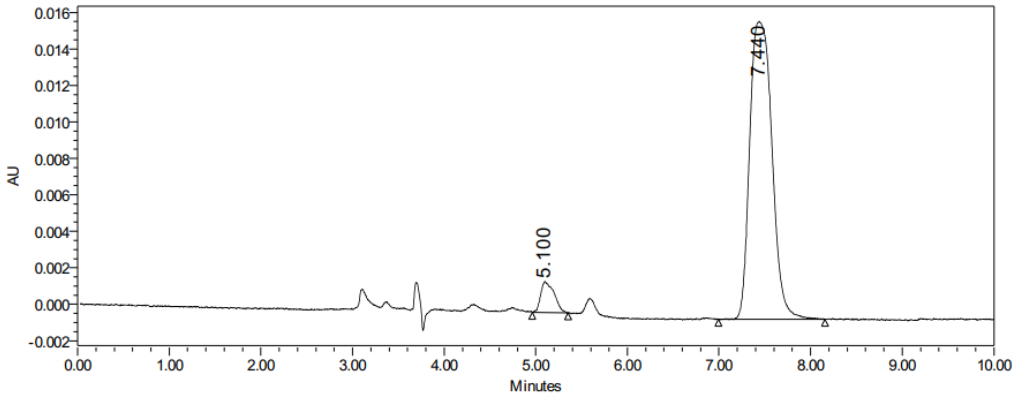

	RT	Area	% Area	Height
1	5.100	17178	5.88	1691
2	7.440	274897	94.12	16319

Rac-**3t**

SAMPLE INFORMATION

Sample Name:	zql-14-41-RAC-IF-1%	Acquired By:	System
Sample Type:	Unknown	Sample Set Name:	0
Vial:	7	Acq. Method Set:	1%210400
Injection #:	1	Processing Method:	14421
Injection Volume:	10.00 ul	Channel Name:	254.0nm
Run Time:	20.0 Minutes	Proc. Chnl. Descr.:	2998 PDA 254.0 nm (2998
Date Acquired:	1/31/2023 4:25:17 PM CST		
Date Processed:	1/31/2023 4:53:05 PM CST		

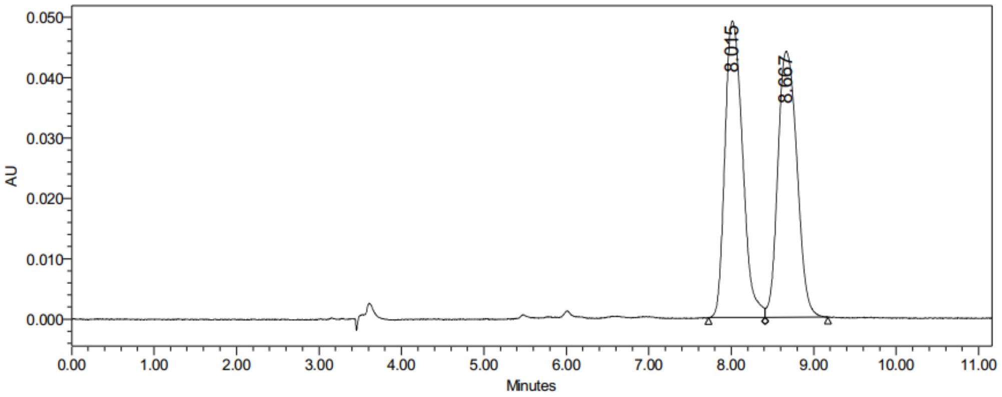

	RT	Area	% Area	Height
1	8.015	729520	50.74	49093
2	8.667	708233	49.26	44038

Asy-**3t**

SAMPLE INFORMATION

Sample Name:	zql-17-193-IF-1%	Acquired By:	System
Sample Type:	Unknown	Sample Set Name	0
Vial:	32	Acq. Method Set:	1%210400
Injection #:	1	Processing Method	3t
Injection Volume:	10.00 ul	Channel Name:	254.0nm
Run Time:	12.0 Minutes	Proc. Chnl. Descr.:	2998 PDA 254.0 nm (2998
Date Acquired:	3/21/2024 19:32:08 CST		
Date Processed:	4/2/2024 22:12:15 CST		

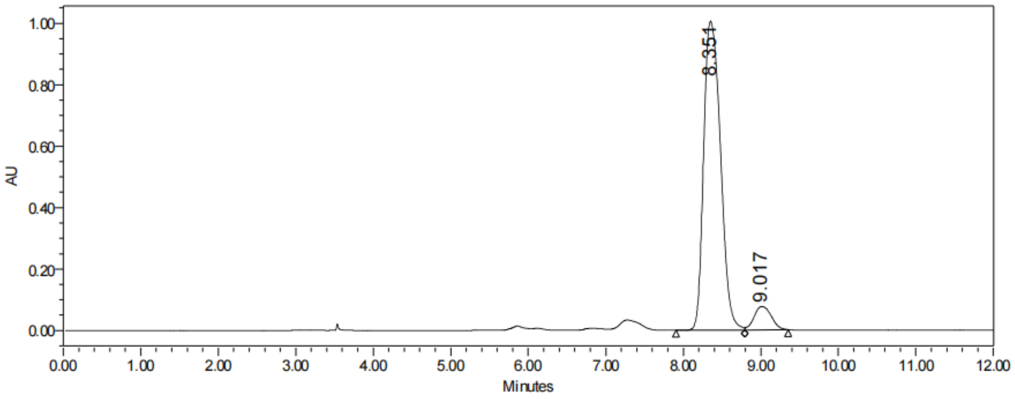

	RT	Area	% Area	Height
1	8.351	15230346	92.89	1004823
2	9.017	1165718	7.11	76055

Rac-**3u**

SAMPLE INFORMATION

Sample Name:	zql-13-344-rac-IC-5%	Acquired By:	System
Sample Type:	Unknown	Sample Set Name	
Vial:	61	Acq. Method Set:	5%210400
Injection #:	1	Processing Method	133451
Injection Volume:	10.00 ul	Channel Name:	254.0nm
Run Time:	15.0 Minutes	Proc. Chnl. Descr.:	2998 PDA 254.0 nm (2998
Date Acquired:	1/7/2023 20:53:00 CST		
Date Processed:	1/7/2023 21:34:54 CST		

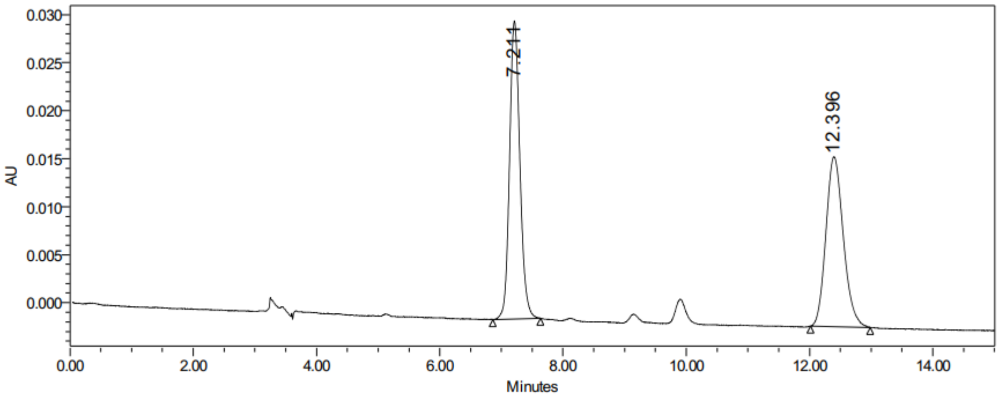

	RT	Area	% Area	Height
1	7.211	361900	51.01	31042
2	12.396	347590	48.99	17723

Asy-**3u**

SAMPLE INFORMATION

Sample Name:	zql-13-345-asy-IC-5%	Acquired By:	System
Sample Type:	Unknown	Sample Set Name	0
Vial:	92	Acq. Method Set:	5%210400
Injection #:	1	Processing Method	133452
Injection Volume:	10.00 ul	Channel Name:	254.0nm
Run Time:	15.0 Minutes	Proc. Chnl. Descr.:	2998 PDA 254.0 nm (2998
Date Acquired:	1/7/2023 21:17:43 CST		
Date Processed:	1/7/2023 21:33:55 CST		

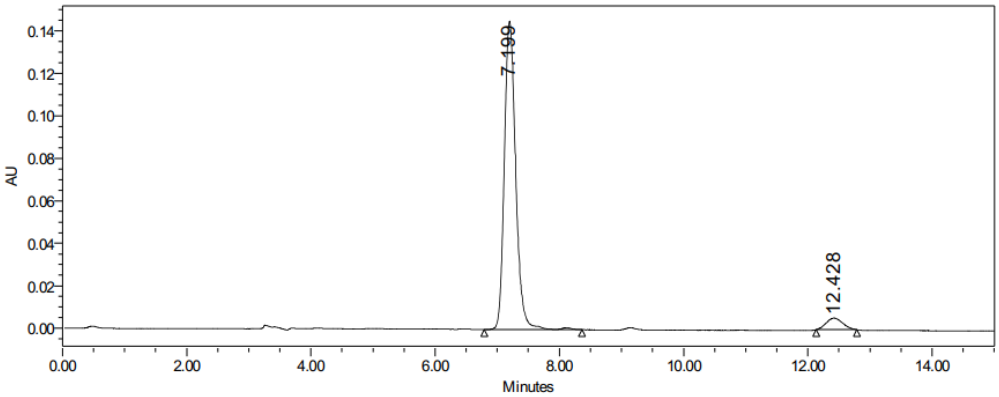

	RT	Area	% Area	Height
1	7.199	1845067	94.82	145036
2	12.428	100769	5.18	5447

Rac-**3v**

SAMPLE INFORMATION

Sample Name:	zql-14-9-RAC-IC-5%	Acquired By:	System
Sample Type:	Unknown	Sample Set Name:	
Vial:	54	Acq. Method Set:	5%210400
Injection #:	1	Processing Method:	1413
Injection Volume:	10.00 ul	Channel Name:	254.0nm
Run Time:	20.0 Minutes	Proc. Chnl. Descr.:	2998 PDA 254.0 nm (2998
Date Acquired:	1/9/2023 10:00:25 PM CST		
Date Processed:	1/10/2023 7:33:34 PM CST		

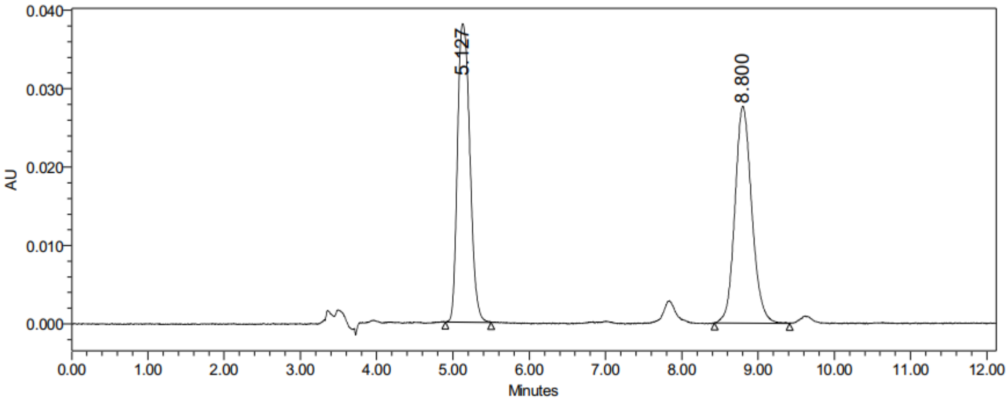

	RT	Area	% Area	Height
1	5.127	429485	50.18	38064
2	8.800	426363	49.82	27672

Asy-**3v**

SAMPLE INFORMATION

Sample Name:	zql-14-13-ASY-IC-5%	Acquired By:	System
Sample Type:	Unknown	Sample Set Name	0
Vial:	33	Acq. Method Set:	5%210400
Injection #:	1	Processing Method	1413
Injection Volume:	10.00 ul	Channel Name:	254.0nm
Run Time:	12.0 Minutes	Proc. Chnl. Descr.:	2998 PDA 254.0 nm (2998
Date Acquired:	1/10/2023 19:05:17 CST		
Date Processed:	1/10/2023 19:22:29 CST		

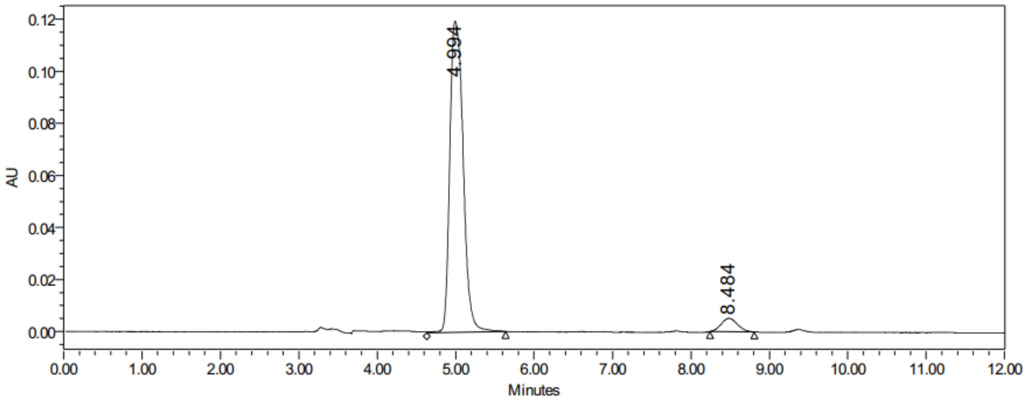

	RT	Area	% Area	Height
1	4.994	1412536	94.98	119544
2	8.484	74722	5.02	5170

Rac-**3w**

SAMPLE INFORMATION

Sample Name:	zql-13-340-RAC-IC-15%	Acquired By:	System
Sample Type:	Unknown	Sample Set Name:	0
Vial:	38	Acq. Method Set:	15%210400
Injection #:	1	Processing Method:	133411
Injection Volume:	10.00 ul	Channel Name:	254.0nm
Run Time:	12.0 Minutes	Proc. Chnl. Descr.:	2998 PDA 254.0 nm (2998
Date Acquired:	1/6/2023 11:14:36 PM CST		
Date Processed:	1/6/2023 11:38:21 PM CST		

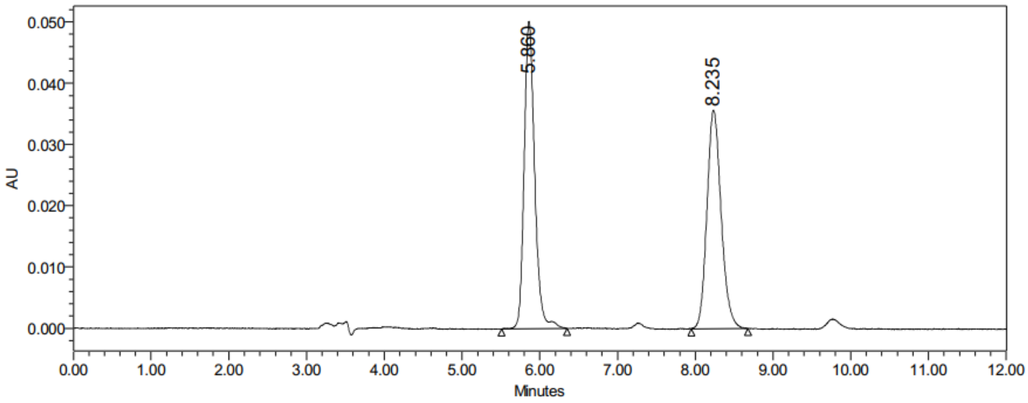

	RT	Area	% Area	Height
1	5.860	472208	50.96	50151
2	8.235	454417	49.04	35719

Asy-**3w**

SAMPLE INFORMATION

Sample Name:	zql-17-194-IC-5%	Acquired By:	System
Sample Type:	Unknown	Sample Set Name:	0
Vial:	90	Acq. Method Set:	15%210400
Injection #:	1	Processing Method:	3w
Injection Volume:	10.00 ul	Channel Name:	254.0nm
Run Time:	12.0 Minutes	Proc. Chnl. Descr.:	2998 PDA 254.0 nm (2998
Date Acquired:	3/21/2024 7:43:22 PM CST		
Date Processed:	4/2/2024 10:03:57 PM CST		

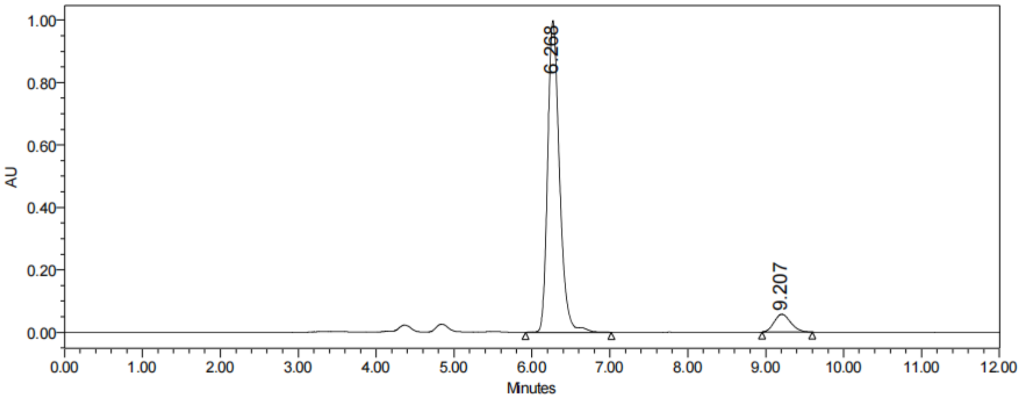

	RT	Area	% Area	Height
1	6.268	10562050	92.51	996829
2	9.207	854680	7.49	57781

Rac-**3x**

SAMPLE INFORMATION

Sample Name:	zql-14-7-RAC-IC-5%	Acquired By:	System
Sample Type:	Unknown	Sample Set Name:	
Vial:	52	Acq. Method Set:	5%210400
Injection #:	1	Processing Method:	1411
Injection Volume:	10.00 ul	Channel Name:	254.0nm
Run Time:	14.0 Minutes	Proc. Chnl. Descr.:	2998 PDA 254.0 nm (2998
Date Acquired:	1/9/2023 10:13:32 PM CST		
Date Processed:	1/10/2023 7:31:44 PM CST		

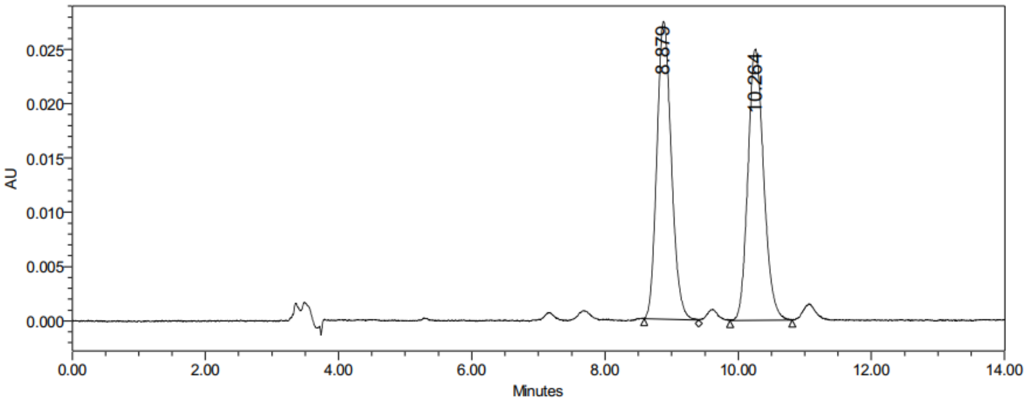

	RT	Area	% Area	Height
1	8.879	413299	50.06	27413
2	10.264	412365	49.94	25005

Asy-**3x**

SAMPLE INFORMATION

Sample Name:	zql-17-198-IC-5%	Acquired By:	System
Sample Type:	Unknown	Sample Set Name	0
Vial:	72	Acq. Method Set:	5%210400
Injection #:	1	Processing Method	3x
Injection Volume:	10.00 ul	Channel Name:	254.0nm
Run Time:	12.0 Minutes	Proc. Chnl. Descr.:	2998 PDA 254.0 nm (2998
Date Acquired:	3/23/2024 15:10:18 CST		
Date Processed:	4/2/2024 22:10:32 CST		

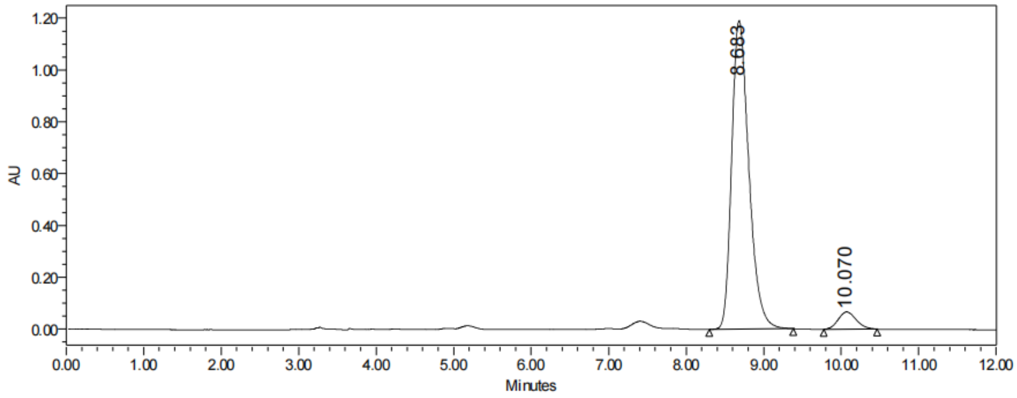

	RT	Area	% Area	Height
1	8.683	18215821	94.40	1189342
2	10.070	1081318	5.60	66627

Rac-**3y**

SAMPLE INFORMATION

Sample Name:	zql-14-8-RAC-IC-5%	Acquired By:	System
Sample Type:	Unknown	Sample Set Name:	
Vial:	53	Acq. Method Set:	5%210400
Injection #:	1	Processing Method:	1412
Injection Volume:	10.00 ul	Channel Name:	254.0nm
Run Time:	20.0 Minutes	Proc. Chnl. Descr.:	2998 PDA 254.0 nm (2998
Date Acquired:	1/9/2023 9:45:29 PM CST		
Date Processed:	1/10/2023 7:32:43 PM CST		

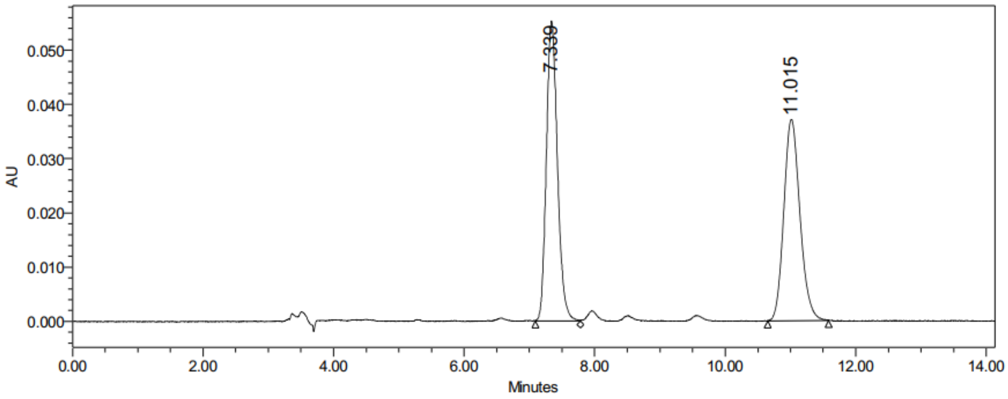

	RT	Area	% Area	Height
1	7.339	651163	50.67	55281
2	11.015	633936	49.33	37110

Asy-**3y**

SAMPLE INFORMATION

Sample Name:	zql-14-12-ASY-IC-5%	Acquired By:	System
Sample Type:	Unknown	Sample Set Name	0
Vial:	32	Acq. Method Set:	5%210400
Injection #:	1	Processing Method	1412
Injection Volume:	10.00 ul	Channel Name:	254.0nm
Run Time:	14.0 Minutes	Proc. Chnl. Descr.:	2998 PDA 254.0 nm (2998
Date Acquired:	1/10/2023 18:50:35 CST		
Date Processed:	1/10/2023 19:20:52 CST		

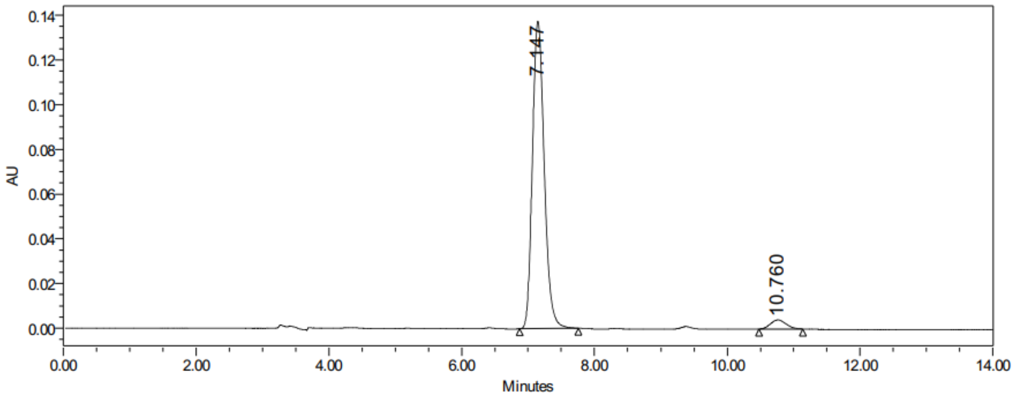

	RT	Area	% Area	Height
1	7.147	1702075	96.19	137457
2	10.760	67376	3.81	4130

Rac-**3z**

SAMPLE INFORMATION

Sample Name:	zql-17-127-RAC-IC-20%	Acquired By:	System
Sample Type:	Unknown	Sample Set Name:	0
Vial:	48	Acq. Method Set:	20%210400
Injection #:	1	Processing Method:	17101
Injection Volume:	10.00 ul	Channel Name:	254.0nm
Run Time:	14.0 Minutes	Proc. Chnl. Descr.:	2998 PDA 254.0 nm (2998
Date Acquired:	3/13/2024 8:50:35 PM CST		
Date Processed:	3/29/2024 8:14:29 PM CST		

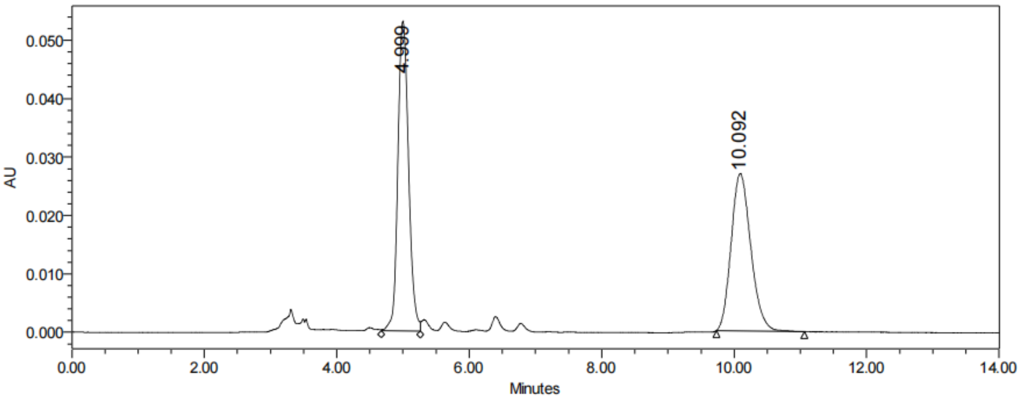

	RT	Area	% Area	Height
1	4.999	593921	51.64	53007
2	10.092	556172	48.36	26904

Asy-**3z**

SAMPLE INFORMATION

Sample Name:	zql-17-216-IC-20%	Acquired By:	System
Sample Type:	Unknown	Sample Set Name	0
Vial:	47	Acq. Method Set:	20%210400
Injection #:	1	Processing Method	171283
Injection Volume:	10.00 ul	Channel Name:	254.0nm
Run Time:	15.0 Minutes	Proc. Chnl. Descr.:	2998 PDA 254.0 nm (2998
Date Acquired:	4/2/2024 19:51:03 CST		
Date Processed:	4/2/2024 22:06:08 CST		

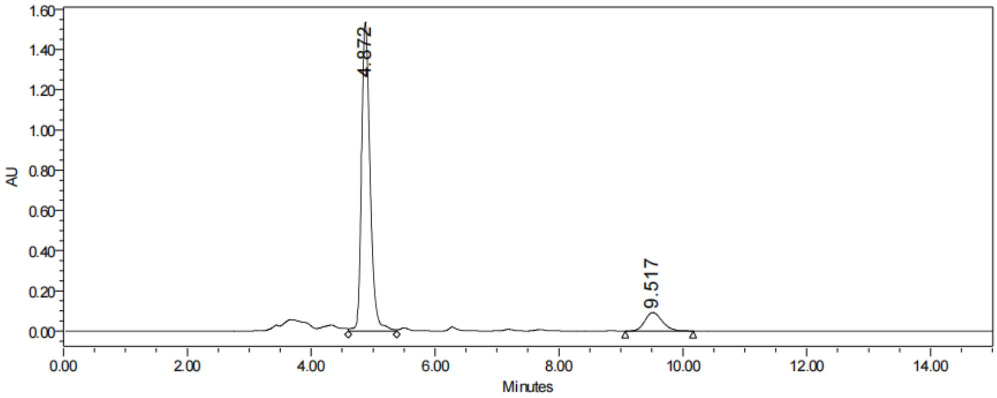

	RT	Area	% Area	Height
1	4.872	14808326	89.29	1534044
2	9.517	1776313	10.71	92073

Rac-**3aa**

SAMPLE INFORMATION

Sample Name:	zql-17-126-RAC-IE-20%	Acquired By:	System
Sample Type:	Unknown	Sample Set Name:	
Vial:	24	Acq. Method Set:	20%210400
Injection #:	1	Processing Method:	17991
Injection Volume:	10.00 ul	Channel Name:	254.0nm
Run Time:	13.0 Minutes	Proc. Chnl. Descr.:	2998 PDA 254.0 nm (2998
Date Acquired:	3/10/2024 2:44:54 PM CST		
Date Processed:	3/29/2024 8:10:44 PM CST		

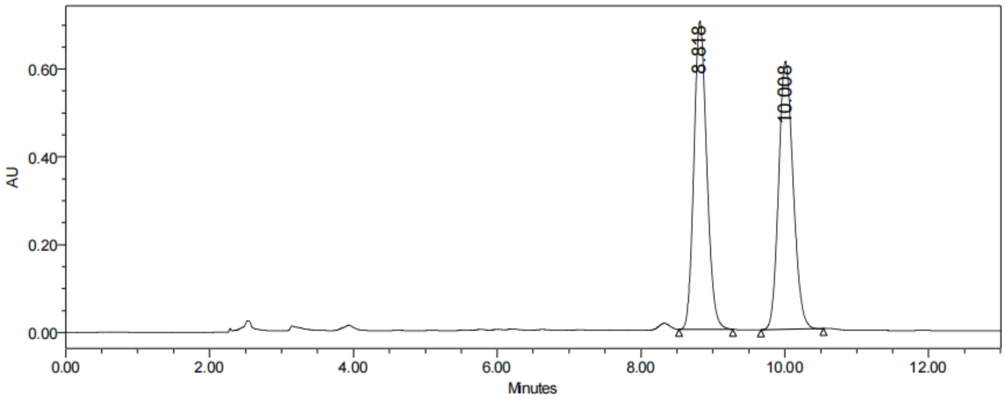

	RT	Area	% Area	Height
1	8.818	8760824	50.50	701671
2	10.008	8586547	49.50	609735

Asy-**3aa**

SAMPLE INFORMATION

Sample Name:	zql-17-220-IE-20%	Acquired By:	System
Sample Type:	Unknown	Sample Set Name:	0
Vial:	63	Acq. Method Set:	20%210400
Injection #:	1	Processing Method:	17993
Injection Volume:	10.00 ul	Channel Name:	254.0nm
Run Time:	13.0 Minutes	Proc. Chnl. Descr.:	2998 PDA 254.0 nm (2998
Date Acquired:	4/4/2024 5:12:49 PM CST		
Date Processed:	4/4/2024 8:03:05 PM CST		

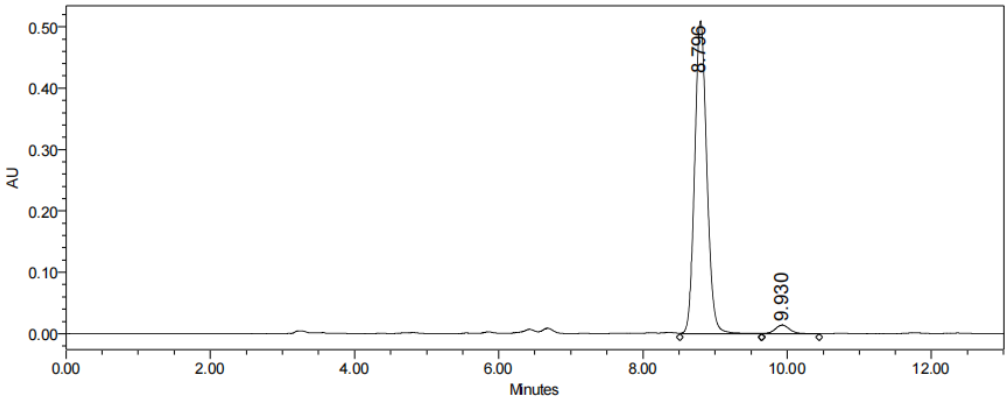

	RT	Area	% Area	Height
1	8.796	5972852	96.91	508883
2	9.930	190686	3.09	14047

Rac-**3ab**

SAMPLE INFORMATION

Sample Name:	zql-111-rac-IC-5%	Acquired By:	System
Sample Type:	Unknown	Sample Set Name:	0
Vial:	106	Acq. Method Set:	5%210400
Injection #:	1	Processing Method:	171071
Injection Volume:	10.00 ul	Channel Name:	254.0nm
Run Time:	20.0 Minutes	Proc. Chnl. Descr.:	2998 PDA 254.0 nm (2998
Date Acquired:	3/6/2024 7:34:08 PM CST		
Date Processed:	3/29/2024 8:31:40 PM CST		

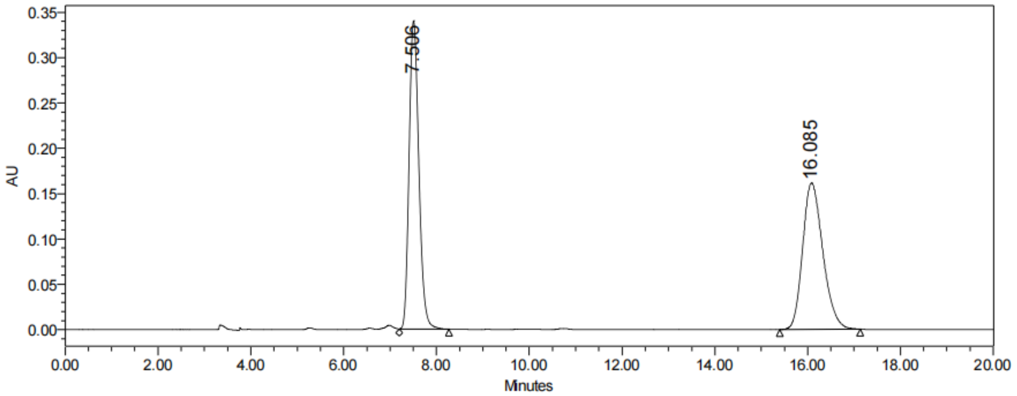

	RT	Area	% Area	Height
1	7.506	4985928	50.66	340018
2	16.085	4855415	49.34	161385

Asy-**3ab**

SAMPLE INFORMATION

Sample Name:	zql-17-107-IC-5%	Acquired By:	System
Sample Type:	Unknown	Sample Set Name:	0
Vial:	103	Acq. Method Set:	5%210400
Injection #:	1	Processing Method:	17107
Injection Volume:	10.00 ul	Channel Name:	254.0nm
Run Time:	20.0 Minutes	Proc. Chnl. Descr.:	2998 PDA 254.0 nm (2998
Date Acquired:	3/6/2024 10:14:46 PM CST		
Date Processed:	3/29/2024 8:26:53 PM CST		

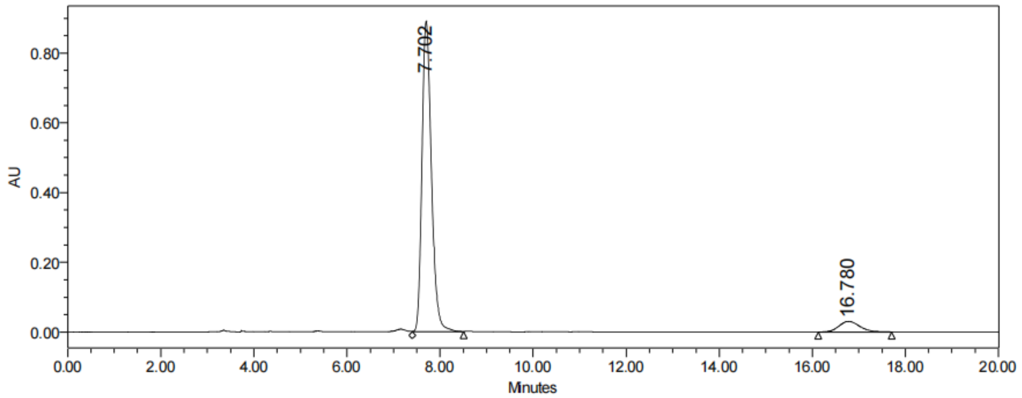

	RT	Area	% Area	Height
1	7.702	12599699	93.05	889888
2	16.780	940539	6.95	30443

Rac-**3ac**

SAMPLE INFORMATION

Sample Name:	zql-112-rac-IC-5%	Acquired By:	System
Sample Type:	Unknown	Sample Set Name:	0
Vial:	107	Acq. Method Set:	5%210400
Injection #:	1	Processing Method:	171082
Injection Volume:	10.00 ul	Channel Name:	254.0nm
Run Time:	20.0 Minutes	Proc. Chnl. Descr.:	2998 PDA 254.0 nm (2998
Date Acquired:	3/6/2024 7:54:50 PM CST		
Date Processed:	3/29/2024 8:30:52 PM CST		

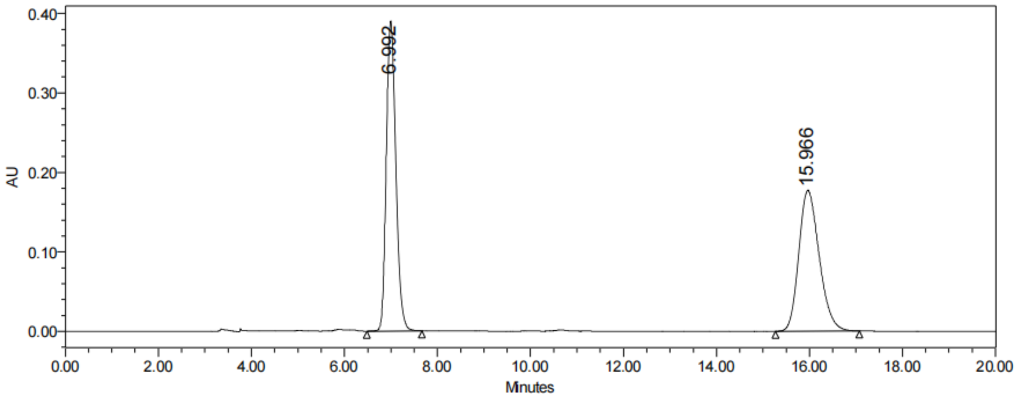

	RT	Area	% Area	Height
1	6.992	5541940	50.68	389568
2	15.966	5393345	49.32	177272

Asy-**3ac**

SAMPLE INFORMATION

Sample Name:	zql-17-108-IC-5%	Acquired By:	System
Sample Type:	Unknown	Sample Set Name:	0
Vial:	104	Acq. Method Set:	5%210400
Injection #:	1	Processing Method:	17108
Injection Volume:	10.00 ul	Channel Name:	254.0nm
Run Time:	20.0 Minutes	Proc. Chnl. Descr.:	2998 PDA 254.0 nm (2998
Date Acquired:	3/6/2024 10:35:27 PM CST		
Date Processed:	3/29/2024 8:26:02 PM CST		

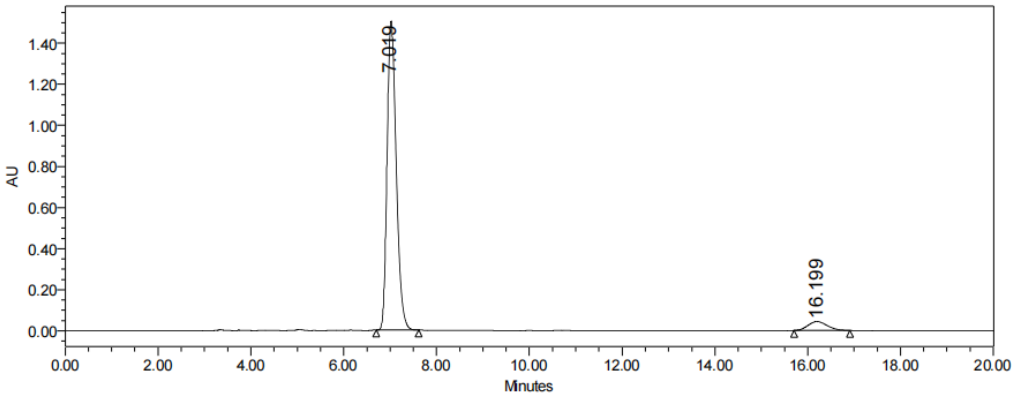

	RT	Area	% Area	Height
1	7.019	20557054	94.12	1501988
2	16.199	1284268	5.88	43503

Rac-**3ad**

SAMPLE INFORMATION

Sample Name:	zql-113-rac-IC-5%	Acquired By:	System
Sample Type:	Unknown	Sample Set Name:	0
Vial:	108	Acq. Method Set:	5%210400
Injection #:	1	Processing Method:	171092
Injection Volume:	10.00 ul	Channel Name:	235.0nm
Run Time:	20.0 Minutes	Proc. Chnl. Descr.:	2998 PDA 235.0 nm (2998
Date Acquired:	3/6/2024 8:15:31 PM CST		
Date Processed:	3/29/2024 8:30:01 PM CST		

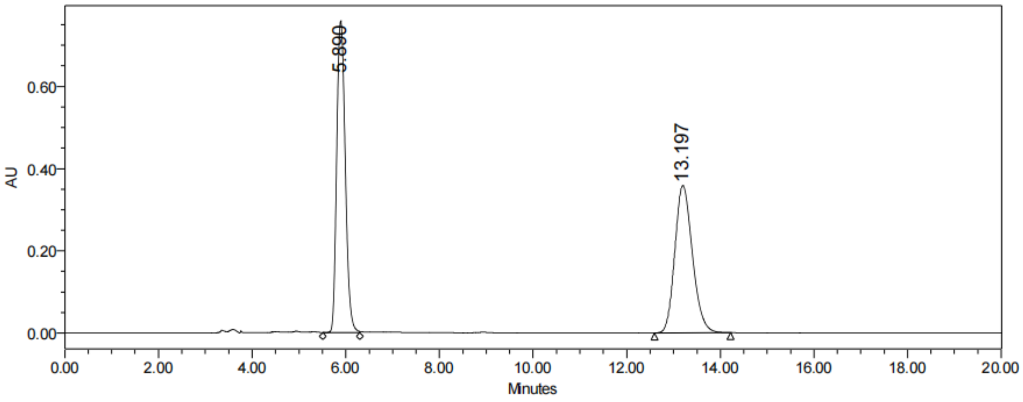

	RT	Area	% Area	Height
1	5.890	9686645	50.88	758293
2	13.197	9352493	49.12	358421

Asy-**3ad**

SAMPLE INFORMATION

Sample Name:	zql-17-109-IC-5%	Acquired By:	System
Sample Type:	Unknown	Sample Set Name:	0
Vial:	105	Acq. Method Set:	5%210400
Injection #:	1	Processing Method:	17109
Injection Volume:	10.00 ul	Channel Name:	235.0nm
Run Time:	20.0 Minutes	Proc. Chnl. Descr.:	2998 PDA 235.0 nm (2998
Date Acquired:	3/6/2024 10:56:10 PM CST		
Date Processed:	3/29/2024 8:24:45 PM CST		

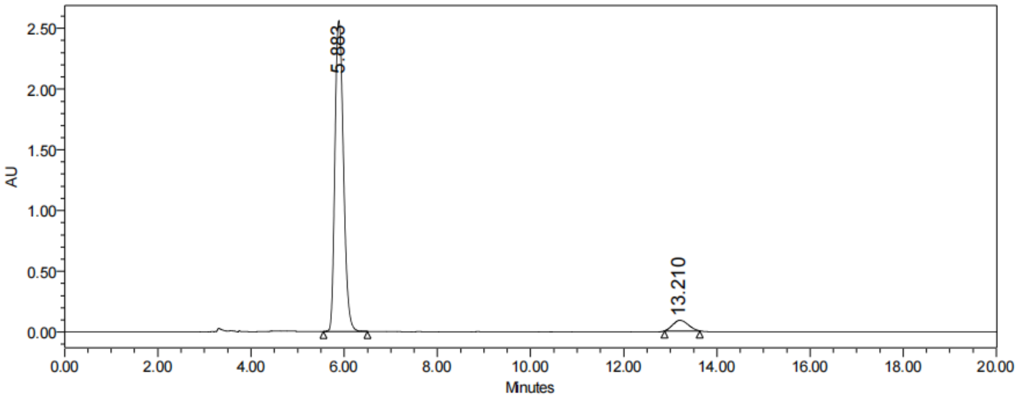

	RT	Area	% Area	Height
1	5.883	32650091	94.31	2556212
2	13.210	1971495	5.69	86734

Rac-**3ae**

SAMPLE INFORMATION			
Sample Name:	zql-114-rac-IC-5%	Acquired By:	System
Sample Type:	Unknown	Sample Set Name:	0
Vial:	109	Acq. Method Set:	5%210400
Injection #:	1	Processing Method:	171101
Injection Volume:	10.00 ul	Channel Name:	254.0nm
Run Time:	20.0 Minutes	Proc. Chnl. Descr.:	2998 PDA 254.0 nm (2998
Date Acquired:	3/6/2024 8:36:13 PM CST		
Date Processed:	3/29/2024 8:27:51 PM CST		

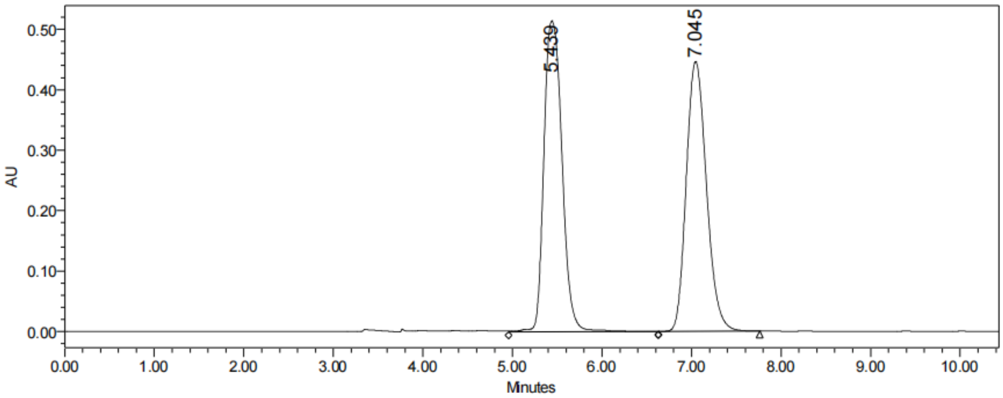

	RT	Area	% Area	Height
1	5.439	7226061	50.76	513765
2	7.045	7009910	49.24	446159

Asy-**3ae**

SAMPLE INFORMATION			
Sample Name:	zql-17-110-IC-5%	Acquired By:	System
Sample Type:	Unknown	Sample Set Name:	0
Vial:	106	Acq. Method Set:	5%210400
Injection #:	1	Processing Method:	17110
Injection Volume:	10.00 ul	Channel Name:	254.0nm
Run Time:	10.0 Minutes	Proc. Chnl. Descr.:	2998 PDA 254.0 nm (2998
Date Acquired:	3/6/2024 11:16:52 PM CST		
Date Processed:	3/29/2024 8:20:43 PM CST		

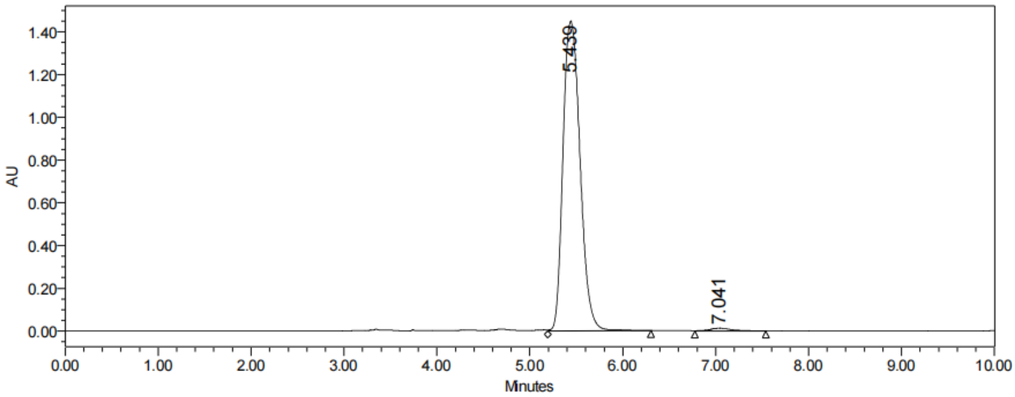

	RT	Area	% Area	Height
1	5.439	18484034	99.02	1448606
2	7.041	183079	0.98	11795

Rac-**3af**

SAMPLE INFORMATION			
Sample Name:	zql-17-208-RAC-IC-20%	Acquired By:	System
Sample Type:	Unknown	Sample Set Name:	0
Vial:	17	Acq. Method Set:	20%210400
Injection #:	1	Processing Method:	172072
Injection Volume:	10.00 ul	Channel Name:	254.0nm
Run Time:	30.0 Minutes	Proc. Chnl. Descr.:	2998 PDA 254.0 nm (2998
Date Acquired:	3/25/2024 8:08:39 PM CST		
Date Processed:	3/29/2024 8:32:43 PM CST		

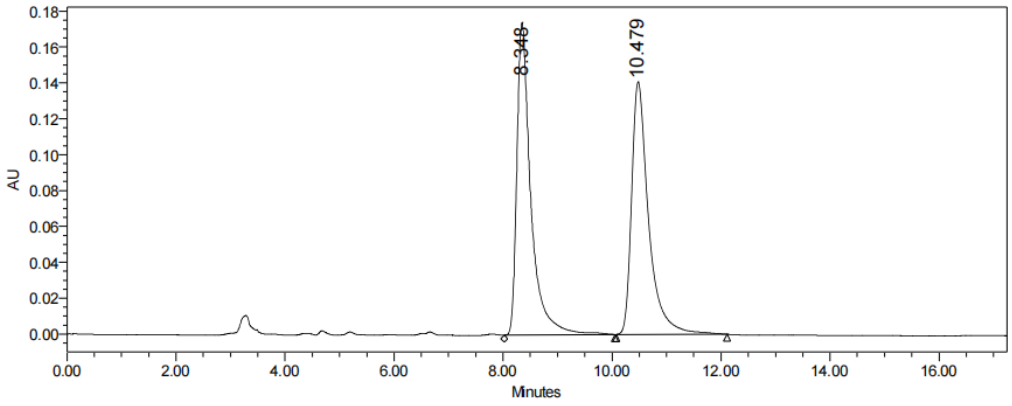

	RT	Area	% Area	Height
1	8.348	3101794	50.35	174184
2	10.479	3058349	49.65	140732

Asy-**3af**

SAMPLE INFORMATION			
Sample Name:	zql-17-207-IC-20%	Acquired By:	System
Sample Type:	Unknown	Sample Set Name:	0
Vial:	28	Acq. Method Set:	20%210400
Injection #:	1	Processing Method:	17207
Injection Volume:	10.00 ul	Channel Name:	254.0nm
Run Time:	17.0 Minutes	Proc. Chnl. Descr.:	2998 PDA 254.0 nm (2998
Date Acquired:	3/25/2024 8:27:44 PM CST		
Date Processed:	3/29/2024 8:33:05 PM CST		

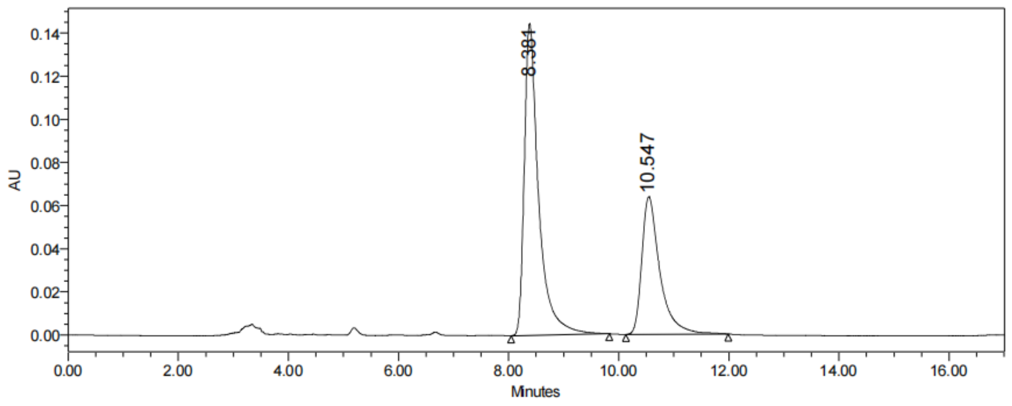

	RT	Area	% Area	Height
1	8.381	2587949	63.93	144439
2	10.547	1460295	36.07	63884

Rac-**3ag**

SAMPLE INFORMATION			
Sample Name:	zql-17-287-rac-2-IE-5%	Acquired By:	System
Sample Type:	Unknown	Sample Set Name:	0
Vial:	81	Acq. Method Set:	5%210400
Injection #:	1	Processing Method:	17287
Injection Volume:	10.00 ul	Channel Name:	254.0nm
Run Time:	16.0 Minutes	Proc. Chnl. Descr.:	2998 PDA 254.0 nm (2998
Date Acquired:	4/30/2024 9:45:47 PM CST		
Date Processed:	5/1/2024 11:24:34 AM CST		

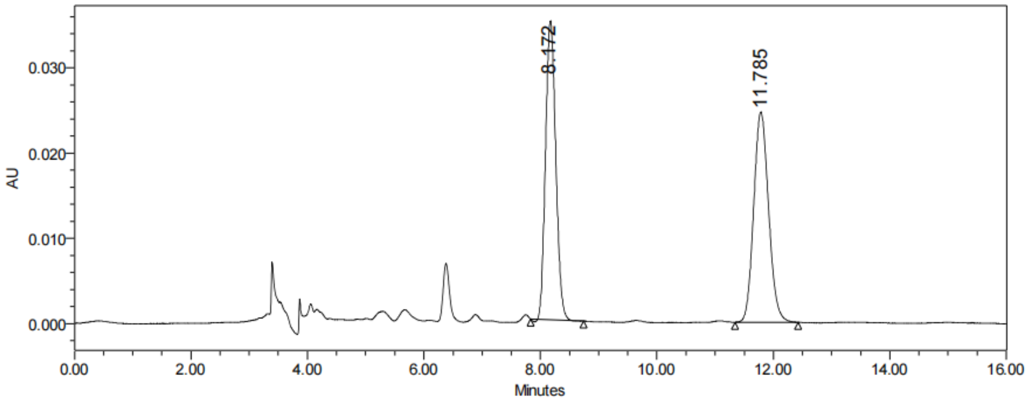

	RT	Area	% Area	Height
1	8.172	430566	49.59	35049
2	11.785	437673	50.41	24670

Asy-**3ag**

SAMPLE INFORMATION			
Sample Name:	zql-17-287-123-2-IE-5%	Acquired By:	System
Sample Type:	Unknown	Sample Set Name:	0
Vial:	82	Acq. Method Set:	5%210400
Injection #:	1	Processing Method:	172872
Injection Volume:	10.00 ul	Channel Name:	254.0nm
Run Time:	16.0 Minutes	Proc. Chnl. Descr.:	2998 PDA 254.0 nm (2998
Date Acquired:	4/30/2024 10:02:28 PM CST		
Date Processed:	5/1/2024 11:25:28 AM CST		

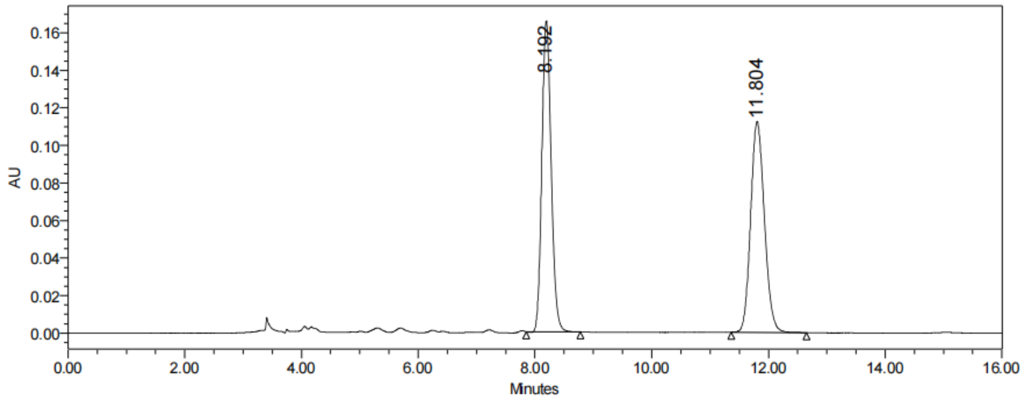

	RT	Area	% Area	Height
1	8.192	1836683	50.06	165459
2	11.804	1832351	49.94	112149

Rac-**8a**

SAMPLE INFORMATION

Sample Name:	zql-12-196-rac-IC-1%	Acquired By:	System
Sample Type:	Unknown	Sample Set Name:	
Vial:	106	Acq. Method Set:	1%210400
Injection #:	1	Processing Method:	122552
Injection Volume:	10.00 ul	Channel Name:	254.0nm
Run Time:	15.0 Minutes	Proc. Chnl. Descr.:	2998 PDA 254.0 nm (2998
Date Acquired:	10/20/2022 7:56:19 PM CST		
Date Processed:	10/20/2022 8:14:58 PM CST		

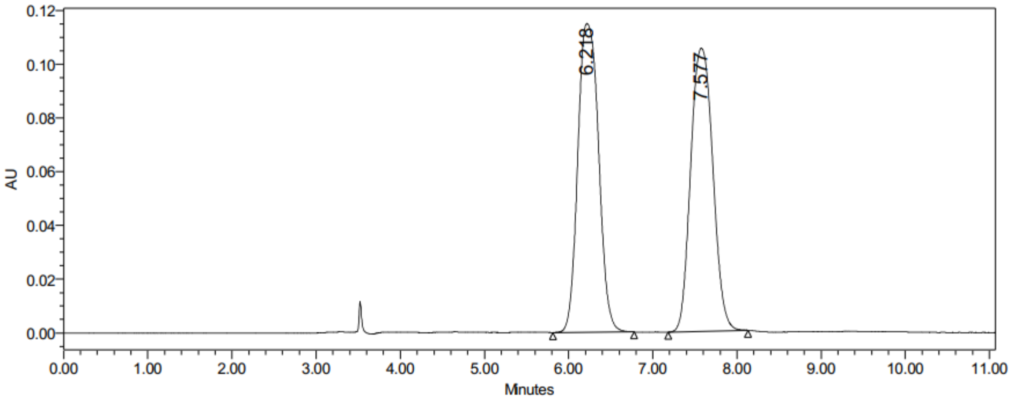

	RT	Area	% Area	Height
1	6.218	1963752	50.35	114823
2	7.577	1936531	49.65	105381

Asy-**8a**

SAMPLE INFORMATION

Sample Name:	zql-12-255-IC-1%	Acquired By:	System
Sample Type:	Unknown	Sample Set Name:	0
Vial:	40	Acq. Method Set:	1%210400
Injection #:	1	Processing Method:	12255
Injection Volume:	10.00 ul	Channel Name:	254.0nm
Run Time:	10.0 Minutes	Proc. Chnl. Descr.:	2998 PDA 254.0 nm (2998
Date Acquired:	8/13/2022 8:27:05 PM CST		
Date Processed:	10/20/2022 8:12:52 PM CST		

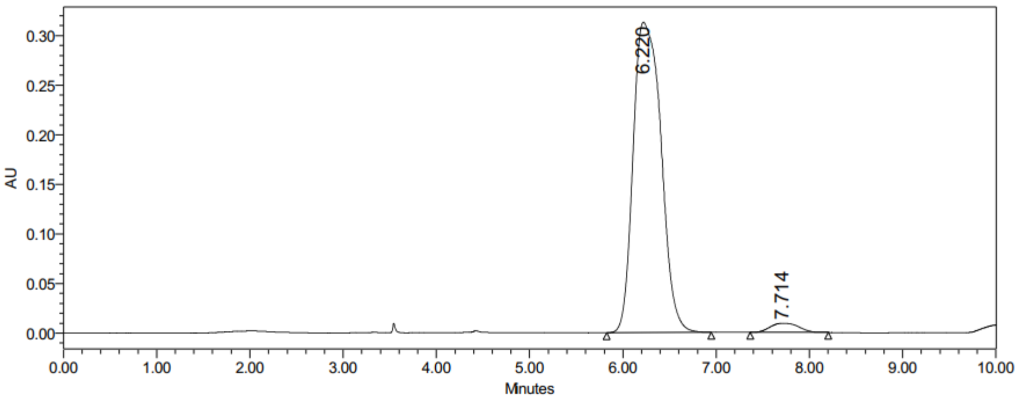

	RT	Area	% Area	Height
1	6.220	6505777	97.08	312803
2	7.714	195620	2.92	9146

Rac-**8b**

SAMPLE INFORMATION

Sample Name:	zql-13-55-2-rac-IG-5%	Acquired By:	System
Sample Type:	Unknown	Sample Set Name:	
Vial:	57	Acq. Method Set:	5%210400
Injection #:	1	Processing Method:	13523
Injection Volume:	10.00 ul	Channel Name:	254.0nm
Run Time:	30.0 Minutes	Proc. Chnl. Descr.:	2998 PDA 254.0 nm (2998
Date Acquired:	10/10/2022 4:24:04 PM CST		
Date Processed:	10/10/2022 7:10:45 PM CST		

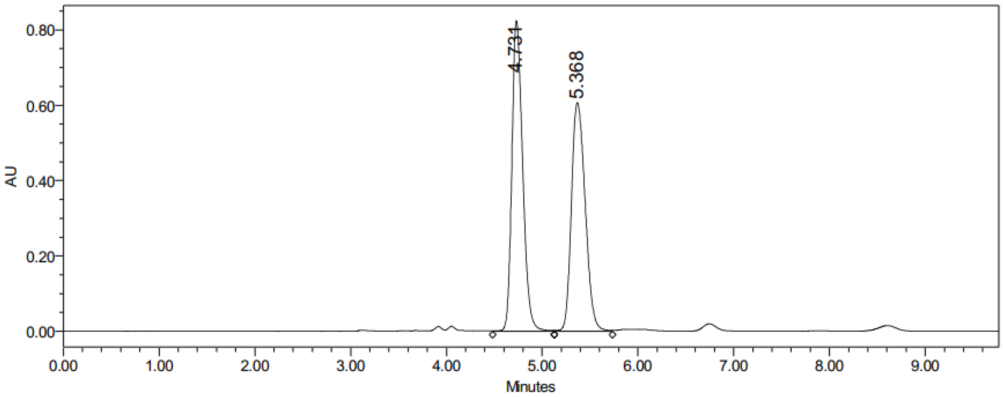

	RT	Area	% Area	Height
1	4.731	6453059	51.50	823599
2	5.368	6077344	48.50	606965

Asy-**8b**

SAMPLE INFORMATION

Sample Name:	zql-13-56-2-ASY-IG-5%	Acquired By:	System
Sample Type:	Unknown	Sample Set Name:	0
Vial:	35	Acq. Method Set:	5%210400
Injection #:	1	Processing Method:	13562
Injection Volume:	10.00 ul	Channel Name:	254.0nm
Run Time:	10.0 Minutes	Proc. Chnl. Descr.:	2998 PDA 254.0 nm (2998
Date Acquired:	10/10/2022 5:29:58 PM CST		
Date Processed:	10/10/2022 7:09:37 PM CST		

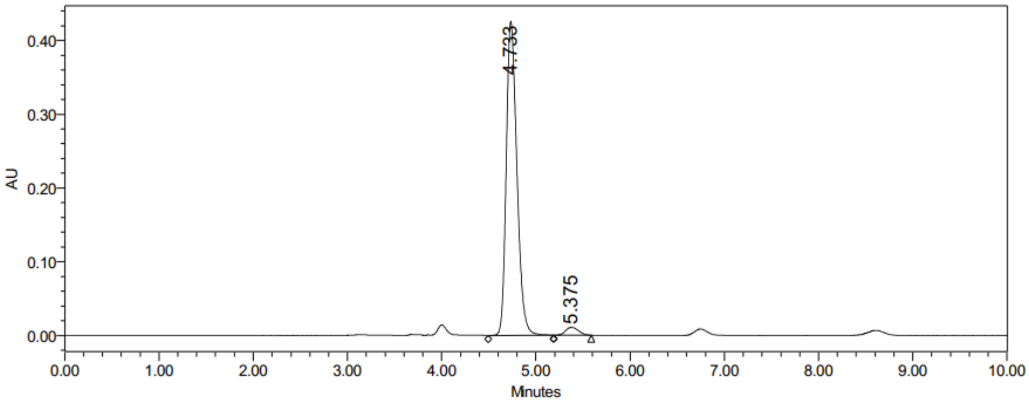

	RT	Area	% Area	Height
1	4.733	3357781	96.94	425925
2	5.375	106114	3.06	10884

Rac-**8c**

SAMPLE INFORMATION

Sample Name:	zql-13-67-rac-IG-5%	Acquired By:	System
Sample Type:	Unknown	Sample Set Name	
Vial:	84	Acq. Method Set:	5%210400
Injection #:	1	Processing Method	1368
Injection Volume:	10.00 ul	Channel Name:	254.0nm
Run Time:	10.0 Minutes	Proc. Chnl. Descr.:	2998 PDA 254.0 nm (2998
Date Acquired:	10/12/2022 8:49:55 PM CST		
Date Processed:	10/12/2022 9:42:24 PM CST		

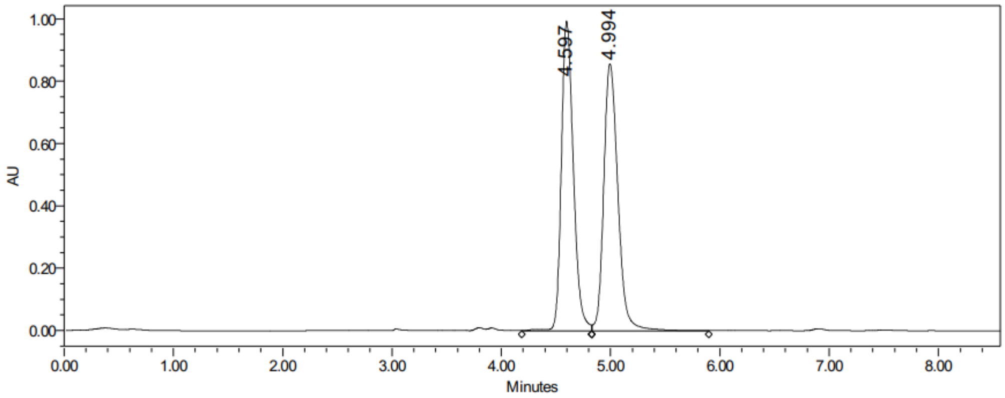

	RT	Area	% Area	Height
1	4.597	7586155	49.47	993928
2	4.994	7748758	50.53	857009

Asy-**8c**

SAMPLE INFORMATION

Sample Name:	zql-17-182-IG-5%	Acquired By:	System
Sample Type:	Unknown	Sample Set Name:	
Vial:	5	Acq. Method Set:	5%210400
Injection #:	1	Processing Method:	8c
Injection Volume:	10.00 ul	Channel Name:	254.0nm
Run Time:	15.0 Minutes	Proc. Chnl. Descr.:	2998 PDA 254.0 nm (2998
Date Acquired:	3/20/2024 10:10:48 AM CST		
Date Processed:	4/2/2024 10:05:20 PM CST		

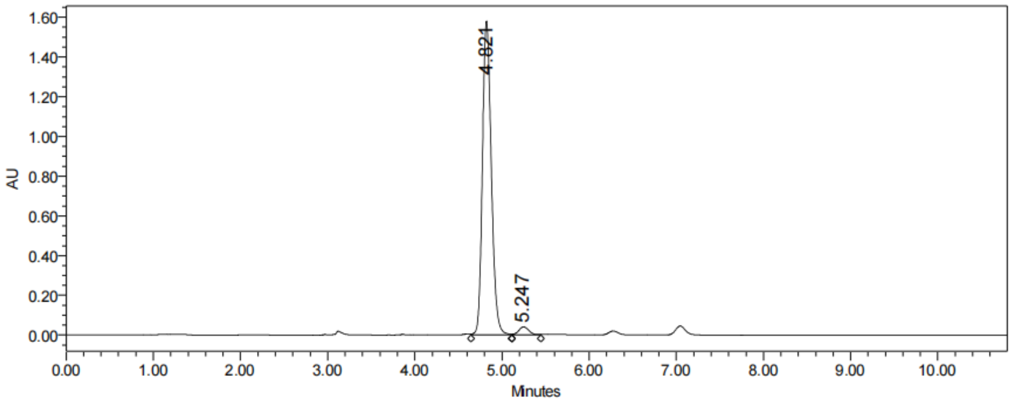

	RT	Area	% Area	Height
1	4.821	11229767	97.10	1579012
2	5.247	335132	2.90	40776

Rac-**8d**

SAMPLE INFORMATION

Sample Name:	zql-13-69-rac-IG-5%	Acquired By:	System
Sample Type:	Unknown	Sample Set Name	
Vial:	113	Acq. Method Set:	5%210400
Injection #:	1	Processing Method	1370
Injection Volume:	10.00 ul	Channel Name:	254.0nm
Run Time:	10.0 Minutes	Proc. Chnl. Descr.:	2998 PDA 254.0 nm (2998
Date Acquired:	10/12/2022 8:59:49 PM CST		
Date Processed:	10/12/2022 9:44:28 PM CST		

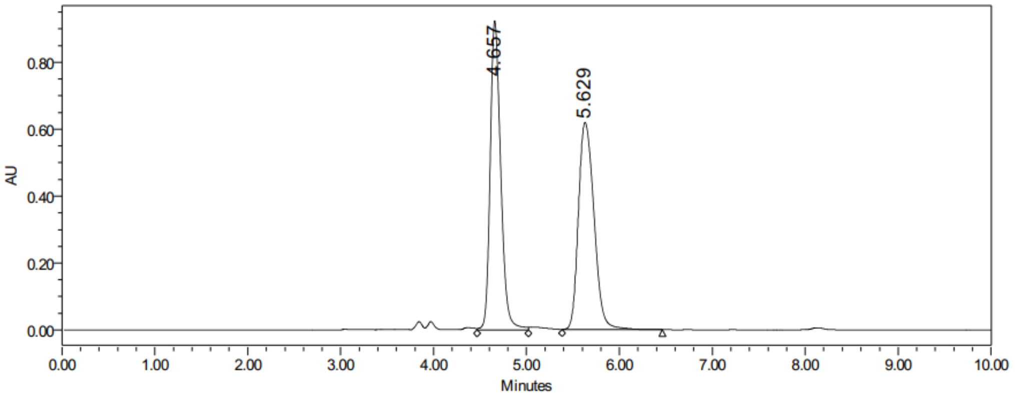

	RT	Area	% Area	Height
1	4.657	7307489	50.19	923007
2	5.629	7251744	49.81	619721

Asy-**8d**

SAMPLE INFORMATION

Sample Name:	zql-13-70-asy-IG-5%	Acquired By:	System
Sample Type:	Unknown	Sample Set Name	0
Vial:	24	Acq. Method Set:	5%210400
Injection #:	1	Processing Method	13702
Injection Volume:	10.00 ul	Channel Name:	254.0nm
Run Time:	10.0 Minutes	Proc. Chnl. Descr.:	2998 PDA 254.0 nm (2998
Date Acquired:	10/12/2022 9:22:33 PM CST		
Date Processed:	10/12/2022 9:46:20 PM CST		

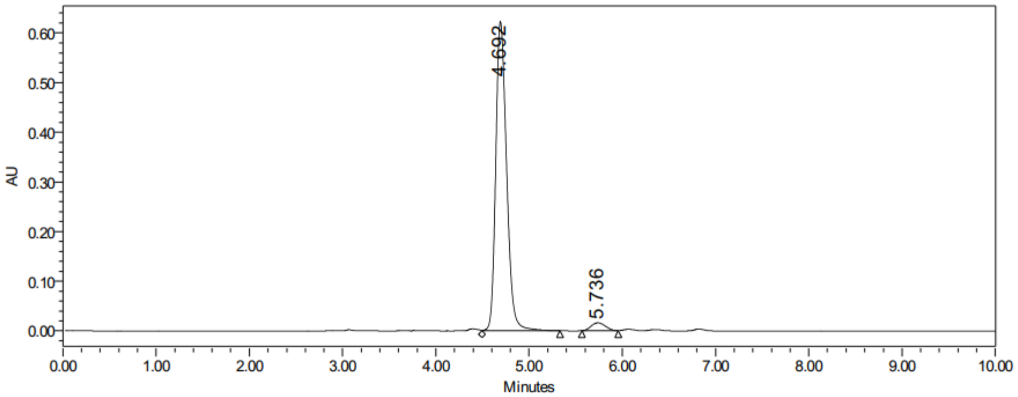

	RT	Area	% Area	Height
1	4.692	5061347	96.86	623216
2	5.736	164042	3.14	15682

Rac-**8e**

SAMPLE INFORMATION

Sample Name:	zql-13-73-rac-IG-5%	Acquired By:	System
Sample Type:	Unknown	Sample Set Name:	
Vial:	19	Acq. Method Set:	5%210400
Injection #:	1	Processing Method:	13732
Injection Volume:	10.00 ul	Channel Name:	254.0nm
Run Time:	11.0 Minutes	Proc. Chnl. Descr.:	2998 PDA 254.0 nm (2998
Date Acquired:	11/7/2022 9:47:25 AM CST		
Date Processed:	11/7/2022 10:05:38 AM CST		

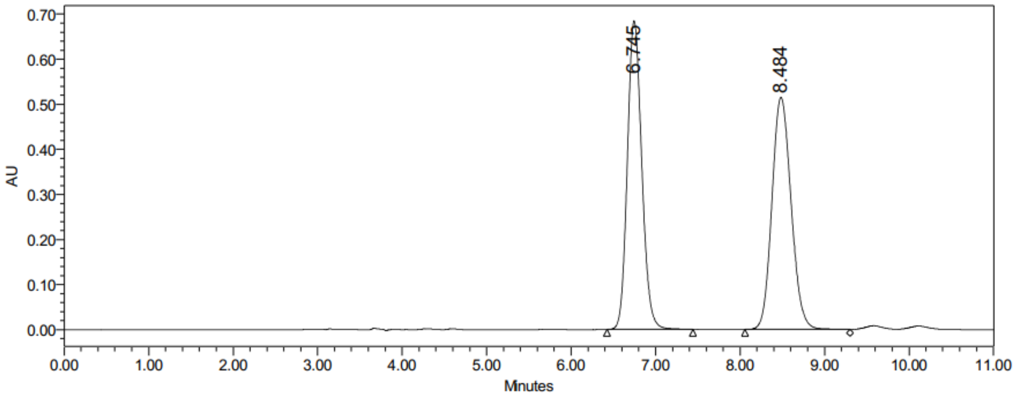

	RT	Area	% Area	Height
1	6.745	8138173	49.95	684578
2	8.484	8152901	50.05	515136

Asy-**8e**

SAMPLE INFORMATION

Sample Name:	zql-17-214-IG-5%	Acquired By:	System
Sample Type:	Unknown	Sample Set Name	0
Vial:	91	Acq. Method Set:	5%210400
Injection #:	1	Processing Method	8e
Injection Volume:	10.00 ul	Channel Name:	254.0nm
Run Time:	15.0 Minutes	Proc. Chnl. Descr.:	2998 PDA 254.0 nm (2998
Date Acquired:	3/28/2024 22:35:46 CST		
Date Processed:	4/2/2024 22:07:52 CST		

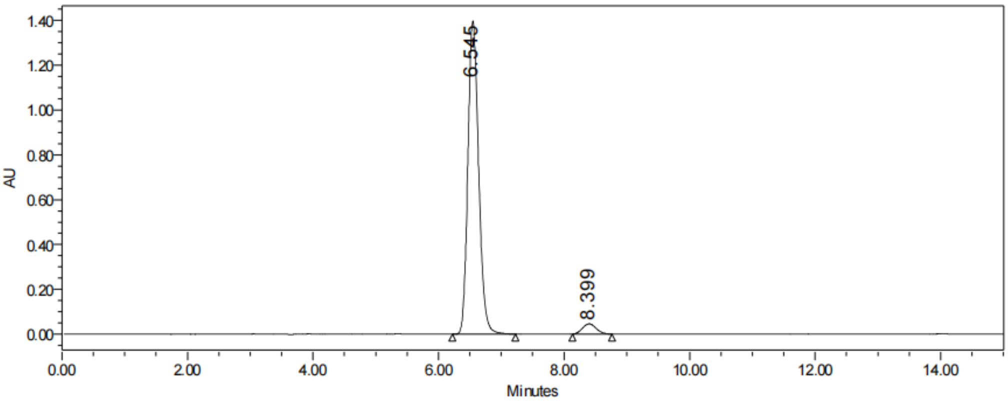

	RT	Area	% Area	Height
1	6.545	16501291	95.93	1393684
2	8.399	699427	4.07	45697

Rac-**8f**

SAMPLE INFORMATION

Sample Name:	zql-12-251-rac-IC-1%	Acquired By:	System
Sample Type:	Unknown	Sample Set Name:	
Vial:	55	Acq. Method Set:	1%210400
Injection #:	1	Processing Method:	12261
Injection Volume:	10.00 ul	Channel Name:	292.0nm
Run Time:	20.0 Minutes	Proc. Chnl. Descr.:	2998 PDA 292.0 nm (2998
Date Acquired:	8/13/2022 8:55:15 PM CST		
Date Processed:	9/15/2022 9:33:13 PM CST		

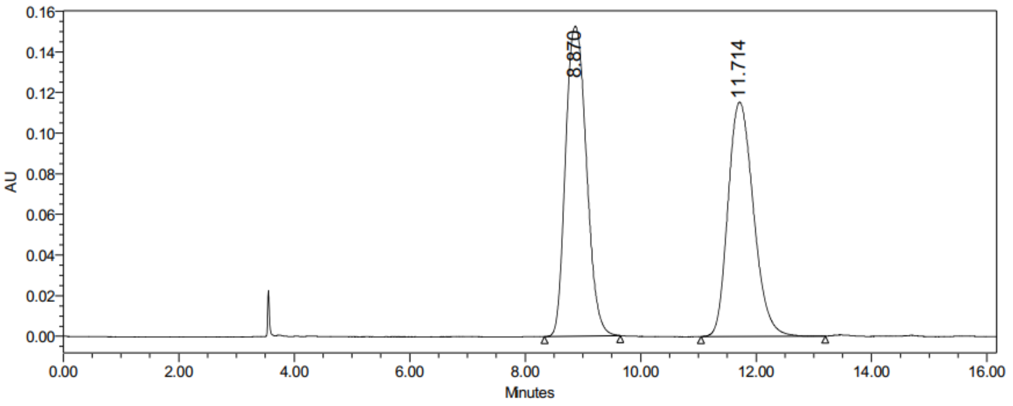

	RT	Area	% Area	Height
1	8.870	3829660	52.12	152582
2	11.714	3518010	47.88	115315

Asy-**8f**

SAMPLE INFORMATION

Sample Name:	zql-12-261-IC-1%	Acquired By:	System
Sample Type:	Unknown	Sample Set Name	0
Vial:	46	Acq. Method Set:	1%210400
Injection #:	1	Processing Method	12261
Injection Volume:	10.00 ul	Channel Name:	292.0nm
Run Time:	16.0 Minutes	Proc. Chnl. Descr.:	2998 PDA 292.0 nm (2998
Date Acquired:	8/16/2022 10:22:31 AM CST		
Date Processed:	9/15/2022 9:28:38 PM CST		

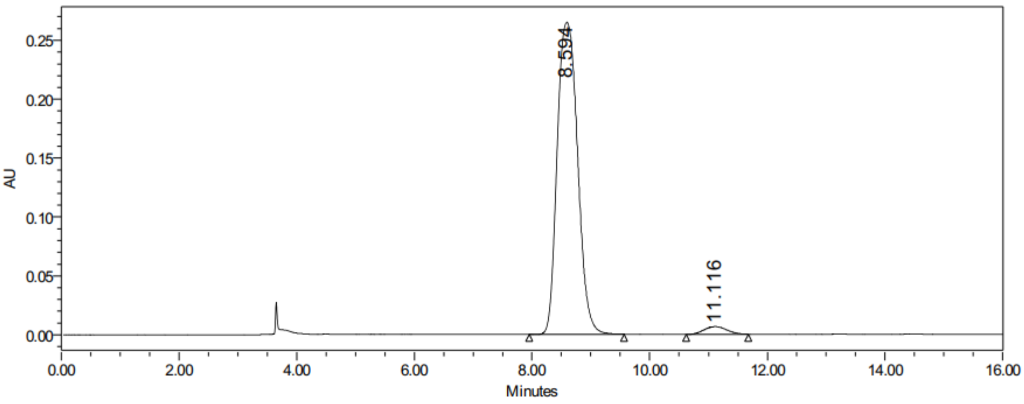

	RT	Area	% Area	Height
1	8.594	6364876	97.34	264776
2	11.116	173618	2.66	6500

Rac-**8g**

SAMPLE INFORMATION

Sample Name:	zql-13-59-rac-IG-5%	Acquired By:	System
Sample Type:	Unknown	Sample Set Name	
Vial:	81	Acq. Method Set:	5%210400
Injection #:	1	Processing Method	1260
Injection Volume:	15.00 ul	Channel Name:	254.0nm
Run Time:	30.0 Minutes	Proc. Chnl. Descr.:	2998 PDA 254.0 nm (2998
Date Acquired:	10/11/2022 9:04:17 PM CST		
Date Processed:	10/11/2022 10:27:29 PM CST		

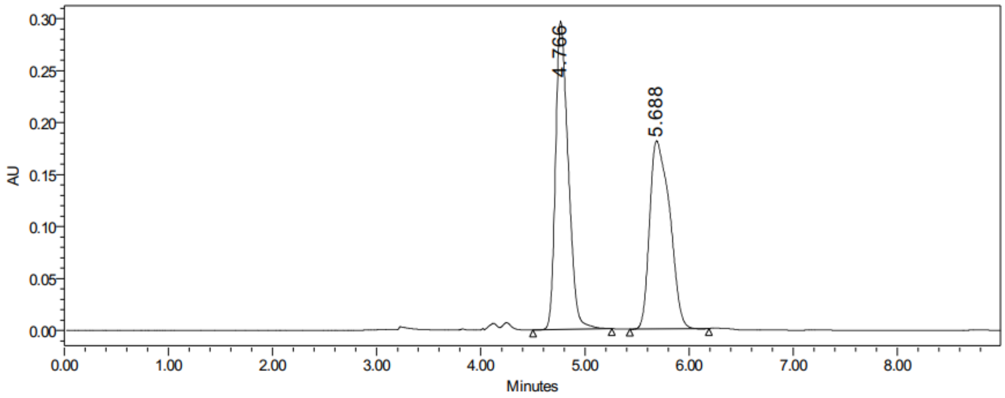

	RT	Area	% Area	Height
1	4.766	2506386	50.85	296825
2	5.688	2422746	49.15	181307

Asy-**8g**

SAMPLE INFORMATION

Sample Name:	zql-13-60-2-asy-IG-5%	Acquired By:	System
Sample Type:	Unknown	Sample Set Name	0
Vial:	84	Acq. Method Set:	5%210400
Injection #:	1	Processing Method	13602
Injection Volume:	15.00 ul	Channel Name:	254.0nm
Run Time:	9.0 Minutes	Proc. Chnl. Descr.:	2998 PDA 254.0 nm (2998
Date Acquired:	10/11/2022 10:00:41 PM CST		
Date Processed:	10/11/2022 10:28:23 PM CST		

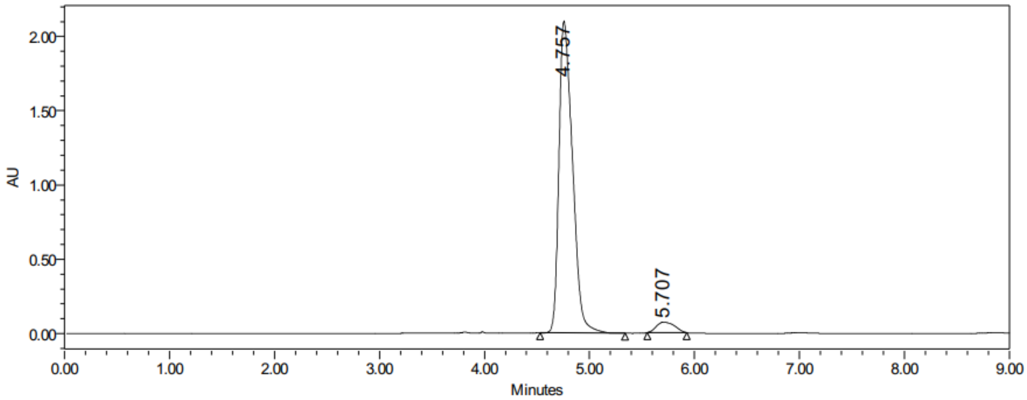

	RT	Area	% Area	Height
1	4.757	18741351	95.67	2099488
2	5.707	849036	4.33	70310

Rac-**8h**

SAMPLE INFORMATION

Sample Name:	zql-13-57-rac-IG-5%	Acquired By:	System
Sample Type:	Unknown	Sample Set Name	0
Vial:	81	Acq. Method Set:	5%210400
Injection #:	1	Processing Method	1358
Injection Volume:	15.00 ul	Channel Name:	254.0nm
Run Time:	9.0 Minutes	Proc. Chnl. Descr.:	2998 PDA 254.0 nm (2998
Date Acquired:	10/11/2022 9:31:33 PM CST		
Date Processed:	10/11/2022 10:25:25 PM CST		

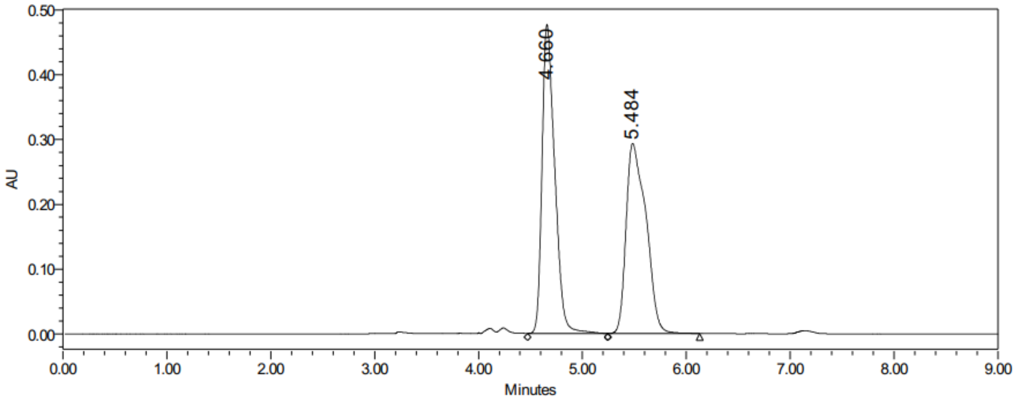

	RT	Area	% Area	Height
1	4.660	4038613	50.96	477075
2	5.484	3886297	49.04	293501

Asy-**8h**

SAMPLE INFORMATION

Sample Name:	zql-13-58-asy-IG-5%	Acquired By:	System
Sample Type:	Unknown	Sample Set Name	0
Vial:	83	Acq. Method Set:	5%210400
Injection #:	1	Processing Method	13582
Injection Volume:	15.00 ul	Channel Name:	254.0nm
Run Time:	9.0 Minutes	Proc. Chnl. Descr.:	2998 PDA 254.0 nm (2998
Date Acquired:	10/11/2022 9:50:58 PM CST		
Date Processed:	10/11/2022 10:26:14 PM CST		

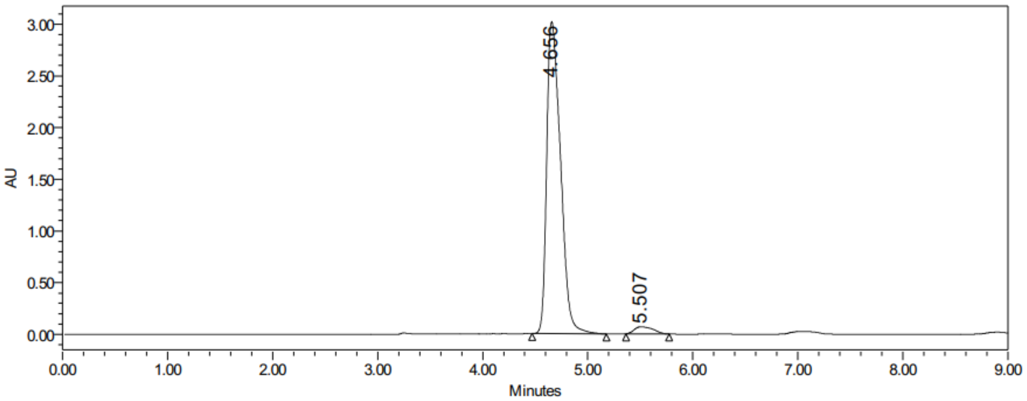

	RT	Area	% Area	Height
1	4.656	27413984	96.95	3016013
2	5.507	862597	3.05	69040

Rac-**8i**

SAMPLE INFORMATION

Sample Name:	zql-13-51-rac-IG-5%	Acquired By:	System
Sample Type:	Unknown	Sample Set Name:	
Vial:	111	Acq. Method Set:	5%210400
Injection #:	1	Processing Method:	13522
Injection Volume:	10.00 ul	Channel Name:	254.0nm
Run Time:	30.0 Minutes	Proc. Chnl. Descr.:	2998 PDA 254.0 nm (2998
Date Acquired:	10/10/2022 3:59:06 PM CST		
Date Processed:	10/10/2022 5:14:26 PM CST		

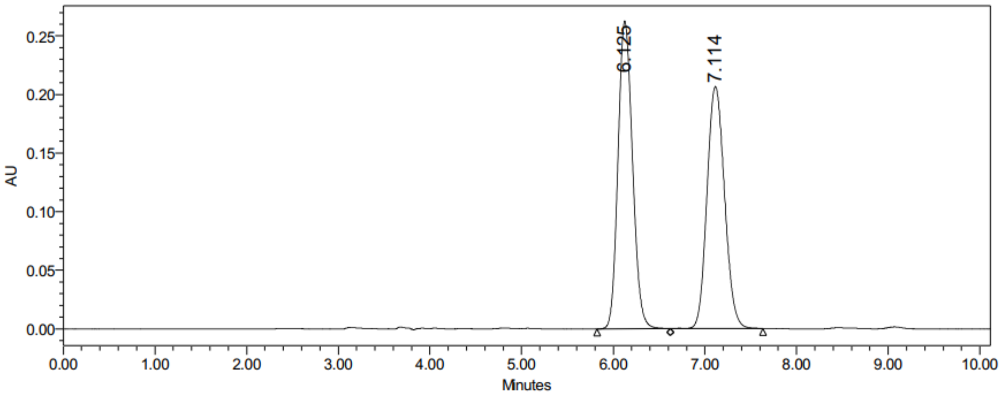

	RT	Area	% Area	Height
1	6.125	2898052	51.28	262567
2	7.114	2753423	48.72	206602

Asy-**8i**

SAMPLE INFORMATION

Sample Name:	zql-13-52-ASY-IG-5%	Acquired By:	System
Sample Type:	Unknown	Sample Set Name:	0
Vial:	31	Acq. Method Set:	5%210400
Injection #:	1	Processing Method:	1352
Injection Volume:	10.00 ul	Channel Name:	254.0nm
Run Time:	10.0 Minutes	Proc. Chnl. Descr.:	2998 PDA 254.0 nm (2998
Date Acquired:	10/10/2022 4:47:08 PM CST		
Date Processed:	10/10/2022 5:12:35 PM CST		

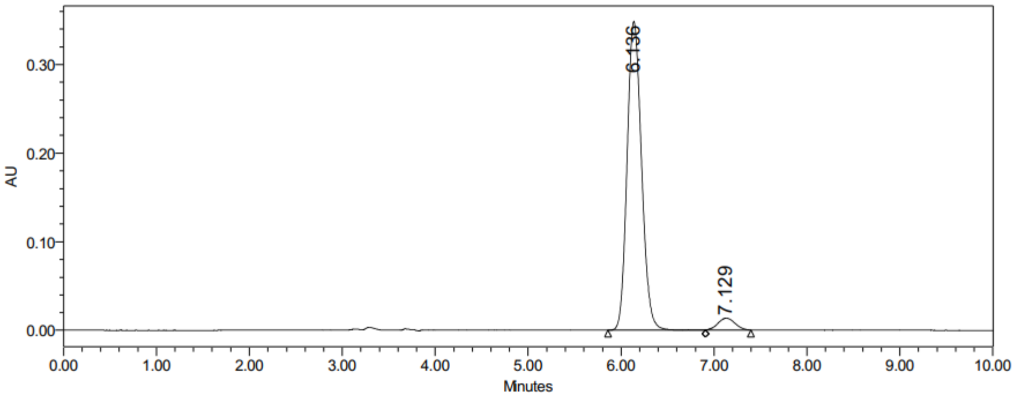

	RT	Area	% Area	Height
1	6.136	3773586	95.65	348792
2	7.129	171455	4.35	13442

Rac-**8j**

SAMPLE INFORMATION

Sample Name:	zql-13-61-rac-IG-5%	Acquired By:	System
Sample Type:	Unknown	Sample Set Name	0
Vial:	82	Acq. Method Set:	5%210400
Injection #:	1	Processing Method	1262
Injection Volume:	15.00 ul	Channel Name:	254.0nm
Run Time:	9.0 Minutes	Proc. Chnl. Descr.:	2998 PDA 254.0 nm (2998
Date Acquired:	10/11/2022 9:41:15 PM CST		
Date Processed:	10/11/2022 10:29:52 PM CST		

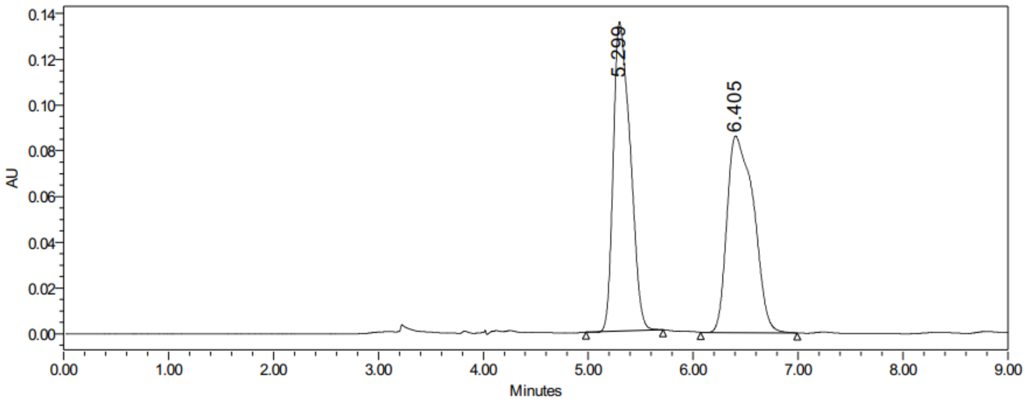

	RT	Area	% Area	Height
1	5.299	1520095	50.07	135204
2	6.405	1515841	49.93	85923

Asy-**8j**

SAMPLE INFORMATION

Sample Name:	zql-13-62-asy-IG-5%	Acquired By:	System
Sample Type:	Unknown	Sample Set Name	0
Vial:	85	Acq. Method Set:	5%210400
Injection #:	1	Processing Method	13622
Injection Volume:	15.00 ul	Channel Name:	254.0nm
Run Time:	9.0 Minutes	Proc. Chnl. Descr.:	2998 PDA 254.0 nm (2998
Date Acquired:	10/11/2022 10:10:24 PM CST		
Date Processed:	10/11/2022 10:30:48 PM CST		

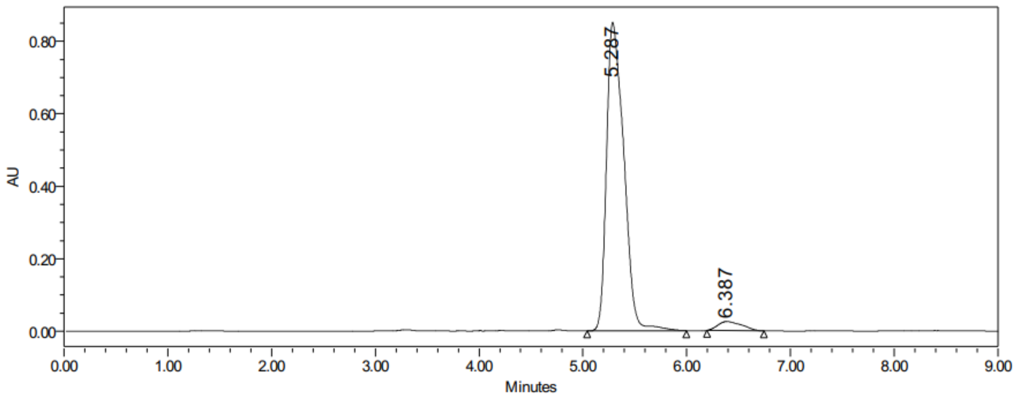

	RT	Area	% Area	Height
1	5.287	9873347	96.05	851055
2	6.387	406271	3.95	25025

Rac-**8k**

SAMPLE INFORMATION

Sample Name:	zql-13-71-rac-IG-5% 07ML	Acquired By:	System
Sample Type:	Unknown	Sample Set Name	
Vial:	44	Acq. Method Set:	5%210400 07ML
Injection #:	1	Processing Method	1372
Injection Volume:	5.00 ul	Channel Name:	254.0nm
Run Time:	30.0 Minutes	Proc. Chnl. Descr.:	2998 PDA 254.0 nm (2998
Date Acquired:	10/13/2022 7:09:52 PM CST		
Date Processed:	10/13/2022 7:40:07 PM CST		

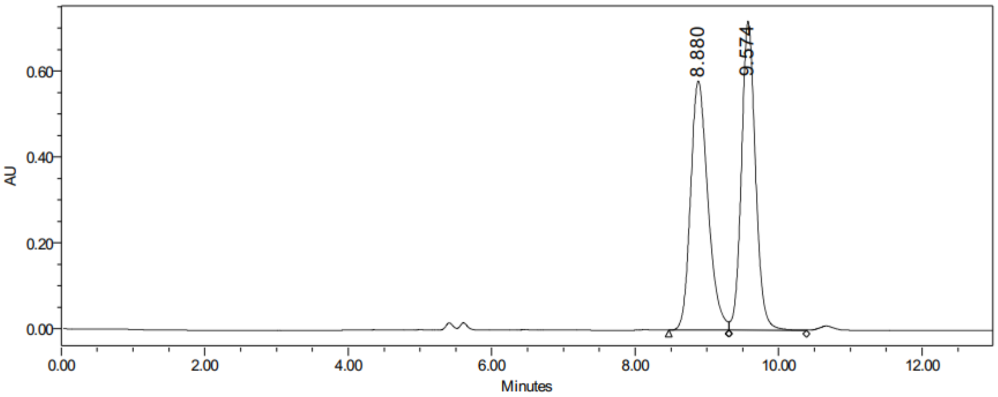

	RT	Area	% Area	Height
1	8.880	9748235	50.07	580690
2	9.574	9720391	49.93	719787

Asy-**8k**

SAMPLE INFORMATION

Sample Name:	zql-13-72-ASY-IG-5% 07ML	Acquired By:	System
Sample Type:	Unknown	Sample Set Name	
Vial:	67	Acq. Method Set:	5%210400 07ML
Injection #:	1	Processing Method	13722
Injection Volume:	5.00 ul	Channel Name:	254.0nm
Run Time:	13.0 Minutes	Proc. Chnl. Descr.:	2998 PDA 254.0 nm (2998
Date Acquired:	10/13/2022 7:23:46 PM CST		
Date Processed:	10/13/2022 7:41:02 PM CST		

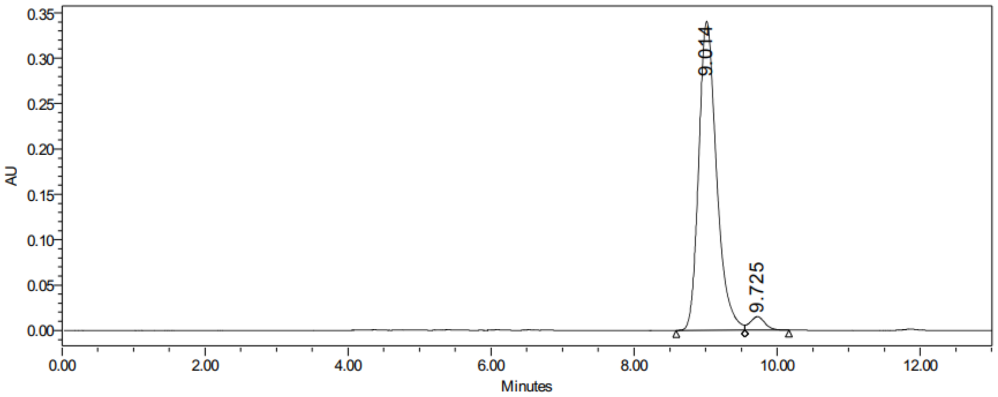

	RT	Area	% Area	Height
1	9.014	5912672	96.46	340422
2	9.725	216747	3.54	14943

Rac-**8l**

SAMPLE INFORMATION

Sample Name:	zql-12-252-rac-IC-1%	Acquired By:	System
Sample Type:	Unknown	Sample Set Name:	0
Vial:	80	Acq. Method Set:	1%210400
Injection #:	1	Processing Method:	12262
Injection Volume:	10.00 ul	Channel Name:	247.0nm
Run Time:	16.0 Minutes	Proc. Chnl. Descr.:	2998 PDA 247.0 nm (2998
Date Acquired:	8/13/2022 9:13:17 PM CST		
Date Processed:	9/15/2022 9:36:02 PM CST		

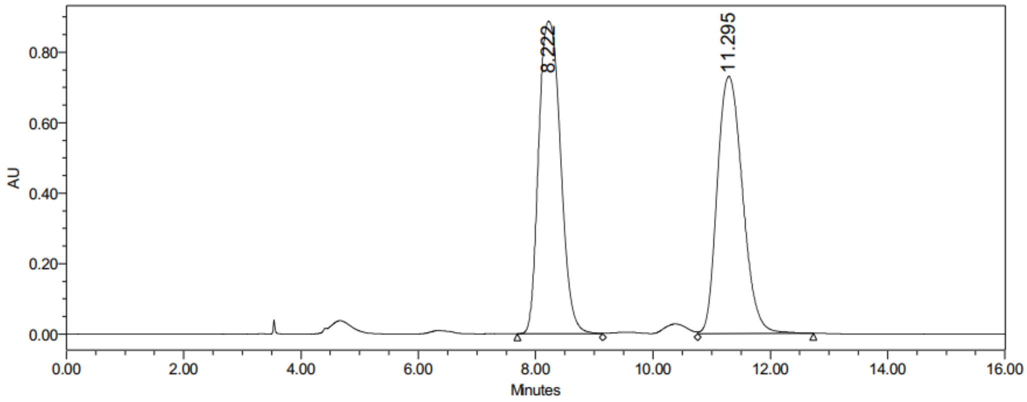

	RT	Area	% Area	Height
1	8.222	23029253	51.29	887184
2	11.295	21874029	48.71	730079

Asy-**8l**

SAMPLE INFORMATION

Sample Name:	zql-12-262-IC-1%	Acquired By:	System
Sample Type:	Unknown	Sample Set Name	0
Vial:	47	Acq. Method Set:	1%210400
Injection #:	1	Processing Method	12262
Injection Volume:	10.00 ul	Channel Name:	247.0nm
Run Time:	16.0 Minutes	Proc. Chnl. Descr.:	2998 PDA 247.0 nm (2998
Date Acquired:	8/16/2022 10:39:10 AM CST		
Date Processed:	9/15/2022 9:33:27 PM CST		

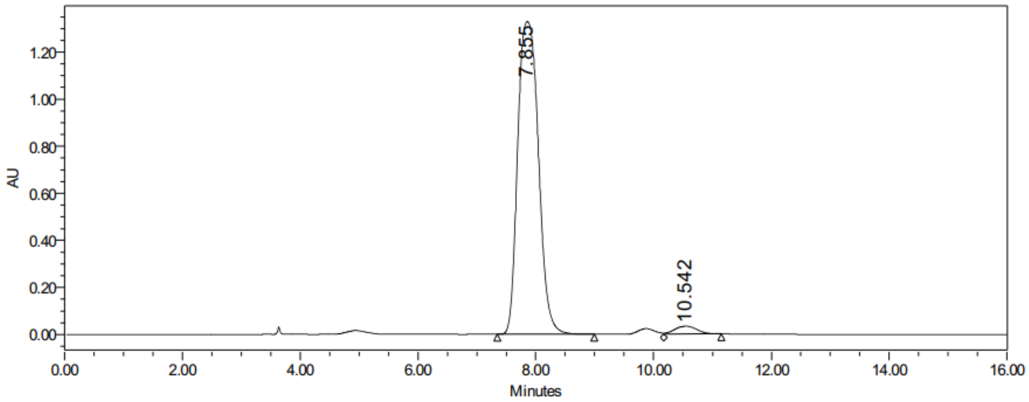

	RT	Area	% Area	Height
1	7.855	32232509	97.41	1329389
2	10.542	857566	2.59	32976

Rac-**8m**

SAMPLE INFORMATION

Sample Name:	zql-13-53-RAC-IG-5%	Acquired By:	System
Sample Type:	Unknown	Sample Set Name:	0
Vial:	32	Acq. Method Set:	5%210400
Injection #:	1	Processing Method:	13542
Injection Volume:	10.00 ul	Channel Name:	254.0nm
Run Time:	10.0 Minutes	Proc. Chnl. Descr.:	2998 PDA 254.0 nm (2998
Date Acquired:	10/10/2022 4:57:50 PM CST		
Date Processed:	10/10/2022 5:27:45 PM CST		

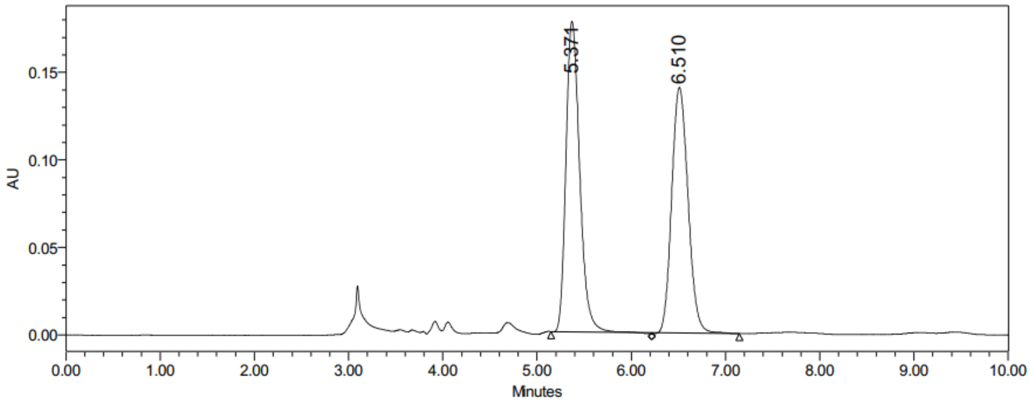

	RT	Area	% Area	Height
1	5.371	1796658	51.50	177349
2	6.510	1692153	48.50	140269

Asy-**8m**

SAMPLE INFORMATION

Sample Name:	zql-13-54-ASY-IG-5%	Acquired By:	System
Sample Type:	Unknown	Sample Set Name:	0
Vial:	33	Acq. Method Set:	5%210400
Injection #:	1	Processing Method:	1354
Injection Volume:	10.00 ul	Channel Name:	254.0nm
Run Time:	10.0 Minutes	Proc. Chnl. Descr.:	2998 PDA 254.0 nm (2998
Date Acquired:	10/10/2022 5:08:33 PM CST		
Date Processed:	10/10/2022 5:28:08 PM CST		

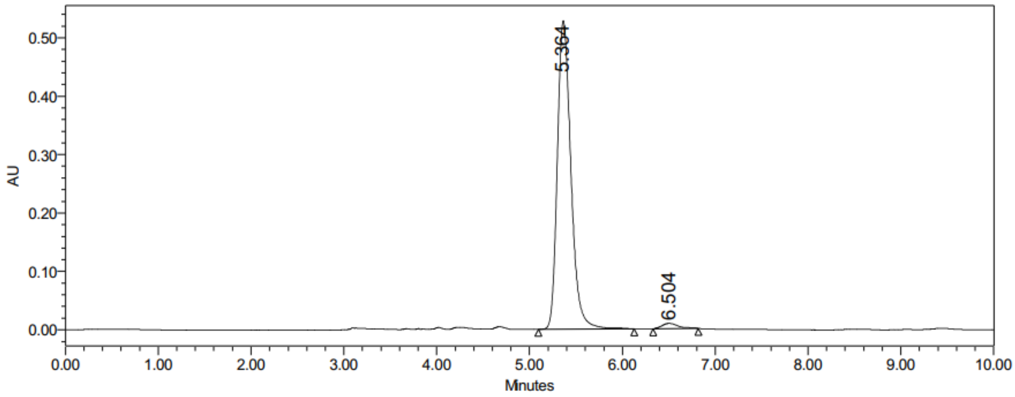

	RT	Area	% Area	Height
1	5.364	5365710	97.96	527840
2	6.504	111544	2.04	9044

Rac-**8n**

SAMPLE INFORMATION

Sample Name:	zql-14-16-rac-IC-1%0.5	Acquired By:	System
Sample Type:	Unknown	Sample Set Name:	1
Vial:	116	Acq. Method Set:	1%210400 05
Injection #:	1	Processing Method:	14162
Injection Volume:	10.00 ul	Channel Name:	250.0nm
Run Time:	18.0 Minutes	Proc. Chnl. Descr.:	2998 PDA 250.0 nm (2998
Date Acquired:	6/12/2023 8:46:20 PM CST		
Date Processed:	6/12/2023 10:25:17 PM CST		

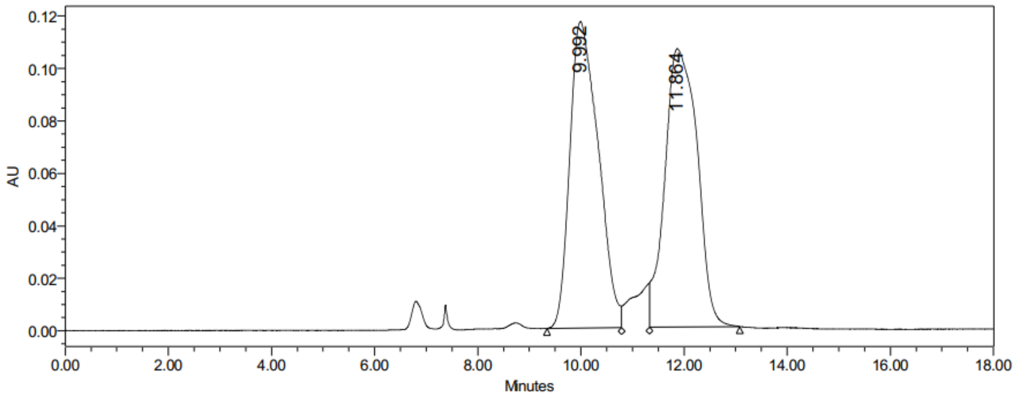

	RT	Area	% Area	Height
1	9.992	4472307	49.10	116900
2	11.864	4635513	50.90	106169

Asy-**8n**

SAMPLE INFORMATION

Sample Name:	zql-14-16-asy-IC-1%0.5	Acquired By:	System
Sample Type:	Unknown	Sample Set Name:	1
Vial:	117	Acq. Method Set:	1%210400 05
Injection #:	1	Processing Method:	1416
Injection Volume:	10.00 ul	Channel Name:	250.0nm
Run Time:	18.0 Minutes	Proc. Chnl. Descr.:	2998 PDA 250.0 nm (2998
Date Acquired:	6/12/2023 9:05:02 PM CST		
Date Processed:	6/12/2023 10:23:45 PM CST		

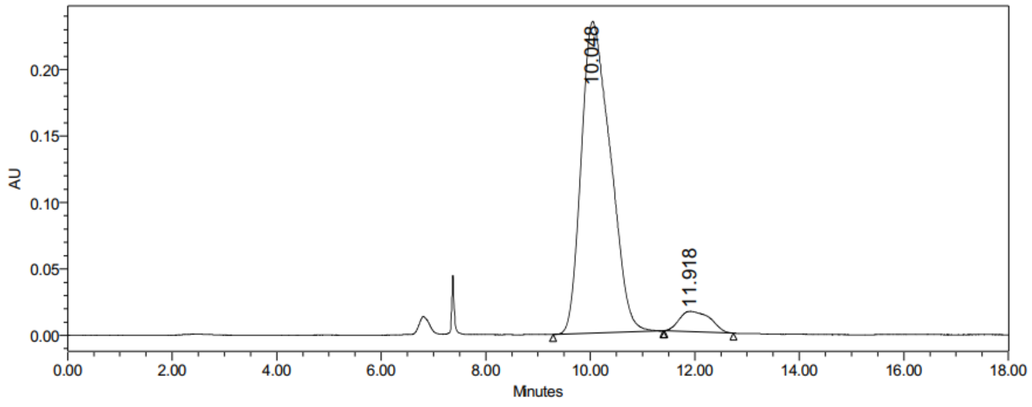

	RT	Area	% Area	Height
1	10.048	9029751	93.48	234444
2	11.918	629538	6.52	15328

Rac-**8o**

SAMPLE INFORMATION

Sample Name:	zql-12-317-RAC-10%	Acquired By:	System
Sample Type:	Unknown	Sample Set Name	
Vial:	120	Acq. Method Set:	10%210400
Injection #:	2	Processing Method	317
Injection Volume:	10.00 ul	Channel Name:	254.0nm
Run Time:	30.0 Minutes	Proc. Chnl. Descr.:	2998 PDA 254.0 nm (2998
Date Acquired:	9/7/2022 7:31:51 PM CST		
Date Processed:	9/7/2022 8:38:15 PM CST		

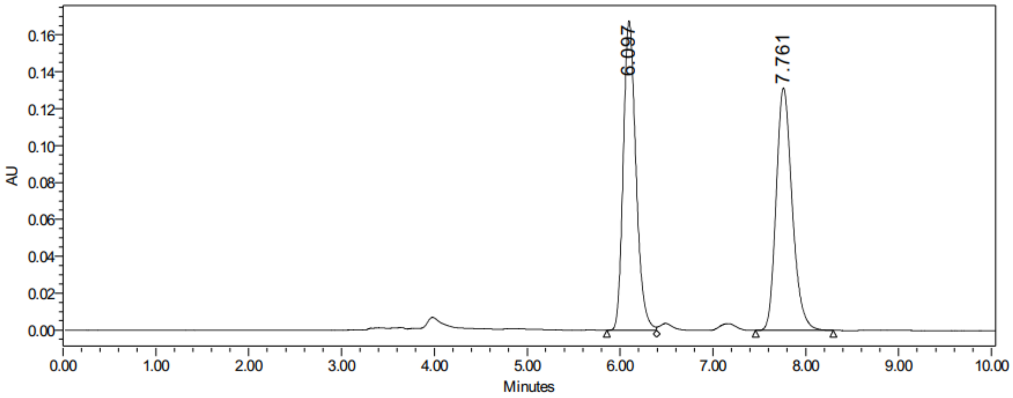

	RT	Area	% Area	Height
1	6.097	1585166	50.06	167654
2	7.761	1581172	49.94	131620

Asy-**8o**

SAMPLE INFORMATION

Sample Name:	zql-12-318-asy-10%	Acquired By:	System
Sample Type:	Unknown	Sample Set Name	
Vial:	46	Acq. Method Set:	10%210400
Injection #:	1	Processing Method	3182
Injection Volume:	20.00 ul	Channel Name:	254.0nm
Run Time:	10.0 Minutes	Proc. Chnl. Descr.:	2998 PDA 254.0 nm (2998
Date Acquired:	9/7/2022 8:27:17 PM CST		
Date Processed:	9/7/2022 8:39:28 PM CST		

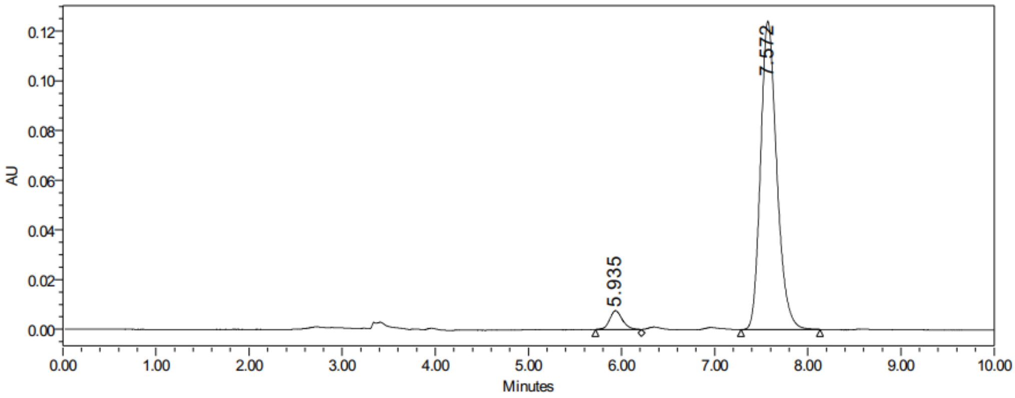

	RT	Area	% Area	Height
1	5.935	71121	4.50	7524
2	7.572	1509287	95.50	124184

Rac-**8p**

SAMPLE INFORMATION

Sample Name:	zql-12-359-rac-IG-5%	Acquired By:	System
Sample Type:	Unknown	Sample Set Name	
Vial:	2	Acq. Method Set:	5%210400
Injection #:	1	Processing Method	134
Injection Volume:	10.00 ul	Channel Name:	254.0nm
Run Time:	30.0 Minutes	Proc. Chnl. Descr.:	2998 PDA 254.0 nm (2998
Date Acquired:	9/17/2022 9:16:08 PM CST		
Date Processed:	9/23/2022 10:23:34 PM CST		

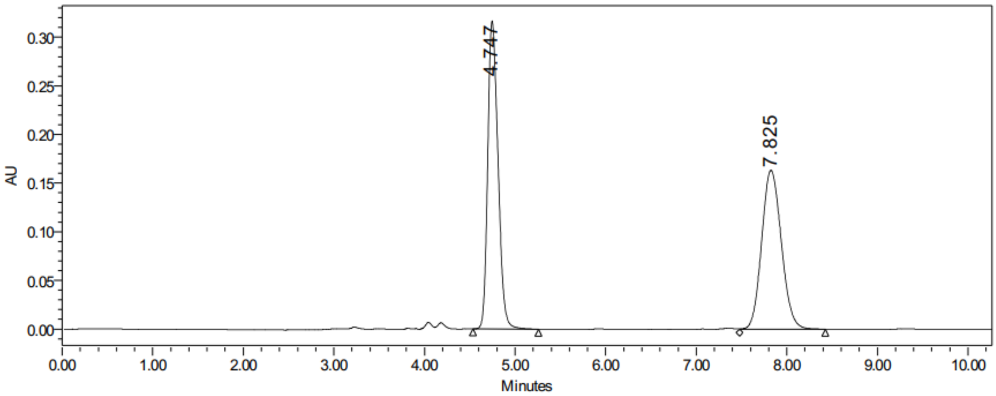

	RT	Area	% Area	Height
1	4.747	2515481	50.40	316524
2	7.825	2475584	49.60	163518

Asy-**8p**

SAMPLE INFORMATION

Sample Name:	zql-13-4-IC-5%	Acquired By:	System
Sample Type:	Unknown	Sample Set Name:	
Vial:	59	Acq. Method Set:	5%210400
Injection #:	1	Processing Method:	134
Injection Volume:	10.00 ul	Channel Name:	254.0nm
Run Time:	10.0 Minutes	Proc. Chnl. Descr.:	2998 PDA 254.0 nm (2998
Date Acquired:	9/23/2022 10:17:27 PM CST		
Date Processed:	9/23/2022 10:28:13 PM CST		

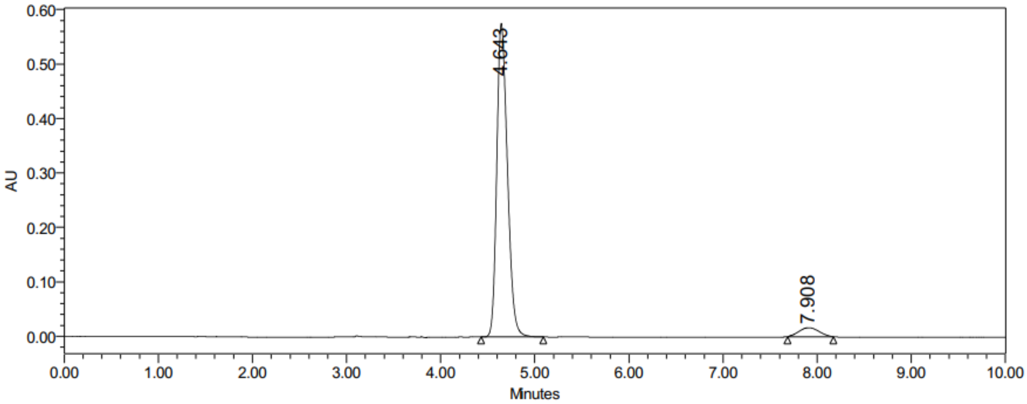

	RT	Area	% Area	Height
1	4.643	4540869	94.97	575170
2	7.908	240530	5.03	16322

Rac-**8q**

SAMPLE INFORMATION

Sample Name:	zql-13-17-RAC-IG-5%	Acquired By:	System
Sample Type:	Unknown	Sample Set Name	
Vial:	86	Acq. Method Set:	5%210400
Injection #:	1	Processing Method	1324
Injection Volume:	10.00 ul	Channel Name:	272.0nm
Run Time:	30.0 Minutes	Proc. Chnl. Descr.:	2998 PDA 272.0 nm (2998
Date Acquired:	9/30/2022 10:27:01 AM CST		
Date Processed:	9/30/2022 6:43:47 PM CST		

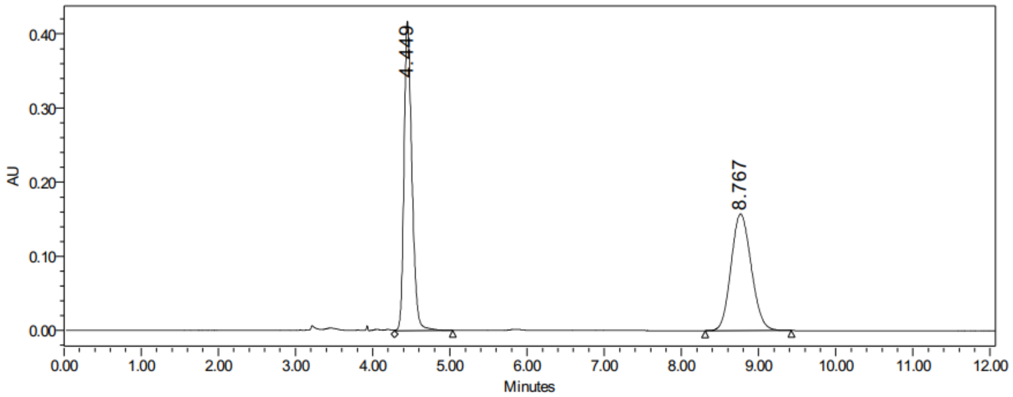

	RT	Area	% Area	Height
1	4.449	2943021	50.76	417172
2	8.767	2854639	49.24	157434

Asy-**8q**

SAMPLE INFORMATION

Sample Name:	zql-13-24-IG-5%	Acquired By:	System
Sample Type:	Unknown	Sample Set Name:	0
Vial:	51	Acq. Method Set:	5%210400
Injection #:	1	Processing Method:	1324
Injection Volume:	10.00 ul	Channel Name:	272.0nm
Run Time:	12.0 Minutes	Proc. Chnl. Descr.:	2998 PDA 272.0 nm (2998
Date Acquired:	9/30/2022 5:36:14 PM CST		
Date Processed:	9/30/2022 6:43:11 PM CST		

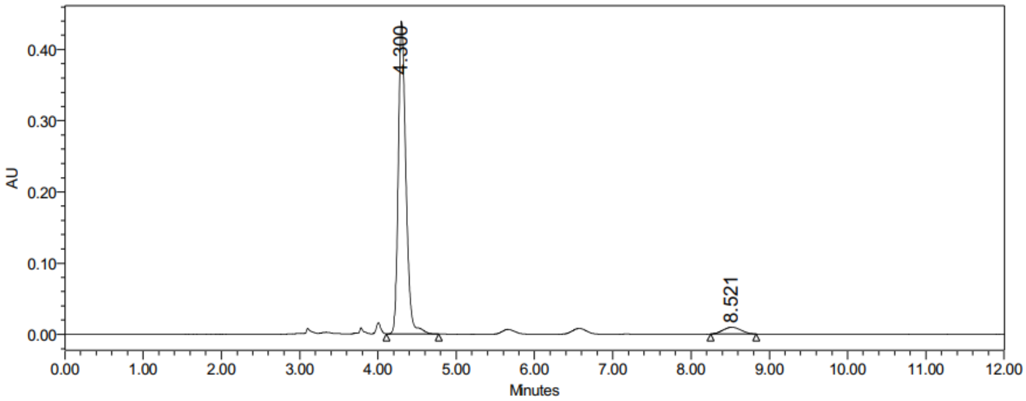

	RT	Area	% Area	Height
1	4.300	2981271	95.04	439174
2	8.521	155697	4.96	9649

Rac-**8r**

SAMPLE INFORMATION

Sample Name:	zql-13-28-rac-IG-5%	Acquired By:	System
Sample Type:	Unknown	Sample Set Name	
Vial:	13	Acq. Method Set:	5%210400
Injection #:	1	Processing Method	1329
Injection Volume:	10.00 ul	Channel Name:	254.0nm
Run Time:	30.0 Minutes	Proc. Chnl. Descr.:	2998 PDA 254.0 nm (2998
Date Acquired:	10/4/2022 9:40:11 AM CST		
Date Processed:	10/6/2022 6:58:59 PM CST		

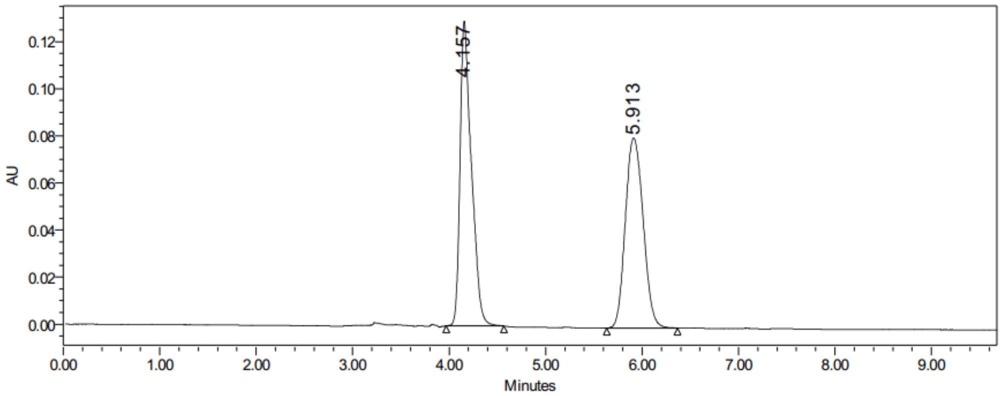

	RT	Area	% Area	Height
1	4.157	1059932	50.63	129315
2	5.913	1033559	49.37	80681

Asy-**8r**

SAMPLE INFORMATION

Sample Name:	zql-13-29-asy-IG-5%	Acquired By:	System
Sample Type:	Unknown	Sample Set Name:	0
Vial:	2	Acq. Method Set:	5%210400
Injection #:	1	Processing Method:	1329
Injection Volume:	10.00 ul	Channel Name:	254.0nm
Run Time:	10.0 Minutes	Proc. Chnl. Descr.:	2998 PDA 254.0 nm (2998
Date Acquired:	10/6/2022 12:26:29 PM CST		
Date Processed:	10/6/2022 7:01:19 PM CST		

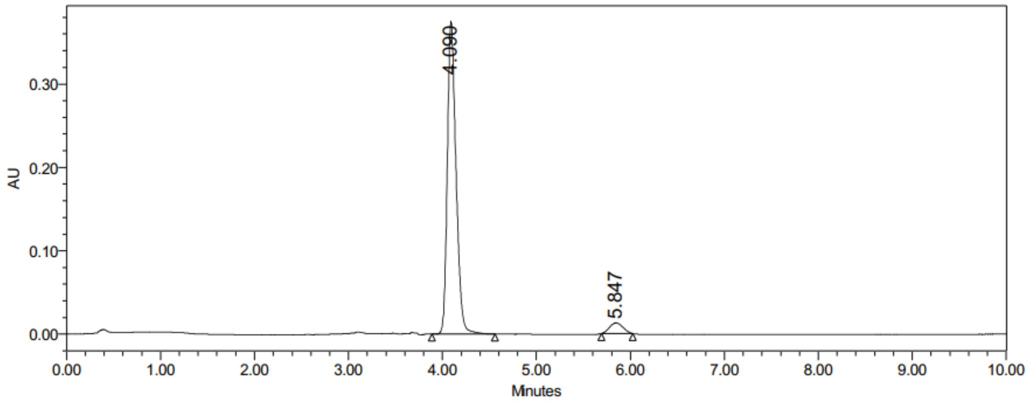

	RT	Area	% Area	Height
1	4.090	2408211	94.99	375271
2	5.847	127033	5.01	12702

Rac-**8s**

SAMPLE INFORMATION

Sample Name:	zql-12-309-rac-IG-1%	Acquired By:	System
Sample Type:	Unknown	Sample Set Name:	
Vial:	24	Acq. Method Set:	1%210400
Injection #:	1	Processing Method:	120346
Injection Volume:	10.00 ul	Channel Name:	273.0nm
Run Time:	16.0 Minutes	Proc. Chnl. Descr.:	2998 PDA 273.0 nm (2998
Date Acquired:	9/15/2022 9:29:18 AM CST		
Date Processed:	9/15/2022 9:55:02 AM CST		

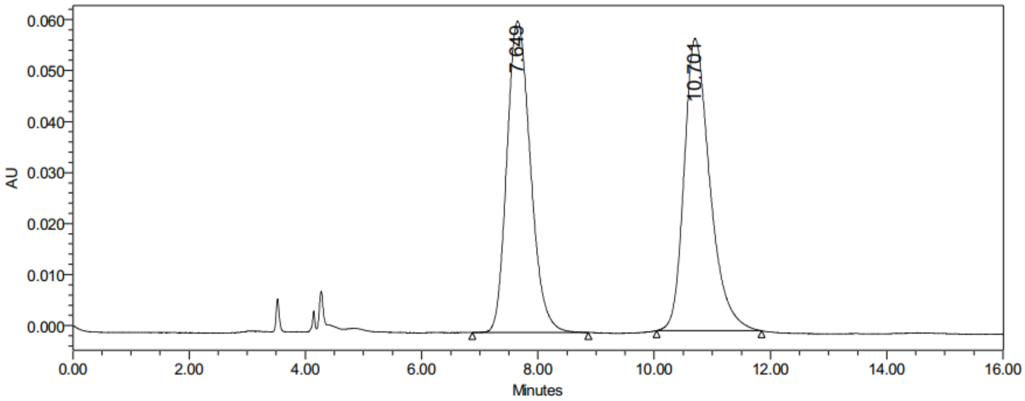

	RT	Area	% Area	Height
1	7.649	1724310	49.36	61071
2	10.701	1768768	50.64	57340

Asy-**8s**

SAMPLE INFORMATION

Sample Name:	zql-12-346-IG-1%	Acquired By:	System
Sample Type:	Unknown	Sample Set Name:	
Vial:	29	Acq. Method Set:	1%210400
Injection #:	1	Processing Method:	12 346
Injection Volume:	10.00 ul	Channel Name:	273.0nm
Run Time:	16.0 Minutes	Proc. Chnl. Descr.:	2998 PDA 273.0 nm (2998
Date Acquired:	9/15/2022 9:11:45 AM CST		
Date Processed:	9/15/2022 9:52:03 AM CST		

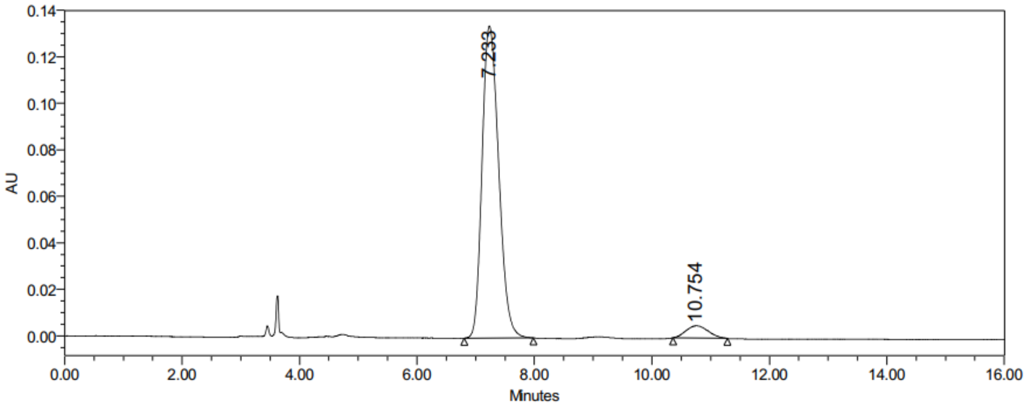

	RT	Area	% Area	Height
1	7.233	2649776	95.03	134163
2	10.754	138436	4.97	5290

Rac-**8t**

SAMPLE INFORMATION

Sample Name:	zql-13-32-rac-IA-5%	Acquired By:	System
Sample Type:	Unknown	Sample Set Name:	
Vial:	92	Acq. Method Set:	5%210400
Injection #:	1	Processing Method:	1333
Injection Volume:	10.00 ul	Channel Name:	254.0nm
Run Time:	30.0 Minutes	Proc. Chnl. Descr.:	2998 PDA 254.0 nm (2998
Date Acquired:	10/6/2022 8:03:18 PM CST		
Date Processed:	10/6/2022 9:26:59 PM CST		

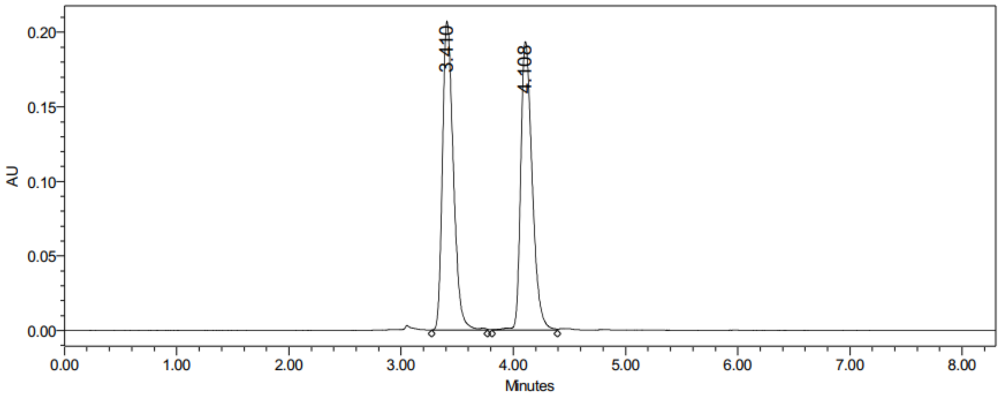

	RT	Area	% Area	Height
1	3.410	1399690	50.39	207329
2	4.108	1378123	49.61	193671

Asy-**8t**

SAMPLE INFORMATION

Sample Name:	zql-13-33-asy-IA-5%	Acquired By:	System
Sample Type:	Unknown	Sample Set Name:	
Vial:	42	Acq. Method Set:	5%210400
Injection #:	1	Processing Method:	13332
Injection Volume:	10.00 ul	Channel Name:	254.0nm
Run Time:	8.0 Minutes	Proc. Chnl. Descr.:	2998 PDA 254.0 nm (2998
Date Acquired:	10/6/2022 8:48:54 PM CST		
Date Processed:	10/6/2022 9:27:53 PM CST		

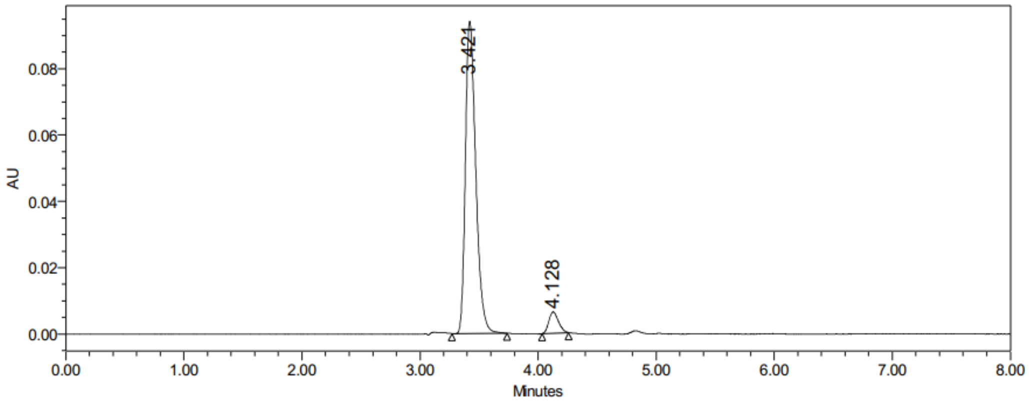

	RT	Area	% Area	Height
1	3.421	569928	94.05	94177
2	4.128	36068	5.95	6383

Rac-**8u**

SAMPLE INFORMATION

Sample Name:	zql-13-1-RAC-IA-5%	Acquired By:	System
Sample Type:	Unknown	Sample Set Name	
Vial:	80	Acq. Method Set:	5%210400
Injection #:	1	Processing Method	132
Injection Volume:	10.00 ul	Channel Name:	254.0nm
Run Time:	30.0 Minutes	Proc. Chnl. Descr.:	2998 PDA 254.0 nm (2998
Date Acquired:	9/23/2022 9:47:25 PM CST		
Date Processed:	9/23/2022 10:04:59 PM CST		

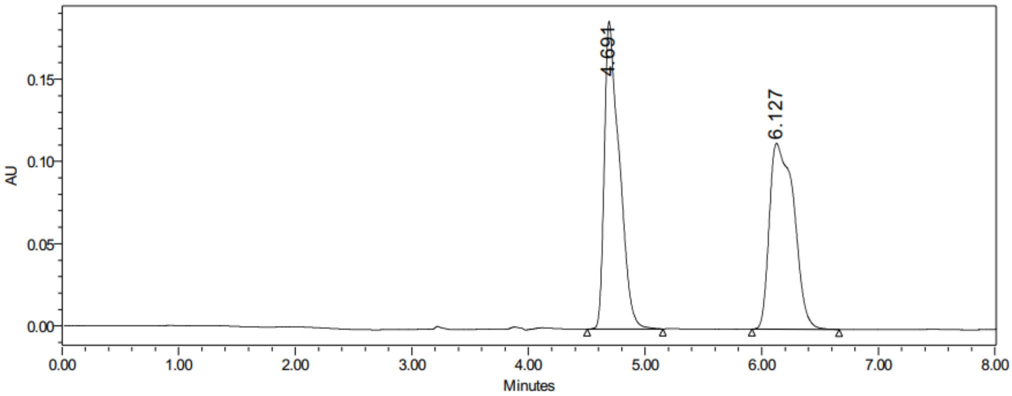

	RT	Area	% Area	Height
1	4.691	1674994	50.40	187127
2	6.127	1648386	49.60	113020

Asy-**8u**

SAMPLE INFORMATION

Sample Name:	zql-13-2-ASY-IA-5%	Acquired By:	System
Sample Type:	Unknown	Sample Set Name	
Vial:	110	Acq. Method Set:	5%210400
Injection #:	1	Processing Method	1322
Injection Volume:	10.00 ul	Channel Name:	254.0nm
Run Time:	8.0 Minutes	Proc. Chnl. Descr.:	2998 PDA 254.0 nm (2998
Date Acquired:	9/23/2022 9:56:53 PM CST		
Date Processed:	9/23/2022 10:06:22 PM CST		

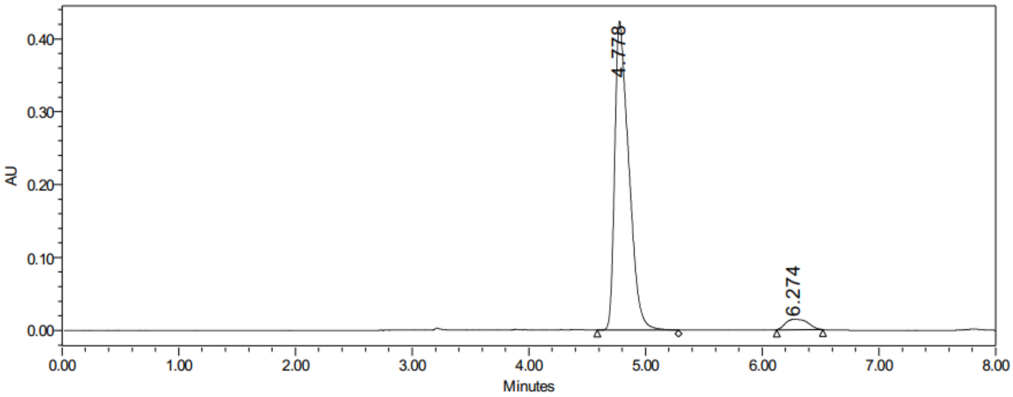

	RT	Area	% Area	Height
1	4.778	3533348	94.94	423771
2	6.274	188228	5.06	14124

Rac-**8v**

SAMPLE INFORMATION

Sample Name:	zql-13-19-rac-IG-5%	Acquired By:	System
Sample Type:	Unknown	Sample Set Name	
Vial:	110	Acq. Method Set:	5%210400
Injection #:	2	Processing Method	1325
Injection Volume:	10.00 ul	Channel Name:	254.0nm
Run Time:	10.0 Minutes	Proc. Chnl. Descr.:	2998 PDA 254.0 nm (2998
Date Acquired:	9/30/2022 9:59:35 AM CST		
Date Processed:	9/30/2022 6:44:34 PM CST		

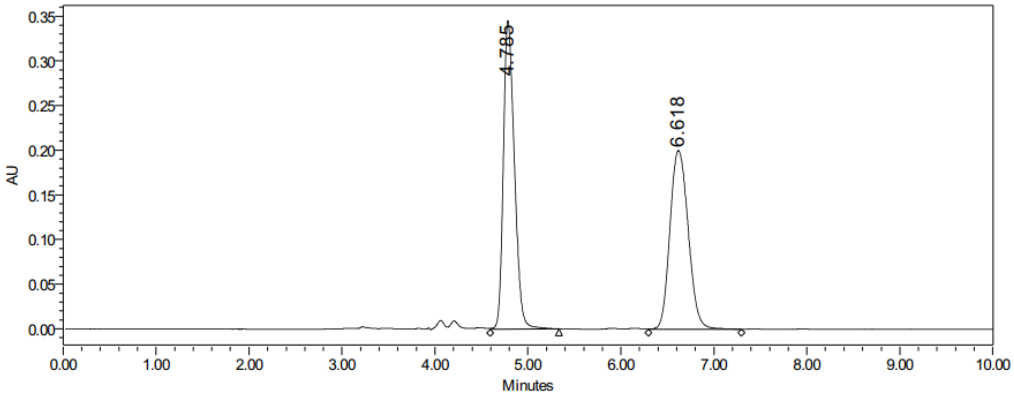

	RT	Area	% Area	Height
1	4.785	2860853	51.14	345243
2	6.618	2733321	48.86	200105

Asy-**8v**

SAMPLE INFORMATION

Sample Name:	zql-13-25-IG-5%	Acquired By:	System
Sample Type:	Unknown	Sample Set Name:	0
Vial:	52	Acq. Method Set:	5%210400
Injection #:	1	Processing Method:	1325
Injection Volume:	10.00 ul	Channel Name:	254.0nm
Run Time:	10.0 Minutes	Proc. Chnl. Descr.:	2998 PDA 254.0 nm (2998
Date Acquired:	9/30/2022 5:48:55 PM CST		
Date Processed:	9/30/2022 6:44:30 PM CST		

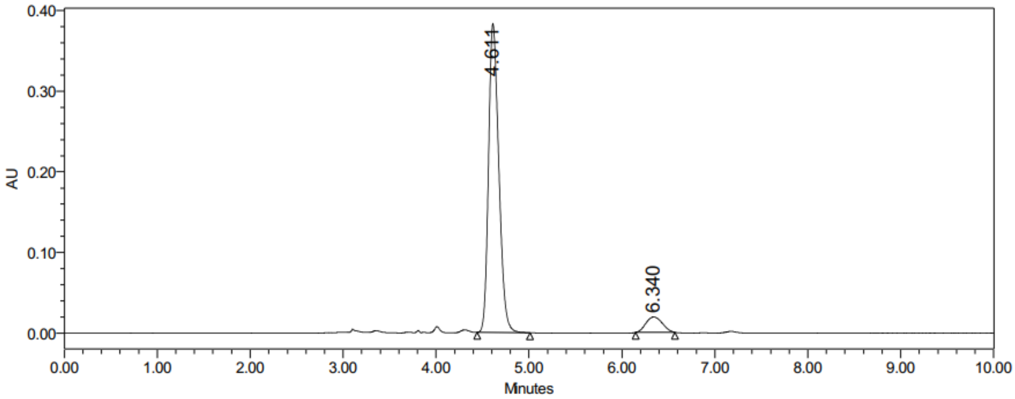

	RT	Area	% Area	Height
1	4.611	2958062	92.65	383107
2	6.340	234778	7.35	19059

Rac-**8w**

SAMPLE INFORMATION

Sample Name:	zql-17-162-RAC-IC-5%	Acquired By:	System
Sample Type:	Unknown	Sample Set Name:	0
Vial:	75	Acq. Method Set:	5%210400
Injection #:	1	Processing Method:	17149
Injection Volume:	10.00 ul	Channel Name:	254.0nm
Run Time:	10.0 Minutes	Proc. Chnl. Descr.:	2998 PDA 254.0 nm (2998
Date Acquired:	3/14/2024 10:45:39 AM CST		
Date Processed:	3/29/2024 8:02:32 PM CST		

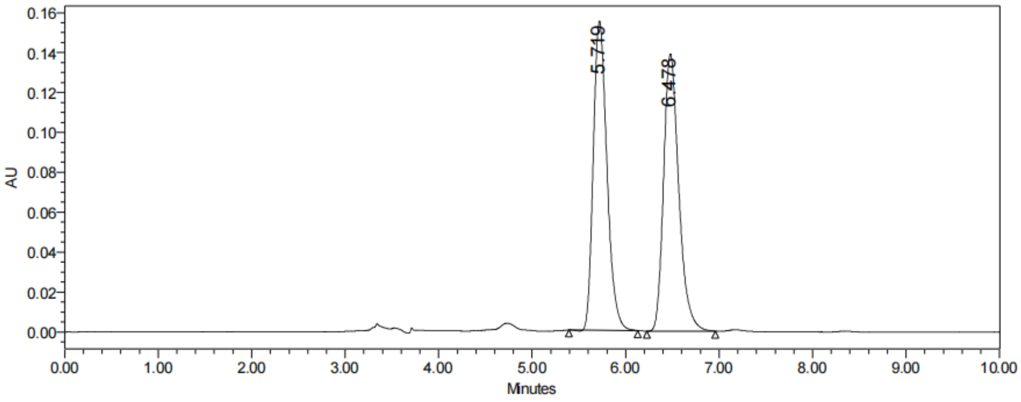

	RT	Area	% Area	Height
1	5.719	1570816	50.10	154776
2	6.478	1564385	49.90	138856

Asy-**8w**

SAMPLE INFORMATION

Sample Name:	zql-17-218-IC-5%	Acquired By:	System
Sample Type:	Unknown	Sample Set Name	0
Vial:	79	Acq. Method Set:	5%210400
Injection #:	1	Processing Method	171493
Injection Volume:	10.00 ul	Channel Name:	254.0nm
Run Time:	10.0 Minutes	Proc. Chnl. Descr.:	2998 PDA 254.0 nm (2998
Date Acquired:	4/4/2024 17:43:18 CST		
Date Processed:	4/4/2024 20:06:48 CST		

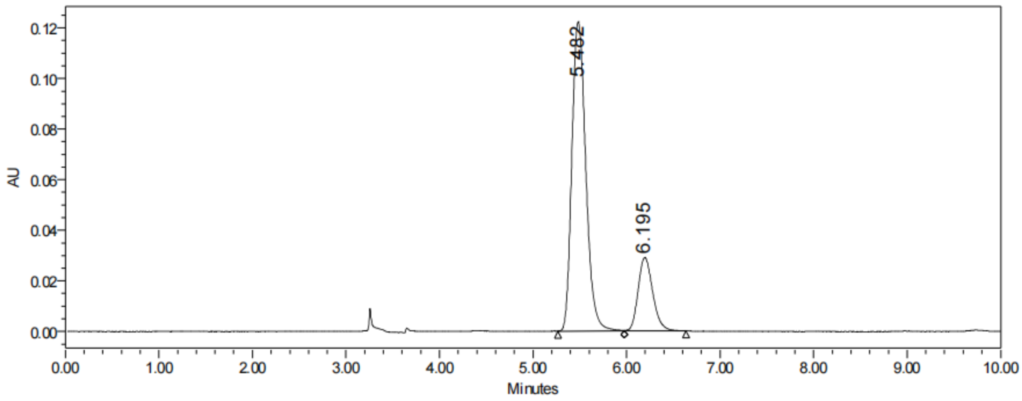

	RT	Area	% Area	Height
1	5.482	1262092	79.94	122213
2	6.195	316626	20.06	29088

Rac-**8x**

SAMPLE INFORMATION

Sample Name:	zql-17-163-RAC-IC-5%	Acquired By:	System
Sample Type:	Unknown	Sample Set Name:	0
Vial:	76	Acq. Method Set:	5%210400
Injection #:	1	Processing Method:	17150
Injection Volume:	10.00 ul	Channel Name:	254.0nm
Run Time:	10.0 Minutes	Proc. Chnl. Descr.:	2998 PDA 254.0 nm (2998
Date Acquired:	3/14/2024 10:56:22 AM CST		
Date Processed:	3/29/2024 8:04:45 PM CST		

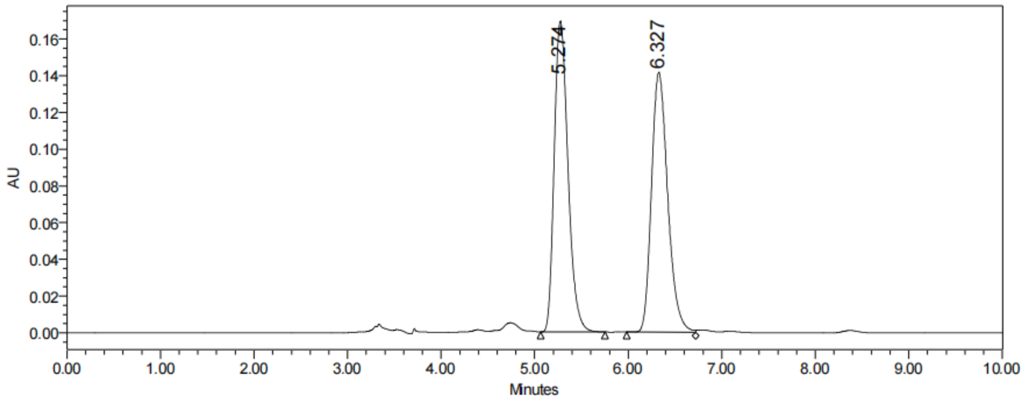

	RT	Area	% Area	Height
1	5.274	1742423	50.63	169195
2	6.327	1699355	49.37	141407

Asy-**8x**

SAMPLE INFORMATION

Sample Name:	zql-17-217-IC-5%	Acquired By:	System
Sample Type:	Unknown	Sample Set Name	0
Vial:	78	Acq. Method Set:	5%210400
Injection #:	1	Processing Method	171503
Injection Volume:	10.00 ul	Channel Name:	254.0nm
Run Time:	10.0 Minutes	Proc. Chnl. Descr.:	2998 PDA 254.0 nm (2998
Date Acquired:	4/4/2024 17:32:39 CST		
Date Processed:	4/4/2024 20:04:19 CST		

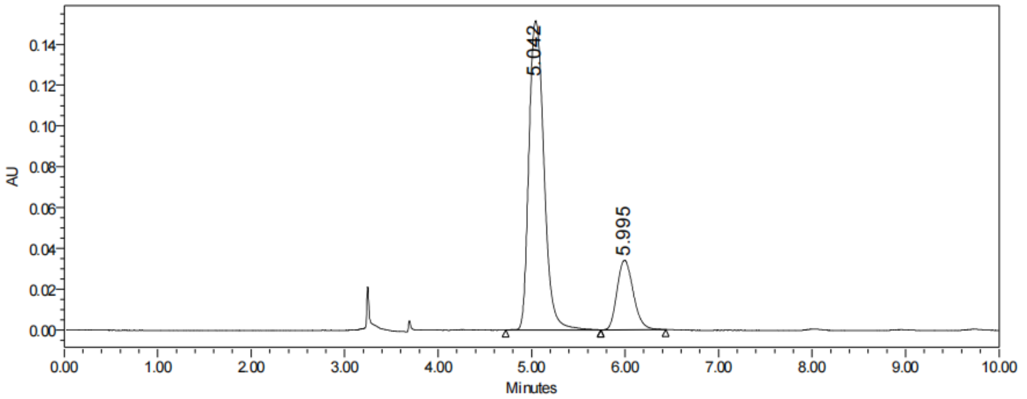

	RT	Area	% Area	Height
1	5.042	1696752	80.56	151409
2	5.995	409406	19.44	34021

Rac-**8y**

SAMPLE INFORMATION

Sample Name:	zql-12-351-RAC-IC-5%	Acquired By:	System
Sample Type:	Unknown	Sample Set Name	
Vial:	75	Acq. Method Set:	5%210400
Injection #:	1	Processing Method	12352
Injection Volume:	10.00 ul	Channel Name:	254.0nm
Run Time:	30.0 Minutes	Proc. Chnl. Descr.:	2998 PDA 254.0 nm (2998
Date Acquired:	9/15/2022 9:20:39 PM CST		
Date Processed:	9/15/2022 9:49:29 PM CST		

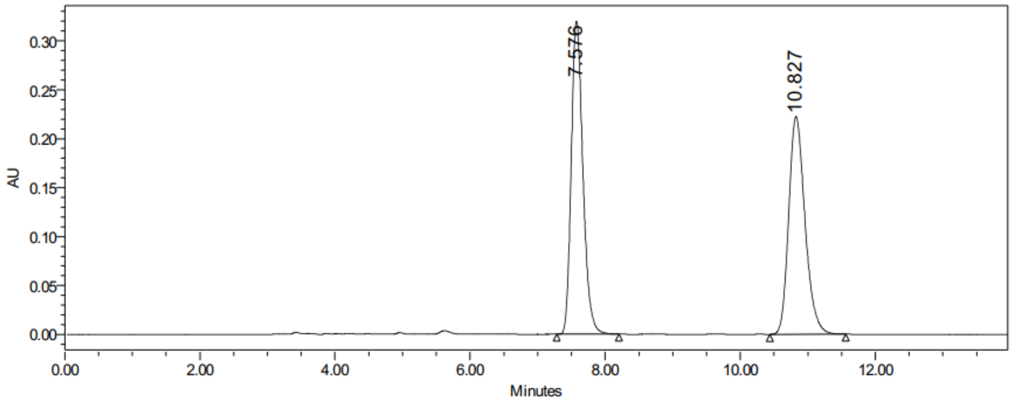

	RT	Area	% Area	Height
1	7.576	3820285	50.57	319745
2	10.827	3734074	49.43	222584

Asy-**8y**

SAMPLE INFORMATION

Sample Name:	zql-17-169-IC-5%	Acquired By:	System
Sample Type:	Unknown	Sample Set Name:	0
Vial:	93	Acq. Method Set:	5%210400
Injection #:	1	Processing Method:	8w
Injection Volume:	10.00 ul	Channel Name:	254.0nm
Run Time:	15.0 Minutes	Proc. Chnl. Descr.:	2998 PDA 254.0 nm (2998
Date Acquired:	3/16/2024 8:10:49 PM CST		
Date Processed:	4/2/2024 10:06:29 PM CST		

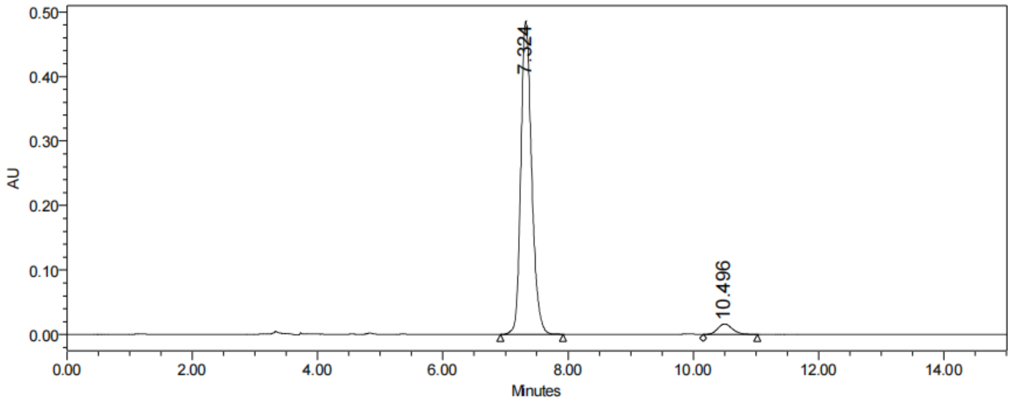

	RT	Area	% Area	Height
1	7.324	5771813	95.55	485635
2	10.496	268776	4.45	16374

Rac-**8z**

SAMPLE INFORMATION

Sample Name:	zql-12-76-RAC-IC-5%	Acquired By:	System
Sample Type:	Unknown	Sample Set Name	
Vial:	85	Acq. Method Set:	5%210400
Injection #:	1	Processing Method	12350
Injection Volume:	10.00 ul	Channel Name:	254.0nm
Run Time:	30.0 Minutes	Proc. Chnl. Descr.:	2998 PDA 254.0 nm (2998
Date Acquired:	9/15/2022 8:48:30 PM CST		
Date Processed:	9/15/2022 9:20:27 PM CST		

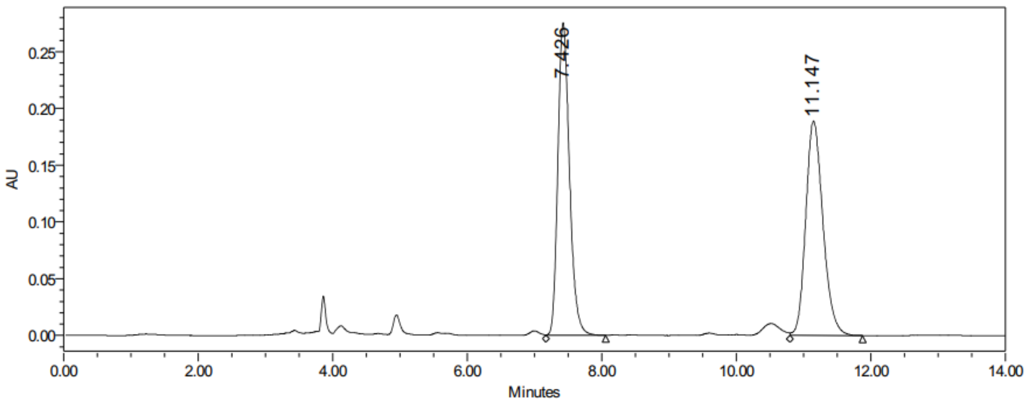

	RT	Area	% Area	Height
1	7.426	3311750	49.78	275101
2	11.147	3340930	50.22	188828

Asy-**8z**

SAMPLE INFORMATION

Sample Name:	zql-12-350-IC-5%	Acquired By:	System
Sample Type:	Unknown	Sample Set Name	
Vial:	55	Acq. Method Set:	5%210400
Injection #:	1	Processing Method	123502
Injection Volume:	10.00 ul	Channel Name:	254.0nm
Run Time:	14.0 Minutes	Proc. Chnl. Descr.:	2998 PDA 254.0 nm (2998
Date Acquired:	9/15/2022 9:04:09 PM CST		
Date Processed:	9/15/2022 9:21:36 PM CST		

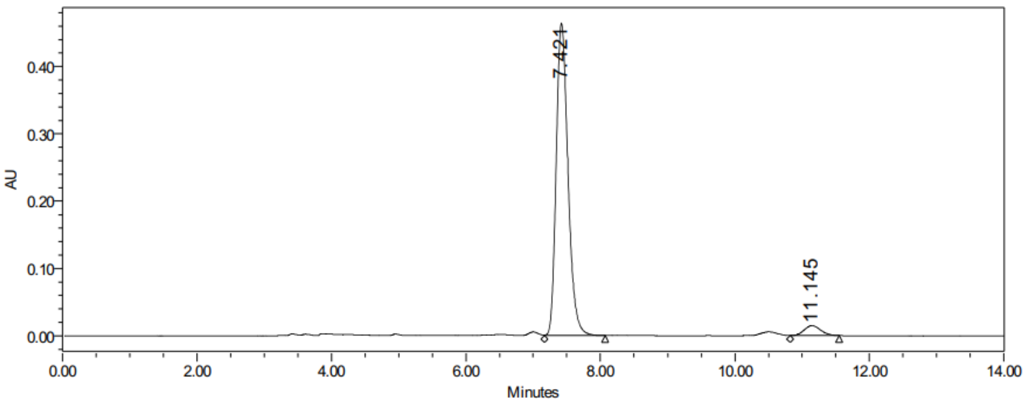

	RT	Area	% Area	Height
1	7.421	5664940	95.69	463798
2	11.145	255167	4.31	14814

Rac-**8aa**

SAMPLE INFORMATION

Sample Name:	zql-12-357-rac-IC-5%	Acquired By:	System
Sample Type:	Unknown	Sample Set Name	
Vial:	1	Acq. Method Set:	5%210400
Injection #:	1	Processing Method	12357
Injection Volume:	10.00 ul	Channel Name:	254.0nm
Run Time:	30.0 Minutes	Proc. Chnl. Descr.:	2998 PDA 254.0 nm (2998
Date Acquired:	9/17/2022 8:19:56 PM CST		
Date Processed:	9/17/2022 9:04:43 PM CST		

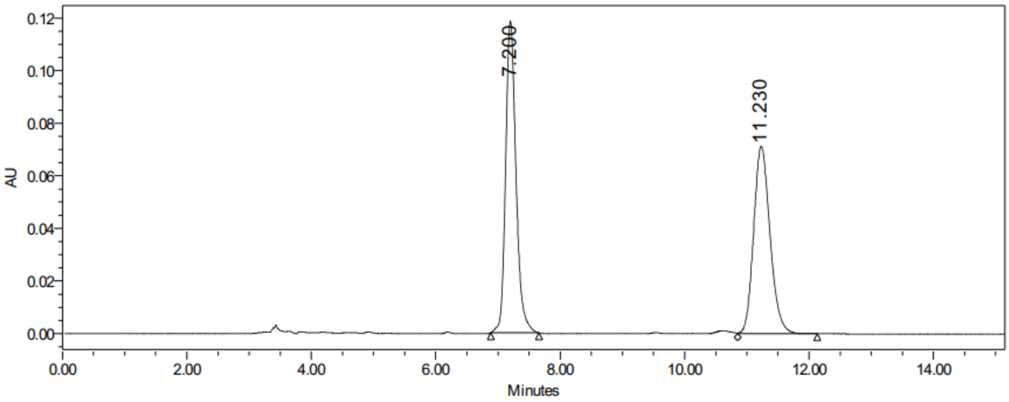

	RT	Area	% Area	Height
1	7.200	1370886	51.33	118435
2	11.230	1300084	48.67	71355

Asy-**8aa**

SAMPLE INFORMATION

Sample Name:	zql-12-358-asy-IC-5%	Acquired By:	System
Sample Type:	Unknown	Sample Set Name	
Vial:	43	Acq. Method Set:	5%210400
Injection #:	1	Processing Method	12358
Injection Volume:	10.00 ul	Channel Name:	254.0nm
Run Time:	15.0 Minutes	Proc. Chnl. Descr.:	2998 PDA 254.0 nm (2998
Date Acquired:	9/17/2022 8:46:10 PM CST		
Date Processed:	9/17/2022 9:05:47 PM CST		

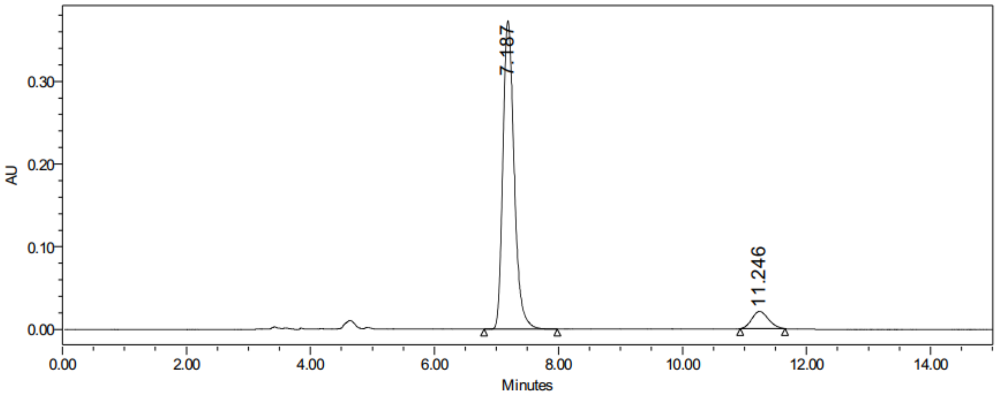

	RT	Area	% Area	Height
1	7.187	4709291	92.39	372783
2	11.246	387914	7.61	20854

Rac-**9**

SAMPLE INFORMATION

Sample Name:	zql-14-175-CHO-RAC-IC-5%	Acquired By:	System
Sample Type:	Unknown	Sample Set Name	0
Vial:	17	Acq. Method Set:	5%210400
Injection #:	1	Processing Method	1417523
Injection Volume:	10.00 ul	Channel Name:	254.0nm
Run Time:	9.0 Minutes	Proc. Chnl. Descr.:	2998 PDA 254.0 nm (2998
Date Acquired:	3/23/2023 16:56:07 CST		
Date Processed:	3/23/2023 19:00:04 CST		

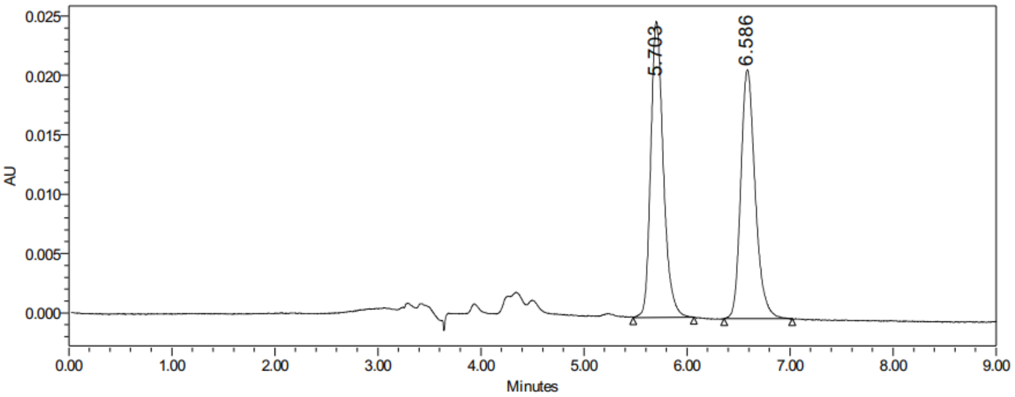

	RT	Area	% Area	Height
1	5.703	211352	50.89	24917
2	6.586	203945	49.11	20986

Asy-**9**

SAMPLE INFORMATION

Sample Name:	zql-14-175-CHO-ASY-IC-5%	Acquired By:	System
Sample Type:	Unknown	Sample Set Name	0
Vial:	18	Acq. Method Set:	5%210400
Injection #:	1	Processing Method	14175cho
Injection Volume:	10.00 ul	Channel Name:	254.0nm
Run Time:	9.0 Minutes	Proc. Chnl. Descr.:	2998 PDA 254.0 nm (2998
Date Acquired:	3/23/2023 17:05:47 CST		
Date Processed:	3/23/2023 19:00:35 CST		

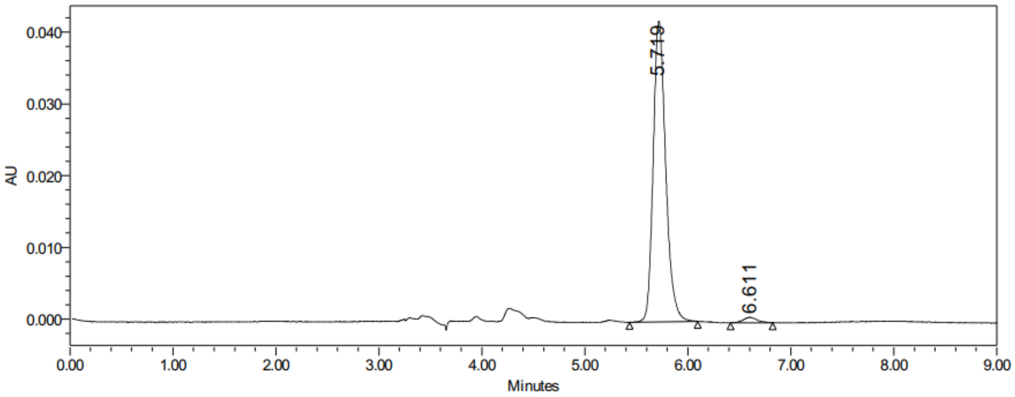

	RT	Area	% Area	Height
1	5.719	352701	98.18	41914
2	6.611	6520	1.82	714

Rac-**10**

SAMPLE INFORMATION

Sample Name:	zql-14-169-IC-RAC-10%	Acquired By:	System
Sample Type:	Unknown	Sample Set Name	
Vial:	62	Acq. Method Set:	10%210400
Injection #:	2	Processing Method	141751
Injection Volume:	10.00 ul	Channel Name:	254.0nm
Run Time:	30.0 Minutes	Proc. Chnl. Descr.:	2998 PDA 254.0 nm (2998
Date Acquired:	3/9/2023 16:22:25 CST		
Date Processed:	3/9/2023 19:35:49 CST		

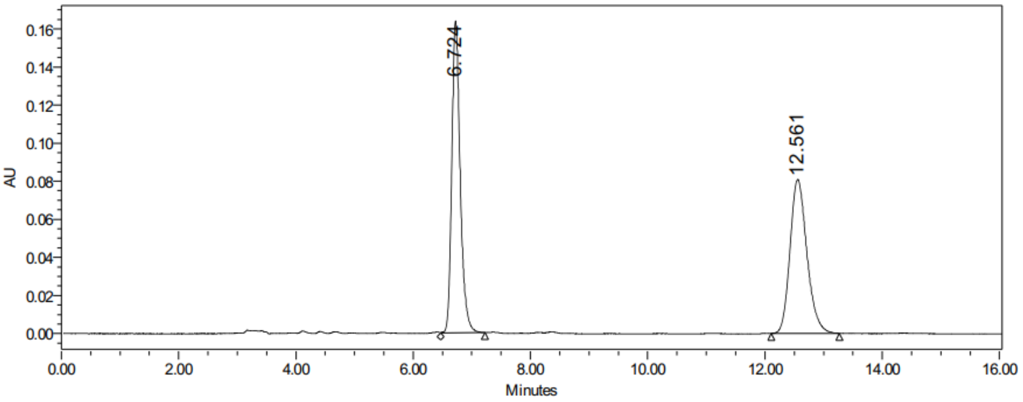

	RT	Area	% Area	Height
1	6.724	1628999	50.25	163700
2	12.561	1612855	49.75	80802

Asy-**10**

SAMPLE INFORMATION

Sample Name:	zql-14-175-asy-IC-10%	Acquired By:	System
Sample Type:	Unknown	Sample Set Name	0
Vial:	86	Acq. Method Set:	10%210400
Injection #:	1	Processing Method	141752
Injection Volume:	10.00 ul	Channel Name:	254.0nm
Run Time:	16.0 Minutes	Proc. Chnl. Descr.:	2998 PDA 254.0 nm (2998
Date Acquired:	3/9/2023 16:40:56 CST		
Date Processed:	3/9/2023 19:34:57 CST		

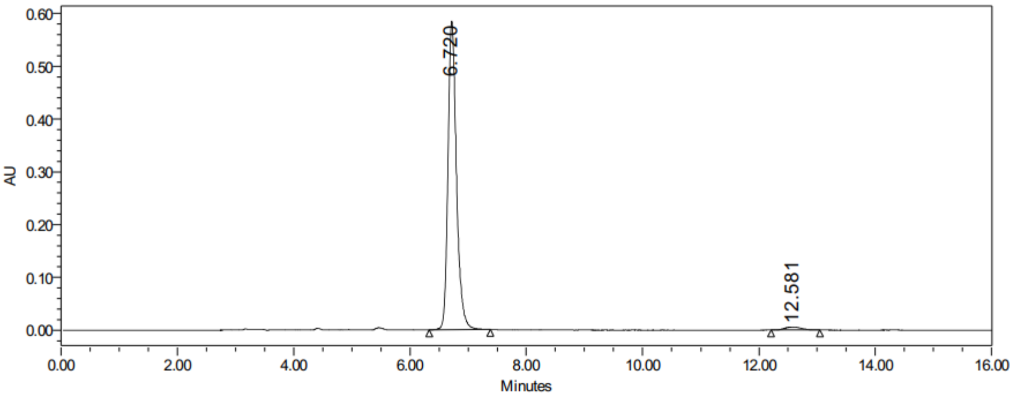

	RT	Area	% Area	Height
1	6.720	5899095	97.99	584360
2	12.581	120797	2.01	6148

Rac-**11**

SAMPLE INFORMATION

Sample Name:	zql-14-222-OMs-rac-IC-30%	Acquired By:	System
Sample Type:	Unknown	Sample Set Name	
Vial:	13	Acq. Method Set:	30%254400
Injection #:	1	Processing Method	142321
Injection Volume:	10.00 ul	Channel Name:	254.0nm
Run Time:	30.0 Minutes	Proc. Chnl. Descr.:	2998 PDA 254.0 nm (2998
Date Acquired:	4/7/2023 9:47:48 CST		
Date Processed:	4/7/2023 10:52:37 CST		

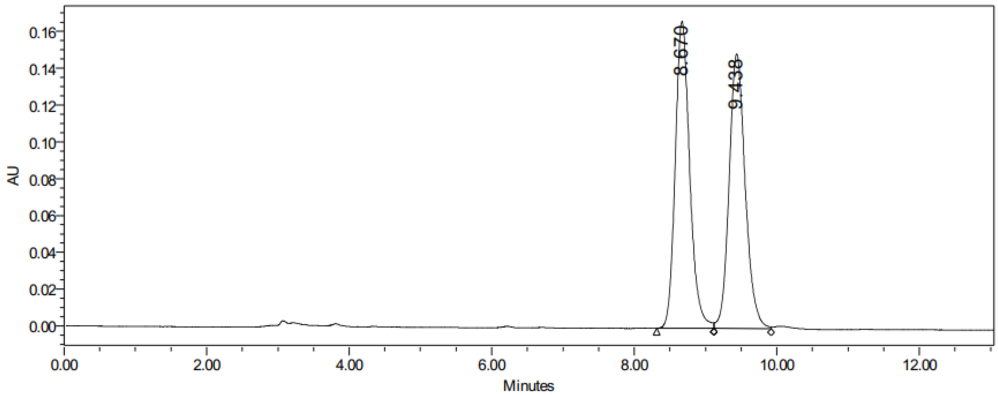

	RT	Area	% Area	Height
1	8.670	2349896	50.21	166796
2	9.438	2330017	49.79	149174

Asy-**11**

SAMPLE INFORMATION

Sample Name:	zql-14-243-2-IC-30%	Acquired By:	System
Sample Type:	Unknown	Sample Set Name	0
Vial:	117	Acq. Method Set:	30%254400
Injection #:	1	Processing Method	14243
Injection Volume:	10.00 ul	Channel Name:	254.0nm
Run Time:	12.5 Minutes	Proc. Chnl. Descr.:	2998 PDA 254.0 nm (2998
Date Acquired:	4/15/2023 12:16:46 CST		
Date Processed:	4/15/2023 15:05:04 CST		

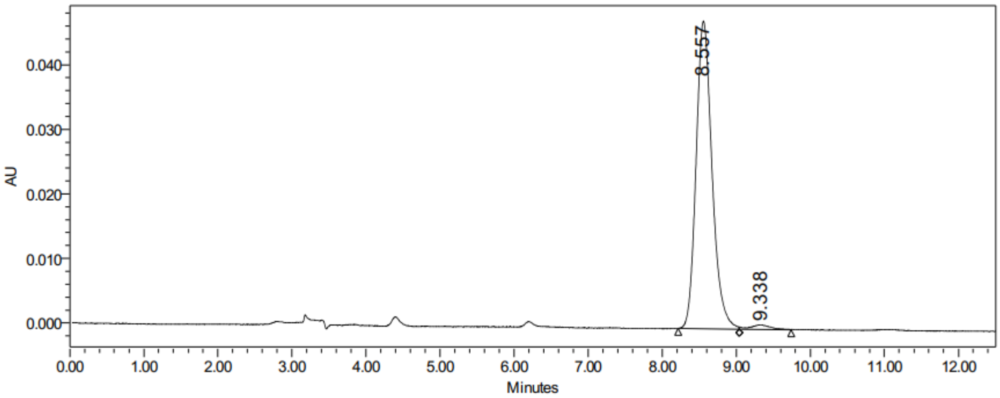

	RT	Area	% Area	Height
1	8.557	711542	98.09	47651
2	9.338	13881	1.91	682

Rac-**12**

SAMPLE INFORMATION

Sample Name:		Acquired By:	System
Sample Type:	Unknown	Sample Set Name	0
Vial:	39	Acq. Method Set:	30%254400
Injection #:	1	Processing Method	142451
Injection Volume:	10.00 ul	Channel Name:	254.0nm
Run Time:	13.0 Minutes	Proc. Chnl. Descr.:	2998 PDA 254.0 nm (2998
Date Acquired:	4/7/2023 12:01:17 CST		
Date Processed:	4/15/2023 19:36:46 CST		

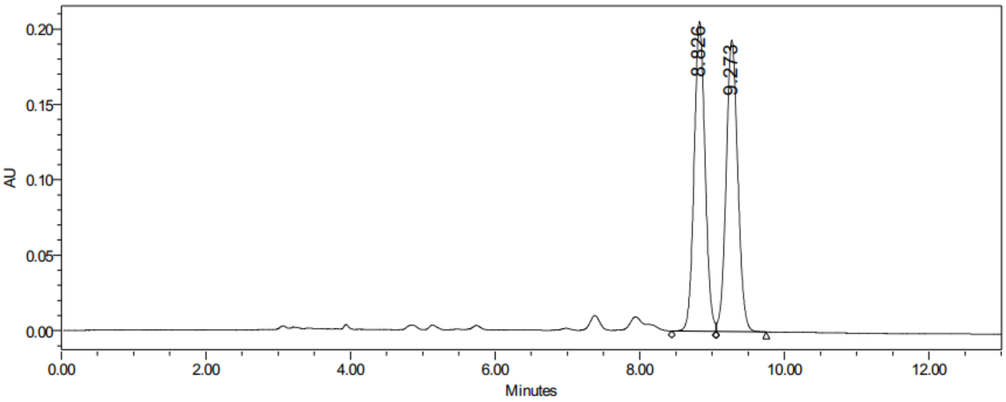

	RT	Area	% Area	Height
1	8.826	2147151	49.76	205503
2	9.273	2168070	50.24	193348

Asy-**12**

SAMPLE INFORMATION

Sample Name:		Acquired By:	System
Sample Type:	Unknown	Sample Set Name	
Vial:	4	Acq. Method Set:	30%254400
Injection #:	1	Processing Method	142453
Injection Volume:	10.00 ul	Channel Name:	254.0nm
Run Time:	13.0 Minutes	Proc. Chnl. Descr.:	2998 PDA 254.0 nm (2998
Date Acquired:	4/15/2023 19:26:16 CST		
Date Processed:	4/15/2023 19:47:18 CST		

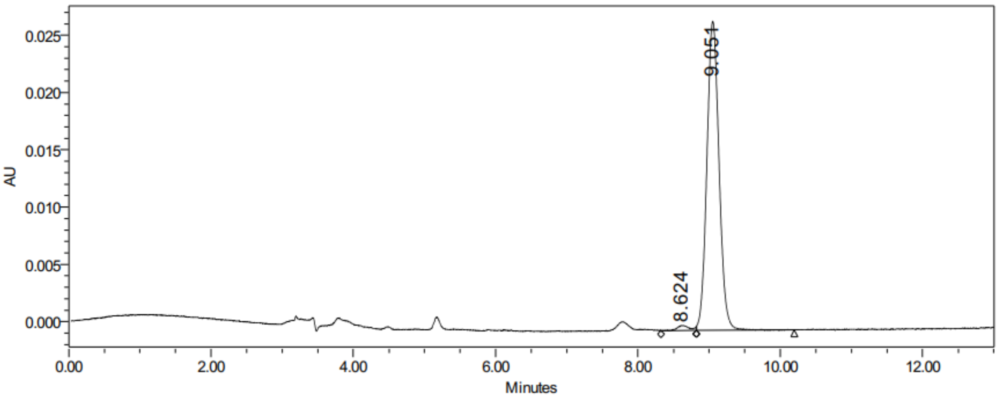

	RT	Area	% Area	Height
1	8.624	5692	1.75	450
2	9.051	319984	98.25	26956

Rac-**13**

SAMPLE INFORMATION

Sample Name:	zql-14-272-1-RAC-IC-30%	Acquired By:	System
Sample Type:	Unknown	Sample Set Name:	
Vial:	83	Acq. Method Set:	30%210400
Injection #:	1	Processing Method:	142721
Injection Volume:	10.00 ul	Channel Name:	254.0nm
Run Time:	12.0 Minutes	Proc. Chnl. Descr.:	2998 PDA 254.0 nm (2998
Date Acquired:	4/21/2023 11:39:53 PM CST		
Date Processed:	4/21/2023 11:52:27 PM CST		

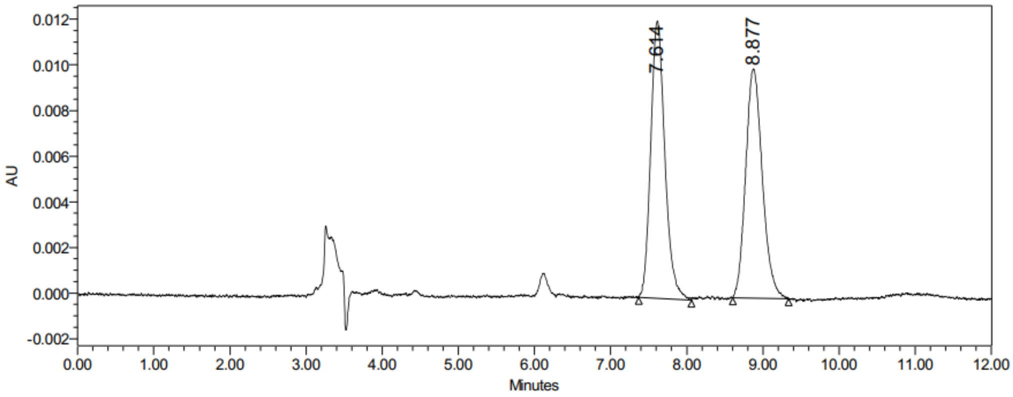

	RT	Area	% Area	Height
1	7.614	155651	50.39	12141
2	8.877	153249	49.61	10037

Asy-**13**

SAMPLE INFORMATION

Sample Name:	zql-14-272-2-ASY-IC-30%	Acquired By:	System
Sample Type:	Unknown	Sample Set Name:	
Vial:	112	Acq. Method Set:	30%210400
Injection #:	1	Processing Method:	142722
Injection Volume:	10.00 ul	Channel Name:	254.0nm
Run Time:	14.0 Minutes	Proc. Chnl. Descr.:	2998 PDA 254.0 nm (2998
Date Acquired:	4/21/2023 11:26:32 PM CST		
Date Processed:	4/21/2023 11:40:18 PM CST		

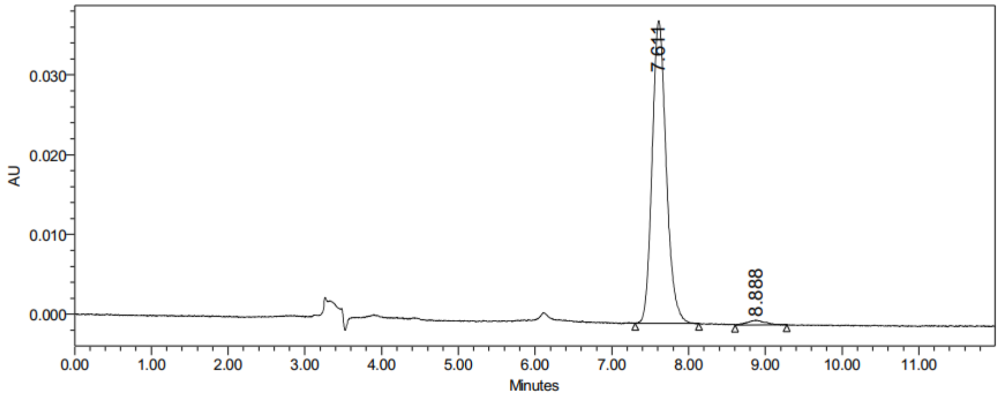

	RT	Area	% Area	Height
1	7.611	482136	98.02	37896
2	8.888	9762	1.98	602

Rac-**14**

SAMPLE INFORMATION

Sample Name:	zql-15-156-1-rac-IG-5%	Acquired By:	System
Sample Type:	Unknown	Sample Set Name:	0
Vial:	71	Acq. Method Set:	5%210400
Injection #:	1	Processing Method:	151572
Injection Volume:	10.00 ul	Channel Name:	300.0nm
Run Time:	12.0 Minutes	Proc. Chnl. Descr.:	2998 PDA 300.0 nm (2998
Date Acquired:	6/17/2023 9:47:07 PM CST		
Date Processed:	6/17/2023 10:14:57 PM CST		

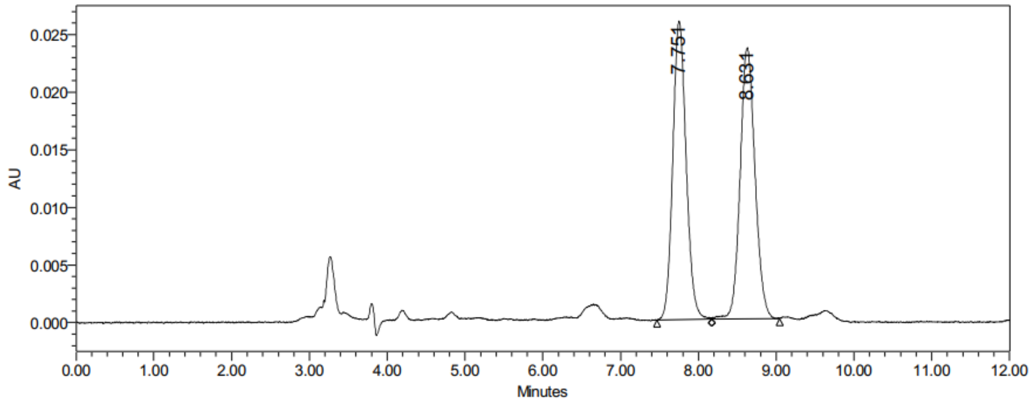

	RT	Area	% Area	Height
1	7.751	309403	49.61	25914
2	8.631	314224	50.39	23563

Asy-**14**

SAMPLE INFORMATION

Sample Name:	zql-15-157-1-CHO-IG-5%	Acquired By:	System
Sample Type:	Unknown	Sample Set Name:	0
Vial:	72	Acq. Method Set:	5%210400
Injection #:	1	Processing Method:	15157
Injection Volume:	10.00 ul	Channel Name:	300.0nm
Run Time:	12.0 Minutes	Proc. Chnl. Descr.:	2998 PDA 300.0 nm (2998
Date Acquired:	6/17/2023 9:59:46 PM CST		
Date Processed:	6/17/2023 10:16:07 PM CST		

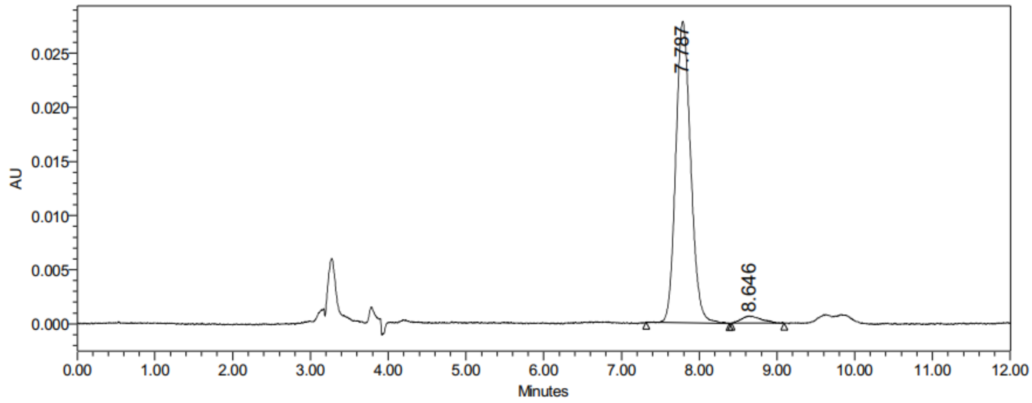

	RT	Area	% Area	Height
1	7.787	371678	97.12	27847
2	8.646	11018	2.88	639

Rac-**15**

S A M P L E   I N F O R M A T I O N

Sample Name:	zql-15-156-2-CN-RAC-IA-5%	Acquired By:	System
Sample Type:	Unknown	Sample Set Name:	
Vial:	39	Acq. Method Set:	5%210400
Injection #:	4	Processing Method:	151562
Injection Volume:	10.00 ul	Channel Name:	285.0nm
Run Time:	9.0 Minutes	Proc. Chnl. Descr.:	2998 PDA 285.0 nm (2998
Date Acquired:	6/19/2023 9:02:25 PM CST		
Date Processed:	6/20/2023 2:44:19 PM CST		

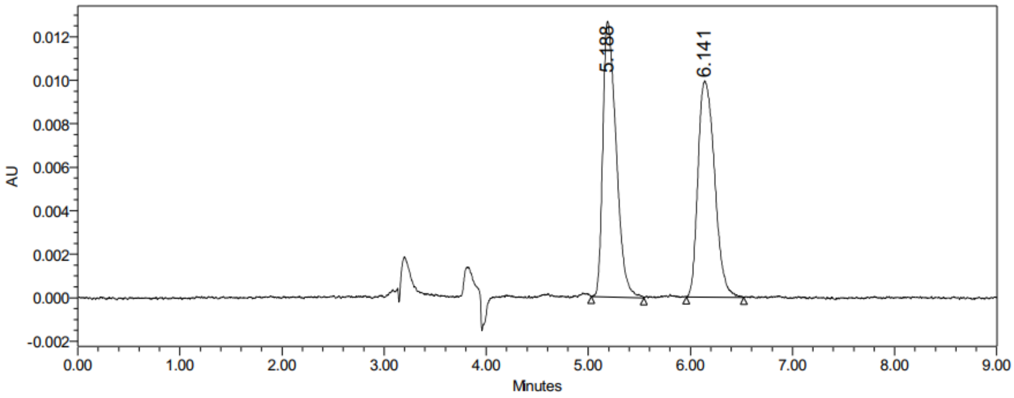

	RT	Area	% Area	Height
1	5.188	114743	50.43	12676
2	6.141	112767	49.57	9939

Asy-**15**

S A M P L E   I N F O R M A T I O N

Sample Name:	zql-15-157-2-CN-ASY-IA-5%	Acquired By:	System
Sample Type:	Unknown	Sample Set Name:	0
Vial:	92	Acq. Method Set:	5%210400
Injection #:	1	Processing Method:	151572
Injection Volume:	10.00 ul	Channel Name:	285.0nm
Run Time:	9.0 Minutes	Proc. Chnl. Descr.:	2998 PDA 285.0 nm (2998
Date Acquired:	6/20/2023 11:58:11 AM CST		
Date Processed:	6/20/2023 2:42:57 PM CST		

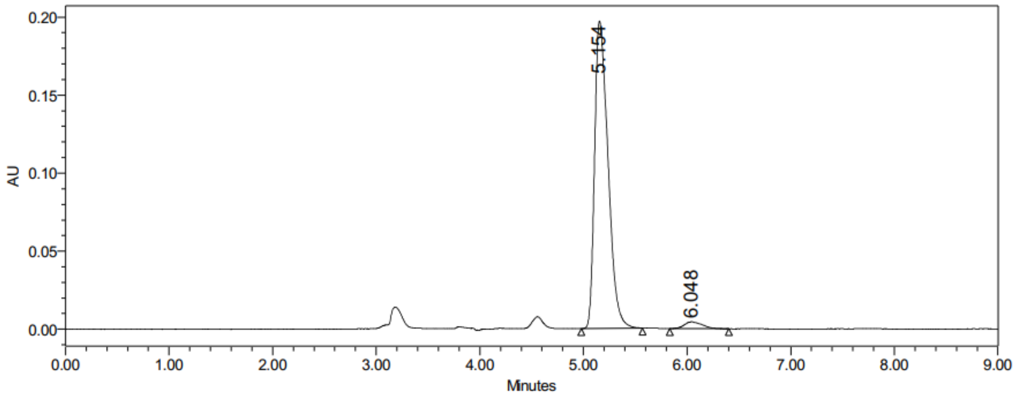

	RT	Area	% Area	Height
1	5.154	1763330	97.14	196956
2	6.048	51869	2.86	4391

Rac-**17**

SAMPLE INFORMATION

Sample Name:	zql-14-216-1-CHO-RAC-IC-5%	Acquired By:	System
Sample Type:	Unknown	Sample Set Name:	0
Vial:	92	Acq. Method Set:	5%210400
Injection #:	1	Processing Method:	142201
Injection Volume:	10.00 ul	Channel Name:	254.0nm
Run Time:	10.0 Minutes	Proc. Chnl. Descr.:	2998 PDA 254.0 nm (2998
Date Acquired:	3/28/2023 11:12:00 AM CST		
Date Processed:	3/28/2023 2:45:59 PM CST		

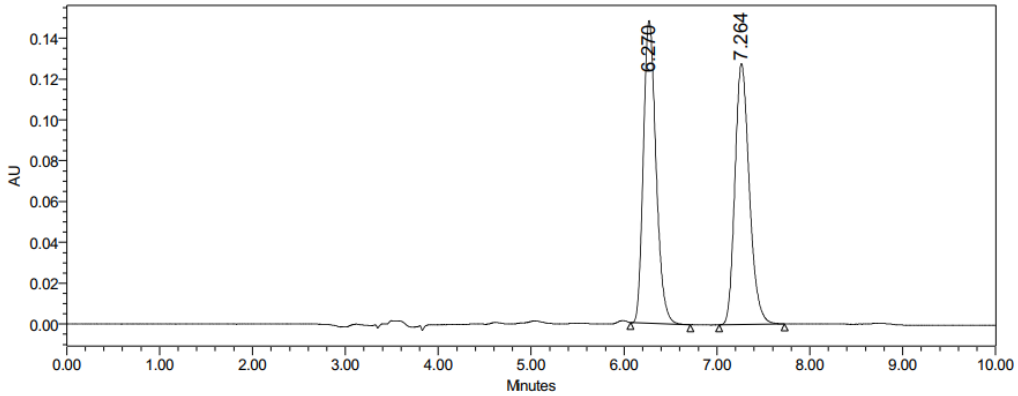

	RT	Area	% Area	Height
1	6.270	1402299	50.49	148265
2	7.264	1375169	49.51	127849

Asy-**17**

SAMPLE INFORMATION

Sample Name:	zql-14-220-CHO-ASY-IC-5%	Acquired By:	System
Sample Type:	Unknown	Sample Set Name:	0
Vial:	93	Acq. Method Set:	5%210400
Injection #:	1	Processing Method:	142202
Injection Volume:	10.00 ul	Channel Name:	254.0nm
Run Time:	10.0 Minutes	Proc. Chnl. Descr.:	2998 PDA 254.0 nm (2998
Date Acquired:	3/28/2023 11:22:43 AM CST		
Date Processed:	3/28/2023 2:47:02 PM CST		

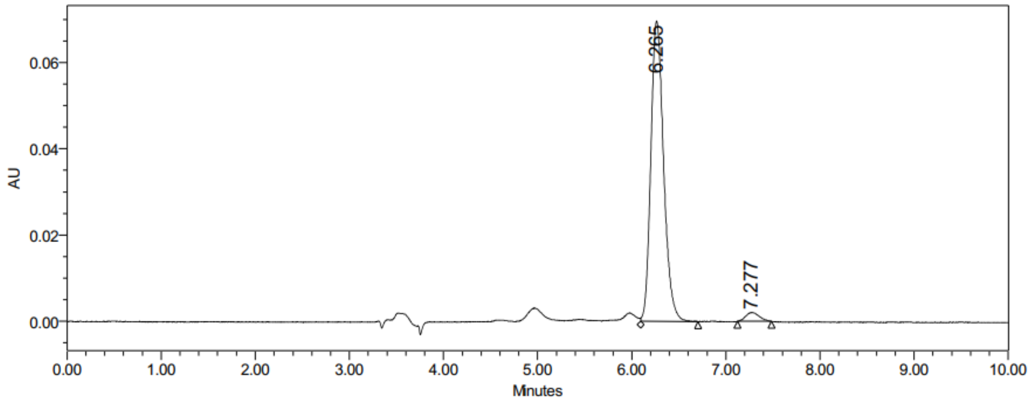

	RT	Area	% Area	Height
1	6.265	655626	96.95	69624
2	7.277	20600	3.05	2065

Rac-**18**

SAMPLE INFORMATION

Sample Name:	zql-15-158-CN-rac-IG-5%	Acquired By:	System
Sample Type:	Unknown	Sample Set Name:	
Vial:	43	Acq. Method Set:	5%210400
Injection #:	1	Processing Method:	151582
Injection Volume:	10.00 ul	Channel Name:	254.0nm
Run Time:	30.0 Minutes	Proc. Chnl. Descr.:	2998 PDA 254.0 nm (2998
Date Acquired:	6/21/2023 10:49:33 AM CST		
Date Processed:	6/28/2023 10:04:16 PM CST		

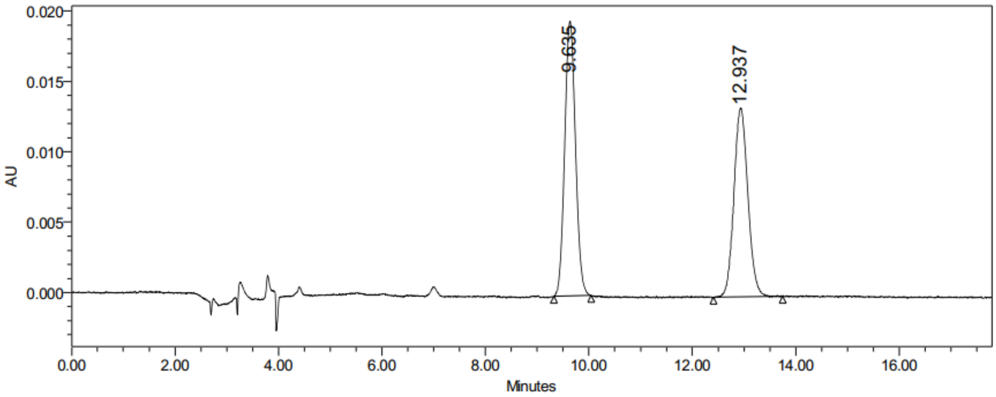

	RT	Area	% Area	Height
1	9.635	281629	52.73	19523
2	12.937	252440	47.27	13408

Asy-**18**

SAMPLE INFORMATION

Sample Name:	zql-15-158-CN-asy	Acquired By:	System
Sample Type:	Unknown	Sample Set Name:	a
Vial:	56	Acq. Method Set:	5%210400
Injection #:	1	Processing Method:	151588
Injection Volume:	10.00 ul	Channel Name:	254.0nm
Run Time:	20.0 Minutes	Proc. Chnl. Descr.:	2998 PDA 254.0 nm (2998
Date Acquired:	6/28/2023 9:22:44 PM CST		
Date Processed:	6/28/2023 10:03:02 PM CST		

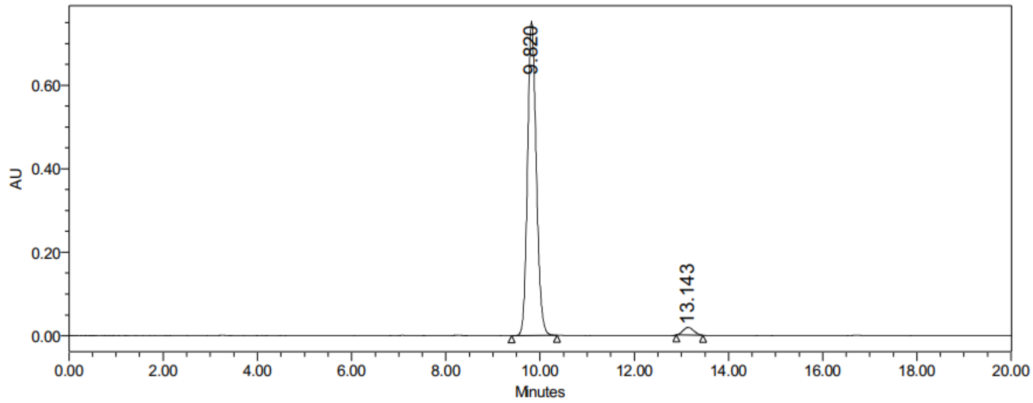

	RT	Area	% Area	Height
1	9.820	9618458	97.01	752398
2	13.143	296027	2.99	18599

14. References

1. Woll, M. G. et al. Discovery and optimization of small molecule splicing modifiers of survival motor neuron 2 as a treatment for spinal muscular atrophy. *J. Med. Chem.* **59**, 6070–6085 (2016).
2. He, Z.-T., Jiang, X. & Hartwig, J. F. Stereodivergent construction of tertiary fluorides in vicinal stereogenic pairs by allylic substitution with iridium and copper catalysts. *J. Am. Chem. Soc.* **141**, 13066–13073 (2019).
3. Zhang, J., Zhu, W., Chen, Z., Zhang, Q. & Guo, C. Dual-catalyzed stereodivergent electrooxidative homocoupling of benzoxazolyl acetate. *J. Am. Chem. Soc.* **146**, 1522–1531 (2024).
4. Pacheco, M. C. & Gouverneur, V. Electrophilic fluorodesilylation of allenylmethylsilanes: a novel entry to 2-fluoro-1,3-dienes. *Org. Lett.* **7**, 1267–1270 (2005).
5. Shan, Q.-C., Hu, L.-M., Qin, W. & Hu, X.-H. Copper-catalyzed cross-nucleophile coupling of β-allenyl silanes with tertiary C–H bonds: a radical approach to branched 1,3-dienes. *Org. Lett.* **23**, 6041–6045 (2021).
6. Lv, Y. et al. Metal-free highly regioselective 1,4-sulfonyliodination of 1,3-enynes. *J. Org. Chem.* **88**, 2034−2045 (2023).
7. Alberti, M. N. & Orfanopoulos, M. The cyclopropyl group as a hypersensitive probe in the singlet oxygen ene reaction mechanism. *Org. Lett.* **10**, 2465–2468 (2008).
8. Roche, C. et al. Synthesis of anti-1,3-diols through $RuCl_3/PPh_3$-mediated hydrogenation of β-hydroxy ketones: an alternative to organoboron reagents. *Eur. J. Org. Chem*. **23**, 3977 (2009).
9. Mizuta, S. et al. Trifluoromethylation of allylsilanes under copper catalysis. *Chem. Eur. J.* **18**, 8583 (2012).
10. Khan, I., Reed-Berendt, B. G., Melen, R. L. & Morrill, L. C. FLP-catalyzed transfer hydrogenation of silyl enol ethers. *Angew. Chem. Int. Ed.* **57**, 12356–12359 (2018).
11. Hayashi, T., Katsuro, Y. & Kumada, M. Nickel-catalyzed cross-coupling of silyl enol ethers with grignard reagents. Regio- and stereocontrolled synthesis of olefins. *Tetrahedron Lett.* **21**, 3915–3918 (1980).
12. Su, Y.-L. et al. Asymmetric α-allylation of aldehydes with alkynes by integrating chiral hydridopalladium and enamine catalysis. *Org. Lett.* **20**, 2403–2406 (2018).
13. Mao, J. et al. Cobalt–bisoxazoline-catalyzed asymmetric Kumada cross-coupling of racemic α-bromo esters with aryl Grignard reagents. *J. Am. Chem. Soc.* **136**, 17662–17668 (2014).
14. Galvin, C. M. & Waymouth, R. M. Electron-rich phenoxyl mediators improve thermodynamic performance of electrocatalytic alcohol oxidation with an Iridium pincer complex. *J. Am. Chem. Soc.* **142**, 19368–19378 (2020).
15. Frisch, M. J. et al. Gaussian 16, Revision C.01. Gaussian Inc, Wallingford CT (2016).
16. Stephens, P. J., Devlin, F. J., Chabalowski, C. F. & Frisch, M. J. Ab initio calculation of vibrational absorption and circular dichroism spectra using density functional force fields. *J. Phys. Chem.* **98**, 11623–11627 (1994).
17. Grimme, S., Ehrlich, S. & Goerigk, L. Effect of the damping function in dispersion corrected density functional theory. *J. Comput. Chem.* **32**, 1456-1465 (2011).
18. Weigend, F. & Ahlrichs, R. Balanced basis sets of split valence, triple zeta valence and quadruple zeta valence quality for H to Rn: Design and assessment of accuracy. *Phys. Chem. Chem. Phys.* **7**, 3297-3305 (2005).
19. Tomasi, J., Mennucci, B. & Cancès, E. The IEF version of the PCM solvation method: an overview of a new method addressed to study molecular solutes at the QM ab initio level. *J.*

Mol. Struct. (THEOCHEM) **464**, 211-226 (1999).
20. Grimme, S. Supramolecular binding thermodynamics by dispersion-corrected density functional theory. *Chem.-Eur. J.* **18**, 9955-9964 (2012).
21. Lu, T. & Chen, Q. Shermo: A general code for calculating molecular thermochemistry properties. *Comput. Theor. Chem.* **1200**, 113249 (2021).
22. Zhao, Y. & Truhlar, D. G. The M06 suite of density functionals for main group thermochemistry, thermochemical kinetics, noncovalent interactions, excited states, and transition elements: two new functionals and systematic testing of four M06-class functionals and 12 other functionals. *Theor. Chem. Acc.* **120**, 215-241 (2008).
23. Grimme, S., Antony. J., Ehrlich. S. & Krieg. H. A consistent and accurate *ab initio* parametrization of density functional dispersion correction (DFT-D) for the 94 elements H-Pu. *J. Chem. Phys.* **132**, 154104 (2010).
24. Marenich, A.V., Cramer, C. J. & Truhlar, D. G. Universal solvation model based on solute electron density and on a continuum model of the solvent defined by the bulk dielectric constant and atomic surface tensions. *J. Phys. Chem. B* **113**, 6378-6396 (2009).
25. Konezny, S. J. et al. Reduction of systematic uncertainty in DFT redox potentials of transition-metal complexes. *J. Phys. Chem. C* **116**, 6349–6356 (2012).
